# ﻿A revision of the South American species of the Morelloid clade (*Solanum* L., Solanaceae)

**DOI:** 10.3897/phytokeys.231.100894

**Published:** 2023-08-29

**Authors:** Sandra Knapp, Tiina Särkinen, Gloria E. Barboza

**Affiliations:** 1 Natural History Museum, Cromwell Road, London SW7 5BD, UK Natural History Museum London United Kingdom; 2 Royal Botanic Garden Edinburgh, 20A Inverleith Row, Edinburgh EH3 5LR, UK Royal Botanic Garden Edinburgh Edinburgh United Kingdom; 3 Instituto Multidisciplinario de Biología Vegetal (CONICET-Universidad Nacional de Córdoba), Casilla de Correo 495, 5000 Córdoba, Argentina Instituto Multidisciplinario de Biología Vegetal (CONICET-Universidad Nacional de Córdoba) Cordoba Argentina

**Keywords:** American tropics, Andes, biodiversity, conservation status, endemism, herbs, South America, Southern Cone, taxonomy

## Abstract

The Morelloid clade, also known as the black nightshades or “Maurella” (Morella), is one of the 10 major clades within the mega-diverse genus *Solanum* L. The clade is most diverse in the central to southern Andes, but species occur around the tropics and subtropics, some extending well into the temperate zone. Plants of the group vary from herbs to short-lived perennials to perennial shrubs that are distinctly woody at the base, they have small mostly white or purplish white flowers and small juicy berries. Due to the complex morphological variation and weedy nature of these plants, coupled with the large number of published synonyms (especially for European taxa), our understanding of species limits and diversity in the Morelloid clade has lagged behind that of other clades in *Solanum*. Here we provide the last in a three-part series of monographic treatments of the morelloid solanums (see PhytoKeys Vols. 106, 125), treating the 62 species occurring in South America. This region is by far the most diverse in the clade, both in terms of species number and morphological diversity. We provide complete synonymy, nomenclatural details, including lecto- and neotypifications where needed, common names and uses, morphological descriptions, illustrations to aid identification both in herbaria and in the field, and distribution maps for all native, non-cultivated species. We include a key to all species, a synoptic character list for the species treated here and links to synoptic online keys for all species of the Morelloid clade. Preliminary conservation assessments following IUCN guidelines are also provided for all native species.

## ﻿Introduction

*Solanum* L., with currently 1,244 accepted species, is one of the largest genera of flowering plants (Frodin 2004; http://www.solanaceaesource.org; http://www.worldfloraonline.org/organisation/Solanaceae). The genus poses a taxonomic challenge not only due to its large size, but also due to the 6,931 published names, many of which are associated with the cultivated and widespread species of the genus, including the potato (*S.tuberosum* L.), tomato (*S.lycopersicum* L.) and the eggplant (*S.melongena* L.). Recent taxonomic and molecular systematic efforts (http://www.solanaceaesource.org) have helped to identify major clades within *Solanum* (e.g., Weese and Bohs 2007), clarify relationships and the monophyly of previously recognised morphological sections (e.g., Stern et al. 2011) and to provide taxonomic revisions for major clades with keys for species identification (e.g., Knapp 2013).

**Figure 1.**
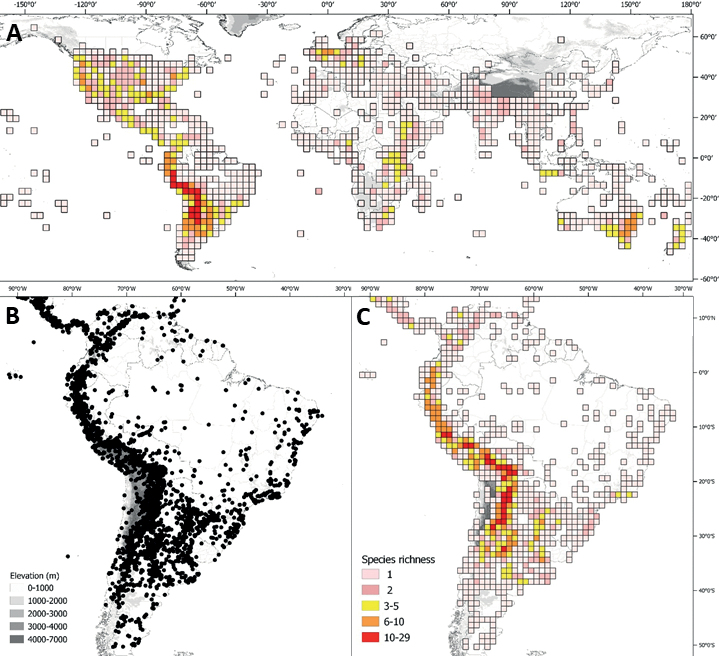
Distribution map of the Morelloid clade of *Solanum***A** species diversity of the clade across the world at 100 km^2^ grid cell resolution (colour legend in subfigure C) **B** georeferenced herbarium specimens studied for South America **C** species diversity in South America, based on the specimens examined for this monograph at 300 km^2^ grid cell resolution.

The Morelloid clade of *Solanum*, also known as the Black nightshades or “Maurella” (Morella), is amongst the 10 robustly supported major clades within *Solanum* (Bohs 2005; Weese and Bohs 2007). This group, which includes the type species of the genus, *S.nigrum* L., has traditionally been considered difficult, due in part to the weedy nature of its species and its worldwide distribution (Fig. 1A). The clade comprises 79 currently accepted non-spiny herbaceous and suffrutescent species with simple or branched hairs with or without glandular tips and inflorescences usually arising from the internodes (Särkinen et al. 2015b). The complex nomenclature of these taxa (662 names for 79 accepted species), especially for extremely widespread taxa such as *S.americanum* and *S.nigrum*, has complicated revisionary work not undertaken on a global level. Ploidy level varies from diploid to hexaploid within the group (e.g., Edmonds and Chweya 1997; Moyetta et al. 2013; Chiarini et al. 2018; Särkinen et al. 2018), also in part contributing to the difficulties in its taxonomy. While taxonomic revisions of the smaller American sections within the morelloids have recently been published (Del Vitto and Petenatti 1999; Barboza 2003; Barboza and Hunziker 2005), the group in its entirety has not been revised since the 19^th^ century (Dunal 1852).

General overviews of black nightshade taxonomy have been published (Edmonds 1977, 1978, 1979a), including geographically focused taxonomic treatments for South America (Edmonds 1972), North America (Schilling 1981) and Africa (Edmonds and Chweya 1997; Olet 2004; Manoko 2007; Edmonds 2012) and detailed cytological and morphological studies (Venkateswarlu and Rao 1972; Edmonds 1982, 1983, 1984). These studies have greatly enhanced our understanding of the complex morphological and ploidy level variation present in the group, but much taxonomic work remains. Revisions of the clade in Africa, Australia, Eurasia and Oceania (Särkinen et al. 2018) and in the Caribbean, North and Central America (Knapp et al. 2019) have clarified the use of names in this group in those areas, where many adventive and widespread taxa are shared. South America, especially the southern central Andes, where more than half of the known species are found (Barboza et al. 2013), is the centre of diversity for morelloids in the Americas (Fig. 1). North and Central America and the Caribbean have a distinctly different complement of species to those from continental South America, although some species with wide distributions span the Americas (e.g., *S.americanum*, *S.nigrescens*). In addition, several species native to Africa, Asia and/or Europe have been introduced or are sporadically cultivated in North America, but are not known from South America (e.g., *S.nigrum*, *S.retroflexum* Dunal).

Here we provide a taxonomic revision of all 62 native and naturalised (or semi-naturalised) species of the Morelloid clade (black nightshades) occurring in South America based on a detailed morphological study. The work presented here is part of our molecular systematic and taxonomic work focusing on producing a global monographic treatment of the Morelloid clade (e.g., Barboza et al. 2013; Särkinen et al. 2013, 2015a, b, 2018; Knapp et al. 2019) across its entire range in a series of geographically focussed works, the first of which treated taxa occurring in Africa, Asia, Australia and the Pacific and Europe (Särkinen et al. 2018), the second of which treated species occurring in North and Central America and the Caribbean (Knapp et al. 2019); this final part of the series treats the area of highest species diversity in the clade, South America and represents the synthesis of our understanding of the species-level taxonomy of this large and seemingly intractable group.

## ﻿History, taxonomy and circumscription of the Morelloid clade

Knowledge of the European species of black nightshades stretches back to the Greeks and Romans (see summary in Särkinen et al. 2018), and perceptions of the toxicity of these plants among European immigrants to the Americas is likely, in part, to have derived from confusion over the identity of *S.nigrum* and *Atropabella-donna* L.

*Solanumnigrum* was the only species of this group treated by Linnaeus (1753). Linnaeus’ circumscription of *S.nigrum* was extremely broad and comprised six infraspecific taxa, many of which were based on the plates in Dillenius’s *Hortus Elthamensis* (Dillenius 1732). He recognised the European *S.nigrum* (as var. vulgare), *S.villosum* Mill. (as var. villosum), and *S.americanum* (as var. patulum), included the African cultivated species *S.scabrum* (as var. guineenense), and recognised the native North American *S.emulans* Raf. (as var. virginicum), but he had not seen material of other species treated here (see individual species treatments for details). He clearly recognised all these taxa as very similar and as variants of a worldwide complex; his diagnosis reads “Habitat in Orbis totius, cultis” [Habitat in all the world, cultivated]. He also noted many of these looked like mixtures (“Tot varietates β, γ, δ, ε, ζ videntur esse hybridae proles”). Linnaeus (1753) did not cite many of the works based on non-European plants (e.g., Piso 1648; Rheede von Draakestein 1689) in *Species plantarum*, despite having previously cited them in *Hortuscliffortianus* (Linnaeus 1737).

Miller (1768), in the sixth edition of his *Gardener’s Dictionary* and the first edition to use Linnaean binomials (see Stearn 1974), described seven members of the Morelloid clade; five of these were coined as new names (*S.villosum*, *S.luteum* Mill., *S.rubrum* Mill., *S.americanum*, and *S.scabrum* Mill.). He did not recognise infraspecific taxa, and when he used Linnaean infraspecific epithets did not indicate he was raising these to species level. He did not include any new American taxa.

Lamarck (1794) recognised seven taxa, including some not known to either Miller or Linnaeus, such as *S.radicans* and *S.corymbosum*, both members of the clade known as the Radicans group (see Särkinen et al. 2015b). He additionally described *S.chenopodioides* Lam., from material said to be from “île de France” (Mauritius; but see species description) and *S.triangulare* Lam. (= *S.americanum*) based on an illustration from Rumphius (1750). Some of these early authors re-used epithets (e.g., *villosum* used by both Miller and Lamarck), but it is not clear whether they were referring to earlier names or not; the principle of priority had not yet become established for botanical naming (Knapp et al. 2004).

Floristic treatments in the 18^th^ and 19^th^ century either did not recognise much diversity in the morelloids (e.g., Ruiz and Pavón 1799) or treated native species either as *S.nigrum* or as multiple infraspecific taxa of *S.nigrum* (e.g., Sendtner 1846). Ruiz and Pavón (1799) included *S.corymbosum* [as *S.cymosum*] in their flora of Peru and Chile as new, and also recorded a “yerba moro” as the European species *S.nigrum*. In his treatment of *Solanum* for *Flora Brasiliensis*Sendtner (1846) treated all morelloids as a complex nested set of names under *S.nigrum*, except for *S.sarrachoides* that he described as distinct. Of the South American species treated here Dunal (1813, 1816) only included *S.chenopodioides*, but as exploration of the diverse forests of Andean South America began and specimens reached European herbaria, he described many more taxa (Dunal 1852). Some authors though continued to question the distinctness of the South American species; Johow (1896) in his study of the flora of the Juan Fernández islands (Chile) suggested that the plants he recognised as *S.furcatum* might be best united with *S.nigrum* (“esta especie es muy afin del *S.nigrum*, con el cual talvez debe unirse”). Botanists continued to describe new morelloid species in the latter part of the 19^th^ century (e.g., Weddell 1857 for the high Andes of Peru and Bolivia; Philippi 1891, 1895 for Chile; Rusby 1896 for Bolivia), but the real explosion of description can be attributed to the German botanist George Bitter (for obituary and complete bibliography see Weber 1928), who worked in Bremen, Göttingen and Berlin in the early part of the 20^th^ century. Bitter coined 103 names at the species level for what are now recognised as morelloid taxa (e.g., Bitter 1912a, 1912b, 1912c, 1913, 1914b, 1916, 1918) and 16 infraspecific names. Of these, 17 are here treated as accepted species (see Contents).

Species continued to be described throughout the 20^th^ century, with floristic treatments for Peru (Macbride 1962), southern Brazil (Smith and Downs 1966; Mentz and Oliveira 2004) and Argentina (Morton 1976; Cabrera 1983 for Prov. Jujuy) adding to and clarifying patterns of diversity in the clade. Edmonds (1972) published a preliminary synopsis of the group for South America that included 17 taxa; her concept of the group excluded any species with branched (dendritic, see Pubescence below) trichomes. Checklists of Ecuador (Short and Knapp 1999), Peru (Brako and Zarucchi 1993), Argentina (Barboza and Romanutti 1999), Bolivia (Nee 2015) and Colombia (Orozco et al. 2022) have all helped clarify distributions and revealed synonymies.

The name for the Morelloid clade is derived from Dunal’s (1813: 119) un-ranked group “Maurella” which included herbaceous or sub-herbaceous species with entire leaves. He included 15 species in his “Maurella”, all of which are still considered members of the Morelloid clade. In his subsequent works (Dunal 1814, 1816) he maintained the group “Maurella”, adding to it taxa described by himself and others, most of which are still considered related (with the exception of *S.quadrangurare* Thunb. = *S.africanum* Mill., a member of the African Non-Spiny clade, see Knapp and Vorontsova 2016). Dumortier (1827) used this group, with a changed spelling to “Morella” for his treatment of the Belgian species. This concept of “Morella” was narrow and included only those species later recognised as members of SolanumsectionSolanum and did not include species now recognised as part of the more broadly defined group (Bohs 2005; Weese and Bohs 2007; Särkinen et al. 2013, 2015b). In the *Prodromus*Dunal (1852) continued to use the name “Morella” with Dumortier’s spelling and erected an entirely new framework for *Solanum* mostly composed of *gradi ambigui* (names of ambiguous rank). Within his “Morella” Dunal (1852) recognised two groups based on inflorescence position, “Morellae spuriae” (6 spp.) and “Morellae verae” (54 spp.). Circumscription of “Morella” remained obscure and loose during most 19^th^ and 20^th^ centuries, with many herbaceous non-spiny taxa treated as members of the group, resulting in the large number of names associated with the Morelloid clade. Many of these names do not belong to the clade as now recognised based on phylogenetic data (Bohs 2005; Weese and Bohs 2007).

Following the rules on the use of autonyms, Seithe (1962) was the first to name as section Solanum the group containing the type species of the genus (*S.nigrum*). She also recognised three other sections now considered as part of the Morelloid clade; sections *Campanulisolanum* Bitter, *Chamaesarachidium* Bitter and *Episarcophyllum* Bitter (all groups confined to South America, see Del Vitto and Petenatti 1999; Barboza 2003; Barboza and Hunziker 2005), now considered part of the larger Morelloid clade (Särkinen et al. 2015b). Danert (1967, 1970) followed her sectional classification with little change in either circumscription or membership. D’Arcy (1972) lectotypified the infrageneric groups and provided an overview of the history of these names across the entire genus *Solanum*.

Child (1994) created two new sections, sect. Solanocharis (Bitter) A.Child comprising *S.albescens* and what he considered its relatives (e.g., *S.rheithrocharis* Bitter [= *S.leptocaulon*], *S.leptocaulon* and *S.poecilochromifolium* Rusby [= *S.gonocladum*]) and sect. Parasolanum A.Child. This latter grouping contained a disparate set of species in part considered related because of their pinnatifid leaves (*S.triflorum* [and its synonyms recognised as distinct], *S.tripartitum*, *S.radicans*, “S. pseudodulcamaroides Schaffer” [= a designation for a Mexican specimen of *S.corymbosum*], *S.palmeri* Vasey & Rose [= *S.umbelliferum* Eschsch. of the Dulcamaroid clade], *S.patagonicum* C.V.Morton [= *S.nitidibaccatum*], *S.annuum*, *S.gilioides*, *S.pygmaeum*, *S.salicifolium* [and its synonyms] and *S.maracayense* Bitter [= *S.pilcomayense*]); the latter three he cited with some hesitation as belonging to his new group. These sections, as is so often the case in *Solanum* (Bohs 2005), do not hold up to closer scrutiny.

Prior to molecular phylogenetic studies, a series of studies based on numerical taxonomy, morphology and crossing experiments were undertaken to understand species relationships, parental origin of polyploids, and species delimitation in the morelloids (Soria and Heiser 1961; Heiser et al. 1965, 1979; Edmonds 1978). The power of these methods has remained limited due to the complex and often overlapping morphological variation between the closely-related species. Species of morelloids show large amounts of morphological variation, especially in growth form, pubescence and leaf morphology.

Within the Morelloids, four well-supported clades have been recognised based on detailed molecular phylogenetic studies (Särkinen et al. 2015b; Gagnon et al. 2022). These clades loosely correspond to the previously recognised morphological sections: 1) the Radicans clade which comprises four species of SolanumsectionParasolanum A.Child (but not including the type species, *S.triflorum*; Bohs 2005), 2) the *Episarcophyllum* clade that includes most species of SolanumsectionEpisarcophyllum Bitter (but not *S.caesium*); 3) the Chamaesarachidium clade that includes two species of SolanumsectionChamaesarachidium Bitter (but not *S.annuum*); and finally the largest and most complex group, 4) the Black nightshade clade, that includes all species of the traditional SolanumsectionSolanum, as well as species not previously associated with what is also known as the *S.nigrum* complex (see below). The first three clades are restricted to the Americas, while the Black nightshade clade has a global distribution with a centre of diversity in the Americas and a secondary centre of diversity in Africa (fig. 1 in Särkinen et al. 2018).

Relationships amongst many species of the Black nightshade clade are complicated by polyploidy (Särkinen et al. 2015b) and the ploidy levels of many of the South American species remain to be assessed (see species descriptions).

The Morelloid clade has been considered difficult, mainly due to the black nightshades (sensu Särkinen et al. 2015b) that comprise many widespread and morphologically variable species, polyploids and many associated names at many taxonomic levels (Särkinen et al. 2015b, 2018; Knapp et al. 2019). Although Edmonds (1972) considered the morelloids to be a species-poor group, our globally focused work with these plants has revealed significant novelty for South America (e.g., Särkinen et al. 2015c, d; Särkinen and Knapp 2016; Knapp and Särkinen 2018; Knapp et al. 2020) as well as significant synonymy, especially for widespread, weedy and cultivated species (e.g., Särkinen et al. 2018; Knapp et al. 2019, 2020).

## ﻿Morphology

### ﻿Habit and stems

**Figure 2.**
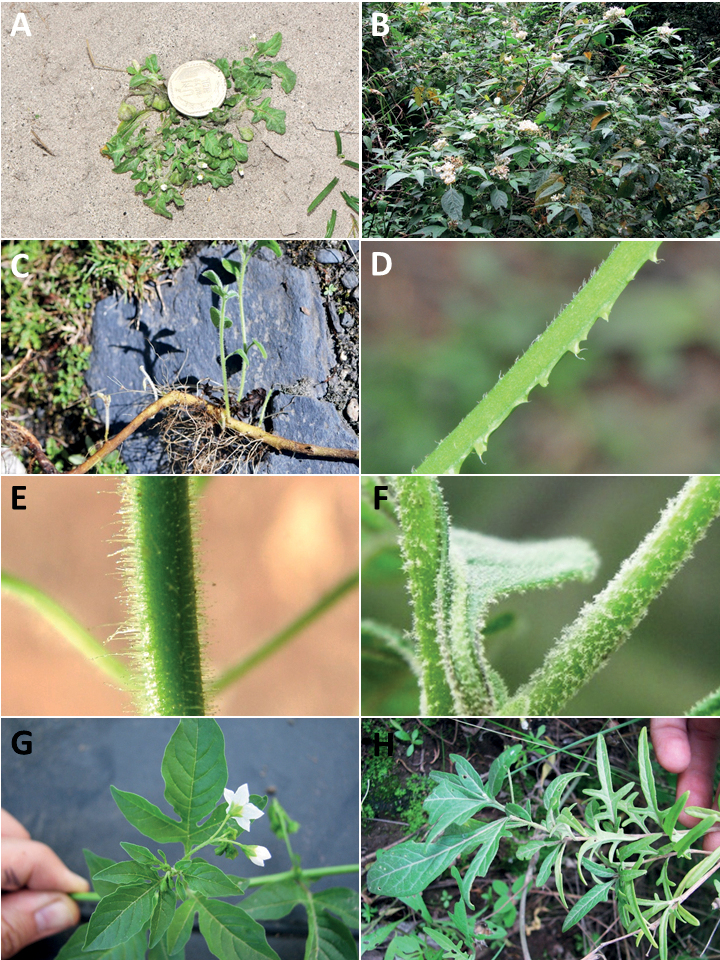
Representative habits and leaves of South American morelloids **A** prostrate annual herbs (*S.weddellii*) **B** large lax shrubs (*S.aloysiifolium*) **C** roots forming along a creeping stem (*S.juninense*) **D** spinose processes on stems of many species (*S.huayavillense*) **E** glandular trichomes found in some members of the clade (*S.glandulosipilosum*) **F** dendritic/branched trichomes found in *S.pallidum***G** regularly 3-lobed leaves in *S.palitans***H** highly variable leaves along a single stem in *S.salicifolium* (**A***Särkinen et al. 4038***B***Barboza et al. 3505***C***Särkinen et al. 4754***D***Barboza et al. 3536***E***Barboza et al. 3520***F***Särkinen et al. 4010***G***Atchison & Gagnon 25***H***Barboza et al. 3473*). Photos by S. Knapp, G. Atchison, and T. Särkinen.

Members of the Morelloid clade are annual to perennial herbs or shrubs, often woody at the base. South American taxa are much more diverse in habit (Fig. 2) than in the rest of the clade’s range (e.g., Särkinen et al. 2018; Knapp et al. 2019). Here, species range from annual herbs (e.g., *S.triflorum*) or large coarse perennial herbs (e.g., *S.pilcomayense*) to short-lived somewhat woody perennials (e.g., *S.arequipense*) to woody shrubs (e.g., *S.gonocladum*). Stems are often weak, and occasionally somewhat scrambling, but can reach 5 m in height (e.g., *S.cochabambense*). Plants of all species usually have herbaceous upper stems, even if the base is woody. The stems can be terete (e.g., *S.americanum*), angled (e.g., *S.antisuyo*) or strongly winged throughout (e.g., *S.marmoratum*); this can be a useful character for identification of herbarium specimens, but note that within species variation is common.

Sympodial growth is characteristic of Solanaceae giving the stems a typical “zig-zag” appearance; details of sympodial structure have proved useful for infrageneric classification within *Solanum* (Child and Lester 1991; Knapp 2002a). Vegetative growth is initially monopodial, but with the onset of flowering, becomes sympodial. The inflorescence is developmentally terminal, and stem continuation is initiated in the axil of the leaf below each inflorescence. Each lateral shoot with alternate leaves arranged in a 1/3 phyllotactic spiral and a terminal inflorescence is termed a sympodial unit. In some cases, when the axes of sympodial units are fused, the inflorescences appear to originate laterally from the middle of an internode; when growth of the axes is suppressed, the leaves appear paired (geminate) at a node (Danert 1958). Further fusion of axes can give rise to inflorescences arising at the node and appearing to be opposite the leaves (Danert 1970). Most members of the Morelloid clade have difoliate sympodial units with leaves usually strongly paired (geminate) at the nodes.

“Spinose” processes are common on herbaceous stems in some species of black nightshades (see Särkinen et al. 2018). They usually occur along the angles of upper parts of larger stems and are often decurrent from leaf bases (Fig. 2D). These are not true prickles, like those found in the “spiny” solanums (Leptostemonum clade, Whalen 1984; Vorontsova and Knapp 2016) but are similar in that they are outgrowths of the epidermis and are usually associated with trichomes as the enlarged basal portions of stem trichomes that have fallen off. They have been used to differentiate species in this group, but these structures are variable within species where they do occur, and even within stems on a single plant. In addition, they often change markedly in appearance when plants are pressed and dried. In South American morelloid species spinose processes are prominent and always present in some species, such as *S.salamancae*, while in others (e.g., *S.huayavillense*) individual plants vary. Their absence, however, can be diagnostic when combined with other characters.

### ﻿Leaves

Species of the Morelloid clade have simple entire, shallowly toothed, deeply 3–5 lobed or deeply pinnatifid leaves that are generally elliptic or ovate in outline (see Fig. 2 and species descriptions). As with other vegetative characters in this group, leaf morphology can be extremely variable within a species or even in a single plant. Basal stem leaves are usually larger than those at the tips, and in some species (e.g., *S.gonocladum*) plants from extremely arid conditions have very small leaves.

Leaf margins vary from entire to deeply sinuate, lobed and pinnatifid. In species with deeply pinnatifid leaves, a wing of leaf blade is always present along the midrib, thus leaves are not strictly pinnate. *Solanumannuum* and *S.salicifolium* have pinnatifid and simple leaves on the same stems (Fig. 2H); other species, such as *S.triflorum*, exhibit considerable variation between plants, from deeply pinnatifid to almost entire leaves. Simple leaves with variously entire or toothed margins are common in the group. In those species with predominantly simple entire leaves at least some specimens have been examined with shallowly toothed leaf margins. In these individuals the teeth often occur only in the basal half to third of the leaf blade. The leaf blade in members of the Morelloid clade is often somewhat decurrent onto the petiole and the leaf base is then cuneate to attenuate. Leaf apices are acute to attenuate but vary considerably within species.

Petiole length to some extent is related to leaf size, and on individual plants larger leaves always have longer petioles. Some species have almost sessile leaves (e.g., *S.gonocladum*, *S.salicifolium*, *S.sinuatirecurvum*, *S.weddellii*) where no abrupt narrowing of the leaf base onto the petiole occurs. The cultivated *S.scabrum* (in South America only known from Brazil) has relatively long petioles relative to leaf size (see Särkinen et al. 2018).

### ﻿Pubescence

Trichomes in species of the Morelloid clade are simple or branched (e.g., *S.pallidum*, Fig. 2F), but never stellate (Seithe 1962). The simple trichomes are usually 1–6-celled and uniseriate; dendritic trichomes are similarly uniseriate and relatively few-celled. Occasionally the trichome base is enlarged with the lowermost cell much larger than more distal cells and these enlarged bases persist as spinose processes on stems (Figs 2D, 149A; sometimes called ‘pseudospines’ by other authors). Much importance has been placed on differences in density of pubescence as a taxonomic character, but pubescence within taxa is continuously variable and apparently also related to environment, with plants growing in sunny sites more densely pubescent.

The presence or absence of glandular trichomes has also been previously treated as taxonomically significant (see Edmonds 1972, 1979b, 1982), with glandular and eglandular morphotypes being treated as separate subspecies or varieties (see Edmonds 2012). Seithe (1962, 1979) showed that in most *Solanum* species glandular trichomes are found on cotyledons and hypocotyls of seedlings and are lost as plants mature; she suggested that species with glandular trichomes were more “primitive”. It is equally probable that the retention of glandular tips on trichomes is a simple paedomorphic character and that it has little taxonomic significance if not correlated with other characteristics. Some species treated here are only occasionally glandular (e.g., *S.triflorum*), with the glandular trichomes very small and sparse (see Subils 1989); in these plants eglandular trichomes dominate. In the species descriptions for this revision we indicate when trichomes are glandular, but if no state is indicated, this means they are eglandular.

Modern developmental work has not been undertaken with morelloid trichomes, but work has been done with the glandular trichomes of tomatoes and their relatives (e.g., Bergau et al. 2015). These studies show that these trichomes play a role in pest defence through release of metabolites in response to insect contact. Local ecological and herbivore pressures may also play a role in the presence or absence of glandular trichomes in the morelloids; this may help explain the highly heterogenous distributions of glandular and eglandular individuals in some morelloid species.

### ﻿Inflorescences

**Figure 3.**
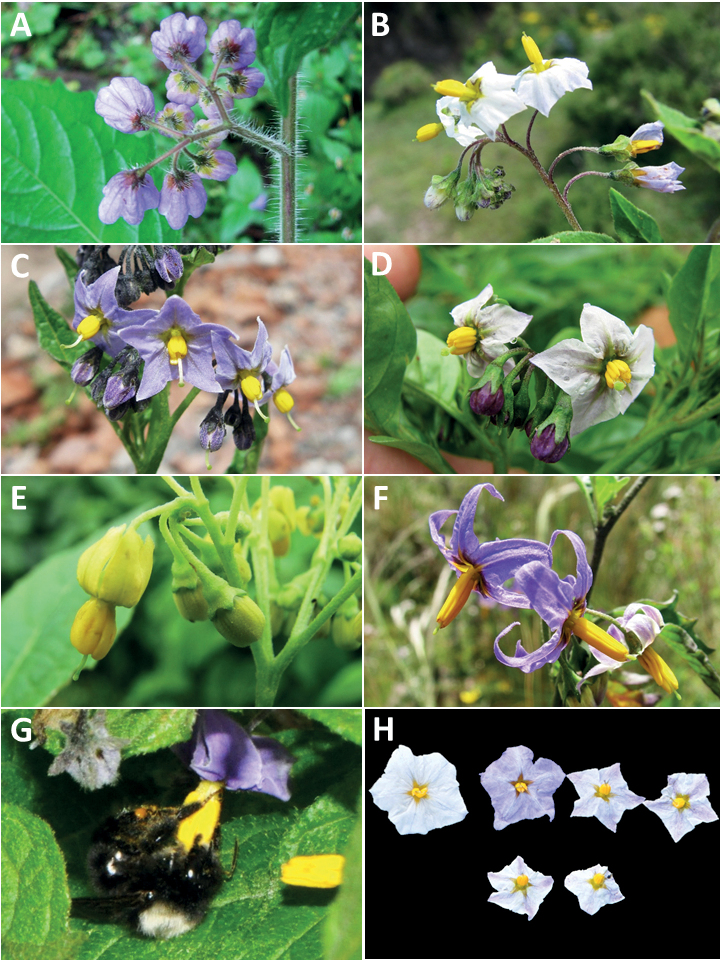
Representative flowers of South American morelloids **A** campanulate purple corollas in forked internodal inflorescences in *S.fiebrigii***B** pentagonal white corollas in *S.annuum***C** broadly stellate purple corollas with long-exerted styles in *S.pentlandii***D** broadly stellate white corollas with slightly exerted globose stigmas in *S.radicans***E** deeply stellate pale yellow corollas in *S.huayavillense***F** deeply stellate purple corollas with dark central eye colouration in *S.salicifolium***G** bumblebee visiting and buzzing the anther cone in *S.cochabambense***H** variation in corolla shape, size and colouration within a single individual of *S.cochabambense* (**A***Barboza et al. 3548***B***Barboza et al. 3495***C***Knapp et al. 10248***D***Knapp et al. 10277***E***Barboza et al. 3531***F***Chiarini et al. 819***G***Särkinen et al. 4036***H***Knapp et al. 10669*). Photos by S. Knapp and T. Särkinen.

The inflorescence of members of the Morelloid clade is developmentally terminal and later overtopped by the leading axillary shoot so that it appears lateral (Fig. 3A); this is the case across the clades of *Solanum* (but see the Pteroidea clade with axillary inflorescences, Knapp and Helgason 1997; Tepe and Bohs 2010). Inflorescences usually arise internodally through axis fusion (Danert 1958, 1967; see above) or appear to arise opposite the geminate leaves (e.g., *S.dianthum*, *S.sarrachoides*) especially on very young shoots (e.g., *S.americanum*). The inflorescences of some species (e.g, *S.riojense*) are terminal with little or no continued shoot growth.

**Figure 4.**
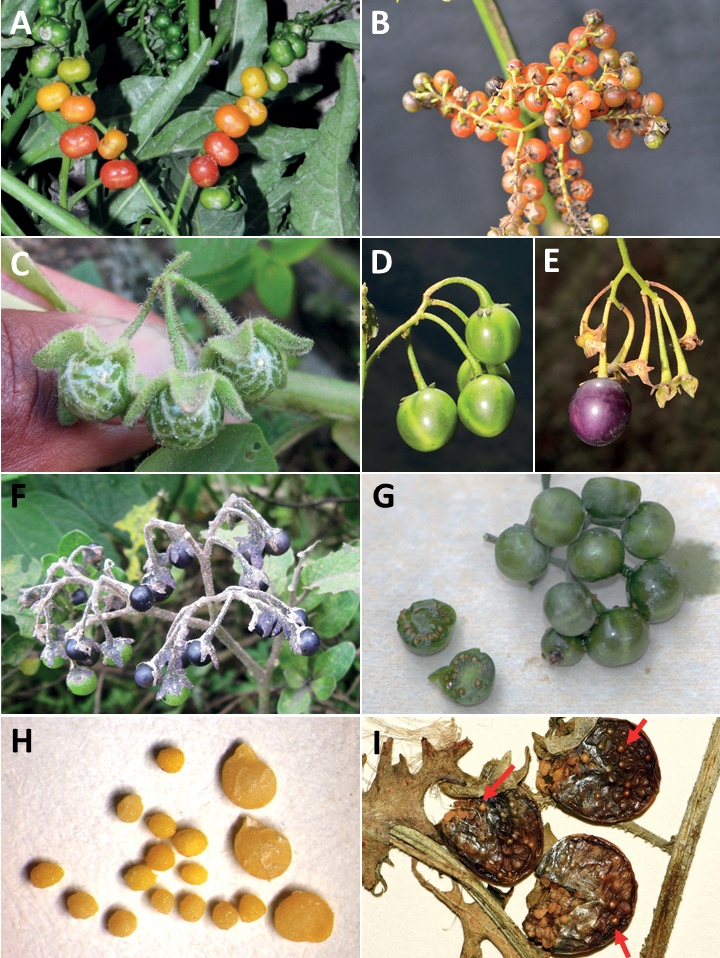
Representative fruits, seeds and stone cells of South American morelloids **A** bilobed fruits in *S.tripartitum* with fully mature red berries at the base of the inflorescence and maturing green, yellow, and orange fruits more distally **B** orange-red fleshy berries of *S.corymbosum* in highly branched inflorescences **C** fully mature marbled fruits in *S.physalifolium***D** immature ellipsoid fruits of *S.antisuyo***E** fully mature fruits of *S.antisuyo***F** immature green fruits amongst fully mature purple-black fruits in *S.cochabambense***G** fully mature fruits of *S.polytrichostylum***H** stone cells (also known as sclerotic granules or brachysclerids) found in the fruits of most species of the Morelloid clade (left side of photo) next to the teardrop shaped seeds (right side of photo; *S.umalilaense* Manoko) **I** stone cells visible in dried fruits of herbarium specimens (*S.triflorum*). (**A***Barboza 3563***B***Särkinen et al. 4604B***C***Knapp et al. 10334***D***Gonzáles 10256***E***Gonzáles 10256***F***Knapp et al. 10363***G***Särkinen et al. 5277***H** Nijmegen accession A24750133 **I***Podlech 8624* BM000848286). Photos by P. Gonzáles, S. Knapp, and T. Särkinen.

The basic inflorescence structure is an unbranched or variously branched scorpioid cyme. Most members of the Morelloid clade have unbranched (simple) or merely forked (once-branched) inflorescences, but some species (e.g., *S.cochabambense*, *S.corymbosum*) have inflorescences that consistently branch more than once (Fig. 4B, F). The degree of branching in some species of the group may also depend upon plant or inflorescence age (e.g., *S.leptocaulon*). In all *Solanum* species the inflorescence expands from the tip producing flowers in a proliferating manner (Lippman et al. 2008).

All members of the group have distinct peduncles, usually somewhat longer than the distal flower-bearing portion, but inflorescence length and flower number vary both between and within species. Many species in the group have condensed cymes, termed “sub-umbellate” inflorescences, where the flower-bearing portion is very short and the pedicels are all very closely spaced and congested at the very tip of the inflorescence (e.g., *S.americanum*, *S.interandinum*). These inflorescences are not true umbels, but are described as such in much previous literature, usually as umbellate or subumbellate cymes (e.g., Edmonds and Chweya 1997; Edmonds 2012). Both peduncles and pedicels usually have pubescence like that of the stems and leaves, or somewhat reduced distally. *Solanumhuayavillense* is unusual in always having a pubescent inflorescence even in plants where the stems and leaves are glabrous.

Pedicels in flower are usually deflexed or spreading, and pedicel position in flower and fruit can be a good species-level character for identification but can be very difficult to see in herbarium specimens. In fruit, pedicels are usually somewhat pendent from the weight of the berry, but are strongly (e.g., *S.gonocladum*, *S.macrotonum*) or weakly (e.g., *S.dianthum*, *S.fiebrigii*) deflexed in some species. Other species have markedly spreading pedicels in fruit (e.g., *S.americanum*). Pedicels in the Morelloid clade have an abscission zone at the very base, and if and when pedicels abscise, the scars are generally flush with the inflorescence axis or sometimes on a tiny, raised stump. Pedicel persistence after fruit ripening and abscission can be important species character in this group (*S.americanum*, *S.antisuyo*), but in South America most species do not have persistent pedicels. In *S.americanum* the ripe berries fall without the pedicels, thus the pedicel is left behind and persists. The presence of old pedicels can be useful for identification of non-flowering herbarium specimens.

### ﻿Calyces

The calyx in all members of the Morelloid clade is 5-merous and synsepalous. The calyx tube is generally conical or occasionally somewhat elongate (e.g., *S.corymbosum*, *S.dianthum*), and the lobes are extremely variable in size and shape from small-deltate and rounded (e.g., *S.antisuyo*) to long-triangular (e.g., *S.glandulosipilosum*, *S.physalidicalyx*, *S.sinuatiexcisum*). The position of the calyx lobes in fruit is an important identification character; they can be strongly reflexed (e.g., *S.americanum*), spreading (e.g., *S.fragile*, *S.grandidentatum*, *S.marmoratum*) or appressed to the berry (e.g., *S.corymbosum*, *S.nigrescens*). The calyces of several species are accrescent in fruit with the calyx lobes expanding to envelop the entire to almost the entire berry (e.g., *S.physalidicalyx*, *S.tweedieanum*). In these species the calyx base in fruit is often invaginate (e.g. *S.gilioides*, Fig. 62H).

### ﻿Corollas

In common with most species of *Solanum*, members of the Morelloid clade have 5-merous sympetalous corollas that are variously lobed (see Fig. 3 for representative corollas in South American species). Floral mutants are often observed, where 4–6-merous corollas can occur on individual plants that are otherwise 5-merous (e.g., *S.americanum*). Colour is generally white or pale violet-tinged (see Fig. 3 and individual species illustrations), but anthocyanin pigmentation can vary depending on environmental growth conditions and in most white-flowered species at least some individuals with purple and violet flowers have been recorded. A single species of the group has pale yellow flowers (*S.huayavillense*). At the base of the corolla tube there can be a ring or irregular area of differently coloured tissue usually referred to as the “eye”; in the species of the Morelloid clade this is usually yellow or greenish yellow but can be dark purple or ringed with blackish purple pigment (Fig. 3D, F, H). This eye is usually similar in texture to the rest of the corolla and not shiny as occurs in the Dulcamaroid clade (Knapp 2013). The colours of the eye usually disappear in herbarium specimens and are rarely noted on labels.

Corollas in the Morelloid clade vary from stellate, deeply stellate, rotate-stellate or pentagonal to occasionally campanulate, and corolla lobes from small-deltate to long-triangular (Fig. 3). Most species have stellate corollas where the lobes and tube are approximately of equal length, and the lobes can be spreading (held horizontally), reflexed or somewhat cupped. *Solanumtriflorum* has deeply stellate corollas, with narrow, reflexed corolla lobes, while *S.corymbosum* has corollas with the lobes approximately the same length as the tubular portion, and the lobes are not strongly reflexed at anthesis. Pentagonal, spreading rotate-stellate corollas with small lobes occur in some species (e.g., *S.annuum*, *S.caesium*, *S.weddellii*); in *S.caesium*, the entire corolla is reflexed at some points during anthesis. A few species have campanulate corollas (Fig. 3A) where the lobes are tiny and never reflexed but rather somewhat cupped (e.g., *S.albescens*, *S.fiebrigii*, *S.leptocaulon*, *S.sinuatiexcisum*); in these species descriptions we have recorded corolla length rather than diameter. These characters, particularly those of the degree to which corolla lobes are reflexed, can be very difficult to see in herbarium specimens. The corolla lobes can be more or less reflexed through the life of the flower as is seen in many other groups of solanums (e.g., Dulcamaroid clade, ANS clade, see Knapp 2013; Knapp and Vorontsova 2016) where flowers last more than one day. Lobes often are spreading on day one, become reflexed to strongly reflexed on subsequent days, and as the flower ages, lobes become spreading again. In some species (e.g., *S.cochabambense*, *S.leptocaulon*) flower size can appear extremely variable within plants (Fig. 3H); this may be due to corolla expansion through the life of the flower as has been demonstrated in *Lycianthes* (Dean 2001; Dean et al. 2020).

Corollas of members of the Morelloid clade are usually very small, as compared to other groups of *Solanum* species; these species have among the tiniest flowers of any *Solanum*. Corolla diameter varies from 4–20 mm; amongst the species treated here *S.marmoratum* has the smallest corollas and *S.albescens* the largest. Adaxial lobe surfaces are usually glabrous, while abaxial corolla lobe surfaces are variously papillate, with longer simple uniseriate trichomes on the margins and tips. Some species have corolla surfaces that are densely puberulent/pubescent where the surfaces are exposed in bud, in these taxa the interpetalar tissue (that is folded within the bud before anthesis) is usually glabrous.

### ﻿Androecium

The stamens of members of the Morelloid clade are mostly equal to very slightly unequal in size and length. In those taxa with slightly unequal stamens the basal-most filament appears to be somewhat longer, but this has not been assessed quantitatively as is the case in other *Solanum* species (Bohs et al. 2007; Aubriot and Knapp 2022). The differences in length are so small that they are unlikely to influence pollinator behaviour as is the case for strongly enantiostylous solanums (e g., *S.rostratum* Dunal of the Androceras clade; see Bowers 1975; Vallejo-Marín 2022). The filament tube and filaments are variously pubescent adaxially. The trichomes on filaments are eglandular, simple and uniseriate; they are usually weak-walled and tangled. The filament tube is generally very short to almost absent and the free portion of the filaments distinct. Filament length in comparison to anther length can be a useful character for distinguishing species. In most species of morelloids treated here the free portion of the filament is about half the anther length, some (e.g., *S.americanum*) have the anthers and filaments of equal length, while other species are characterised by long filaments in relation to anther length (e.g., *S.subtusviolaceum*). The length of filaments can affect the biophysical properties of anther vibration and thus perhaps vibratile pollination (e.g., Timerman et al. 2014; Switzer and Combes 2016), and may be an important characteristic involved in speciation in this group. In some species (e.g., *S.marmoratum*) filaments elongate throughout anthesis, beginning shorter than the anthers and much longer at the end of flowering.

Anthers of members of the Morelloid clade conform to the poricidal morphology of all other species of *Solanum* (Knapp 2001). In common with other “non-spiny” solanums, the anther is ellipsoid and the terminal pore usually “unzips” during anthesis to become an elongate slit. The tapering and somewhat beaked anthers of *S.woodii* (Fig. 185C) are unique in the group; when originally described (Särkinen and Knapp 2016) this species was thought to be closely related to *S.anomalostemon* S.Knapp & M.Nee, an unusual species with heart-shaped anthers and no close relatives (Gagnon et al. 2022). The anthers are loosely connivent, and not connected by either “glue” (as in *S.dulcamara* L., Glover et al. 2004) or elongate papillae (as in the tomatoes, see Peralta et al. 2008). Anther size is an important identification feature in the Morelloid clade, where anthers vary from less than 1 mm (*S.americanum*, *S.marmoratum*) to ca. 5 mm long (*S.dianthum*, *S.gonocladum*, *S.hunzikeri*, *S.tweedieanum*); in such small flowers, small differences can be very important.

### ﻿Gynoecium

The gynoecium in members of the Morelloid clade is bicarpellate; the carpels are fused in a superior ovary with axile placentation. The ovary is glabrous, and usually conical to globose. The flowers lack nectaries, as do all species of *Solanum*. The style is straight (Fig. 3B, E) or slightly curved (Fig. 3C) and usually sparsely to densely pubescent in the lower half to third where it is enclosed in the anther cone. It is usually exserted from the anther cone, but in some species (e.g., most populations of *S.americanum*, *S.corymbosum*, *S.marmoratum*, *S.weddellii*) only barely exceeds the length of the stamens. This may be related to self-fertilisation and thus self-compatibility, as has been observed in the tomatoes (Rick et al. 1977, 1978, 1979; Rick and Tanksley 1981; Peralta et al. 2008), but all species of the Morelloid clade studied so far have been self-compatible (Edmonds 1979a; Schilling and Heiser 1979; Eijlander and Stiekema 1994; Olet 2004). We have observed marked difference in style length and exsertion over the course of anthesis in *S.marmoratum* (see species description) and so have described this character as included, exserted or long-exserted without giving specific measurements, as the detailed floral biology of morelloid species is so poorly known. Flowers collected at different stages of anthesis can be quite disparate and little is known about changes in style length over the course of anthesis for the majority of species.

Species of the Morelloid clade do not have markedly heterostylous flowers. The stigma is either very minutely capitate (e.g., *S.nigrescens*) or larger and more obviously globose-capitate (e.g., *S.corymbosum*, *S.radicans*) and sometimes bilobed (e.g., *S.arequipense*, *S.weddellii*). In Solanaceae the ovules are anatropous and the seeds non-arillate.

### ﻿Fruits

As with all species of *Solanum*, the fruit is a bicarpellate berry. Fruits of members of the Morelloid clade are small (usually less than 1 cm in diameter) and juicy, with thin pericarp that is often shiny (Fig. 4). Most species have globose berries, but those of *S.palitans* are somewhat flattened and those of *S.tripartitum* are conspicuously bilobed (Fig. 4A). Berry colour is usually green, yellowish green or varying shades of purple and purple-black (Fig. 4D–G); immature berries are usually described as green on herbarium labels. The berries of *S.tweedieanum* are almost cream-coloured when ripe and could be described as whitish green. Marbling with white occurs in several species with green mature berries (e.g., *S.marmoratum*, *S.physalifolium*, Fig. 4C); these markings often disappear in herbarium specimens and are not usually recorded on labels. *Solanumpalitans* has yellow translucent berries and *S.corymbosum* and *S.tripartitum* orange to red berries that are more opaque (Figs 4B, 42D, 108D, 176D). Colour polymorphisms are common in species of this group; *Solanumaloysiifolium*, for example, has individuals and populations with green or purple berries. Manoko (2007) showed that berry colour did not differentiate groups within European populations of *S.nigrum*. Despite this variation, berry colour is an important identification aid in this group, but is often not recorded on herbarium labels, especially those of older specimens. Some species of South American morelloids have berries that turn from green to mottled purple or purple-tinged as they ripen (e.g., *S.interandinum*) so berry colour is often difficult to interpret from herbarium labels.

The pericarp (epicarp) of the berries is thin and either matte (e.g., *S.aloysiifolium*, *S.chenopodioides*) or shiny (e.g., *S.americanum*, *S.gonocladum*, *S.physalifolium*). Surface characteristics are useful for species identification, especially when combined with other characters (see discussion of *S.americanum*). The mesocarp is always juicy and very liquid; these fruits are eaten by both birds and mammals (including people). In general, the mesocarp of fresh fruits is green or greenish yellow, but in species with purple berries it is sometimes purplish (*S.americanum*, *S.scabrum*). This character is rarely mentioned on herbarium labels.

Berries of members of the Morelloid clade contain small, hard inclusions commonly referred to as stone cells or brachysclereids (Bitter 1911, 1914a), known also from other groups of non-spiny solanum such as the Pachyphylla clade (ex-*Cyphomandra* Sendtn., Bohs 1998) and the Archaesolanum clade (Symon 1994). These concretions are composed of modified sclerenchyma cells with massively enlarged cell walls (Fig. 4H); the stone cells of pears and quinces (Rosaceae) are classic examples of this cell type. Neither their function nor their origin in Solanaceae is known. Bitter (1914a) suggested that they existed in an evolutionary series in the family, with more “advanced” taxa lacking stone cells altogether (e.g., the spiny solanums). Some members of the Archaesolanum clade have more stone cells than seeds in each berry (e.g., *S.aviculare* G.Forst. with an average of 12–55 seeds and 491–607 stone cells, Symon 1994). Stone cells in the Morelloid clade are usually quite small and are always more or less spherical, ca. 0.5 mm in diameter, and brown to white in colour; they sometimes have irregular surface patterning. Stone cells can usually be easily seen in dried specimens without dissecting the berry (see fig. 1 in Bitter 1914a; Fig. 4I); they appear globose and are often larger than the seeds. Sometimes stone cells of different sizes are found in the same berry, but this character is not consistent within species, except for in the members of the Radicans clade where there are consistently two large apical inclusions and varying numbers of smaller stone cells scattered in the berry flesh. The number of stone cells is usually relatively consistent within a species, and varies from absent (e.g., *S.annuum*, *S.chenopodioides*, *S.fragile*, *S.scabrum*) to (1)2–4(-6) (e.g., *S.arequipense*, *S.macrotonum*, *S.nigrescens*) to more than 10 (e.g., *S.echegarayi*, *S.furcatum*, *S.triflorum*). *Solanumamericanum* varies from having 0 to 4 stone cells per berry. Bitter (1914a) reported that in crosses involving Morelloid species with and without stone cells hybrid progeny had stone cells present in the fruit, indicating that this was an inherited character. Cultivated species (e.g., *S.scabrum*) tend to lack stone cells; this may be related to human-mediated selection.

### ﻿Seeds

Members of the Morelloid clade usually have flattened seeds, like many other solanums. Seed shape varies from rounded to reniform and markedly kidney-shaped. Most species have teardrop shaped seeds, with the hilum and micropyle at one of the short ends of the seed (Fig. 4H), which is unusual as most species of *Solanum* have reniform seeds. Seed size varies from 1–3 mm long, in general polyploid species have larger seeds than diploids (e.g., *S.americanum* seed size is 1–1.5 mm, while that of *S.scabrum* is 2–2.8 mm). Seed number per berry in the Morelloid clade is generally quite high, with usually 30–50 seeds in each berry, but *S.annuum* consistently has only one or two seeds per berry.

Seed coat morphology has been suggested as a useful character for species-level taxonomy in *Solanum* (Souèges 1907; Lester and Durrands 1984) and has been useful in delimiting groups in some clades (e.g., Geminata clade, Knapp 2002a). Most of the species treated here have sinuate-walled (digitate) testal cells. The lateral walls of these cells of the outer epidermal layer develop lignified radial thickenings that form as hair-like structures (Souèges 1907; Lester and Durrands 1984; Peralta et al. 2008). When the outer wall of the epidermis is removed, either naturally (e.g., by passage through frugivore guts, see Barnea et al. 1990) or by enzymatic digestion (Lester and Durrands 1984; Knapp 2002a) seeds appear pubescent; seed measurements here include these projections. Edmonds (1983) examined seed coat patterns in some members of the Morelloid clade (as SolanumsectionSolanum) and found no useful variation for delimiting either species or species groups. *Solanum.annuum*, *S.gilioides* and *S.weddellii* (previously recognised as section Chamaesarachidium; Barboza 2003) have tuberculate seeds (Fig. 18M), a character state found nowhere else in the Morelloid clade (interestingly, tuberculate seeds are also found in desert-dwelling species of the unrelated spiny Androceras clade; Whalen 1979).

### ﻿Chromosomes

Chromosome numbers in the Morelloid clade are variations on the base number of 12 (Table 1); most species are diploid, but several tetraploid species of uncertain parentage are found in the clade. The chromosomes are very small; median, submedian and subterminal centromeres have been reported (Bhiravamurty 1975). The Morelloid clade, along with the potatoes, is one of the few lineages in *Solanum* where polyploidy is common (see discussion of polyploidy and hybridisation in Chiarini et al. 2018; Särkinen et al. 2018). Polyploidy is common in the members of the group found outside the Americas, but apparently less so in South American species. Variation in ploidy level within a species is not common in *Solanum*, but some species appear to have populations with different chromosome numbers (e.g., *S.interandinum*, *S.macrotonum*, see species descriptions); similar variation occurs elsewhere in *Solanum* in the potatoes (see Spooner et al. 2014). Genome sizes in unreplicated gametic nuclei (C-values) of morelloid species vary between 0.60 pg in *S.tripartitum* and 3.10 pg in *S.nigrum* (Bennett and Leitch 2019).

**Table 1.** Chromosome numbers of South American species of the Morelloid clade; n refers to haploid counts, 2n refers to diploid counts, -- indicates no vouchered chromosome count available. For discussion, references and vouchers see individual species treatments.

Species	Chromosome number
*Solanumalbescens* (Britton) Hunz.	–
*Solanumalliariifolium* M.Nee & Särkinen	–
*Solanumaloysiifolium* Dunal	n = 12
*Solanumamericanum* Mill.	n = 12
*Solanumannuum* C.V.Morton	n = 12
*Solanumantisuyo* Särkinen & S.Knapp	–
*Solanumarequipense* Bitter	2n = 48
*Solanumarenicola* Särkinen & P.Gonzáles	–
*Solanumcaatingae* S.Knapp & Särkinen	–
*Solanumcaesium* Griseb.	–
*Solanumchenopodioides* Lam.	n = 12
*Solanumcochabambense* Bitter	n = 12
*Solanumcorymbosum* Jacq.	2n = 24
*Solanumdianthum* Rusby	–
*Solanumechegarayi* Hieron.	n = 12
*Solanumenantiophyllanthum* Bitter	–
*Solanumfiebrigii* Bitter	2n = 24
*Solanumfragile* Wedd.	2n = 48
*Solanumfurcatum* Dunal	2n = 72
*Solanumgilioides* Rusby	–
*Solanumglandulosipilosum* Bitter	n = 12
*Solanumgonocladum* Dunal	–
*Solanumgrandidentatum* Phil.	2n = 24
*Solanumhuayavillense* Del Vitto & Peten.	–
*Solanumhunzikeri* Chiarini & Cantero	–
*Solanuminterandinum* Bitter	n = 12; n = 24; n = 48
*Solanumjuninense* Bitter	2n = 24
*Solanumleptocaulon* Van Heurck & Müll.-Arg.	–
*Solanumlongifilamentum* Särkinen & P.Gonzáles	–
*Solanummacrotonum* Bitter	2n = 24; n = 36
*Solanummarmoratum* Barboza & S.Knapp	–
*Solanummichaelis* Särkinen & S.Knapp	–
*Solanumnigrescens* M.Martens & Galeotti	n = 12
*Solanumnitidibaccatum* Bitter	n = 12
*Solanumpalitans* C.V.Morton	n = 12
*Solanumpallidum* Rusby	2n = 24
*Solanumpaucidens* Bitter	–
*Solanumpentlandii* Dunal	2n = 24
*Solanumphysalidicalyx* Bitter	–
*Solanumphysalifolium* Rusby	–
*Solanumpilcomayense* Morong	n = 12
*Solanumpolytrichostylum* Bitter	2n = 24
*Solanumprofusum* C.V.Morton	–
*Solanumpseudoamericanum* Särkinen, P.Gonzáles & S.Knapp	2n = 24
*Solanumpygmaeum* Cav.	n = 12
*Solanumradicans* L.f.	2n = 24
*Solanumrhizomatum* Särkinen & M.Nee	–
*Solanumriojense* Bitter	n = 12
*Solanumsalamancae* Hunz. & Barboza	–
*Solanumsalicifolium* Phil.	n = 12
*Solanumsarrachoides* Sendtn.	–
*Solanumscabrum* Mill.	–
*Solanumsinuatiexcisum* Bitter	–
*Solanumsinuatirecurvum* Bitter	n = 12
*Solanumsubtusviolaceum* Bitter	–
*Solanumtiinae* Barboza & S.Knapp	n = 12
*Solanumtriflorum* Nutt.	–
*Solanumtripartitum* Dunal	–
*Solanumtweedieanum* Hook.	n = 12
*Solanumweddellii* Phil.	2n = 24
*Solanumwoodii* Särkinen & S.Knapp	–
*Solanumzuloagae* Cabrera	n = 12

Many chromosome counts are reported for members of this group, often as unvouchered counts of “Solanumnigrum”. In the species treatments we only report counts that are based on our own counts or those that are vouchered and for which we have verified the specimen in question. Many of the numbers reported in previous publications (e.g., Edmonds 1972, 1977, 1978b, 1986; Edmonds and Chweya 1997) are based on plants grown from wild collected seed, but we have not been able to trace the vouchers and so these are not reported here. Where the count based on a voucher that we have not located for verification is the only one for a species we indicate by citing the count as “reported as”. We report chromosome numbers as they are presented in the publications – either as meiotic counts (n) or mitotic counts (2n).

## ﻿Biology and natural history

### ﻿Habitats and distribution

**Figure 5.**
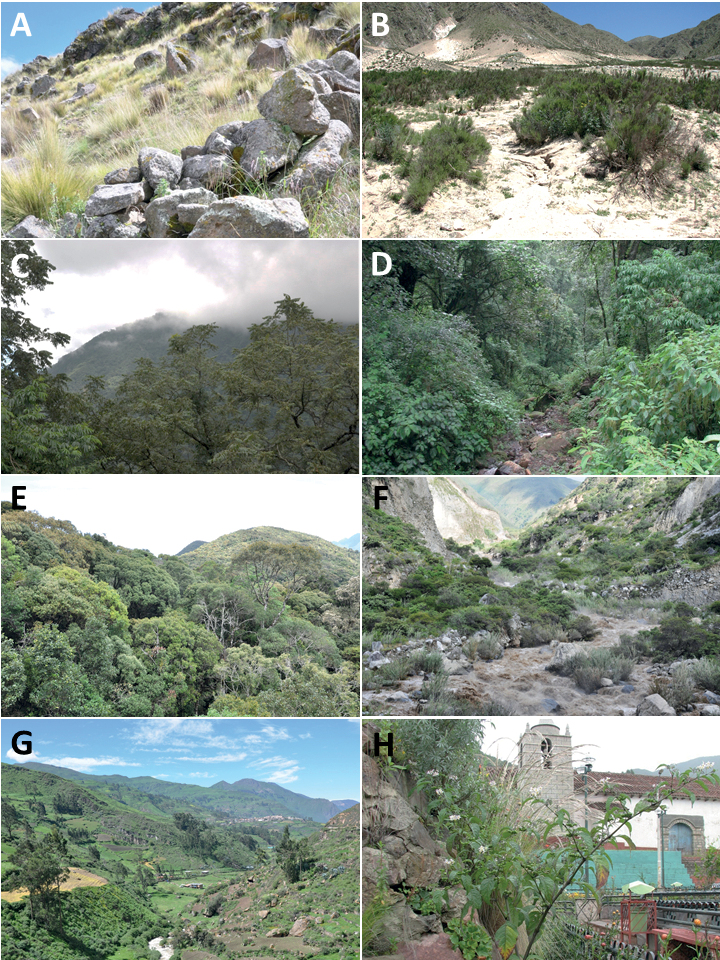
Representative habitats of morelloid solanums in South America **A** rocky areas in Puna grassland in Toccto, Prov. Huamanga (Ayacucho, Peru) at ca. 4,000 m elevation (*S.fragile*) **B** sandy habitats near Quebrada de Randolfo in Dpto. Belén (Catamarca, Argentina) at ca. 2,800 m elevation (*S.weddellii*) **C** andean montane cloud forest (Yungas) in Abra de las Cañas, Parque Nacional Calilegua (Jujuy, Argentina), at ca. 1,600 m elevation (*S.huayavillense*) **D** moist ravine in moist montane forest at Abra Colorada in Dpto. Ledesma (Jujuy, Argentina) near Parque Nacional Calilegua (*S.fiebrigii*) **E** seasonal ombrophyllous forest (mata Atlântica) in Parque Nacional do Itatiaia (Rio de Janeiro, Brazil) at ca. 2,100 m elevation (*S.enantiophyllanthum*) **F** dry river gorge in seasonally dry tropical forest near Abancay (Cusco, Peru) at ca. 2,000 m elevation (*S.physalifolium*) **G** disturbed Andean montane village landscape with agriculture near Canta (Lima, Peru) at ca. 2,800 m elevation (*S.arequipense*, *S.pseudoamericanum* and *S.radicans*) **H** disturbed urban area in the town of Vischongo, Prov. Vilcashuamán (Ayacucho, Peru) at ca. 3,200 m elevation (*S.polytrichostylum*) (**A***Knapp et al. 10259***B***Barboza et al. 3475***C***Barboza et al. 3536***D***Barboza et al. 3548***E***Giacomin et al. 2036***F***Knapp et al. 10334***G***Gonzáles et al. 2875-2877***H***Knapp et al. 10279*). Photos by S. Knapp and T. Särkinen.

Members of the Morelloid clade are usually plants of disturbed habitats and occur in landslides, along roads and streams, and at the edges of cultivated fields (Fig. 5). Many of the species have broad elevational ranges (e.g., *S.americanum*) and extremely broad geographical distributions (see Tables 2, 3). Species diversity in South America is highest in the central and southern central Andes (see Fig. 1C); Bolivia is home to 41 species and Argentina 38 species.

**Table 2.** South American species of the Morelloid clade with their country distributions; country-level endemics are in bold face type (for details of extra-south American distribution of these species see Särkinen et al. 2018 and Knapp et al. 2019).

Species	Country-level distribution
***Solanumalbescens* (Britton) Hunz.**	Bolivia
***Solanumalliariifolium* M.Nee & Särkinen**	Bolivia
*Solanumaloysiifolium* Dunal	Argentina, Bolivia
*Solanumamericanum* Mill.	Argentina, Brazil, Bolivia, Colombia, Chile (?), Ecuador, French Guiana, Guyana, Paraguay, Peru, Suriname, Uruguay, Venezuela (also adventive or native worldwide)
***Solanumannuum* C.V.Morton**	Argentina
*Solanumantisuyo* Särkinen & S.Knapp	Bolivia, Ecuador, Peru
***Solanumarequipense* Bitter**	Peru
*Solanumarenicola* Särkinen & P.Gonzáles	Bolivia, Peru
***Solanumcaatingae* S.Knapp & Särkinen**	Brazil
*Solanumcaesium* Griseb.	Argentina, Bolivia
*Solanumchenopodioides* Lam.	Argentina, Brazil, Paraguay, Uruguay (adventive worldwide)
*Solanumcochabambense* Bitter	Argentina, Bolivia, Peru
*Solanumcorymbosum* Jacq.	Peru (also probably introduced in Mexico)
*Solanumdianthum* Rusby	Bolivia, Peru
*Solanumechegarayi* Hieron.	Argentina, Chile
***Solanumenantiophyllanthum* Bitter**	Brazil
*Solanumfiebrigii* Bitter	Argentina, Bolivia, Peru
*Solanumfragile* Wedd.	Argentina, Bolivia, Peru
*Solanumfurcatum* Dunal	Argentina, Chile
*Solanumgilioides* Rusby	Argentina, Bolivia
*Solanumglandulosipilosum* Bitter	Argentina, Bolivia
*Solanumgonocladum* Dunal	Bolivia, Chile, Peru
*Solanumgrandidentatum* Phil.	Argentina, Bolivia, Chile, Ecuador, Peru
*Solanumhuayavillense* Del Vitto & Peten.	Argentina, Bolivia
*Solanumhunzikeri* Chiarini & Cantero	Argentina, Bolivia
*Solanuminterandinum* Bitter	Colombia, Bolivia, Ecuador, Peru, Venezuela
*Solanumjuninense* Bitter	Bolivia, Peru
*Solanumleptocaulon* Van Heurck & Müll.-Arg.	Bolivia, Peru
*Solanumlongifilamentum* Särkinen & P.Gonzáles	Bolivia, Ecuador, Peru
*Solanummacrotonum* Bitter	Colombia, Ecuador, Venezuela (also Central America and the Caribbean)
***Solanummarmoratum* Barboza & S.Knapp**	Argentina
*Solanummichaelis* Särkinen & S.Knapp	Argentina, Bolivia, Paraguay
*Solanumnigrescens* M.Martens & Galeotti	Colombia, Ecuador, French Guiana, Guyana, Suriname, Venezuela (also North and Central America and the Caribbean)
*Solanumnitidibaccatum* Bitter	Argentina, Chile, Ecuador(?), Peru (introduced and weedy worldwide)
*Solanumpalitans* C.V.Morton	Argentina, Bolivia (introduced to Australia)
*Solanumpallidum* Rusby	Bolivia, Peru
*Solanumpaucidens* Bitter	Argentina, Brazil, Paraguay
*Solanumpentlandii* Dunal	Bolivia, Peru
*Solanumphysalidicalyx* Bitter	Argentina, Bolivia
*Solanumphysalifolium* Rusby	Argentina, Bolivia, Peru
*Solanumpilcomayense* Morong	Argentina, Bolivia, Brazil, Paraguay
*Solanumpolytrichostylum* Bitter	Bolivia, Peru
***Solanumprofusum* C.V.Morton**	Argentina
*Solanumpseudoamericanum* Särkinen, P.Gonzáles & S.Knapp	Bolivia, Ecuador, Peru
*Solanumpygmaeum* Cav.	Argentina, Chile (?) (introduced in Europe and Australia)
*Solanumradicans* L.f.	Bolivia, Chile, Ecuador, Peru
***Solanumrhizomatum* Särkinen & M.Nee**	Bolivia
***Solanumriojense* Bitter**	Argentina
***Solanumsalamancae* Hunz. & Barboza**	Argentina
*Solanumsalicifolium* Phil.	Argentina, Paraguay
*Solanumsarrachoides* Sendtn.	Argentina, Brazil, Paraguay, Uruguay (introduced sporadically in temperate zones worldwide)
*Solanumscabrum* Mill.	Brazil (native to Africa, introduced as a food crop)
*Solanumsinuatiexcisum* Bitter	Argentina, Bolivia, Peru
*Solanumsinuatirecurvum* Bitter	Argentina, Bolivia, Chile
*Solanumsubtusviolaceum* Bitter	Bolivia, Peru
***Solanumtiinae* Barboza & S.Knapp**	Argentina
*Solanumtriflorum* Nutt.	Argentina, Bolivia (also North America, and introduced elsewhere)
*Solanumtripartitum* Dunal	Argentina, Bolivia
*Solanumtweedieanum* Hook.	Argentina, Bolivia
*Solanumweddellii* Phil	Argentina, Bolivia, Chile, Peru
*Solanumwoodii* Särkinen & S.Knapp	Argentina, Bolivia
*Solanumzuloagae* Cabrera	Argentina, Bolivia

**Table 3.** Distribution of species of the Morelloid clade in South America by country. Country-level endemics in bold; introduced species are in parentheses.

Country	Species
Argentina	aloysiifolium, americanum, **annuum**, caesium, chenopodioides, cochabambense, echegarayi, fiebrigii, fragile, furcatum, gilioides, glandulosipilosum, grandidentatum, huayavillense, hunzikeri, **marmoratum**, michaelis, nitidibaccatum, palitans, paucidens, physalidicalyx, physalifolium, pilcomayense, **profusum**, pygmaeum, **riojense**, **salamancae**, salicifolium, sarrachoides, sinuatiexcisum, sinuatirecurvum, **tiinae**, triflorum, tripartitum, tweedieanum, weddellii, woodii, zuloagae
Bolivia	**albescens**, **alliariifolium**, aloysiifolium, americanum, antisuyo, arenicola, caesium, cochabambense, dianthum, fiebrigii, fragile, gilioides, glandulosipilosum, gonocladum, grandidentatum, huayavillense, hunzikeri, interandinum, juninense, leptocaulon, longifilamentum, michaelis, palitans, pallidum, pentlandii, physalidicalyx, physalifolium, pilcomayense, polytrichostylum, pseudoamericanum, radicans, **rhizomatum**, sinuatiexcisum, sinuatirecurvum, subtusviolaceum, tripartitum, triflorum, tweedieanum, weddellii, woodii, zuloagae
Brazil	americanum, **caatingae**, chenopodioides, **enantiophyllanthum**, paucidens, pilcomayense, sarrachoides, (scabrum)
Chile	americanum, echegarayi, furcatum, gonocladum, grandidentatum, nitidibaccatum, pygmaeum (?), radicans, sinuatirecurvum, weddellii
Colombia	americanum, interandinum, macrotonum, nigrescens
Ecuador	americanum, antisuyo, grandidentatum, interandinum, longifilamentum, macrotonum, nigrescens, (nitidibaccatum), pseudoamericanum, radicans
French Guiana	americanum, nigrescens
Guyana	americanum, nigrescens
Paraguay	americanum, chenopodioides, michaelis, paucidens, pilcomayense, salicifolium, sarrachoides, tweedieanum
Peru	americanum, antisuyo, **arequipense**, arenicola, cochabambense, corymbosum, dianthum, fiebrigii, fragile, gonocladum, grandidentatum, interandinum, juninense, leptocaulon, longifilamentum, nitidibaccatum, pallidum, pentlandii, physalifolium, polytrichostylum, pseudoamericanum, radicans, sinuatiexcisum, subtusviolaceum, weddellii
Suriname	americanum, nigrescens
Uruguay	americanum, chenopodioides, pygmaeum, sarrachoides
Venezuela	americanum, interandinum, macrotonum, nigrescens

The largest number of endemic species is found in Argentina (six endemic of 38 species, see Table 2) followed by Bolivia (three endemic of 41 species) and Brazil (two endemic of eight species) and Peru (a single endemic of 25 species). Endemic species in Argentina occupy a wide range of habitats, from the low elevation pampas in the centre of country (*S.marmoratum*) to the high Andes (*S.riojense*), but most are from the Andean foothills, often in the drier habitats defined as prepuna (Cabrera 1976). The high diversity in Bolivia is due to sharing of species from both the south and north; Bolivia shares 16 species with Argentina but not with Peru, and 14 species with Peru and Ecuador but not with Argentina. Eight species of morelloids are found across all three countries (Argentina, Bolivia and Peru). We list the status and general distribution of the species in the group in Table 2, and in Table 3 document country distribution from herbarium specimens (see Materials and methods).

The NOA (“nor-oeste-Argentina”, sensu Aagensen et al. 2012), where Argentina borders Bolivia, is home to many of the endemic and semi-endemic species treated here. This area of endemism is composed of several overlapping more discrete areas of endemism in northern Argentina, such as the so-called Jujuy, Tucumán and Jujuy-Tucumán areas of endemism (sensu Aagensen et al. 2012). Several species previously considered as Argentine endemics (e.g., *S.huayavillense*, *S.hunzikeri*, *S.zuloagae*) have only recently been collected in southwestern Bolivia, confirming the suggestion of Aagensen et al. (2012) that the northern boundary of their NOA (border of Argentina and Bolivia) could change with future collecting. The 16 shared species between Bolivia and Argentina all occur within this previously defined area of endemism.

In contrast to our findings from North America, Europe and Australia (see Särkinen et al. 2018; Knapp et al. 2019), adventive species are rare to non-existent in South America. The single clearly introduced species in South America is *S.scabrum*, only known to us from two old collections (see species description). It is likely that this plant, widely cultivated in Africa for both its leaves and fruits, was brought to Brazil by enslaved Africans during the 19^th^ century. Unlike *S.aethiopicum* L. (“Gilo” an African species of the Leptostemonum Clade, see Vorontsova and Knapp 2016), whose use is widespread in Brazil today, *S.scabrum* appears not to have persisted in cultivation.

South America appears to have been a source, rather than a sink, of adventive species; several South American members of the group (e.g., *S.nigrescens*, *S.nitidibaccatum*) are registered as noxious weeds of agriculture (see below) in both Europe and North America (Ogg et al. 1981; Rogers and Ogg 1981; Defelice 2003; Orgeron et al. 2018). *Solanumtriflorum* is listed as a declared weed in Tasmania (Weed Management Act 1999 2000).

### ﻿Pollination and dispersal

Like most solanums, flowers of members of the Morelloid clade are buzz-pollinated by bees (Buchmann et al. 1977; De Luca and Vallejo-Marín 2013). Females of solitary bees and bumblebees vibrate the anthers with their indirect flight muscles causing pollen to “squirt” out of the terminal pores; they curl their bodies over the anther cone (Fig. 3G) and rotate around the flower (Buchmann et al. 1977). Bees then groom the pollen from their bodies and pack it into their corbiculae (hollow areas on the hind legs), but they cannot reach the area of the venter that contacts the stigma of the next flower and so pollen is transferred from flower to flower. Smaller bees visit and buzz individual anthers (Symon 1979), but do not usually contact the stigma and thus in solanums with large flowers these small bees are more properly seen as pollen thieves. Some bees also exhibit “milking” behaviour, where insects grasp the lower part of the anthers and try to force pollen out of the apical pores using upwards pressure (Buchmann et al. 1977). “Gleaning” of loose pollen grains is also done by various small bees and flies (Symon 1979; Knapp 1986). Buchmann et al. (1977) studied the morelloid *S.douglasii* Dunal in the southwestern United Sates where flowers were visited and buzzed by a wide range of bees in various families, but no more recent pollination studies have been carried out in this group.

The juicy berries with thin pericarp (skins) of members of the Morelloid clade that are typical of bird-dispersed fruits (Knapp 2002b). Studies of dispersal of morelloid species have mostly been done on those occurring in the USA with native bird and mammal frugivores (quail, American robins and deer mice; Tamboia et al. 1996). Green fruits are expected to be more attractive to mammals, but Tamboia et al. (1996) found that both birds and mammal preferred the purple berries of *S.americanum* to the green berries of *S.sarrachoides* (probably = *S.nitidibaccatum*, no vouchers cited). The suite of characters expected to be attractive to mammals such as green colour, odour, and abscission shortly after ripening are all found in some of the morelloids, suggesting that mammals may be important fruit dispersers for these plants as well.

Glycoalkaloid concentrations are very low in ripe berries of *S.americanum* and other members of the Morelloid clade that have been tested (Cipollini et al. 2002), and levels are similar across the clade. Higher concentrations in unripe fruit (Cipollini et al. 2002) of these species make them unattractive to frugivores (Cipollini and Levey 1997a). This loss of secondary metabolites in ripe berries is common across *Solanum* species with brightly coloured, fleshy fruits (e.g., Bradley et al. 1979) and is most likely related to fruit persistence (Cipollini and Levey 1997b), where risk of fungal infection is balanced by probability of animal ingestion and thus dispersal. Glycoalkaloids are known to have a constipating effect (see above, e.g., Gerard 1597) and to inhibit seed germination after ingestion (Cipollini and Levey 1997b), but Wahaj et al. (1998) found that ripe berries of *S.americanum* had a laxative effect on birds thus speeding seed passage through the gut. They suggested this was due to some other chemical compound (perhaps calystegines (?), see Dräger et al. 1994).

### ﻿Conservation status

Preliminary conservation assessments for all species of the Morelloid clade treated here (including introduced taxa) are presented in Table 4. Many of these species can be assigned the status of Least Concern; we have based our assessments primarily on Extent of Occurrence (EOO), because Area of Occurrence (AOO) is highly influenced by collection effort or georeferencing deficit but see individual species treatments for discussion.

**Table 4.** Preliminary threat assessments for morelloid species in South America following IUCN Red Listing (IUCN 2022) guidelines. For details see individual species treatments.

Species	EOO (km^2^)	AOO (km^2^)	Preliminary threat status
*Solanumalbescens* (Britton) Hunz.	7,953	20	EN
*Solanumalliariifolium* M.Nee & Särkinen	18,992	56	VU
*Solanumaloysiifolium* Dunal	1,349,765	1,596	LC
*Solanumamericanum* Mill.	89,639,763	9,828	LC
*Solanumannuum* C.V.Morton	25,287	72	VU
*Solanumantisuyo* Särkinen & S.Knapp	1,089,690	400	LC
*Solanumarequipense* Bitter	748,101	164	LC
*Solanumarenicola* Särkinen & P.Gonzáles	255,276	224	LC
*Solanumcaatingae* S.Knapp & Särkinen	267,575	32	LC
*Solanumcaesium* Griseb.	117,146	184	LC
*Solanumchenopodioides* Lam.	95,008,211	1,560	LC
*Solanumcochabambense* Bitter	7,244,968	1,132	LC
*Solanumcorymbosum* Jacq. (excl. Mexico, Juan Fernández islands)	338,062	240	LC
*Solanumdianthum* Rusby	79,792	188	LC
*Solanumechegarayi* Hieron.	352,787	408	LC
*Solanumenantiophyllanthum* Bitter	14,689	92	VU
*Solanumfiebrigii* Bitter	1,079,092	356	LC
*Solanumfragile* Wedd.	338,395	176	LC
*Solanumfurcatum* Dunal (excl. range in North America, Australia)	342,557	168	LC
*Solanumgilioides* Rusby	139,358	64	LC
*Solanumglandulosipilosum* Bitter	269,652	140	LC
*Solanumgonocladum* Dunal	541,223	284	LC
*Solanumgrandidentatum* Phil.	1,114,912	300	LC
*Solanumhuayavillense* Del Vitto & Peten.	80,000	92	LC
*Solanumhunzikeri* Chiarini & Cantero	97,182	84	NT
*Solanuminterandinum* Bitter	13,454,357	1,148	LC
*Solanumjuninense* Bitter	197,081	120	LC
*Solanumleptocaulon* Van Heurck & Müll.-Arg.	66,386	120	LC
*Solanumlongifilamentum* Särkinen & P.Gonzáles	1,008,132	468	LC
*Solanummacrotonum* Bitter	4,218,133	936	LC
*Solanummarmoratum* Barboza & S.Knapp	266,502	100	LC
*Solanummichaelis* Särkinen & S.Knapp	163,888	48	LC
*Solanumnigrescens* M.Martens & Galeotti	21,536,739	4,280	LC
*Solanumnitidibaccatum* Bitter	188,100,484	1,824	LC
*Solanumpalitans* C.V.Morton	1,039,251	436	LC
*Solanumpallidum* Rusby	140,455	340	LC
*Solanumpaucidens* Bitter	1,233,243	196	LC
*Solanumpentlandii* Dunal	190,050	228	LC
*Solanumphysalidicalyx* Bitter	605,225	312	LC
*Solanumphysalifolium* Rusby	757,522	172	LC
*Solanumpilcomayense* Morong	15,437,317	768	LC
*Solanumpolytrichostylum* Bitter	432,164	244	LC
*Solanumprofusum* C.V.Morton	4,852	28	EN
*Solanumpseudoamericanum* Särkinen, P.Gonzáles & S.Knapp	668,293	180	LC
*Solanumpygmaeum* Cav.	18,428,537	596	LC
*Solanumradicans* L.f.	2,210,753	484	LC
*Solanumrhizomatum* Särkinen & M.Nee	71,565	80	LC
*Solanumriojense* Bitter	127,545	84	LC
*Solanumsalamancae* Hunz. & Barboza	79,244	84	LC
*Solanumsalicifolium* Phil	1,063,580	896	LC
*Solanumsarrachoides* Sendtn.	127,308,309	372	LC
*Solanumscabrum* Mill.	Not assessed, native to Africa (see Särkinen et al. 2018)		
*Solanumsinuatiexcisum* Bitter	625,487	148	LC
*Solanumsinuatirecurvum* Bitter	231,683	344	LC
*Solanumsubtusviolaceum* Bitter	163,921	100	LC
*Solanumtiinae* Barboza & S.Knapp	40,977	84	LC
*Solanumtriflorum* Nutt.	92,225,775	3,708	LC
*Solanumtripartitum* Dunal	410,690	564	LC
*Solanumtweedieanum* Hook.	2,010,678	1,420	LC
*Solanumweddellii* Phil.	603,950	220	VU
*Solanumwoodii* Särkinen & S.Knapp	122,138	64	LC
*Solanumzuloagae* Cabrera	144,608	108	LC

Most morelloid species are weedy and widely distributed; outside of the Americas, many species are also cultivated (e.g., *S.scabrum*, *S.tarderemotum* Bitter, *S.villosum*) and are distributed widely via human migration. Many introductions of species from Europe, particularly to North America, may have resulted from transport of soil or seed with introduced crops, but even casual visitors to far-flung places have been implicated in the introduction of alien species (Chown et al. 2012). Several of the South American species that are adventive in other parts of the world were transported via wool, particularly from Argentina (e.g., *S.chenopodioides*). The genetic structure of populations of extremely widespread species such as *S.americanum* will need to be investigated to determine if structure exists in the distribution that can be related to natural or human-mediated causes. Most of the South American species of morelloids, however, are confined to the continent; some are widespread (e.g., *S.cochabambense*, *S.nigrescens*) while others have quite narrow distributions (e.g., *S.annuum*, *S.caatingae*). It is possible that many of the species known from few collections are under-collected due to the common perception that all these species are the same, common, weedy and thus not worth collecting.

## ﻿Uses

Black nightshades are used as potherbs (often referred to on English language labels as “spinach”) worldwide, especially in Africa (Chweya and Eyzaguirre 1999). In the Americas, these plants are used in similar ways, especially among communities of African origin, but also more widely (Knapp et al. 2019). It is not clear whether the use of leaves of morelloid solanums was brought to the Americas by enslaved peoples from Africa; it is more likely their use as potherbs developed in parallel on both continents.

The use of these species as potherbs is much less prevalent in South America than in Central and North America (Knapp et al. 2019), although for Brazil Ferreira Kinupp and Lorenzi (2021) report use of leaves of *S.americanum* as a braised vegetable and fruits used in soups and jams. African diaspora descended from enslaved people in Brazil sporadically cultivated *S.scabrum* in coastal southeastern Brazil, but there are few recent collections, so its use may have disappeared, although other African solanaceaeous crops remain important elements of Brazilian cuisine (e.g., *S.aethiopicum* L., “Gilo” of the Leptostemonum clade).

Verification of uses of individual species is complicated by the lack of voucher specimens and the use of the name *S.nigrum* or its many complex synonyms in use in previous literature, where application in many cases is not clear (e.g., Hoehne 1939; González Torres 2009). Pérez-Arbeláez (1978) mentions “Solanumamericanum-nigrum” (possibly referring to *S.americanum*, *S.interandinum*, *S.macrotonum* and/or *S.nigrescens*) as a useful plant called hierbamora in Colombia, and describes the fruits, perhaps suggesting they are consumed. In Paraguay, González Torres (2009) describes “Solanum pterocaulum, y var. E. DC.” (possibly referring to *S.americanum*, *S.paucidens* and/or *S.pilcomayense*) with the common name of aguara kyja asu (Guaraní) as being toxic, but counterintuitively he also says that the fruits are perfectly safe to eat, and have medicinal use in treatment of spasmodic urine retention. For Brazil, Lorenzi and Abreu Matos (2021) cite a wide variety of common names and uses for *S.americanum* (possibly also referring to *S.paucidens*), including use as an analgesic, sedative, narcotic, expectorant, anti-aphrodisiac, diuretic, emollient, vermifuge and for joint pain, and medicinal use in treatment of psoriasis, eczema, ulcers and relief from itching.

In Araucano communities of Argentina, fruits of *S.chenopodioides* are eaten by children (Martínez Crovetto 1968) and in the Chaco area they are used as a pigment (Martínez Crovetto 1965). Several species are used medicinally in both Argentina and Paraguay (e.g., *S.americanum*, Keller 2007; Ibarrola and Degen 2011; Kujawska and Hilgert 2014; *S.nitidibaccatum*, Molares and Ladio 2012; *S.palitans*, Ceballos and Perea 2014; *S.pilcomayense*, Martínez Crovetto 1981; *S.tripartitum*, Hurrel 1991; Lupo and Echenique 1997). In Andean Peru several species (e.g., *S.cochabambense*, *S.corymbosum*, *S.pentlandii*) are used medicinally for a wide variety of uses, from purgatives to colic to stimulating hair growth (Franquemont et al. 1990; Roersch 1994). See individual species descriptions for details where uses are unambiguously assignable.

## ﻿Species concepts

Our goal for the treatment of the Morelloid clade has been to provide circumscriptions for the members of this morphologically variable group of species, while clearly highlighting areas, taxa and populations where further in-depth research would be useful. Our decisions to recognise species has relied on having clear morphological discontinuities to define easily distinguishable species. Delimitation of species follows what is known as the “morphological cluster” species concept (Mallet 1995; Knapp 2008a) where we recognise, “assemblages of individuals with morphological features in common and separate from other such assemblages by correlated morphological discontinuities in a number of features” (Davis and Heywood 1963). We have tried to emphasise similarities between populations instead of differences, which so often reflect incomplete collecting or local variation. Biological (Mayr 1982), phylogenetic (Cracraft 1989) and other finely defined species concepts (see Mallet 1995) are almost impossible to apply in practice and are therefore of little utility in a practical sense (see Knapp 2008a). It is important, however, to clearly state the criteria for the delimitation of species, rather than dogmatically follow particular ideological lines (see Luckow 1995; Davis 1997). In a few cases, molecular phylogenetic data have helped us to recognise a set of somewhat cryptic clusters as distinct species based on complex morphological variation (*S.arequipense* and *S.furcatum*; *S.nitidibaccatum*, *S.physalifolium*, and *S.sarrachoides*; *S.fragile* and *S.grandidentatum*). Even in these cases, we have not always had the opportunity to sample multiple individuals per species to confirm species monophyly but have used single accessions of each morphological cluster to show their distinct placement in the phylogeny away from their morphologically most closely related cluster (Särkinen et al. 2015b; Gagnon et al. 2022). Specific morphological characters used for recognition are detailed with each species description and in the key.

We have not recognised subspecies or varieties, but have rather described and documented variation where present, rather than formalised such variability with a name which then encumbers the literature. Although infraspecific taxa have been recognised by others within the morelloids, we do not recognise any here due to the complex morphological variation observed within each species, where the inspection of large number of specimens quickly reveals no apparent natural breaks in variation, but rather a mixing between highly morphologically variable populations of widespread species. Some potential reasons for variability and intergradation are recent divergence, hybridisation and environmental influence on morphology. We have been conservative in our approach, recognising as distinct entities those population systems (sets of specimens) that differ in several morphological characteristics. Many of the species in the group (and of morelloids in general) are extremely widespread and variable; variation exists in certain characters, but the pattern of variation is such that no reliable units can be consistently extracted, nor is geography a completely reliable predictor of character states. Here variability within and between populations seems more important than the variations of the extremes other taxonomists have recognised as distinct. We describe this variation realising that others may wish to interpret it differently. Widespread species often harbour cryptic diversity (Cavers et al. 2013), especially in groups such as the Morelloid clade, where differences between species are relatively small, so future research on the widespread variable entities recognised here will be needed (e.g., *S.americanum*, *S.cochabambense*, *S.interandinum*, *S.macrotonum*, *S.nigrescens*).

## ﻿Materials and methods

Our taxonomic treatment is based on results from recent molecular systematic studies considering the taxonomy of the section and the molecular phylogenetic study of the entire Morelloid clade by Särkinen et al. (2015b). Molecular data have been useful for recognition of some species that are very similar morphologically, but distinct phylogenetically (see Species concepts above). Molecular data also help delimit the Morelloid clade, confirming the inclusion of most suspected species of the group (65 of the total 79 currently known morelloid species sampled in the latest molecular phylogeny; Gagnon et al. 2022). Phylogenetic studies show some species previously considered members of the clade to have other affinities (e.g., *S.concarense* Hunz. now placed in Dulcamaroids, and *S.reductum* C.V.Morton now placed in the Geminata clade; Särkinen et al. 2015b; Gagnon et al. 2022) and in some cases include previously excluded taxa (e.g., *S.salicifolium*; Särkinen et al. 2015b).

Descriptions are based on field work and physical and virtual examination of 33,673 [of 24,619 collections] herbarium specimens (of which 16,352 specimens and 11,157 collections were from South America) from 317 herbaria (see Suppl. materials 2 and 3): A, AAU, AD, AK, ALCB, ANG, APSC, ARIZ, ASC, ASE, ASU, AZU, B, BA, BAA, BAB, BAF, BAH, BART, BBB, BBLM, BBLM-OWY, BCRU, BH, BHCB, BHSC, BISH, BLMVL, BM, BOIS, BOLV, BONN, BP, BR, BRI, BRU, BRY, BSD, BSHC, BSN, BUT, B-W, C, CAL, CANB, CANU, CAS, CCNL, CEN, CEPEC, CESJ, CGE, CHR, CIC, CICY, CLEMS, CM, CNS, CO, COI, COL, COLO, CONC, CONN, CORD, CPUN, CR, CRMO, CTES, CUZ, DBG, DD, DES, DNA, DR, DS, DSC, DSM, DUKE, DVPR, E, EA, EAC, ECON, EIU, EKY, EMC, ESA, EWU, F, FCQ, FHO, FI, FR, FSU, FT, FUEL, FURB, G, GA, GAS, GB, G-BOIS, G-DC, GH, GILAN, GMUF, GOET, GZU, H, HA, HAJB, HAMAB, HAO, HAS, HB, HBG, HCF, HF, HFSL, HO, HOH, HOXA, HPSU, HST, HSTM, HUCS, HUEFS, HUEM, HUFSJ, HUSA, HUT, IAC, ICN, ID, IDS, IFP, ILLS, INB, IND, INPA, IPA, JE, JEPS, JOI, JPB, K, KESC, KFTA, KHD, KIRI, KUFS, L, LAE, LAGU, LD, LE, LEA, LG, LIL, LINN, LL, LOJA, LP, LPB, LSU, LU, M, MA, MAC, MAINE, MARY, MASS, MBM, MBML, MCNS, MEL, MEN, MERL, MEXU, MHES, MHU, MICH, MIN, MISS, MISSA, MO, MOL, MONT, MONTU, MOR, MPU, MPUC, MSC, MT, MU, MY, N/A, NCBS, NCU, NDG, NE, NEB, NEBC, NHA, NHT, NIJ, NS, NSW, NY, OBI, OS, OSC, OTA, OUPR, OXF, P, PACA, PAL, PBL, PERTH, PEUFR, PH, P-LA, PNNL, POM, PSM, Q, QAP, QCA, QCNE, QPLS, QRS, R, RAB, RB, RENO, RIOC, RM, RSA, S, SASK, SBBG, SCA, SD, SF, SGO, SI, SING, SJRP, SMDB, SOC, SP, SPF, SPSF, SPWH, SRFA, SRP, SRSC, STU, TAN, TCD, TEX, TO, TTRS, TUB, U, UB, UBC, UC, UCR, UCS, UDBC, UEC, UFP, UFRN, UMO, UNA, UNCC, UNOP, UNSL, UOS, UPCB, UPS, URV, US, USF, USFS, USM, USMS, USZ, UT, UTC, V, VEN, VIES, VMSL, VPI, VSC, VT, W, WAG, WCSU, WCW, WELT, WGCH, WIS, WOLL, WRSL, WS, WSTC, WTU, WU, WWB, YA, YU, Z. Some of these specimens were examined digitally through individual herbarium portals; we include only those specimens we have been able to unequivocally identify from these images or that are duplicates of collections we have personally examined. We have compared introduced and adventive species across their entire ranges, not only collections from South America.

Measurements were made from dried herbarium material supplemented by measurements and observations from living material. Colours (e.g., corollas, fruits etc.) are described from living material or from herbarium label data. Specimens with latitude and longitude data on the labels were mapped directly. Some species had few or no georeferenced collections; in these cases we retrospectively georeferenced the collections using available locality data. Species distribution maps were constructed with the points in the centres of degree squares in a 1° square grid. Conservation threat status was assessed following the IUCN Red List Categories and Criteria (IUCN 2022) using the GIS-based method of Moat (2007) as implemented in the online assessment tools in GeoCat (http://geocat.kew.org). The Extent of Occurrence (EOO) measures the range of the species, and the Area of Occupancy (AOO) represents the number of occupied points within that range based on the default grid size of 2 km^2^. Preliminary conservation assessments largely used EOO since AOO is prone to collection and georeferencing bias (see section on Conservation status).

Type specimens for many morelloids have proved difficult to trace; most of the names for the introduced European species (e.g., *S.nigrum*, *S.villosum*) and for North and Central American species introduced elsewhere (e.g., *S.americanum*, *S.triflorum*) have been treated in Särkinen et al. (2018). Decisions on choices of lectotypes and synonymy can be found there.

Where specific herbaria have not been cited in protologues we have followed McNeill (2014) and designated lectotypes rather than assuming holotypes exist. We cite page numbers for all previous lectotypifications. In general, we have lectotypified names with the best preserved, or in some cases with the only, herbarium sheet we have seen; in these cases, we have not outlined our reasoning in detail. Where there has been difficulty or where the choice may not be obvious, we detail our reasoning at the end of the species discussions. Wherever possible, we have designated specimens in the country where the types were collected as the lectotypes (e.g., Smith and Figueiredo 2011). When lectotypes have been designated inadvertently (Prado et al. 2015), we indicate how the type was cited in the lectotypifying work (e.g., [as holotype] or [as type]).

Georg Bitter described many taxa of *Solanum* in the course of his monumental work on the genus *Solanum* and worked widely in Germany in the period between the two World Wars (Weber 1928), including, but not exclusively at Berlin (Vorontsova and Knapp 2010). His protologues sometimes include specific herbarium citations, but often do not. We have cited specimens as holotypes only when a single specimen with a single herbarium citation is indicated in the protologue; we have not assumed his types were/are all in B. For Bolivian species based on the collections of Otto Buchtien he often cited “herb. Buchtien” or “herb. Boliv. Buchtien”. This collection came to the Smithsonian Institution during the period of P.C. Standley’s curatorship (1909–1928) of the US National Herbarium (US) and many of the specimens are annotated by Buchtien with Bitter’s new names; we have selected these US duplicates as lectotypes in these cases (see Morton and Stern 1966). Buchtien often used the same number series for different collecting voyages, so care must be taken with assigning “duplicates” as isotypes (see for example the various synonyms of *S.gonocladum* and *S.pallidum*).

Type specimens with sheet numbers are cited with the herbarium acronym followed by the sheet number (e.g., S [acc. # 04-2998]); barcodes are written as a continuous string in the way they are read by barcode readers (e.g., G00104280, MO-1781232); in citations the barcodes are cited first, followed by accession numbers [e.g., US (00027289, acc. # 1416199)]. For widely distributed and adventive species we have cited only types based on material from the Americas; the synonymy for *S.americanum* in particular is extensive and includes many names based on collections from outside of the Americas. Details of names based on types from Africa, Asia, Australia, Europe and Oceania can be found in Särkinen et al. (2018) or on Solanaceae Source (www.solanaceasoursce.org).

All collections seen for this study are presented in Supplementary material. An index to numbered collections from South America is presented in Suppl. material 1; a searchable csv file for all South American specimens is presented in Suppl. material 2 and a searchable csv file with all specimens seen (including all those from outside South America) is in Suppl. material 3. These Supplementary materials files and full specimen details are also available on the Solanaceae Source website (www.solanaceaesource.org) and in the dataset for this study deposited in the Natural History Museum Data Portal (https://doi.org/10.5519/3fh6f88q).

Citation of literature follows BPH-2 (Bridson 2004) with alterations implemented in IPNI (International Plant Names Index, http://www.ipni.org) and Harvard University Index of Botanical Publications (http://kiki.huh.harvard.edu/databases/publication_index.html). Following Knapp (2013) we have used the square bracket convention for publications in which a species is described by one author in a publication edited or compiled by another (e.g., the traditional “in” attributions such as Dunal in DC. for those taxa described by Dunal in Candolle’s *Prodromus Systematis Naturalis Regni Vegetabilis*). This work is cited here as Prodr. [A.P. de Candolle] and the names are thus attributed only to Dunal. Standard forms of author names are according to IPNI (International Plant Names Index, http://www.ipni.org).

## ﻿Taxonomic treatment

### ﻿The Morelloid clade sensu Bohs (2005) and Särkinen et al. (2015b)

**The Morelloid clade**

**The Morelloid clade, sensu Bohs (2005) and Särkinen et al. (2013, 2015b)**

*Solanum* grad. ambig. *Maurella* Dunal, Hist. Solanum 119, 151. 1813. Lectotype species. *S.nigrum* L. (designated by D’Arcy 1972).

SolanumsectionMorella Dumort., Fl. Belg. 39. 1827. Lectotype species. *S.nigrum* L. (designated by D’Arcy 1972).

SolanumsectionInermis G.Don, Gen. Syst. 4: 400. 1838. Lectotype species. *S.nigrum* L. (designated by D’Arcy 1972).

*Solanum* grad ambig. *Morella* G.Don, Gen. Syst. 4: 411. 1838. Lectotype. *S.nigrum* L. (designated by D’Arcy 1972).

SolanumsectionPachystemonum Dunal, Prodr. [A. P. de Candolle] 13(1): 28, 31. 1852. Lectotype species. *S.nigrum* L. (designated by D’Arcy 1972).

SolanumsubsectionMorella Dunal, Prodr. [A. P. de Candolle] 13(1): 28, 44. 1852. Lectotype species. *S.nigrum* L. (designated by D’Arcy 1972).

SolanumsectionCampanulisolanum Bitter, Repert. Spec. Nov. Regni Veg. 11: 234. 1912. Lectotype species. *S.fiebrigii* Bitter (designated by Seithe 1962).

SolanumsectionEpisarcophyllum Bitter, Repert. Spec. Nov. Regni Veg. 11: 241. 1912. Lectotype species. *S.sinuatirecurvum* Bitter (designated by Seithe 1962).

*Solanocharis* Bitter, Repert. Spec. Nov. Regni Veg. 15: 153. 1918. Type species. *Solanocharisalbescens* (Britton) Bitter (= *Solanumalbescens* (Britton) Hunz.)

SolanumsectionMorella (Dunal) Bitter, Bot. Jahrb. 54: 416, 493. 1917. Lectotype species. *S.nigrum* L. (designated by D’Arcy 1972).

SolanumsectionChamaesarachidium Bitter, Repert. Spec. Nov. Regni Veg. 15: 93. 1919. Type species. *S.chamaesarachidium* Bitter (= *S.weddellii* Phil.).

*Solanum* series *Transcaucasica* Pojark., Bot. Mater.Gerb.Inst. Komorova Akad. Nauk S.S.S.R. 17: 332. 1955. Lectotype species. *S.transcauscasica* Pojark. (= *S.villosum* Mill.) (designated by D’Arcy 1972 [as type]).

*Solanum* series *Alata* Pojark., Bot. Mater.Gerb.Inst. Komorova Akad. Nauk S.S.S.R. 17: 336.1955. Type species. *S.alatum* Moench [nom. et typ. cons.] (= *S.villosum* Mill.) (designated by D’Arcy 1972 [as type]).

*Solanum* series *Pseudoflava* Pojark., Bot. Mater.Gerb.Inst. Komorova Akad. Nauk S.S.S.R. 17: 338. 1955. Type species. *S.pseudoflavum* Pojark. (= *S.villosum* Mill.) (designated by D’Arcy 1972 [as type]).

SolanumsectionParasolanum Child, Feddes Repert. 95: 142. 1984. Type species. *S.triflorum* Nutt.

SolanumsectionSolanocharis (Bitter) Child, Feddes Repert. 95: 147. 1984, as ‘*Solancharis*’ Type species. *Solanumalbescens* (Britton) Hunz.

SolanumsectionDulcamara (Moench) Dumort. subsect. 2 “*herbaceous plants confined to the central Andes*” of Nee (1999: 295) [includes the species of Child’s section Parasolanum excluding the type]

SolanumsectionSolanum subsects. 1 “*Solanum*”, 2 “*Glandularpubescent group*”, 3 “*Campanulisolanum*”, 4 “*Chamaesarachidium*” and 6 “*Episarcophyllum*” of Nee (1999: 306–308), excluding his subsect. 5 “*Gonatotrichum*” [now recognised as being part of the Brevantherum clade, see Stern et al. 2013].

*Solanum* series *Lutea* Pojark. ex Ivanina, Bot. Zhurn. (Moscow & Leningrad) 85(6): 144. 2000. Type species. *S.villosum* Mill.

**Description.** Annual to perennial herbs, subshrubs or shrubs, often woody at the base; unarmed. Stems terete or angled, sometimes hollow, lacking true prickles but sometimes with spinose processes along the angles, glabrous or pubescent with simple or branched (forked and dendritic) uniseriate trichomes, these eglandular or glandular. Sympodial units difoliate, trifoliate or plurifoliate, the leaves usually not geminate. Leaves simple with entire or variously dentate or lobed margins or occasionally deeply pinnatifid, concolorous or less commonly discolorous, glabrous to densely pubescent with eglandular and/or glandular simple or branched (only in South America) uniseriate trichomes; petioles generally well developed, the leaves sessile in some species. Inflorescences opposite the leaves or internodal, unbranched, forked or many times branched, not bracteate (except in *S.triflorum* where a single bracteole sometimes present), with few to many (up to 100) flowers, these clustered at the tip (umbelliform or sub-umbelliform) or spaced along the axis; peduncle various, usually not longer than the inflorescence branches; pedicels articulated at the base (in *S.interius* Rydb. of North America the basal flower with the articulation slightly above the base), either flush with the axis or leaving a small stump, occasionally with a cup-shaped base in fruit (*S.caesium*). Flowers 5-merous (occasionally 4-merous or fasciate and 6–7-merous in *S.scabrum*), actinomorphic to very slightly zygomorphic in filament length or calyx lobe length, cosexual (hermaphroditic). Calyx with the lobes deltate to spathulate to long-triangular. Corolla deeply to broadly stellate or pentagonal and rotate-stellate, rarely campanulate, white or purplish-tinged to lavender or purple, rarely pale yellow (*S.huayavillense*), usually with an “eye” at the base of the lobes of a contrasting colour (yellow, green or dark purplish black), the lobes spreading or reflexed at anthesis. Stamens equal or very slightly unequal, the filaments equal to very slightly unequal, glabrous or more usually densely pubescent with tangled uniseriate weak-walled simple uniseriate trichomes, the anthers ellipsoid (slightly tapering in *S.scabrum*; somewhat beaked in *S.woodii*) and connivent, with distal pores that elongate to slits with drying and/or age. Ovary conical, glabrous or occasionally very minutely puberulent; style straight or curved and bent, usually pubescent with simple uniseriate trichomes in the lower half, exserted from the anther cone, sometimes only very slightly so; stigma minutely capitate to capitate or clavate. Fruit a globose, flattened (depressed) or ellipsoid juicy berry with thin pericarp, green, blackish purple, yellow or reddish orange at maturity, occasionally marbled with white (e.g., *S.physalifolium*), opaque or translucent, glabrous; fruiting pedicels spreading or deflexed, occasionally secund, either remaining on the plant after fruit drop (persistent) or not; fruiting calyx lobes spreading, reflexed, appressed or accrescent at fruit maturity; accrescent lobes appressed or inflated, the base sometimes invaginate. Seeds mostly flattened and teardrop shaped, occasionally reniform or rounded, yellow or tan to dark brown, the surfaces minutely pitted or in a few species tuberculate (e.g., *S.annuum*, *S.gilioides*, *S.weddellii*). Stone cells absent or present, if present few to numerous. Chromosome number: n = 12, 24, 36 (see Särkinen et al. 2018, and individual species treatments).

**Distribution.** A worldwide species group occurring in on all continents except Antarctica, but with highest species diversity in the central and southern Andes and Africa.

**Discussion.** In the synonymy of the group presented here we have included all groups that are members of the clade as we define it; for more detailed discussion of morphology and group definition see Särkinen et al. (2015b). *Solanumnigrum* is the lectotype species of *Solanum* (Hitchcock and Green 1929), and thus if the Morelloid clade were to be formally recognised at the infrageneric level it would necessarily be called *Solanum* independent of rank (as recognised by Seithe 1962).

Members of the Morelloid clade are among the most widely collected of solanums, in part because are they are herbaceous, widespread and often weedy. They are also among the most difficult to identify, due to their extreme vegetative plasticity (see Morphology above) and their lack of striking distinguishing characters. Combinations of characters are most useful for identification, and we have included these in the species treatments as well as in the keys. Geography is very helpful in assisting with species identification in this group, but the large number of potentially invasive and introduced species means one must exercise caution if a species is not readily identifiable and consider species not currently known from the area (taking into account variation of course).

The Morelloid clade suffers from two extreme sorts of taxonomic recognition issues. Firstly, in many parts of the world all taxa have been treated as a single highly variable species (usually *S.nigrum*, see for example Sendtner 1846) and local endemic taxa are overlooked. Secondly, and especially in Europe in the late 19^th^ and early 20^th^ century, many minor variants were described and were then transferred and recombined at different taxonomic levels, creating a confusing morass of names, many of which lack types. The latter is unfortunate because of the nomenclatural work entailed in sorting out the identities and types for these names is time-consuming and often quite difficult (see Särkinen et al. 2018), but the former is more serious, because endemic taxa have been overlooked (e.g., *S.caatingae*, *S.marmoratum*) and thus have possibly been placed at risk due to their being equated with widespread invasive weeds.

These plants are all remarkably superficially similar and distinguishing features often involve minute differences in anther length; geography is often a good indicator of what species one has, but not always. Combinations of characters are useful in identifying these species and to this end we provide a synoptic character list after the main dichotomous key.

### ﻿Artificial key to the Morelloid species of South America

A global multi-access key that includes all of the taxa in this monograph can be found at http://xper3.fr/xper3GeneratedFiles/publish/identification/-3915026624309343770/mkey.html and under the identification tab on Solanaceae Source. Country-level keys have been published for Argentina (Knapp et al. 2020) and Brazil (Knapp and Särkinen 2018). Geography is only indicated in couplets that terminate in a species name.

**Table:** 

1a	Plants viscid-pubescent with multicellular, uniseriate glandular trichomes on stems and leaves (these sometimes confined to the new growth); sticky to the touch	**2a**
1b	Plants variously pubescent or glabrous, not viscid-pubescent with multicellular glandular trichomes; not sticky to the touch	**22**
2a	Inflorescence forked or with multiple branches	**3a**
2b	Inflorescence unbranched	**10**
3a	Corolla campanulate; anther connectives somewhat enlarged. Argentina, Bolivia, Peru	***Solanumfiebrigii***
3b	Corolla pentagonal to stellate; anther connectives not enlarged	**4a**
4a	Fruiting calyx accrescent, markedly enlarging in fruit, mostly enclosing the mature berry; mature berry cream-coloured, tightly enclosed in the calyx. Argentina, Bolivia	***Solanumtweedieanum***
4b	Fruiting calyx spreading or appressed to the berry, not markedly enlarging and enclosing the berry; mature berry variously coloured, not cream-coloured	**5a**
5a	Calyx lobes in flower long-triangular, longer than the tube to twice the length of the tube	**6a**
5b	Calyx lobes in flower deltate to triangular, equal to the length of the tube	**7a**
6a	Leaves somewhat rubbery or fleshy, strongly decurrent onto the winged stems; corolla rotate to pentagonal; flowers spaced along the inflorescence axis; pedicels in fruit strongly deflexed; mature berry greenish orange or yellow. Argentina, Bolivia	***Solanumcaesium***
6b	Leaves membranous, not decurrent onto winged stems; corolla stellate; flowers clustered at inflorescence tips; pedicels in fruit spreading; mature berry green. Bolivia, Peru	***Solanumsubtusviolaceum***
7a	Anthers 4–4.5 mm long; corolla deeply stellate, divided nearly to the base; buds elongate-ellipsoid; leaves usually entire (occasionally toothed at the very base). Argentina, Bolivia	***Solanumglandulosipilosum***
7b	Anthers less than 4 mm long; corolla broadly stellate, divided to halfway to the base; buds ellipsoid or globose; leaves usually toothed along the whole margin	**8a**
8a	Sympodial units plurifoliate, the leaves not geminate; buds ellipsoid; stone cells present in berries. Bolivia, Peru	***Solanumjuninense***
8b	Sympodial units difoliate, the leaves geminate or not; buds globose; stone cells absent in berries	**9a**
9a	Herbs or small shrubs of disturbed areas (e.g., landslides, cultivations, houses), generally lacking a woody rootstock; foliage rank-smelling; calyx lobes 1–1.5 mm long, acute-tipped. Bolivia, Chile, Ecuador, Peru	***Solanumgrandidentatum***
9b	Herbs of high elevation dry puna vegetation generally associated with rocks and not with disturbed areas, with woody rootstocks (brittle at the base of green stems); foliage without odour; calyx lobes 2–3 mm long, blunt-tipped. Bolivia, Chile, Peru	***Solanumfragile***
10a	Fruiting calyx not markedly enlarging and accrescent to enclose the berry, the lobes spreading or appressed to the base of the fruit	**11a**
10b	Fruiting calyx markedly enlarging and accrescent, the lobes enclosing or more than half enclosing the mature berry	**16а**
11a	Corolla campanulate to rotate; glandular trichomes often confined to new growth and absent from older stems. Argentina, Bolivia, Peru	***Solanumsinuatiexcisum***
11b	Corolla variously stellate; glandular trichomes on entire plant	**12a**
12a	Calyx lobes 0.2–1.5 mm long, appressed to the base of the berry	**13a**
12b	Calyx lobes greater than 1 mm long, spreading or appressed in fruit	**14a**
13a	Calyx lobes 0.2–0.5 mm long, acute; anthers 3–4 mm long; stone cells 4 per berry. Amazonian Region	***Solanumarenicola***
13b	Calyx lobes 1–1.5 mm long, spathulate; anthers 1.8–2.2 mm long; stone cells absent. Brazilian caatinga	***Solanumcaatingae***
14a	Calyx lobes long-triangular, different in texture to the calyx tube; stone cells present in berries. Bolivia, Peru	***Solanumsubtusviolaceum***
14b	Calyx lobes ovate to triangular, similar in texture from the calyx tube; stone cells absent	**15a**
15a	Anthers 3–3.8 mm long, wider at the base; corolla strongly exserted from the bud before anthesis, exceeding the tips of the lobes. Argentina, Bolivia	***Solanumwoodii***
15b	Anthers 2.5–3.2 mm long, ellipsoid, of equal width along entire length; corolla barely exceeding the calyx lobe tips before anthesis. Argentina, Bolivia	***Solanummichaelis***
16a	Fruiting calyx inflated and completely enclosing the berry, the tube longer than the lobes. Argentina, Bolivia	***Solanumphysalidicalyx***
16b	Fruiting calyx not inflated, tightly enclosing the berry or somewhat spreading (half or more than half enclosing the berry)	**17a**
17a	Calyx completely enclosing the bud; fruiting calyx covering more than half the berry; mature berry green; inflorescence opposite the leaves; plants delicate annuals. Argentina, Brazil, Paraguay, Uruguay	***Solanumsarrachoides***
17b	Calyx not completely enclosing the bud; fruiting calyx covering half the berry; mature berry green with white marbling or cream-coloured; inflorescence usually internodal, occasionally some inflorescences on a plant almost opposite the leaves; plants woody at the base, or more robust annual weeds	**18a**
18a	Anthers less than 1 mm long; corolla usually with a purple or blackish purple central star; plants herbaceous annual weeds. Argentina, Chile (introduced elsewhere)	***Solanumnitidibaccatum***
18b	Anthers greater than 1 mm long; corolla with a green central star; plants woody at the base (often rhizomatous)	**19a**
19a	Leaves narrowly ellipsoid to lanceolate; stone cells absent in berries. Argentina	***Solanumprofusum***
19b	Leaves variously ovate to ellipsoid; stone cells present in berries	**20a**
20a	Anthers ca. 2 mm long; calyx lobes spreading in fruit, not tightly appressed to the berry; leaves ovate to elliptic-ovate. Argentina, Bolivia, southern Peru	***Solanumphysalifolium***
20b	Anthers usually longer than 3.5 mm long (occasionally as short as 2.6 mm long in poorly developed flowers), usually 4–5 mm long; calyx lobes narrowly triangular, tightly appressed to the berry; leaves rhombic to elliptic	**21a**
21a	Leaf bases truncate, distinctly narrowing to a petiole; anthers ca. 1 mm wide; stone cells 6–8 per berry. Argentina, Bolivia, Paraguay	***Solanumtweedieanum***
21b	Leaf bases attenuate onto the petiole and stem, the petiole winged; anthers 1.2–1.5 mm wide; stone cells 10–11 per berry. Northern Argentina, Bolivia	***Solanumhunzikeri***
22a	Leaves pinnatisect, divided halfway or more to the midrib (occasionally with some simple leaves, but the majority pinnatisect)	**23a**
22b	Leaves simple, the margins toothed or not, not divided into leaflets	**31a**
23a	Mature berries red, orange, yellow or greenish orange, translucent and usually somewhat depressed or flattened; two large apical stone cells present	**24a**
23b	Mature berries green, purple or yellow (if yellow not translucent), globose to ellipsoid; stone cells present or absent	**26a**
24a	Mature berries translucent yellow; corolla less than 1 cm in diameter; plants usually prostrate and creeping, rooting at the nodes. Argentina, Bolivia	***Solanumpalitans***
24b	Mature berries orange or red; corolla ca. 1 cm in diameter; plants herbs or subshrubs, not markedly prostrate	**25a**
25a	Leaves three-parted; stem terete or only slightly angled; inflorescence several times branched; mature berries red, markedly bilobed when immature. Argentina, Bolivia	***Solanumtripartitum***
25b	Leaves 5-parted; stem strongly angled to winged; inflorescence unbranched (very rarely forked); mature berries orange or orange-yellow, somewhat flattened but not strongly bilobed. Bolivia, Chile, Ecuador, Peru	***Solanumradicans***
26a	Tiny annual herbs 5–30 cm tall; corolla pentagonal to rotate; fruiting calyx variously accrescent; seeds tuberculate	**27a**
26b	Annual or perennial herbs or subshrubs (5)20–150 cm tall; corolla shallowly to deeply stellate; fruiting calyx not markedly accrescent; seeds minutely pitted, not tuberculate	**29a**
27a	Fruiting calyx not enclosing the berry, a spreading plate-like structure; inflorescence with 8–12 flowers; berry with only 2 seeds. Argentina	***Solanumannuum***
27b	Fruiting calyx partly to completely enclosing the berry; inflorescence with 2–5 (6) flowers; berry with more than 2 seeds (to 20)	**28a**
28a	Calyx lobes broadly elliptic to ovate, rounded at the tips, only partially enclosing the berry at maturity; anthers ca. 1 mm long; style only just exceeding the anther cone; plants of loose, sandy soils. Argentina, Bolivia, Chile, Peru	***Solanumweddellii***
28b	Calyx lobes long-triangular, pointed at the tips, inflated and completely enclosing the berry at maturity; anthers usually more than 1 mm long; style clearly exserted from the anther cone; plants of rocky. Argentina, Bolivia	***Solanumgilioides***
29а	Buds narrowly ellipsoid; anthers less than 4 mm long, narrowly ellipsoid and very narrow relative to length; inflorescence with “bracteoles” amongst the pedicels; berry green. Argentina, Bolivia (introduced elsewhere)	***Solanumtriflorum***
29b	Buds ellipsoid; anthers more than 4 mm long, ellipsoid; inflorescence without “bracteoles”; berry purple or yellow	**30a**
30a	Leaves membranous, extremely variable in shape even on single plants; corolla less than 2 cm in diameter, deeply stellate, the lobes reflexed at anthesis; berry purple or purplish red, soft in texture, with ca. 10 stone cells per berry. Argentina, Paraguay	***Solanumsalicifolium***
30b	Leaves thick and coriaceous, somewhat fleshy; corolla more than 2 cm in diameter, shallowly stellate, the lobes spreading; berry yellow, leathery in texture, stone cells absent. Argentina, Bolivia, Chile	***Solanumsinuatirecurvum***
31a	Leaves coriaceous or fleshy, the margins often strongly revolute	**32a**
31b	Leaves membranous, the margins not strongly revolute	**36**
32a	Buds narrowly ellipsoid; anthers less than 1 mm wide; pubescence of stiff antrorse trichomes; annual herbs. Argentina, Bolivia (introduced globally)	***Solanumtriflorum***
32b	Buds ellipsoid to broadly ellipsoid; anthers 1 mm wide or wider; pubescence of unicellular papillae or tangled soft white trichomes, not stiff and antrorse; perennials from a woody base (resprouting from the rhizome every season) or fleshy herbs	**33a**
33a	Fleshy herbs; stems decumbent or somewhat erect; flowers widely spaced on the inflorescence axis; corolla uniformly white; mature berries translucent yellow or pale orange. Argentina, Bolivia	***Solanumcaesium***
33b	Perennials from a woody base (resprouting from the rhizome every season); flowers clustered; corolla white or purple with a central star; mature berries yellow, green or purple, not translucent	**34a**
34a	Stems glabrous or with an even covering of minute papillate unicellular trichomes; inflorescence with more than 4 flowers; corolla white or pale violet. Argentina, Chile	***Solanumechegarayi***
34b	Stems with pubescence of tangled white multicellular trichomes; inflorescences with fewer than 4 flowers; corolla lilac, deep purple or lilac-striped	**35a**
35a	Flowering pedicels 1–2 cm long; calyx lobes acute at the tips; corolla 1–1.2 cm in diameter, deep purple; anthers 4–5.5 mm long; fruiting pedicels 1.5–2 cm long; berry 1–1.5 cm in diameter, bright yellow at maturity. Argentina, Bolivia, Chile	***Solanumsinuatirecurvum***
35b	Flowering pedicels 0.8–1.1 cm long; calyx lobes rounded at the tips; corolla 1.8–2 cm in diameter, pale lilac or white and lilac; anthers 3.5–4.5 mm long; fruiting pedicels 1.3–1.5 cm long; berry to 1.1 cm in diameter, green or purple. Argentina	***Solanumriojense***
36a	Stems and leaves with multicellular dendritic (branched) trichomes. Bolivia, Peru	***Solanumpallidum***
36b	Stems and leaves glabrous or with multicellular simple (unbranched) trichomes	**37а**
37а	Stem angled with prominent spinulose processes; sympodial units difoliate, the leaves usually geminate; fruiting calyx accrescent and inflated, completely enclosing the berry. Argentina	***Solanumsalamancae***
37b	Stem terete or angled, without prominent and persistent spinulose processes; sympodial units difoliate or plurifoliate, the leaves geminate or not geminate; fruiting calyx not accrescent and not completely enclosing the berry	**38a**
38a	Mature berries red; inflorescences many times branched	**39a**
38b	Mature berries green, yellow or purple; inflorescences branched or unbranched	**40а**
39a	Leaves elliptic, the base attenuate; filaments glabrous; berries 0.6–0.7 cm in diameter, somewhat bilobed; fruiting pedicels 0.6–0.7 cm long; local population in Salta, Argentina (see species description, most populations with pinnatifid leaves)	***Solanumtripartitum***
39b	Leaves ovate-lanceolate, the base narrowly attenuate; filaments pubescent adaxially; berries 0.4–0.6 cm in diameter, globose; fruiting pedicels 0.2–0.3 cm long. Peru (introduced to Mexico)	***Solanumcorymbosum***
40a	Anthers less than or equal to 3 mm long	**41a**
40b	Anthers more than 3 mm long	**60a**
41a	Stems strongly winged; berry green marbled with white. Argentina	***Solanummarmoratum***
41b	Stems terete or only angled, not strongly winged; berry purple, green or blackish purple, not marbled	**42a**
42a	Inflorescences several times branched or forked	**43a**
42b	Inflorescences unbranched (only occasionally forked)	**52a**
43a	Corolla pale yellow or cream coloured; calyx tube slightly urceolate. Argentina, Bolivia	***Solanumhuayavillense***
43b	Corolla white or various shades of purple or lilac; calyx tube cup-shaped, not at all urceolate	**44a**
44a	Flower buds globose; styles long-exserted at anthesis (often protruding from the bud)	**45a**
44b	Flower buds ellipsoid or obellipsoid (if globose then most inflorescences unbranched and berries not shiny); styles not markedly long-exserted at anthesis	**47a**
45a	Inflorescences many times branched; anthers 2–2.5 mm long; leaves strongly toothed; stone cells absent. Bolivia, Peru	***Solanumpentlandii***
45b	Inflorescences forked, only rarely more than once-branched (*S.arequipense*); anthers 2.3–3.6 mm long; leaves entire or toothed; stone cells present	**46a**
46a	Stone cells more than 6 per berry; inflorescence branches only moderately divergent; calyx lobes deltate. Argentina, Chile	***Solanumfurcatum***
46b	Stone cells absent or only 2 per berry; inflorescence branches strongly divergent; calyx lobes elongate-deltate. Peru	***Solanumarequipense***
47a	Mature berry shiny, usually 1–2 cm in diameter; anthers ochre-yellow, slightly tapering; seeds greater than 2 mm long; cultivated plants (from Africa)	***Solanumscabrum***
47b	Mature berries shiny or matte, less than 2 cm in diameter; anthers bright yellow, ellipsoid; seeds less than 2 mm long; wild plants	**48a**
48a	Fruiting pedicels strongly secund; flowers evenly spaced along the inflorescence axis. Argentina, Brazil, Paraguay	***Solanumpaucidens***
48b	Fruiting pedicels not markedly secund; flowers clustered at the tips of inflorescence branches	**49a**
49a	Anthers 2.5 mm long or less. Bolivia, Ecuador, Peru	***Solanumpseudoamericanum***
49b	Anthers longer than 2.5 mm (if shorter then more than five stone cells per berry)	**50a**
50a	Woody shrubs; peduncles and old inflorescence axes remaining on plants; calyx lobes long triangular. Colombia, Bolivia, Ecuador, Peru	***Solanuminterandinum***
50b	Coarse herbs, often woody at the base; old inflorescences not remaining on plants; calyx lobes deltate	**51a**
51a	Berries globose; stone cells more than five per berry; anthers 2–3 mm long; fruiting pedicels 1–1.2 cm long, not markedly woody, not persistent after fruit maturity. Colombia, Ecuador, French Guiana, Guyana, Suriname, Venezuela	***Solanumnigrescens***
51b	Berries ellipsoid; stone cells two per berry or absent; anthers 2.8–3.4 mm long; fruiting pedicels 1.1–2.2 cm long, markedly woody and persisting after fruit maturity. Bolivia, Ecuador, Peru	***Solanumantisuyo***
52a	Prostrate woody shrubs; corollas campanulate	**53a**
52b	Erect or scandent shrubs or herbs; corollas variously stellate	**54a**
53a	Leaves coriaceous, glabrous except for a few trichomes along the veins; trichomes not stiff and antrorse; flowers 1.5–2 cm long. Bolivia	***Solanumalbescens***
53b	Leaves membranous or chartaceous, uniformly pubescent on the lamina; trichomes stiff and antrorse; flowers 1–1.2 cm long. Bolivia, Peru	***Solanumleptocaulon***
54a	Anthers less than 1.5 mm long; calyx lobes strongly reflexed in mature fruit. Widespread throughout	***Solanumamericanum***
54b	Anthers more than 1.5 mm long; calyx lobes appressed or spreading in mature fruit	**55a**
55a	Mature berry shiny, usually 1–2 cm in diameter; anthers ochre-yellow, slightly tapering; seeds greater than 2 mm long; cultivated plants	***Solanumscabrum***
55b	Mature berries shiny or matte, less than 2 cm in diameter; anthers bright yellow; seeds less than 2 mm long; wild plants	**56a**
56a	Peduncle in fruit at right angles or more commonly strongly deflexed downwards; mature berries matte with a slightly glaucous bloom. Argentina, Brazil, Paraguay, Uruguay (adventive worldwide)	***Solanumchenopodioides***
56b	Peduncle in fruit not at right angles or deflexed downwards; mature berries shiny or somewhat shiny	**57а**
57а	Fruiting pedicels 1.5–1.7 cm long, strongly deflexed; corolla 1–2 cm in diameter. Colombia, Ecuador, Venezuela	***Solanummacrotonum***
57b	Fruiting pedicels less than 1.5 cm long, spreading; corolla less than 1 cm in diameter	**58a**
58a	Stone cells absent; fruiting pedicels 0.4–0.7 cm long, persistent; buds globose. Bolivia, Ecuador, Peru	***Solanumpseudoamericanum***
58b	Stone cells present; fruiting pedicels 1–1.2 cm long, not persistent; buds ellipsoid	**59a**
59a	Corolla 0.5–0.6 cm in diameter, the lobes strongly reflexed at anthesis; fruiting calyx lobes spreading. Bolivia, Ecuador, Peru	***Solanumlongifilamentum***
59b	Corolla 0.8–1 cm in diameter, the lobes spreading to slightly reflexed; fruiting calyx lobes appressed to the berry. Colombia, Ecuador, Venezuela	***Solanumnigrescens***
60a	Corolla campanulate; prostrate woody shrubs. Bolivia	***Solanumalbescens***
60b	Corolla variously stellate; herbs or weak shrubs (sometimes rhizomatous)	**61a**
61а	Inflorescence unbranched (rarely forked, if so, then unbranched inflorescences on the same plant)	**62a**
61b	Inflorescence always forked or many times branched	**69а**
62a	Small plants from underground rhizomes, the herbaceous above ground parts delicate. Argentina, Chile (introduced elsewhere)	***Solanumpygmaeum***
62b	Shrubs or herbs, the above ground parts not weak and delicate	**63a**
63a	Stems terete; leaves ovate to orbicular; buds globose. Bolivia	***Solanumalliariifolium***
63b	Stems ridged or angled at least in new growth; leaves elliptic or ovate; buds ellipsoid or narrowly ellipsoid	**64a**
64a	Subshrubs from a markedly woody base; pedicels inserted in an enlarged swelling of the inflorescence axis, clustered; plants sometimes with entire, toothed and deeply pinnatifid leaves on the same plant. Argentina, Paraguay	***Solanumsalicifolium***
64b	Coarse herbs of subshrubs to shrubs; pedicels not in an enlarged swelling of the inflorescence axis; leaves elliptic, more or less uniform in shape on a single plant	**65a**
65a	Anthers greater than 4 mm long	**66a**
65b	Anthers less than 4 mm long	**67a**
66a	Leaves not paired at the nodes (geminate); abaxial leaf surfaces almost glabrous; flower buds ellipsoid; calyx lobes deltate to triangular, the apices acute; corolla deeply stellate, lobed nearly to the base. Southeastern Brazil	***Solanumenantiophyllanthum***
66b	Leaves paired at the nodes (geminate); abaxial leaf surfaces evenly pubescent; flower buds globose; calyx lobes spathulate; corolla broadly stellate, lobed halfway to the base. Bolivia	***Solanumdianthum***
67a	Berries ellipsoid; fruiting pedicels markedly woody. Bolivia, Ecuador, Peru	***Solanumantisuyo***
67b	Berries globose; fruiting pedicels not markedly woody	**68a**
68a	Fruiting pedicels 1.5–1.7 cm long, strongly deflexed; anthers 2.7–4 mm long. Colombia, Ecuador, Venezuela	***Solanummacrotonum***
68b	Fruiting pedicels 1–1.2 cm long, spreading; anthers 1.7–3.4 mm long. Bolivia, Ecuador, Peru	***Solanumlongifilamentum***
69a	Pubescence of stems and leaves strongly antrorse and appressed; stems strongly angled. Northern Argentina	***Solanumtiinae***
69b	Pubescence of stems and leaves, if present, spreading or if appressed not markedly antrorse; stems terete or only weakly ridged and angled	**70a**
70a	Flower buds narrowly ellipsoid, ca. twice as long as wide; corolla deeply stellate, divided nearly to the base; anthers 4–5 mm long	**71a**
70b	Flower buds globose, ellipsoid or ovoid, less than twice as long as wide; corolla lobed ca. halfway to the base; anthers 2.5–4.5 mm long	**73а**
71a	Corolla 1.9–2.2 cm in diameter; buds striped with white and violet (this persisting in dried specimens); berries 1 cm in diameter or greater, translucent dark green at maturity. Peru, Bolivia	***Solanumpolytrichostylum***
71b	Corolla only to 1.5 cm in diameter; buds uniform in colour; berries less than 1 cm in diameter, green or purple, not markedly translucent	**72a**
72a	Berries 0.4–0.5 cm in diameter, matte; pedicels in fruit 1–1.2 cm long, spreading or slightly deflexed, not secund; flowers clustered in upper half of inflorescence branches; calyx lobes in flower 1–1.5 mm long. Argentina, Bolivia	***Solanumaloysiifolium***
72b	Berries 0.8–1 cm in diameter, somewhat shiny; pedicels in fruit 0.7–1 cm long, strongly deflexed and appearing secund on the inflorescence axis; flowers evenly spaced along the inflorescence axis; calyx lobes in flower 0.5–1 mm long. Argentina, Brazil	***Solanumpaucidens***
73a	Leaf margins ciliate; calyx lobes narrowly triangular. Argentina	***Solanumzuloagae***
73b	Leaf margins not ciliate; calyx lobes variously deltate to triangular	**74a**
74a	Plants small herbs to 50 cm from underground rhizomes; buds ovoid, somewhat tapered at the tips. Argentina, Bolivia	***Solanumrhizomatum***
74b	Plants coarse herbs or shrubs, usually greater than 50 cm high and from a woody base, not rhizomatous; buds globose or ellipsoid, not tapered at the tips	**75a**
75a	Leaves triangular in outline; leaf bases abruptly truncate to hastate (occasionally slightly cordate); coarse herbs, often of wet places	***Solanumpilcomayense***
75b	Leaves elliptic or ovate in outline; leaf bases acute to attenuate; shrubs or subshrubs with woody base, various habitats	**76a**
76a	Sympodial units difoliate and the leaves paired at the nodes (geminate); inflorescences opposite the geminate leaf pair, with 2–6 flowers (this species only very rarely with forked inflorescences and more than 6 flowers). Bolivia	***Solanumdianthum***
76b	Sympodial units difoliate or plurifoliate, the leaves not paired at the nodes (geminate); inflorescences internodal or terminal, with more than 10 flowers	**77a**
77a	Buds globose; style long-exserted from the anther cone at anthesis, longer than the anther cone; anthers 2.5–3.5 mm long. Argentina, Chile	***Solanumfurcatum***
77b	Buds ellipsoid; style exserted from the anther cone at anthesis but not longer than it; anthers 3.5–4.5 mm long	**78a**
78a	Inflorescence forked; fruiting pedicels strongly deflexed with a kink at the base; corolla 1.3–2 cm in diameter; leaf trichomes to 0.5 mm long, not markedly soft and spreading; small woody shrubs, usually ca. 1 m high or less. Bolivia, Chile, Peru	***Solanumgonocladum***
78b	Inflorescence many times branched; fruiting pedicels spreading; corolla 2–3 cm in diameter; leaf trichomes to 1 mm long, soft and spreading; highly variable in size, but usually large sprawling shrubs 1–3 m, often with branches to 5 m long. Argentina, Bolivia, Peru	***Solanumcochabambense***

### ﻿Synoptical character list for the morelloids of South America

Many of these species are polymorphic in a number of these characters; please refer to species descriptions for details.

Rhizomatous herbs or subshrubs: *S.echegarayi*, *S.profusum*, *S.pygmaeum*, *S.rhizomatum*, *S.riojense*, *S.sinuatirecurvum*, *S.tweedieanum*.

Plants with adventitious roots: *S.alliariifolium*, *S.corymbosum*, *S.palitans*, *S.radicans*, *S.tripartitum*.

Apparently annual plants: *S.americanum*, *S.annuum*, *S.corymbosum*, *S.gilioides*, *S.grandidentatum*, *S.michaelis*, *S.nitidibaccatum*, *S.palitans*, *S.physalifolium*, *S.pseudoamericanum*, *S.sarrachoides*, *S.triflorum*, *S.weddellii*, *S.woodii*.

Large shrubby plants: *S.americanum*, *S.arequipense*, *S.arenicola*, *S.chenopodioides*, *S.cochabambense*, *S.enantiophyllanthum*, *S.fiebrigii*, *S.furcatum*, *S.interandinum*, *S.juninense*, *S.macrotonum*, *S.nigrescens*, *S.pallidum*, *S.paucidens*, *S.pilcomayense*, *S.polytrichostylum*, *S.pseudoamericanum*, *S.salicifolium*, *S.sinuatiexcisum*, *S.subtusviolaceum*, *S.zuloagae*.

Plants from robust woody stems: *S.echegarayi*, *S.fragile*, *S.gonocladum*

Stems strongly winged (wings or angles > 0.4 mm wide): *S.corymbosum*, *S.fragile*, *S.grandidentatum*, *S.interandinum*, *S.marmoratum*, *S.pentlandii*, *S.radicans*, *S.salamancae*, *S.tiinae*.

Stems with “spinose” processes: *S.americanum*, *S.arequipense*, *S.cochabambense*, *S.dianthum*, *S.interandinum*, *S.macrotonum*, *S.marmoratum*, *S.nigrescens*, *S.paucidens*, *S.pentlandii*, *S.physalifolium*, *S.radicans*, *S.rhizomatum*, *S.salamancae*, *S.scabrum*, *S.tiinae*.

Stems with long glandular trichomes: *S.arenicola*, *S.caatingae*, *S.caesium*, *S.fiebrigii*, *S.fragile*, *S.gilioides*, *S.glandulosipilosum*, *S.grandidentatum*, *S.hunzikeri*, *S.juninense*, *S.michaelis*, *S.nitidibaccatum*, *S.physalidicalyx*, *S.physalifolium*, *S.profusum*, *S.sarrachoides*, *S.sinuatiexcisum*, *S.subtusviolaceum*, *S.tweedieanum*, *S.woodii*.

Stem pubescence strongly antrorse: *S.gonocladum*, *S.leptocaulon*, *S.salamancae*, *S.salicifolium*, *S.tiinae*, *S.triflorum*.

Stem pubescence soft and curling or tangled: *S.albescens*, *S.dianthum*, *S.riojense*, *S.sinuatirecurvum*, *S.weddellii*.

Stem pubescence dendritic (branched): *S.pallidum*.

Leaves sessile (petiole absent): *S.echegarayi*, *S.gilioides*, *S.huayavillense*, *S.hunzikeri*, *S.leptocaulon*, *S.profusum*, *S.riojense*, *S.salicifolium*, *S.sinuatirecurvum*, *S.tiinae*, *S.tripartitum*.

Leaves pinnatifid (compound or lobed more than halfway to the midrib): *S.annuum*, *S.gilioides*, *S.palitans*, *S.radicans*, *S.salicifolium*, *S.tripartitum*, *S.triflorum*.

Leaves deeply and regularly 3-lobed: *S.palitans*, *S.tripartitum*.

Leaves deeply and regularly 3-lobed: *S.radicans*.

Leaves fleshy, the margins often revolute: *S.echegarayi*, *S.riojense*, *S.sinuatirecurvum*, *S.triflorum*, *S.weddellii*.

Leaves glabrous: *S.corymbosum*, *S.echegarayi*, *S.huayavillense*, *S.palitans*, *S.tripartitum*.

Leaves with ciliate margins: *S.huayavillense*, *S.zuloagae*.

Bud globose: *S.americanum*, *S.annuum*, *S.corymbosum*, *S.fragile*, *S.grandidentatum*, *S.palitans*, *S.pentlandii*, *S.radicans*, *S.tripartitum*, *S.weddellii*.

Buds narrowly elliptic: *S.aloysiifolium*, *S.glandulosipilosum*, *S.polytrichostylum*, *S.triflorum*.

Inflorescences opposite the leaves: *S.dianthum*, *S.physalidicalyx*, *S.sarrachoides*.

Inflorescences many times branched (more than forked): (*S.aloysiifolium*), *S.cochabambense*, *S.corymbosum*, *S.fiebrigii*, *S.huayavillense*, *S.interandinum*, *S.juninense*, *S.pallidum*, *S.pentlandii*, (*S.polytrichostylum*), *S.tripartitum*, *S.zuloagae*.

Corolla deeply stellate, interpetalar tissue appearing absent: *S.aloysiifolium*, *S.arenicola*, *S.chenopodioides*, *S.salicifolium*, *S.triflorum*.

Corolla pentagonal or broadly rotate: *S.annuum*, *S.caesium*, *S.corymbosum*, *S.gilioides*, *S.palitans*, *S.physalifolium*, *S.riojense*, *S.sarrachoides*, *S.sinuatirecurvum*, *S.weddellii*.

Corolla campanulate (very shallowly lobed and cup-shaped): *S.albescens*, *S.fiebrigii*, *S.leptocaulon*, *S.sinuatiexcisum*.

Flowers (pale) yellow: *S.huayavillense*.

Anthers less than 1.5 mm long: *S.americanum*, *S.annuum*, *S.corymbosum*, *S.gilioides*, *S.marmoratum*, *S.nitidibaccatum*, *S.weddellii*.

Anthers more than 5 mm long: *S.dianthum*, *S.gonocladum*, *S.hunzikeri*, *S.tweedieanum*.

Styles long-exserted (exserted portion longer than the anthers) from the anther cone: *S.arequipense*, *S.fragile*, *S.furcatum*, *S.pentlandii*, *S.pseudoamericanum*.

Style included within the anther cone (or just barely exserted): *S.americanum*, *S.marmoratum*, *S.weddellii*.

Berries less than 1 cm in diameter: *S.aloysiifolium*, *S.annuum*, *S.echegarayi*, *S.longifilamentum*, *S.paucidens*.

Berries depressed or flattened (sub-ovoid): *S.cochabambense*, *S.michaelis*, *S.palitans*, *S.radicans*, *S.tripartitum*.

Berries ellipsoid: *S.antisuyo*, *S.gilioides*, *S.weddellii*.

Berries 2-lobed (easier to see in immature fruit): *S.palitans*, *S.radicans*, *S.tripartitum*.

Mature berries orange or yellow: *S.alliariifolium*, *S.caesium*, *S.palitans*, *S.riojense*, *S.radicans*.

Mature berries red: *S.corymbosum*, *S.tripartitum*.

Mature berries translucent green marbled with white: *nitidibaccatum*, *physalifolium*, *marmoratum*.

Mature berries shiny: *S.americanum*, *S.nitidibaccatum*, *S.marmoratum*, *S.physalifolium*, *S.sarrachoides*, *S.scabrum*.

Fruiting pedicels persistent after fruit ripening (i.e., lack of abscission): *S.americanum*, *S.antisuyo*, *S.sinuatirecurvum*.

Fruiting calyx accrescent (inflated or appressed): *S.annuum*, *S.gilioides*, *S.hunzikeri*, *S.marmoratum*, *S.michaelis*, *S.nitidibaccatum*, *S.physalidicalyx*, *S.physalifolium*, *S.profusum*, *S.salamancae*, *S.sarrachoides*, *S.tweedieanum*, *S.weddellii*.

Fruiting calyx inflated and completely enclosing the berry, invaginate at the base: *S.physalidicalyx*, *S.salamancae*.

Seeds fewer than 10 per berry: *S.annuum*, *S.echegarayi*, *S.gilioides*, *S.huayavillense*, *S.sinuatirecurvum*, *S.weddellii*.

Seeds more than 50 per berry: *S.aloysiifolium*, *S.caatingae*, *S.caesium*, *S.fiebrigii*, *S.interandinum*, *S.juninense*, *S.marmoratum*, *S.nigrescens*, *S.paucidens*, *S.pilcomayense*, *S.polytrichostylum*, *S.pygmaeum*, *S.sarrachoides*, *S.scabrum*, *S.sinuatiexcisum*, *S.triflorum*.

Seeds tuberculate: *S.annuum*, *S.gilioides*, *S.weddellii*.

Stone cells absent: *S.americanum*, *S.annuum*, *S.antisuyo*, *S.arequipense*, *S.caatingae*, *S.chenopodioides*, *S.fragile*, *S.gilioides*, *S.grandidentatum*, *S.huayavillense*, *S.michaelis*, *S.nitidibaccatum*, *S.pentlandii*, *S.physalidicalyx*, *S.physalifolium*, *S.profusum*, *S.pseudoamericanum*, *S.riojense*, *S.scabrum*, *S.sinuatirecurvum*, *S.weddellii. S.woodii*.

Stone cells 10 or more per berry: *S.aloysiifolium*, *S.cochabambense*, *S.echegarayi*, *S.furcatum*, *S.glandulosipilosum*, *S.hunzikeri*, *S.interandinum*, *S.nigrescens*, *S.salicifolium*, *S.triflorum*.

### ﻿Species descriptions

#### ﻿1. Solanum albescens

Taxon classificationPlantaeSolanalesSolanaceae

(Britton) Hunz., Kurtziana 4: 137. 1967.

Figs 6 , 7

Poecilochroma albescens Britton, Mem. Torrey Bot. Club 4: 91. 1896. Type. Bolivia. La Paz: vic. Mapiri, 8,000 ft., Sep 1892, *M. Bang 1575* (lectotype, designated by Bitter 1918, pg. 154; second step, designated here: W [acc. # 0100785 (1893–5558)]; isolectotypes: BM [BM000887671], E [E00504823], F [v0093033F], GH [00077569], K [K000590161, K000590162], M [M-0171597], MICH [MICH1109891], MO [MO-171597, acc. # 2218441], NY [00022197], PH [00020419], US [00027289, acc. # 1416199; 01014184, acc. # 98763], WU [acc. # 0120000]).
Capsicum albescens (Britton) Kuntze, Revis. Gen. Pl. 3[3]: 218. 1898 [28 Sep 1898]. Type. Based on Poecilochromaalbescens Britton.
Solanocharis albescens (Britton) Bitter, Repert. Spec. Nov. Regni Veg. 15: 153. 1918. Type. Based on Poecilochromaalbescens Britton.

##### Type.

Based on *Poecilochromaalbescens* Britton.

**Figure 6.**
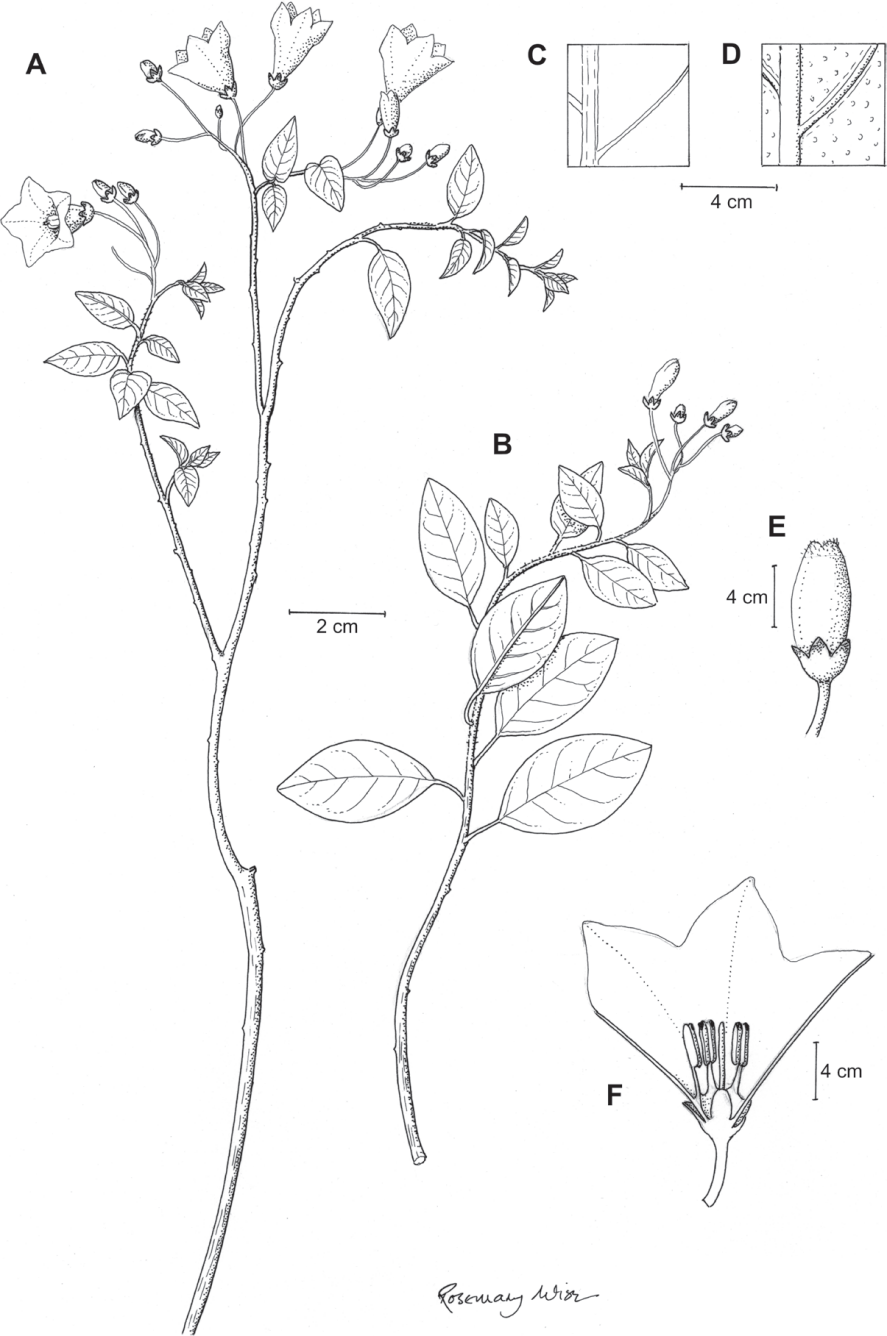
*Solanumalbescens***A** flowering branch **B** branch with flower buds **C** detail of abaxial leaf surface **D** detail of adaxial leaf surface **E** bud **F** dissected flower (**A–F***Brooke 6905*). Illustration by R. Wise.

##### Description.

Straggling shrublet to 0.5 m high, the branches often rooting where in contact with the soil, woody at the base. Stems terete, densely pubescent with eglandular 4–5-celled simple uniseriate trichomes ca. 0.5 mm long, these antrorse, crisped and curly at the tips, the basal cell enlarged; new growth sparsely pubescent with eglandular simple uniseriate 4–5-celled trichomes ca. 0.5 mm long along the veins and margins, these curled at the tips like those of the stem; bark of older stems whitish yellow, glabrescent, somewhat corky and peeling. Sympodial units plurifoliate, the leaves not geminate. Leaves simple, the blades 1–4 cm long, 0.4–1.9 cm wide, elliptic, widest at the middle, thick and fleshy or somewhat rubbery-coriaceous, discolorous and paler beneath; adaxial surfaces glabrous, with a few simple uniseriate trichomes along the sunken midrib; abaxial surfaces glabrous or with a few simple uniseriate trichomes scattered on veins and lamina; principal veins 3–4 pairs, sunken or obscure adaxially, drying yellowish; base acute; margins entire, slightly revolute and ciliate with simple uniseriate trichomes ca. 0.5 mm long; apex acute, the tip sometimes slightly rounded; petioles 0.1–0.3 cm long, glabrous or with a few scattered simple uniseriate trichomes adaxially. Inflorescences terminal or opposite the leaves, unbranched, 0.5–2 cm long, with 2–5 flowers clustered at the tips, glabrous to sparsely pubescent in the lower half (peduncle) with simple uniseriate 4–5-celled curly trichomes like those of the stems; peduncle 0.4–0.5 cm long; pedicels 1.5–2 cm long, ca. 0.5 mm in diameter at the base, ca. 1 mm in diameter at the apex, filiform and spreading or drooping, glabrous, articulated at the base leaving a tiny sleeve or peg ca. 0.5 mm long; pedicel scars 1–2 mm apart in the distal part of the inflorescence. Buds ellipsoid, the corolla strongly exserted from the calyx before anthesis. Flowers 5-merous, cosexual (hermaphroditic). Calyx tube 1.5–2 mm long, cup-shaped, the lobes 1–1.5 mm long, ca. 1.5 mm wide, deltate with a strongly swollen tip, this fleshy(?) tip drying dark and with a few simple uniseriate trichomes 0.1–0.2 m long at the very apex. Corolla 3–4 cm in diameter, 1.5–1.8 cm long, white, campanulate, lobed 1/8–1/6 of the way to the base, the lobes 3–5 mm long, 5–5.5 mm wide, the lobes not spreading, slightly curved inwards, adaxially glabrous, abaxially densely papillate, the papillae denser along the tips and margins. Stamens equal; filament tube minute; free portion of the filaments 1–1.5 mm long, glabrous; anthers 2.5–3.2 mm long, ellipsoid, yellow, poricidal at the tips, the pores lengthening to slits with age. Ovary conical, glabrous; style 7–7.5 mm long, straight, exserted beyond the anther cone, glabrous, fully included within the campanulate corolla; stigma minute, merely a widening of the style tip, the surfaces minutely papillate. Fruit and seeds not known. Chromosome number not known.

**Figure 7.**
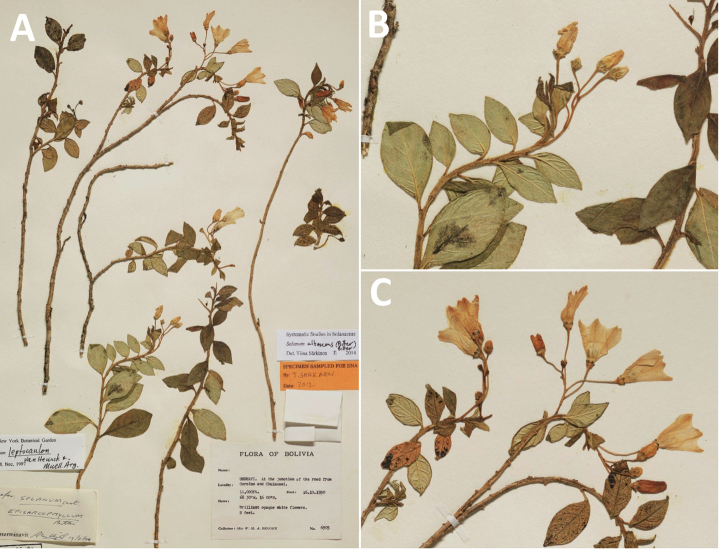
*Solanumalbescens***A** habit **B** habit with flower buds **C** habit with flowers at anthesis (**A–C***Brooke 6905* [BM000887669]). Reproduced with permission of the Trustees of the Natural History Museum.

##### Distribution

**Figure 8.**
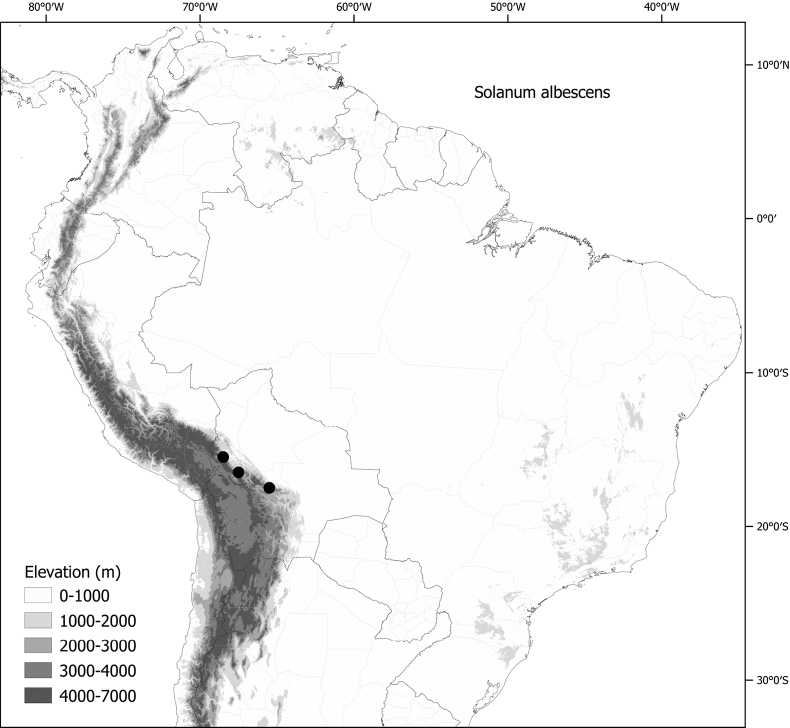
Distribution map of *Solanumalbescens*.

**(Fig. 8).***Solanumalbescens* is endemic to the Bolivian Andes (Depts. Cochabamba, La Paz).

##### Ecology and habitat.

*Solanumalbescens* is a plant of cloud forests and grassy areas near treeline on steep slopes, from 2,700 to 3,500 m elevation.

##### Common names and uses.

Bolivia. La Paz: kurpusa (*Girault s.n.*). No uses recorded.

##### Preliminary conservation status

**(IUCN 2022).** Endangered [EN – B1,2ab(ii, iv) D2]. EOO = 7,953 km^2^ [VU]; AOO = 20 km^2^ [EN]. The EOO would suggest that *S.albescens* should be assessed as Vulnerable, but the small number of known populations (< 5) and the paucity of recent collections suggest an Endangered status based on the AOO is more realistic. *Solanumalbescens* has not been collected within any protected area.

##### Discussion.

*Solanumalbescens* is an unusual species in the Morelloid clade with large, campanulate corollas and a woody creeping habit. It was first described as a member of the genus *Peocilochroma* Miers (Miers 1848) on the basis of its corolla shape (*Poecilochroma* is now considered a synonym of the genus *Saracha* Ruiz & Pav.). Bitter (1918) later recognised it as a monospecific genus *Solanocharis* Bitter unrelated to *Poecilochroma*, but he did not suggest relationships due to lack of mature fruit on the few specimens he examined. Hunziker (1967) recognised it as a species of *Solanum* based on the morphology of the androecium and inflorescence, and considered it related to *S.macbridei* Hunz. & Lallana (a member of the Dulcamaroid clade with similar pedicel sleeves, campanulate corollas and small, leathery leaves; Knapp 2013). Child (1994) erected SolanumsectionSolanocharis, to include *S.albescens*, *S.rheithrocharis* Bitter (here considered a synonym of *S.leptocaulon*), *S.leptocaulon* and *S.poecilochromifolium* Rusby (as ‘*poecilochromophyllum*’, here considered a synonym of *S.gonocladum*) based on their habit as decumbent shrubs with somewhat campanulate corollas.

*Solanumalbescens* is morphologically similar to *S.leptocaulon*, sharing with it a decumbent habit, high elevation distribution and campanulate flowers. The species differ in leaf texture (those of *S.albescens* are much more leathery/coriaceous than those of *S.leptocaulon*), pubescence (*S.albescens* has curling trichomes confined to the stems or only on leaf margins, while the leaves of *S.leptocaulon* are often pubescent on the lamina and the trichomes are stiff and usually antrorse) and flower size (1.5–2 cm long in *S.albescens* and 1–1.2 cm long in *S.leptocaulon*). Although the anthers of both species are similar in size (2.5–3 mm long) the relative size of the anthers is strikingly smaller in *S.albescens* due to the much larger corolla. The tips of the calyx lobes in *S.albescens* appear to be fleshy and somewhat swollen but this needs confirmation with field examination.

In some publications and databases, the authorship of *Poecilochromaalbescens* is given as Britton ex Rusby because it was published in an enumeration of plants collected by Miguel Bang that was authored by H.H. Rusby (Rusby 1896). In the protologue the name *Poecilochromaalbescens* was explicitly attributed to N.L. Britton, and we are following the International Plant Names Index (https://ipni.org/n/204701-2) in attributing both name and description to Britton. In the protologue of *Poecilochromaalbescens* (Rusby 1896) two collections were cited: *Bang 1575* and *Rusby 2563*, without citing a specific herbarium. In describing his new genus *Solanocharis* Bitter, Georg Bitter (1918) cited only *Bang 1575* as type, thus effectively lectotypifying the name, but he cited four herbaria (“herb. Berol., Monac., Vindob., Vratisl.”). We narrow this here and select the sheet of *Bang 1575* held in Vienna (W) cited by Bitter (1918) as “Vindob.” as the second step lectotype; it is the best preserved of the duplicates cited by Bitter that we have seen.

#### ﻿2. Solanum alliariifolium

Taxon classificationPlantaeSolanalesSolanaceae

M.Nee & Särkinen, PhytoKeys 47: 99. 2015.

Figs 9 , 10

##### Type.

Bolivia. Santa Cruz: Prov. Vallegrande: 6.5 km by air SW of Guadalupe on road to Pucará, at turnoff to Santa Ana, 18°36'S, 64°07'W, 2,675 m, 15 Dec 1990, *M. Nee 40315* (holotype: LPB; isotypes: MO [MO-2537105, acc. # 6458011], NY [00852828], USZ).

**Figure 9.**
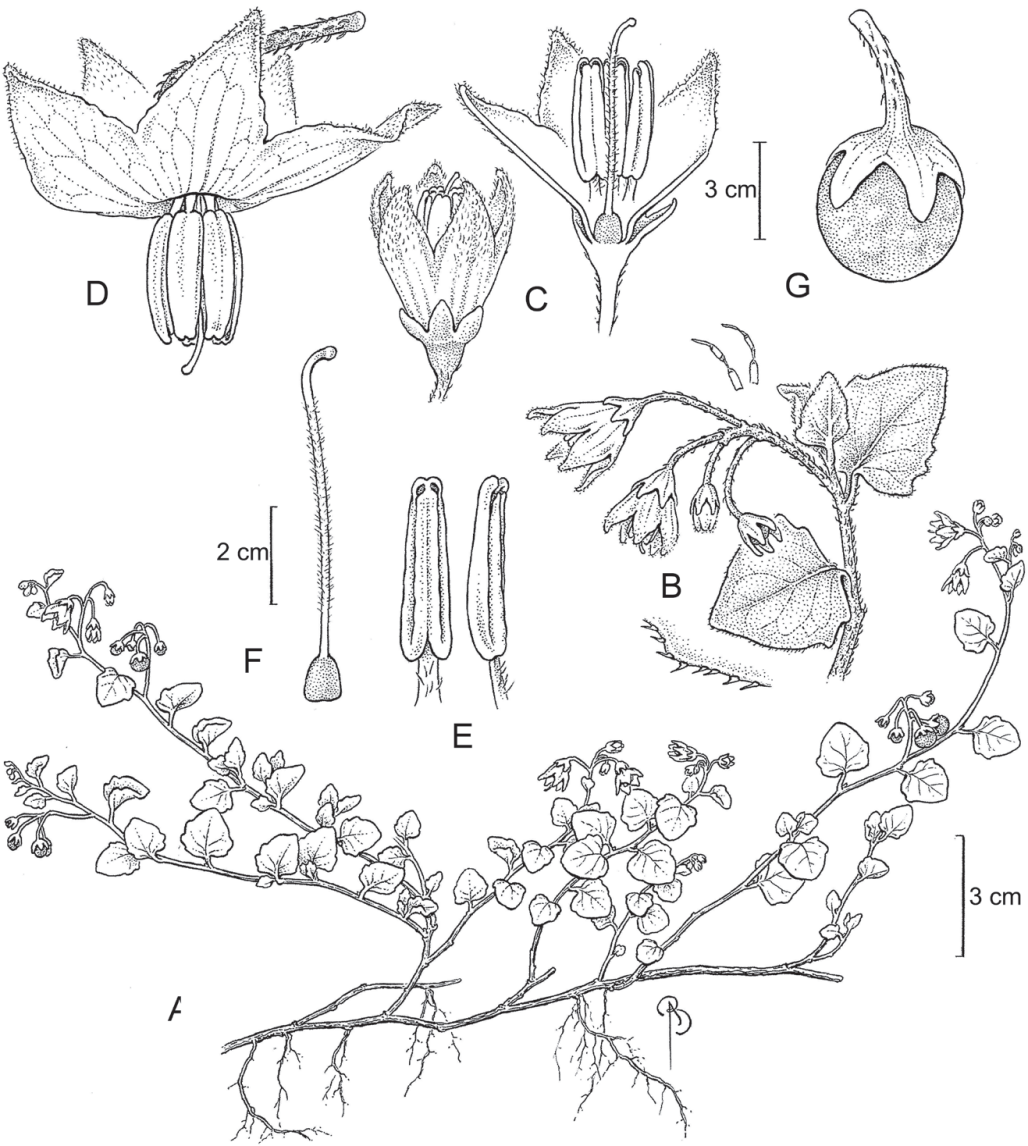
*Solanumalliariifolium***A** habit **B** inflorescence with details of pubescence and ciliate leaf margins **C** flower just prior to anthesis, with and without corolla lobes removed **D** flower at anthesis **E** stamens **F** gynoecium **G** fruit (**A–C, E–G***Nee 40315***D***Vargas 787*). Illustration by B. Angell. Previously published in Särkinen et al. (2015d: 100).

##### Description.

Slender perennial herb to 0.3 m high, with multiple long, creeping stems arising from a central taproot, stems up to 50 cm long, rooting at nodes. Stems terete, glabrous or sparsely pubescent with spreading translucent 4–6-celled simple uniseriate trichomes ca. 0.2 mm long. Sympodial units difoliate, not geminate. Leaves simple, the blades 1.5–3.6 cm long, 0.9–2.3 cm wide, broadly ovate to orbicular, widest at the middle or in the lower third, membranous, concolorous; adaxial surface glabrous; abaxial surface glabrous or sparsely pubescent with appressed 1–3-celled simple uniseriate trichomes along veins and leaf margins; principal veins 3–4 pairs; base rounded to attenuate, occasionally decurrent; margins entire, undulate, or shallowly lobed; apex acute; petiole 0.7–1.5 cm long, sparsely pubescent with simple 1–3-celled uniseriate trichomes like those of the stems, especially on young leaves. Inflorescences opposite the leaves, unbranched, 1.5–3 cm long, with 2–6 flowers, sparsely pubescent with simple uniseriate 4–6-celled spreading trichomes; peduncle 1–3 cm long, 0.4–0.5 mm in diameter at the apex and 0.6 mm in diameter at the base; pedicels 0.6–0.9 cm long, ca. 0.4 mm in diameter at the base and ca. 0.5 mm in diameter at the apex, straight and spreading at anthesis, articulated at the base; pedicel scars spaced 0.2–1.5 mm apart. Buds globose, white or purple-tinged. Flowers 5-merous, cosexual (hermaphroditic). Calyx tube ca. 1.4–1.5 mm long, the lobes 1.6–2 mm long, rectangular in outline with rounded to acute apices, somewhat spreading at anthesis, sparsely pubescent with simple 1–4-celled uniseriate trichomes. Corolla 1.4–1.6 cm in diameter, white to pale or deep violet-blue, with a dark purple ring against yellow-green central star at the base, stellate, lobed to the middle, the lobes ca. 4–5 mm long, 2–2.5 mm wide, reflexed at anthesis, densely pubescent abaxially with 1–2-celled simple uniseriate trichomes, these usually shorter than the trichomes of stems and leaves. Stamens equal; filament tube minute; free portion of the filaments 1.1–1.6 mm long, pubescent with 4–7-celled uniseriate trichomes at the base adaxially; anthers 3.5–4 mm long, 0.8–1 mm wide, ellipsoid to rectangular in outline, yellow, poricidal at the tips, the pores lengthening to slits with age. Ovary globose, glabrous; style 5–6 mm long, straight, exserted beyond the anther cone, densely pubescent with 2–3-celled simple uniseriate trichomes in the basal 2/3; stigma clavate, minutely papillate. Fruit a globose berry, 0.4–0.5 cm in diameter, green when developing, mature berries unknown, the pericarp thin, matte, opaque, glabrous; fruiting pedicels 1.1–3.2 cm long, ca. 0.4 mm in diameter at the base, ca. 0.6 mm in diameter at the apex, spreading, becoming somewhat woody, not persistent; fruiting calyx lobes 2.8–3.2 mm long, spreading. Seeds (10)15–20 per berry, ca. 1.5–1.7 mm long, ca. 1.2–1.3 mm wide, flattened, reniform, pale-brown, the sub-lateral hilum positioned close to the middle, the testal cells pentagonal in outline. Stone cells ca. 2 per berry, ca. 0.5 mm in diameter, cream-coloured. Chromosome number: not known.

**Figure 10.**
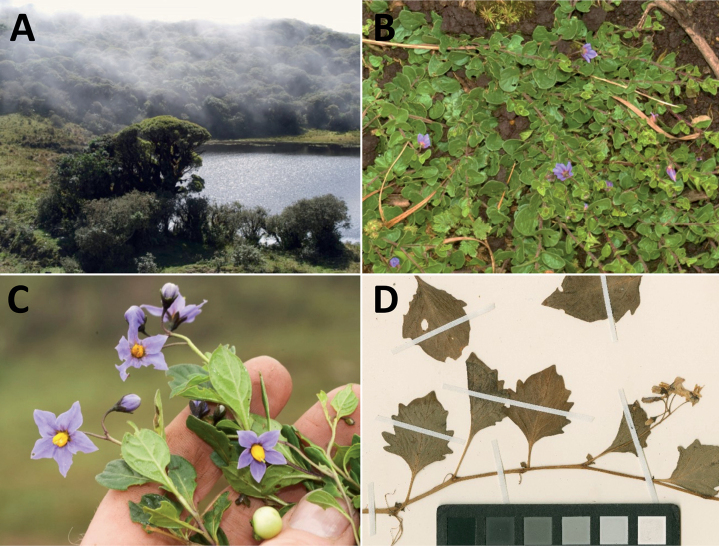
*Solanumalliariifolium***A** habitat **B** habit **C** flowering and fruiting branch **D** creeping stem rooting along nodes (**A–C***Nee & Wen 53903***D***Ochoa 12022* [US 02999214, acc. # 2982650], reproduced with permission of the Smithsonian Institution). Photos of live plants and habitat by M. Nee.

##### Distribution

**Figure 11.**
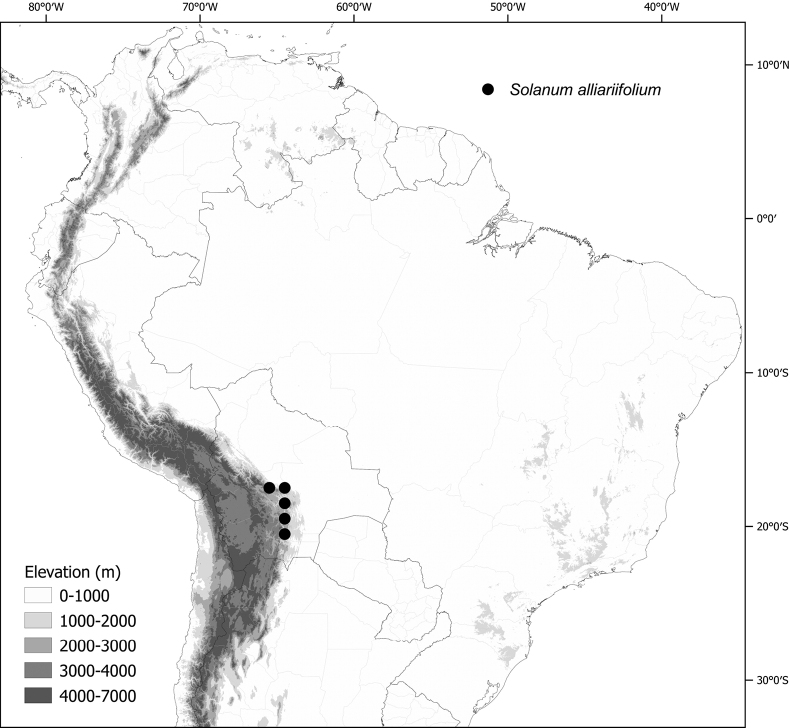
Distribution map of *Solanumalliariifolium*.

**(Fig. 11).***Solanumalliariifolium* is endemic to the eastern Bolivian Andes (Depts. Chuquisaca, Cochabamba, Santa Cruz).

##### Ecology and habitat.

*Solanumalliariifolium* is found in montane forests with *Podocarpusparlatorei* Pilg. (Podocarpaceae) and *Alnusacuminata* Kunth (Betulaceae) often in open areas close to water sources, near rivers and moist depressions, and marshy meadows on sandy or rocky substrate, between 1,900 and 3,200 m elevation.

##### Common names and uses.

None recorded.

##### Preliminary threat status

**(IUCN 2022).** Vulnerable [VU – B1, 2a,b(ii, iv), D2]. EOO = 18,992 km^2^ [VU]; AOO 56 km^2^ [EN]. Särkinen et al. (2015c) also assigned a preliminary IUCN threat status of Vulnerable (VU, B1) to *S.alliariifolium* based on the small extent of occurrence. No occurrences are known within the protected area network in Bolivia thus far, but collection data indicate that the species endures grazing pressures relatively well. We have no additional data with which to change this initial assessment.

##### Discussion.

*Solanumalliariifolium* is distinct within the morelloids in being a slender creeping herb rooting along nodes, with broadly ovate to orbicular leaves with crenate to shallowly lobed margins. It is morphologically most similar to *S.leptocaulon*, which occurs in similar montane habitats in Bolivia and in southern Peru, but the latter species is a small scrambling shrublet with entire-margined, ovate-lanceolate leaves. *Solanumleptocaulon* further differs from *S.alliariifolium* in having a campanulate corolla lobed only 1/3 of the way to the base, rather than a stellate corolla lobed to 2/3 to the base with the lobes clearly reflexed at anthesis.

#### ﻿3. Solanum aloysiifolium

Taxon classificationPlantaeSolanalesSolanaceae

Dunal, Prodr. [A. P. de Candolle] 13(1): 73. 1852.

Figs 2B , 12 , 13

Solanum filiforme Ruiz & Pav. var. lanceolatum Kuntze, Revis. Gen. Pl. 3(2): 225. 1898. Type. Argentina. Jujuy: sin. loc., *P.G. Lorentz & G. Hieronymus 1074* (lectotype, designated by Barboza et al. 2013, pg. 236: NY [00688918]).
Solanum lorentzii Bitter, Repert. Spec. Nov. Regni Veg. 11: 2. 1912. Type. Argentina. Achiral, bei San Andres, Sep 1873, *P.G. Lorentz & G. Hieronymus 440* (lectotype, designated here: CORD [CORD00004234]; isolectotypes: CORD [CORD00004235], GOET).
Solanum oligodontum Bitter, Repert. Spec. Nov. Regni Veg. 11: 215. 1912. Type. Bolivia. Tarija: “Huayavilla, Bolivia australis, apud coloniam”, 1903–1904, *K. Fiebrig 3428* (holotype: B, destroyed [F neg. 2718], no duplicates found).
Solanum bermejense Bitter, Repert. Spec. Nov. Regni Veg. 13: 87. 1913. Type. Bolivia [Argentina]. Bermejo, 17 Nov 1903, *K. Fiebrig 2131* (lectotype, designated by Barboza et al. 2013, pg. 236: SI [003294, acc. # 065940]; isolectotypes: GOET [003485], HBG [HBG511408], M [M0171774]).
Solanum polytrichostylum Bitter var. lorentzii (Bitter) Edmonds, Kew. Bull. 27: 106. 1972. Type. Based on Solanumlorentzii Bitter.
Solanum collectaneum C.V.Morton, Revis. Argentine Sp. Solanum 67. 1976. Type. Argentina. Tucumán: Dpto. Capital, Río Salí, 19 Sep 1920, *S. Venturi 919* (holotype: US [00027521, acc. # 1548836]; isotypes: A [00077601], SI [003299, acc. # 162492; 065950, acc. # 065950]).

##### Type.

Bolivia. Chuquisaca: “In Boliviae collibus siccis Chuquisaca”, Dec, *A. D’Orbigny 1208* (holotype: P [P00319385]; isotype: F [v0073195F]).

**Figure 12.**
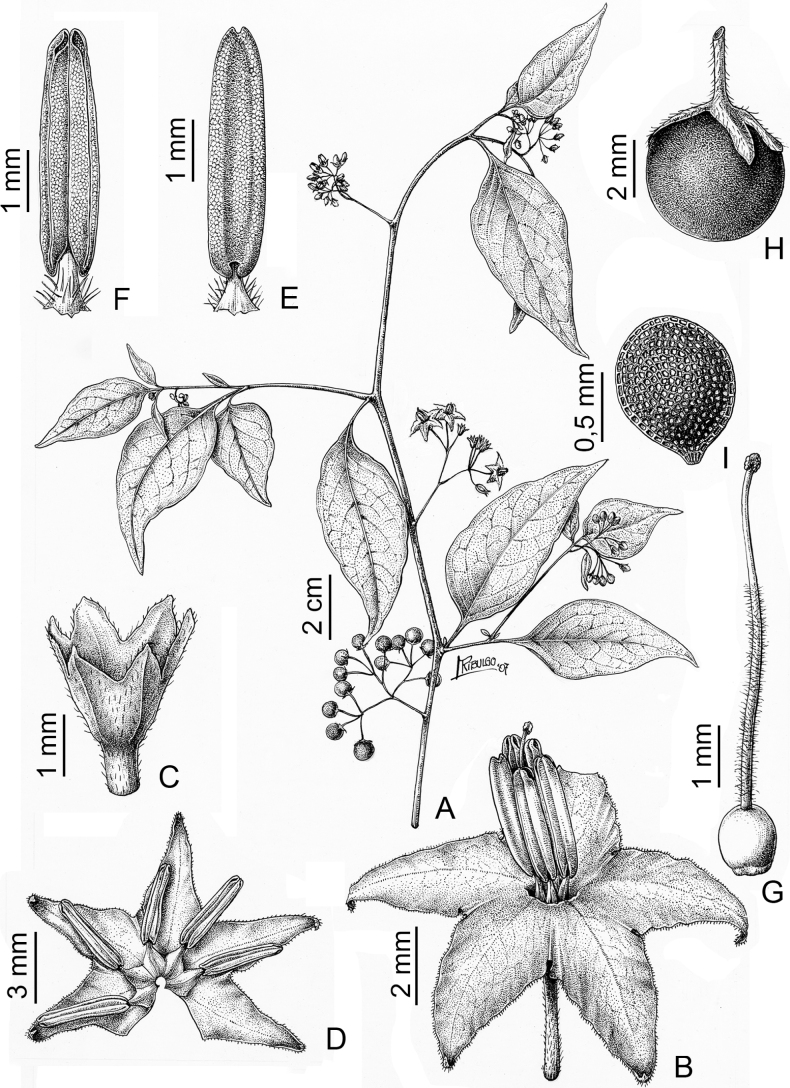
*Solanumaloysiifolium***A** flowering and fruiting branch **B** flower **C** calyx **D** dissected flower **E** stamen, dorsal view **F** stamen, ventral view **G** gynoecium **H** fruit **I** seed (**A–I***Barboza et al. 1072*). Illustration by L. Ribulgo.

##### Description.

Shrub to 4 m, woody or subwoody, often with lax sprawling branches, occasionally growing as a small herb. Stems terete, sparsely pubescent with white eglandular 2–3(5)-celled simple uniseriate trichomes to 0.5 mm long, these appressed and usually antrorse; new growth densely pubescent with white eglandular simple uniseriate trichomes like those of the stems, appearing whitish grey in herbarium specimens; bark of older stems pale green-grey. Sympodial units difoliate, the leaves not geminate. Leaves simple, the blades 3–10(19) cm long, 1.5–5(9) cm wide, elliptic to narrowly elliptic, widest at or just below the middle, membranous to chartaceous, concolorous; adaxial surfaces nearly glabrous to sparsely and evenly pubescent with white eglandular simple uniseriate trichomes ca. 0.5 mm long, the pubescence denser on the veins; abaxial surfaces sparsely to moderately pubescent with similar white simple uniseriate trichomes; principal veins 5–6 pairs, more densely pubescent than the lamina; base truncate-attenuate to attenuate, not markedly decurrent onto the stem; margins entire or occasionally irregularly dentate in the basal half; apex acute; petioles 0.3–1 cm long, sparsely to moderately pubescent with white eglandular simple uniseriate trichomes ca. 0.5 mm long like the stems and venation. Inflorescences internodal, forked or occasionally twice-forked, 1.5–4 cm long, with 10–20 flowers borne at the upper half of each branch, moderately to densely pubescent with white eglandular simple uniseriate trichomes to 0.5 mm long, more densely pubescent than the stem; peduncle 0.5–1.8 cm long; pedicels 0.6–0.8 cm long, 0.4–0.5 mm in diameter at the base, ca. 1 mm in diameter at the apex, tapering, spreading at anthesis, pubescent with white simple uniseriate trichomes like the rest of the inflorescence, the pubescence sparser than on the inflorescence axis, articulated at the base; pedicel scars irregularly spaced 0.5–1 mm apart, slightly raised from the inflorescence axis. Buds long-ellipsoid, the corolla strongly exserted from the calyx before anthesis. Flowers 5-merous, cosexual (hermaphroditic). Calyx tube 1–1.2 mm long, conical, the lobes 1–1.5 mm long, 1–1.5 mm wide, deltate with an acute tip, sparsely pubescent with white eglandular simple uniseriate trichomes to 0.5 mm long like the pedicels. Corolla 1.2–1.5 cm in diameter, white or occasionally purple-tinged, with a yellowish green central star often with darker margins, stellate, lobed 3/4 of the way to the base, the lobes 4–5 mm long, 1.5–2 mm wide, reflexed at anthesis and spreading with age, glabrous adaxially, sparsely and minutely puberulent and papillate abaxially long the petal midveins tips and margins, the trichomes sparse, to 0.2 mm long, spreading. Stamens equal or occasionally slightly unequal; filament tube minute; free portion of the filaments 0.5–1 mm long, pubescent adaxially with eglandular tangled simple uniseriate trichomes; anthers 4–4.5(5.5) mm long, 0.6–0.7(1) mm wide, narrowly ellipsoid, yellow, poricidal at the tips, the pores lengthening to slits with age. Ovary conical, glabrous; style 6.5–8 mm long, straight, exserted beyond the anther cone, densely papillate-pubescent in the lower 2/3 where included in the anther cone; stigma capitate to small-capitate, the surfaces minutely papillate. Fruit a globose berry, 0.4–0.5 cm in diameter, green or purple to greenish purple when ripe, the pericarp thin, matte, opaque, glabrous; fruiting pedicels 1–1.2 cm long, ca. 0.5 mm in diameter at the base, ca. 1 mm in diameter at the apex, not markedly woody, deflexed to slightly spreading, not persistent; fruiting calyx not accrescent, appressed to the berry, the lobes to 1.5 mm long, occasionally somewhat spreading or reflexed. Seeds 40–60 per berry, 1–1.5 mm long, 0.9–1 mm wide, teardrop shaped, pale tan or yellowish brown, the surfaces minutely pitted, the testal cells shallowly sinuate in outline (nearly rectangular). Stone cells 10 per berry, 4 larger to ca. 1 mm in diameter, 6 smaller ca. 0.5 mm in diameter, all scattered through the berry flesh, cream-coloured. Chromosome number: n = 12 (Moscone 1992, as *S.lorentzii* and S.lorentziivar.montigenum; vouchers *Hunziker et al. 24691, 24711, 24872, 24826, 24833*, *Subils et al. 3478, 3496, 3497, 3499*; Moyetta et al. 2013; voucher *Barboza et al. 2210*).

**Figure 13.**
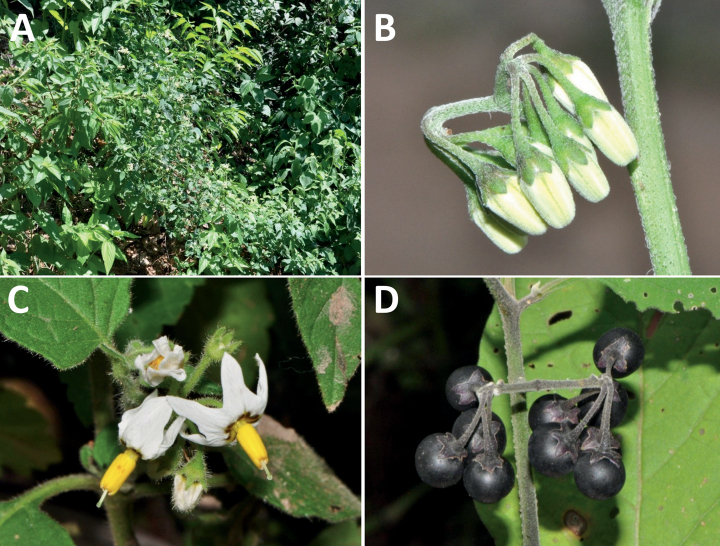
*Solanumaloysiifolium***A** habit **B** inflorescence in bud **C** flowers at full anthesis **D** fully mature fruits (**A***Barboza et al. 3506***B***Barboza et al. 3566***C***Barboza et al. 3565***D***Barboza et al. 3532*). Photos by S. Knapp.

##### Distribution

**Figure 14.**
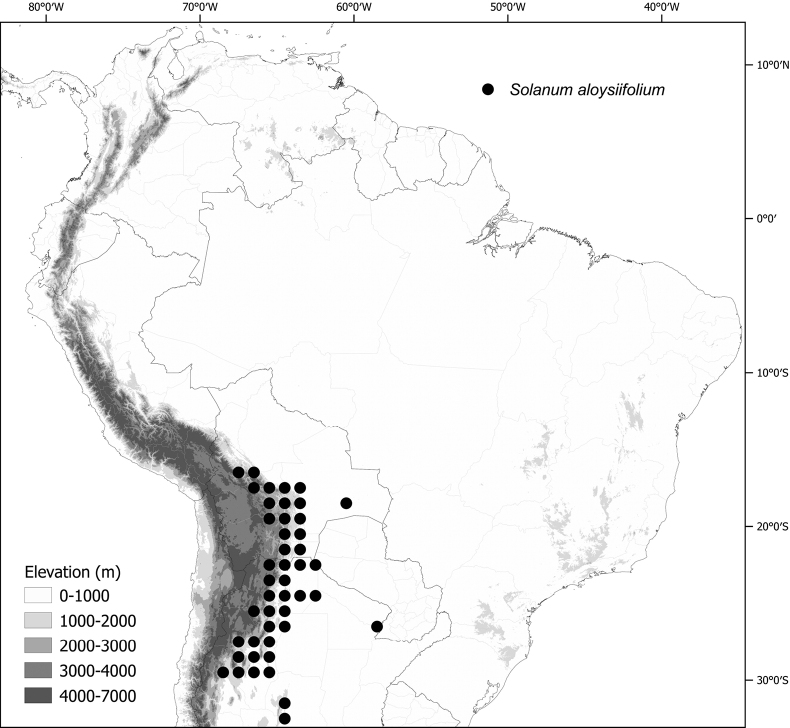
Distribution map of *Solanumaloysiifolium*.

**(Fig. 14).***Solanumaloysiifolium* is widely distributed and occurs from central Bolivia to central Argentina. Its southernmost range only just reaches Córdoba Province in central Argentina and it is much more common further north and westward towards the Andes.

##### Ecology and habitat.

*Solanumaloysiifolium* is a weedy species, often growing along roadsides and in open, disturbed areas in a wide variety of habitats, from 100 to 3,000 m elevation. Plants often occur in large patches and can be remarkably morphologically divergent in different habitats.

##### Common names and uses.

Argentina. Salta: papa de la vibora (*Hilgert & Lamas 1691*), sacha ají (*Anon. s.n.*, 7 Jun 1905), yerba mora (*Hilgert 2519, 2251*). Used medicinally in local communities around Parque Nacional Baritú in montane Salta, Argentina (Hilgert and Gil 2006).

##### Preliminary conservation status

**(IUCN 2022).** Least Concern [LC]. EOO = 1,349,765 km^2^ [LC]; AOO = 1,596 km^2^ [VU]. *Solanumaloysiifolium* is a common and widespread species that occurs in several protected areas in both Bolivia and Argentina. It grows in large stands in disturbed areas over its entire range. The small AOO certainly reflects collecting and georeferencing deficit.

##### Discussion.

*Solanumaloysiifolium* is widely distributed and a common plant of disturbed areas in northern Argentina. It often forms large stands and can range from tiny shrubs to almost tree-like forms. Like many species of morelloids (e.g., *S.nigrum*, see Särkinen et al. 2018) fruit colour is polymorphic with plants having either green or purple berries at maturity. The long-pedunculate forked inflorescences with narrowly elliptic buds that develop into deeply stellate corollas with a greenish yellow central star of shiny tissue make this species distinctive.

Barboza et al. (2013) placed *S.cochabambense* in synonymy with *S.aloysiifolium*, but further study throughout the range of *S.cochabambense* confirmed the distinctness of the two taxa. Individual specimens collected in sympatry can be difficult to identify. *Solanumcochabambense* differs from *S.aloysiifolium* in its more highly branched inflorescences (those of *S.aloysiifolium* are usually only forked), buds that are ellipsoid with long-triangular calyx lobes rather than narrowly ellipsoid with short-triangular calyx lobes and larger less deeply stellate corollas. The anthers of *S.aloysiifolium* are narrow relative to their length (3.9–5 mm long and 0.6–1 mm wide in *S.aloysiifolium* versus 3.5–4 mm long and 0.9–1.2 mm wide in *S.cochabambense*) but this character can be difficult to see in the absence of comparative material. The berries of *S.cochabambense* are larger (1–1.2 cm in diameter) than those of *S.aloysiifolium* (0.5–0.6 cm in diameter), with similar numbers of stone cells.

Leaf margins in *S.aloysiifolium* are usually entire, but very occasionally some plants (e.g., *Nee 31497*) have leaves with irregularly toothed margins, especially towards the base. Leaves of *S.aloysiifolium* are usually narrower than those of *S.cochabambense* where they grow in sympatry, but this is not a consistently reliable character.

*Solanumaloysiifolium* could also be confused with the widespread and weedy *S.chenopodioides*; both taxa have matte berries and deeply stellate corollas. They differ in inflorescence morphology (forked in *S.aloysiifolium*, unbranched in *S.chenopodioides*) and stone cell number (ca. 10 or more in *S.aloysiifolium*, absent in *S.chenopodioides*). The distinctive down-turned fruiting peduncle of *S.chenopodioides* is never found in *S.aloysiifolium*.

#### ﻿4. Solanum americanum

Taxon classificationPlantaeSolanalesSolanaceae

Mill., Gard. Dict. ed. 8, no. 5. 1768.

**Figure 15.**
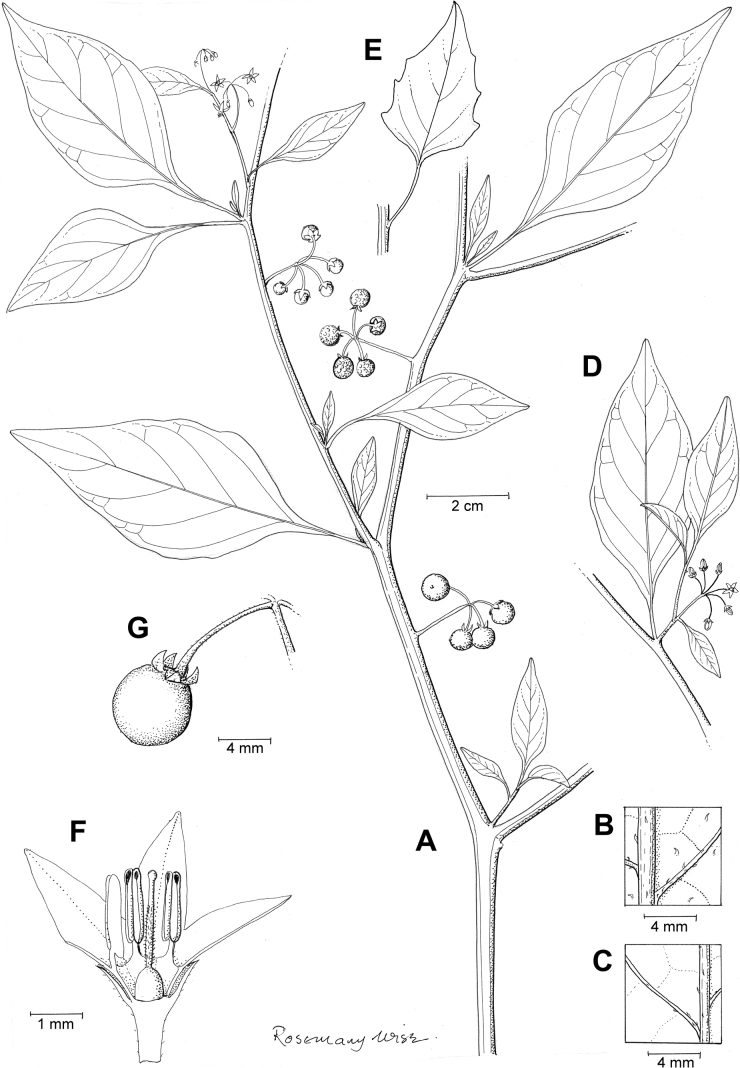
*Solanumamericanum***A** habit **B** detail of abaxial leaf surface **C** detail of adaxial leaf surface **D** branch with inflorescence **E** leaf **F** dissected flower **G** fruit (**A–D, F, G***Cremers 8084***E***Farrugia et al. 2773*). Illustration by R. Wise. Previously published in Särkinen et al. (2018: 57) and Knapp et al. (2019: 38).

**Figure 16.**
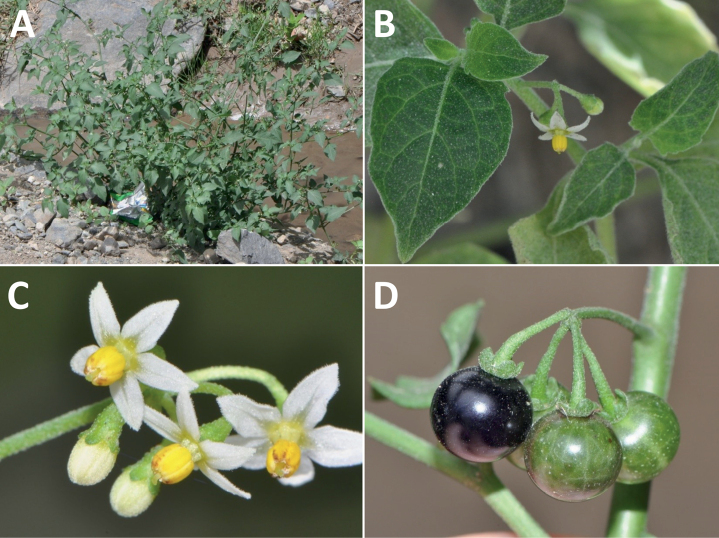
*Solanumamericanum***A** habit **B** leaves and young inflorescence **C** buds and flowers **D** mature, shiny black fruits with reflexed calyx lobes (**A, D***Knapp et al. 10210***B***Knapp et al. 10205***C***Knapp et al. 10360*). Photos by S. Knapp. Previously published in Särkinen et al. (2018: 58) and Knapp et al. (2019: 39).

Figs 15 , 16 Types based on American specimens only; for full synonymy, see Särkinen et al. (2018: 51–56)

Solanum oleraceum Dunal, Encycl. [J. Lamarck & al.] Suppl. 3: 750. 1814. Type. “Antilles” *Herb, Richard s.n.* (lectotype, designated by D’Arcy 1974a, pg. 735: P [P00319557]; isolectotypes: G-DC [G00144258], MPU [n.v.]).
Solanum erythrocarpon G.Mey., Prim. Fl. Esseq. 109. 1818. Type. Suriname. Saramacca: Hamburg (Essequibo), *E.K. Rodschied 31* (lectotype, designated by Särkinen et al. 2018, pg. 52: GOET [GOET003505]).
Solanum nigrum Vell., Fl. Flumin. 85. 1829 [1825], nom. illeg., not Solanumnigrum L. (1753). Type. Brazil. [Rio de Janeiro]: “undequaeque nascitur” (lectotype, designated by Knapp et al. 2015, pg. 832: [illustration] Original parchment plate of Flora Fluminensis in the Manuscript Section of the Biblioteca Nacional, Rio de Janeiro [cat. no.: mss1198651_112] and later published in Vellozo, Fl. Flumin. Icon. 2: tab. 109. 1831).
Solanum tenuiflorum Steud., Nomencl. ed. 2, 2: 606. 1841. Type. Based on (replacement name for) Solanumnigrum Vell.
Solanum indecorum A.Rich., Hist. Fls. Cuba, Fanerogamia 11: 121. 1841. Type. Cuba. Sin loc., 1836, *R. de la Sagra s.n.* (lectotype, designated by Särkinen et al. 2018, pg. 52: P [P00370899]).
Solanum nigrum L. var. angulosum Sendtn., Fl. Bras. (Martius) 10: 16. 1846, as SolanumnigrumL.subsp.nodiflorum(Jacq.) Sendtn.var.angulosum Sendtn. Type. Based on Solanumtenuiflorum Steud. (= Solanumnigrum Vell.).
Solanum nigrum L. subsp. aguaraquiya Sendtn., Fl. Bras. (Martius) 10: 17. 1846. Type. Brazil. Rio Grande do Sul: “Pat. Joan a St. Barbara”, *C.F.P. Martius s.n.* (lectotype, designated by Särkinen et al. 2018, pg. 52: M [M-0171809]; isolectotype: M [M-0171810]).
Solanum nigrum L. var. minus Hook.f., Trans. Linn. Soc. London 20(2): 201. 1847, as “*minor*” Type. Ecuador. Galápagos Islands: James Island [Santiago], *C. Darwin s.n.* (lectotype, designated by Särkinen et al. 2018, pg. 52: CGE [CGE00297]; isolectotype: K [K000922162]).
Solanum amarantoides Dunal, Prodr. [A. P. de Candolle] 13(1): 55. 1852. Type. Brazil. Rio de Janeiro, *C. Gaudichaud 522* (lectotype, designated by D’Arcy 1974a, pg. 735 [as holotype]; second step designated by Särkinen et al. 2018, pg. 52: P [P00319574]; isolectotypes: P [P00319575], MPU [n.v.]).
Solanum pterocaulum Dunal var. aguaraquiya (Sendtn.) Dunal, Prodr. [A. P. de Candolle] 13(1): 52. 1852, as ‘*pterocaulon>*’. Type. Based on Solanumnigrum L. subsp. aguaraquiya Sendtn.
Solanum ptychanthum Dunal, Prodr. [A. P. de Candolle] 13(1): 54. 1852. Type. United States of America. Georgia: Chatham Co., Savannah, *Anon. s.n.* (holotype: G-DC [G00144485]).
Solanum nodiflorum Jacq. var. macrophyllum Dunal, Prodr. [A. P. de Candolle] 13(1): 46. 1852. Type. Brazil. Rio de Janeiro: Rio de Janeiro, *C. Gaudichaud 521* (lectotype, designated by D’Arcy 1974a, pg. 735: P [P00319582]; isolectotypes: P [P00319583, P00319585], G-DC [G00144100], G [G00343373]).
Solanum nodiflorum Jacq. var. acuminatum Dunal, Prodr. [A. P. de Candolle] 13(1): 46. 1852. Type. Brazil. Minas Gerais: Sin loc., *M. Vauthier 537* (lectotype, designated by D’Arcy 1974a, pg. 735 [as type ex Herb. Drake]: P [P00319615]; isolectotypes: P [P00319614], G-DC [G00343360]).
Solanum nodiflorum Jacq. var. petiolastrum Dunal, Prodr. [A. P. de Candolle] 13(1): 46. 1852. Type. Brazil. Rio de Janeiro: Novo Friburgo, 1842, *P. Claussen 180* (holotype: P [P00319584]).
Solanum inops Dunal, Prodr. [A. P. de Candolle] 13(1): 55. 1852. Type. Mexico. “sin. loc.” [Tamaulipas: Tampico, 4 Feb 1827], *J.L. Berlandier 46* (holotype: G-DC [G00144469]; isotypes: BM [BM000775579], F [F0073104F], LE, P [P00336046, P00336047, P00336048], W [acc. # 1889-0291394, acc. # 1889-0144848]).
Solanum nigrum L. var. oleraceum (Dunal) Hitchc., Rep. Missouri Bot. Gard 4: 111. 1893. Type. Based on Solanumoleraceum Dunal.
Solanum nigrum L. var. americanum (Mill.) O.E.Schulz, Symb. Antill. (Urban) 6: 160. 1909. Type. Based on Solanumamericanum Mill.
Solanum nigrum L. forma grandifolium O.E.Schulz, Symb. Antill. (Urban) 6: 160. 1909, as SolanumnigrumL.var.americanum(Mill.) O.E.Schulzformagrandiifolia O.E.Schulz. Type. Puerto Rico. “Prope Cayey in sylvis ad rivulum superiorem m. Sept. fl. et. fr.”, *P.E.E. Sintenis 2429* (no herbarium cited; no duplicates found).
Solanum nigrum L. forma parvifolium O.E.Schulz, Symb. Antill. (Urban) 6: 160. 1909, as SolanumnigrumL.var.americanum(Mill.) O.E.Schulzformaparvifolia O.E.Schulz. Type. Cuba. La Habana: Santiago de las Vegas “Baker Herb. Cub. 3377” (no herbarium cited; no duplicates found).
Solanum minutibaccatum Bitter, Repert. Spec. Nov. Regni Veg. 10: 549. 1912. Type. Bolivia. La Paz: “San Carlos, bei Mapiri”, 750 m, Aug 1907, *O. Buchtien 1443* (lectotype, designated by Särkinen et al. 2018, pg. 54: US [00027684, acc. # 1175843]; isotypes: GOET [GOET003478], NY [00172089]).
Solanum inconspicuum Bitter, Repert. Spec. Nov. Regni Veg. 11: 204. 1912. Type. Peru. Lima: Lima, 12 Jul 1910, *C. Seler 222* (holotype: B, destroyed; no duplicates found).
Solanum tenellum Bitter, Repert. Spec. Nov. Regni Veg. 11: 219. 1912. Type. Brasil. Minas Gerais: “Prope urbem Caldas florens fructibusque instructum”, 4 Oct 1869, *A.F. Regnell III 970* (holotype: UPS; isotype: US [00027821, acc. # 201069]).
Solanum minutibaccatum Bitter subsp. curtipedunculatum Bitter, Repert. Spec. Nov. Regni Veg. 11: 205. 1912. Type. Bolivia. La Paz: Guanai-Tipuani, Apr-Jun 1892, *M. Bang 1462* (holotype: W [acc. # 1893-0005615]; isotypes: BM [BM000617672], E [E00106087], M [M-0171808], MO [MO-503647, acc. # 1713464], NDG [NDG42278], NY [00172090, 00172091, 00172092], PH [00030453], US [00027685, acc. # 1324656; 02835359], WIS [0256198WIS]).
Solanum sciaphilum Bitter, Repert. Spec. Nov. Regni Veg. 11: 220. 1912. Type. Brazil. Santa Catarina: Pedras Grandes, Aug 1890, *E. Ule 1678* (holotype: B, destroyed [F neg. 2851]; lectotype, designated by Särkinen et al. 2018, pg. 54: HBG [HBG-511539]; isolectotype: HBG [HBG-511540]).
Solanum curtipes Bitter, Repert. Spec. Nov. Regni Veg. 11: 228. 1912. Type. Paraguay. Cordillera: San Bernardino, Aug 1898–1899, *É. Hassler 3104* (holotype: B, destroyed; lectotype, designated by Morton 1976, pg. 149: G [G00306710]; isolectotypes: G [G00306711, G00306712, G00306713, G00306714], K [K000532497], P [P00325762], NY [00139112], UC [UC950837]).
Solanum calvum Bitter, Repert. Spec. Nov. Regni Veg. 12: 81. 1913. Type. Mexico. Baja California: Guadalupe Island, 1875, *E. Palmer 60* [pro parte] (holotype: UPS; isotypes: BM [BM001017192], MO [MO-159620, acc. # 5257812; MO-568722, acc. # 1713454], NY [00138967, 00759880], YU [YU065319]).
Solanum nodiflorum Jacq. var. sapucayense Chodat, Bull. Soc. Bot. Genève, sér. 2, 8: 150. 1916. Type. Paraguay. Paraguarí: Sapucaí [“Sapucay”], 1914, *R. Chodat & W. Vischer 46* (holotype: G [G00306708]).

##### Type.

Cultivated at the Chelsea Physic Garden [in protologue said to “grow naturally in Virginia”], *Herb. Miller s.n.* (lectotype, designated by Edmonds 1972, pg. 103 [as type]: BM [BM000617683]).

##### Description.

Annual to short-lived perennial herbs up to 1.5 m high, subwoody at base. Stems terete or somewhat angled with ridges, older stems sometimes with spinose processes, not markedly hollow; new growth pubescent with simple, spreading, uniseriate 2–8-celled eglandular trichomes 0.2–0.8 mm long, often clustered along the stem angles; older stems glabrescent. Sympodial units difoliate, the leaves not geminate. Leaves simple, the blades 3.5–10.5 cm long, 1–4.5 cm wide, ovate to elliptic, widest at the middle or in the lower third, membranous, concolorous or slightly discolorous; adaxial surface sparsely pubescent with simple, uniseriate trichomes like those on stem, these evenly spread along the lamina and the veins; abaxial surface similar but more densely pubescent; major veins 3–6 pairs; base attenuate, decurrent on the petiole; margins entire or occasionally sinuate-dentate; apex acute; petioles (0.3-)2–3.8(-4) cm long, sparsely pubescent with simple uniseriate trichomes like those on stems. Inflorescences internodal, unbranched or extremely rarely forked, 0.6–2.5 cm long, with (3-)4–6(8) flowers (outside of South America very rarely with many flowers in unusual many-branched inflorescences) clustered near the tips (umbelliform to sub-umbelliform), sparsely pubescent with simple uniseriate trichomes like those on stems; peduncle (0.5-)1–1.8 cm long, delicate; pedicels 3–9 mm long, 0.2–0.3 mm in diameter at the base and 0.4–0.5 mm at the apex, stout, straight and spreading, articulated at the base; pedicel scars spaced 0–0.5 mm apart, clustered at the tip of the inflorescence. Buds broadly ellipsoid, the corolla exserted 1/3 beyond the calyx lobe tips before anthesis. Flowers 5-merous, cosexual (hermaphroditic). Calyx tube 0.8–1.3 mm long, the lobes 0.3–0.5 mm long, 0.5–0.6 mm wide, broadly triangular with obtuse apices, sparsely pubescent with simple uniseriate trichomes like those of the stem. Corolla 0.3–0.6 cm in diameter, stellate, white with a yellow-green central portion near the base, lobed halfway to 2/3 of the way to the base, the lobes 2–3.2 mm long, 1–2.5 mm wide, strongly reflexed at anthesis, later spreading, densely papillate abaxially with 1–4-celled simple uniseriate trichomes, these denser on the tips and margins. Stamens equal; filament tube minute; free portion of the filaments 0.5–0.8 mm long, adaxially pubescent with tangled uniseriate trichomes; anthers 0.7–1.5 mm long, 0.5–0.6 mm wide, ellipsoid to almost globose and very plump-looking, yellow, poricidal at the tips, the pores lengthening to slits with age and drying. Ovary globose, glabrous; style 2.2–2.6 mm long, straight, almost included to exserted beyond the anther cone, densely pubescent with 2–3-celled simple uniseriate trichomes 2/3 from the base where included in the anther cone; stigma minutely capitate, the surface minutely papillate, green in live plants. Fruit a globose berry, 0.4–0.9(-1.2) cm in diameter, purplish-black at maturity, the surface of the pericarp markedly shiny, opaque, glabrous; fruiting pedicels 1.3–1.8 cm long, ca. 0.7–1 mm in diameter at the base, 0.8–1 mm in diameter at the apex, stout, straight and spreading, spaced ca. 1(-3) mm apart or tightly clustered, persistent, remaining on the plant and persistent on older inflorescences; fruiting calyx lobes not accrescent, the tube less than 1 mm long, the lobes 1(-2) mm long, strongly reflexed at fruit maturity. Seeds 30–50 per berry, 1–1.5 mm long, 0.8–1.3 mm wide, flattened and teardrop shaped with a subapical hilum, pale yellow, the surfaces minutely pitted, the testal cells pentagonal in outline. Stone cells mostly absent (Australia, South Pacific, and South America), but if present (North America, Mexico, Caribbean, Eurasia and Africa) 2–4(6) per berry, 2–4 larger ones > 0.5 mm, and two smaller ones < 0.5 mm in diameter. Chromosome number: n = 12 (see Särkinen et al. 2018 for vouchers).

##### Distribution

**Figure 17.**
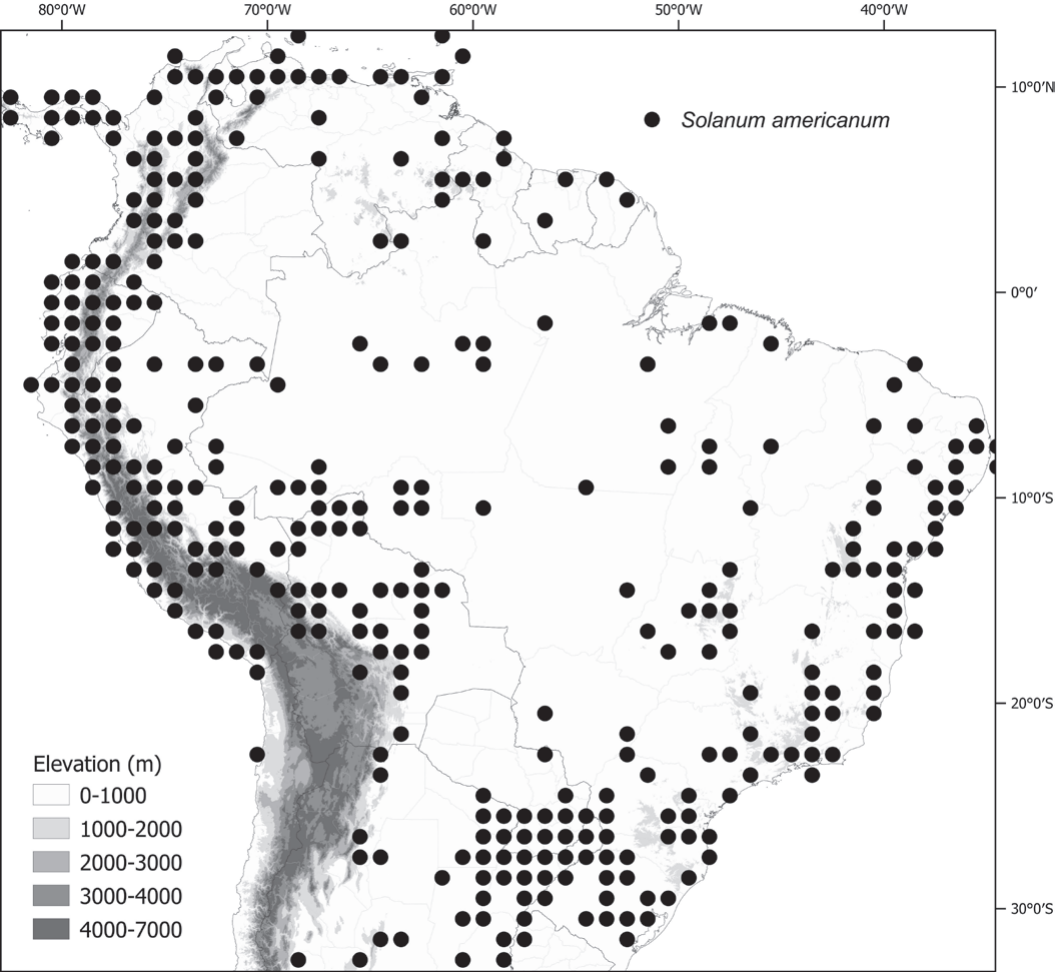
Distribution map of *Solanumamericanum* in South America. For distribution elsewhere, see Särkinen et al. (2018: 60) and Knapp et al. (2019: 43).

**(Fig. 17).***Solanumamericanum* is a globally distributed weed found throughout the tropics and subtropics; it is not clear where it is native, or if this circumtropical distribution is its native range. In South America it occurs in every country and as far south as 37°S latitude.

##### Ecology and habitat.

*Solanumamericanum* is a weedy species that colonises disturbed soil and it is found in open areas, along roads, treefall gaps and at the back of beaches from sea level to 2,000 m elevation.

##### Common names and uses.

Argentina. Misiones: ka’a ete’I (Mbyá Guaraní, Keller 2007). Paraguay. Arachichu (Ibarrola and Degen 2011). Brazil. araxixu, caraxixá, erva-de-bicho, erva-mocó, erva-moura, guaraquinha, maria-preta, maria-pretinha, pimenta de cachorro, pimento de rato, pimenta de rato (Lorenzi and Abreu Matos 2002, could also refer to *S.paucidens*); caraxixá, erva moura, guaraquinha, maria-pretinha, pimento de galinha (Ferreira Kinupp and Lorenzi 2021); Pernambuco: erva moura (Rodrigues and Andrade 2014).

In Argentina, fruits are used as compresses and poultices to treat boils (Kujawska and Hilgert 2014), and in both Paraguay and Argentina the fruits and leaves have been reported as used medicinally (Keller 2007; Ibarrola and Degen 2011). In rural communities of Pernambuco, Brazil, macerated leaves are used to treat fungal infections, gastritis, varicose veins and bruises (Rodrigues and Andrade 2014). Uses reported in Lorenzi and Abreu Matos (2002) are discussed in the section on uses.

Across its range in South America *S.americanum* is often known by the common name yerba (hierba) mora (Spanish) or erva-moura (Portuguese). In Mexico (see Knapp et al. 2019) and outside the Americas (see Särkinen et al. 2018) the leaves are eaten as cooked greens (potherbs) but we have seen no records of these uses of *S.americanum* from South America.

##### Preliminary conservation status

**(IUCN 2022).** Least Concern [LC]. EOO = 89,639,763 km^2^ [LC]; AOO = 9,828km^2^ [LC]. *Solanumamericanum* is a cosmopolitan weed of the tropics and subtropics (see Särkinen et al. 2018; Knapp et al. 2019).

##### Discussion.

*Solanumamericanum* is the most widespread and common species of the morelloid solanums (see Särkinen et al. 2018), and quite possibly the most widely distributed species in *Solanum*. It has been implicated as the diploid parent in the polyploid events that gave rise to the species occurring outside of the Americas (e.g., Edmonds 1977; Poczai and Hyvönen 2011), although this has been disputed (Ma 1995)

*Solanumamericanum* can be easily recognised in fruit by its shiny black berries with small, strongly reflexed calyx lobes that are held on erect or spreading pedicels. In flower, the species has tiny almost globose anthers 0.8–1.5 mm long and short filaments usually less than 1 mm long. Ripe berries of *S.americanum* are shiny black (but that can be difficult to see in herbarium specimens) and in South America lack stone cells; in North and Central America and the Caribbean berries usually have four stone cells in each. When berries ripen in *S.americanum* they fall from the plant leaving the stout, spreading pedicels with reflexed calyces behind.

*Solanumnigrescens* differs from *S.americanum* in having larger anthers always more than 2 mm long, matte black or green fruits that are held on spreading or deflexed pedicels that drop with the berry, and calyx lobes appressed to the berry in fruit. Berries of *S.nigrescens* have more than 5 (usually 5–6 large and several smaller) stone cells, while plants of *S.americanum* from South America usually lack stone cells. Inflorescences of *S.americanum* tend to be more sub-umbelliform in appearance than those of *S.nigrescens*, and calyx lobes of *S.americanum* are strongly reflexed and smaller relative to berry size in fruit.

Manoko et al. (2007) distinguished *S.americanum* and *S.nodiflorum* using AFLP markers; we re-examined the material they used and consider the plants they called *S.nodiflorum* to be *S.americanum* as defined here, and plants they called *S.americanum* represent specimens of *S.nigrescens* (see Särkinen et al. 2018: 61).

Populations from Río Pastaza, Río Morone, and Río Nanay watersheds in Amazonian Ecuador and Peru have anthers ca. 2 mm long and somewhat more elongate inflorescences than in the rest of the species range. The plants fit well within the circumscription of *S.americanum* however, with shiny black fruits with reflexed calyx lobes. Variation in pedicel spacing is observed in other parts of the species range, but the larger anther size is unique to these populations in lowland Ecuador and Peru.

Typification details for the many synonyms of *S.americanum* can be found in Särkinen et al. (2018).

#### ﻿5. Solanum annuum

Taxon classificationPlantaeSolanalesSolanaceae

C.V.Morton, Revis. Argentine Sp. Solanum 102. 1976.

Figs 3B , 18 , 19

Solanum micrantherum Cabrera, Hickenia 1(31): 168. 1978. Type. Argentina. Catamarca: Andalgalá, El Candado, *P. Jörgensen 978* (holotype: SI [003327]; isotypes: CORD [CORD00006989, fragment], GH, MO [MO-2127099, acc. # 818835], US [028337125, acc. # 921698]).

##### Type.

Argentina. Salta: Dpto. Rosario de Lerma, Campo Quijano, 17 Jan 1929, *S. Venturi 8507* (holotype: US [00027454, acc. # 1549043]).

**Figure 18.**
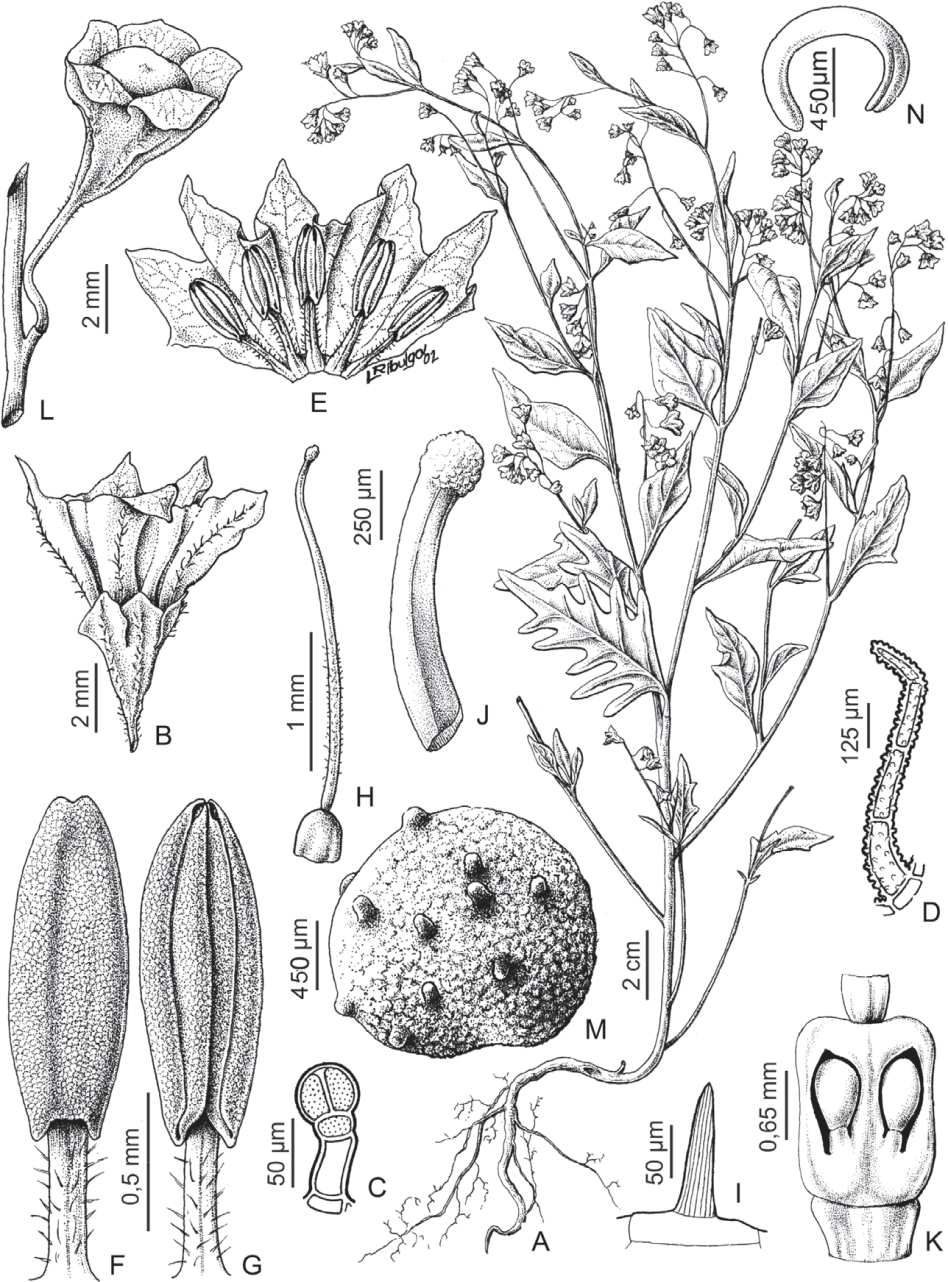
*Solanumannuum***A** plant **B** flower **C** glandular trichome of the calyx **D** eglandular trichome of the calyx **E** dissected flower **F** stamen, dorsal view **G** stamen, ventral view **H** gynoecium **I** eglandular trichome of the style **J** apex of the style and stigma **K** ovary, longitudinal section **L** fruit **M** seed **N** embryo (**A–N***Hunziker et al. 24901*). Illustration by L. Ribulgo. Previously published in Barboza et al. (2013: 238).

##### Description.

Tiny annual herbs 0.05–0.5 m high, erect or, if larger, the plants spreading. Stems terete, often drying purple, moderately pubescent with eglandular, white simple uniseriate trichomes ca. 0.5 mm long, these antrorse; new growth densely pubescent with eglandular white simple uniseriate trichomes like those of the stems; older stems greenish brown. Sympodial units difoliate or trifoliate, the leaves not geminate. Leaves simple to deeply pinnatifid, both types present on single stems, the blades (1)1.5–5 cm long, (0.5)0.6–2.5 cm wide, ovate to ovate-elliptic in outline, widest in the lower third, membranous, concolorous; adaxial surfaces sparsely pubescent with eglandular white simple uniseriate trichomes to 0.5 mm long like those of the stems; abaxial surfaces similarly sparsely pubescent but the trichomes denser along the veins; principal veins 3–4 pairs, corresponding to numbers of lobes in pinnatifid leaves; base acute to somewhat attenuate along the petiole; margins entire to deeply pinnatifid, entire leaves often at base of plant, the lobes long-triangular with rounded tips, the sinuses reaching nearly to the midrib in the most deeply pinnatifid leaves; petiole 0.2–1.5 cm long, sparsely pubescent with eglandular simple uniseriate trichomes like those of the stems. Inflorescences opposite the leaves, unbranched or rarely forked, 1.5–5 cm long, with 5–12 flowers, sparsely pubescent with eglandular simple white uniseriate trichomes ca. 0.5 mm long; peduncle 0.6–5 cm long; pedicels 0.7–0.9 cm long, ca. 0.5 mm in diameter at the base, ca. 0.5 mm in diameter at the apex, filiform and spreading, sparsely pubescent with simple uniseriate trichomes like the rest of the inflorescence, articulated near the base, leaving a small raised stump on the inflorescence axis; pedicel scars irregularly spaced 1.5–5 mm apart. Buds globose, the corolla halfway exserted from the corolla tube before anthesis. Flowers 5-merous, cosexual (hermaphroditic). Calyx tube 0.75–1 mm long, conical, the lobes 1–2 mm long, ca. 1 mm wide, deltate or very rarely triangular, sometimes somewhat unequal in size, the apices rounded or rarely acute, sparsely pubescent with eglandular simple uniseriate trichomes ca. 0.5 mm long like those of the pedicel. Corolla 0.7–1.5 cm in diameter, white to pale lavender, pentagonal to very shallowly stellate, lobed ca. 1/4 of the way to the base, the lobes 1.5–2.5 mm long, 3–4 mm wide, spreading at anthesis, glabrous on both surfaces but with a few unicellular papillae on the lobe tips. Stamens equal; filament tube ca. 0.5 mm long; free portion of the filaments 1–1.5 mm long, densely pubescent with tangled eglandular simple uniseriate trichomes abaxially, the trichomes with verrucose surfaces; anthers 2.5–4 mm long, 0.75–1 mm wide, ellipsoid, yellow, poricidal at the tips, the pores lengthening to slits with age. Ovary conical, glabrous; style (3.5)5.5–7 mm long, straight, often included in the anther cone, densely pubescent in the lower 2/3 to 1/2 ; stigma capitate, the surface minutely papillate. Fruit a globose berry, 0.25–0.3 cm in diameter, green when mature, the pericarp thin, matte, opaque, glabrous; fruiting pedicels 0.25–0.8 cm long, ca. 0.5 mm in diameter at the base, not markedly woody, pendent or deflexed, not persistent; fruiting calyx accrescent but not covering the berry, instead spreading as a subtending open cup, the tube to 4 mm long, the lobes 3–4 mm long, 3–4 mm wide, rounded. Seeds 1–2 per berry, 2.5–2.6 mm long, 2.1–2.5 mm wide, rounded and only slightly flattened, dark brown, the surfaces minutely pitted and tuberculate, the testal cells rectangular in outline. Stone cells absent. Chromosome number: n = 12 (Moscone 1992; voucher *Hunziker 24901*, as *S.nicandricalyx*).

**Figure 19.**
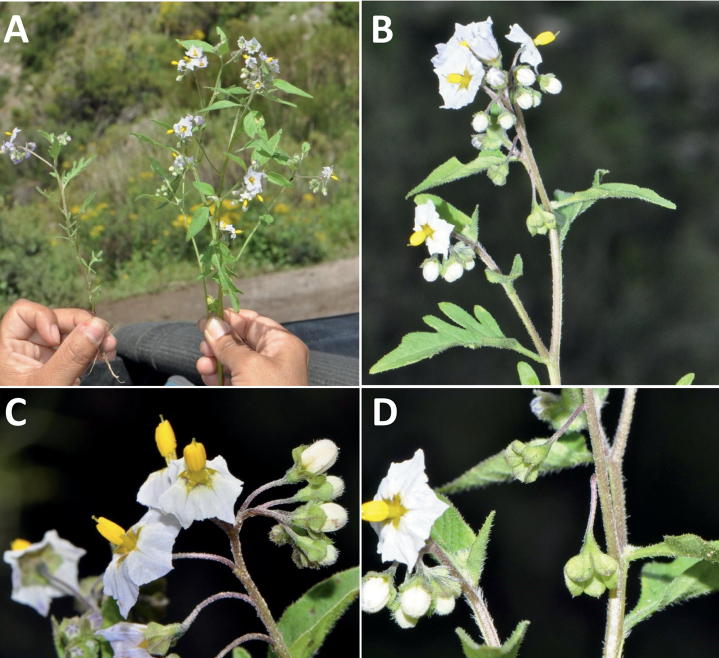
*Solanumannuum***A** habit **B** flowering stem with dissected leaves **C** flowering stem with entire leaves **D** developing fruits surrounded by accrescent calyx (**A–D***Barboza et al. 3495*). Photos by S. Knapp.

##### Distribution

**Figure 20.**
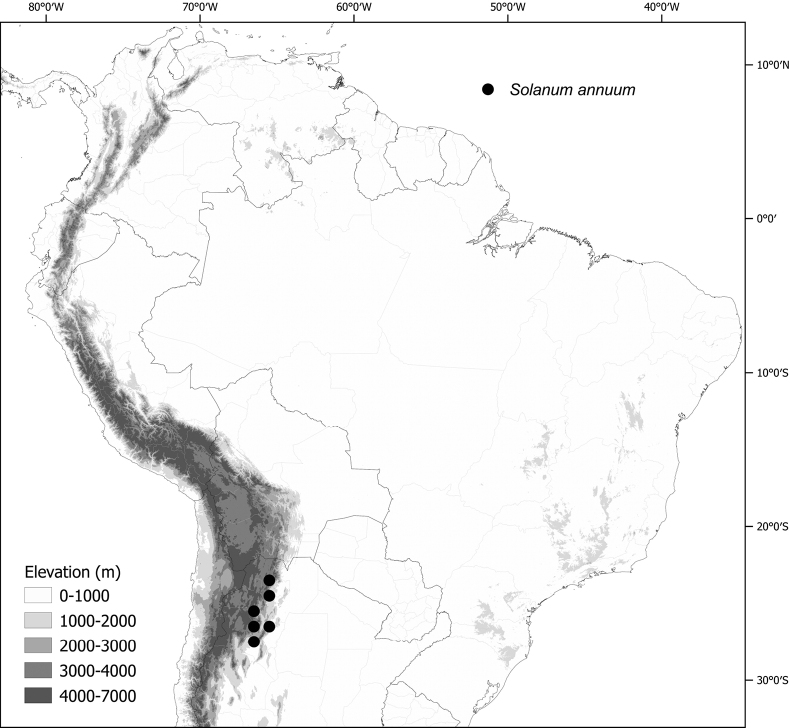
Distribution map of *Solanumannuum*.

**(Fig. 20).***Solanumannuum* is endemic to northern Argentina (Prov. Jujuy, Salta, Tucumán, and Catamarca).

##### Ecology and habitat.

*Solanumannuum* is found in prepuna and puna habitats, often in open and disturbed areas; from 2,100 to 3,300 m elevation.

##### Common names and uses.

None recorded.

##### Preliminary conservation status

**(IUCN 2022).** Vulnerable [VU – B2 a,b(iii, iv), D2]). EOO = 25,287 km^2^ [LC]; AOO = 72 km^2^ [EN]. The large EOO of *S.annuum* suggests it is not of particular conservation concern, and the smaller AOO is perhaps the result of collecting deficit. We suggest this species warrants some concern as it occurs in fewer than five sites and populations are small and widely dispersed; most collections have been made in the area of Tafí del Valle.

##### Discussion.

*Solanumannuum* is a distinctive species; it is small annual herb with leaves that are extremely variable in shape even on the same plant (Fig. 19A). The inflorescence is long and filiform, and the calyx is a spreading cup, somewhat like those of *S.weddellii* and *S.gilioides*. Unlike those taxa, however, the calyx does not become accrescent and fully envelop the fruit but remains an expanded plate-like structure subtending the tiny berry (Fig. 19D). Morton (1976) thought *S.annuum* was related to *S.salicifolium* (as *S.incisum*) by virtue of its pedunculate, multiflowered inflorescence and often incised leaf shape and placed it in his sect. Dulcamara p.p.; Cabrera (1978) suggested it was probably a member of sect. Campanulisolanum and related to *S.sinuatiexcisum* and *S.fiebrigii*, based on its rotate/campanulate corolla. Habit (small annual herbs vs. shrub or perennial herbs), corolla shape (pentagonal-rotate versus deeply stellate) and anther size (1.2–2.7 mm long vs 3.5–5 mm long) easily distinguish *S.annuum* from *S.salicifolium*, while the number and ornamentation of the seeds (two tuberculate seeds vs. many minutely pitted seeds) easily distinguish it from *S.sinuatiexcisum* and *S.fiebrigii*. Barboza (2003) treated *S.annuum* as a member of section Chamaesarachidium, along with *S.gilioides* and *S.weddellii* (as *S.chamaesarachidium*) with which it shares the herbaceous habit, accrescent calyx in fruit and tuberculate seeds. The calyx is less accrescent in *S.annuum* than in the other two taxa included by Barboza (2003) and the single-seeded locules of *S.annuum* are unique in morelloids. In molecular phylogenetic analyses (Särkinen et al. 2015b; Gagnon et al. 2022) *S.annuum* is a member the Black nightshade clade but is not sister to *S.gilioides* and *S.weddellii*, the two other morelloid species with tuberculate seeds. Within the Black nightshade clade it is in an unresolved polytomy involving *S.furcatum* and the rest of the clade (see fig. 2 of Särkinen et al. 2015b), suggesting that the tuberculate seeds may be homoplasious.

#### ﻿6. Solanum antisuyo

Taxon classificationPlantaeSolanalesSolanaceae

Särkinen & S.Knapp, PhytoKeys 44: 47. 2015.

**Figure 21.**
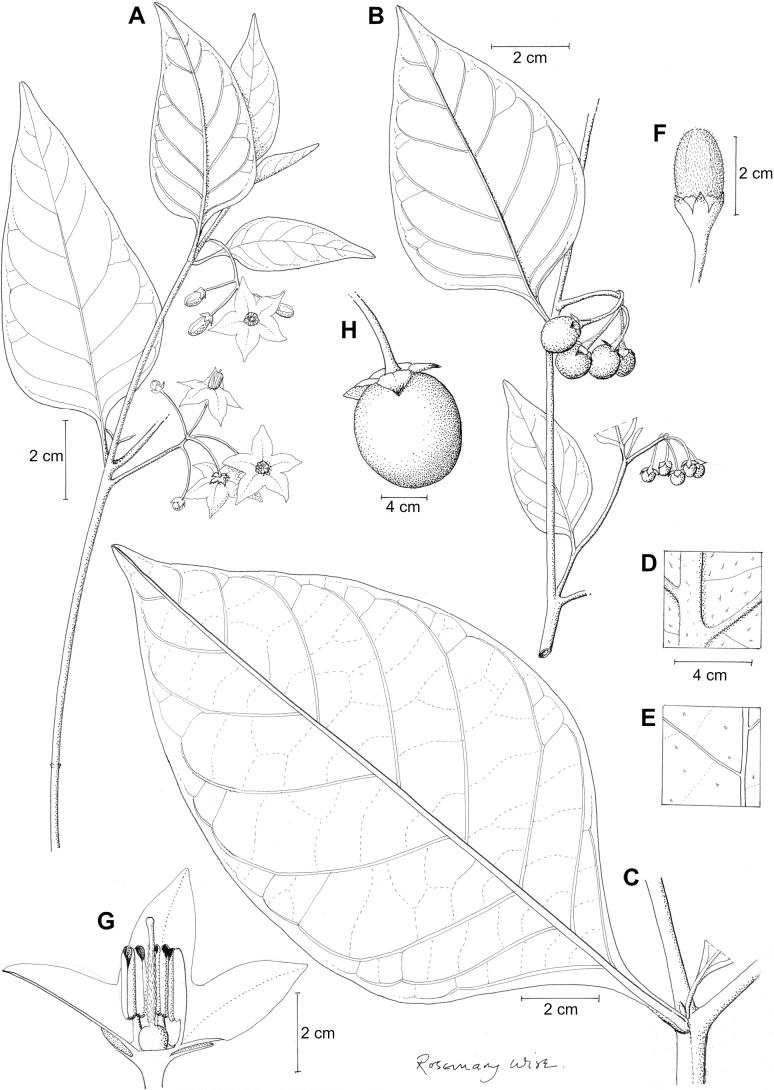
*Solanumantisuyo***A** flowering branch **B** fruiting branch **C** stem with a leaf node **D** detail of abaxial leaf surface **E** detail of adaxial leaf surface **F** flower bud **G** dissected flower **H** fruit (**A–H***Knapp et al. 10435*). Illustration by R. Wise.

**Figure 22.**
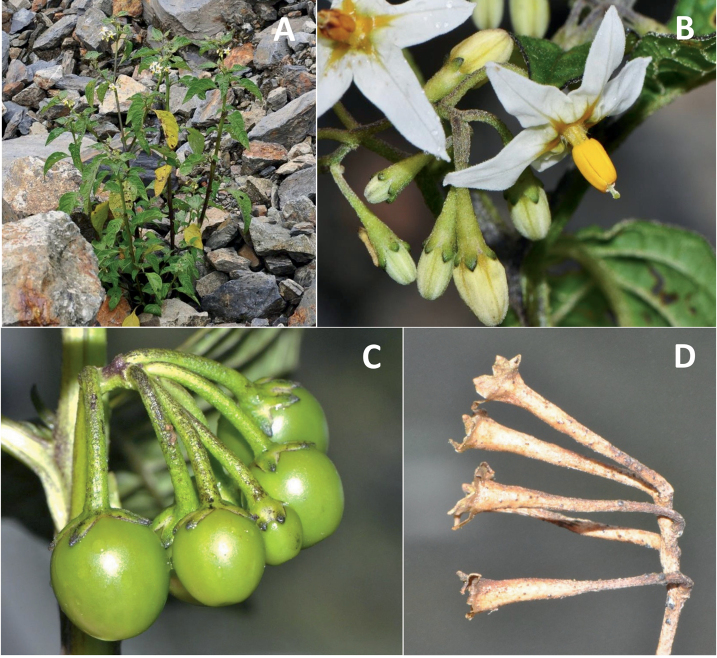
*Solanumantisuyo***A** habit in rocky landslide **B** buds and flowers, showing the distinct calyx with long tube and minute, but somewhat fleshy, purplish green lobes **C** slightly ellipsoid fruits with deflexed pedicels, with appressed calyx lobes often splitting in fruit **D** woody pedicels of the infructescence (**A, B***Knapp et al. 10399***C***Knapp et al. 10401***D***Knapp et al. 10435*). Photos S. Knapp. Previously published in Särkinen et al. (2015c: 48).

Figs 4D, E , 21 , 22

##### Type.

Peru. Cusco: Prov. Paucartambo, 1 km from Puesto de Vigilancia of Parque Nacional de Manu on road from Paucartambo to Pilcopata coming from Puesto, 13°12'05"S, 71°37'21"W, 3,480 m, 15 Mar 2012, S. *Knapp, P. Gonzáles, A. Matthews & T. Särkinen 10435* (holotype: USM (acc. # 00268057); isotypes: BM [BM001114929], F, HUSA, HUT, MO).

##### Description.

Stout herbs or subwoody shrubs up to 1.5 m high, much branching at base, the individual branches up to 1 m long. Stems 2-ridged or slightly winged especially towards base, 0.4–0.6 cm in diameter, purple-coloured especially at leaf nodes, nearly glabrous, sparsely pubescent with simple uniseriate, much reduced 1–3-celled trichomes especially on the often purple-coloured young growth. Sympodial units difoliate, not geminate. Leaves simple, the blades 2–17 cm long, 1.2–8.4 cm wide, broadly ovate-lanceolate, widest in the lower third, membranous to somewhat fleshy, slightly discolorous; adaxial and abaxial surfaces sparsely pubescent with more or less appressed 1–3-celled simple uniseriate trichomes 0.1–0.2 mm long; principal veins 7–10 pairs; base rounded, decurrent on the petiole; margins entire, often purple tinged; apex acute to acuminate; petiole 0.3–1.2 cm long, occasionally narrowly winged, sparsely pubescent with simple uniseriate trichomes like those of the stems and leaves. Inflorescences internodal, unbranched or forked, 1.4–4 cm long, with 5–14 flowers arising very close together, sparsely pubescent with appressed 1–2-celled simple uniseriate trichomes similar to those on stem and leaves; peduncle 1–3.3 cm long, if the inflorescence branched then the peduncle 0.2–0.4 cm long, short and congested; pedicels 1–1.2 cm long, 0.5–0.6 mm in diameter at the base expanding gradually to 1–1.2 mm in diameter at apex, straight and spreading at anthesis, recurving and becoming woody in fruit, not dehiscing; pedicel scars spaced 0–2 mm apart. Buds conical-ellipsoid, cream-coloured, the corolla strongly exserted from the calyx tube before anthesis. Flowers 5-merous, cosexual (hermaphroditic). Calyx tube 1.5–2 mm long, green, the lobes 0.7–0.9 mm long, broadly deltate with rounded apices, purple-coloured, sparsely pubescent with 1-celled simple uniseriate trichomes. Corolla 1.2–2.4 cm in diameter, stellate, white or rarely lilac with a yellow to yellow-green central star at the base, lobed slightly less than halfway to the base, the lobes ca. 9–15 mm long, 4–5 mm wide, spreading to reflexed at anthesis, pubescent abaxially with 1–3-celled simple uniseriate trichomes shorter than the trichomes of the stems and leaves, sparsely pubescent adaxially at base near the filaments with 5–7-celled simple uniseriate trichomes. Stamens equal or slightly unequal; filament tube ca. 2 mm long, adaxially pubescent with 5–7-celled simple uniseriate trichomes; free portion of the filaments ca. 2 mm long, sometimes slightly longer in two lowermost anthers at anthesis (perhaps elongating late in anthesis), pubescent like the tube; anthers ca. (2.8)3–3.4 mm long, 1 mm wide, ellipsoid, yellow, poricidal at the tips, the pores lengthening to slits with age. Ovary cylindrical, pubescent 2/3 from the base with 2–3-celled simple uniseriate trichomes; style ca. 6 mm long, straight, exserted beyond the anther cone, densely pubescent up to 2/3 of the length with 2–3-celled simple uniseriate trichomes at the base; stigma globose, minutely papillate, pale yellow in live plants. Fruit an ellipsoid berry, 0.8–1.1 cm in diameter, green turning translucent yellowish green to deep purple when ripe, the pericarp relatively thick, shiny, somewhat translucent, glabrous; fruiting pedicels 1.1–2.2 cm long, ca. 1 mm in diameter at the base and 1.5 mm at apex, deflexed and woody in fruit, purple-coloured, persistent and remaining on the plant after fruit drops; fruiting calyx lobes tightly appressed to the berry, purple-coloured, calyx often splitting into two larger lobes. Seeds 35–45 per berry, ca. 1.1 mm long, ca. 1.7 mm wide, concave-reniform, narrower at one end, brown, the hilum positioned sub-laterally towards the narrower end, the testal cells pentagonal in outline. Stone cells (0)2 per berry, usually equatorially positioned, ca. 1 mm in diameter, cream-coloured. Chromosome number: not known.

##### Distribution

**Figure 23.**
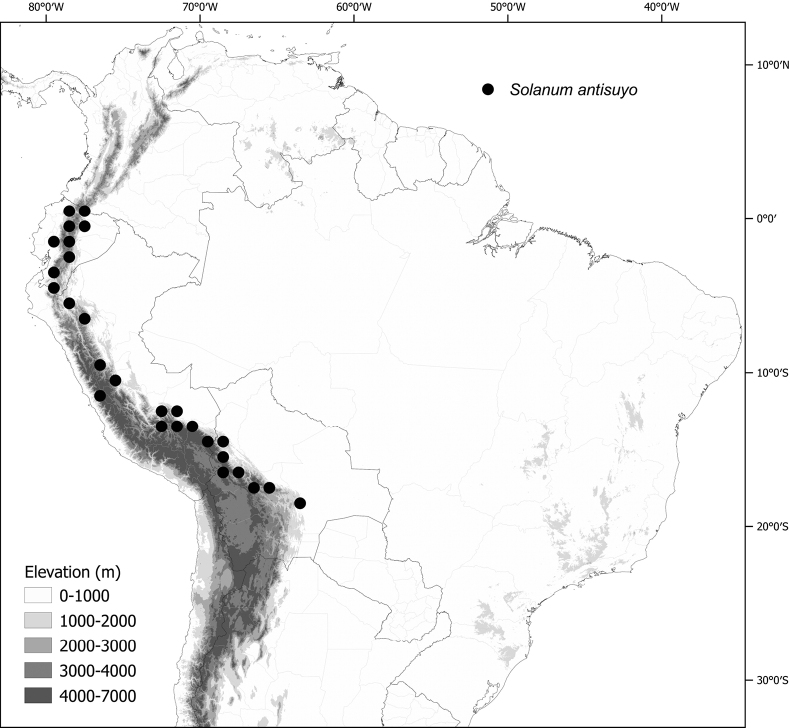
Distribution map of *Solanumantisuyo*.

**(Fig. 23).***Solanumantisuyo* occurs primarily on the eastern Andean slopes in Ecuador (Prov. Azuay, Bolívar, Chimborazo, Cotopaxi, Loja, Napo, Pichincha, Zamora-Chinchipe), Peru (Depts. Amazonas, Cusco, Huánuco, Pasco, Piura, Puno), and Bolivia (Depts. Cochabamba, La Paz).

##### Ecology and habitat.

*Solanumantisuyo* is primarily found growing in secondary vegetation, disturbed roadsides, landslides, and gravelly slopes in ‘ceja de selva’ (forest edges at treeline), montane cloud forest and *Polylepis* (Rosaceae) forests; from (1,000-) 2,000 to 3,600 (-3,900) m in elevation.

##### Common names and uses.

None recorded.

##### Preliminary conservation status

**(IUCN 2022).** Least Concern [LC]. EOO = 1,089,690 km^2^ [LC]; AOO = 400 km^2^ [LC]. *Solanumantisuyo* grows readily in disturbed sites and combined with its wide range, it appears to have relatively low threat status despite the generally increasing human pressure and habitat destruction in the Andes. It occurs within protected areas in both Peru (Parque Nacional Manu) and Bolivia (Parque Nacional Madidi).

##### Discussion.

*Solanumantisuyo* is morphologically most similar to *S.polytrichostylum* with which it has been conflated in the past. It can be distinguished by its usually simple inflorescences where pedicels are spaced ca. 1–3 mm apart along the short flowering-bearing portion of the axis compared to consistently branched inflorescences with the flowers congested at the branch tips in *S.polytrichostylum*; bud morphology also differs with the buds of *S.polytrichostylum* always somewhat elongate and usually cream with purple stripes, while those of *S.antisuyo* are more ellipsoid and usually of a single colour. The fruits of *S.antisuyo* are somewhat ellipsoid and borne on pedicels that markedly enlarge towards the apex as compared to the spherical berries on less obviously expanded pedicels of *S.polytrichostylum*. The the seeds also differ in colour (brown in *S.antisuyo* versus yellow in *S.polytrichostylum*). *Solanumantisuyo* has the calyx tube longer than the smaller, purple-tinged calyx lobes while *S.polytrichostylum* has calyx tubes shorter than the slightly larger, triangular calyx lobes; the styles of *S.polytrichostylum* are always more exserted (2–4 mm versus 1–2 mm beyond the anther cone) than those of *S.antisuyo*; fruiting pedicels of *S.antisuyo* persist after fruit drop (see Fig. 22D), while those of *S.polytrichostylum* generally do not. The two species are also ecologically somewhat distinct, with *S.polytrichostylum* restricted to streams and moist roadsides, and *S.antisuyo* is found in drier areas in gravel, disturbed areas, and landslides. Other sympatric members of the Morelloid clade without glandular trichomes with which *S.antisuyo* could be confused include *S.cochabambense* that has smaller, spherical fruits, larger violet corollas that are more rotate in outline, and denser indumentum with longer 3–7-celled simple hairs, and *S.pallidum* that has branched rather than simple hairs.

Variation in growth form and flower colour can be observed in the field, where individuals growing in more humid conditions grow into stout herbs to ca. 1.5 m high, while individuals in drier, higher elevation habitats in rocky landslides are stunted herbs reaching only ca. 40 cm in height. Colour variation in corolla is common within morelloids and *Solanum* species in general; most specimens of *S.antisuyo* have creamy white petals, but occasional specimens with lilac corollas are known (e.g., *Särkinen et al. 4048, 4049*, and *4053*).

#### ﻿7. Solanum arenicola

Taxon classificationPlantaeSolanalesSolanaceae

Särkinen & P.Gonzáles, PhytoKeys 44: 53. 2015.

Figs 24 , 25

##### Type.

Peru. Madre de Dios: Prov. Tambopata, in the boat harbor of Infierno, ca. 20 km SW by road from Puerto Maldonado, 12°44'06"S, 69°13'47"W, 186 m, 3 Aug 2014, *T. Särkinen & A. Balarezo 4866* (holotype: USM; isotypes: to be distributed to BM, E, F, GHMDD, HOXA, MO, MOL).

**Figure 24.**
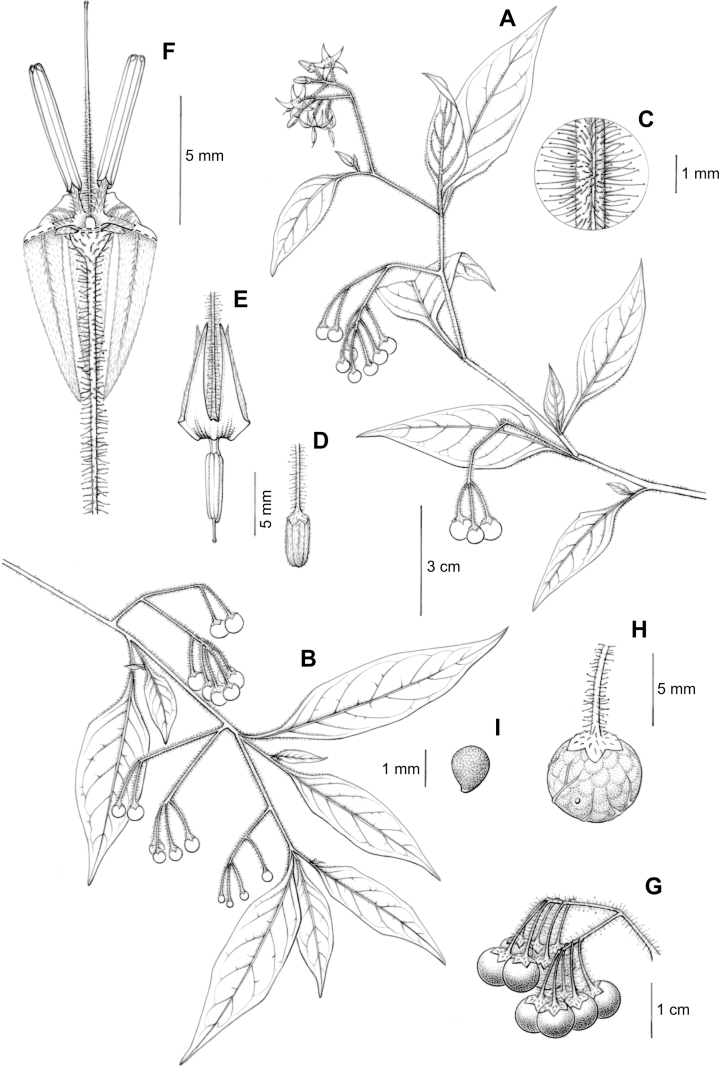
*Solanumarenicola***A** flowering and fruiting branch **B** fruiting branch **C** stem detail with glandular multi-cellular trichomes **D** flower bud **E** flower at full anthesis **F** dissected flower **G** infructescence **H** fruit **I** seed (**A–I***Parada & Rojas 2506*). Illustration by C. Banks.

##### Description.

Herb or vigorous, weak-stemmed shrub 0.2–1.5 m high. Stems slightly angled, sparsely to densely glandular-pubescent with simple, translucent, uniseriate 3–8-celled trichomes 0.8–2 mm long with glandular tips; new growth densely pubescent with spreading glandular trichomes like those of the stem. Sympodial units difoliate, not geminate. Leaves simple, the blades 2.6–13 cm long, 0.8–5 cm wide, ovate to broadly ovate, widest in the lower third, membranous, discolorous; adaxial surface glabrous; abaxial surface paler or tinged with purple, sparsely pubescent with simple uniseriate trichomes like those of the stem restricted to the veins; principal veins 5–7 pairs; base acute to cuneate and decurrent on the petiole; margins variable from entire to undulate to shallowly lobed; apex acute-acuminate; petiole 0.5–5 cm long, sparsely to densely pubescent with glandular trichomes like those of the stems. Inflorescences internodal, unbranched, 2–3.5 cm long, with 3–8(9) flowers, sparsely to densely pubescent with spreading glandular trichomes like those of the stem; peduncle 1–2.4 cm long; pedicels 0.5–0.7 cm long, ca. 0.3 mm in diameter at the base and 0.4 mm at apex, straight and spreading, articulated at the base; pedicel scars unevenly spaced 1–2.5 mm apart. Buds ellipsoid, the corolla strongly exserted from the calyx tube long before anthesis. Flowers 5–merous, cosexual (hermaphroditic). Calyx tube ca. 1 mm long, shallow, the lobes 0.2–0.5 mm long, triangular with acute apices, sparsely to densely pubescent with glandular trichomes like those of the stem. Corolla 0.8–1.2 cm in diameter, stellate, white with a purple-yellow or yellow-green central eye at the base, lobed 2/3 to the base, the lobes ca. 3.5–4 mm long, 1–1.5 mm wide, strongly reflexed at anthesis, later spreading, densely pubescent abaxially with glandular trichomes like those of the stems, glabrous adaxially. Stamens more or less equal; filament tube 1–1.2 mm long; free portion of the filaments slightly unequal in length, the lower two ca. 1.5 mm long, the upper three ca. 1–1.2 mm long, sparsely pubescent with simple uniseriate 1–3-celled trichomes on the side facing the ovary; anthers 3–4 mm long, 0.8–0.9 mm wide at base and 0.5–0.6 mm wide at apex, cylindrical, narrowing towards the apex, yellow, poricidal at the tips, the pores lengthening to slits with age. Ovary ellipsoid, glabrous; style 4–5.7 mm long, straight, long-exserted beyond the anther cone, densely pubescent up to 2/3 of the length with 1–6-celled simple uniseriate trichomes, these longer at the base and becoming gradually shorter towards the middle; stigma clavate, minutely papillate. Fruit a globose berry, 0.35–0.7 cm in diameter, green, turning purplish black when ripe, the pericarp thin, shiny, opaque, glabrous; fruiting pedicels 1–2 cm long, ca. 0.5 mm in diameter at the base, ca. 0.6 mm in diameter at apex, strongly recurved, not persistent; fruiting calyx lobes appressed to the berry, the tips not reflexed. Seeds 35–45 per berry, ca. 0.8 mm long, ca. 0.6 mm wide, flattened-reniform, narrowing towards one end, yellow, the sub-laterally positioned hilum positioned towards the narrower end, the testal cells pentagonal in outline. Stone cells 4 per berry, 0.75–1 mm in diameter, scattered throughout, relatively large compared to the seeds, white or cream-coloured. Chromosome number: not known.

**Figure 25.**
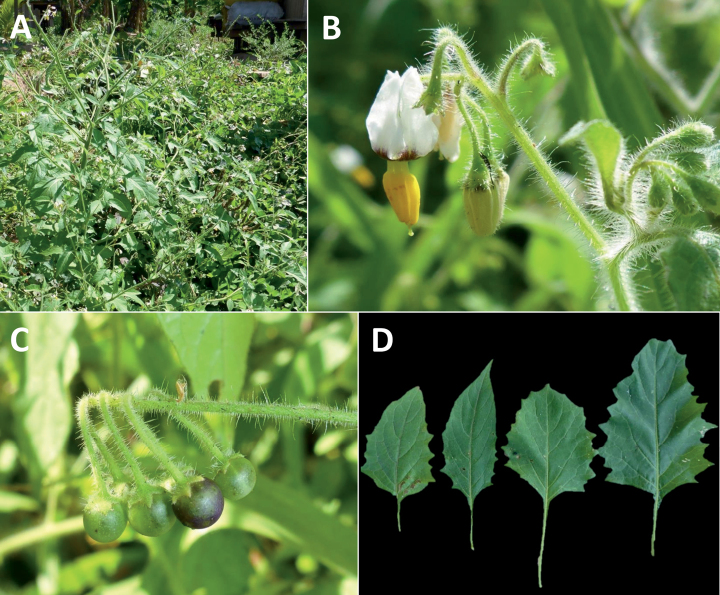
*Solanumarenicola***A** habit **B** buds and flowers, showing the dense glandular pubescence **C** maturing fruits with reflexed pedicels **D** leaf size and shape variation within an individual plant (**A–D***Särkinen & Balarezo 4866*). Photos by T. Särkinen. Previously published in Särkinen et al. (2015c: 54).

##### Distribution

**Figure 26.**
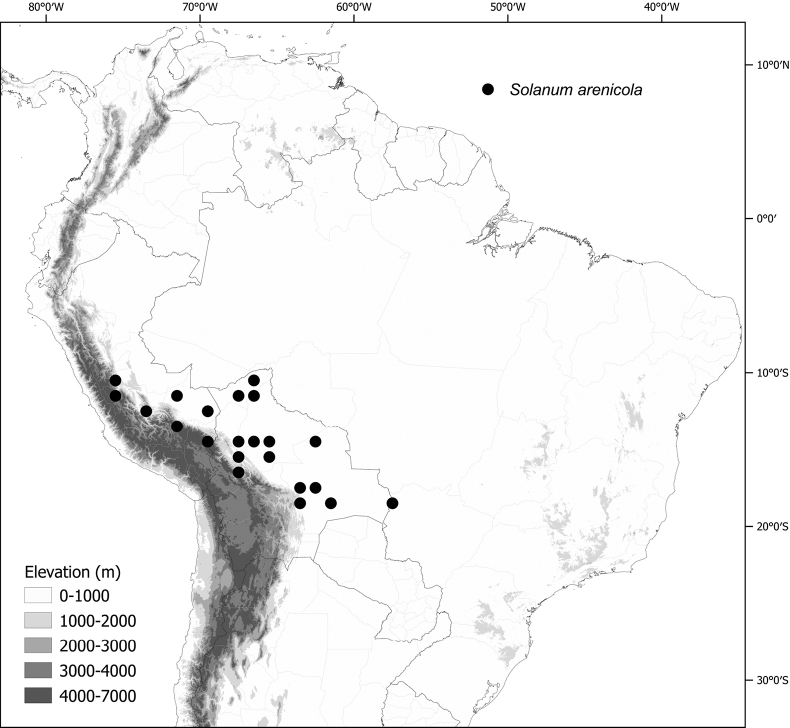
Distribution map of *Solanumarenicola*.

**(Fig. 26).***Solanumarenicola* occurs in the Amazonian slopes of the Andes in Bolivia (Depts. Beni, La Paz, Pando, Santa Cruz) and Peru (Depts. Cusco, Junín, Madre de Dios, Pasco, Puno). Currently, *S.arenicola* is known from central and southern Peru and from Bolivia, but it is likely that the species also occurs in adjacent areas of Brazil in the State of Rondônia, where the Río Madre de Dios and Río Beni join and cross into Brazil.

##### Ecology and habitat.

*Solanumarenicola* grows on sandbanks and river margins, tree fall gaps, and in disturbed sites near houses and fields in open, sandy soil in lowland moist rain forest, with occasional records from seasonally dry semi-deciduous forests, often associated with lowland rain forest pioneer species; from 0 to 600 (1,300) m elevation.

##### Common names and uses.

None recorded.

##### Preliminary conservation status

**(IUCN 2022).** Least Concern [LC]. EOO = 748,101 km^2^ [LC]; AOO = 164 km^2^ [EN]. *Solanumarenicola* grows in disturbed sites along rivers, tree falls, and cultivations where bare sandy soils are available, and its association with other pioneer species indicates that the species is not sensitive to human disturbance from expanding construction and agriculture. It occurs within protected areas in Peru (Parque Nacional Manu) and Bolivia (Parque Nacional Amboró).

##### Discussion.

*Solanumarenicola* is one of the few morelloids known from lowland humid forests in South America. It can be easily distinguished from *S.americanum*, the only other similar morelloid species found in these habitats in its larger anthers (3–4 mm long versus less than 1.5 mm long) and its glandular pubescence. Specimens without locality information can be easily confused with *S.nigrescens* of Central and northern South America, *S.aloysiifolium* of middle to high elevations in Argentina and Bolivia or *S.subtusviolaceum* of low to middle elevations in Peru and Bolivia. Both *S.arenicola* and *S.nigrescens* have unbranched inflorescences, but *S.arenicola* differs in having longer anthers (3–4 mm long) compared to *S.nigrescens* (2–2.5 mm long) and in the possession of glandular hairs (*S.nigrescens* is eglandular). The anthers are similar in size and shape to those of *S.aloysiifolium*, but *S.arenicola* has unbranched inflorescences and glandular pubescence, while *S.aloysiifolium* has forked inflorescences (sometimes many branched) and is eglandular. *Solanumarenicola* differs from *S.subtusviolaceum* in having internodal inflorescences (versus leaf-opposed), much reduced calyx lobes to only 0.5 mm long (versus 2–3.5 mm long), and a more exserted style extending 2–3 mm beyond the anther cone at anthesis (versus 0–0.5 mm).

#### ﻿8. Solanum arequipense

Taxon classificationPlantaeSolanalesSolanaceae

Bitter, Repert. Spec. Nov. Regni Veg. 11: 204. 1912.

Figs 27 , 28

Solanum furcatum Dunal var. subdentatum Nees, Nov. Act. Acad. Caes. Leop. 19, Suppl. 1: 386. 1843. Type. “Peruvia ad Arequipam, Aprili” *F.J.F. Meyen s.n.* (no specimens cited; no original material located). Peru. Arequipa: Prov. Arequipa, 2 km on dirt road from Cayma (northern outskits of Arequipa) to Charcani Grande, along Rio Chili; turn off from Cayma main road to ‘Egasa Centrales Hidroelectricas Charcani Santuario Virgen de Chapi’; within the Egasa hydroelectrical company’s perimeter ca. 50 m from the river, 2,518 m, 25 May 2012, *T. Särkinen, A. Mathews & P. Gonzáles 4099* (neotype, designated here: USM; isoneotypes: BM [BM001114853, BM001114854, BM001114856]).
Solanum furcatum Dunal var. subintegerrimum Nees, Nov. Act. Acad. Caes. Leop. 19, Suppl. 1: 386. 1843. Type. “Chile: Copiapó, Aprili; Peruvia: circa Tacoram [Volcán Tacora], Aprili” both syntypes collected by *F.J.F. Meyen s.n.* (no specimens cited; no original material located). Peru. Tacna: Prov. Tarata, Río Chacavira, camino a Caro, margen derecha de Río Chacavira, 3070–3480 m, 5 Dec 1997, *M.I. La Torre 1890* (neotype, designated here: USM [acc. # 159556]).

##### Type.

Peru. Arequipa: sin. loc., *C. Seler 204* (holotype: B, destroyed [F neg. 2597]; lectotype, designated here: LE [LE00016838]).

**Figure 27.**
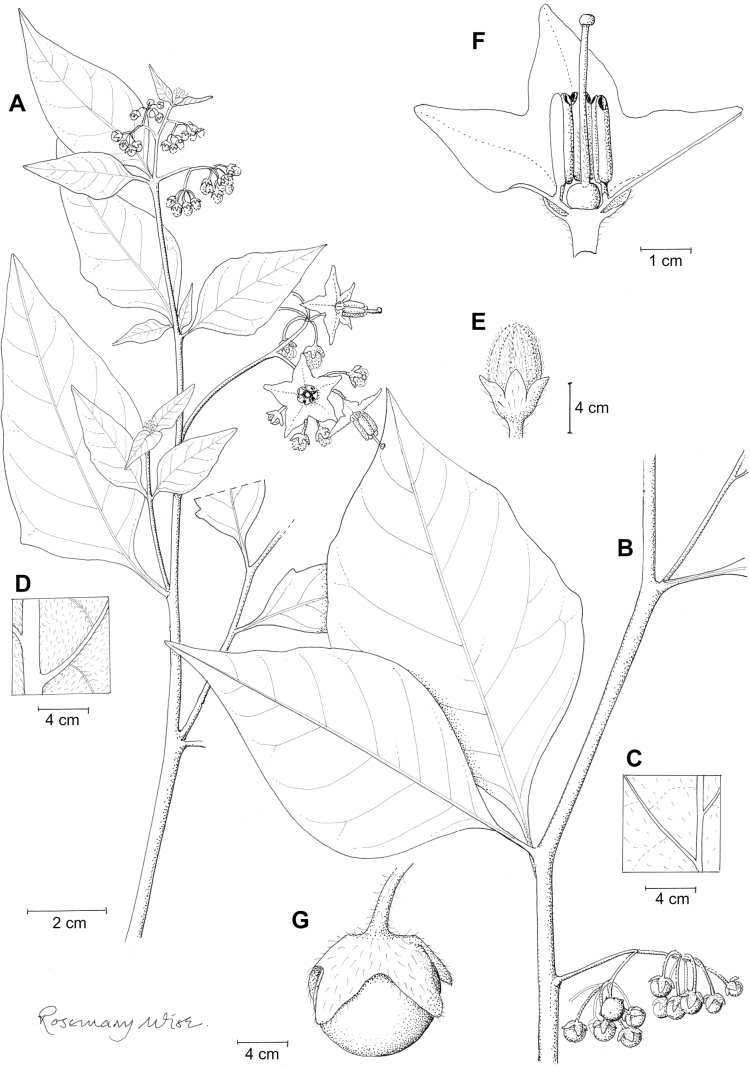
*Solanumarequipense***A** flowering branch **B** fruiting branch **C** detail of abaxial leaf surface **D** detail of adaxial leaf surface **E** flower bud **F** dissected flower **G** fruit (**A–G***Särkinen et al. 4095*). Illustration by R. Wise. Previously published in Knapp et al. (2019: 67) as *S.furcatum*.

##### Description.

Subwoody shrubs 0.3–1.5 m high, the branches erect. Stems terete or somewhat angled with a wing less than 0.5 mm wide and with a few spinescent processes along the angles, sparsely pubescent with white eglandular simple uniseriate 3–7-celled trichomes 0.5–1 mm long, these appressed and antrorse or somewhat spreading; new growth densely papillate with tiny glandular (?) 1-celled papillae and densely pubescent with white eglandular simple uniseriate trichomes like those of the stems. Sympodial units difoliate, the leaves geminate or not geminate. Leaves simple or occasionally toothed, the blades 3.2–14 cm long, 1.5–6 cm wide, larger on older branches, elliptic to somewhat ovate, widest in the lower half, membranous, more or less concolorous; adaxial surfaces almost glabrous to sparsely and evenly pubescent with erect eglandular simple uniseriate 5–7-celled trichomes of varying lengths to 1 mm long, these denser on the veins; abaxial surfaces almost glabrous to sparsely and evenly pubescent with simple uniseriate trichomes like the adaxial surfaces; principal veins 5–7 pairs, more densely pubescent than the lamina; base attenuate to truncate and abruptly attenuate, winged onto the petiole; margins entire or irregularly and shallowly toothed, the teeth ca. 2 mm long, ca. 10 mm wide, if present irregular in size and shape, the sinuses rounded and reaching ca. 1/10 of the way to the midrib; apex acute to acuminate; petioles 0.5–2 cm long, the winged portion narrowing towards base. Inflorescences internodal or opposite the leaves, forked or more than once forked (e.g., *Gonzáles et al. 2870*) with widely diverging branches, 2–6 cm long, with 10–20 flowers in the distal half of the branches, sparsely pubescent with appressed or slightly spreading eglandular simple uniseriate trichomes to 1 mm long like those of the stems; peduncle 1.1–3 cm long; pedicels 0.5–0.8 cm long, ca. 0.5 mm in diameter at the base, ca. 0.75 mm in diameter at the apex, filiform and slightly tapering, spreading at anthesis, sparsely pubescent to nearly glabrous like the rest of the inflorescence, articulate at the base; pedicel scars regularly spaced in the distal parts of the inflorescence branches ca. 1 mm apart. Buds globose, the corolla strongly exserted from the calyx before anthesis. Flowers 5-merous, cosexual (hermaphroditic). Calyx tube 1–1.5 mm long, conical to slightly cup-shaped, the lobes 1–2 mm long, 0.75–1 mm wide, elongate-deltate with the tips rounded or acute, sparsely pubescent with eglandular simple uniseriate trichomes like the stems and leaves, usually drying dark greyish black. Corolla 1.5–1.6 cm in diameter, white, white tinged with violet or pale violet, with a green eye, stellate, lobed ca. halfway to the base, the lobes 4.5–5 mm long, 4–5.5 mm wide, broadly deltate, reflexed or spreading at anthesis, adaxially glabrous, abaxially densely white puberulent with white simple uniseriate trichomes ca. 0.5 mm long. Stamens equal; filament tube minute; free portion of the filaments 1–1.2 mm long, densely pubescent adaxially with tangled transparent simple uniseriate trichomes; anthers 2.5–3 mm long, 1–1.5 mm wide, broadly ellipsoid, yellow, poricidal at the tips, the pores lengthening to slits with age. Ovary conical, glabrous; style 6–9 mm long, straight (curved in bud), long- exserted from the anther cone, densely pubescent in the lower third with transparent simple uniseriate trichomes; stigma globose or small-capitate, sometimes bilobed, the surface minutely papillate. Fruit a globose berry, 0.5–0.6 cm in diameter, pale green when immature, ripening to greyish green tinged with purple when ripe, the pericarp thick, matte, opaque, glabrous; fruiting pedicels 1–1.1 cm long, 0.75–1 mm in diameter at the base and apex, not markedly woody, strongly deflexed, not persistent; fruiting calyx not markedly enlarged or accrescent, the lobes to ca. 2 mm long, strongly appressed to the berry. Seeds 12–20 per berry, ca. 2 mm long, ca. 1.5 mm wide, flattened and teardrop shaped, reddish brown, the surfaces minutely pitted, the testal cells pentagonal in outline. Stone cells 2 per berry or absent, ca. 1 mm in diameter, cream-coloured. Chromosome number: 2n = 48 (Chiarini et al. 2017, voucher *Särkinen et al. 4083*, as *S.furcatum*). A count of 2n = 72 was reported by Edmonds 1977, based on *Hawkes et al. 4111*, but we have been unable to locate this voucher to verify its identity.

**Figure 28.**
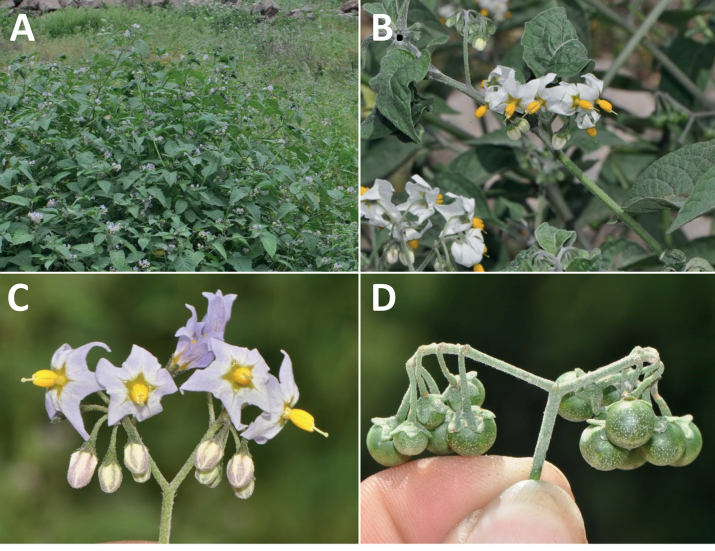
*Solanumarequipense***A** habit **B** flowering branch **C** flowers and buds **D** developing fruits (**A–D***Särkinen et al. 4084*). Photos by P. Gonzáles.

##### Distribution

**Figure 29.**
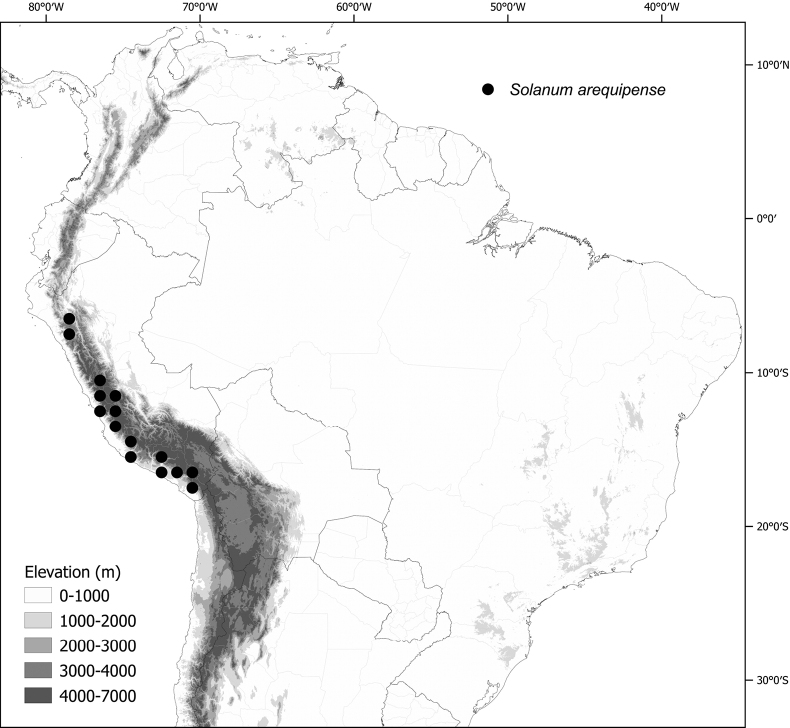
Distribution map of *Solanumarequipense*.

**(Fig. 29).***Solanumarequipense* is endemic to the slopes of the Andes in Peru (Depts. Ancash, Arequipa, Ayacucho, Cajamarca, Junín, Huancavelica, Lima, Moquegua, Puno, Tacna), occurring mostly on the western slopes of the cordillera.

##### Ecology and habitat.

*Solanumarequipense* grows in low elevation coastal ‘lomas’ formations and in open scrubby areas and along streams in higher elevation moist and cloud forests; from 200 to 4,400 m elevation.

##### Common names and uses.

Peru. Moquegua: hierba mora (*Núñez 6*). No uses recorded.

##### Preliminary conservation status

**(IUCN 2022).** Least Concern [LC]. EOO = 255,276 km^2^ [LC]; AOO = 224 km^2^ [EN]. *Solanumarequipense* is widely distributed in a wide range of habitats; like most morelloid species it thrives in disturbed areas. It occurs in several protected areas in Peru (Bosque de Zarate, Lomas de Atiquipa, Parque Nacional Huascarán).

##### Discussion.

*Solanumarequipense* is morphologically very similar to *S.furcatum* of central Chile and adjacent Andean Argentina and has been previously confused with that species (the plate published as *S.furcatum* in Knapp et al. 2019:67 is *S.arequipense* and is here reproduced with the correct identification). The species share forked inflorescences, globose buds with styles that are often exserted prior to anthesis, and greenish purple mature fruits. *Solanumarequipense* differs from *S.furcatum* in having no or only two apical stone cells in the fruits, while *S.furcatum* has more than six that are easily seen though the pericarp. Both species are tetraploid (see above and description of *S.furcatum*), but in analyses based on DNA sequence data the two species are not closely related (Gagnon et al. 2022); they may share different parentage. *Solanumpentlandii* also has similar globose buds and exserted styles but has much shorter anthers (less than 2 mm versus 2.5–3 mm in *S.arequipense*) and shiny green berries that lack stone cells. Plants of *S.arequipense* are generally woodier than those of *S.pentlandii* and occur at lower elevations. The two species are sympatric in central Peru, where *S.arequipense* has been collected at high elevations. Leaf lobing is usually more pronounced in *S.pentlandii*, but this is not consistent across the species range.

In describing *Solanumarequipense*, Bitter (1912b) cited a single specimen in the Berlin herbarium (F neg. 2597) that is now destroyed. We select here as lectotype the only duplicate of *Seler 204* we have seen, the sheet in the Komarov Institute in St. Petersburg (LE00016838); it is indicated as a gift from Berlin and the label “Solanum (Morella) arequipense Bitt. / 1912 Bitter” is in Bitter’s handwriting.

No herbaria were cited in the protologue of the descriptions of any of the four varieties of *S.furcatum* described by Nees von Esenbeck (1843) from the collections of Franz Meyen’s trip around the world (1831–32). The four taxa were distinguished based on leaf shape differences, a character notoriously variable in the Morelloid clade. Nees von Esenbeck (1843) cited two collections each for three varieties, mixing plants from the distributions of *S.arequipense* and *S.furcatum* (see discussion of *S.furcatum*). The only one citing a single collection was S.furcatumvar.subdentatum (Nees von Esenbeck 1843). Franz Meyen’s herbarium from his South American travels was held in B and destroyed, and we have found no duplicates of these collections (see also *S.furcatum*) nor were any specimens photographed by J.F. Macbride. We have chosen to neotypify var.subdentatum with a recent collection from near the single cited locality in Peru (Arequipa, *Särkinen et al. 4099*). Solanumfurcatumvar.subintegerrimum was based on collections from Copiapó in northcentral Chile and from the area around Volcán Tacora (border of Peru and Chile); we have not seen any collections from near Copiapó of either *S.furcatum* or *S.arequipense*, so we neotypify it with a collection from near the border of Peru and Chile at high elevation (*La Torre 1890*, USM acc. # 159556).

#### ﻿9. Solanum caatingae

Taxon classificationPlantaeSolanalesSolanaceae

S.Knapp & Särkinen, PhytoKeys 108: 3. 2018.

Fig. 30

##### Type.

Brazil. Bahia: Mun. Maracajú, Lagoa Itaparica 10 km W of São Inacio-Xique-Xique road at the turning 13.1 km N of São Inacio, 300–400 m, 26 Feb 1977, *R.M. Harley [with S.J. Mayo, R.M. Storr & T.S. Santos] 19125* (holotype: RB [RB00464327, acc. # 271981]; isotypes: CEPEC [acc. # 19367], K [K001336337]).

##### Description.

Perennial herbs, 0.4–1 m high, perhaps occasionally annual or only persisting for a few years. Stems terete or slightly angled, lacking spinose processes; young stems densely to sparsely pubescent with spreading glandular, simple uniseriate trichomes 0.5–1 mm long, the trichomes 4–15 celled, drying translucent; new growth densely glandular pubescent; bark of older stems greenish-brown or pale tan. Sympodial units unifoliate or difoliate, the leaves not geminate. Leaves simple, shallowly toothed, the blades 2.5–10 cm long, 1–4.5 cm wide, ovate to broadly elliptic, widest in the lower half, membranous, concolorous; adaxial and abaxial surfaces evenly glandular-pubescent with simple uniseriate trichomes to 2 mm long, these denser abaxially and along the veins, densely pubescent with minute glandular papillae on both leaf surfaces especially in young leaves; principal veins 4–6 pairs, drying paler than the lamina; base truncate and then abruptly attenuate on to the distal part of the petiole; margins shallowly and irregularly toothed, the teeth ca. 0.5 mm long, rounded at the tips and broadly deltate to semi-circular in outline; apex acuminate, the tip blunt; petiole (0.5) 1–2 cm, only winged from the attenuate leaf base in the distal half to third. Inflorescences internodal, unbranched or forked, subumbelliform with most flowers in the distal portion or spaced ca. 0.5 mm apart, 2–3.5 cm long, with 5–8 flowers, densely and finely glandular-pubescent like the stems and leaves; peduncle 1.8–3 cm long; pedicels 0.7–0.8 cm long at anthesis, ca. 0.5 mm in diameter at the base, ca. 0.7 mm in diameter at the apex, slender and tapering, densely glandular-pubescent with short uniseriate trichomes and glandular papillae, spreading at anthesis, articulated at the base but the articulation point somewhat swollen and leaving a minute stump that is darker in colour than the axis, this especially visible in fruiting material; pedicels scars closely packed in the distal part of the inflorescence to 0.5 mm apart, with the lowermost ca. 1 mm distant from the rest. Buds globose to broadly ellipsoid, the corolla strongly exserted from the calyx tube before anthesis. Flowers 5-merous, cosexual (hermaphroditic). Calyx tube 1–1.5 mm long, conical to broadly conical, the lobes 1–1.5 mm long, ca. 1 mm wide, deltate and spathulate, densely glandular-pubescent like the pedicels with uniseriate trichomes and papillae, the tips rounded. Corolla 0.6–0.9 cm in diameter, white with a darker (green?) central star, stellate, lobed 2/3–3/4 of the way to the base, the lobes 2.5–3.5 mm long, 1.5–3 mm wide, triangular, reflexed to spreading at anthesis, the abaxial surfaces glabrous to sparsely papillate with a few glandular trichomes ca. 0.2 mm long. Stamens equal; filament tube minute; free portion of the filaments 0.5–1 mm long, glabrous or sparsely pubescent with a few weak tangled simple uniseriate trichomes adaxially at the very base; anthers 1.8–2.2 mm long, 0.7–1 mm wide, ellipsoid, bright yellow, smooth, poricidal at the tips, the pores elongating to slits with age. Ovary conical, glabrous; style 3.5–4 mm long, straight, exserted beyond the anther cone, sparsely glandular pubescent with weak tangled trichomes and papillae in the basal half where included in the anther cone; stigma minutely capitate, densely papillate, not markedly different from the style. Fruit a globose berry, 0.7–1 cm in diameter, green when young, maturing shiny black, the pericarp thin, not translucent when dry (drying black), opaque, glabrous; fruiting pedicels 0.9–1.2 mm long, tapering from a base ca. 1 mm in diameter to an apex 1–1.2 mm in diameter, not distinctly woody, spreading and becoming deflexed at fruit maturity, persistent and remaining on inflorescence; fruiting calyx not accrescent, the tube 1–1.5 mm long, the lobes 2–2.5 mm long, spreading and later reflexed, covering the lower ca. 1/4 of the berry, the abaxial surfaces not densely papillate (different from *S.americanum* where the surfaces are densely papillate). Seeds (30)50–80 per berry, 1–1.5 mm long, 1–1.2 mm wide, teardrop shaped with a subapical hilum, reddish-gold, the surfaces minutely pitted, the testal cells pentagonal. Stone cells absent. Chromosome number: Not known.

**Figure 30.**
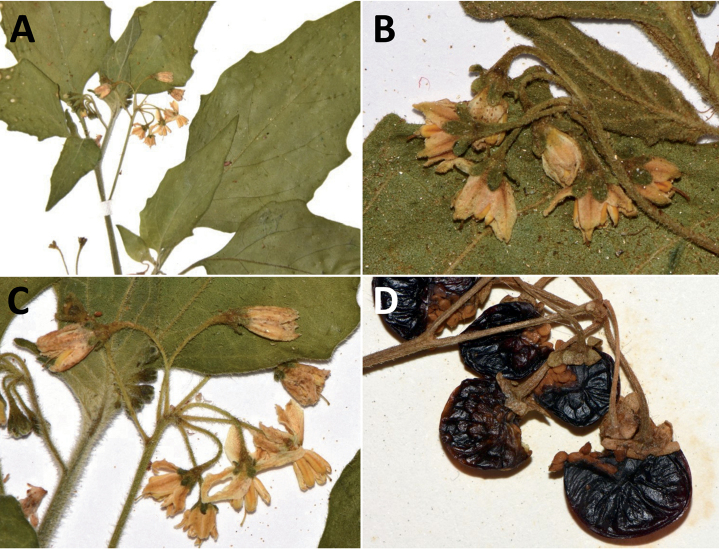
*Solanumcaatingae***A** habit **B** inflorescence in bud **C** inflorescence with flowers **D** mature, shiny black fruits with reflexed calyx lobes (**A, C, D***Harley et al. 19125* [RB 00464327, acc. # 27181] **B***Costa-Lima et al. 1862* [RB 01145300, acc. # 654975]). Reproduced with permission of Jardin Botânico de Rio de Janeiro.

##### Distribution

**Figure 31.**
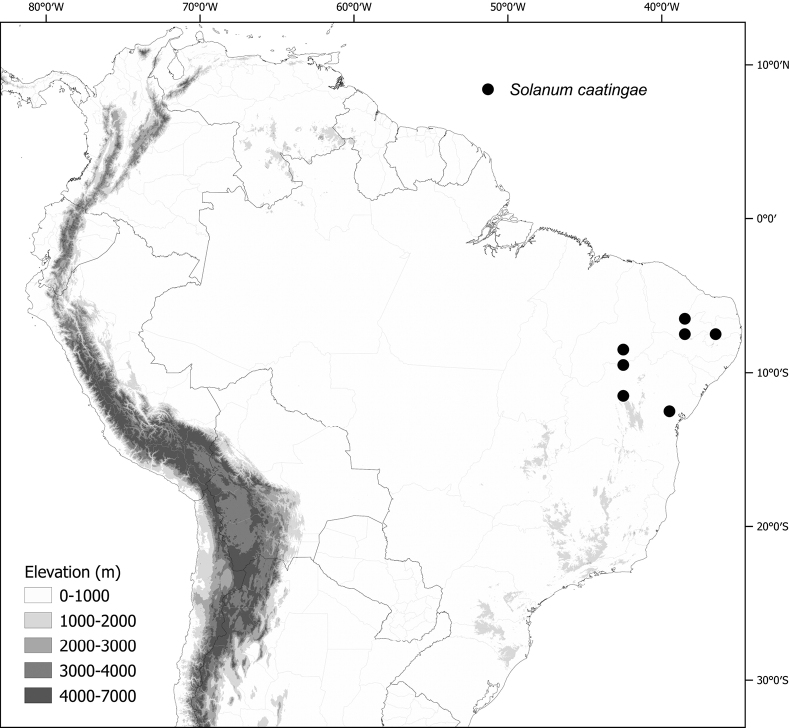
Distribution map of *Solanumcaatingae*.

**(Fig. 31).***Solanumcaatingae* is endemic to Brazil; widely scattered collections are known from the States of Bahia, Ceará, Paraiba, Piauí and Goiás.

##### Ecology and habitat.

*Solanumcaatingae* grows in dry formations known as “caatinga” or “savana estépica” (Eiten 1983; Prado 2003; IBGE 2004), between 300 and 400 m elevation. The caatinga is a complex mosaic of many biomes, ranging from the thorn forests of the caatinga proper (see Andrade-Lima 1981) to gallery forest, to humid forests on higher elevations (“brejos de altitude”) and cerrado savannas (Andrade-Lima 1981; Lleras 1997). Like many other morelloid species, *S.caatingae* apparently grows in somewhat disturbed and moist areas within the broader more xerophytic habitat and details of its ecological preferences will remain somewhat unclear until more field observations and collections can be made.

##### Common names and uses.

None recorded.

##### Preliminary conservation status

**(IUCN 2022).** Endangered (EN – B2 a, b(ii, iii, iv)). EOO = 267,575 km^2^ [LC]; AOO = 32 km^2^ [EN]. In spite of its large EOO, we suggest that *S.caatingae* merits the status of Endangered, as did Knapp and Särkinen (2018). The caatinga habitat is highly fragmented and under severe threat from fire and agriculture. Further studies in this dry forest habitat will certainly reveal more populations of this interesting species.

##### Discussion.

*Solanumcaatingae* is morphologically most similar to the widespread circumtropical weed *S.americanum*. It differs from *S.americanum* most strikingly in its spreading glandular pubescence of translucent trichomes (versus appressed eglandular pubescence of white trichomes), its usually more deeply and sharply toothed leaf margins and longer anthers (ca. 2 mm long versus ca. 1.5 mm long). Several other glandular pubescent species of herbaceous solanums occur in the dry forests of South America, but these are mostly from the Chaco biome and do not overlap in distribution with *S.caatingae* (see Särkinen and Knapp 2016). *Solanumcaatingae* can, however, be distinguished from these species (e.g., *S.michaelis*, *S.nitidibaccatum*, *S.physalidicalyx*, *S.physaliifolium*, *S.tweedieanum* and *S.woodii*) by its calyx that is not accrescent in fruit with the lobes spreading or slightly reflexed and its shiny black berries with no stone cells. The glandular-pubescent Amazonian species *S.arenicola* differs from *S.caatingae* in its larger flowers (8–12 mm in diameter versus 6–9 mm in diameter), longer anthers (3–4 × 0.8–0.9 mm versus 1.8–2.2 × 0.7–1 mm) and smaller berries (3.5–7 mm versus 7–10 mm in diameter) that contain stone cells. *Solanumcaatingae* can be distinguished from *S.tweedieanum* in its smaller anthers (1.8–2.2 mm versus ca. 5 mm long), non-accrescent calyx in fruit (*S.tweedieanum* has an accrescent calyx) and distribution (northeastern Brazil versus Argentina, Bolivia, and Paraguay).

Sendtner (1846) included a specimen of *S.caatingae* (collected by E. Pohl from Rio Maranhão, probably *Pohl 2393* from W) in his concept of S.nigrumsubsp.atriplicifolium (Gillies ex Nees) Sendtn. (= *S.tweedieanum*). *Solanumcaatingae* can be distinguished from *S.tweedieanum* in its smaller anthers (1.8–2.2 mm versus c. 5 mm long), non-accrescent calyx in fruit (*S.tweedieanum* has an accrescent calyx) and distribution in low elevation Brazil versus the eastern slopes of the Andes in the Southern Cone.

#### ﻿10. Solanum caesium

Taxon classificationPlantaeSolanalesSolanaceae

Griseb., Abh. Königl. Ges. Wiss. Göttingen 24: 252. 1879.

Figs 32 , 33

Solanum oranense Bitter, Repert. Spec. Nov. Regni Veg. 13: 170. 1914. Type. Argentina: Salta: Orán, Río de las Piedras, 3 Nov 1911, *M. Lillo 10884* (lectotype, designated by Barboza et al. 2013, pg. 240: LIL [LIL001450]; isolectotypes: G, LIL [LIL001451], SI [051837, 075020]).

##### Type.

Argentina. Salta: Dpto. Orán, “Río Blanco, bei Oran”, 17 Oct 1873, *P.G. Lorentz & G. Hieronymus 351* (lectotype, designated by Barboza et al. 2013, pg. 240: GOET [GOET003491]; isolectotypes: B, destroyed [F neg. 2766], CORD [CORD00006114], F [fragment of B duplicate, V0073222F, acc. # 621208], GOET [GOET003490, GOET003489]).

**Figure 32.**
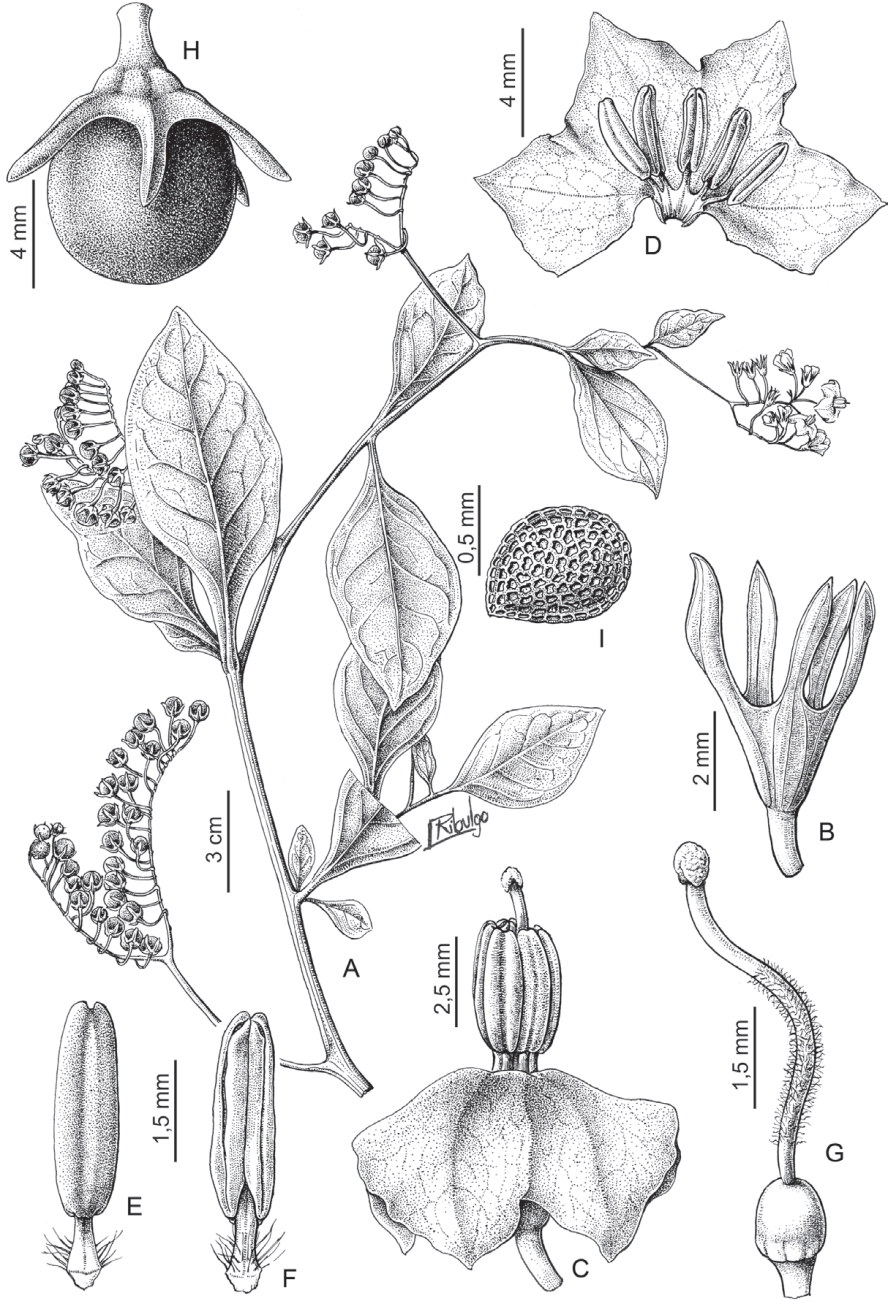
*Solanumcaesium***A** branch with flowers and fruits **B** calyx **C** flower **D** dissected flower **E** stamen, dorsal view **F** stamen, ventral view **G** gynoecium **H** fruit **I** seed (**A–I***Barboza et al. 2249*). Illustration by L. Ribulgo. Previously published in Barboza et al. (2013: 240).

##### Description.

Large sprawling perennial herbs forming patches 1–2 m in diameter, the branches sometimes to several metres long. Stems strongly angled with wings ca. 1 mm wide, slightly fleshy and watery or rubbery, glabrous or with a mix of eglandular and glandular (only in Bolivia, see below) simple uniseriate trichomes, the eglandular trichomes 4–6-celled, ca. 0.5 mm long, the glandular trichomes denser, 4–6-celled, to 1.5 mm long, the terminal gland a single cell; new growth densely papillate and glabrous to moderately pubescent with simple uniseriate trichomes like those of the stems; older stems green or yellowish green. Sympodial units difoliate, the leaves not geminate. Leaves simple, often toothed, the blades (2.4)7–13 cm long, (1.7)2.5–8 cm wide, elliptic-ovate to narrowly elliptic-ovate, widest in the lower half, membranous to fleshy (watery), concolorous but with very distinct calcium oxalate inclusions in the mesophyll (crystal sand); adaxial surfaces glabrous or with a few glandular or eglandular trichomes to 1 mm long on the lamina; abaxial surfaces with the lamina glabrous or densely glandular-pubescent along the veins, the lamina densely papillate; principal veins 6–8 pairs, often forking distinctly before the margin, drying yellowish green, glabrous or densely pubescent with eglandular or glandular simple uniseriate trichomes; base attenuate onto the petiole and then onto the stem; margins entire or with a few large teeth (both can occur on the same stems), the teeth 1.1–2 mm long, 2–3 mm wide, broadly deltate with acute apices, the sinuses rounded, reaching ca. 1.3 of the way to the midrib; apex acute; petioles winged from the leaf base, 0.5–6 cm long. Inflorescences internodal, usually forked, but occasionally unbranched, (4)8–20 cm long, with 10–40 flowers borne along the branches, glabrous or sparsely pubescent with eglandular and glandular simple uniseriate trichomes like the stems; peduncle 2.5–10 cm long; pedicels 0.9–1.1 cm long, 0.5–0.75 mm in diameter at the base, 1.2–1.5 mm in diameter at the apex, fleshy and tapering, spreading at anthesis, glabrous or sparsely pubescent to densely pubescent with glandular simple uniseriate trichomes like those of the stems and leaves, articulated at the base leaving a distinct cup ca. 0.5 mm deep; pedicel scars ca. 2.5 mm apart. Buds ellipsoid, the corolla included within the calyx lobes until just before anthesis. Flowers 5-merous, cosexual (hermaphroditic). Calyx tube 1.5–2 mm long, conical; the lobes 2.5–4.5 mm long, 0.75–1 mm wide, long triangular and slightly narrower near the lobe base, often somewhat unequal in size, glabrous or sparsely glandular-pubescent with simple uniseriate trichomes to 1 mm long like the rest of the plant. Corolla 1.6–1.8(2) cm in diameter, white, rotate to shallowly stellate, lobed ca. 1/4 of the way to the base, the lobes 2.5–3 mm long, 3–5 mm wide, broadly deltate, reflexed to spreading at anthesis, adaxially glabrous, abaxially densely papillate and with a few longer simple uniseriate trichomes to 0.4 mm long. Stamens equal; filament tube to 0.5 mm long; free portion of the filaments 0.75–1.1 mm long, densely pubescent adaxially with tangled transparent simple uniseriate trichomes; anthers 3–4 mm long, 1–1.2 mm wide, ellipsoid, yellow, poricidal at the tips, the pores lengthening to slits with age. Ovary conical, glabrous; style 6–7 mm long, straight, exserted beyond the anther cone, densely papillate-pubescent in the lower half to 2/3; stigma capitate or bi-lobed and slightly heart-shaped, bright green in live plants, the surface minutely papillate. Fruit a globose berry, 0.5–0.8 cm in diameter, green when immature, becoming greenish orange when ripe, the pericarp thin, shiny, translucent when ripe, glabrous; fruiting pedicels 1.2–1.5 cm long, 0.5–0.6 mm in diameter at the base, 1–1.1 mm in diameter at the apex, fleshy, strongly deflexed and secund with a kink at the base, not persistent; fruiting calyx somewhat accrescent, the tube to 3 mm long, the lobes to ca. 6 mm long, ca. 2 mm wide, appressed to and enclosing the berry like a cage. Seeds more than 100 per berry, ca. 0.75 mm long, ca. 0.5 mm wide, not markedly flattened, teardrop shaped, pale yellow or creamy tan, the surfaces minutely pitted, the testal cells more or less rectangular in outline. Stone cells 2 at the apex of the berry, ca. 1 mm in diameter, cream-coloured, larger than the seeds but barely distinguishable in herbarium specimens. Chromosome number: not known.

**Figure 33.**
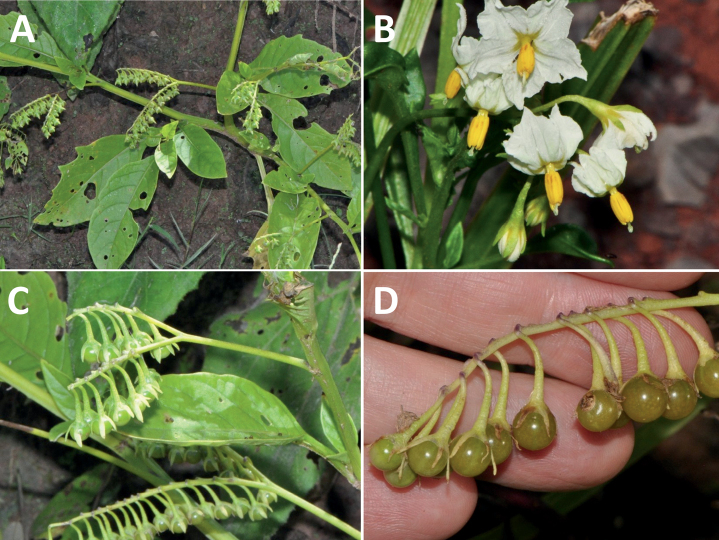
*Solanumcaesium***A** habit **B** inflorescence with buds and flowers **C** infructescence with developing fruits **D** mature fruits (**A, C, D***Barboza et al. 3530***B***Barboza et al. 3541*). Photos by S. Knapp.

##### Distribution

**Figure 34.**
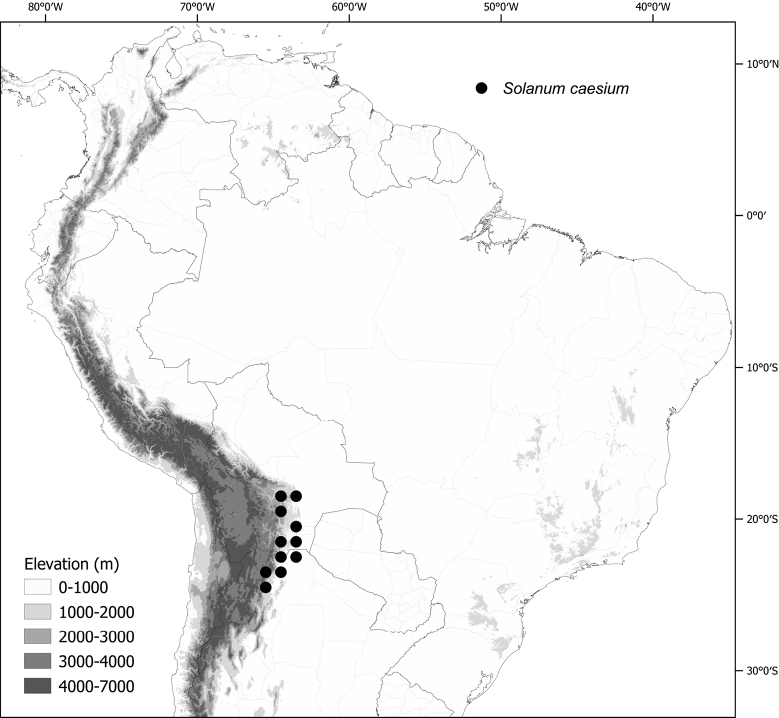
Distribution map of *Solanumcaesium*.

**(Fig. 34).***Solanumcaesium* is known from the eastern slopes of the Andes in Bolivia (Depts. Chuquisaca, Santa Cruz, Tarija) and Argentina (Provs. Jujuy, Salta).

##### Ecology and habitat.

*Solanumcaesium* grows in wet forests and semi-deciduous forests, often in disturbed areas such as landslides, along roads and streams; from 400 to 2,100 m elevation.

##### Common names and uses.

Bolivia. Tarija: flor de oro (*Coro-Rojas 1440*). No uses recorded.

##### Preliminary conservation status

**(IUCN 2022).** Least Concern [LC]. EOO = 117,146 km^2^ [LC]; AOO = 184 km^2^ [EN]. *Solanumcaesium* is widespread and common across its range and is a plant of disturbed areas. It occurs in protected areas in both Bolivia (Parque Nacional Serrania Aguarague) and Argentina (Parque Nacional Calilegua).

##### Discussion.

*Solanumcaesium* is distinctive and not easily confused with any other morelloid in South America. The fleshy, almost succulent leaves that are usually glabrous, lax forked inflorescences with spaced flowers and reflexed pedicels that develop a distinct kink at the base in fruit, long-triangular calyx lobes that enclose the yellowish orange berry like a cage and the rotate corolla are all found in combination only in *S.caesium*. The fleshy leaves are similar to those of some populations of *S.pentlandii*, but that is a species of high elevations in Peru and Bolivia and has much smaller stellate flowers that are usually violet. It has been suggested (Del Vitto and Petenatti 1999) that *S.caesium* is related to the members of the *Episarcophyllum* clade; molecular sequence data (Gagnon et al. 2022) show this is not the case, but that *S.caesium* is a species of somewhat uncertain affinities.

*Solanumcaesium* can form large plants and populations along open areas on roadsides and landslips. Plants throughout most of the species range are glabrous, except for populations from Santa Cruz (Bolivia) between Bermejo and Angostura where all plants seen have glandular pubescence (e.g., *Wood 8652*, *Nee 35614*, *Cardenas 4636*, *Nee 35134*, *Wood 22538*).

#### ﻿11. Solanum chenopodioides

Taxon classificationPlantaeSolanalesSolanaceae

Lam., Tabl. Encycl. 2: 18. 1794.

**Figure 35.**
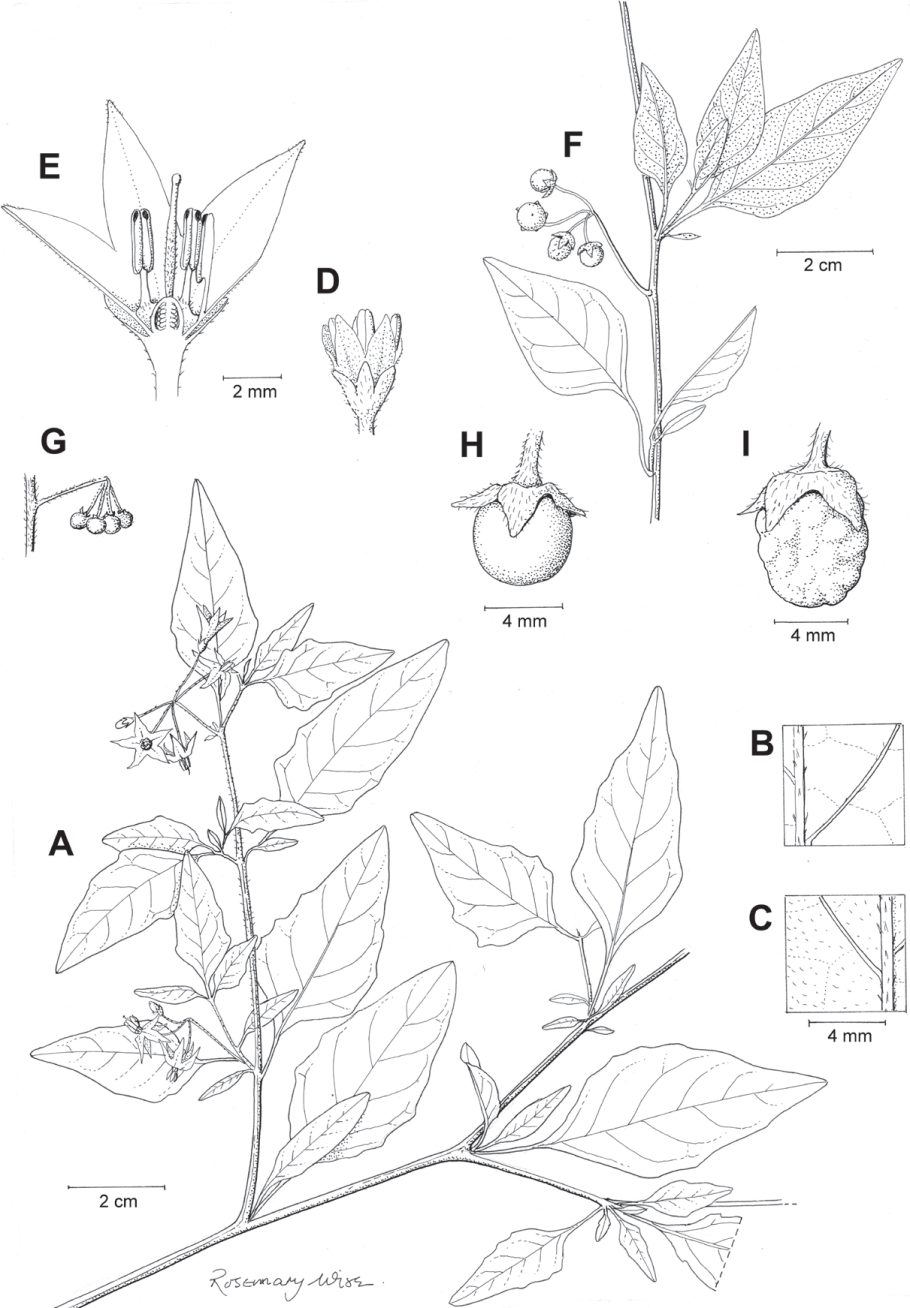
*Solanumchenopodioides***A** habit **B** detail of adaxial leaf surface **C** detail of abaxial leaf surface **D** opening bud **E** dissected flower **F** fruiting branch **G** detail of infructescence **H** maturing fruit **I** fully mature fruit (**A–E***Fox s.n.***F–I***Hieronymus s.n.*). Illustration by R. Wise. Previously published in Barboza et al. (2013: 242), Särkinen et al. (2018: 67) and Knapp et al. (2019: 46).

**Figure 36.**
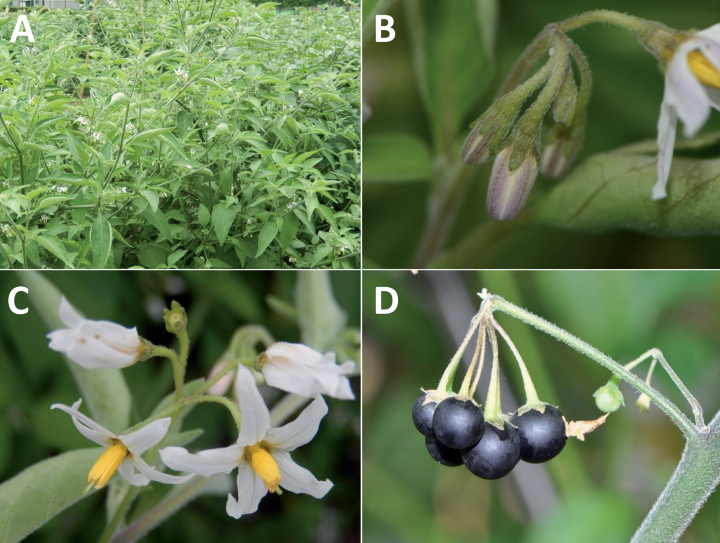
*Solanumchenopodioides***A** habit **B** buds **C** flowers at full anthesis **D** fully mature dull black fruits with appressed calyx lobes (**A–D** Nijmegen acc. # A14750051). Photos by S. Knapp and G. van der Weerden. Previously published in Särkinen et al. (2018: 68) and Knapp et al. (2019: 47).

Figs 35 , 36 Types based on American specimens only; for full synonymy, see Särkinen et al. (2018: 65–66)

Solanum sublobatum Willd. ex Roem. & Schult., Syst. Veg., ed. 15 bis [Roemer & Schultes] 4: 664. 1819. Type. Argentina. Buenos Aires, *Anon. s.n.* [probably *P. Commerson*] (*Herb. Willdenow 4336*) (lectotype, designated by Edmonds 1972, pg. 105 [as type ex photo]: B [B-W04336-01-0]).
Solanum besseri Weinm., Syst. Veg., ed. 15 bis [Roemer & Schultes] 4: 593. 1819. Type. “In America” [cultivated in Europe?], *Anon. s.n.* (no specimens cited; no original material located; neotype, designated by Särkinen et al. 2018, pg. 65: G-DC [G00144198]).
Solanum subspatulatum Sendtn., Fl. Bras. (Martius) 10: 45, tab. 4, figs 16–18. 1846. Type. Brazil. Sin. loc., *F. Sellow s.n.* (holotype: B, destroyed [F neg. 3183]; lectotype, designated by D’Arcy 1974a, pg. 735 [as type]: P [P00384051]; isolectotype: F [v0361921F, acc. # 621700, fragment]).
Witheringia chenopodioides (Lam.) J.Rémy, Fl. Chil. [Gay] 5: 69. 1849. Type. Based on Solanumchenopodioides Lam.
Solanum chenopodiifolium Dunal, Prodr. [A. P. de Candolle] 13(1): 44. 1852. Type. Argentina/Uruguay. “Buenos Aires et Montevideo”, *P. Commerson s.n.* (lectotype, designated by Edmonds 1972, pg. 108 [as holotype], second step designated by Särkinen et al. 2018, pg. 65: P [P00384081]).
Solanum gracile Dunal, Prodr. [A.P. de Candolle] 13(1): 54. 1852, nom. illeg., not Solanumgracile Sendtn. (1846). Type. Brazil. Rio de Janeiro: “Rio de Janeiro”, 1831–1833, *C. Gaudichaud 520* (lectotype, designated by Henderson 1974, pg. 46: G-DC [G00144391]; isolectotypes: G [G00343457], P [P00384052, P00384053]).
Solanum gracile Dunal var. microphyllum Dunal, Prodr. [A. P. de Candolle] 13(1): 54. 1852. Type. Argentina/Uruguay. “Circa Buenos Ayres et Montevideo”, *P. Commerson s.n.* (lectotype, designated by Morton 1976, pg. 151: P [P00384061, Morton neg. 8207]; possible isolectotype: F [v0073283F, acc. # 976485, fragment only]).
Solanum isabellei Dunal, Prodr. [A. P. de Candolle] 13(1): 153. 1852. Type. Uruguay. Montevideo, Lat. aust. 34°45'08", 1838, *A. Isabelle s.n.* (lectotype, designated by Särkinen et al. 2018, pg. 65: G-DC (G00145645); isolectotypes: F [v0073298F, acc. # 680251; v0073299F, acc. # 680253], K [K000585686], P [P00384071], W [acc. # 1889-115034]).
Solanum nodiflorum Jacq. var. microphyllum Hassl., Repert. Spec. Nov. Regni Veg. 9: 118. 1911. Type. Paraguay. Estrella: Mar, *É. Hassler 10271* (holotype: G [n.v.], Morton photo 8612).
Solanum vile Bitter, Repert. Spec. Nov. Regni Veg. 11: 221. 1912. Type. Brazil. Rio de Janeiro: Restinga do Harpoador, *E. Ule 4310* (lectotype, designated by Särkinen et al. 2018, pg. 66: CORD [CORD00004277]; isolectotype: HBG [HBG511507]).
Solanum gracilius Herter, Rev. Sudamer. Bot. 7: 266. 1943. Type: Based on (replacement name for) S.gracile Dunal.
Solanum ottonis Hyl., Uppsala Univ. Årsskr. 7: 279. 1945. Type. Based on (replacement name for) Solanumgracile Dunal.

##### Type.

Mauritius. “Ex ins. Mauritiana”, *Herb. Lamarck s.n.* (lectotype, designated by Barboza et al. 2013, pg. 242: P [P00357629]).

##### Description.

Annual herbs to short-lived perennial shrubs up to 1 m high, subwoody and branching at base. Stems terete, green-grey to straw colour, sprawling, somewhat weak and decumbent, not markedly hollow; new growth usually densely pubescent with simple, uniseriate appressed 1–6-celled eglandular trichomes, these 0.1–0.6 mm long; older stems more sparsely pubescent, glabrescent. Sympodial units difoliate, the leaves not geminate. Leaves simple, the blades 1.5–5.5(-7) cm long, 0.5–3(-3.5) cm wide, lanceolate to narrowly ovate, rarely ovate, widest at the middle or slightly below, membranous, discolorous; adaxial surface green, sparsely pubescent with appressed 1–4-celled translucent, simple, uniseriate trichomes like those on stem, these denser along the veins; abaxial surface pale grey, more densely pubescent with trichomes like those of the upper surface evenly distributed across lamina and veins; major veins 3–6 pairs, not clearly evident abaxially; base attenuate, decurrent on the petiole; margins entire or sinuate; apex acute to obtuse; petioles (0.5–)1–1.5(–3.5) cm long, sparsely pubescent with simple uniseriate trichomes like those of the stems and leaves. Inflorescences generally internodal but appearing to arise opposite the leaves on young shoots, unbranched or rarely forked, 1–2.5(–4) cm long, with 3–7(–10) flowers clustered near the tips (sub-umbelliform), sparsely pubescent with appressed 1–2-celled simple uniseriate trichomes; peduncle 1–2.3(–4) cm long, strongly deflexed downwards in fruit; pedicels 5–10 mm long, ca. 0.5 mm in diameter at the base and 1 mm in diameter at the apex, straight and spreading, articulated at the base; pedicel scars spaced ca. 0–1 mm apart. Buds elongate-oblong, the corolla only slightly exserted from the calyx tube before anthesis. Flowers 5-merous, cosexual (hermaphroditic). Calyx tube 2–3 mm long, conical, the lobes 0.6–1.2 mm long, less than 1 mm wide, broadly deltate to triangular with acute to obtuse apices, sparsely pubescent with 1–4-celled appressed hairs like those on stem but shorter. Corolla 0.6–1.2 cm in diameter, white with a black and yellow-green central portion near the base, the black colour usually distal to the yellow green, deeply stellate, lobed 4/5 of the way to the base, the lobes 3.5–4 mm long, 1.5–1.9 mm wide, strongly reflexed at anthesis, later spreading, densely puberulent-papillate abaxially with 1–4-celled simple uniseriate trichomes, these denser on the tips and margins. Stamens equal; filament tube minute; free portion of the filaments 0.6–1 mm long, adaxially pubescent with simple tangled uniseriate 4–6-celled simple trichomes; anthers (2-)2.3–2.8 mm long, 0.5–0.8 mm wide, narrowly ellipsoid, yellow, poricidal at the tips, the pores lengthening to slits with age and drying, the connective becoming darker brown with age in dry plants. Ovary globose, glabrous; style 3.7–4.5 mm long, straight, exserted beyond the anther cone, densely pubescent with 2–3-celled simple uniseriate trichomes in the lower half where it is included in the anther cone, exserted up to 1.5 mm beyond the anther cone; stigma capitate, minutely papillate, green in live plants. Fruit a globose berry, 0.4–0.9 cm in diameter, dull purplish black at maturity, the pericarp thin, matte and somewhat glaucous, opaque, glabrous; fruiting pedicels 0.6–1.3 cm long, (0.4)0.8–1.4 mm in diameter at the base, 1–2 mm in diameter at the apex, deflexed and slightly curving, not persistent, but the downwards pointing peduncle often persistent on older stems; fruiting calyx not accrescent, the tube less than 1 mm long, the lobes 1–1.5 mm long, appressed against the berry. Seeds (13-)20–35(-50) per berry, 1.2–1.4 mm long, 1–1.2 mm wide, flattened and teardrop shaped with a subapical hilum, pale yellow, the surfaces minutely pitted, the testal cells pentagonal in outline. Stone cells absent. Chromosome number: *2n* = 24 (see Särkinen et al. 2018).

##### Distribution

**Figure 37.**
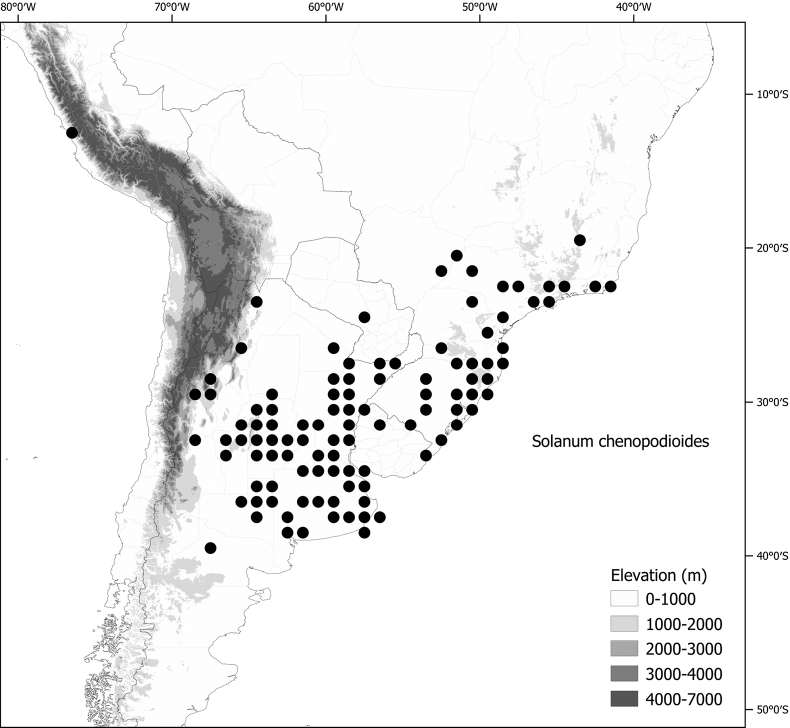
Distribution map of *Solanumchenopodioides* in South America. For distribution elsewhere see Särkinen et al. (2018: 70) and Knapp et al. (2019: 50).

**(Fig. 37).***Solanumchenopodioides* is native to southern South America, and has been introduced globally, largely with the wool trade. In South America it is known from the littoral of Argentina (Provs. Buenos Aires, Chaco, Córdoba, Corrientes, Entre Rios, Jujuy, La Pampa, La Rioja, Mendoza, Río Negro, Salta, San Luis, Santa Fé, Tucumán), southern Brazil (Provs. Mato Grosso do Sul, Minas Gerais, Rio de Janeiro, Paraná, Rio Grande do Sul, São Paulo and Santa Catarina), Paraguay (Depts. Amambay, Canindeyú, Itapúa, Presidente Hayes) and Uruguay (Colonia, Florida, Lavalleja, Maldonado, Montevideo, Rivera, Rocha, Salto, San José, Tacuarembó) with sporadic occurrences elsewhere, where it may be introduced.

##### Ecology and habitat.

*Solanumchenopodioides* is a weedy species, growing in disturbed areas in many different vegetation types, close to urban areas and human-altered habitats; from 0 and 2,400 m elevation.

##### Common names and uses.

Argentina. Buenos Aires: kushú-kushú (as *S.sublobatum*, Martínez Crovetto 1968), yerba mora (*Robles et al. 1869*); Córdoba: yerba mora (*Müller et al. 308*); Salta: iój s(l) I s(l) I (Vilela, as *S.gracile*, Martínez Crovetto 1965). Uruguay. Montevideo: yerba mora (*Barattini s.n.*). *Solanumchenopodioides* is considered to be toxic for cattle due to the high solanidine content of unripe fruits (Marzocca 1994), but as fruits ripen the alkaloid content decreases and fruits are apparently eaten without effect (Gallo 1987). In Argentina, fruits of *S.chenopodioides* are eaten by children of the Araucarian peoples (Martínez Crovetto 1968) and the Vilela people use them as a purple dye (Martínez Crovetto 1965).

##### Preliminary conservation status

**(IUCN 2022).** Least Concern [LC]. Worldwide distribution: EOO = 95,008,211 km^2^ [LC]; AOO = 1,560 km^2^ [LC]. *Solanumchenopodioides* is a widespread weed of disturbed areas (see Barboza et al. 2013; Särkinen et al. 2018; Knapp et al. 2019) and is widely introduced outside of and very common within its native range.

##### Discussion.

*Solanumchenopodioides* is a weedy, ruderal species occurring in open disturbed areas throughout its range. It is somewhat similar morphologically to *S.pilcomayense*, with which it is sympatric in Argentina, but differs in its elliptic leaves with acute to attenuate bases (versus triangular leaves with truncate to hastate bases), smaller anthers (2–2.8 mm long versus 3–4 mm long), and deltate or triangular versus spathulate calyx lobes. The fruiting peduncle of *S.chenopodioides* bends downwards at the base so it is held at an angle of ca. 45-degree with respect to the stem (see Figs 35G, 36D), but this character is not always obvious in herbarium specimens. Anthers in *S.chenopodioides* are always much longer (2–2.8 mm) than in *S.americanum* (0.8–1.5 mm), and the berries are matte (versus shiny) in texture and always lack stone cells (versus often with 2–4 stone cells per berry in *S.americanum*).

Typification details for the synonyms of *S.chenopodioides* and a more comprehensive discussion of its worldwide distribution as a weed of wool waste used in agriculture can be found in Särkinen et al. (2018) and Knapp et al. (2019).

#### ﻿12. Solanum cochabambense

Taxon classificationPlantaeSolanalesSolanaceae

Bitter, Repert. Spec. Nov. Regni Veg. 10: 553. 1912.

**Figure 38.**
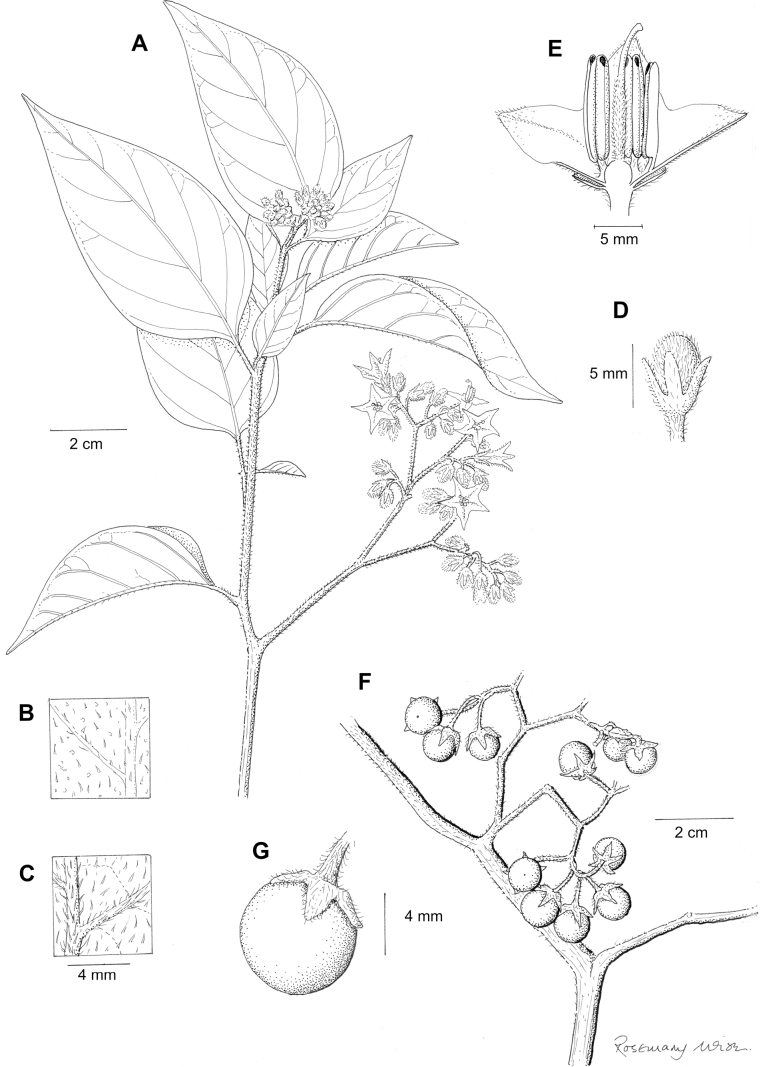
*Solanumcochabambense***A** flowering branch **B** detail of adaxial leaf surface **C** detail of abaxial leaf surface **D** flower bud **E** dissected flower **F** fruiting branch **G** maturing fruit (**A–D***Knapp et al. 10341*; E *Cárdenas 5577***F, G***Knapp et al. 10339*). Illustration by R. Wise.

**Figure 39.**
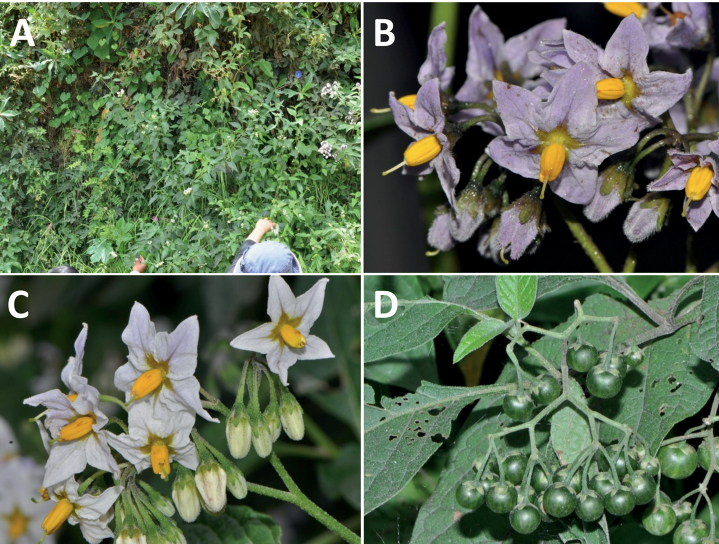
*Solanumcochabambense***A** habit **B** inflorescence with purple corollas **C** infructescence with white corollas **D** mature fruits (**A, C***Knapp et al.10363***B***Knapp 10287***D***Barboza et al. 3500*). Photos by S. Knapp.

Figs 3G, H , 4F , 38 , 39

Solanum extuspellitum Bitter, Repert. Spec. Nov. Regni Veg. 10: 555. 1912. Type. Bolivia. Tarija, 2,300 m, 30 Dec 1903, *K. Fiebrig 2439* (holotype: B, destroyed [F neg. 2711]; lectotype, designated here: F [V0361919F, acc. # 621247]).
Solanum extuspellitum Bitter subsp. subcoeruleum Bitter, Repert. Spec. Nov. Regni Veg. 10: 556. 1912. Type. Bolivia. Tarija, 2,300 m, 30 Dec 1903, *K. Fiebrig 2439* (holotype: B, destroyed [F neg. 2711]; lectotype, designated here: F [v0361919F, acc. # 621247]).
Solanum lorentzii Bitter var. tucumanicum Bitter, Repert. Spec. Nov. Regni Veg. 10: 556. 1912. Type. Argentina. Tucumán: sin. loc., *P.G. Lorentz & G. Hieronymus 1155* (holotype: B, destroyed; lectotype, designated by Barboza et al. 2103, pg. 236: CORD [CORD00004238]; isotypes: CORD [CORD00004239, CORD00004240], F [v0073320F, acc. # 50929], K [K000585687], SI [SI003323]).
Solanum decachondrum Bitter, Repert. Spec. Nov. Regni Veg. 11: 228. 1912. Type. Bolivia. Cochabamba: Cercado, May 1909, *O. Buchtien 2411* (lectotype, designated here: US [00027539, acc. # 700102]; isolectotypes: US [01014170, acc. # 1175973]).
Solanum decachondrum Bitter var. latiusculum Bitter, Repert. Spec. Nov. Regni Veg. 11: 229. 1912. Type. Bolivia. Cochabamba: Cercado, May 1909, *O. Buchtien 2412* (lectotype, designated here: US [00027538, acc. # 1177823]; isolectotypes: GOET [GOET009219], NY [00139124]).
Solanum decachondrum Bitter var. longiusculum Bitter, Repert. Spec. Nov. Regni Veg. 11: 229. 1912. Type. Bolivia. Cochabamba: Cercado, May 1909, *O. Buchtien 2411* (lectotype, designated here: US [01014170, acc. # 1175973]; isolectotype: US [00027539, acc. # 700102]).
Solanum probolospermum Bitter, Bot. Jahrb. Syst. 54, Beibl. 119: 10. 1916. Type. Peru. Huánuco: Valle del Río Pozuzo encima de Saria, 22 Jul 1913, *A. Weberbauer 6789* (no herbaria cited; lectotype, designated here: MOL[MOL00005139]; isolectotypes: B, destroyed [F neg. 2682], F [v0043286F, acc. # 647965], GH [01011893], MOL [MOL00005138], US [00027756, acc. # 1444969]).
Solanum lorentzii Bitter var. montigenum C.V.Morton, Revis. Argentine Sp. Solanum 136. 1976. Type. Argentina. Tucumán: Dpto. Chicligasta: Estancia Santa Rosa, 8 Jan 1927, *S. Venturi 4760* (holotype: US [03271889, acc. # 1548937]; isotypes: F [v0073318F, acc. # 695929; v0073319F, acc. # 637505], LP [LP010202, acc. # 010393], MO [MO-2127157, acc. # 960405] S [acc. # R-3117], SI [003322]).
Solanum montigenum (C.V.Morton) Cabrera, Fl. Prov. Jujuy 8: 435. 1983. Type. Based on SolanumlorentziiBittervar.montigenum C.V.Morton.

##### Type.

Bolivia. Cochabamba: Vic. Cochabamba, 1891, *M. Bang 1151* (lectotype, designated by Barboza et al. 2013, pg. 236: NY [00139097]; isolectotypes: BM [BM000617675], BR [BR0000005538553], CAL [acc.# 316673], E [E00190740], G [G00343347], GH [00077599], MO [MO-503629, acc. # 1815484], NY [00139096], PH [00030399], US [00610905, acc. # 92001; 00027515, acc. # 1324496], WIS [0256183WIS]).

##### Description.

Lax subwoody or woody shrubs, often vine-like with very long stems, to 5 m long, to 3 m if erect. Stems erect or sprawling, terete or slightly angled with tiny spinescent processes along the angles, moderately pubescent with eglandular white simple uniseriate 2–6-celled trichomes to 1 mm long, these soft and spreading; new growth densely white pubescent with eglandular simple uniseriate trichomes like those of the stems; bark of older stems pale brown, glabrescent. Sympodial units difoliate or plurifoliate, the leaves not geminate. Leaves simple or occasionally shallowly toothed, the blades 3.5–16 cm long, 1.5–8 cm wide, variable within an individual plant and always larger on lower stems, elliptic to narrowly elliptic, widest in the lower half, membranous, discolorous; adaxial surfaces sparsely pubescent with soft, spreading, eglandular simple uniseriate trichomes to 1 mm long, like those of the stems, these denser on the veins; abaxial surfaces more densely pubescent with simple uniseriate trichomes, the lamina still visible; principal veins 7–9 pairs, densely pubescent on abaxial surfaces; base acute, somewhat attenuate onto the petiole; margins entire or rarely shallowly toothed, the teeth if present in the basal part of the leaf, ca. 1 mm long, ca. 1.5 mm wide, with acute apices (see *Brooke 5125*, one duplicate entire, one toothed); apex acute to somewhat acuminate; petiole 0.5–2.8 cm long, slightly winged from the decurrent leaf bases in the distal part. Inflorescences internodal or terminating branches, several times branched, 3–13 cm long, with 10–80+ flowers clustered at the branch tips, moderately pubescent with soft, spreading eglandular simple uniseriate trichomes to 1 mm long like those of the stems; peduncle 1.7–10 cm long; pedicels 0.6–1 cm long, 0.5–0.75 mm in diameter at the base, 1–1.5 mm in diameter at the apex, tapering, spreading at anthesis, moderately pubescent like the inflorescence axes, articulated at the base; pedicel scars 0.5–1 mm part at the branched tips. Buds ellipsoid, occasionally somewhat inflated, the corolla strongly exserted from the calyx before anthesis. Flowers 5-merous, cosexual (hermaphroditic). Calyx tube 1–1.5 mm long, conical, the lobes 1.2–2 mm long, 1–1.5 mm wide, narrowly deltate, moderately pubescent with simple uniseriate trichomes like the rest of the plant. Corolla 2–3 cm in diameter, extremely variable through anthesis in size and colour, pale violet to whitish violet, with a pale greenish yellow eye, stellate, lobed 1/3 to 1/2 of the way to the base, the lobes 4–6 mm long, 4–5 mm wide, deltate or broadly deltate, spreading to slightly reflexed at anthesis, glabrous adaxially or with scattered uniseriate trichomes ca. 0.2 mm long at the tips and margins, abaxially densely papillate-puberulent with papillae and simple uniseriate 1–3-celled trichomes to 0.5 mm long along the lobe midveins, tips and margins, the interpetalar tissue glabrous. Stamens equal; filament tube to 0.25 mm long; free portion of the filaments 1–1.5 mm long, pubescent adaxially with densely tangled, transparent weak simple uniseriate trichomes; anthers 3.5–4.5 mm long, 1.2–1.5 mm wide, ellipsoid, yellow, poricidal at the tips, the pores lengthening to slits with age. Ovary conical, glabrous; style 9–10 mm long, straight at anthesis (curved in bud), exserted beyond the anther cone, densely pubescent with transparent simple uniseriate trichomes in the lower half; stigma clavate, somewhat bilobed or capitate, green or dark cream in live plants, the surface minutely papillate. Fruit a globose berry, (0.9)1–1.2 cm in diameter, green and usually maturing purplish black, the pericarp thin, matte, translucent when berry ripe, glabrous; fruiting pedicels 1–1.7 cm long, ca. 1.2 mm in diameter at the base, ca. 2 mm in diameter at the apex, slightly woody, deflexed or spreading, not persistent; fruiting calyx somewhat enlarged, the tube to 2 mm long, the lobes to 2 mm long, appressed to the berry. Seeds 30–50 per berry, 1.5–2 mm long, 1–1.5 mm wide, flattened and teardrop shaped, pale brown to golden tan, the surfaces minutely pitted, the testal cells elongate with sinuate walls. Stone cells 8–12 per berry, 0.5–1 mm in diameter, cream-coloured, distributed throughout the mesocarp. Chromosome number: n = 12 (Edmonds 1972, voucher *Steinbach 34*, as S.polytrichostylumvar.lorentzii; Moscone 1992, voucher *Subils et al. 3253*, as *S.aloysiifolium*; Moyetta et al. 2013, voucher *Barboza et al. 2152*, as *S.aloysiifolium*).

##### Distribution

**Figure 40.**
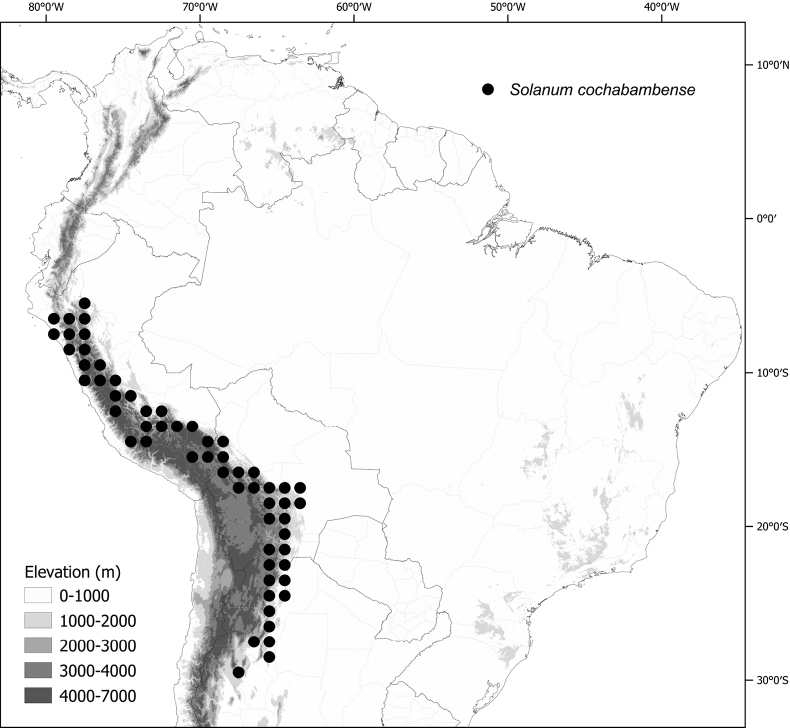
Distribution map of *Solanumcochabambense*.

**(Fig. 40).***Solanumcochabambense* occurs from the eastern Andean slopes from northern Peru (Depts. Amazonas, Ancash, Apurímac, Arequipa, Cajamarca, Cusco, Huancavelica, Huánuco, Junín, La Libertad, Puno, San Martín) throughout the Andean cordillera to Bolivia (Depts. Chuquisaca, Cochabamba, La Paz, Oruro, Potosí, Santa Cruz, Tarija) and northern Argentina (Provs. Jujuy, Salta, Tucumán).

##### Ecology and habitat.

*Solanumcochabambense* grows in a wide variety of middle to high elevation forest types, often at roadsides or in landslips and treefalls, from 150 to 4,120 m; most collections are from elevations above 1,000 m. The single collection from low elevation (*Roque 295* from 150 m in Camaná, Arequipa, Peru) comes from an area where landslides (‘huaicos’) are common and perhaps represents seeds washed down from higher elevations.

##### Common names and uses.

Bolivia. La Paz: chinchi-chinchi (*Beck 27781*), cusmayo (*Lewis 881659*). Peru. Ancash: atoqpa papán (papa de zorro) (*Gamarra 662*); Cusco: ccaya-ccaya (*Mexia 8079*), chinchi-chinchi (*Herrera 819*); muya khaya (*Franquemont et al. 297*); qusmayllu (*Franquemont et al. 348*); Huánuco: shopta (*Weberbauer 6789*); Puno: chitinqoya (Roersch 1994). In the southern Peruvian Quechua community of Chinchero (Cusco, Peru) leaves are used as cattle forage (Franquemont et al. 1990, as *S.aloysiifolium*) and as a wash for the head, especially for hangovers (Franquemont et al. 1990, as. *S.glandulosipilosum*). In southern Peru more generally leaves of *S.cochabambense* are used medicinally in a tea in the treatment of flu and colds (resfrío) and to counter difficulty in urination, and as a macerated plaster to alleviate rheumatic pains (Roersch 1994, as *S.aloysiifolium*).

##### Preliminary conservation status

**(IUCN 2022).** Least Concern [LC]. EOO = 7,244,968 km^2^ [LC]; AOO = 1,132 km^2^ [VU]. *Solanumcochabambense* is a common plant of disturbed areas. Further study may reveal variation that warrants taxonomic distinction, and this preliminary assessment will need revisiting. *Solanumcochabambense* occurs within several protected areas across its range (see Supplementary materials).

##### Discussion.

*Solanumcochabambense* is one of the most variable and widespread morelloid species in South America. Barboza et al. (2013) placed *S.cochabambense* in synonymy with *S.aloysiifolium*, with which it is sympatric in northern Argentina. Further study throughout the range of *S.cochabambense* confirmed the distinctness of the two species, but individual specimens collected in sympatry can be difficult to identify. *Solanumcochabambense* differs from *S.aloysiifolium* in its more highly branched inflorescences (those of *S.aloysiifolium* are usually only forked), buds that are ellipsoid rather than narrowly ellipsoid and larger corollas and berries. The anthers of *S.aloysiifolium* are narrow relative to their length (3.9–5 mm long and 0.6–1 mm wide in *S.aloysiifolium* versus 3.5–4 mm long and 0.9–1.2 mm wide in *S.cochabambense*) but this character can be difficult to see in the absence of comparative material. The berries of *S.cochabambense* are larger (1–1.2 cm in diameter) than those of *S.aloysiifolium* (0.5–0.6 cm in diameter), with similar numbers of stone cells.

In Bolivia *S.cochabambense* is partially sympatric with and morphologically very similar to *S.pallidum*. *Solanumpallidum* differs in its possession of dendritic trichomes, while *S.cochabambense* has only simple trichomes.

In the northern part of its range, *S.cochabambense* can be confused with *S.arequipense*, *S.juninense* and *S.interandinum*. *Solanumjuninense* differs in its possession of glandular trichomes whereas *S.cochabambense* is always eglandular. *Solanumarequipense* has blunt-tipped calyx lobes, anthers 2.5–3 mm long and a strongly capitate stigma, while *S.cochabambense* has long-triangular calyx lobes with acute apices, anthers 3.5–4 mm long and a clavate to only somewhat capitate stigma. The calyx lobes of *S.interandinum* are longer and more pointed than those of *S.cochabambense*, and the flowers are smaller (0.8–1.4(1.8) cm in diameter versus 2–3 cm in diameter in *S.cochabambense*).

The extreme variability seen across the range of *S.cochabambense* may indicate there are several distinct species contained within our rather broad circumscription. In some cases, duplicate collections from the same locality show that variation is present within a single population, which has helped us to recognise this group of specimens as a morphologically variable single species: an example of such variation is leaf margins varying from entire to toothed in duplicates of *Brooke 5125*. Similarly, variation in corolla shape and size was evident in the field in some populations, as well as inflorescence structure (e.g., *Knapp et al. 10391, Knapp et al. 10392, Knapp et al. 10393, Knapp et al. 10669*). Variation in other characters such as indumentum, calyx lobe shape and size, and other characters may represent fixed differences between populations, but based on our study of the specimens available across geographic space, we circumscribe this as a single highly variable species. Future studies at the population level throughout the range will be important to identify potential taxonomically recognisable segregates in this species.

Bitter (1912a) described *S.extuspellitum* and its variety *subcoerulum* using the same collection (*Fiebrig 2439*), citing “p. pt. herb. Berol.!” in each protologue. The sheet in B (now destroyed but photographed as F neg. 2711) has two stems, one with the label “Solanumextuspellitum n. sp.” and the other with the label “Solanum (extuspellitum subsp.) subcoeruleum Bitt.” – this latter suggesting he had originally considered naming the latter at the specific level. The stems differ only in pubescence density, and both are referrable to *S.cochabambense*. It is impossible to tell from which stem the fragment held in F came, so we are using it as the lectotype for both names. We have found no other duplicates of *Fiebrig 2439*.

Later that same year (Bitter 1912c), he described *S.decachondrum* and its varieties *longiusculum* and *latiusculum* using two collections of Otto Buchtien (*Buchtien 2411* and *2412*), citing no herbarium but indicating with “!” that he had seen them. Otto Buchtien’s private herbarium that Bitter cited was donated to the Smithsonian (US) in the 1920s (Morton and Stern 1966), so lectotypes for names based on Buchtien’s collections should be in US. Bitter cited both numbers in the protologue of the species, then used *Buchtien 2411* for var. longiusculum and *Buchtien 2412* for var. latiusculum. None of the duplicates of these collections we have seen has annotations in Bitter’s hand, but one duplicate in US (01014170, acc. # 1175973) is annotated as var. longuisculum by Buchtien and is here designated the lectotype of var. longuisculum. The other duplicate of *Buchtien 2411* at US (00027539, acc. # 700102) is designated as the lectotype of *S.decachondrum*. We lectotypify var. latiusculum with the US duplicate (000275538, acc. # 1177823) of *Buchtien 2412*, as it is the best preserved with both flowers and fruits and is annotated as var. latiusculum in Bitter’s hand.

Bitter (1916) described *S.probolospermum* citing *Weberbauer 6789* but without citing a herbarium. Many duplicates of this collection number have been preserved, and we select the better preserved of the two duplicates of *Weberbauer 6789* in the herbarium of the Universidad Nacional Agraria La Molina (MOL00005139) as the lectotype. Weberbauer’s original personal herbarium is held in MOL.

#### ﻿13. Solanum corymbosum

Taxon classificationPlantaeSolanalesSolanaceae

Jacq., Collectanea [Jacquin] 1: 78. 1787.

Figs 4B , 41 , 42

Solanum corymbiferum J.F.Gmel., Syst. Nat., ed. 13[bis] 2(1): 384. 1791, nom. superfl. illeg. Type. Based on Solanumcorymbosum Jacq. (cited in synonymy).
Solanum parviflorum Nocca, Ann. Bot. (Usteri) 6: 61.1793, nom. superfl. illeg. Type: Based on Solanumcorymbosum Jacq. (cited in synonymy).
Solanum parviflorum Salisb., Prodr.Stirp. Chap. Allerton 134. 1796, nom. superfl. illeg. Type. Based on Solanumcorymbosum Jacq. (cited in synonymy).
Solanum cymosum Ruiz & Pav., Fl. Peruv. [Ruiz & Pavon] 2: 31, t. 160. 1799. Type. Peru. “Habitat in Peruviae cultis, versuris et subhumidis locis per Limae et Chancay Provincias”, *H. Ruiz & J.A. Pavón s.n.* (lectotype, designated by Knapp 2008b, pg. 312: MA [MA-747100]).
Solanum corymbosum Jacq. var. cymosum (Ruiz & Pav.) Pers., Syn. Pl. (Persoon) 1: 223. 1805. Type. Based on Solanumcymosum Ruiz & Pav.
Solanum leptanthum Dunal var. parvifolium Dunal, Solan. Syn. 9. 1816. Type. Peru. Cajamarca: sin. loc., *F.W.H.A. von Humboldt & A. Bonpland s.n.* (lectotype, designated by Knapp et al. 2019, pg. 50: P [P00670610]; isolectotypes: P [P00136337, P00136338]).
Solanum azureum Van Geert, Cat. Gén. 1879–1880 [Van Geert]: Solanumazureum. 1879. Type. Cultivated in the nursery of Auguste Van Geert in Gand, Belgium, from seeds sent by Mr. Roezl from Peru (no specimens cited; no original material found).

##### Type.

Cultivated in Vienna [“Hort. Bot. Vindob.”] seeds said to be from Peru, *N. von Jacquin s.n.* (lectotype, designated by D’Arcy 1970, pg. 559: W [acc. # 0022473]).

**Figure 41.**
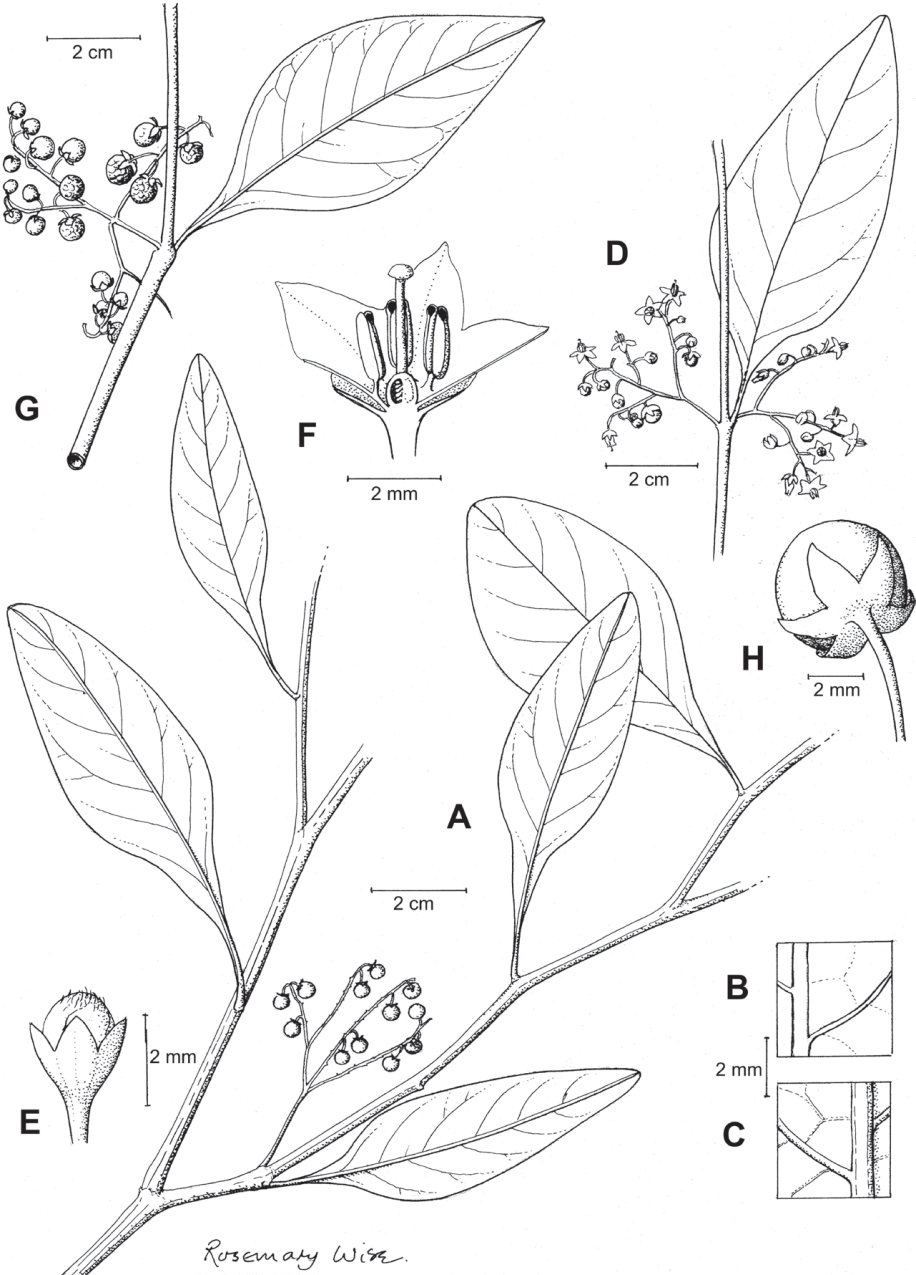
*Solanumcorymbosum***A** habit **B** detail of adaxial leaf surface **C** detail of abaxial leaf surface **D** flowering branch **E** floral bud **F** dissected flower **G** fruiting branch **H** maturing fruit (**A–F***van der Werff et al. 14657***G, H***Ochoa 14625*). Illustration by R. Wise. Previously published in Knapp et al. (2019: 52).

##### Description.

Annual to short-lived perennial subwoody herbs to 0.5 m high, branching at base. Stems terete, green to straw colour, sprawling, somewhat weak and decumbent, not markedly hollow; new growth nearly glabrous to sparsely pubescent with weak simple, uniseriate appressed 1–8-celled eglandular trichomes, these ca. 0.3 mm long; older stems glabrescent. Sympodial units difoliate or occasionally trifoliate, the leaves not geminate. Leaves simple, the blades 4.5–8 cm long, 1.5–4 cm wide, ovate-lanceolate, widest in the lower third, chartaceous to subcoriaceous, concolorous; both surfaces glabrous or sometimes sparsely ciliate near the base of the winged petiole; major veins 7–9 pairs, not clearly evident abaxially in live plants, paler in herbarium specimens; base long-attenuate, decurrent on the petiole; margins entire (in Peru rarely slightly 3-lobed, *Croat 58409*); apex acute; petioles 0.5–1 cm, glabrous to sparsely puberulent, winged to the base. Inflorescences internodal or opposite the leaves, 4–7 times branched, 2–3 cm long, with 20–50(-60) flowers spaced along the axis, nearly glabrous to sparsely pubescent; peduncle 0.1–2 cm, straight in fruit; pedicels 2–2.5 mm long, less than 0.5 mm in diameter at the base, ca. 0.5 mm in diameter at the apex, spreading, articulated at the base; pedicel scars spaced 1–3 mm apart. Buds globose, the corolla about halfway exserted from the calyx tube before anthesis, the tips of the corolla lobes often much more pubescent than the calyx. Flowers 5-merous, cosexual (hermaphroditic). Calyx tube 0.5–1 mm long, conical or broadly conical, the lobes 0.5–0.6 mm long, ca. 0.5 mm wide, broadly triangular, glabrous to very sparsely puberulent with simple, uniseriate trichomes. Corolla 0.5–1 cm in diameter, white or purple, the abaxial surface usually purple, rotate-stellate, the lobes 1–2.5 mm long, 1–1.5 mm wide, broadly triangular, reflexed at anthesis, later spreading, glabrous adaxially, minutely white-puberulent abaxially on the tips. Stamens equal; filament tube minute; free portion of the filaments ca. 0.2 mm long, adaxially pubescent with simple tangled white trichomes; anthers 0.8–1.5(-1.8) mm long, ca. 0.5 mm wide, ellipsoid, yellow, somewhat connivent, poricidal at the tips, the pores lengthening to slits with age. Ovary globose, glabrous; style ca. 2 mm long, straight, hardly exserted beyond the anther cone, pubescent in the lower 2/3 with tangled, white uniseriate simple weak-walled trichomes; stigma globose-capitate, minutely papillate, pale green in live plants. Fruit a globose berry, 0.4–0.6 cm in diameter, orange to red when ripe, opaque, the pericarp shiny or matte, translucent, glabrous; fruiting pedicels 0.2–0.3 cm long, ca. 0.5 mm in diameter at base, ca. 0.6 mm in diameter at the apex, strongly recurved at the very base, not persistent; fruiting calyx scarcely accrescent, the tube ca. 1 mm long, the lobes 1–1.3 mm long, appressed to the berry. Seeds 20–30 per berry, 1.5–1.8 mm long, 1.2–1.4 mm wide, flattened reniform with a central hilum, light yellow-tan or reddish brown in herbarium material, the surfaces minutely pitted, the testal cells with sinuate margins. Stone cells 2, ca. 1.5 mm in diameter, globose, prominent near the apex of the berry. Chromosome number: 2n = 24 (Chiarini et al. 2017, voucher *Särkinen et al. 4075*).

**Figure 42.**
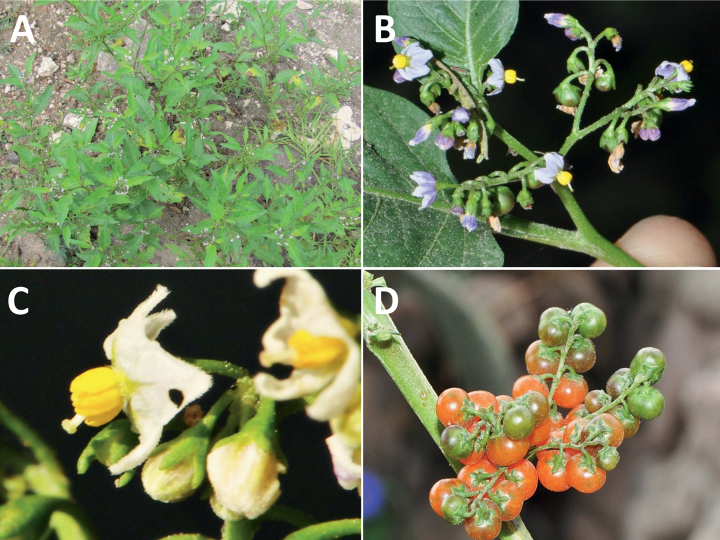
*Solanumcorymbosum***A** habit **B** inflorescence **C** flowers at full anthesis and buds **D** fully mature red-orange fruits with appressed calyx lobes (**A***Särkinen et al. 4604B***B, D***Särkinen et al. 4078***D***Särkinen et al. 4509*). Photos by T. Särkinen. Previously published in Knapp et al. (2019: 53).

##### Distribution

**Figure 43.**
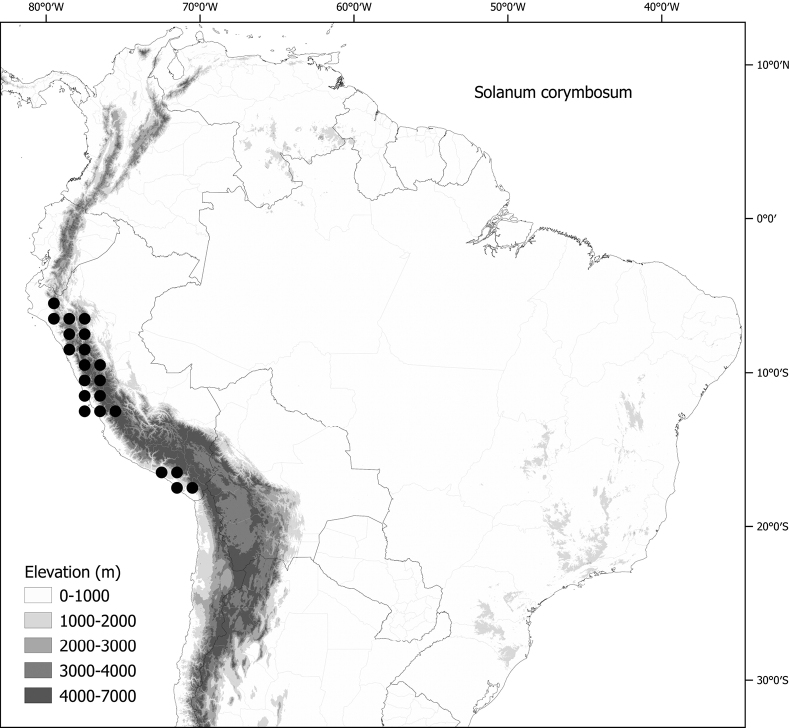
Distribution map of *Solanumcorymbosum* in South America. For distribution in Mexico, see Knapp et al. (2019: 54).

**(Fig. 43).***Solanumcorymbosum* occurs on the western slopes of the Andes in Peru (Depts. Amazonas, Ancash, Cajamarca, Huánuco, Lambayeque, La Libertad, Lima, Moquegua), and is naturalised in central and southern Mexico, possibly through introduction in colonial times (see discussion in Knapp et al. 2019).

##### Ecology and habitat.

*Solanumcorymbosum* grows in open, disturbed areas in landslides and along roads from sea level [in coastal lomas vegetation] to 2,900 m elevation.

##### Common names and uses.

Peru. Ancash: cchapchinya (*Gómez 51*); Cusco: ñuñuma, qusmayllu (Roersch 1994, as *S.radicans*); Huánuco: puslita mullaca (*Cárdenas 12275*); La Libertad: hierba mora (*Leiva et al. 707*); Lima: hierba mora (*Ferreyra 716*, *Ridoutt 11183a*); Puno: chetenguya (Roersch 1994, as. *S.radicans*). Roersch (1994) records use of leaves in a tea as treatment for sore throats and tonsillitis, macerated leaves as a plaster for swellings, and as a wash for colicky babies and for rheumatism (in combination with other medicinal plants). Although Roersch reports this plant as *S.radicans*, the illustration (Roersch 1994: 633) and description are clearly *S.corymbosum*.

##### Preliminary conservation status

**(IUCN 2022).** Least Concern [LC]. EOO = 338,062 km^2^ [LC]; AOO = 240 km^2^ [EN]; calculated using South American distribution only. *Solanumcorymbosum* has a disjunct distribution in Peru and Mexico; in its native range in Peru the species is quite widely distributed, but in Mexico potential morphological differences from Peruvian populations suggests it could be of conservation concern in its introduced range (Knapp et al. 2019). In Peru it occurs in several protected areas (e.g., Reserva Nacional Calipuy and the lower elevations of Parque Nacional Huascarán).

##### Discussion.

*Solanumcorymbosum* is a member of the Radicans group (Särkinen et al. 2015b) and has an unusual disjunct distribution in Peru and Mexico; Mexican populations are thought to represent an introduction of this species in post-Columbian times (Knapp et al. 2019). Populations in Mexico show nearly identical haplotypes to those from the coastal regions in Peru (Mitchell 2014), supporting this hypothesis.

*Solanumcorymbosum* can be distinguished from other members of the Radicans group in its simple, entire leaves, small orange to red fruits with two large apical stone cells, its highly branched inflorescences and diminutive flowers with rotate-stellate corollas that are usually white adaxially and purple abaxially. Other members of the group have 3- to 5-lobed leaves (e.g., *S.palitans*, *S.radicans*, *S.tripartitum*), although a population of *S.tripartitum* from the Province of Salta, Argentina appears to be uniformly simple-leaved. Corolla size of *S.corymbosum* overlaps with these plants at its upper range, but flowers of *S.corymbosum* are generally smaller (0.5–1 cm in diameter) than those of *S.tripartitum* (0.9–1.1 cm in diameter), and *S.tripartitum* has more than two stone cells per berry. The two species are not sympatric.

#### ﻿14. Solanum dianthum

Taxon classificationPlantaeSolanalesSolanaceae

Rusby, Bull. New York Bot. Gard. 4: 420. 1907.

Figs 44 , 45

Solanum hylobium Bitter, Repert. Spec. Nov. Regni Veg. 11: 223. 1912. Type. Bolivia. La Paz: Prov. Nor Yungas, Unduavi, Nov 1910, *O. Buchtien 768* (no herbaria cited; lectotype, designated here: US [00027609, acc. # 1176007]; isolectotypes: CORD [CORD00013412], GH [00077682], GOET [GOET003539, GOET003540], NY [00172030], US [00027608, acc. # 175975; 00650471, acc. # 7073337]).

##### Type.

Bolivia. La Paz: Nor Yungas, Unduavi, Sep 1894, *M. Bang 2492* (no herbaria cited; lectotype, designated here: NY [00139131], isolectotypes: F [v0073257F, acc. # 163985], GH [00077615], K [K000585512], MO [MO-503628, acc. # 3830685], NY [00139130], WIS [v0256186WIS]).

**Figure 44.**
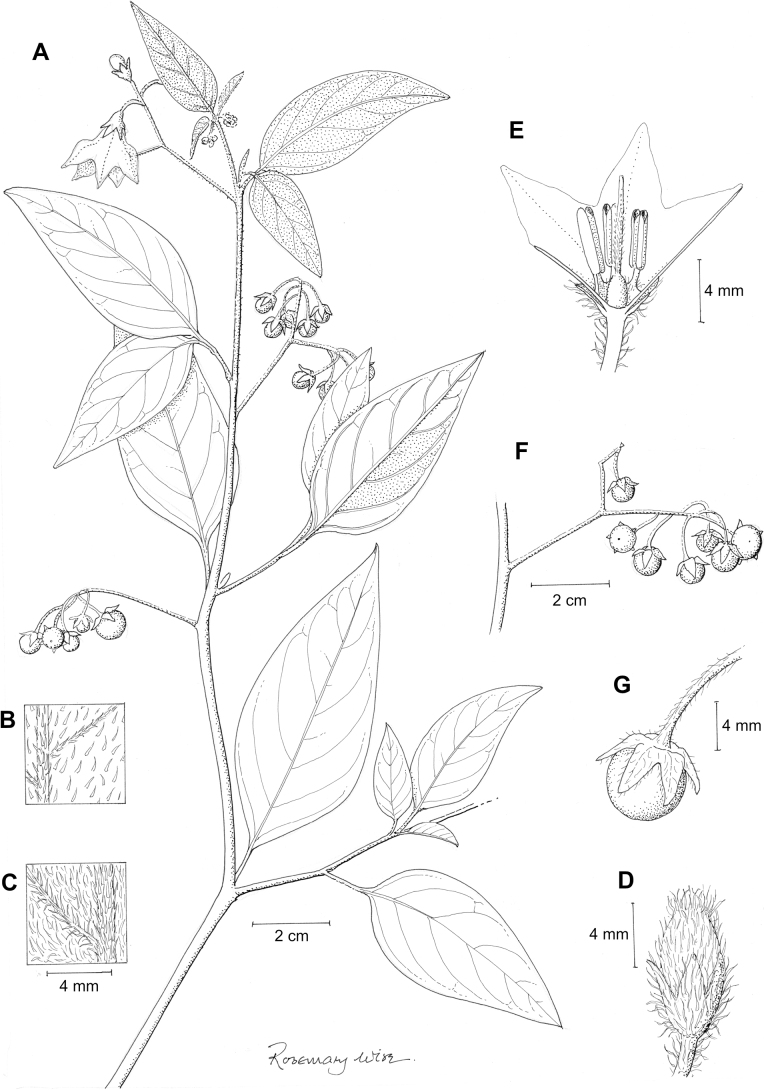
*Solanumdianthum***A** habit **B** detail of adaxial leaf surface **C** detail of abaxial leaf surface **D** flower bud **E** dissected flower **F** fruiting branch **G** maturing fruit (**A–F***Steinbach 231*). Illustration by R. Wise.

##### Description.

Weak straggly shrubs or suffrutescent herbs, to 2 m high, often supported on other plants. Stems terete or slightly winged, occasionally with spinescent processes, moderately to densely pubescent with transparent or translucent eglandular simple, uniseriate 6–10-celled trichomes to 2 mm long, these spreading or somewhat appressed (longer, more spreading trichomes in populations from Unduavi, Bolivia); new growth densely pubescent with the same trichomes as those of the stems; bark of older stems yellowish brown, glabrescent. Sympodial units difoliate, the leaves geminate and usually paired at the nodes. Leaves simple, the blades 1.5–9 cm long, 0.8–4 cm wide, narrowly elliptic to elliptic (ovate in some plants from Unduavi populations), widest at the middle or in the lower half, membranous, concolorous, but some plants from Sud Yungas, Bolivia (e.g., *Solomon 6043, 7297, 13691, 13854*) dark purple beneath; adaxial surfaces sparsely and evenly pubescent with eglandular simple uniseriate trichomes ca. 1 mm long, these to 6-celled, appressed to the lamina and antrorse or somewhat more spreading; abaxial surfaces similarly pubescent, but the trichomes denser on the veins; principal veins 6–8 pairs, drying yellowish below; base acute (truncate or sightly cordate in Unduavi populations); margins entire, occasionally with a few irregular teeth to 3 mm long, 3 mm wide; apex acute to slightly elongate-acute; petioles (0.3)0.5–1.8 cm long, highly dependent on size of leaves, pubescent like the stems and leaves. Inflorescences opposite the leaves or very occasionally internodal, unbranched or occasionally forked, 1–4 cm long, with 2–6 flowers clustered at the tips of the branches, moderately pubescent with eglandular transparent or translucent simple uniseriate trichomes ca. 1 mm long, these appressed or spreading; peduncle 0.9–3.8 cm long; pedicels 0.8–1.4 cm long, ca. 0.5 mm in diameter at the base, ca. 1.5 mm in diameter at the apex, moderately to sparsely pubescent with trichomes like the rest of the inflorescence, articulated at the base; pedicel scars tightly packed at the inflorescence branch tips to the lowermost ca. 1 mm distant. Buds globose to broadly elliptic, the corolla included within the calyx lobes until just before anthesis, densely white-pubescent. Flowers 5-merous, cosexual (hermaphroditic). Calyx tube 1.5–3 mm long, elongate cup-shaped, the lobes 1.5–4 mm long, 1.2–2 mm wide, triangular to somewhat spathulate with a constricted base, moderately to sparsely pubescent with transparent to translucent eglandular simple uniseriate trichomes to 1 mm long, these spreading or somewhat appressed, the tip acute or rounded, the sinuses rounded. Corolla 1.5–2.5 cm in diameter, violet, pale violet or occasionally white, with a yellow-green or dark purple central star, stellate, lobed 2/3 to 3/4 of the way to the base, the lobes 7–10 mm long, 2.5–5 mm wide, spreading at anthesis, adaxially glabrous, abaxially densely pubescent-puberulent with white eglandular simple uniseriate trichomes to 1.2 mm long, longest along the petal midveins and at the tips, the pubescence especially obvious in buds. Stamens equal; filament tube minute; free portion of the filaments 1.5–2 mm long, sparsely pubescent adaxially with tangled transparent simple uniseriate trichomes; anthers 3.5–5 mm long, 1–1.5 mm wide, ellipsoid, yellow, poricidal at the tips, the pores lengthening to slits with age. Ovary conical, glabrous; style 8–10 mm long, straight, exserted beyond the anther cone, densely pubescent in the lower half with simple uniseriate trichomes; stigma capitate to slightly bilobed, the surface minutely papillate, green in live plants. Fruit a globose berry, (0.5)0.9–1 cm in diameter, green or greenish black when mature, the pericarp thin, matte, opaque, glabrous; fruiting pedicels 1.3–1.5 cm long, 0.7–1 mm in diameter at the base, 1.5–2 mm in diameter at the apex, not markedly woody, deflexed (“fruit hanging” fide *Nee et al. 51880*), not persistent; fruiting calyx slightly enlarging, the lobes ca. 6 mm long, ca. 3 mm wide, spreading with the tips reflexed. Seeds 10–20 per berry, ca. 1.5 mm long, ca. 1.2 mm wide, ovoid teardrop shaped, not markedly flattened, pale brownish yellow or straw-coloured, the surfaces minutely pitted, the testal cells pentagonal to rectangular in outline with strength walls. Stone cells 4–6(8) per berry, scattered through the mesocarp, ca. 0.5 mm in diameter, cream-coloured. Chromosome number: not known.

**Figure 45.**
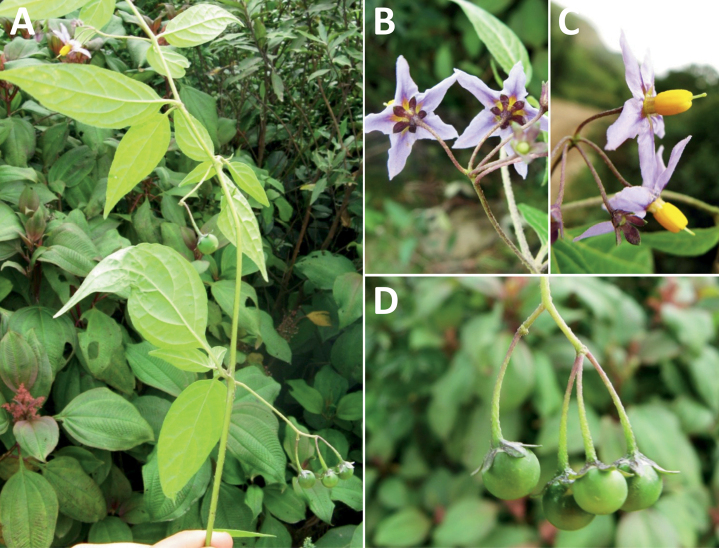
*Solanumdianthum***A** habit **B, C** flowers at anthesis **D** developing fruits (**A–D***Nee et al. 55311*). Photos by S. Stern.

##### Distribution

**Figure 46.**
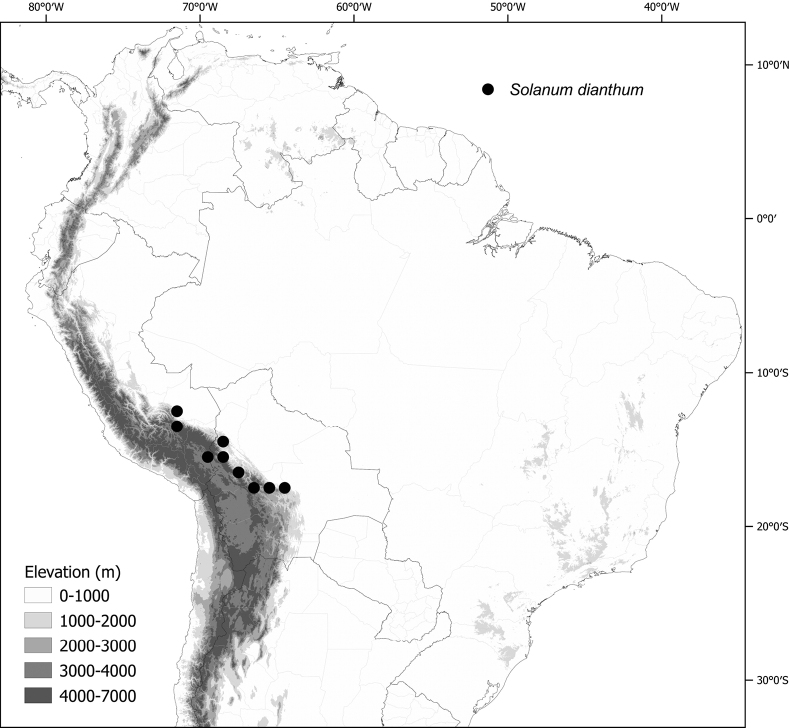
Distribution map of *Solanumdianthum*.

**(Fig. 46).***Solanumdianthum* occurs in the Andes of southern Peru (Dept. Cusco) and northern Bolivia (Depts. Cochabamba, La Paz, Santa Cruz).

##### Ecology and habitat.

*Solanumdianthum* grows in cloud forests, cloud forest margins and open grasslands at the edges of forests, often in tree falls or roadsides, from 1,640 to 3,900 m elevation.

##### Common names and uses.

None recorded.

##### Preliminary conservation status

**(IUCN 2022).** Least Concern [LC]. EOO = 79,792 km^2^ [LC]; AOO = 188 km^2^ [EN]. Like most morelloid species *S.dianthum* is a plant of open areas and has a relatively wide distribution. It occurs within protected areas in Bolivia (Area Natural de Manejo Integrado Apolobamba).

##### Discussion.

*Solanumdianthum* as circumscribed here is quite variable in pubescence, with some populations (notably those from around Unduavi, Bolivia) having loose spreading pubescence and somewhat more ovate leaves. Both this morphological variant and plants with appressed and somewhat strigose pubescence and more elliptic leaves are present on one of the sheets of the type collection (*Bang 2492*, NY, barcode 00139130). On an annotation slip on that sheet, C.V. Morton suggested that the small branch with looser pubescence in the centre of the sheet represented a different taxon. Examination of a range of specimens however suggest that this pubescence type grades into the more common appressed pubescence of the other sheets of *Bang 2492*, and that these collections, while on the face of it quite different in pubescence, are conspecific.

*Solanumdianthum* is somewhat similar morphologically to *S.leptocaulon*, but differs in its non-prostrate habit, stellate (versus campanulate) corollas and much larger anthers (3.5–5 mm long versus 2.5–3 mm long).

Most collections of *S.dianthum* have inflorescences opposite the leaves, but populations from around Siberia and Comarapa (Santa Cruz/Cochabamba, Bolivia) more or less uniformly have internodal inflorescences and white flowers with apparently reflexed corolla lobes at anthesis (e.g., *Nee & Solomon 34074*, *Davidson 3852*). These specimens are reminiscent of *S.subtusviolaceum*, but not glandular, and have the elongate calyx tube and slightly spathulate calyx lobes of *S.dianthum*. One of these collections, *Steinbach 231*, said on the label to be from “Angostura, Cercado de Santa Cruz 550m” is certainly mislabelled and instead is from Angostura in Prov. Cercado (Cochabamba) near the city of Cochabamba. Several collections from the northern part of the range have extremely large leaves and more robust, branched inflorescences than other collections of *S.dianthum*; these do, however, fall within the range of flower and fruit morphology for the species (e.g., *Lewis 88996*, *Valenzuela et al. 5933*). Further geographical sampling and molecular assessment across the entire range of *S.dianthum* as defined here will certainly clarify this complex set of morphologies.

*Solanumdianthum* was described using the collection *Bang 2492*, which has two duplicates in NY. One of these has a branch of apparently different material glued in the centre of the sheet (NY barcode 00139130), while the other is clearly from a single plant (NY barcode 00139131). Although the first of these has Bang’s original field label, we select the second (NY barcode 00139131) as the lectotype of *S.dianthum* in case future taxonomists feel the branch in the centre does represent a different species (see discussion above).

Bitter (1912b) described *S.hylobium* using *Buchtien 768*, but without citing a herbarium. We here select the best preserved of the duplicates we have seen (US, barcode 00027609, acc. # 1176007) as the lectotype of this name. The sheet is annotated as “Solanumhylobium Bitt., n. sp.” in Buchtien’s hand. Another sheet with the collection number *Buchtien 768* in US (barcode 02054047, acc. # 1177099) is not *Solanum*, but is instead a specimen of *Desmodiumtortuosum* (Sw.) DC. (Leguminosae) from a different locality “Millegasaya in Nord-Yungas” and different date “1917/XII”.

#### ﻿15. Solanum echegarayi

Taxon classificationPlantaeSolanalesSolanaceae

Hieron., Bol. Acad. Nac. Cienc. (Cordoba) 9: 58. 1881.

Figs 47 , 48

Solanum juncalense Reiche, Anales Univ. Chile 124: 459. 1909. Type. Chile. Región VII (Valparaiso): [Los Andes] Juncal [protologue: “Cordilleras de la provincia de Aconcagua, Juncal”], 15 Jan, *O. Buchtien 150* (no herbaria or collector cited; neotype, designated here: SGO [SGO000004574]).
Solanum hastatilobum Bitter, Repert. Spec. Nov. Regni Veg. 13: 246. 1912. Type. Argentina. San Luis: Quebrada del Salado, cerca de Bebida de las Varas, 9 Mar 1882, *C. Galander s.n.* (holotype: B [destroyed]; lectotype, designated by Barboza et al. 2103, pg. 249: CORD [CORD00004221]).
Solanum juncalense Reiche subsp. aconcaguae Bitter, Repert. Spec. Nov. Regni Veg. 12: 156. 1913. Type. Argentina. Mendoza: Dpto. Las Heras, “Puente del Inca, in viciniis montis Aconcagua”, 23 Feb 1903, *G.A. Malme 2956* (holotype: S [acc. # 10-15685]; isotypes: G [G00343486], MO [MO-256207, acc. # 2741560], US [00027638, acc. # 1572914]).
Solanum hastatilobum Bitter subsp. brachyphyllum Bitter, Repert. Spec. Nov. Regni Veg. 13: 171. 1914. Type. Argentina. San Juan: Dpto. Angaco: Cumbre del Gato, Cerro Pico de Palo, *T. Stuckert 7029* (lectotype, designated by Barboza et al. 2103, pg. 249: G [G00343383]).
Solanum glaberrimum C.V.Morton, Revis. Argentine Sp. Solanum 82. 1976. Type. Argentina. La Rioja: Quebrada de la Troya, 21 Feb 1941, *G. Covas 1235* (holotype: GH [00062989]; isotypes: LP [LP010903, acc. # 048953], NY [00076825], US [00027581, acc. # 2639762, fragment of GH holotype]).

##### Type.

Argentina. San Juan: Salida de la Quebrada del Leoncito, Jan 1876, *S. Echegaray s.n.* (holotype: CORD [CORD00004197]; isotype: US [00027559, acc. # 2678279]).

**Figure 47.**
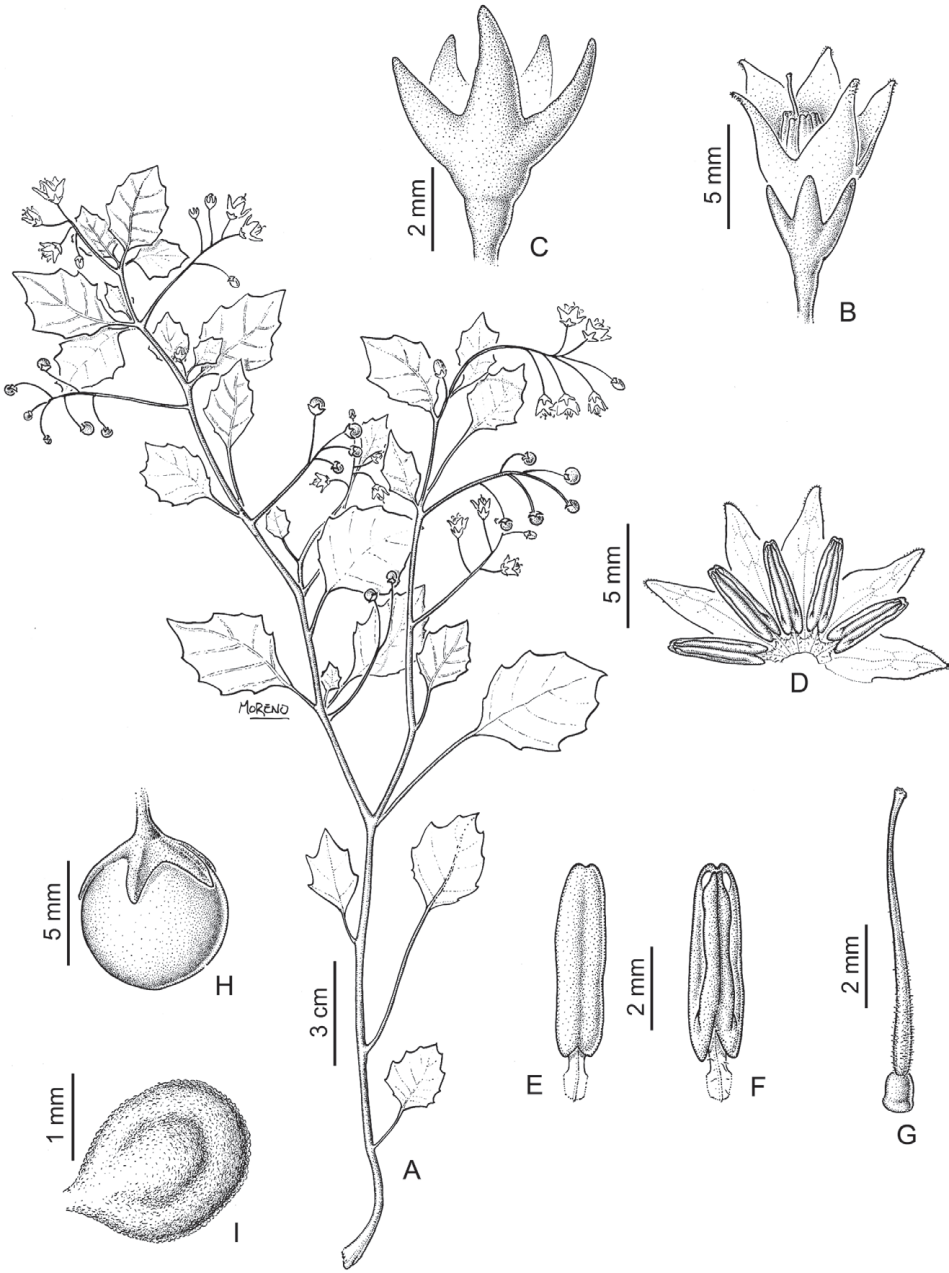
*Solanumechegarayi***A** flowering and fruiting branch **B** flower **C** calyx **D** dissected flower **E** stamen, dorsal view **F** stamen, ventral view **G** gynoecium **H** fruit **I** seed (**A–I***Biurrun et al. 5038*). Illustration by M. Moreno. Previously published in Barboza et al. (2013: 249), as *S.hastatilobum*.

##### Description.

Sprawling perennial herbs from woody rhizomes (underground rootstocks), prostrate to semi-erect, 0.1–0.5 m high, woody at the base, extremely variable in size depending on season of collection. Stems angled or slightly winged from the decurrent leaf bases, completely glabrous to sparsely and minutely pubescent with eglandular antrorse 1–2-celled simple uniseriate trichomes 0.1–0.2 mm long, these more like papillae, soon deciduous and the stems glabrescent; new growth glabrous to sparely papillate like the stems; bark of older stems pale tan or brown. Sympodial units difoliate, the leaves not geminate. Leaves simple and usually shallowly lobed, the blades (0.5)1.5–4.5 cm long, (0.3)0.5–2.2 cm wide, elliptic to ovate, widest at the middle or in the lower half, thick, fleshy and rubbery in texture in live plants, concolorous, extremely variable on individual plants and through the growing season; adaxial and abaxial surfaces glabrous, occasionally with a few scattered eglandular 1–2-celled simple uniseriate trichomes on the midrib; principal veins 3–5 pairs, often not visible in live or dried plants, if visible drying yellowish cream on herbarium specimens; base attenuate to truncate, always decurrent onto the petiole with a wing of leaf tissue; margins lobed, the lobes deltate, apically acute, often basiscopic (pointing towards stem), the sinuses reaching 1/4 to halfway to the midrib, revolute; apex acute; petiole 0.3–1.1 cm long, always winged with leaf tissue, glabrous or minutely puberulent with antrorse eglandular papillae. Inflorescences internodal or almost opposite the leaves, unbranched, (1)1.5–6.5 cm long, with 4–10 flowers, usually only 1–2 open at a time, glabrous or minutely puberulent with antrorse papillae like the rest of the plant; peduncle 0.5–2 cm long; pedicels 0.7–1.1 cm long, ca. 0.75 mm in diameter at the base, ca. 1.5 mm in diameter at the apex, tapering, glabrous or minutely papillate, articulated at the base; pedicel scars in pairs, each pair spaced ca. 2.5 mm apart. Buds ellipsoid, the corolla included in the calyx tube until just before anthesis due to rapid expansion of buds. Flowers 5-merous, cosexual (hermaphroditic). Calyx tube 1.5–2 mm long, conical, the lobes 2.5–4(5) mm long, 1–1.5 mm wide, long-triangular, rigid and fleshy, glabrous or minutely puberulent-papillate like the rest of the plant. Corolla 1.4–2 cm in diameter, white or pale violet, with a greenish yellow central eye edged with paler yellow, stellate, lobed ca. halfway to the base, the lobes 5–6 mm long, 2.5–4 mm wide, reflexed to spreading at anthesis, glabrous adaxially, glabrous or minutely puberulent abaxially with mixed eglandular simple uniseriate trichomes and papillae along the midvein, densely papillate at tips and margins. Stamens equal; filament tube less than 0.2 mm; free portion of the filaments ca. 1 mm long, glabrous or with a few eglandular tangled simple uniseriate trichomes to 0.5 mm long adaxially; anthers 4.5–5 mm long, 1–1.5 mm wide, ellipsoid, yellow, poricidal at the tips, the pores lengthening to slits with age. Ovary conical, glabrous; style 8.5–9 mm long, straight, exserted beyond the anther cone, minutely papillate in the lower half within the anther cone; stigma capitate, the surface minutely papillate, green in live plants. Fruit a globose berry, 0.7–1.2 cm in diameter, green or purplish green at maturity, the pericarp thin, shiny, opaque or slightly translucent, glabrous; fruiting pedicels 1–1.4 cm long, ca. 0.7 mm in diameter at the base, ca. 2 mm in diameter at the apex, spreading, not markedly woody, not persistent; fruiting calyx not accrescent, the lobes 2.5–4 mm long, 1–1.5 mm wide, spreading and slightly reflexed at the tips, fleshy and rubbery in live plants, somewhat woody in dried specimens. Seeds (5)10–20 per berry, ca. 2 mm long, 1.5–2 mm wide, reddish brown, teardrop shaped, the surfaces minutely pitted, the testal cells sinuate in outline in the seed centre, rectangular at the margins. Stone cells 10–12 per berry, 1–1.5 mm in diameter, pale creamy white. Chromosome number: n = 12 (Moscone 1992, voucher *Barboza 62*; Moyetta et al. 2013, voucher *Chiapella et al. 2630*, as *S.hastatilobum*).

**Figure 48.**
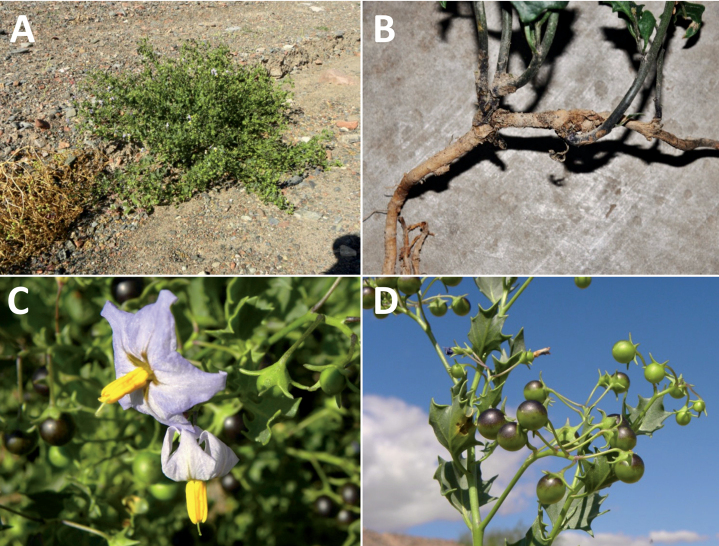
*Solanumechegarayi***A** habit **B** underground woody rhizomes **C** flowers at anthesis **D** immature and mature fruits (**A, C, D***Barboza et al. 4783***B***Knapp et al. 10540*). Photos by G.E. Barboza and S. Knapp.

##### Distribution

**Figure 49.**
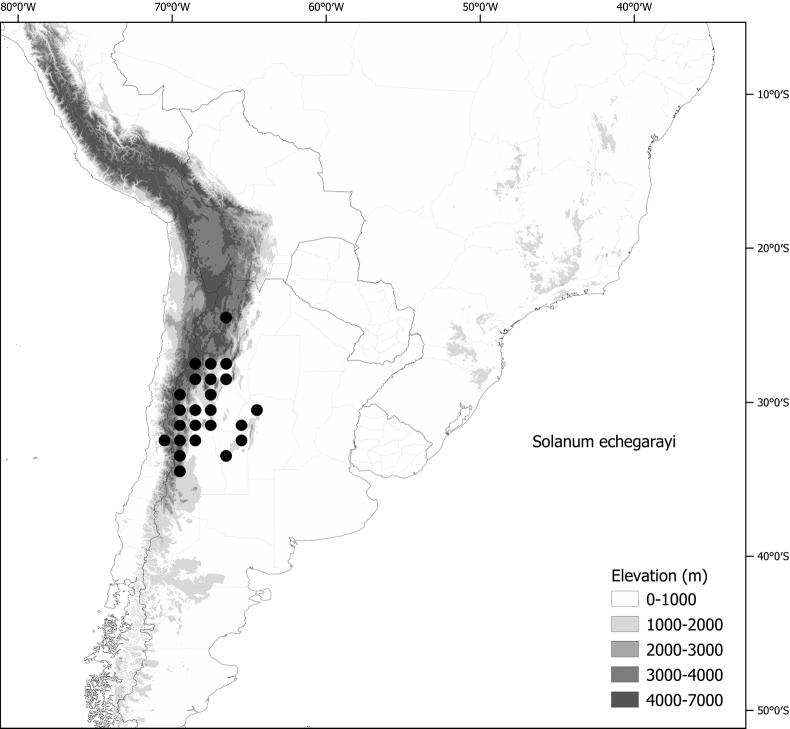
Distribution map of *Solanumechegarayi*.

**(Fig. 49).***Solanumechegarayi* occurs in the Andes of central Argentina (Provs. Catamarca, Córdoba, La Rioja, Mendoza, Salta, San Juan, San Luis) and adjacent Chile (Región V [Valparaíso]).

##### Ecology and habitat.

*Solanumechegarayi* grows in dry, scrubby habitats, usually at high elevation, and in open rocky areas, often where little other vegetation occurs, from 650 to 4,200 m elevation.

##### Common names and uses.

None recorded.

##### Preliminary conservation status

**(IUCN 2022).** Least Concern [LC]. EOO = 352,787 km^2^ [LC]; AOO = 408 km^2^ [EN]. *Solanumechegarayi* is widespread along the Andes and occurs in disturbed habitats. It is found in several protected areas throughout its range in Argentina (e.g., Parque Nacional Talampaya, Parque Provincial Volcán Tupungato, Parque Nacional El Leoncito).

##### Discussion.

*Solanumechegarayi* is a fleshy, almost succulent plant with deep woody rhizomes from which new shoots arise every growing season. It is a member of the *Episarcophyllum* clade (Särkinen et al. 2015b) together with *S.riojense* and *S.sinuatirecurvum*, but not related to *S.caesium* which has previously (Del Vitto and Petenatti 1999) been thought to have affinities to these taxa (Gagnon et al. 2022). All species of the *Episarcophyllum* clade are perennial herbs with woody underground rhizomes and occur in dry habitats in Argentina and neighbouring Chile, generally above 2,000 m elevation. The species all have slightly thick and fleshy leaves that appear succulent when compared to other species of the Morelloid clade.

*Solanumechegarayi* and *S.riojense* have long been confused due to a mix-up of type specimens (see below). *Solanumechegarayi* differs from *S.riojense* in its lack of cobwebby, tangled trichomes and in its sharply pointed rather than rounded calyx lobe apices. *Solanumsinuatirecurvum* also has cobwebby trichomes and differs from *S.echegarayi* in its much larger berries (more than 1 cm in diameter versus usually less than 1 cm in diameter) with a yellow, leathery pericarp rather than a green to greenish purple, somewhat translucent pericarp.

*Solanumechegarayi* is very variable depending upon when in the growing season the plant is collected; plants from early in the season are quite small and can look markedly different from those collected later in the season. In addition, specimens are often collected without the deep rhizomes, and so have the appearance of ephemeral annuals. Plants arise from deep underground stems (see Figs 2C, 48B) and the junction of vegetative shoots and rhizomes is quite fragile. Characters used to distinguish the species here recognised as synonyms of *S.echegarayi* that were regarded as distinct taxa by Del Vitto and Petenatti (1999) overlap broadly across the species range; plants from northern part of the range from lower elevations (described as *S.hastatilobatum*) tend to have smaller fruits that are green or purple and more hastate leaves, but all intermediate variations occur (for example, the collections *Barboza et al. 3447, 3450* from high elevation in La Rioja Province match these plants to some degree). The highest degree of variation occurs in the Province of San Juan, where intensive study of climatic and environmental conditions will prove useful in untangling patterns of vegetative variation. Molecular sequence data suggest there is some regional variation in *S.echegarayi* (Gagnon et al. 2022) but further sampling of all three species in this small clade (e.g., *S.echegarayi*, *S.riojense*, *S.sinuatirecurvum*) will be necessary to confirm these results.

Morton (1976) lectotypified *S.echegarayi* with a specimen in CORD (CORD00012856) labelled “Echegaray 472”, indicating the type in B was destroyed. Hieronymus used only specimens at CORD in his description (as clearly noted in the introduction to his catalogue of Echegaray’s collections; Hieronymus 1881); Morton’s (1976) lectotypification is thus superfluous. In addition, the specimen selected by Morton (1976) is densely pubescent with cobwebby hairs on the new growth and does not match the protologue, where the plant is described as completely glabrous; this specimen is likely a label mix-up for *Hieronymous & Neiderlein 472*, a syntype of *S.riojense* (see discussion under *S.riojense*). The sheets labelled “Echegaray 472” at CORD and SI (CORD [CORD00012856], SI [003309]) are therefore excluded from consideration as type material of *S.echegarayi*.

*Solanumjuncalense* was described from material from ”Cordilleras de la provincia de Aconcagua (Juncal, 2,200 m)”, with no collector or herbarium cited. A specimen in SGO (SGO000004574) from [Nevado] Juncal and the same elevation (*Buchtien 150*) and annotated “S. juncalense R” is almost certainly original material and is here selected as the neotype.

#### ﻿16. Solanum enantiophyllanthum

Taxon classificationPlantaeSolanalesSolanaceae

Bitter, Repert. Spec. Nov. Regni Veg. 11: 224. 1912.

Figs 50 , 51

Solanum itatiaiae Glaz. ex Edmonds, Kew Bull. 27: 109. 1972, nom. illeg., non Solanumitatiaiae Dusén (1907). Type. Brazil. Minas Gerais: Campos de l’Itatiaia, près du Rancho, 19 Nov 1876, *A. Glaziou 8867* (holotype: K [K000532495]; isotypes: P [P00336081, P00336082]).

##### Type.

Brazil. [Rio de Janeiro]: Serra do Itatiaia, Retiro do Ramos, 30 Jun 1902, *P. Dusén 663* (holotype: W [acc. # 1909-007993]; isotypes: S [acc. # 04-2909], US [00027566, acc. # 1055545]).

**Figure 50.**
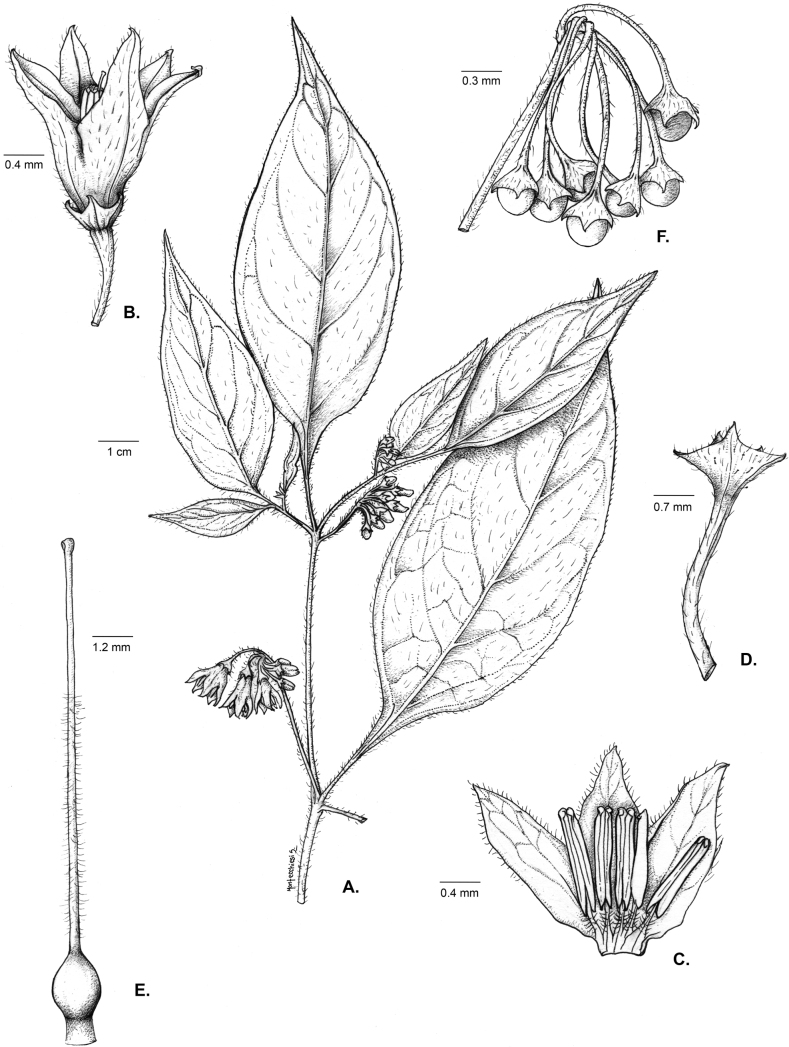
*Solanumenantiophyllanthum***A** flowering branch **B** flower **C** dissected flower **D** calyx **E** gynoecium **F** infructescence (**A–F***Hatschbach et al. 35846*). Illustration by S. Montecchiesi.

##### Description.

Herbs or subwoody shrubs with lax spreading branches, 1–2 m high. Stems terete, sparsely pubescent with scattered white eglandular 3–4-celled simple uniseriate trichomes 0.5–1 mm long, glabrescent with age; new growth densely pubescent with white eglandular 3–6-celled simple uniseriate trichomes 0.5–1 mm long, these spreading or laxly antrorse; bark of older stems pale greenish grey. Sympodial units difoliate, the leaves not geminate. Leaves simple, occasionally shallowly lobed, the blades 3–15 cm long, 1.5–9 cm wide, elliptic to ovate, widest in the lower third, membranous to chartaceous, slightly discolorous; adaxial surfaces very sparsely p ubescent on the lamina with a few scattered white eglandular 2–4-celled simple uniseriate trichomes to 0.5 mm long, these denser along the veins; abaxial surfaces with the lamina glabrous and a few scattered white eglandular trichomes like those of the adaxial surfaces along the veins; principal veins 5–6 pairs, pubescent above and below, pale above and dark below in herbarium specimens; base abruptly attenuate or truncate, not markedly decurrent along the stem; margins entire or very shallowly lobed in the basal quarter, especially in larger leaves, all margins ciliate-pubescent with white eglandular 2–4-celled simple uniseriate trichomes ca. 0.5 mm long; apex acute; petiole 0.5–1.5 cm long, sparsely pubescent with simple uniseriate trichomes like those of the veins. Inflorescences opposite the leaves, unbranched or occasionally forked, 1–3 cm long, with 3–7 flowers clustered at the tip and the inflorescence subumbellate, moderately pubescent with white eglandular simple uniseriate trichomes 0.5–0.7 mm long; peduncle 0.9–2.5 cm long; pedicels 0.8–1 cm long, ca. 0.5 mm in diameter at the base, ca. 1.2 mm in diameter at the apex, spreading at anthesis, pubescent with simple uniseriate trichomes like those of the inflorescence axis, articulated at the base; pedicel scars tightly packed at the tip of the inflorescence, to 1.5 mm apart in the most basal flowers. Buds elliptic to obovoid, the corolla strongly exserted from the calyx tube before anthesis. Flowers 5-merous, cosexual (hermaphroditic). Calyx tube 1.5–2 mm long, conical, the lobes 1–2 mm long, ca. 1 mm wide, narrowly deltate to triangular with acute apices, moderately pubescent with white simple uniseriate trichomes like those of the pedicel. Corolla 1.9–2 cm in diameter, white or white tinged with violet, with a purple-green central star, stellate, lobed ca. 2/3 of the way to the base, the lobes 8–9 mm long, 4–4.5 mm wide, spreading or slightly reflexed at anthesis, adaxially glabrous, abaxially densely puberulent-papillate with tiny simple uniseriate trichomes to 0.3 mm long. Stamens equal; filament tube minute; free portion of the filaments ca. 1.5 mm long, with a few tangled simple uniseriate trichomes adaxially; anthers 4.5–6 mm long, 1.2–1.5 mm wide, ellipsoid, yellow, poricidal at the tips, the pores lengthening to slits with age. Ovary conical, glabrous; style 8–9 mm long, straight, exserted beyond the anther cone, densely pubescent with weak trichomes and papillae in the lower third; stigma not enlarged, merely a broadening of the style tip, straight, the surface minutely papillate. Fruit a globose berry, 0.7–1 cm in diameter, green when mature, the pericarp thin, slightly shiny, translucent, glabrous; fruiting pedicels 1–1.2 cm long, ca. 0.5 mm in diameter at the base, tapering to ca. 1.5 mm in diameter at the apex, strongly deflexed, not persistent; fruiting calyx not markedly enlarged or accrescent, the tube appressed to the berry, the lobes to 2 mm long, spreading. Seeds 20–30 per berry, 1–1.2 mm long, 0.7–1 mm wide, teardrop shaped, the surfaces minutely pitted, the testal cells pentagonal in outline. Stone cells 4, in the distal half of the berry, ca. 0.4 mm in diameter, cream-coloured. Chromosome number: not known.

**Figure 51.**
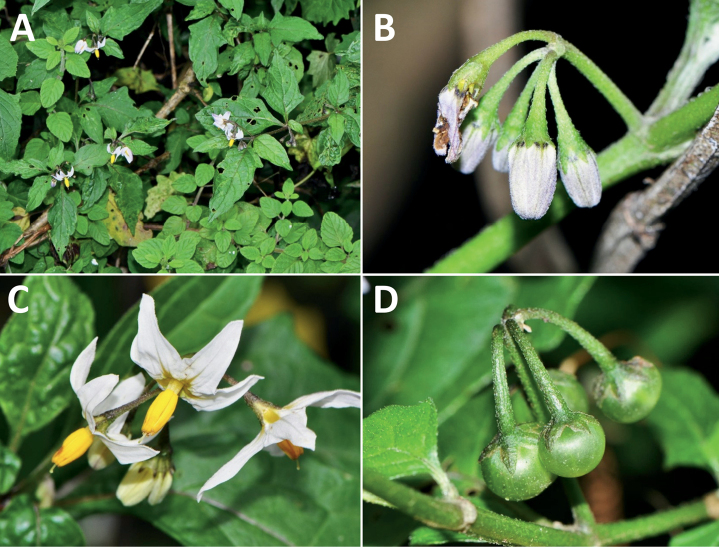
*Solanumenantiophyllanthum***A** habit **B** inflorescences with buds **C** inflorescence with flowers at full anthesis **D** Maturing fruits (**A,B, D***Giacomin et al. 2036***C***Giacomin et al. 2039*).

##### Distribution

**Figure 52.**
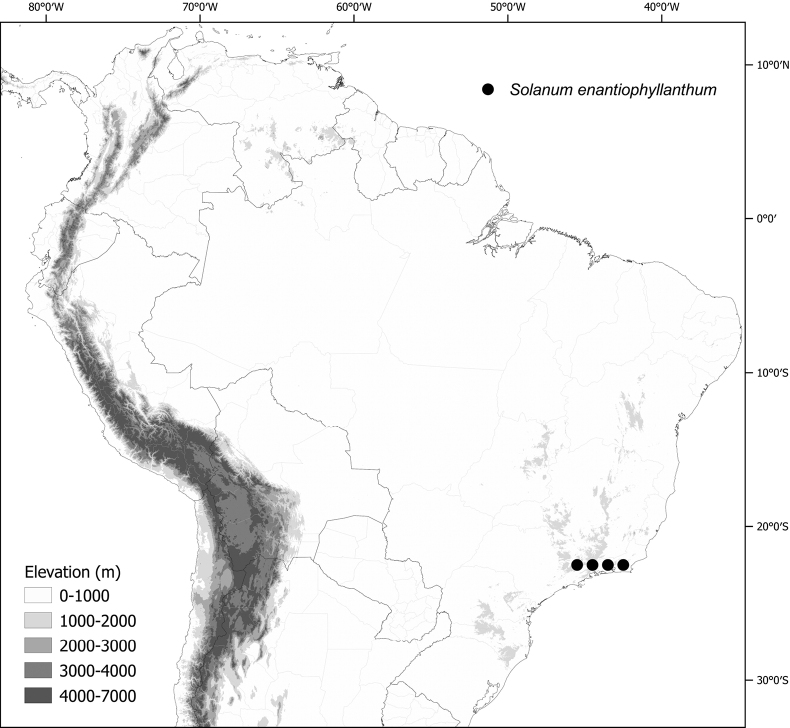
Distribution map of *Solanumenantiophyllanthum*.

**(Fig. 52).***Solanumenantiophyllanthum* is endemic to Brazil (States of Minas Gerais, Rio de Janeiro, São Paulo) with most collections coming from the Serra do Itatiaia.

##### Ecology and habitat.

*Solanumenantiophyllanthum* grows in open areas along roads and grassland edges in high elevation forests and grassy habitats; from (1,000) 2,000 to 2,600 m elevation.

##### Common names and uses.

Brazil. Rio de Janeiro: erva-moura (*Andrade 274*). No uses recorded.

##### Preliminary conservation status

**(IUCN 2022).** Vulnerable (VU – B2 a, b(iii), D2). EOO = 14,689 km^2^ [VU]; AOO = 92 km^2^ [EN]. *Solanumenantiophyllanthum* occurs in widely separated high elevation populations (< 5 locations) in the Serra do Mar of southeastern Brazil. Although it occurs in the protected area of Serra do Itatiaia at the junction of Rio de Janeiro, Minas Gerais and São Paulo States, we feel it merits some conservation concern because of its narrow range and the fact that it is not common where it occurs.

##### Discussion.

*Solanumenantiophyllanthum* is morphologically similar to *S.paucidens* with which it is broadly sympatric. *Solanumenantiophyllanthum* occurs within the larger range of *S.paucidens*, but at higher (usually above 2,000 m) elevations. The species can be distinguished by inflorescence morphology and anther length; *S.enantiophyllanthum* has flowers clustered at the tip of the (usually) unbranched inflorescence and anthers 4.5–6 mm long, while flowers of *S.paucidens* are spaced along the inflorescence axis and anthers are 2.5–3.5 mm long. The fruiting pedicels of *S.paucidens* are strongly curved at the base, making the infructescence appear somewhat secund, while those of *S.enantiophyllanthum* are merely deflexed.

The subumbellate inflorescences of large flowers and deflexed fruiting pedicels make *S.enantiophyllanthum* somewhat like *S.macrotonum* of northern South America, Central America and the Caribbean. The species differ in distribution, but also in flower size (corollas 1–2 cm in diameter, anthers 3–4 mm long in *S.macrotonum* versus corollas 1.9–2 cm in diameter, anthers 4.5–6 mm long in *S.enantiophyllanthum*), calyx lobe morphology (broadly deltate in *S.macrotonum* versus narrowly deltate in *S.enantiophyllanthum*) and in the number of stone cells in the berry (usually more than four in *S.macrotonum*, strictly four in *S.enantiophyllanthum*).

#### ﻿17. Solanum fiebrigii

Taxon classificationPlantaeSolanalesSolanaceae

Bitter, Repert. Spec. Nov. Regni Veg. 10: 556. 1912.

Figs 3A , 53 , 54

Solanum codonanthum Bitter, Repert. Spec. Nov. Regni Veg. 11: 235. 1912. Type. Argentina. Tucumán: Siambón, Jan 1874, *P.G. Lorentz & G. Hieronymus 818* (lectotype, designated by Barboza and Hunziker 2005, pg. 61: CORD [CORD00004182]).

##### Type.

Argentina. Salta: Santa Victoria, “Toldos prope Bermejo”, 20 Dec 1903, *K. Fiebrig 2421* (lectotype, designated by Barboza and Hunziker 2005, pg. 61: F [v0075528F, acc. # 621246, fragment of destroyed B duplicate]; isolectotype: B [destroyed, F. neg. 2712]).

**Figure 53.**
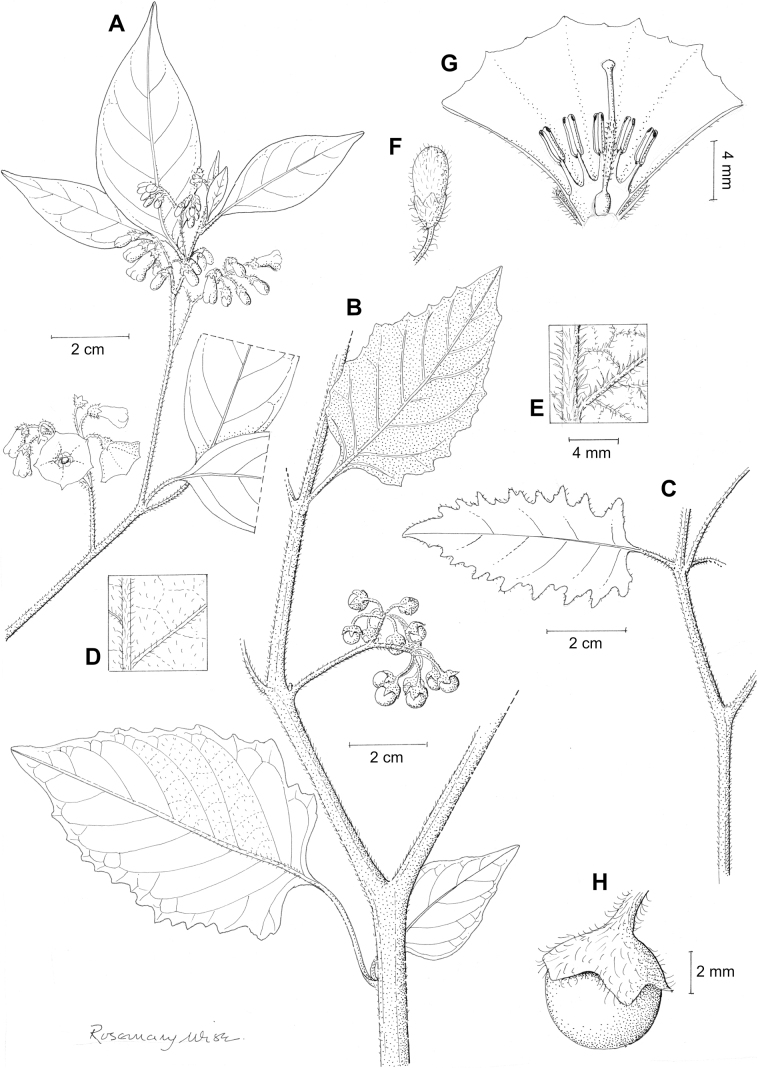
*Solanumfiebrigii***A** flowering branch **B** flower **C** variation in leaf shape and margin **D** detail of adaxial leaf surface **E** detail of abaxial leaf surface **F** flower bud **G** dissected flower **H** maturing fruit (**A, F, G***Wood 1810***B, D, E, H***Brooke 5851***C***Renvoize 3477*). Illustration by R. Wise.

##### Description.

Herbs or herbaceous shrubs, 0.5–2 m high, erect or the branches somewhat spreading. Stems terete to slightly angled with longitudinal ridges, densely to moderately pubescent with transparent glandular and eglandular 5–9-celled simple uniseriate trichomes 1–3 mm long, the terminal gland if present single-celled, glabrescent with age; new growth densely pubescent with glandular and eglandular 5–9-celled trichomes like those of the stems, viscid to the touch; bark of older stems pale greenish yellow. Sympodial units difoliate, the leaves not geminate. Leaves simple or shallowly toothed, the blades (4-) 6–15 (-16) cm long, (2.2-) 3–8.2 cm wide, ovate or narrowly elliptic, widest in the lower half or near the middle, membranous, concolorous; adaxial surfaces sparsely pubescent with transparent glandular and eglandular simple uniseriate trichomes 1–4 mm long, these 3–5-celled, spreading, denser along the midrib and principal veins; abaxial surfaces with similar pubescence on the lamina, but the trichomes much denser along the midrib and veins; principal veins 6–8 pairs, densely pubescent; base abruptly truncate then attenuate onto the petiole, usually somewhat oblique; margin serrulate to very shallowly and unevenly toothed, with 7 to 13 (-15) teeth ca. 2 mm long, these directed distally, the sinuses narrow; apex acuminate; petiole 0.5–2 (-4.5) cm long, mixed glandular and eglandular pubescent with transparent simple uniseriate trichomes like those of the stems. Inflorescences internodal, forked or further dichotomously branched, 2.5–6 cm long, with 10–20 flowers borne near the tips of the branches, moderately to densely pubescent with mixed glandular and eglandular transparent simple uniseriate trichomes like those of the stems; peduncle 1–2 cm long; pedicels 0.6–1 cm long, ca. 0.5 mm in diameter at the base, 1–1.3 mm in diameter at the apex, spreading at anthesis, pubescent with transparent glandular and eglandular simple uniseriate trichomes 0.5–1 mm long, articulated at the base; pedicel scars irregularly spaced 0.5–1.5 mm apart, enlarged and small projections from the axis, darker in herbarium specimens. Buds ovoid, the corolla strongly exserted from the calyx tube before anthesis. Flowers 5-merous, cosexual (hermaphroditic). Calyx tube 1.2–1.5 mm long, conical, the lobes (0.8-) 1.5–2 mm long, slightly unequal, deltate or occasionally triangular from elongate apices, pubescent with glandular and eglandular trichomes like those of the rest of the inflorescence, to 1.5 mm long and usually longer than those of the pedicels. Corolla 1.1–1.5 cm in diameter, campanulate, light purple or violet, lobed less than 1/8 of the way to the base, the lobes 1–1.5 mm long, 3–4 mm wide, reduced to 5 inconspicuous introrse tips in live plants, adaxially glabrous, abaxially sparsely papillate with minute transparent eglandular trichomes, these denser near the tips. Stamens equal; filament tube to 0.5 mm; free portion of the filaments 1.5–2 mm long, adaxially sparsely pubescent with tangled, transparent eglandular simple uniseriate trichomes; anthers 3–4(5) mm long, 1–1.6 mm wide, ellipsoidal to obellipsoidal and widest in the distal third, yellow, poricidal at the tips, the pores lengthening to slits with age. Ovary ovoid to conical, glabrous; style 7.5–10 mm long, straight, exserted beyond the anther cone, pubescent in the basal third with tangled eglandular trichomes, fully included in the campanulate corolla; stigma capitate to saddle-shaped and somewhat bilobed, the surfaces minutely papillate. Fruit a globose berry, 0.6–0.8 cm in diameter, green when ripe, the pericarp thin, matte, opaque, glabrous; fruiting pedicels 1–1.2 mm long, ca. 0.5 mm in diameter at the base, 1–1.3 mm in diameter at the apex, deflexed, not persistent; fruiting calyx not to very slightly accrescent, appressed to the berry, the tube 2–2.5 mm long, the lobes 2–2.5 mm long, somewhat glabrescent. Seeds 40–60 per berry, ca. 1.5 mm long, ca. 1 mm wide, flattened and teardrop shaped, pale tan, the surfaces minutely pitted, the testal cells pentagonal in outline. Stone cells 3–4(-6) per berry, 0.5–0.6 mm in diameter, scattered through the mesocarp, cream-coloured. Chromosome number: 2n = 24 (Chiarini et al. 2017; voucher *Chiarini 1227*).

**Figure 54.**
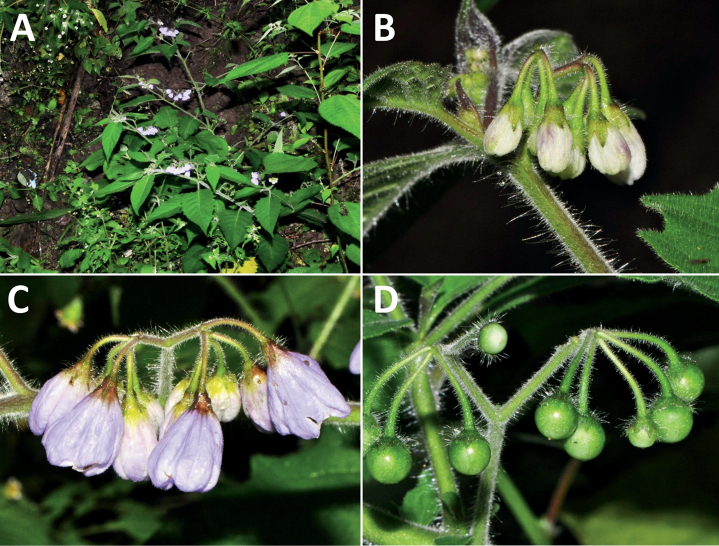
*Solanumfiebrigii***A** habit **B** inflorescences with buds **C** inflorescence with flowers at full anthesis **D** maturing fruits (**A–D***Barboza 3548*).

##### Distribution

**Figure 55.**
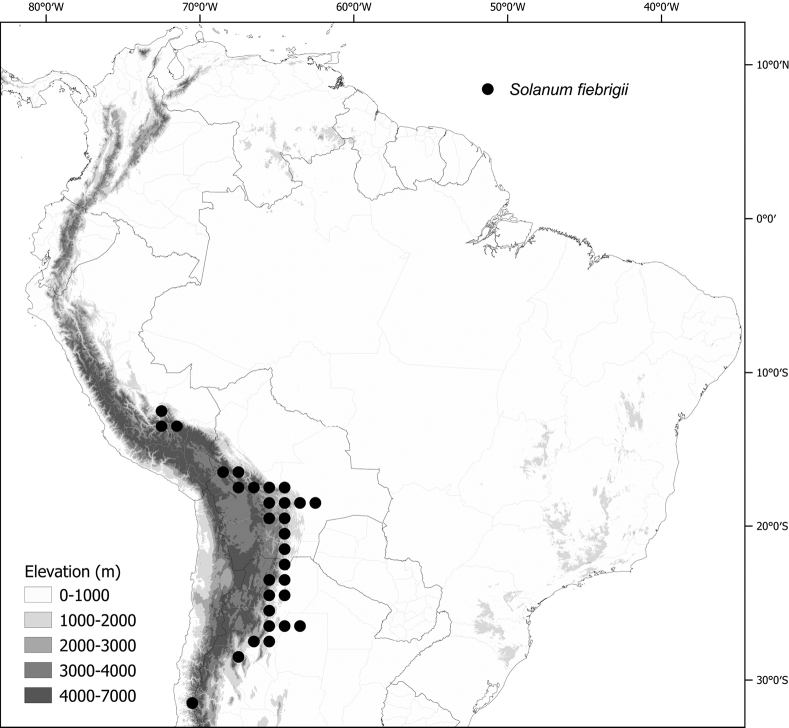
Distribution map of *Solanumfiebrigii*.

**(Fig. 55).***Solanumfiebrigii* occurs from northern Argentina (Provs. Jujuy, Salta, Tucumán, and Catamarca) to Bolivia (Depts. Cochabamba, La Paz, Santa Cruz, Tarija). A few collections have also been registered from southern Peru (Dept. Cusco).

##### Ecology and habitat.

*Solanumfiebrigii* is found in understory of montane and premontane forests (‘yungas’) with rich and moist soil and often occurs along streams and in other damp microhabitats; most commonly collected at middle to high elevations from 1,000 to 4,100 m, less often from 500 to 800 m elevation.

##### Common names and uses.

Bolivia. La Paz: chini chincha (*Girault B. s.n.*). No uses recorded.

##### Preliminary conservation status

**(IUCN 2022).** Least Concern [LC]. EOO = 1,079,092 km^2^ [LC]; AOO = 356 km^2^ [VU]. *Solanumfiebrigii* is widespread plant of disturbed areas; it occurs within protected areas in Argentina (e.g., Parque Nacional Calilegua).

##### Discussion.

*Solanumfiebrigii* along with the morphologically similar *S.sinuatiexcisum* were segregated into the small subsection Campanulisolanum Bitter (Bitter 1912b; Barboza and Hunziker 2005) based on the campanulate corolla shape and dense long pubescence that gives the plants a ‘shaggy’ appearance. Särkinen et al. (2015b) showed they are sister species but nested in the larger Black nightshade clade. *Solanumfiebrigii* differs from *S.sinuatiexcisum* in its forked (versus unbranched) inflorescence, its deltate to triangular calyx lobes that are shorter than or equal in length to the calyx tube (versus long-triangular calyx lobes that are always longer than the calyx tube).

Bitter (1914a) reported up to 15 stone cells per berry for *S.fiebrigii* (as *S.codonanthum*); none of the many berries we have examined has had this many stone cells. The vouchers he cited (*Lorentz & Hieronymus 181*, *899*) were in the Berlin Herbarium; duplicates we have examined have had either no berries or only four stone cells per berry.

#### ﻿18. Solanum fragile

Taxon classificationPlantaeSolanalesSolanaceae

Wedd., Chlor. And. 2: 105. 1859.

Figs 56 , 57

Solanum atriplicifolium Gillies ex Nees var. minus Gillies ex Nees, Nov. Act. Acad. Caes. Leop. 19, Suppl. 1: 387. 1843. Type. Peru. “Laguna de Titicaca, 12,400 ft.”, “In planitie circa Tacoram [Volcán Tacora], 14,000–17,000 ft., Apr” both syntypes collected by *F.J.F. Meyen s.n.* (no herbaria cited; possible original material: B, destroyed [F neg. 2598]). Peru. Puno: Prov. Puno, 19.5 km from Puno on rd to Tiquillaca, 3,982 m, 22 Mar 2012, *T. Särkinen, A. Mathews & P. Gonzáles 4058* (neotype, designated here: USM [acc. # 00264006]; isoneotype: BM [BM001114837]).
Solanum hauthalii Bitter, Bot. Jahrb. Syst. 50, Beibl. 111: 61. 1913. Type. Bolivia. La Paz: “La Paz-Palca-Illimani, 3,600–4,800 m”, *R. Hauthal 269* (syntype: B, destroyed [F. neg. 2714]); “in valle inferoire Chuquiaguillo [Chuquiguillo] prope La Paz ad orientem, 3,500–4,000 m”, *R. Hauthal 165* (no herbarium cited). Bolivia. La Paz: Pacajes, hills above the town of Comanche, 4,100 m, 4 Feb 1995, *E. Emschwiller EE-383* (neotype, designated here: LPB; isoneotypes: BH [000040588], F [v0472073F, acc. # 2286981; v0472074F, acc. # 2289672], NY [00852739]).

##### Type.

Peru. Tacna: “rochers humides de la Cordillère de Tacora”, 4,000 m, 1851, *H. Weddell s.n.* (lectotype, designated by Edmonds 1972, pg. 101 [as holotype]: P [P00335346]).

**Figure 56.**
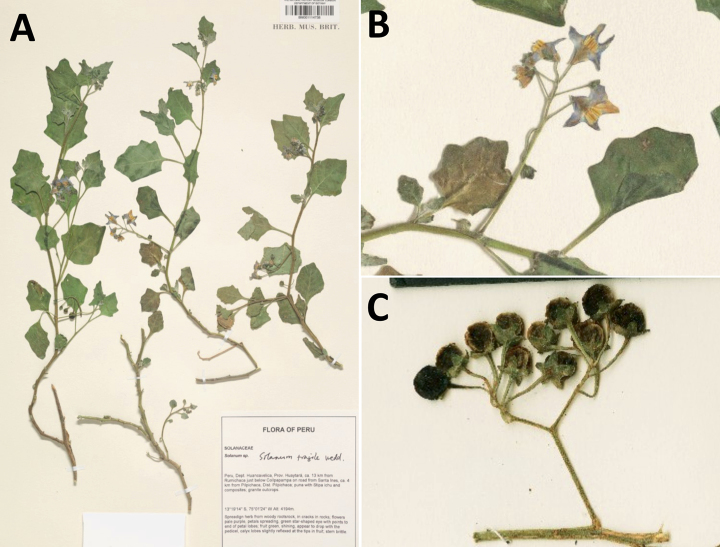
*Solanumfragile***A** habit **B** inflorescence with flowers **C** inflorescence with fruits (**A, B***Knapp et al. 10233* [BM001114738] **C***Beck 11788* [K000658368]). Reproduced with permission of the Trustees of the Natural History Museum and Royal Botanic Gardens, Kew.

##### Description.

Herb or shrublet from a woody base to 0.4 m high, the branches erect to spreading, brittle at the base, easily breaking from the woody rootstock. Stems slightly angled, densely pubescent with transparent to whitish cream mixed eglandular and glandular 2–3 celled simple uniseriate trichomes to 0.5 mm long, the gland if present single-celled; new growth densely pubescent with the same transparent to whitish cream mixed eglandular and glandular 2–3 celled simple uniseriate trichomes to 0.5 mm long; bark of older stems pale yellowish brown, glabrescent. Sympodial units difoliate, the leaves not geminate. Leaves simple and shallowly toothed, the blades 1.2–7 cm long, 0.7–4.5 cm wide, ovate to rhomboid, widest in the lower half, membranous to somewhat fleshy and rubbery, discolorous; adaxial surfaces sparsely to moderately and evenly pubescent with stiff, patent, transparent glandular 2–3-celled simple uniseriate trichomes to 0.5 mm long, these to 1 mm long on the veins; abaxial surfaces similarly glandular-pubescent, the pubescence slightly denser, but not markedly so; principal veins 4–5 pairs, drying dark brown to blackish brown, more densely pubescent than the lamina especially abaxially; base truncate and abruptly attenuate onto the petiole; margins shallowly toothed, the teeth 1–2 mm long, 2–4 mm wide, with rounded tips, the sinuses reaching less than 1/8 of the way to the midrib; apex acute to rounded; petioles 0.2–0.4 cm long, the winged portion from the decurrent leaf base very narrow, densely pubescent with transparent to whitish cream mixed eglandular and glandular 2–3 celled simple uniseriate trichomes to 0.5 mm long. Inflorescences internodal, forked or less commonly several times branched, (1.5)3–5 cm long, with (3)9–12 flowers clustered in the distal parts of the branches, densely pubescent with transparent to whitish cream mixed eglandular and glandular 2–3 celled simple uniseriate trichomes to 0.5 mm long like the stems; peduncle 1–2 cm long; pedicels 0.7–1 cm long, ca. 0.75 mm in diameter at the base and apex, not markedly tapering, spreading at anthesis, densely pubescent with transparent to whitish cream mixed eglandular and glandular 2–3 celled simple uniseriate trichomes to 0.5 mm long, articulated at the base; pedicel scars irregularly spaced 0.5–1(5) mm apart. Buds globose, the corolla halfway exserted from the calyx before anthesis. Flowers 5-merous, cosexual (hermaphroditic). Calyx tube 1–2 mm long, strongly cup-shaped and abruptly narrowing to the pedicel apex, the lobes 2–3 mm long, ca. 1 mm wide, triangular with blunt tips, densely pubescent with transparent to whitish cream mixed eglandular and glandular 2–3 celled simple uniseriate trichomes to 0.5 mm long and glandular papillae. Corolla 1.5–1.6 cm in diameter, white or violet, with a green eye extending along the lobe midveins, stellate, lobed ca. halfway to the base, the lobes 5–6 mm long, 3–4 mm wide, broadly deltate, spreading at anthesis, adaxially glabrous, abaxially densely puberulent with white eglandular simple uniseriate trichomes to 0.4 mm long, densely papillate on tips and margins. Stamens equal; filament tube minute; free portion of the filaments ca. 0.5 mm, sparsely pubescent with tangled transparent simple uniseriate trichomes adaxially; anthers 2.5–3 mm long, ca. 1.5 mm wide, plumply ellipsoid, yellow, poricidal at the tips, the pores lengthening to slits with age. Ovary glabrous, conical; style ca. 9 mm long (*Knapp et al. 10259* with styles 4 mm long), strongly curved in bud, straight, long-exserted from the anther cone, glabrous; stigma large, globose and capitate, the surface minutely papillate, bright green in live plants. Fruit a globose berry, 0.5–0.8 cm in diameter, green when ripe, the pericarp glabrous, thin or somewhat stiff and leathery, shiny, opaque, glabrous; fruiting pedicels 0.8–1.2 cm long, ca. 0.75 mm in diameter at the base, ca. 1 mm in diameter at the apex, not markedly woody, deflexed, not persistent or occasionally remaining on the inflorescence axis; fruiting calyx somewhat enlarged, the tube to 2 mm long, the lobes to 3 mm long, spreading and the tips slightly reflexed. Seeds ca. 30 per berry, ca. 2 mm long, ca. 1.5 mm wide, flattened to slightly ovoid reniform, straw-coloured or yellowish brown, the surfaces minutely pitted, the testal cells sinuate in outline. Stone cells absent. Chromosome number: reported as 2n = 48 (Edmonds 1972, 1977, vouchers *Iltis et al. 481b*, *Hawkes et al. 4110*, *Gade s.n.*; none found for verification).

**Figure 57.**
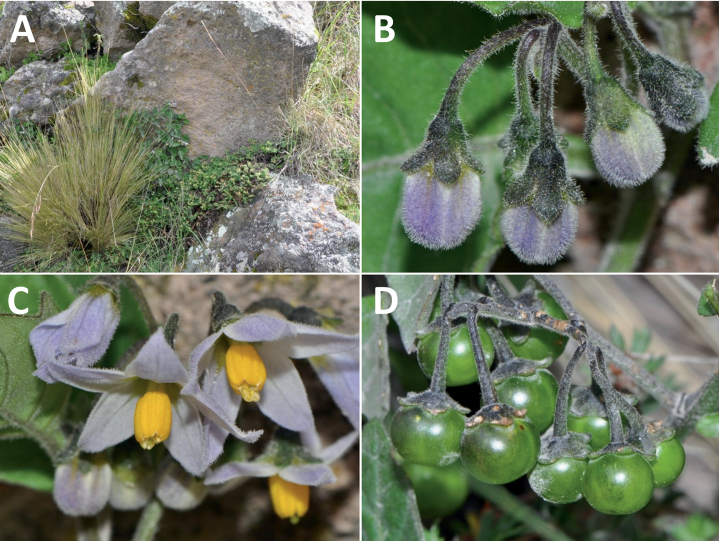
*Solanumfragile***A** habit **B** inflorescence with buds **C** flowers at full anthesis **D** maturing fruits (**A–D***Knapp et al. 10259*). Photos by S. Knapp.

##### Distribution

**Figure 58.**
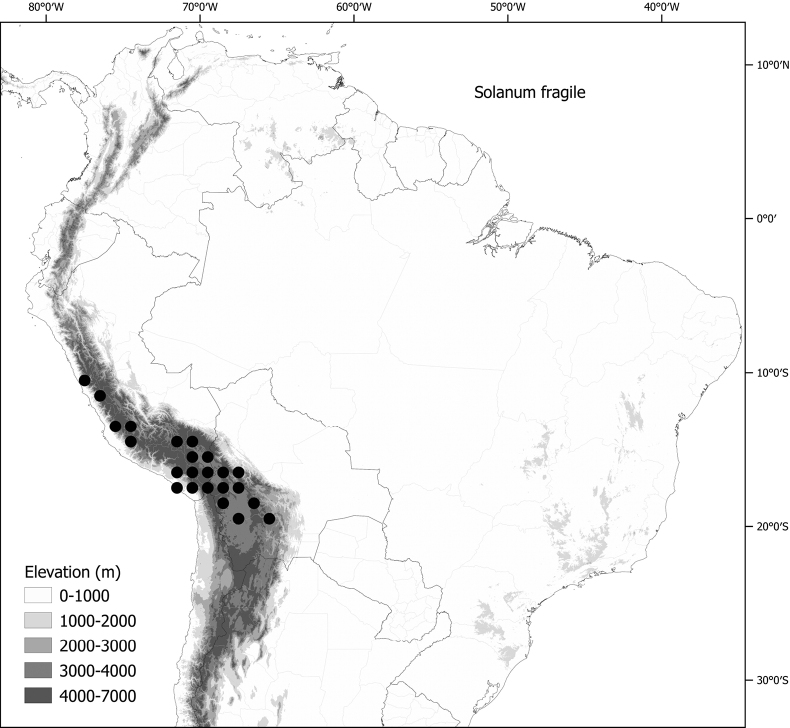
Distribution map of *Solanumfragile*.

**(Fig. 58).***Solanumfragile* is an Andean species, occurring from Peru (Depts. Ancash, Arequipa, Ayacucho, Cusco, Huancavelica, Lima, Moquegua, Puno, Tacna) to Bolivia (Depts. La Paz, Oruro, Potosí) and northern Chile (Regíon I [Tarapacá]).

##### Ecology and habitat.

*Solanumfragile* grows in grassy puna vegetation among rocks and at the bases of cliffs, from 2,165 to 4,500 m elevation.

##### Common names and uses.

Peru. Ancash: japchilla (*Cerrate & Ferreyra 7015*). No uses recorded.

##### Preliminary conservation status

**(IUCN 2022).** Least Concern [LC]. EOO = 338,395 km^2^ [LC]; AOO = 176 km^2^ [EN]. *Solanumfragile* is not common where it occurs but has a relatively wide distribution and does not appear to be habitat specific. It is found in the region of the Lake Titicaca Reserve in Peru and Bolivia but has not been specifically recorded within a protected area.

##### Discussion.

*Solanumfragile* is morphologically similar to the sympatric *S.grandidentatum*. Both are glandular-pubescent plants with incised, shallowly lobed leaves and green berries. *Solanumfragile* differs from *S.grandidentatum* in its possession of a woody rootstock with brittle stems (herbarium specimens are often only of the single stems that break off); *S.grandidentatum* is a shrubby plant with conspicuous aboveground branching. In live plants in the field, leaves of *S.fragile*, although glandular-pubescent, are odourless, but those of *S.grandidentatum* have a strong odour; leaf bases of *S.fragile* are truncate, while those of *S.grandidentatum* are more attenuate. Although the stamens of these two species are similar, the ratio of anthers to filaments is markedly different; *S.fragile* has anthers 2.5–3 mm long and filaments ca. 0.5 mm long, while *S.grandidentatum* has anthers 2–2.5 mm long and filaments 1–1.2 mm long.

Molecular sequence data suggest the two species are not closely related (Särkinen et al. 2015b; Gagnon et al. 2022), but this result could be affected by polyploidy. *Solanumgrandidentatum* has a vouchered chromosome count of 2n = 48 (tetraploid) and *S.fragile* is also recorded as being tetraploid (2n = 48, see above) but we have been unable to find the vouchers for this information; it needs reconfirmation. Both species are part of weakly supported groups (polytomies), but different ones (see appendix S11 in Gagnon et al. 2022).

In describing S.atriplicifoliumvar.minus, Nees von Esenbeck (1843) expressed some doubt as to its identity (“Var. β alieni quid prae se fert, et dubito, an huiis specie, an potius Solani furcati, nanam [nanum] prolem esse dicam” – [Var. β is strange and may be dwarf or perhaps *Solanumfurcatum*]). No herbaria were cited in the protologue, but Franz Meyen’s herbarium from his South American travels was held in B and destroyed. A sheet in B photographed by J.F. Macbride (F. neg. 2598) is annotated by Bitter with an observation that it was the sheet referred to by Dunal (1852) as S.atriplicifoliumvar.minus but clearly was not that species (“Dies ist die Pflanze vom Originalfundort des *S.atriplicifolium* Gill. Var. β minus bei Dunal in DC. N. 78 haupt offtenbar nicht mit *S.atriplicifolium* zusammen” – [This is the plant that is the original of S.atriplicifolium Gill. var. β minus in Dunal in DC. n. 78 and is obviously not associated with S.atriplicifolium]). To date we have found no duplicates of either Meyen collection cited in the protologue, but have not comprehensively searched all the herbaria where duplicates might be found. We select here a recent collection for southern Peru in the Lake Titicaca area (*Särkinen et al. 2058*, USM acc. # 00264006) as a neotype.

Bitter (1913) cited two collections (*Hauthal 165, 269*) in the protologue of *S.hauthalii*, citing “herb. Berol.” as the location only for *Hauthal 269* and no herbarium for *Hauthal 165*. The specimen of *Hauthal 269* was photographed in Berlin (F neg. 2714) and corresponds to *S.fragile*; we have found no duplicates of either of these collections where Rodolfo Hauthal’s specimens are known to be deposited (e.g., GOET, NY *fide*Funk and Mori 1989). Although we have seen no recent collections from the trajectory between La Paz and Nevado Illimani we have selected a recent collection from a similar elevation with many duplicates as the neotype for this name (*Emschwiller EE-383*, neotype at LPB).

#### ﻿19. Solanum furcatum

Taxon classificationPlantaeSolanalesSolanaceae

Dunal, Encycl. [J. Lamarck & al.] Suppl. 3: 750. 1814.

Figs 59 , 60

Solanum deltoideum Colla, Herb. Pedem. 4: 273. 1835. Type. Cultivated in Italy at “h. Ripul:” [Hortus Ripulensis], the seeds originally sent by C. Bertero from Chile [“Chili Quillota”] (no specimens cited; lectotype, designated by Särkinen et al. 2018, pg. 73: TO [herb. Colla]).
Solanum furcatum Dunal var. glabrum G.Don, Gen. Hist. 4: 412. 1837. Type. Cultivated “Native of Peru” (no specimens cited; no original material located).
Solanum furcatum Dunal var. pilosum G.Don, Gen. Hist. 4: 412. 1837. Type. Cultivated “Native of Peru” (no specimens cited; no original material located).
Solanum furcatum Dunal var. acutidentatum Nees, Nov. Act. Acad. Caes. Leop. 19, Suppl. 1: 386. 1843, as “*acutedentatum*”. Type. “Chile ad Valparaiso, Februario; Peruvia in planitie circa Tacoram [Volcán Tacora], alt. 14,000–17,000’ [feet], Aprili” both syntypes collected by *F.J.F. Meyen s.n.* (no specimens cited; no original material located). Chile. Région V (Valparaiso): Prov. Valparaiso, Dunas de Concón, 22 Dec 2008, *M. Gardner & S Knees 8356* (neotype, designated here: E [E00282600]; isoneotype: BM [BM001120031], CONC [?], SGO [?]).
Solanum furcatum Dunal var. obtusidentatum Nees, Nov. Act. Acad. Caes. Leop. 19, Suppl. 1: 386. 1843, as “*obtusedentatum*”. Type. “Chile. Prov. de San Fernando in Llano del Rio Tinguiririca, 3,000’ [feet] alt., Martio”; Peruvia ad Arequipam, Aprili” both syntypes collected by *F.J.F. Meyen s.n.* (no specimens cited; no original material located). Chile. Région VI (O’Higgins): Prov. Colchagua, San Fernando, s.d., *R.A. Philippi s.n.* (neotype, designated here: G [G00443353]).
Witheringia furcata (Dunal) J.Rémy, Fl. Chil. [Gay] 5: 67. 1849. Type. Based on Solanumfurcatum Dunal.
Solanum pterocaulum Dunal var. dichotimiflorum Dunal, Prodr. [A. P. de Candolle] 13(1): 52. 1852, as ‘*opterocaulon*’. Type. Cultivated in France at Montpellier “Solanum speciosum hort. botan” (no specimens cited, described from living plants “v.v. hort. Monsp.”; neotype, designated by Särkinen et al. 2018, pg. 73: MPU [MPU310703]).
Solanum crenatodentatum Dunal, Prodr. [A. P. de Candolle] 13(1): 54. 1852. Type. Chile. Région VI (O’Higgins): Colchagua, San Fernando, “in selibus chilensibus San Fernando”, Mar 1831, *C. Gay 2* (lectotype, designated by D’Arcy 1974a, pg. 738: P [P00337274]).
Solanum rancaguense Dunal, Prodr. [A. P. de Candolle] 13(1): 150. 1852. Type. Chile. Région VI (O’Higgins): Rancagua, May-Oct 1828, *C. Bertero 633* (lectotype, designated by Edmonds 1972, pg. 107 [as holotype], second step designated by Särkinen et al. 2018, pg. 73: P [P00384088]; isolectotypes: BM [BM000617677], G [G00144259], M [M-0171928], MO [MO-503700], NY [NY00743695], P [P00384089], P [P00384090], P [P00384091], P [P00384092], P [P00482266], W [acc. # 1889-0283789]).
Solanum bridgesii Phil., Linnaea 33: 203. 1864. Type. Chile. Región V (Valparaíso): Panquegue, *R.A. Philippi s.n.* (lectotype, designated by Särkinen et al. 2018, pg. 74: SGO [SGO000004549]).
Solanum coxii Phil., Linnaea 33: 200. 1864. Type. Chile. Región X (Los Lagos): Todos los Santos, 1862, *G. Cox 38* (lectotype, designated by Särkinen et al. 2018, pg. 74: SGO [SGO000004555]; isolectotype: W [acc. # 1903-0010246]).
Solanum rancaguinum Phil., Anales Univ. Chile 43: 523. 1873. Type. Chile. Región VI (O’Higgins): Rancagua, Mar 1828, *C. Bertero s.n.* (lectotype, designated by Särkinen et al. 2018, pg. 74: SGO [SGO000004594]).
Solanum caudiculatum Phil., Anales Univ. Chile 91: 12. 1895. Type. Chile. Región VIII (Bío-Bío): Prov. Ñuble, Coigüeco, *F. Puga s.n.* (no original material located, not at SGO).
Solanum subandinum Phil., Anales Univ. Chile 91: 13. 1895, nom. illeg., not Solanumsubandinum F.Meigen (1893). Type. Chile. Región XIII (Metropolitana): Santiago, Las Condes, *R.A. Philippi s.n.* (lectotype, designated by Särkinen et al. 2018, pg. 74: SGO [SGO000004600 = F neg. 2745]).
Solanum ocellatum Phil., Anales Univ. Chile 91: 14. 1895. Type. Chile. Región XIII (Metropolitana): Prope Colina, *F. Philippi s.n.* (lectotype, designated by Särkinen et al. 2018, pg. 74: SGO [SGO000004582]; isotypes: SGO [SGO000004581], W [acc. # 1903-0010230]).
Solanum nigrum L. var. crentatodentatum (Dunal) O.E.Schulz, Symb. Antill. (Urban) 6: 160. 1909. Type. Based on Solanumcrenatodentatum Dunal.
Solanum bridgesii Phil. var. ocellatum (Phil.) Witasek ex Reiche, Anales Univ. Chile 124: 460. 1909. Type. Based on Solanumocellatum Phil.
Solanum andinum Reiche, Fl. Chile 5: 346. 1910. Type. Based on (replacement name for) Solanumsubandinum Phil.
Solanum tredecimgranum Bitter, Repert. Spec. Nov. Regni Veg. 11: 6. 1912. Type. Chile. Región V (Valparaíso): Valparaíso, 17 Aug 1895, *O. Buchtien s.n.* (lectotype, designated by Barboza et al. 2013, pg. 246: US [US00432692, acc. # 139293]; isolectotypes: HBG [HBG-511497], US [US00681745, acc. # 139294]).
Solanum robinsonianum Bitter, Repert. Spec. Nov. Regni Veg. 11: 7. 1912. Type. Chile. Región V (Valparaíso): Juan Fernández Island, *R.A. Philippi 742* (holotype: B, destroyed [F neg. 2743]; lectotype, designated by Särkinen et al. 2018, pg. 74: W [acc. # 0001347]).
Solanum masafueranum Bitter & Skottsb., Nat. Hist. Juan Fernandez & Easter Island 2: 167, pl. 14. 1922. Type. Chile. Región V (Valparaíso): Juan Fernández Islands, Masafuera [Isla Alejandro Selkirk], Las Chozas, 715 m, 3 Mar 1917 [20 Feb 1917 on label], *C. Skottsberg & I. Skottsberg 363* (lectotype, designated by Särkinen et al. 2018, pg. 74: S [acc. # 04-2947]; isolectotypes: BM [BM000617676], LD [1643307], K [K000585692], NY [00172084], GOET [GOET003548], GB [GB0048742], P [P00337092], UPS [acc. # 104031]).
Solanum spretum C.V.Morton & L.B.Sm., Revis. Argentine Sp. Solanum 132. 1976. Type. Argentina. Río Negro: Bariloche, 19 Mar 1939, *A.L. Cabrera 5024* (holotype: GH [00077764]; isotypes F [v0073411F, acc. # 1007493], LP [LP006791]).

##### Type.

Peru? [more likely Chile]. “Cette plante croît au Perou”, *J. Dombey [343*] (lectotype, first step designated by Edmonds 1972, pg. 107 [as holotype], second step designated by Barboza et al. 2013, pg. 246: P [P00335357]; isolectotypes: B, destroyed [F neg. 2729]; CORD [CORD00006928], F [v0043232F, acc. # 976864], G [G00359946], G-DC [G00144483], P [P00335358]).

**Figure 59.**
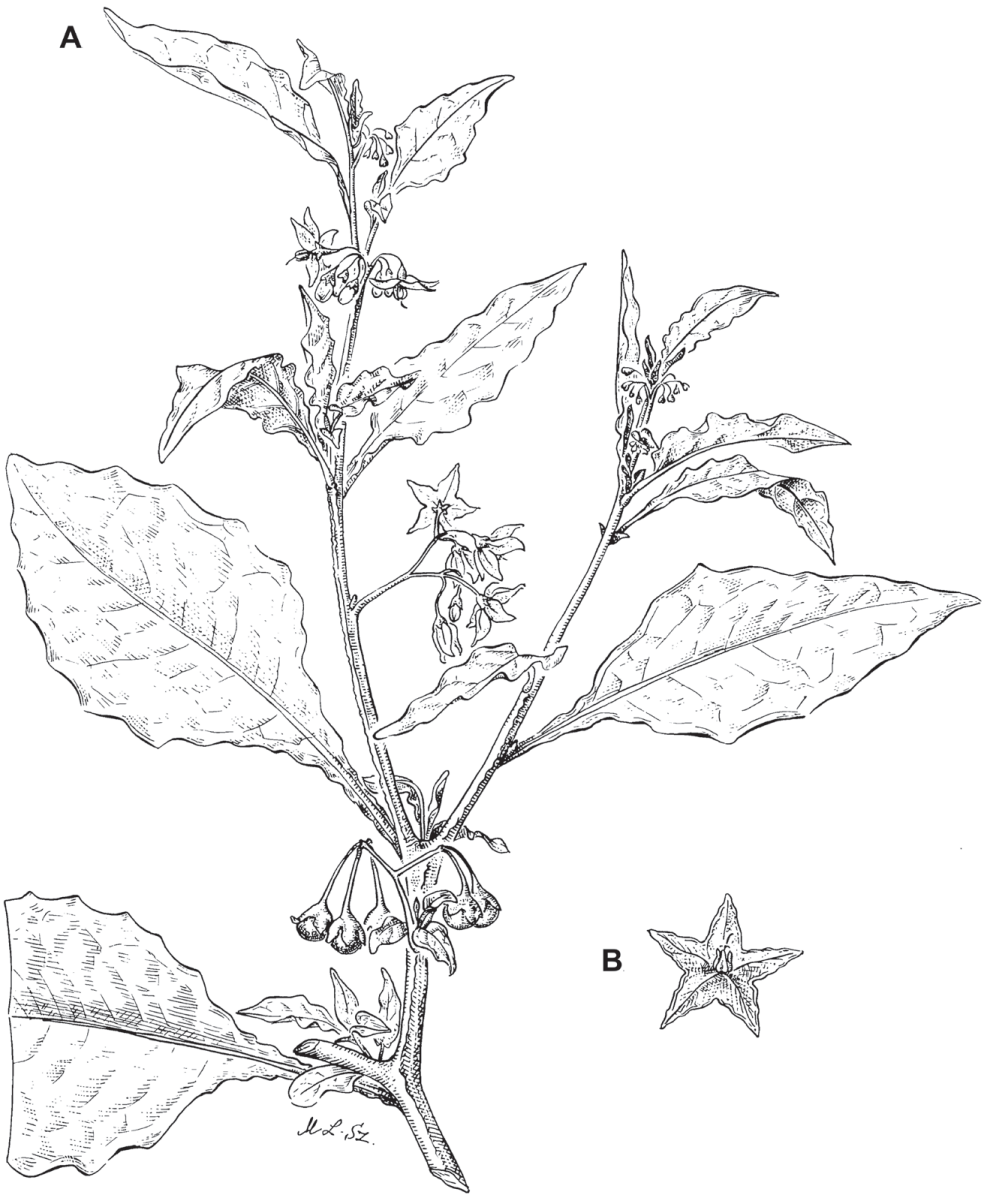
*Solanumfurcatum***A** habit **B** flower (**A, B***Anonymous s.n.*, grown from seed sent by J. Edmonds, originally from California [ADW 42421]). Illustration by M.L. Szent-Ivany, first published in Symon (1981) and previously published in Särkinen et al. (2018). Courtesy of the Board of the Botanic Gardens and State Herbarium (Adelaide, South Australia), reproduced with permission.

##### Description.

Annual or subwoody perennial herbs to 1 m high, erect to lax, sprawling to ca. 2 m across. Stems terete or ridged, green to purple tinged, not markedly hollow, sparsely pubescent with simple, uniseriate 1–5-celled eglandular trichomes 0.1–0.5 mm long; new growth sparsely to densely pubescent with similar simple, uniseriate 1–5-celled eglandular trichomes; older stems sparsely pubescent to glabrescent, pale yellowish brown. Sympodial units difoliate, the leaves not geminate. Leaves simple and shallowly sinuate, the blades (1.5–)4–8(–12) cm long, (0.6–)2.2–4.6(–6.5) cm wide, ovate to rhomboidal, widest in the lower half to third, membranous, discolorous; adaxial surface sparsely pubescent with simple, uniseriate trichomes like those on stem, these evenly spread along lamina and veins; abaxial surface more densely pubescent; major veins 4–6 pairs; base cuneate to acute, the two sides slightly unequal, decurrent on the petiole; margins entire or sinuate-dentate, this more pronounced in basal part of the leaf; apex acute; petioles 1–3.5 cm long, sparsely pubescent with simple uniseriate trichomes like those on stem. Inflorescences internodal, forked or more rarely unbranched, (1–)1.5–3(–4) cm long, with 6–14 flowers clustered at the tips (sub-umbelliform) or evenly spaced along the axis, sparsely pubescent with simple uniseriate trichomes like those on stem; peduncle (1–)1.5–2 cm long; pedicels 4–7.5 mm long, 0.2–0.3 mm in diameter at the base and 0.3–0.4 mm in diameter at the apex, straight and spreading, articulated at the base; pedicel scars spaced ca. 0.2–2.5 mm apart. Buds subglobose, the corolla exserted 1/3–1/2 from the calyx tube before anthesis. Flowers 5-merous, cosexual (hermaphroditic). Calyx tube 2–3 mm long, conical, the lobes 0.8–1.5 mm long, 0.6–1 mm wide, rectangular to narrowly obovate with obtuse to short-acute apices, pubescent with simple uniseriate trichomes like those on stem but shorter. Corolla 1.2–2 cm in diameter, white to lilac with a green or yellow-green central portion near the base, this sometimes purplish near the lobe midvein, stellate, lobed 1/3–1/2 of the way to the base, the lobes 5.5–7 mm long, 2.8–5.5 mm wide, strongly reflexed at anthesis, later spreading, densely pubescent abaxially with 1–4-celled simple uniseriate trichomes, especially along the margins and apex, these shorter than the trichomes of the stems and leaves. Stamens equal; filament tube minute; free portion of the filaments 0.9–1.6 (2) mm long, adaxially pubescent with tangled uniseriate 4–6-celled simple trichomes; anthers 2.3–3.3(-3.6) mm long, 0.8–1 mm wide, ellipsoid, yellow, poricidal at the tips, the pores lengthening to slits with age. Ovary globose, glabrous; style 6–6.5 mm long, straight or somewhat curved, long-exserted beyond the anther cone, densely pubescent with 2–3-celled simple uniseriate trichomes in the lower 1/2–2/3; stigma capitate, minutely papillate, yellow or green in live plants. Fruit a globose berry, 0.6–0.9 cm in diameter, dull green to purple at maturity, the pericarp matte, opaque, glabrous; fruiting pedicels 0.7–1.2 cm long, 0.2–0.4 mm in diameter at the base, 0.5–1 mm in diameter at the apex, strongly deflexed, not persistent; fruiting calyx not accrescent, the tube 1–2 mm long, the lobes 1.5–2.5 mm long, appressed against the berry. Seeds 30–40 per berry, 1.8–2 mm long, 1.4–1.5 mm wide, flattened and teardrop shaped with a subapical hilum, yellow-brown, the surface minutely pitted, the testal cells pentagonal in outline. Stone cells 6–14 per berry, 0.8–1 mm in diameter, scattered throughout the berry, cream-coloured. Chromosome number: 2n = 72 (Stebbins and Paddock 1949, from Californian plants no voucher cited; Edmonds 1977, voucher from Chile *Hjerting & Rahn 552*, not found or verified).

**Figure 60.**
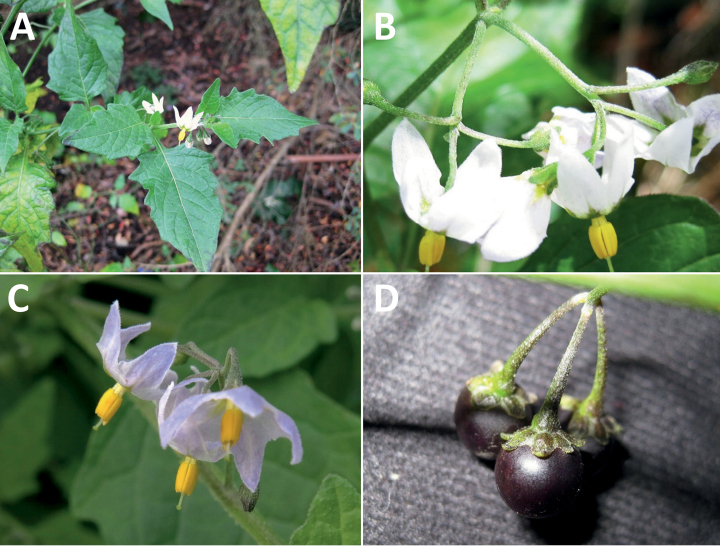
*Solanumfurcatum***A** flowering branch **B** inflorescence with flowers at full anthesis **C** developing fruits **D** mature fruits (**A, B, D***Knapp s.n.* Golden Gate Park **C***Gardner & Knees 8322*). Photos by S. Knapp and M. Gardner. **A, B, D** previously published in Särkinen et al. (2018) and Knapp et al. (2019).

##### Distribution

**Figure 61.**
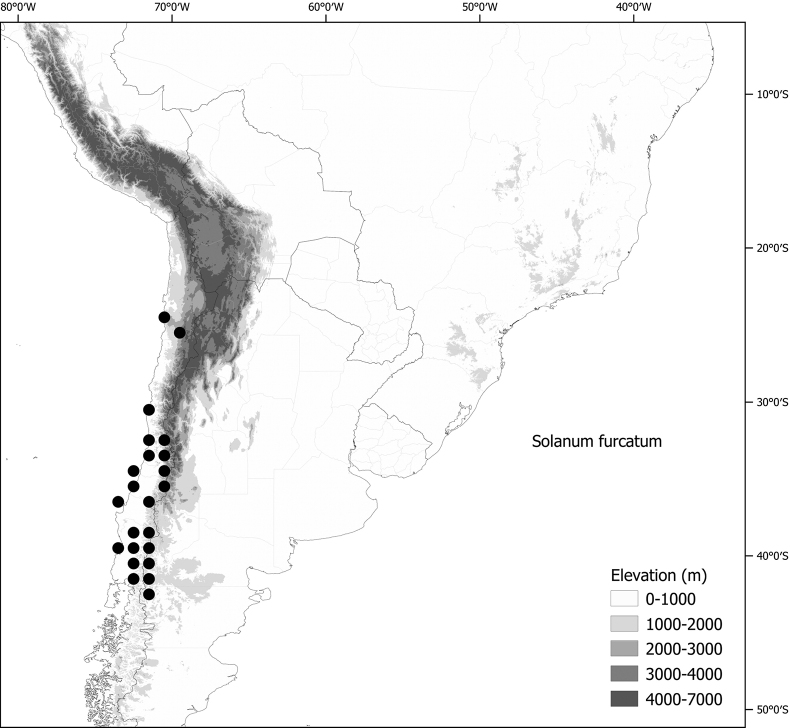
Distribution map of *Solanumfurcatum* in South America. For adventive distribution in North America and Australia, see Knapp et al. (2019) and Särkinen et al. (2018) respectively.

**(Fig. 61).***Solanumfurcatum* is native to temperate southern Chile (including the Juan Fernández Islands; Regions I [Tarapacá], II [Antofagasta], IV [Coquimbo], V [Valparaiso], VI [O’Higgins], VII [Maule], VIII [Bío-Bío], IX [Araucania], X [Los Lagos], XIII [Metropolitana], XIV [Los Rios]) and adjacent southern Andean Argentina (Provs. Chubut, Neuquén, Río Negro). It is locally introduced and naturalised along the west coast of the United States of America, Australia and New Zealand (see Särkinen et al. 2018; Knapp et al. 2019).

##### Ecology and habitat.

In its native range *S.furcatum* is a plant of disturbed areas and forest edges in *Nothofagus* (Nothofagaceae) forests; from near sea level in the more southern part of its range to 2,300 m elevation.

##### Common names and uses.

Chile. Región V (Valparaiso): yerba mora (*Philippi s.n.*); Región VI (O’Higgins): yerba mora (*Bertero 633*); Región VIII (Bío-Bío): llaqui (*Junge 2611*). No uses recorded.

##### Preliminary conservation status

**(IUCN 2022).** Least Concern [LC]. EOO = 342,557 km^2^ [LC]; AOO = 168 km^2^ [EN]; calculated using South American distribution only and excluding the Juan Fernández Islands. *Solanumfurcatum* has a relatively large range in Chile and adjacent Argentina and is a plant of disturbed areas. It occurs within several Chilean protected areas and in Parque Nacional Bariloche (Argentina). The populations on the Juan Fernández Islands are within the Juan Fernández Archipelago National Park (Chile).

##### Discussion.

*Solanumfurcatum* is similar to *S.arequipense*, an endemic species found to the north in western Peru. The two taxa have long been confused (e.g., Nees von Esenbeck 1843) and in our treatment of *S.furcatum* in its introduced range outside of South America (Särkinen et al. 2018), we considered them to be conspecific (e.g., Knapp et al. 2019: 67 illustrated *S.furcatum* with a drawing of *S.arequipense*). We recognise these two species here based on the subtle morphological differences that correspond to geographically distinct distributions. They are supported as evolutionarily distinct based on a molecular phylogeny that shows samples of the two species in distinct well supported clades (Gagnon et al. 2022). Both taxa exhibit the combination of a long-exserted style with the exserted portion equal to or longer than the length of the anthers, shallowly lobed corolla, calyx lobes separated by a paler sinus, forked to unbranched inflorescences (rarely several times branching), and usually large, lush leaves. The inflorescence branches of *S.furcatum* are less strongly divergent than those of *S.arequipense*, and the berries of *S.furcatum* always have more than six stone cells, while those of *S.arequipense* have only two or stone cells are absent. On an annotation on the now-destroyed duplicate of Dombey’s type gathering of *S.furcatum* in B (F neg. 2723) Georg Bitter noted the presence of eight stone cells in the berries. Material of *S.furcatum* from Juan Fernández Islands differs from that collected in mainland Chile in having flowers with styles barely exserted from the anther tube. This could indicate flowers that are autogamous, a reproductive adaptation commonly associated with island life (Schueller 2004).

*Solanumpentlandii* also has similar globose buds and exserted styles but has much shorter anthers (less than 2 mm versus 2.5–3 mm in *S.furcatum*) and shiny green berries that lack stone cells. *Solanumpentlandii* occurs at high elevations in disturbed, nitrogen-rich areas in Peru and Bolivia and is not sympatric with *S.furcatum*.

Details of typification for the synonyms of *S.furcatum* can be found in Särkinen et al. (2018). In earlier works (Särkinen et al. 2018; Knapp et al. 2019) we did not lectotypify the four varieties of of *S.furcatum* described by Nees von Esenbeck (1843) from the collections of Franz Meyen’s trip around the world (1831–32). Franz Meyen’s herbarium from his South American travels was held in B and destroyed, and we have found no duplicates of these collections (see also *S.arequipense*) nor were any specimens photographed by J.F. Macbride as is the case for many other species. Nees von Esenbeck’s four taxa were distinguished based on leaf shape differences, notoriously difficult in the Morelloid clade. He (Nees von Esenbeck 1843) cited two collections each for three varieties, mixing plants from the distributions of *S.arequipense* and *S.furcatum* (see discussion of var.subdentatum and var. subintegerrimum under *S.arequipense*). Var. acutedentatum was based on collections from Valparaiso and from “planitie circa Tacoram, alt. 14000–17000[ft]” [Volcán Tacora on the Chile/Peru border]; var.obtusedentatum on collections from San Fernando in central Chile and Arequipa in Peru. We have chosen to neotypify these two varieties with specimens from areas near the cited collections from Chile, in order to link the varietal names with *S.furcatum*. Searches in SGO and CONC failed to reveal duplicates of either *Philippi s.n.* from San Fernando or *Gardner & Knees 8356*, so we have reluctantly used as neotypes for var. obtusedentatum a collection we have seen in G and for var. acutedentatum the E duplicate we have seen of *Gardner & Knees 8356*, rather than duplicates in Chilean herbaria.

Specimens in Paris used by J. Rémy (1849) to describe *Witheringiarubra* J.Rémy are of plants of *S.furcatum* (see P00335356). He cited *S.rubrum* Mill. in synonymy and was clearly making a new combination based on that name. The type of the name *Witheringiarubra* is the type of *S.rubrum* Mill., itself a synonym of *S.villosum* (BM000942563 see Särkinen et al. 2018) although Särkinen et al. (2018) failed to site it in the synonym of *S.villosum*. *Witheringiarubra* (Mill.) J.Rémy is not a synonym of *S.furcatum*.

#### ﻿20. Solanum gilioides

Taxon classificationPlantaeSolanalesSolanaceae

Rusby, Mem. Torrey Bot. Club 4: 228. 1895.

Figs 62 , 63

Solanum nicandricalyx Cabrera, Bol. Soc. Argent. Bot. 13(4): 326. 1971. Type. Argentina. Jujuy: Dpto. Tilcara: Falda Grande, Cerro de Guairahuasi, *A. Cabrera & P. Hernández 14026* (holotype: LP; isotype: CORD [CORD00012842, fragment of LP holotype]).

##### Type.

Bolivia. Cochabamba: “vic. Cochabamba”, 1891, *M. Bang 938* (no herbaria cited; lectotype, designated here: NY [00172004, R-hand plant stems only]; isotypes: BM [BM000778106], E [E00190739], G [G00370047], GH [00077670], K [K000585518], NDG [NDG45048], NY [00172003], PH [00030413], US [00027580, acc. # 1324554; 00650469, acc. # 3412819]).

**Figure 62.**
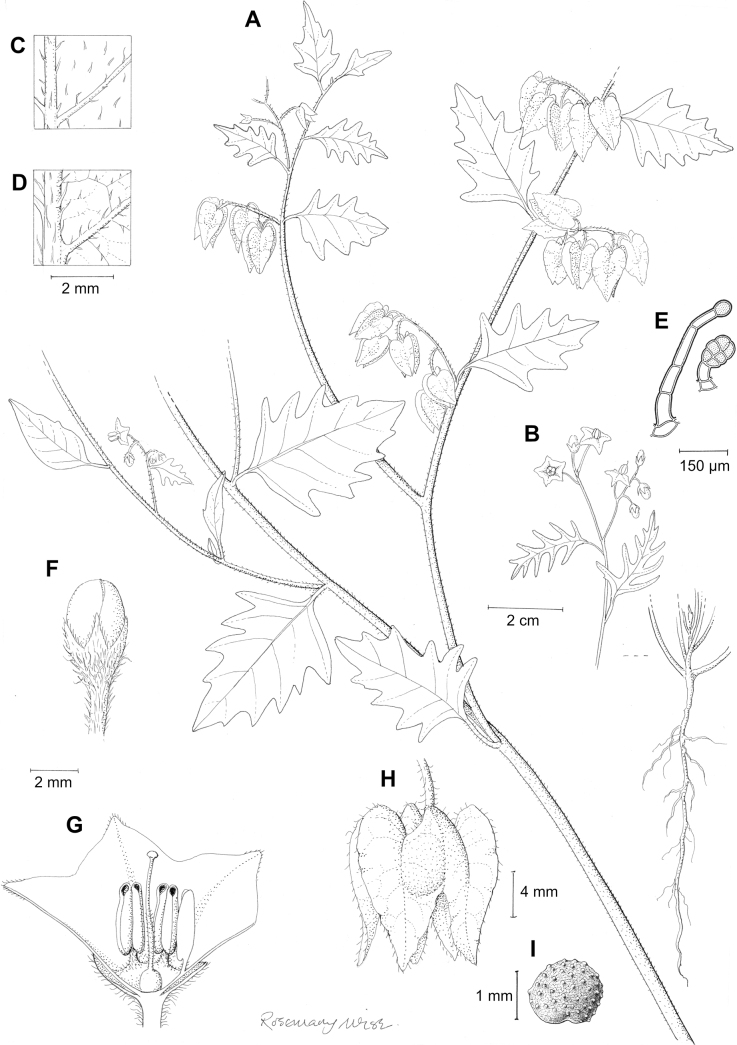
*Solanumgilioides***A** habit **B** flowering plant showing different leaf shapes and annual habit **C** detail of adaxial leaf surface **D** detail of abaxial leaf surface **E** trichomes on leaves **F** flower bud **G** dissected flower **H** maturing fruit with inflated calyx **I** seed (**A, C, D, H***Wood et al. 21974***B, F, G***Wood et al. 19056***E, I***Negritto et al. 429*). Illustration by R. Wise and L. Ribulgo.

##### Description.

Small annual herbs (0.05) 0.1–0.5 m high, usually prostrate and spreading. Stems terete, sparsely pubescent with transparent 4–6-celled simple uniseriate trichomes 0.5–1 mm long, these mixed glandular and eglandular; new growth densely to moderately pubescent with a mixture of glandular 1-celled papillae and transparent 4–6-celled simple uniseriate trichomes 0.5–1.5 mm long; older stems pale greenish yellow, glabrescent. Sympodial units difoliate, the leaves not geminate. Leaves simple, shallowly to deeply lobed, extremely variable even on a single plant, the blades 1.5–6.5 cm long, 0.6–2.4 cm long, narrowly elliptic in outline, widest at the middle, membranous to slightly thick and fleshy, concolorous; adaxial surfaces glabrous; abaxial surfaces sparsely pubescent with mixed glandular and eglandular 4–6-celled simple uniseriate trichomes 0.5–1 mm long on the veins and margins; principal veins 3–4(5) pairs, each ending in a lobe; base attenuate onto the petiole; margins shallowly to deeply lobed, the sinuses reaching ca. halfway to the midrib or less, the lobes 0.3–1 cm long, irregular, triangular to deltate with acute tips; apex acute and somewhat rounded; petiole 0.5–1.4 cm long, sparsely pubescent with eglandular white uniseriate trichomes ca. 0.5 mm long. Inflorescences opposite the leaves, unbranched, 1.2–4.5 cm long, with 2–7 flowers clustered at the tip, moderately pubescent with mixed glandular and eglandular simple uniseriate trichomes 0.5–1 mm long, always denser and longer than the stem pubescence; peduncle 1.2–5 cm long; pedicels (0.5)1–1.3 cm long, ca. 0.5 mm in diameter at the base, ca. 0.5 mm in diameter at the apex, drying purple in herbarium specimens, filiform, spreading at anthesis, moderately pubescent with a mixture of glandular papillae and eglandular simple uniseriate trichomes ca. 0.5 mm long, similar in density to the inflorescence, articulated at the base; pedicel scars tightly packed and spaced 3–5 mm apart in both flower and fruit. Buds globose, the corolla only just exserted from the calyx before anthesis. Flowers 5-merous, cosexual (hermaphroditic). Calyx tube ca. 2 mm long, conical, the lobes 2–2.5 mm long, 1.5–2 mm wide, narrowly deltate, sparsely to moderately pubescent with glandular papillae and eglandular simple uniseriate trichomes to 0.5 mm like those of the rest of the inflorescence, the venation prominent and drying dark purple or black. Corolla ca. 1.6 cm in diameter, violet to purple with a green central eye, rotate, lobed less than 1/4 of the way to the base, the lobes (acumens) 1–2 mm long, 3–4 mm wide, spreading or slightly cupped at anthesis, adaxially glabrous, abaxially glabrous but densely papillose on the acumen tips. Stamens equal; filament tube ca. 0.5 mm long; free portion of the filaments ca. 1.5 mm long, sparsely pubescent adaxially with tangled transparent simple uniseriate trichomes; anthers 1–3 mm long, 0.6–1 mm wide, yellow, ellipsoid with a somewhat prolonged and pointed tip, poricidal at the tips, the pores lengthening to slits with age. Ovary conical, glabrous; style (1)3–6 mm long (plants possibly heterostylous?), straight, exserted beyond the anther cone, glabrous; stigma capitate, the surface minutely papillose. Fruit a globose to somewhat ellipsoid berry, 0.6–0.7 cm long, 0.4–0.6 cm in diameter, green when mature(?), the pericarp thin, matte, opaque, glabrous; fruiting pedicels ca. 1.2 cm long, ca. 0.5 mm in diameter at the base, ca. 0.5 mm in diameter at the apex, not markedly woody, erect or spreading, not persistent; fruiting calyx accrescent and inflated, completely covering the berry, the tube ca. 5 mm long, strongly angled, the lobes ca. 10 mm long, ca. 6 mm wide, sharply pointed, somewhat overlapping and creating strong angles in the suture, the venation very evident, often drying blue or purple, the base invaginate. Seeds 9–20 per berry, 1.7–2.2 mm long, 1.4–1.7 mm wide, reniform, dark brown, the surfaces tuberculate, the testal cells pentagonal to rectangular in outline. Stone cells absent. Chromosome number: not known.

**Figure 63.**
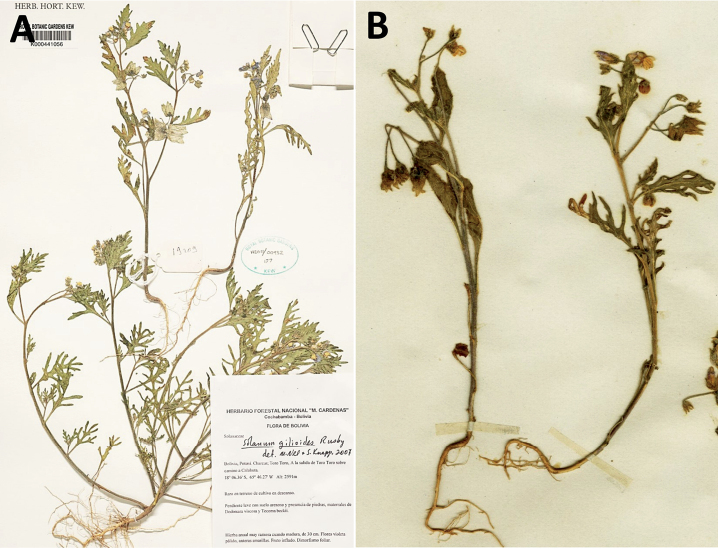
*Solanumgilioides***A** habit **B** smaller habit with variable leaf shape (**A***Wood et al. 19209* [K000441056] **B***Bang 938* [E00190739]). Reproduced with permission of the Trustees of the Royal Botanic Gardens, Kew and Royal Botanic Garden Edinburgh.

##### Distribution

**Figure 64.**
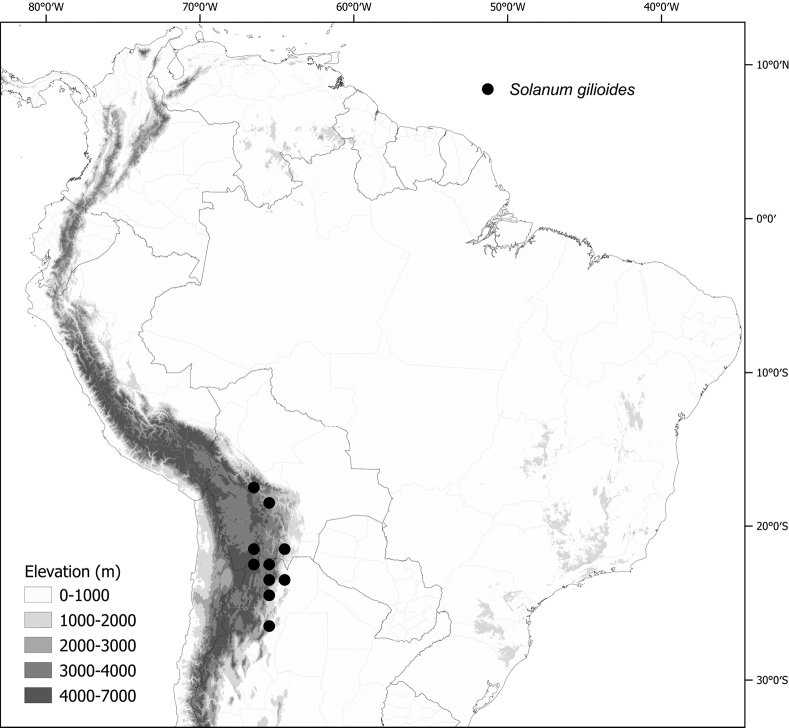
Distribution map of *Solanumgilioides*.

**(Fig. 64).***Solanumgilioides* is found from Bolivia (Depts. Cochabamba, Potosí, Tarija) to northern Argentina (Provs. Jujuy, Salta, Tucumán).

##### Ecology and habitat.

*Solanumgilioides* grows in rocky, grassy puna habitats, from 2,500 to 4,200 m, usually growing above 3,000 m elevation.

##### Common names and uses.

None recorded.

##### Preliminary conservation status.

Least Concern [LC]. EOO = 139,358 km^2^ [LC]; AOO = 64 km^2^ [EN]. Although relatively rarely collected, *S.gilioides* occurs over a wide geographic range and in places rarely visited by botanists. The high elevation habitats where it occurs, however, are often the sites of mines, and *S.gilioides* has not been recorded within any protected area. It may in future warrant an assessment of Near Threatened.

##### Discussion.

*Solanumgilioides* is a species of high elevations and was segregated, along with *S.annuum* and *S.weddellii* (as *S.chamaesarachidium*) as section Chamaesarachidium Bitter (Barboza 2003). Phylogenetic analysis with molecular sequence data confirms the close relationship of *S.gilioides* and *S.weddelli*, but not *S.annuum* (Särkinen et al. 2015b), whose relationships appear to be with other Black nightshades. *Solanumgilioides* is broadly sympatric with *S.weddellii*, also of high elevations, but that species tends to occur in sandy, rather than rocky habitats. The taxa both have strongly inflated calyces in fruit, but those of *S.gilioides* are larger and the lobes have acute to acuminate tips. The calyx lobes of *S.gilioides* are fused, like those of most species of *Physalis* while in *S.weddellii* the lobes remain free but overlapping. In both dried and fresh material of *S.gilioides* the stiff calyx lobes often have striking purple veins (Fig. 63A). Pubescence in *S.gilioides* is eglandular except for tiny glandular papillae, while *S.weddellii* often has longer, several celled glandular trichomes on leaves and stems. The flowers of *S.gilioides* are larger than those of *S.weddellii* (ca. 1.6 cm versus 0.6 cm in diameter, with anthers 1–3 mm long versus ca. 1 mm long).

The lectotype we have selected for *S.gilioides* (NY, barcode 00172004) is the sheet incorrectly referred to as “holotype” by Barboza et al. (2013). Only the right-hand stems on this sheet are referrable to *S.gilioides*, the single stem on the left is a small plant of *S.sisymbriifolium* Lam., a member of the Leptostemonum clade. The two taxa are not easily confused, as *S.sisymbriifolium* has copious prickles and stellate pubescence.

#### ﻿21. Solanum glandulosipilosum

Taxon classificationPlantaeSolanalesSolanaceae

Bitter, Repert. Spec. Nov. Regni Veg. 11: 213. 1912.

Figs 2E , 65 , 66

Solanum adenochlamys Bitter, Repert. Spec. Nov. Regni Veg. 13: 169. 1914. Type. Argentina. Salta: Rosario de la Frontera, 7 Jan 1905, *M. Lillo 3851* (lectotype, designated by Barboza et al. 2013, pg. 248: CORD [CORD00004103]; isolectotypes: A [01011895], G, LIL [LIL001446, acc. # 89084], NY [00139045]).
Solanum fabrisii Cabrera, Hickenia 1(31): 164. 1978. Type. Argentina. Jujuy: Santa Bárbara, El Fuerte, Loma Grande, 22 Nov 1970, *A.L. Cabrera & H. Fabris 21071* (no herbaria cited; lectotype, designated here: SI [003282, acc. # 065903]; isotypes: CORD [CORD00006801], LP [LP005354], SI [003662, acc. # 074664]).

##### Type.

Argentina. Tucumán: Siambón, Sierra de Tucumán, 11–17 Jan 1873, *P.G. Lorentz & G. Hieronymus 1035* (holotype: B, destroyed [F neg. 2776]; lectotype, designated by Barboza et al. 2013, pg. 248: CORD [CORD00004216]; isolectotype: GOET [GOET003257]).

**Figure 65.**
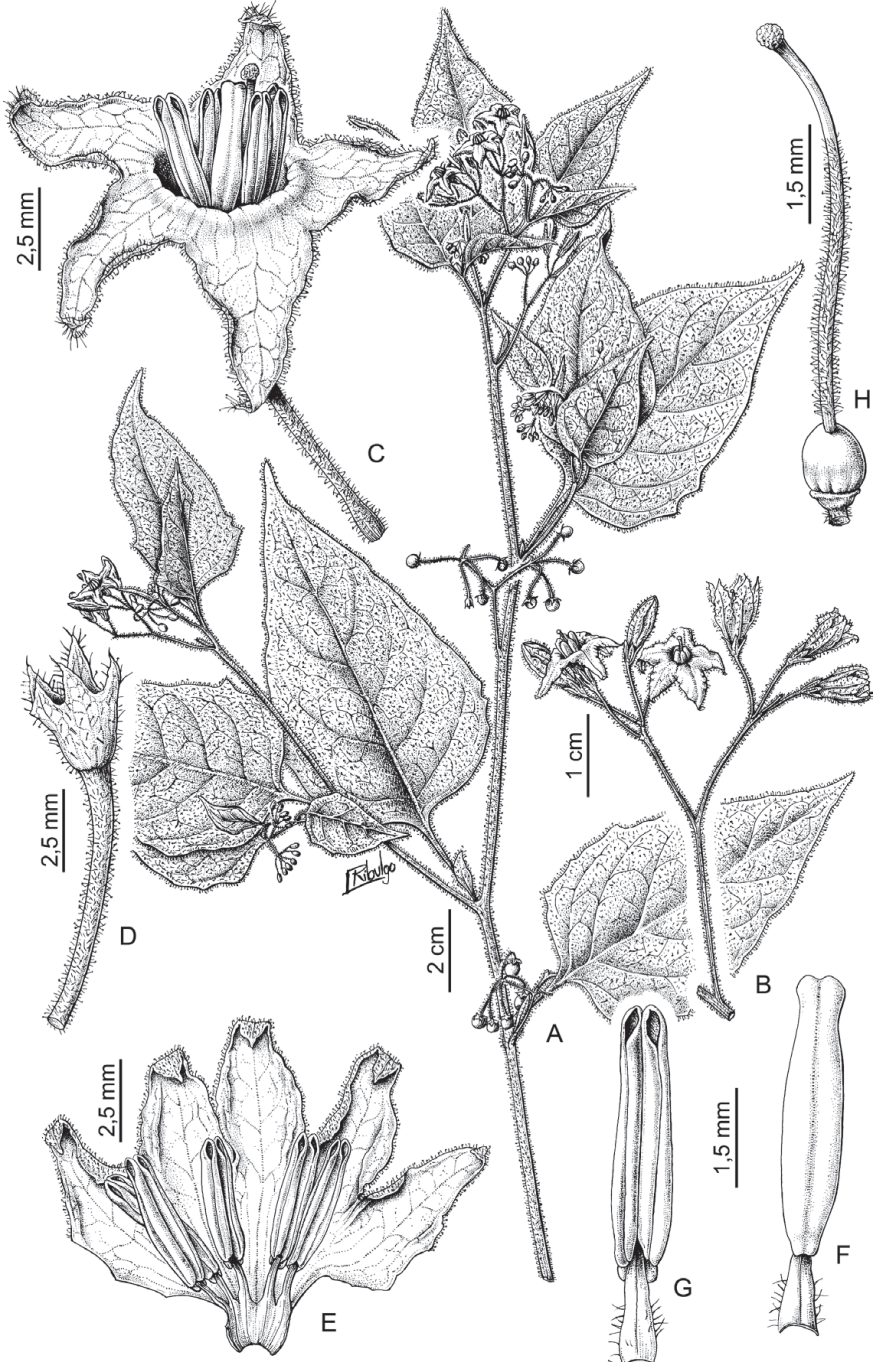
*Solanumglandulosipilosum***A** flowering and fruiting branch **B** inflorescence **C** flower **D** calyx **E** dissected flower **F** stamen, dorsal view **G** stamen, ventral view **H** gynoecium (**A–H***Venturi 2450*). Illustration by L. Ribulgo. Previously published in Barboza et al. (2013: 248).

##### Description.

Woody perennial herbs 0.6–1.2 m high, erect with a woody base. Stems terete, densely papillate and glandular-pubescent with transparent 2–8-celled simple uniseriate trichomes 0.5–1.5 mm long, these spreading; new growth densely glandular-pubescent with 2–8-celled transparent simple uniseriate trichomes like the stems, of varying lengths; bark of older stems greenish brown, pubescent (not markedly glabrescent). Sympodial units difoliate, the leaves not geminate. Leaves simple, entire, the blades 3.5–9(17) cm long, 1.7–5.5.(8.5) cm wide, ovate to narrowly ovate to elliptic, widest in the lower third or near the middle, membranous, concolorous, the lower leaves can be very large and are not often preserved on herbarium specimens; adaxial surfaces moderately and evenly glandular-pubescent with transparent simple uniseriate trichomes, these to 1 mm long on veins, shorter on the lamina; abaxial surfaces glandular pubescent like the upper surfaces, but the trichomes denser along the veins; principal veins 5–8 pairs; base more or less truncate to acute, oblique, not strongly decurrent onto the petiole; margins entire or occasionally slightly toothed in the lower third to half of the leaf blade; apex acute to acuminate; petiole 0.7–2(4) cm long, glandular-pubescent like the stems. Inflorescences internodal or occasionally opposite the leaves, forked or less commonly unbranched, 1–3 cm long, with 10–20 flowers clustered at the tips of the branches, glandular-pubescent with spreading, transparent simple uniseriate trichomes like those of the stems; peduncle 0.9–1.5 cm long, very obvious and erect in forked inflorescences; pedicels 0.7–0.9 cm long, ca. 0.25 mm in diameter at the base, ca. 0.5 mm in diameter at the apex, filiform, spreading at anthesis, densely glandular-pubescent with simple uniseriate trichomes to 1.5 mm long; pedicel scars closely spaced less than 0.5 mm apart at the tips of the branches to irregularly spaced ca. 1 mm apart in fruiting inflorescences, articulated at the base. Buds narrowly ellipsoid, the corolla strongly exserted from calyx before anthesis. Flowers 5-merous, cosexual (hermaphroditic). Calyx tube 1–1.5 mm long, conical, the lobes 1–2.5 mm long, ca. 1 mm wide, narrowly triangular, densely glandular-pubescent with spreading, transparent, simple uniseriate trichomes 1–1.5 mm long. Corolla 1.2–1.6 cm in diameter, white with a green central eye rimmed with purple or brown, stellate, lobed ca. 2/3 of the way to the base, the lobes 2–4 mm long, 3–6 mm wide, deltate, reflexed at anthesis, adaxially glabrous, abaxially glandular-pubescent with simple uniseriate trichomes especially along the midvein and at the tip. Stamens equal; filament tube minute; free portion of the filaments 1–1.5 mm long, glabrous or with a few tangled simple uniseriate trichomes adaxially; anthers 4–4.5 mm long, 1–1.1 mm wide, narrowly ellipsoid, yellow, poricidal at the tips, the pores lengthening to slits with age. Ovary conical, glabrous; style 5.5–7 mm long, straight, exserted beyond the anther cone, densely papillate with eglandular trichomes in the lower third; stigma clavate to capitate and lightly bilobed, the surface minutely papillate. Fruit a globose berry, 0.4–0.6 cm in diameter, green when mature, the pericarp thin, matte or slightly shiny, opaque, glabrous; fruiting pedicels 1–1.2 cm long, ca. 0.5 mm in diameter at the base, ca. 0.75 mm in diameter at the apex, not markedly woody, spreading, not persistent; fruiting calyx not accrescent, appressed to the berry to slightly spreading. Seeds 20–30 per berry, 1–1.2 mm long, 0.8–1 mm wide, flattened and teardrop shaped, pale yellowish tan, the surfaces minutely pitted, the testal cells sinuate in outline. Stone cells 6 (14 *fide*Bitter 1914a), scattered through the mesocarp, 4 ca. 1 mm in diameter, 2 ca. 0.4 mm in diameter. Chromosome number: n = 12 (Moscone 1992; voucher *Subils et al. 3609*).

**Figure 66.**
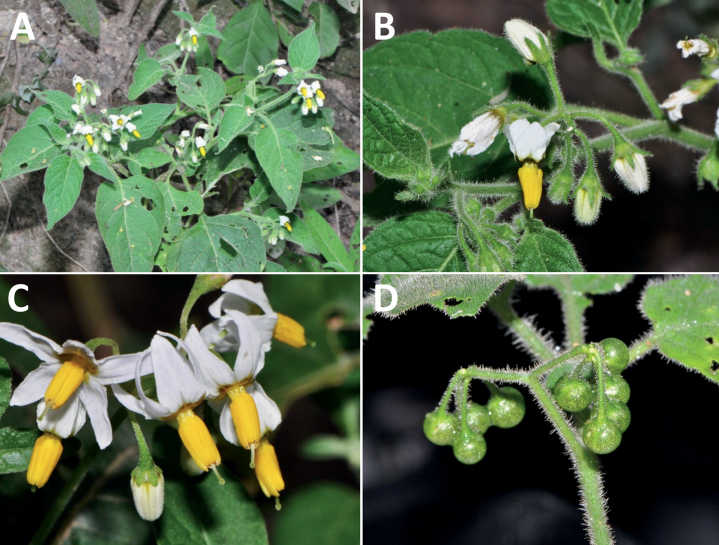
*Solanumglandulosipilosum***A** habit **B** inflorescence with buds **C** flowers at full anthesis **D** maturing fruits (**A–C***Barboza et al. 3520*; **D***Barboza et al. 3504*). Photos by S. Knapp.

##### Distribution

**Figure 67.**
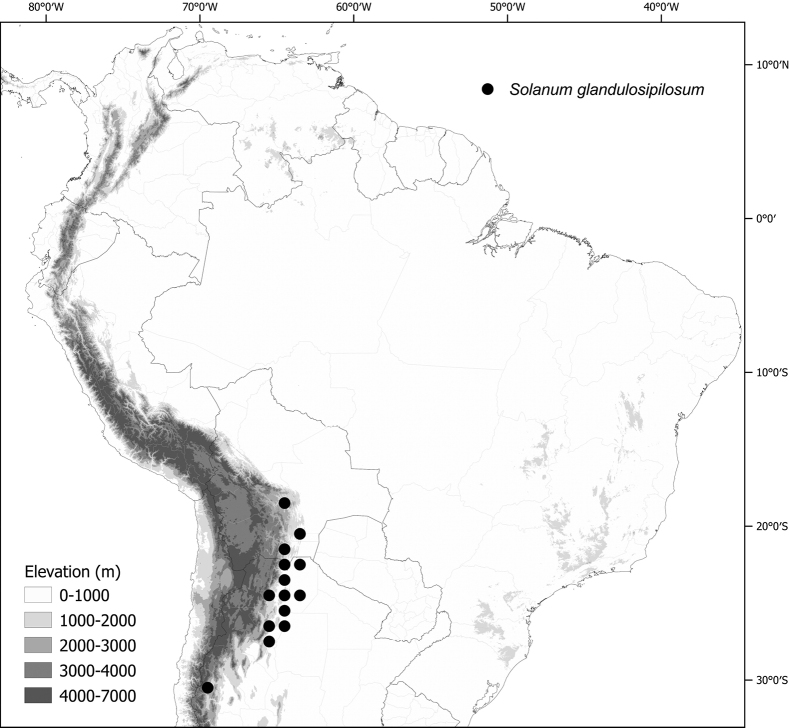
Distribution map of *Solanumglandulosipilosum*.

**(Fig. 67).***Solanumglandulosipilosum* is known from southern Bolivia (Provs. Chuquisaca and Tarija) and northern Argentina (Depts. Jujuy, Salta and Tucumán).

##### Ecology and habitat.

*Solanumglandulosipilosum* grows in moist forests, often in somewhat disturbed sites, from 350 to 2,640 m elevation.

##### Common names and uses.

None recorded.

##### Preliminary conservation status

**(IUCN 2022).** Least Concern [LC]. EOO = 269,652 km^2^ [LC]; AOO = 140 km^2^ [EN]. *Solanumglandulosipilosum* is a plant of disturbed and open areas and is relatively widely distributed. It has been recorded from protected areas in Argentina (Parque Nacional Baritú) and from near the Reserva Nacional de Tariquía (Bolivia).

##### Discussion.

*Solanumglandulosipilosum* is morphologically most similar to *S.aloysiifolium*, sharing with that species narrowly ellipsoid buds and small green or purple berries. It differs from *S.aloysiifolium* in its copious glandular pubescence and fewer (6 versus 10) stone cells per berry. The two species are sympatric, growing in similar disturbed and moist forest habitats, but are easily distinguishable vegetatively. Särkinen et al. (2015b) did not resolve the two species as sister; *S.glandulosipilosum* resolved as a member of a group with *S.americanum*, *S.nigrescens* and other North American taxa together with a large number of polyploid taxa with no obvious morphological affinity, while *S.aloysiifolium* was sister to somewhat similar and geographically close *S.chenopodioides* and *S.enantiophyllanthum*.

In describing *Solanumfabrisii*, Cabrera (1978) cited only the herbarium SI, not which of the two sheets of *Cabrera & Fabris 21701* held there was the type; one sheet is annotated as “holotype” with a typed label and is the best preserved of the two duplicates at SI (003282); we select this sheet as the lectotype.

#### ﻿22. Solanum gonocladum

Taxon classificationPlantaeSolanalesSolanaceae

Dunal, Prodr. [A. P. de Candolle] 13(1): 93. 1852.

Figs 68 , 69

Solanum poecilochromifolium Rusby, Bull. New York Bot. Gard. 4: 419. 1907. Type. Bolivia. sin loc., sin. dat., *M. Bang 2515* (no herbaria cited; lectotype, designated here: NY [00172135]; isolectotypes: K [K000585519], NY [00172134], US [00027749, acc. # 1324710]).
Solanum bangii Bitter, Repert. Spec. Nov. Regni Veg. 10: 552. 1912. Type. Bolivia. La Paz: vic. La Paz, 10,000 ft., 1889, *M. Bang 64* (lectotype, designated by Barboza et al. 2013, pg. 264: BM [BM000778230]; isolectotypes: BR [BR0000005538201, BR0000005538539], G [G00343455], NY [00172113], PH [00030385]).
Solanum atricoeruleum Bitter, Repert. Spec. Nov. Regni Veg. 10: 563. 1912.
Type. Bolivia. La Paz: sin. loc., 3,800 m, Apr 1910, *O. Buchtien 2964* (no herbaria cited; lectotype, designated here: US [01919650, acc. # 1133279]; isolectotypes: NY [00139058], US [01919649, acc. # 700119]).
Solanum nanum Bitter, Repert. Spec. Nov. Regni Veg. 10: 564. 1912. Type. Bolivia. La Paz: sin. loc., 3,800 m, Apr 1910, *O. Buchtien 2963* (no herbaria cited; lectotype, designated here: US [00027700, acc. # 133298]; isolectotypes: GOET [GOET003481], US [00027465, acc. # 1133278; 01014276, acc. # 700118], NY [00172103]).

##### Type.

Bolivia. La Paz: circa Roma de la Paz, A. *D’Orbigny 1541* (lectotype, designated here: P [P00335462]; isotypes: G [00359947], P [P00335463], W [acc. # 1889-0127571]).

**Figure 68.**
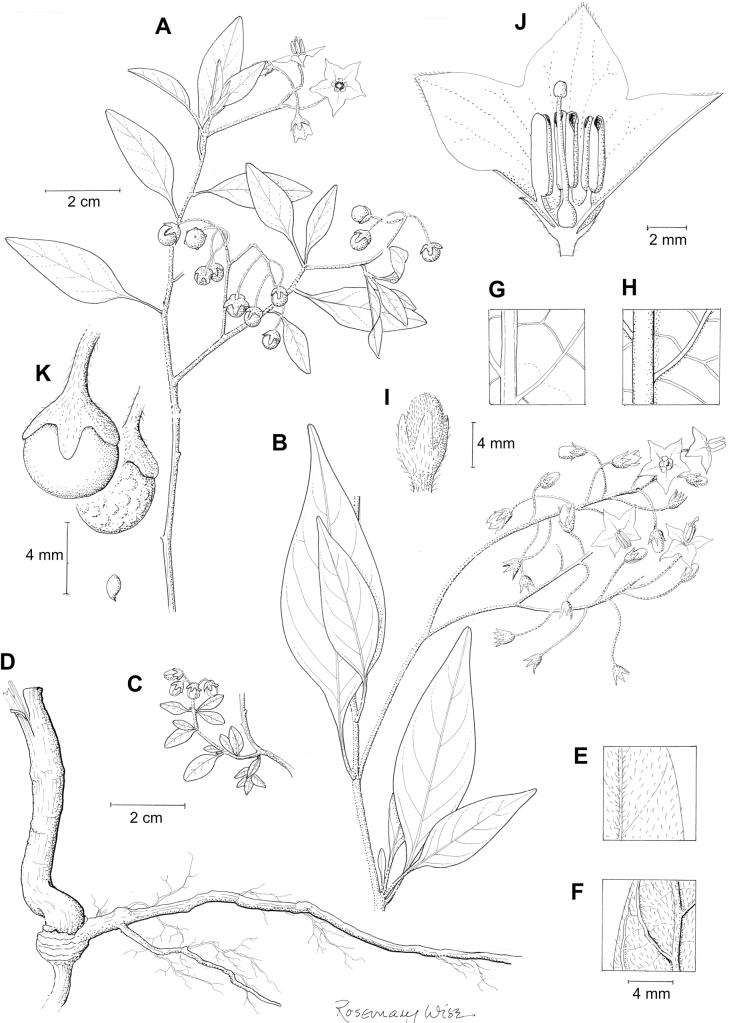
*Solanumgonocladum***A** habit with flowers and fruits **B** flowering habit with larger leaves **C** flowering habit with smaller leaves **D** woody base of stem with roots **E** detail of adaxial leaf surface **F** detail of abaxial leaf surface **G** detail of adaxial leaf surface (glabrous individual) **H** detail of abaxial leaf surface (glabrous individual) **I** bud **J** dissected flower **K** fully mature fruit with seed (**A, E, F, K***Buchtien 4454***B, I***Buchtien 8665***C, D***Buchtien 2964***G, H, J***Buchtien 4452*). Illustration by R. Wise.

##### Description.

Small shrubs to 1 m high, often caespitose, the base markedly woody. Stems terete, with a very leafy appearance, moderately pubescent with white eglandular, simple few-celled uniseriate trichomes to 0.5 mm long, these usually strongly antrorse; new growth densely white pubescent with eglandular simple uniseriate trichomes like those of the stems; bark of older stems pale greenish or greyish brown. Sympodial units plurifoliate, the leaves not geminate, often clustered in groups of different sizes at the nodes giving the plant a very leafy appearance. Leaves simple, the blades 0.9–8 cm long, 0.3–3.2 cm wide, narrowly elliptic to elliptic, widest at or just above the middle, membranous to chartaceous, concolorous; adaxial surfaces sparsely to moderately and evenly (to very densely in extremely small-leaved plants) pubescent with white eglandular simple few-celled uniseriate trichomes to 0.5 mm long; abaxial surfaces similarly pubescent, but the trichomes denser along the veins; principal veins 4–5 pairs, more densely pubescent than the lamina abaxially; base attenuate, decurrent along the petiole but not along the stem; margins entire or very occasionally with a few scattered teeth to ca. 1 mm long, ca. 1 mm wide; apex acute to slightly obtuse, with the ultimate tip rounded; petioles absent and the leaves sessile from the attenuate bases, the winged portion to 1 cm long. Inflorescences opposite the leaves, forked (occasionally unbranched, e.g., *Nee 34108*), 2–6(–10) cm long, with 20–30 flowers borne in the distal half of the branches, evenly pubescent with antrorse white eglandular simple few-celled trichomes ca. 0.5 mm long like those of the stems; peduncle 1–3 cm long; pedicels 0.9–1.4 cm long, ca. 0.5 mm in diameter at the base, ca. 1 mm in diameter at the apex, rather stout-looking, evenly pubescent like the rest of the inflorescence, spreading at anthesis, articulated at the base; pedicel scars evenly spaced 1–3 mm apart. Buds ellipsoid, the corolla ca. halfway exserted from the calyx before anthesis. Flowers 5-merous, cosexual (hermaphroditic). Calyx tube 2–2.5 mm long, cup-shaped, the lobes 1.5–2 mm long, 1.2–1.5 mm wide, usually shorter than the tube, deltate to short-triangular with rounded tips, usually drying black, evenly pubescent with white eglandular simple few-celled uniseriate trichomes ca. 0.5 mm long, these usually somewhat antrorse, the sinuses thinner and in dry material appearing somewhat scarious. Corolla 1.3–2 cm in diameter, pale purple to violet with a yellow central star, stellate, lobed ca. halfway to the base, the lobes ca. 5 mm long, 3.5–6 mm wide, spreading at anthesis, adaxially glabrous, abaxially densely pubescent-puberulent where exposed in bud with eglandular simple uniseriate trichomes to 0.2 mm long or less, the interpetalar tissue glabrous. Stamens equal; filament tube minute; free portion of the filaments 1–1.5 mm long, with a few transparent tangled simple uniseriate trichomes at the base; anthers 4–4.5 mm long, 1.5–1.75 mm wide, ellipsoid, yellow, poricidal at the tips, the pores lengthening to slits with age. Ovary conical, glabrous; style 7–8 mm long, straight (curved in bud), long-exserted beyond the anther cone (sometimes exserted from the closed corolla in bud), densely pubescent in the lower half; stigma large capitate, the surfaces minutely papillate, green in live plants. Fruit a globose berry, 0.8–1 cm in diameter, greenish yellow when ripe, the pericarp thin, more or less shiny, translucent, glabrous; fruiting pedicels 1.4–1.5 cm long, ca. 0.7 mm in diameter, ca. 1.2 mm in diameter at the apex, somewhat woody, strongly deflexed at the base with a distinct kink at the very base so the fruits almost point back towards the main stem, not persistent; fruiting calyx not markedly accrescent, the tube 2–3 mm long, appressed on the berry, the lobes 2–2.5 mm long, spreading, with the tips reflexed and markedly rounded. Seeds 20–40 per berry, ca. 2 mm long, 1.2–1.5 mm long, flattened and teardrop shaped, reddish brown, the surfaces minutely pitted, the testal cells sinuate in outline. Stone cells 4–6 per berry, 2 apical ca. 1 mm in diameter, the rest (2–4) equatorial or scattered, ca. 0.7 mm in diameter, all cream-coloured. Chromosome number: not known.

**Figure 69.**
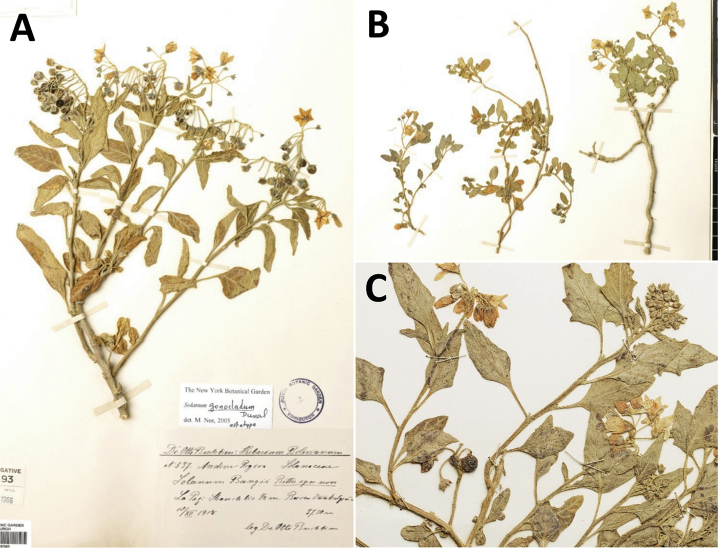
*Solanumgonocladum***A** habit **B** smaller habit **C** details of flowering and fruiting branches (**A***Buchtien 537* [E00426360] **B***Buchtien 467* [E00426359] **C***Balls 5892* [E00426434]). Reproduced with permission of the Trustees of the Royal Botanic Garden Edinburgh.

##### Distribution

**Figure 70.**
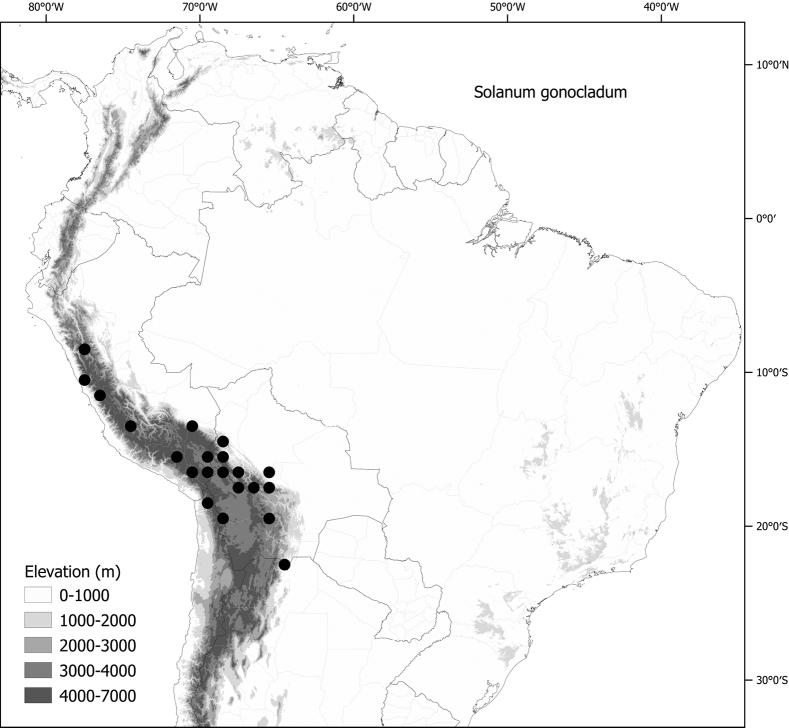
Distribution map of *Solanumgonocladum*.

**(Fig. 70).***Solanumgonocladum* is a high elevation Andean species, occurring from central and southern Peru (Depts. Ancash, Ayacucho, Cusco, Junín, Moquegua, Puno), Bolivia (Depts. Chuquisaca, Cochabamba, La Paz, Potosí) into northern Chile (Region XV [Arica y Parinacota]).

##### Ecology and habitat.

*Solanumgonocladum* is a plant of open spaces, often occurring in rocky landslides and outcrops; it is most commonly collected in puna or pre-puna habitats from (1,500) 2,600 to 4,000 m elevation.

##### Common names and uses.

Peru. Cusco: chinchi-chinchi (*Herrera 2178*); Moquegua: ñuccho hembra, ñuccho con pelo (*Montesinos 920*). No uses recorded.

##### Preliminary conservation status

**(IUCN 2022).** Least Concern [LC]. EOO = 541,223 km^2^ [LC]; AOO = 284 km^2^ [EN]. *Solanumgonocladum* occurs widely in the high Andes and is often a plant of open, disturbed areas. It has been recorded from protected areas in Bolivia (e.g., Parque Nacional Tunari).

##### Discussion.

*Solanumgonocladum* is an upright shrubby plant often with scrambling stems that are extremely woody at the base. The flowers are large in comparison to other South American morelloid species (to 2 cm in diameter, with anthers to 5 mm long) and the darkened spathulate calyx lobes that hug the base of the berry are diagnostic. It is usually a species of puna regions at high elevation, although *Eyerdam 24741* (F, K) was collected at 1,500 m elevation. *Solanumgonocladum* is somewhat similar morphologically to *S.interandinum*, with which it shares spathulate calyx lobes and strongly deflexed fruiting pedicels but differs from it in its slightly larger flowers (1.6–2 cm in diameter versus 0.8–1.4 cm in diameter, with anthers 3–5 mm long versus 2.5–3 mm long), and larger berries (0.8–1 cm in diameter versus 0.6–0.8 cm in diameter). In both species the inflorescence often remains on older stems after flowers and fruits have fallen. In the consensus phylogeny of Särkinen et al. (2015b)*S.gonocladum* was part of a clade including *S.pallidum* and *S.cochabambense* (as. *S.probolospermum*).

One collection from the Bolivian Department of Potosí (*Wood 10648*) is included here with some reservation. It is the only collection we have seen from this far south in Bolivia, and in the phylogenetic reconstructions of Särkinen et al. (2015b) it does not cluster with other accessions of *S.gonocladum* nor with *S.salicifolium* which it also somewhat resembles. Since the ploidy level of *S.gonocladum* remains untested and the accession could be clustering with one or the other parent if *S.gonocladum* is a polyploid, we hesitate to recognise this collection as a new taxon until further collecting in southern Bolivia reveals additional accessions.

In describing *S.gonocladum*, Dunal (1852) cited two specimens he had seen in Paris both collected by Alcide d’Orbigny (*d’Orbigny 1541* and *1536B*); we have selected the more complete of these two gatherings at P (*d’Orbigny 1541*, P003355462) that is annotated by Dunal and has the type locality on the original label as the lectotype for this name.

The protologue of *S.poecilochromifolium* does not cite a herbarium (Rusby 1907); we have selected the best preserved of the duplicates of *Bang 2515* at NY (barcode 00172135) with flowers and fruits as the lectotype.

*Solanumbangii* was lectotypified by Barboza et al. (2013) with the specimen of *Bang 64* at BM (BM000778230). Other duplicates of *Bang 64* were also used by Rusby (1895) to describe *S.pallidum*, so care must be taken in assigning lectotype status to duplicates of *Bang 64* (see discussion in description of *S.pallidum*).

Bitter (1912a) described both *S.atricocoeruleum* and *S.nanum* from collections made by Otto Buchtien in Bolivia but cited no specific herbaria. Both *Buchtien 2964* and *Buchtien 2963 pro parte* were cited in the protologue of *S.atricocoeruleum* and only *Buchtien 2963 pro parte* was cited for *S.nanum*, differentiating the duplicate cited as being smaller. We have therefore selected *Buchtien 2964* at US (barcode 01919650, acc. # 1133279) annotated by Bitter as *S.atricocoeruleum* as the lectotype for *S.atricocoeruleum* and *Buchtien 2963* (US barcode 00027700, acc. # 133298, annotated as *S.nanum* by Bitter) as the lectotype of *S.nanum*. Other duplicates of *Buchtien 2963* (e.g., GOET003481) were annotated by Bitter as *S.atricocoeruleum*. A number of collections with the collecting number *Buchtien 2964* (e.g., B_10_0248774, HBG-511402, HBG-511403, HBG-511404) have collection dates in the 1930s, too late to be original material for *S.atricoeruleum*, despite bearing the same collection number.

#### ﻿23. Solanum grandidentatum

Taxon classificationPlantaeSolanalesSolanaceae

Phil., Anales Mus. Nac. Chile, Segunda Secc., Bot. 1891: 64. 1891.

Figs 71 , 72

Solanum tarapacanum Phil., Anales Mus. Nac. Chile, Segunda Secc., Bot. 1891: 65. 1891.
Chile. Región I (Tarapacá): Prov. Tarapacá, Chiapa, 16 Mar 1885, *C.F. Rahmer s.n.* (lectotype, designated here: SGO [SGO000004602, acc. # 055509]; isolectotype; SGO [SGO000004601, acc. # 042709]).
Solanum sanfurgoi Phil., Anales Univ. Chile 91: 10. 1895. Type. Chile. Región VII (Maule): “Maule, Inter Curanipe et Buchupureo”, *L. Sanfurgo s.n.* (lectotype, designated here: SGO [SGO000004598, acc. # 055542]).
Solanum excisirhombeum Bitter, Repert. Spec. Nov. Regni Veg. 11: 1. 1912. Type. Peru. Ancash: Prov. Cajatambo, “In marginibus viarum prope Tallenga”, 3,600 m, 14 Apr 1903, *A. Weberbauer 2868* (holotype: B, destroyed [F neg. 2604]; lectotype, designated here: MOL [MOL00005705]).
Solanum myriadenium Bitter, Repert. Spec. Nov. Regni Veg. 12: 157. 1913. Type. Argentina. Jujuy: El Moreno, 11 Dec 1901, *R.E. Fries 890* (holotype: S [acc. # 04-2955]; isotypes: G, P [P00335348]).

##### Type.

Chile. Region I (Tarapacá): Paroma, 25 Feb 1885, *F. Philippi s.n.* (lectotype, designated here: SGO [SGO000004568, acc. # 055521]; possible isolectotype: SGO [SGO000004570a, acc. # 055520, left-hand plant only]).

**Figure 71.**
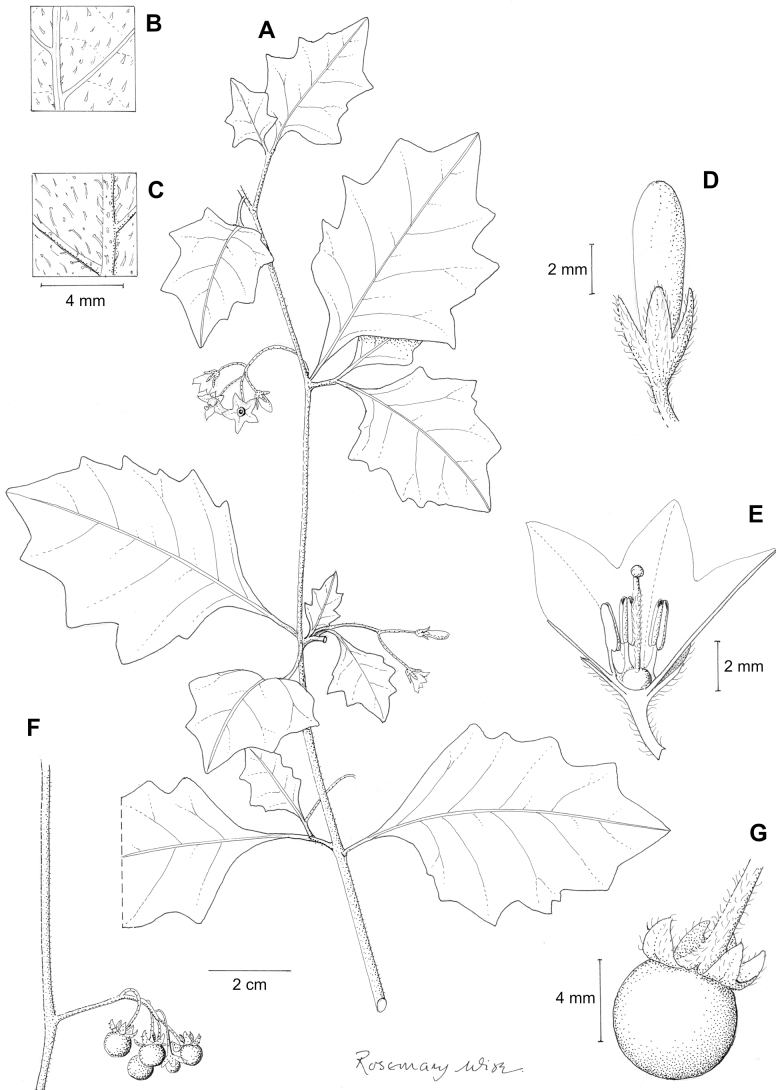
*Solanumgrandidentatum***A** habit **B** detail of adaxial leaf surface **C** detail of abaxial leaf surface **D** bud **E** dissected flower **F** infructescence **G** fully mature fruit (**A–D, F, G***Särkinen et al. 4003***E***Knapp et al. 10324*). Illustration by R. Wise.

##### Description.

Small shrubs or subshrubs, 0.3–0.7 m high, sprawling to 1 m in diameter, the branches usually erect, but decumbent as they elongate. Stems terete or slightly angled, moderately to densely pubescent with whitish or transparent mixed glandular and eglandular 2–3-celled simple uniseriate trichomes mostly ca. 0.5 mm long, a few longer and to 1 mm long, the gland single-celled; new growth densely pubescent with whitish or transparent mixed glandular and eglandular 2–3-celled simple uniseriate trichomes ca. 0.5 mm long; bark of older stems pale brown, glabrescent. Sympodial units difoliate, the leaves geminate or not geminate. Leaves simple and shallowly toothed, the blades 1.5–6.5 cm long, 0.9–5 cm wide, ovate to elliptic-ovate, widest in the lower third to quarter, membranous to slight fleshy and rubbery (smell of rhubarb fide *Knapp et al. 10219*), slightly discolorous; adaxial surfaces moderately and evenly pubescent with white glandular 2–3-celled simple uniseriate trichomes 0.5–0.75 mm long, these denser along the veins; abaxial surfaces similarly pubescent with white glandular 2–3-celled simple uniseriate trichomes 0.5–0.75 mm long, with a few trichomes longer and to 1 mm long; base cuneate then attenuate and decurrent onto the petiole; margins shallowly and irregularly toothed, the teeth ca. 2 mm long, ca. 1.5 mm wide, with rounded to acute tips, the sinuses rounded, reaching to 1/6 of the way to the midrib; apex acute; petioles 0.4–1.2 cm long, narrowly winged from the decurrent leaf base, pubescent with a mix of eglandular and glandular simple uniseriate trichomes like those of the stems. Inflorescences internodal, unbranched or forked, 1.5–3(5) cm long, with 7–20 flowers clustered in the distal parts of the branches, pubescent with whitish or transparent mixed glandular and eglandular 2–3-celled simple uniseriate trichomes mostly ca. 0.5 mm long, a few longer and to 1 mm long, the gland single-celled; peduncle 1–2.5 cm long; pedicels 0.5–0.8 cm long, ca. 0.5 mm in diameter at the base, ca. 1 mm in diameter at the apex, tapering, spreading or deflexed, pubescent with a mix of glandular and eglandular 2–3-celled simple uniseriate trichomes like the inflorescence axis, articulated at the base leaving a small stump or peg to 1 mm long on the axis; pedicel scars 0.5–1.5 mm apart, small raised pegs. Buds globose, the corolla ca. halfway exserted from the calyx before anthesis. Flowers 5-merous, cosexual (hermaphroditic). Calyx tube 0.5–1 mm long, conical, the lobes 1–1.5 mm long, ca. 0.75 mm wide, broadly triangular, reflexed in both flower and fruit, pubescent with whitish or transparent mixed glandular and eglandular 2–3-celled simple uniseriate trichomes mostly ca. 0.5 mm long like the pedicels and stems. Corolla 1.2–1.5 cm in diameter, white, white tinged with violet, with a greenish yellow eye, stellate, lobed 2/3 to 1/2 way to the base, the lobes 4.5–6 mm long, ca. 4 mm wide, broadly deltate, slightly cupped (campanulate) to spreading at anthesis, adaxially glabrous, abaxially sparsely to moderately pubescent with white eglandular 2–3-celled simple uniseriate trichomes to 0.5 mm long, these denser on the tips and margins. Stamens equal; filament tube to 0.5 mm long; free portion of the filaments 1–1.2 mm long, densely pubescent with tangled transparent simple uniseriate trichomes adaxially; anthers 2–2.5 mm long, ca. 1 mm wide, plumply ellipsoid, yellow, poricidal at the tips, the pores lengthening to slits with age. Ovary conical, glabrous; style 5–7.5 mm long, strongly curved in bud, straight at anthesis, exserted beyond the anther cone, densely pubescent in the lower half with transparent eglandular simple uniseriate trichomes; stigma ovoid-capitate, green in live plants, the surface minutely papillate. Fruit a globose berry, 0.6–0.8 cm in diameter, green when immature, green with white stripes along the carpel divisions when ripe, the pericarp thin, matte to somewhat shiny, becoming translucent with ripening, glabrous; fruiting pedicels 0.9–1.1 cm long, ca. 0.75 mm in diameter at the base, 1–1.2 mm in diameter at the apex, not markedly woody, deflexed, not persistent; fruiting calyx not markedly enlarged or accrescent, the lobes to 3.5 mm long, the tips reflexed and sticky on both surfaces. Seeds 20–30 per berry, 1.5–2 mm long, 1–1.5 mm wide, flattened teardrop shape, reddish brown, the surfaces minutely pitted, the testal cells sinuate in outline. Stone cells absent. Chromosome number: 2n = 48 (Chiarini et al. 2017, voucher *Knapp et al. 10413*; also recorded on sheets of *Heiser 4863*, *4910*, *5002*, *6036*, *6062*). Edmonds (1977) reported a count of 2n = 48 from *Jørgensen 2632* collected from seeds grown from *Heiser 5002*.

**Figure 72.**
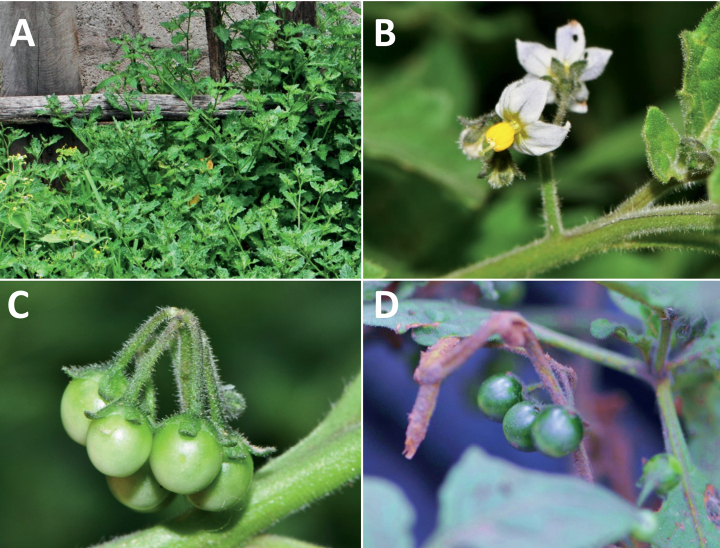
*Solanumgrandidentatum***A** habit **B** flowers at full anthesis **C** maturing fruits **D** fully mature fruits (**A–C***Knapp et al. 10413***D***Särkinen et al. 4699*). Photos by S. Knapp and T. Särkinen.

##### Distribution

**Figure 73.**
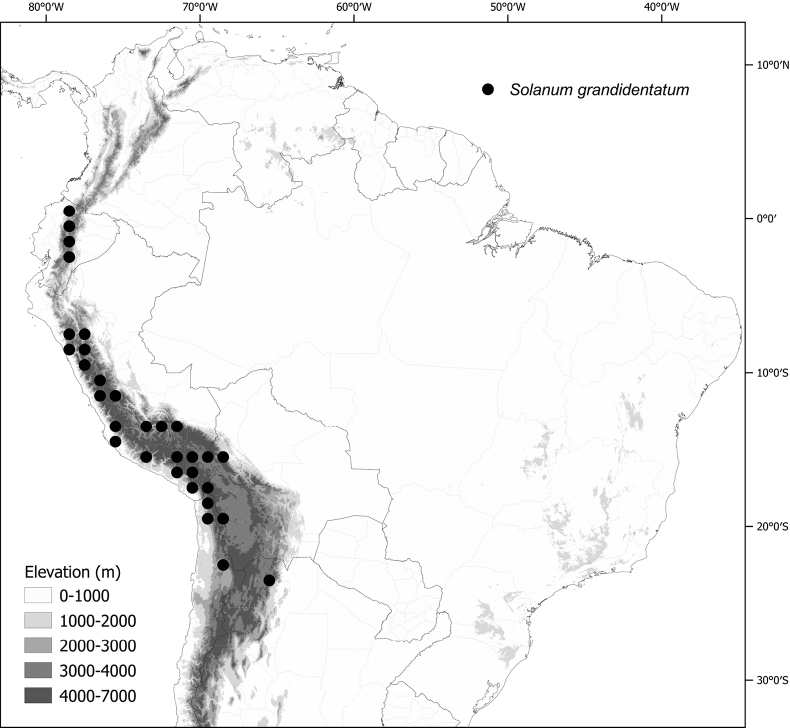
Distribution map of *Solanumgrandidentatum*.

**(Fig. 73).***Solanumgrandidentatum* occurs in the Andes of Ecuador (Provs. Cañar, Cotopaxi, Chimborazo, Pichincha, Tungurahua), Peru (Depts. Ancash, Apurímac, Arequipa, Cajamarca, Cusco, Huancavelica, Huánuco, Junín, Lima, La Libertad, Moquegua, San Martín, Tacna), Bolivia (Depts. La Paz, Potosí), reaching northern Chile (Regiones I [Tarapacá], II [Antofagasta] and XV [Arica y Parinacota]) and Argentina (Prov. Jujuy).

##### Ecology and habitat.

*Solanumgrandidentatum* is a plant of medium to high elevation open areas, often along roads or streams, or in disturbed areas such as landslides or roadcuts. It seems to grow in more fertile soils and is often found directly outside dwellings or in waste channels. It is most commonly collected from (1,300-) 2,000 to 4,500 m elevation, but a single collection from near the Panamericana in central Perú (*Ferreyra 18050*, USM) probably represents seeds brought from the Andes in seasonal mudslides. Similar low elevation occurrences of high elevation solanums on the outwashes of ‘huaicos’ have been documented in the Tomato clade (e.g., *S.corneliomulleri* J.F.Macbr., Peralta et al. 2008).

##### Common names and uses.

Ecuador. Chimborazo: yerba mora (*Acosta Solís 7598*); Pichincha: hierba mora (*Cerón 15909*). Peru. Huánuco: orqu qapachinya (Quechua, *Carter 86*). No uses recorded.

##### Preliminary conservation status

**(IUCN 2022).** Least Concern [LC]. EOO = 1,114,912 km^2^ [LC]; AOO = 300 km^2^ [EN]. *Solanumgrandidentatum* is widely distributed and thrives in areas highly disturbed by people. In has been collected in several protected areas throughout its range (e.g., Parque Nacional Apolobamba, Bolivia; around the historical sites of Pisaq and Sacsayhuamán in Peru; Reserva Geobotanica del Pululahuá in Ecuador).

##### Discussion.

*Solanumgrandidentatum* was long known as *S.excisirhombeum* (e.g., Edmonds 1972), but Philippi’s (1891) name has priority. This shrubby Andean species is morphologically very similar to *S.fragile* but differs in its shrubby habit (*S.fragile* has a woody underground rootstock, and specimens often consist of the brittle, herbaceous stems only), its distinct somewhat musty odour when fresh, and narrower, lanceolate calyx lobes. Both taxa are densely glandular pubescent and lack stone cells in the fruit. Calyx lobes of *S.grandidentatum* are 1–1.5 mm long with acuminate or acute tips, while those of *S.fragile* are 2–3 mm long with blunt tips; leaves can also be useful in distinguishing the two taxa, those of *S.grandidentatum* are more attenuate at the base. *Solanumgrandidentatum* is a tetraploid while the chromosome number of *S.fragile* (Edmonds 1972, 1977) needs confirmation with vouchered accessions (see discussion under *S.fragile*).

Molecular sequence data suggest the two species are not closely related (Särkinen et al. 2015b; Gagnon et al. 2022), but this result could be affected by polyploidy. Both species are part of weakly supported groups (polytomies), but different ones (see appendix S11 in Gagnon et al. 2022).

The description of *S.grandidentatum* was based on specimens “Ad Paroma in rupibus lecta, nec non ad Sibaya” (Philippi 1891), suggesting that the only relevant locality was Paroma. No collector or herbarium was cited, but the publication was based on Rudolfo Philippi’s son’s (Federico Philippi) collections from Tarapacá (done together with Carlos Rahmer). The introduction to the catalogue details when collections were done together and when separately (Philippi 1891). We have lectotypified this name with a specimen in the herbarium in Santiago (SGO000004568, acc. # 05552) that corresponds with the protologue. The right-hand stem on an additional sheet (SGO000004570a, acc. # 055520) maybe a duplicate, and thus an isolectotype, but since dates were not mentioned in the protologue it is difficult to be sure; the left-hand stem on this sheet was collected by Carlos Rahmer in Sibaya and is perhaps the other collection mentioned in the protologue. *Solanumtarapacense* was described in the same publication, from “Chiapa et Calcallmay (3700 m.s.m.) lecta”; we select as the lectotype of this species an un-numbered collection of Carlos Rahmer from Chiapa at SGO (SGO000004602, acc. # 055509). *Solanumsanfurgoi* was described citing a collection of “Ludovicus Sanfurgo” without citation of a herbarium; we lectotypify this with a sheet collected by Sanfurgo in the locality cited in the protologue (Philippi 1895) in SGO (SGO000004598, acc. # 055542).

Bitter (1912b) based *S.excisirhombeum* on *Weberbauer 2868* in “herb. Berol.”; this specimen is no longer extant, and we select here the sheet of *Weberbauer 2868* in MOL (MOL0000575) as the lectotype.

#### ﻿24. Solanum huayavillense

Taxon classificationPlantaeSolanalesSolanaceae

Del Vitto & Peten., Kurtziana 24: 167. 1995.

Figs 3E , 74 , 75

Solanum pachyantherum Bitter, Repert. Spec. Nov. Regni Veg. 11: 206. 1912., nom. illeg., non Solanumpachyantherum Witasek (1910). Type. Bolivia. Tarija: Huayavilla, 6 Dec 1903, *K. Fiebrig 2507* (lectotype, designated by Barboza et al. 2013, pg. 250: CORD [CORD00004254]; isolectotype: SI [003330, acc. # 055986]).

##### Type.

Based on *Solanumpachyantherum* Bitter.

**Figure 74.**
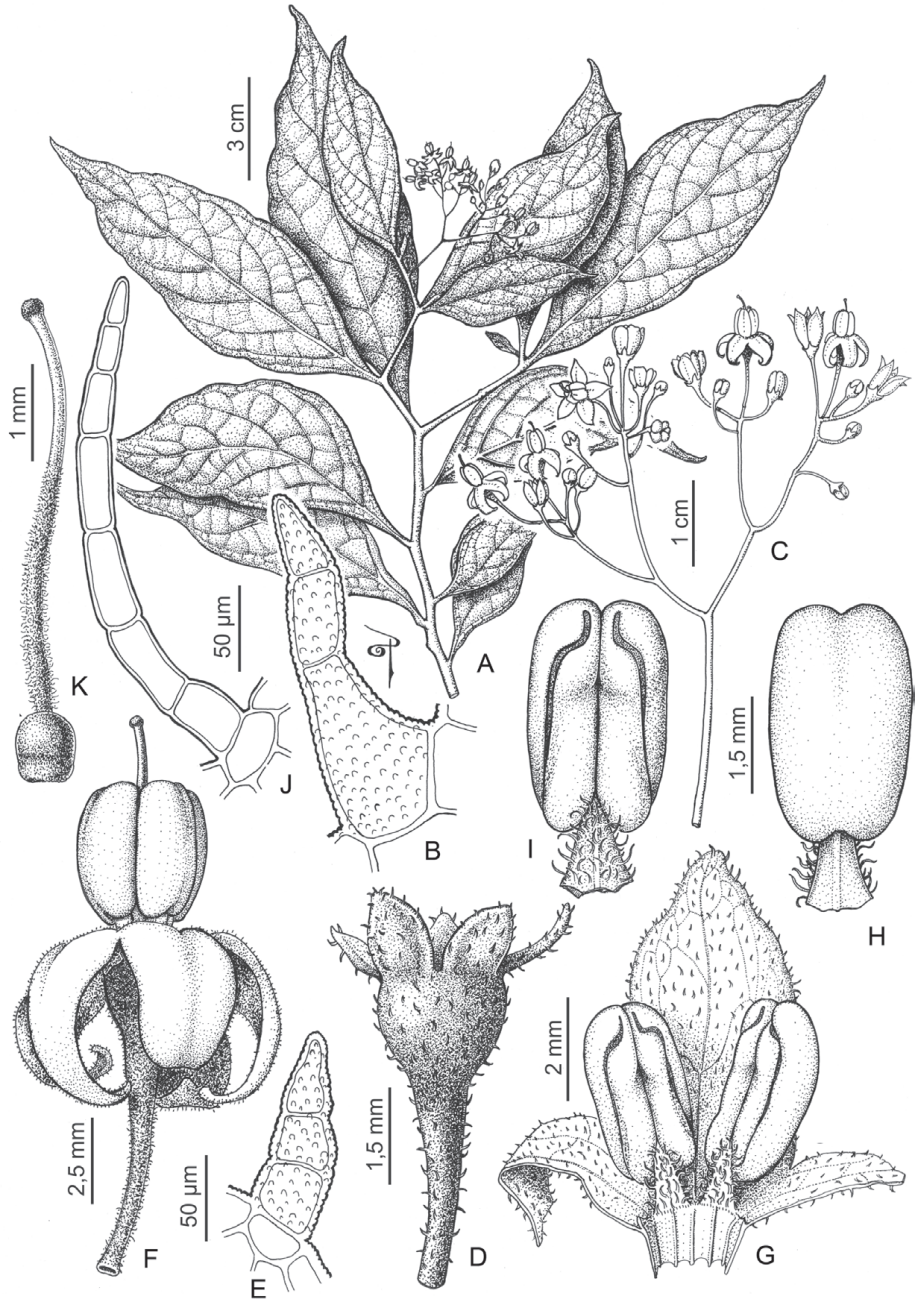
*Solanumhuayavillense***A** flowering branch **B** eglandular trichome of the leaf **C** inflorescence **D** calyx **E** eglandular trichome of the calyx **F** flower **G** sector of dissected flower **H** stamen, dorsal view **I** stamen, ventral view **J** eglandular trichome of the filament **K** gynoecium (**A–K***Barboza et al. 2255*). Illustration by P. Peralta. Previously published in Barboza et al. (2013: 250).

##### Description.

Erect perennial herbs or subshrubs 0.8–1 m high. Stems terete, glabrous to moderately pubescent with eglandular, simple uniseriate 2–6-celled trichomes to 0.7 mm long, these spreading and often moniliform, forming spinose processes on older stems; new growth moderately to densely pubescent with eglandular simple uniseriate trichomes 0.5–0.7 mm long; bark of older stems glabrescent, pale yellowish tan. Sympodial units difoliate, the leaves geminate, equal in size and shape, or one leaf of the pair slightly smaller. Leaves simple, the blades 4.5–15 cm long, 1.5–6 cm wide, elliptic to broadly elliptic, widest at the middle, membranous, concolorous; adaxial surfaces glabrous to sparsely and evenly pubescent with eglandular simple uniseriate trichomes to 1 mm long; abaxially glabrous to sparsely pubescent with eglandular simple uniseriate trichomes along the veins; principal veins 5–8 pairs, drying yellowish green on abaxially surfaces; base attenuate onto the petiole and the leaves sessile; margins entire, with sparse unicellular hooked trichomes ca. 0.1 mm long; apex acute to more commonly acuminate; petiole absent or occasionally to 0.2 cm long. Inflorescences internodal, several times branched, to 8 cm long, with 20 to 40 flowers, moderately pubescent with eglandular simple uniseriate trichomes to 0.5 mm (even if stems and leaves are glabrous the inflorescence is pubescent); peduncle 1–3 cm long; pedicels 0.6–0.7 cm, ca. 0.5 mm in diameter at the base, ca. 1.5 mm in diameter at the apex, tapering, deflexed to spreading at anthesis, sparsely to moderately pubescent with eglandular simple uniseriate trichomes like those of the rest of the inflorescence, articulated at the base; pedicel scars irregularly spaced 0.5–1.5 mm apart. Buds globose to broadly ellipsoid, the corolla strongly exserted from the calyx tube before anthesis. Flowers 5-merous, cosexual (hermaphroditic). Calyx tube 0.5–1 mm long, conical from the expanded apex of pedicel, the lobes 1–1.5 mm long, deltate with rounded tips, in live plants somewhat expanded and globose, pubescent with eglandular simple uniseriate trichomes like the pedicels and inflorescence axis. Corolla 0.9–1.2 cm in diameter, pale, clear yellow, stellate, lobed 3/4 of the way to the base, the lobes 4–5 mm long, 2–3 mm wide, reflexed at anthesis, glabrous adaxially, minutely papillate abaxially especially at the tips and along the margins. Stamens 5, equal; filament tube ca. 0.5 mm long; free portion of the filaments 0.25–0.5 mm long, densely pubescent with weak, tangled simple uniseriate trichomes adaxially; anthers 2.5–3 mm long, 1.5–1.75 mm wide, broadly ellipsoid, yellow, poricidal at the tips, the pores lengthening to slits with age. Ovary conical, glabrous; style 5.5–6.5 mm long, straight, exserted beyond the anther cone, densely pubescent in the lower half with simple 2–3-celled uniseriate trichomes; stigma minutely capitate, merely the slightly expanded style apex, minutely papillate, green in live plants. Fruit a globose berry, 0.4–0.6 cm in diameter, green when mature, the pericarp thin and translucent, matte, glabrous; fruiting pedicels 0.8–1.2 cm long, ca. 0.5 mm in diameter at the base, not markedly woody, deflexed or spreading, not persistent; fruiting calyx not accrescent, somewhat spreading. Seeds 6–8 per berry, 1–1.5 mm long, 1–1.5 mm wide, teardrop shaped but not markedly flattened, pale yellowish tan, the surfaces minutely pitted, the testal cells sinuate in outline. Stone cells absent (Argentina) or 4 (Bolivia), ca. 0.4 mm in diameter, 2 situated apically and 2 centrally. Chromosome number: not known.

**Figure 75.**
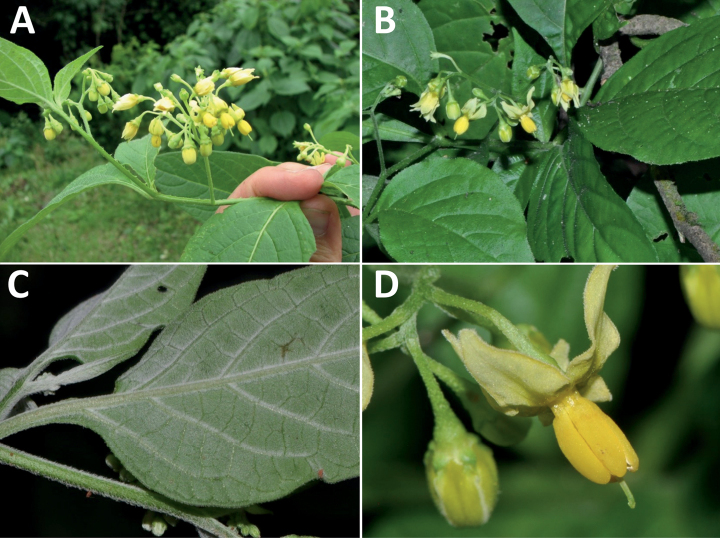
*Solanumhuayavillense***A** habit **B** habit **C** leaf pubescence (abaxial surface) **D** flowers at anthesis (**A, B–D***Barboza et al. 3531***C***Barboza et al. 3532*). Photos by S. Knapp.

##### Distribution

**Figure 76.**
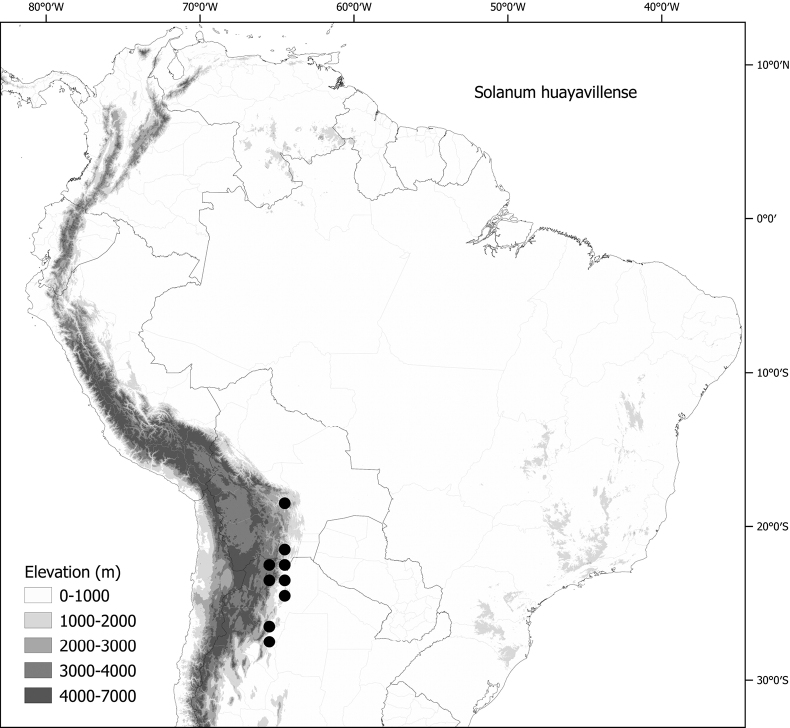
Distribution map of *Solanumhuayavillense*.

**(Fig. 76).***Solanumhuayavillense* is narrowly distributed in southern Bolivia (Depts. Chuquisaca, Tarija) and Argentina (Depts. Catamarca, Jujuy, Salta, Tucumán).

##### Ecology and habitat.

*Solanumhuayavillense* occurs in the understory of cloud forest (‘yungas’) either in the understorey proper or at edges of clearings or treefalls, from 1,200 to 2,950 m elevation.

##### Common names and uses.

None recorded.

##### Preliminary conservation status

**(IUCN 2022).** Least Concern [LC]. EOO = 80,000 km^2^ [LC]; AOO = 92 km^2^ [EN]. *Solanumhuayavillense* is relatively common where it is found in at least two protected areas in Argentina (Parque Nacional Baritú and Parque Nacional Calilegua).

##### Discussion.

*Solanumhuayavillense* is unique in the morelloid clade, and unusual in *Solanum* outside of the tomatoes, in having pale yellow, rather than white or violet, flowers. The yellow of *S.huayavillense* flowers is paler than that of the tomatoes (see Peralta et al. 2008) but still clearly yellow. It is not known whether this yellow is due to carotenoid or flavonoid content, as no chemical analysis has been undertaken. Vegetatively and in fruit *S.huayavillense* resembles *S.zuloagae* from slightly lower elevations in Argentina. The two species share lax growth, with long branches scrambling over adjacent vegetation, thin membranous leaves with sparsely ciliate margins and tiny flowers with short, stubby anthers. The flowers of *S.huayavillense* are slightly smaller than those of *S.zuloagae* (0.9–1.2 cm in diameter versus 1.2–1.8 cm in diameter) and differ in colour (yellow versus clear white). The calyx tube is longer than the lobes in *S.huayavillense* and somewhat urceolate (Fig. 75D), while in *S.zuloagae* the lobes are longer than the tube, and often unequal in size.

#### ﻿25. Solanum hunzikeri

Taxon classificationPlantaeSolanalesSolanaceae

Chiarini & Cantero, PhytoKeys 164: 40. 2020.

Figs 77 , 78

##### Type.

Argentina. Catamarca: Dpto. Ambato, Los Morteritos, Sierra de Ambato, falda E, subiendo desde El Rodeo hacia el Cerro Manchado [Cerro Manchao], 2,300–2,400 m, 13 Jan 1973, *A.T. Hunziker & R. Subils 22205* (holotype: CORD [CORD00013086]).

**Figure 77.**
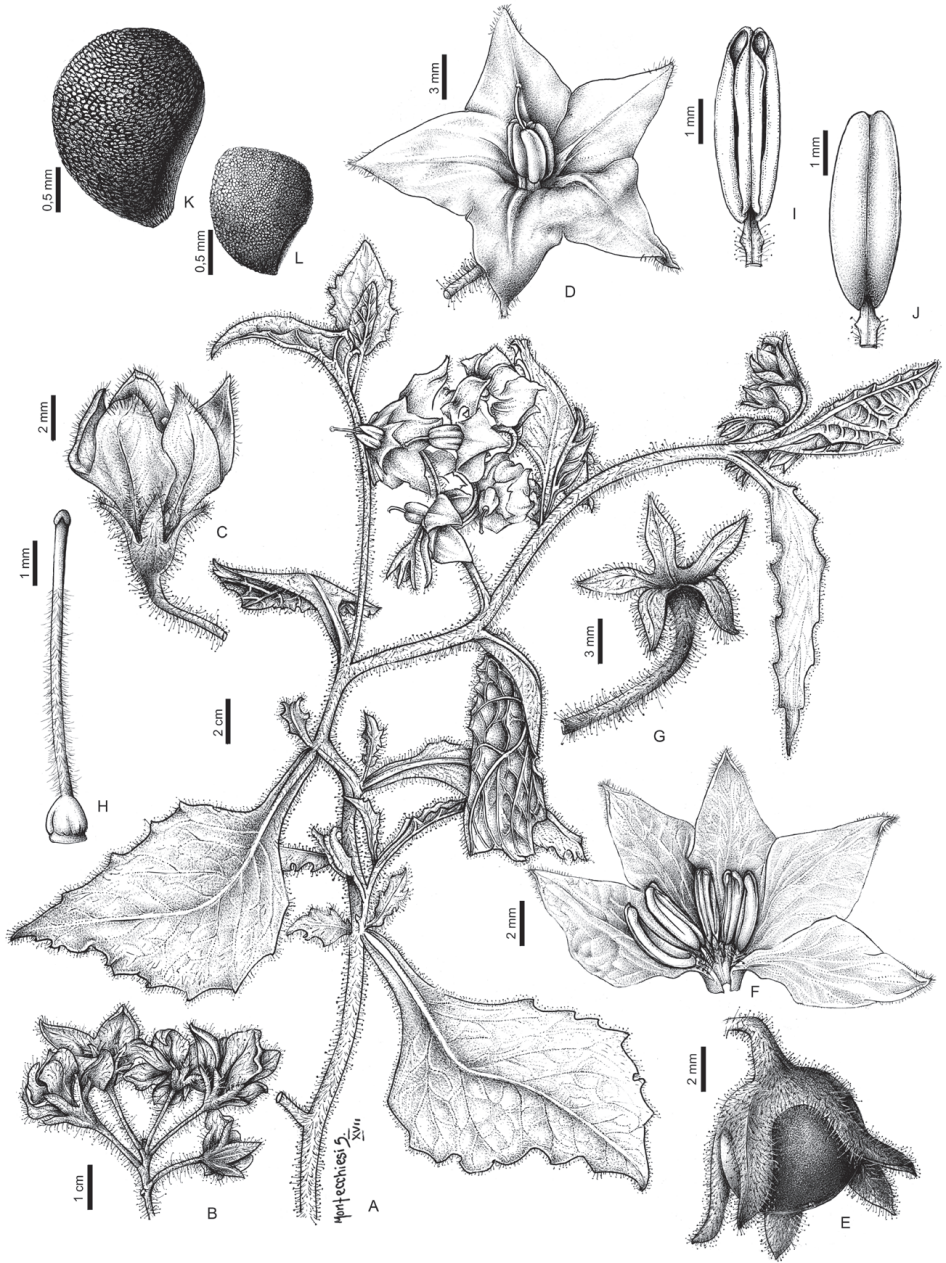
*Solanumhunzikeri***A** flowering stem **B** inflorescence **C** flower **D** open flower **E** immature fruit showing the accrescent calyx not completely covering the berry **F** flower showing pubescent adaxial surface of the filaments **G** calyx **H** gynoecium **I** stamen, ventral view **J** stamen, dorsal view **K** seed **L** stone cell (**A–D, F–J***Barboza 4763***E, K, L***Hunziker & Subils 22205*). Illustration by S. Montecchiesi. Previously published in Knapp et al. (2020: 41).

##### Description.

Herb or subshrub from a woody base ca. 0.5 m high. Stems terete or only slightly angled, densely glandular pubescent with glandular papillae and transparent spreading simple 3–8-celled uniseriate trichomes 0.5–1 mm long, some to 1.5 mm long; bark of older stems pale brown, glabrescent; new growth densely glandular pubescent with simple uniseriate trichomes to 1 mm long. Sympodial units plurifoliate, the leaves not geminate. Leaves simple, entire to shallowly toothed, the blades (2-)4.5–14 cm long, (1.1-)2–7 cm wide, elliptic in outline, widest at the middle, membranous or somewhat thick and fleshy, concolorous; adaxial surface moderately and evenly glandular pubescent with transparent spreading, simple uniseriate trichomes ca. 0.5 mm long on the lamina, ca. 1 mm long on the veins; abaxial surface moderately and evenly glandular pubescent like the adaxial surface, but the trichomes denser and longer, to 1.5 mm long; principal veins 4–7 pairs, densely glandular pubescent; base attenuate and strongly decurrent onto the petiole; margins entire or shallowly toothed, the teeth, if present, 1–2 mm long, 2–3 mm wide, broadly deltate with somewhat rounded tips; apex acute; petioles absent and the leaves sessile or 0–0.1 mm long, the decurrent leaf bases running onto the stem, glandular pubescent like the stems and leaves. Inflorescences opposite the leaves, unbranched but occasionally forked (*Rodríguez 1421*), 2.5–4 cm long, with 10–20 flowers, densely glandular pubescent with transparent spreading simple uniseriate trichomes to 1.5 mm long; peduncle 1.2–2.5 cm long; pedicels 1.3–1.5 cm long, 0.5–0.7 mm in diameter at the base, ca. 1.5 mm in diameter at the apex, spreading at anthesis, densely glandular pubescent, articulated at the base; pedicel scars irregularly spaced 1–2 mm apart. Buds ellipsoid, the corolla ca. halfway exserted from the calyx before anthesis. Flowers 5-merous, cosexual (hermaphroditic). Calyx tube 2–3 mm long, conical, the lobes 2.5–4 mm long, long-triangular, densely glandular pubescent with simple uniseriate trichomes like the pedicels and rest of the inflorescence, the tips acuminate and somewhat recurved at anthesis. Corolla 1.6–2.5 cm in diameter, pale lilac to violet with a yellow-green central star, stellate, lobed ca. 1/2 way to the base, the lobes 5–5.5 mm long, 4–5.5 mm wide, deltate, reflexed or spreading at anthesis, adaxially glabrous, abaxially sparsely glandular papillate especially on the midvein, tips and margins; stamens equal; filament tube 0.35–0.5 mm; free portion of the filaments 1–1.5 mm, almost glabrous, but with a few tangled transparent eglandular simple uniseriate trichomes adaxially; anthers 4–5.5 mm long, 1.25–1.6 mm wide, ellipsoid, yellow, poricidal at the tips, the pores lengthening to slits with age. Ovary conical, glabrous; style 7–8 mm long, straight, exserted beyond the anther cone, densely papillate with a few longer simple trichomes in the lower third; stigma large capitate to slightly bilobed, the surface minutely papillate. Fruit a globose berry, 1–1.2 cm in diameter, green (?) at maturity, the pericarp glabrous, thin, matte, opaque, glabrous; fruiting pedicels 1.5–2 cm long, ca. 1.5 mm in diameter at the base, ca. 2 mm in diameter at the apex, somewhat woody, deflexed from the weight of the berry, glandular pubescent to somewhat glabrescent, not persistent; fruiting calyx accrescent in young fruit tightly investing the berry, the tube 3–5 mm long, later tearing and the berry exposed, the lobes 3–5 mm long, ca. 3 mm wide, appressed to spreading. Seeds ca. 40 per berry, 1.5–2 mm long, 1–1.7 mm wide, flattened and teardrop shaped with an apical hilum, reddish brown, the surfaces minutely pitted, testal morphology not clearly seen. Stone cells 10–11 per berry, 1–1.3 mm in diameter, globose, scattered throughout the berry. Chromosome number not known (but see comments on DNA content below).

**Figure 78.**
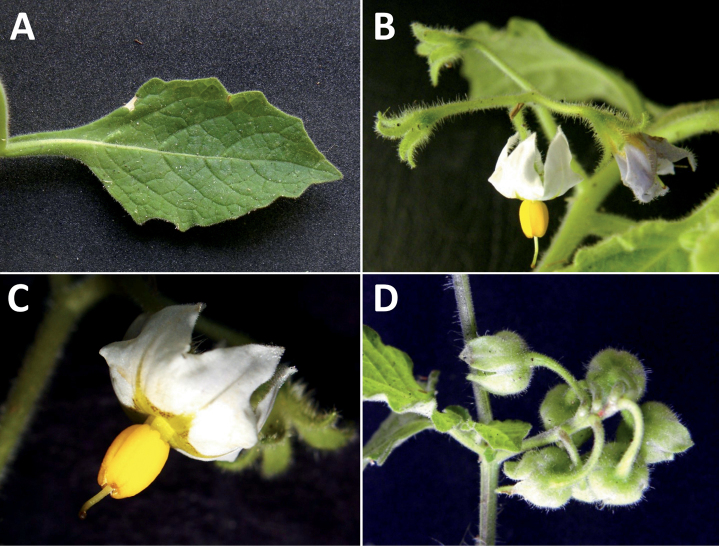
*Solanumhunzikeri***A** leaf (adaxial surface) showing attenuate base **B** inflorescence **C** flower at anthesis **D** maturing infructescence with fruits enclosed in the enlarged calyx (**A–D***Barboza et al. 4763*). Photos by M. Gritti. Previously published in Knapp et al. (2020: 39).

##### Distribution

**Figure 79.**
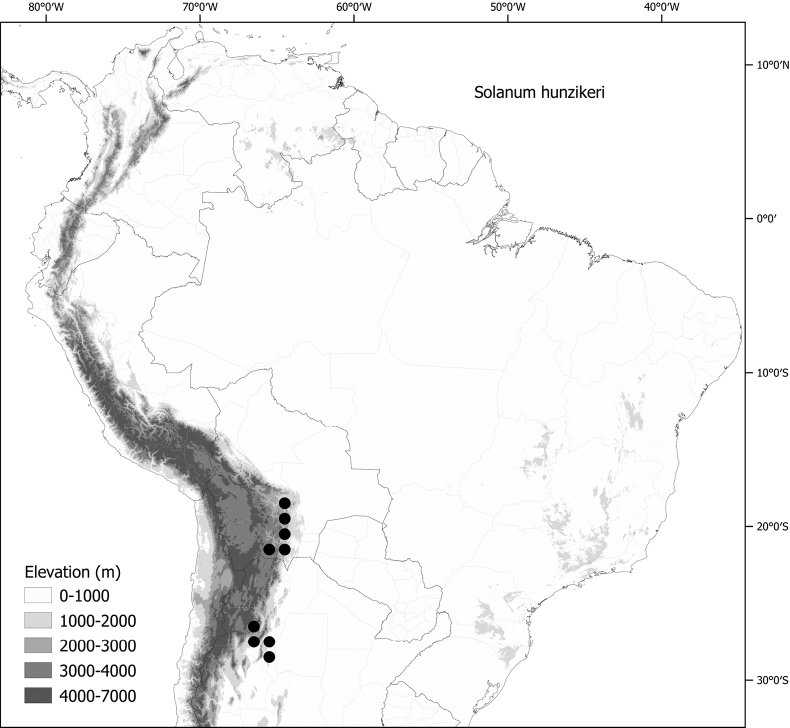
Distribution map of *Solanumhunzikeri*.

**(Fig. 79).***Solanumhunzikeri* occurs in Argentina (Provs. Catamarca and Tucumán) and north to Bolivia (Depts. Chuquisaca and Tarija). The somewhat disjunct distribution is possibly due to loss or lack of the wet high elevation foggy grassland habitat in intervening areas. Most Argentine collections are from the Ambato area of endemism of Aagensen et al. (2012).

##### Ecology and habitat.

*Solanumhunzikeri* is confined to wet cloud forests and foggy grasslands above 1,800 m elevation; it also grows in the ecotones between these vegetation types.

##### Common names and uses.

None recorded.

##### Preliminary conservation status

**(IUCN 2022).** Near Threatened [NT]. EOO = 97,182 km^2^ [LC]; AOO = 80 km^2^ [EN]. Although the large extent of occurrence would suggest *S.hunzikeri* is not of conservation concern, the limited number of localities (<10), the specialised habitat and the disjunct distribution suggest the species should be considered as under some threat. *Solanumhunzikeri* occurs in a very restricted habitat in which there are few officially protected areas. In these landscapes the main threat to the ecosystem is over-grazing; the introduction of alien forage species has severely altered the nature of the high elevation foggy grasslands and forest edges in which *S.hunzikeri* occurs. Some populations are found in currently protected areas such as the Parque Nacional Aconquija, but these areas are considered too small and isolated to provide long term conservation (Brown 1995). Knapp et al. (2020) assigned a preliminary threat status of Vulnerable (VU, B2a,b(iii)) for *S.hunzikeri*. The exploration of these relatively inaccessible habitats in the area between the currently known populations of *S.hunzikeri* is a priority.

##### Discussion.

*Solanumhunzikeri* was long recognised as distinct from other glandular-pubescent species in Argentina but has only recently been named (Knapp et al. 2020) when additional specimens allowed us to clarify its differences from the widespread and highly variable *S.tweedieanum*. *Solanumhunzikeri* can be distinguished from *S.tweedieanum* populations in similar high elevation areas in its strongly attenuate and winged leaf bases; those of *S.tweedieanum* are more truncate. The single collection we have seen of *S.hunzikeri* with mature fruit (*Rodríguez 1421* from Salta) has the calyx not covering any part of the mature berry; berries of *S.tweedieanum* are tightly covered by the accrescent calyx for at least 50% of their length. Berries of *S.tweedieanum* are pale cream when ripe, while berry colour in *S.hunzikeri* is not known. More collections of *S.hunzikeri* in fruit are needed to assess these differences. Preliminary data on DNA content suggest that, like *S.tweedieanum* (Moscone 1992), *S.hunzikeri* is diploid (F. Chiarini unpubl.).

#### ﻿26. Solanum interandinum

Taxon classificationPlantaeSolanalesSolanaceae

Bitter, Repert. Spec. Nov. Regni Veg. 11: 217. 1912.

Figs 80 , 81

Solanum onagrifolium Bitter, Repert. Spec. Nov. Regni Veg. 11: 216. 1912. Type. Ecuador. “crescit in tota altiplan. Frequens”, *A. Sodiro, A. 114/12* (holotype B, destroyed [F neg. 2677]; lectotype, designated here: QPLS).
Solanum egranulatum Bitter, Repert. Spec. Nov. Regni Veg. 11: 217. 1912. Type. Ecuador. “In altiplanitie interandina”, *A. Sodiro 114/12 pro parte* (holotype: B, destroyed [F neg. 2660, of sheet annotated as Solanumegranulosum by Bitter]; lectotype, designated here: QPLS).
Solanum densepilosulum Bitter, Repert. Spec. Nov. Regni Veg. 11: 218. 1912. Type. Ecuador. Sin. loc. “in tota altiplanitie passim una cum S.onagrifolium, S.interandinum, S.egranulatum sub nom. “S.pterocaulon Dunal” a cl. Sodiro lectum herb. Berol.”, *A. Sodiro* (no specific collection cited).
Solanum soriae Heiser, Ci. & Naturaleza [Quito] 6: 57. 1963. Type. Ecuador. Pichincha: Lloa, in fence row by stream, *C.B. Heiser 5093* (holotype: IND [IND1000062]; isotype: IND [IND1000061]).
Solanum pentlandii Dunal subsp. interandinum (Bitter) Edmonds, Kew Bull. 27: 110. 1972. Type. Based on Solanuminterandinum Bitter.
Solanum melanostictocarpum Gilli, Repert. Spec. Nov. Regni Veg. 94: 321. 1983. Type. Ecuador. Chimborazo: Rain bei Cuatras Esquinas NO von Guaranda, *A. Gilli 97* (holotype: W [acc. # 1981-0011277]).
Solanum zahlbruckneri Bitter, Repert. Spec. Nov. Regni Veg. 11: 203. 1912. Type. Peru. Cajamarca. Cutervo, *C. de Jelski 46* (holotype: W [acc. # 1891-0004329]; isotypes: F [v0043302F, acc. # 871534, fragment of holotype], MO [MO-3008928, acc. # 1691555], S [acc. # 04-2998]).

##### Type.

Ecuador. “In tota altiplanitie passim, communissima in altiplanitie interandina”, *A. Sodiro 114/12 pro parte* (holotype: B, destroyed; lectotype, designated here: QPLS).

**Figure 80.**
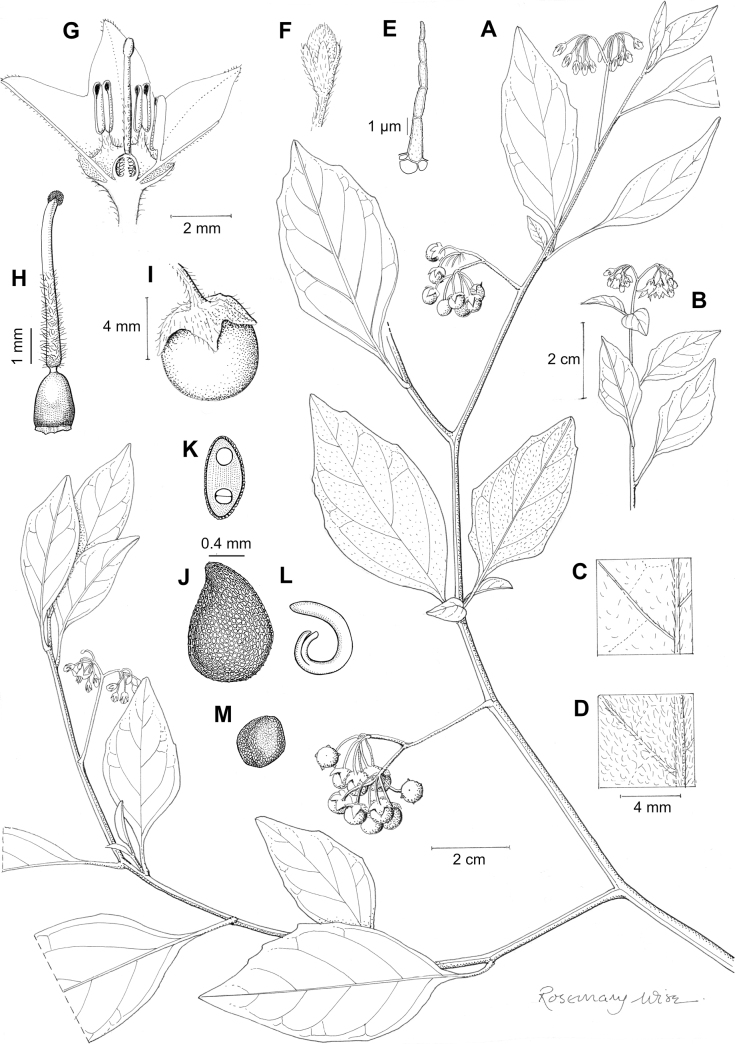
*Solanuminterandinum***A** habit **B** flowering habit with smaller leaves **C** detail of adaxial leaf surface **D** detail of abaxial leaf surface **E** eglandular multicellular trichome on stem and leaves **F** flower bud **G** dissected flower **H** gynoecium **I** fruit **J** seed **K** seed, cross section **L** embryo **M** stone cell (**A, C, D, F, G, I***Fosberg 20565***B***Hitchcock 21006***E, H, J–M***Cabrera 13875*). Illustration by R. Wise and M.T. Cabrera.

##### Description.

Small shrubs or occasionally woody herbs to 1 m high, the branches erect, always woody at base. Stems terete or angled, if angled the wings to 1 mm wide and with spinescent processes, sparsely pubescent with white eglandular simple uniseriate 2–7-celled trichomes 0.5–0.75 mm long, these usually antrorse; new growth densely to moderately pubescent with the same simple uniseriate trichomes as those of the stems; bark of older stems brown, glabrescent. Sympodial units difoliate to plurifoliate, the leaves usually not geminate, but sometimes appearing paired. Leaves simple, occasionally shallowly lobed, the blades 1.8–7.5(12) cm long, 0.8–4(5.5) cm wide, narrowly elliptic to elliptic, sometimes slightly ovate, widest in the lower third, membranous, discolorous; adaxial surfaces sparsely to moderately pubescent with usually transparent antrorse eglandular simple uniseriate trichomes 0.5–1 mm long; abaxial surfaces densely pubescent with tangled white eglandular simple uniseriate trichomes 0.5–1 mm long; principal veins 5–7 pairs, obscured by pubescence below in dry specimens; base attenuate onto the petiole; margins entire; apex acute; petioles 0.3–2 cm long, narrowly winged from the leaf bases. Inflorescences internodal or opposite the leaves, forked or several times branched, 1.5–5(7) cm long, with 10–20 flowers densely or loosely clustered at the branch tips, pubescent with white eglandular simple uniseriate 2–7-celled trichomes 0.5–0.75 mm long, these usually antrorse; peduncle 1–5 cm long; pedicels 0.4–0.6 cm long, ca. 0.5 mm in diameter at the base, ca. 0.75 mm in diameter at the apex, slightly tapering, spreading at anthesis, pubescent with white eglandular simple uniseriate trichomes like the rest of the inflorescence, articulated at the base; pedicel scars 0–1.5 mm apart in the distal part of the inflorescence branches. Buds ellipsoid, the corolla strongly exserted from the calyx before anthesis, the style often exserted in bud, the buds appearing striped in live plants. Flowers 5-merous, cosexual (hermaphroditic). Calyx tube 0.5–1.2 mm long, conical from the tapered pedicel, the lobes ca. 1 mm long, 0.75–1 mm wide, deltate to broadly triangular with acute apices, pubescent with antrorse white eglandular simple uniseriate 2–7-celled trichomes 0.5–0.75 mm long. Corolla 0.8–1.4(1.8) cm in diameter, pale violet or white and violet striped or violet abaxially, stellate, lobed 2/3 to 1/2 way to the base, the lobes 4–6 mm long, 3–4 mm wide, deltate, spreading or reflexed, adaxially glabrous, abaxially evenly papillate-puberulent with simple trichomes to 0.2 mm long, these denser at the tips and margins. Stamens equal; filament tube minute; free portion of the filaments 0.5–1 mm long, densely pubescent with tangled transparent simple uniseriate trichomes adaxially; anthers 2.5–3 mm long, 1–1.4 mm wide, ellipsoid, yellow, poricidal at the tips, the pores lengthening to slits with age. Ovary conical, glabrous; style 5.5–7 mm long, straight, often curved in bud, exserted beyond the anther cone, densely puberulent in the lower half or 2/3; stigma small capitate, the surfaces minutely papillose. Fruit a globose berry, 0.6–0.8 cm in diameter, whitish green when immature, ripening to green or green with irregular black-purple blotches, the pericarp thin, matte, opaque, glabrous; fruiting pedicels 0.9–1 cm long, ca. 1 mm in diameter at the base, ca. 1.2 mm in diameter at the apex, woody, strongly deflexed, not persistent, but peduncle and inflorescence branches persistent on older stems; fruiting calyx not markedly enlarged, the lobes 2–2.5 mm long, ca. 1.8 mm wide, strongly appressed to the berry. Seeds 30–60 per berry, ca. 1.5 mm long, 1.1–2 mm wide, flattened and teardrop shaped to reniform, pale reddish brown or cream, the surfaces minutely pitted, the testal cells sinuate in outline. Stone cells 6–10 per berry, ca. 0.5 mm in diameter, cream-coloured, distributed throughout the berry. Chromosome number: n = 12 (Heiser 1963, vouchers *Heiser 4920*, *4922*, *4973*, *5085*, *5093*, *6037*, *6061*, *1697*, as *S.soriae*); n = 24 (Heiser 1963, vouchers *Heiser 4962a*, *4978*, *5084*, *6017*, *6020*, *6021*, *6051*, *6068*); n = 48 (number written on herbarium vouchers *Heiser 4993*, *5081*, *6081*).

**Figure 81.**
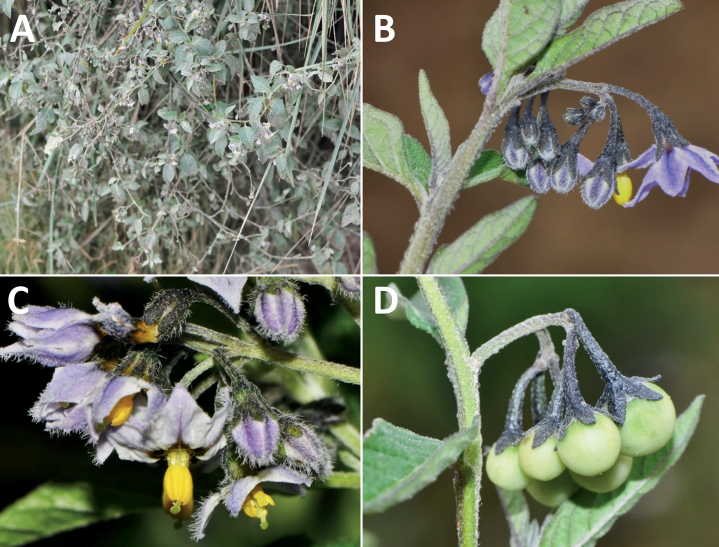
*Solanuminterandinum***A** habit **B** inflorescence with bud **C** flowers at full anthesis **D** maturing fruits (**A***Särkinen et al. 4693***B–D***Knapp et al. 10259*). Photos by S. Knapp and T. Särkinen.

##### Distribution

**Figure 82.**
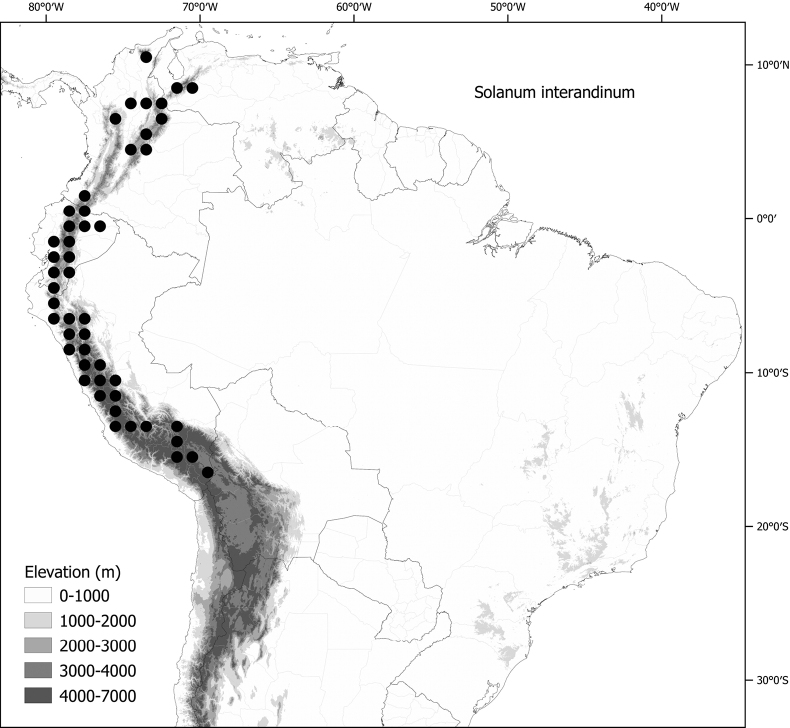
Distribution map of *Solanuminterandinum*.

**(Fig. 82).***Solanuminterandinum* is a common species and is widely distributed in the Andes in Colombia (Antioquia, Boyacá, Cauca, Cundinamarca, Huila, La Guajira, Magdalena, Nariño, Norte de Santander, Putumayo, Santander, Valle de Cauca), Venezuela (States of Mérida, Táchira), Ecuador (Azuay, Bolívar, Cañar, Carchi, Chimborazo, Cotopaxi, Imbabura, Loja, Los Rios, Manabí, Napo, Pastaza, Pichincha, Tungurahua) to central Peru (Depts. Amazonas, Ancash, Arequipa, Apurímac, Ayacucho. Cajamarca, Cusco, Huancavelica, Huánuco, Junín, La Libertad, Lima, Piura, Puno, San Martín), with a southerly collection from Bolivia (Dept. La Paz, *Plowman & Davis 5135*).

##### Ecology and habitat.

*Solanuminterandinum* grows in open areas in high elevation cloud forests and forest margins (‘ceja de selva’); most collections have been made between 1,000 and 4,000 m elevation, but a few collections from the western slopes of the Andes in both Ecuador and Peru have been made at lower elevations (50 to 600 m).

##### Common names and uses.

Colombia. Caldas: yerba mora (*Grisales 9*); Putumayo: yerbamora (*Criollo 16*); Ecuador. Azuay: hierba mora (*Cerón 16328*), moradilla (*Cerón 115617*), mortiño (*Cerón 15277, 15323*, *16051*, *16328*, *16351*); Bolivar: hierba mora (*Argüello 118*, *Falconi & Argüello 79*); Cañar: hierba mora (*Cerón 14476*, *14957*, *16076*, *16459*, *17636*), mortiña (*Camp & Prieto E-2467*, *Cerón 14857*); Carchi: hierba mora (*Cerón 6974*); Chimborazo: hierba (yerba) mora (*Caranqui 1244*, *Cerón 14651*, *14716*, *15198*, *15377*, *16478*, *17458*, *Cerón & Gallo 19626*); Cotopaxi: yerba (hierba) mora (*Barclay & Juajibioy 8038*, *Cerón 7087*, *Cerón et al. 11617*); Imbabura: hierba mora (*Bailey 73*, *Cerón & Montesdeoca 12527*); Manabí: yerba mora, pili muyo (*Prácticas de Recolección s.n.*); Napo: huami hierba mora, yerba mora embra (*Baez et al. 31*); Pichincha: hierba mora (*Ugent & Ugent 5579*, *Cerón 6841*, *Chiriboga Q. 27*, *Mantilla 41*, *Mena et al. 466*, *Paredes 145*, *Prácticas de Recolección s.n., 32, Putcher* 55, *128*, *251*, *Vargas N. s.n.*), pili muyo (*Prácticas de Recolección 32*); Tungurahua: hierba (yerba) mora (*Delgado et al. 177*, *Lligado 51*, *Paredes s.n.*), jachafili (Quichua, *Lligado 51*); Peru. Puno: muña mayo (*Hoogte 3836*). Venezuela. Mérida: yerba mora (*Gehriger 15*). No uses recorded.

##### Preliminary conservation status

**(IUCN 2022).** Least Concern [LC]. EOO = 13,454,357 km^2^ [LC]; AOO = 1,148 km^2^ [EN]. *Solanuminterandinum* is a common, widely distributed species that occupies disturbed and open habitats. It is found in several protected areas within its range (e.g., common in Parque Nacional Huascarán, Peru).

##### Discussion.

*Solanuminterandinum* is one of the commonest species of morelloids in the northern Andes and is called hierba mora (black nightshade) throughout its range. It is highly variable, as are most widespread morelloid species (see discussion of synonymy below); plants with the flowers clustered at the tips of the inflorescence branches have been called *S.interandinum* whereas those with the flowers more spaced along the inflorescence axes have been called *S.zahlbruckneri*.

*Solanuminterandinum* is a small shrub where the old, forked inflorescences remain on the plant long after the fruits have fallen (along with their pedicels). The leaf margins are usually entire and only rarely with some shallow lobing near the base and leaf size is highly variable within and between individual plants. It is morphologically similar to *S.nigrescens* in northern Colombia and Venezuela, *S.cochabambense* in Peru and *S.gonocladum* in Peru and Bolivia. *Solanumnigrescens* is a more herbaceous plant, with an unbranched (very occasionally forked) inflorescence that is not woody and persistent after fruit fall. Anthers of *S.interandinum* are longer (2.5–3 mm long on filaments 0.5–1 mm long versus 2–2.8 mm long on filaments 0.5–2 mm long) relative to the filaments than those of *S.nigrescens*, and the calyx lobes are more than 1 mm long and long-triangular rather than less than 1 mm long and deltate to broadly deltate in *S.nigrescens*. *Solanumcochabambense* is a much larger plant, with more branched (rather than always forked) inflorescences and larger flowers (2–3 cm in diameter with anthers 3.5–4.5 mm long versus 1–1.2 cm in diameter with anthers 2–3 mm long in *S.interandinum*). In central Peru it can be very difficult to tell these two species apart with single herbarium specimens, and it is possible they hybridise. In northern Bolivia and southern Peru *S.interandinum* is somewhat confusable with *S.gonocladum*, but that species has larger flowers (1.3–2 cm in diameter with anthers 4–4.5 mm long versus 1–1.2 cm in diameter with anthers 2–3 mm long in *S.interandinum*) and spathulate (rather than apically pointed) calyx lobes. Both these species have calyx lobes that dry dark in herbarium specimens.

Bitter (1912b) appears to have used different duplicates of *Sodiro 114/12* in Berlin to describe *S.interandinum*, *S.egranulatum*, *S.onagrifolium* and (probably) *S.densepilosum*. The only Macbride photographs of any of these are of sheets annotated by Bitter as *S.onagrifolium* (F neg. 2677) and *S.egranulatum* (F. neg. 2660). These clearly correspond to the same species, if not the same gathering. Bitter often used duplicates of the same gathering as the basis for both infraspecific and specific epithets based on minor differences in leaf shape (e.g., *S.ruizii* S.Knapp, see Knapp 2013; *S.gonocladum* in this monograph); *S.interandinum*, however, is the most extreme case we have ever encountered. We have only found a single sheet of *Sodiro 114/12* in QPLS, which we have designated as the lectotype of all these names (see above), except *S.densepilosum* which was characterised as “in tota alta planitie passim una cum S.onagrifolium, S. internadinum, S.egranulatum sub nom. “S.pterocaulon Dun.” a cl. Sodiro lectum herb. Berol” (Bitter 1912b). *Solanumdensepilosum* is distinguished as a species with intermediate sized anthers, and with enlarging fruiting calyces. Based on description alone, it is hard to assign the name to any existing species with certainty, but it fits within the circumscription of *S.interandinum* here. Because the original description lacks any specific locality, there is little possibility of re-collecting type material. We place it in synonymy here, but without designating a neotype. Of all of these simultaneously published names, *S.interandinum* is the only one that has been previously used (Edmonds 1972; Short and Knapp 1999), so we reduce the others to synonymy.

#### ﻿27. Solanum juninense

Taxon classificationPlantaeSolanalesSolanaceae

Bitter, Bot. Jahrb. Syst. 54, Beibl. 119: 11. 1916.

Figs 2C , 83 , 84

##### Type.

Peru. Junín: Cerca de Huancayo, 11 Apr 1913, *A. Weberbauer 6598* (no herbaria cited; lectotype, designated here: MOL [MOL00005056]; isolectotypes: B, destroyed [F neg. 2613], F [v0043244F, acc. # 627963; v0043245F, acc. # 847835, fragment of specimen from B], MOL [MOL00005057, MOL00005058], US [00027639, acc. # 1473478; 01014171, acc. # 1444708]).

**Figure 83.**
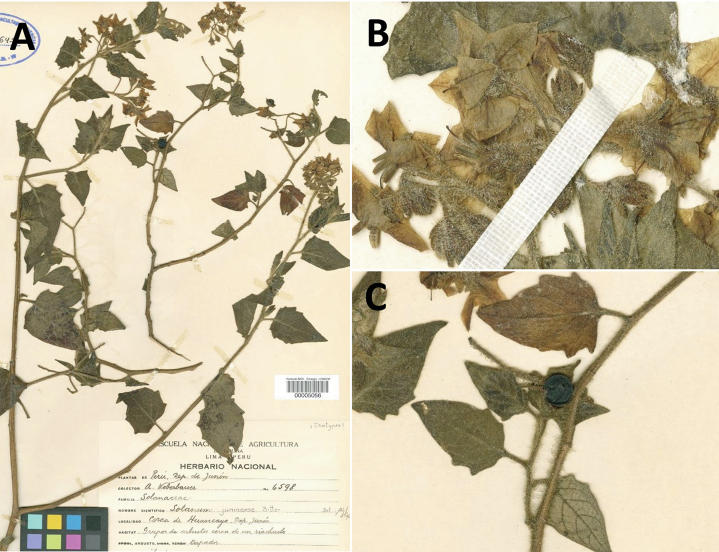
*Solanumjuninense***A** habit **B** inflorescence with buds **C** developing fruit (**A, C***Weberbauer 6598* [MOL00005056] **B***Weberbauer 6598* [vF0043244F]). Reproduced with permission of the Field Museum of Natural History and the Universidad Nacional Agraria La Molina.

##### Description.

Scrambling shrubs or woody herbs to 1 m high, the branches lax and supported by other vegetation. Stems terete, densely pubescent with transparent glandular 6–8-celled simple uniseriate trichomes to 2 mm long, the gland single-celled, globose; new growth densely pubescent with sessile glands and transparent glandular 6–8-celled simple uniseriate trichomes to 2 mm long; bark of older stems pale yellowish green or brown, glabrescent. Sympodial units plurifoliate, the leaves not geminate. Leaves simple, occasionally shallowly toothed, the blades 2.5–8 cm long, 0.8–5.3 cm wide, elliptic, elliptic-ovate or narrowly ovate, widest in the lower half, membranous, slightly discolorous; adaxial surfaces sparsely to moderately pubescent with transparent glandular simple uniseriate trichomes 1–2 mm long; abaxial surfaces glabrous to sparsely glandular-pubescent on the lamina, densely pubescent with transparent glandular simple uniseriate trichomes 1–2 mm long along the veins; principal veins 5–6 pairs, densely glandular-pubescent abaxially; base acute to cuneate; margins entire to undulate or very shallowly and irregularly toothed, if present the teeth 1–2 mm long, 1–2 mm wide, rounded at the tips, the sinuses rounded and reaching to less than 1/10 of the way to the midrib; apex acute to acuminate; petioles 0.5–3.5 cm long, moderately to densely pubescent with transparent glandular 6–8-celled simple uniseriate trichomes to 2 mm long, the gland single-celled. Inflorescences terminal at branch tips or more rarely internodal, forked or very occasionally more branched, 3–5 mm long, with (4)10–20 flowers clustered in the distal half of the branches, densely pubescent with transparent glandular 6–8-celled simple uniseriate trichomes to 2 mm long, the gland single-celled; peduncle 1–3 cm long; pedicels 0.5–0.8 cm long, ca. 0.5 mm in diameter at the base, ca. 0.75 mm in diameter at the apex, slightly tapering, spreading at anthesis, pubescent with transparent glandular 6–8-celled simple uniseriate trichomes like those of the inflorescence axes, articulated at the base, leaving a small raised bump; pedicel scars irregularly spaced 1–2 mm apart, slightly raised. Buds ellipsoid, the corolla strongly exserted from the calyx before anthesis. Flowers 5-merous, cosexual (hermaphroditic). Calyx tube 1–1.5 mm long, conical, the lobes 1.5–2 mm long, ca. 1 mm wide, triangular with acute tips, pubescent with transparent glandular 6–8-celled simple uniseriate trichomes ca. 1 mm long, the gland single-celled. Corolla 1.2–1.5 cm in diameter, white, pale lilac to deep purple, with a darker purple or greenish purple eye, stellate, lobed 1/3 to 1/2 of the way to the base, the lobes 4–5 mm long, 3.5–7 mm wide, broadly deltate, spreading at anthesis, adaxially glabrous, abaxially densely pubescent with transparent mixed eglandular and glandular simple uniseriate trichomes to 0.5 mm long, densely papillate on tips and margins. Stamens equal; filament tube minute; free portion of the filaments 1.5–2 mm long, glabrous or sparsely pubescent with tangled transparent simple uniseriate trichomes abaxially; anthers 3–3.5 mm long, ca. 1 mm wide, ellipsoid, yellow, poricidal at the tips, the pores lengthening to slits with age. Ovary conical, glabrous; style 5–7 mm long, straight, exserted beyond the anther cone, densely glandular-pubescent in the lower half with transparent trichomes; stigma small-capitate, the surface minutely papillate. Fruit a globose berry, 0.7–0.9 cm in diameter, greenish purple or dark green when ripe, the pericarp thin, shiny, translucent, glabrous; fruiting pedicels 0.8–0.9 cm long, ca. 1 mm in diameter at the base, ca. 1.5 mm in diameter at the apex, not markedly woody, spreading or deflexed, not persistent; fruiting calyx not markedly enlarged or accrescent, the lobes to 2.5 mm long, spreading or appressed to the berry. Seeds 40–50 per berry, ca. 2 mm long, ca. 1.5 mm wide, flattened and teardrop shaped, pale straw-coloured, the surfaces minutely pitted, the testal cells rectangular in outline with thick walls. Stone cells (1)2 per berry, scattered in mesocarp, 0.75–1 mm in diameter, cream-coloured. Chromosome number: 2n = 24 (Chiarini et al. 2017, voucher *Särkinen et al. 4754*).

**Figure 84.**
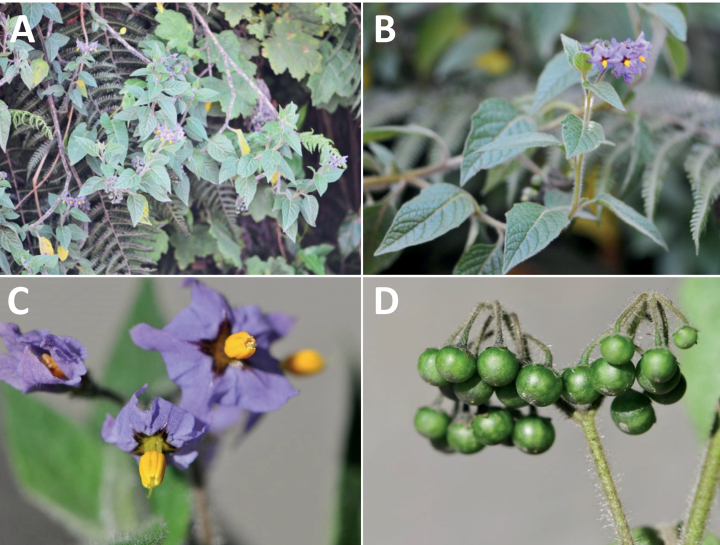
*Solanumjuninense***A** habit **B** flowering branch **C** flowers at anthesis **D** maturing fruits (**A–D***Särkinen et al. 4754*). Photos by T. Särkinen.

##### Distribution

**Figure 85.**
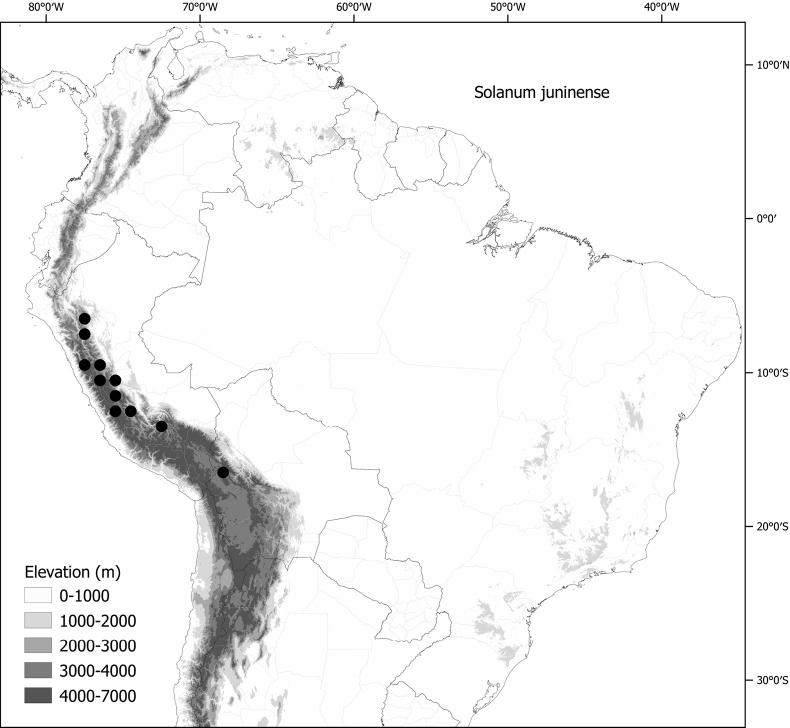
Distribution map of *Solanumjuninense*.

**(Fig. 85).***Solanumjuninense* occurs in the Andes of Peru (Depts. Amazonas, Ancash, Ayacucho, Cajamarca, Huancavelica, Junín, La Libertad, Pasco, San Martin) and Bolivia (a single collection from Dept. La Paz, *Solomon 16463*). We expect to see more collections in the future from southern Peru and northern Bolivia, but currently there is a disjunction between the Peruvian and Bolivian populations.

##### Ecology and habitat.

*Solanumjuninense* grows in cloud forests and cloud forest margins (‘ceja de selva’), often in open roadsides, treefalls and along streams ditches and moist depressions, from 1,800 to 4,200 m elevation.

##### Common names and uses.

Peru. Junín: hierba mora (*Marcelo Peña et al. 1895*). No uses recorded.

##### Preliminary conservation status

**(IUCN 2022).** Least Concern [LC]. EOO = 197,081 km^2^ [LC]; AOO = 120 km^2^ [EN]. *Solanumjuninense* has a relatively wide distribution and is found in at least two protected areas within its range (e.g., Parque Nacional Abiseo and Parque Nacional Yanachaga-Chemillén, Peru).

##### Discussion.

*Solanumjuninense* is one of the few sticky-pubescent morelloids (with *S.arenicola* and *S.subtusviolaceum*) without an accrescent calyx. It is most similar to *S.subtusviolaceum*, and both can be distinguished from other glandular-pubescent species by their non-accrescent fruiting calyces and highly branched inflorescences. *Solanumarenicola* is a plant of the Amazonian foothills, while both *S.juninense* and *S.subtusviolaceum* are Andean taxa. *Solanumjuninense* differs from *S.subtusviolaceum* in its plurifoliate sympodia with elliptic to narrowly elliptic leaves (versus unifoliate or difoliate sympodia with ovate to rhomboid leaves), acute to cuneate leaf base (versus truncate in *S.subtusviolaceum*), with shorter calyx lobes (1.5–2 mm long versus 2.5–3.5 mm long and sometimes toothed), slightly smaller corollas (1.2–1.5 cm in diameter versus 1.8–2 cm in diameter) with deltate rather than triangular lobes and one or two stone cells (versus four in *S.subtusviolaceum*) in each berry.

Bitter (1916) described *S.juninense* citing a collection of August Weberbauer (*Weberbauer 6598*) but no herbarium. We select here the best preserved of the duplicates held in the herbarium of the Universidad Nacional Agraria La Molina (MOL00005056) as the lectotype. Weberbauer’s original personal herbarium is held in MOL.

#### ﻿28. Solanum leptocaulon

Taxon classificationPlantaeSolanalesSolanaceae

Van Heurck & Müll.Arg., Observ. Bot. 40. 1870.

Figs 86 , 87

Solanum rheithrocharis Bitter, Repert. Spec. Nov. Regni Veg. 13: 91. 1914. Type: Bolivia. Cochabamba: “aplinen region oberhalb Incacorral”, ca. 3,200 m, Jan 1908, *T. Herzog 806* (lectotype, designated here: Z [Z-000229529]; isolectotype: L [L 0403634]).).
Solanum pongoense Rusby, Mem. New York Bot. Gard. 7: 348. 1927. Type. Bolivia. La Paz: Pongo de Quime, 5 Jul 1921, *O.E. White 165* (holotype: NY [00172138]; isotype: US [00027754, acc. # 1185617]).

##### Type.

Bolivia. La Paz: Larecaja, “viciniis Yani, in scopulosis”, Mar 1858, *G. Mandon 404* (lectotype, designated here: G [G00359948 = F neg. 23126, two sheets]; isotypes BM [BM000778198], BR [BR0000005537884], F [v0073313F, acc. # 680216], G [G00370041], GH [00077702], K [K000585550], NY [00172062, 00022559, 00172063], P [P00336757], S [acc. # 04-2925]).

**Figure 86.**
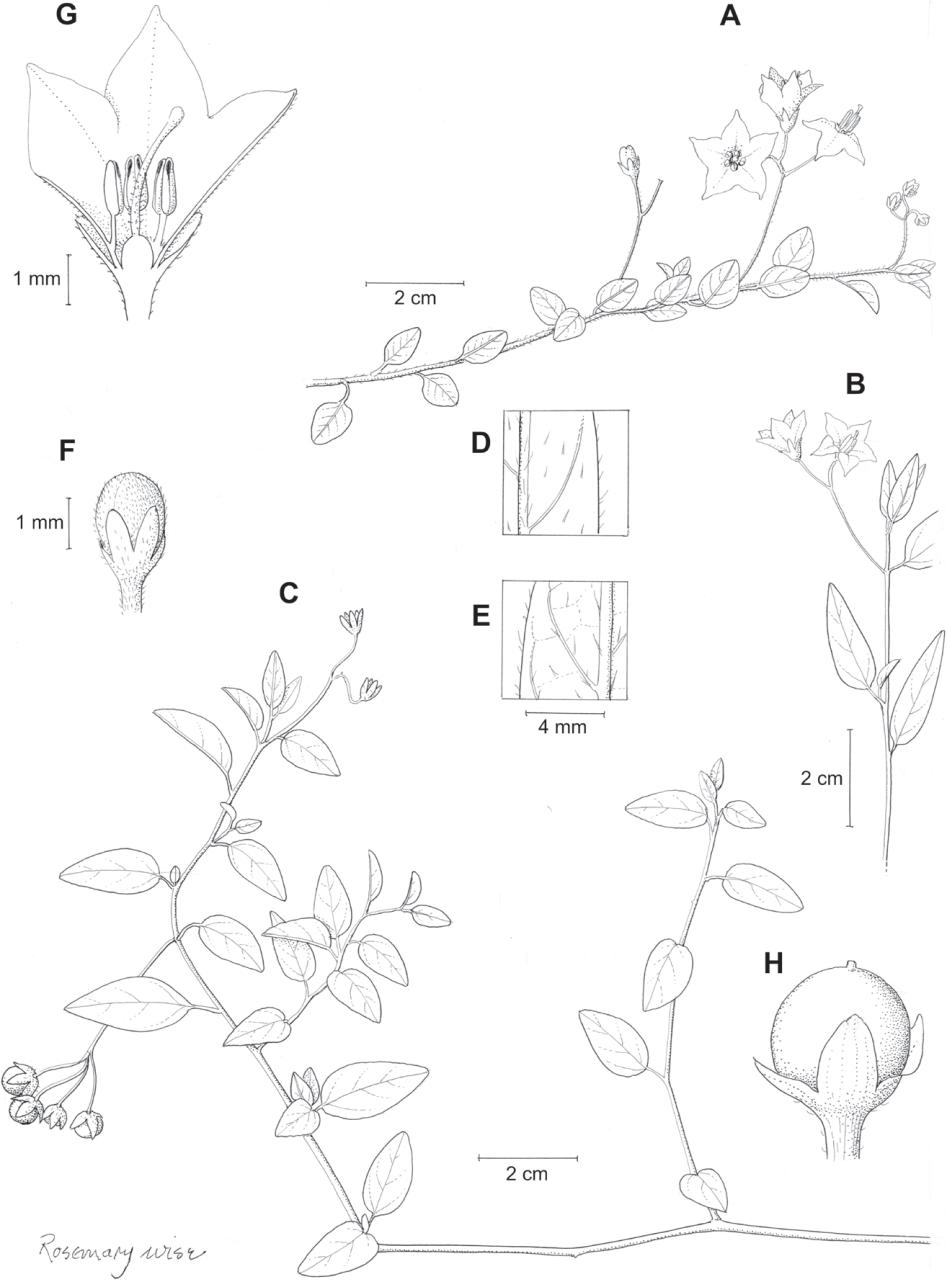
*Solanumleptocaulon***A** flowering habit **B** flowering habit with larger leaves **C** fruiting habit **D** detail of adaxial leaf surface **E** detail of abaxial leaf surface **F** floral bud **G** dissected flower **H** maturing fruit (**A, C–E***Brooke 6038***B***Ugent & Ugent 5066***F, G, H***Steinbach 648*). Illustration by R. Wise.

##### Description.

Herbs or creeping subshrubs, often sprawling and rooting at the nodes to 0.5(1) m high; stems terete, sparsely pubescent with white eglandular simple uniseriate trichomes 0.5–0.75 mm long, these stiff and antrorse, 3–4-celled, the cells elongate; new growth sparsely to moderately pubescent with antrorse white simple uniseriate trichomes like those of the stems, or occasionally almost glabrous; bark of older stems pale grey or brown, glabrescent. Sympodial units plurifoliate, the leaves not geminate, but sometimes paired at the nodes. Leaves simple, the blades 0.9–5 cm long, 0.5–2 cm wide, narrowly elliptic to elliptic, widest at the middle or occasionally with some leaves widest in the lower third and somewhat hastate, membranous or slightly thick and fleshy, concolorous; adaxial surfaces evenly and sparsely to moderately pubescent with white eglandular simple 2–4-celled uniseriate trichomes 0.5–0.8(2) mm long, these stiff and antrorse; abaxially similarly but more sparsely pubescent with stiff antrorse trichomes, these densest along the veins, but also some on the lamina; principal veins 3–5 pairs, barely visible above except for the prominent somewhat keeled midrib, often drying yellowish brown below; base acute to somewhat acuminate; margins entire and minutely revolute, sometimes with two teeth ca. 1 mm long near the base; apex acute or somewhat rounded; petioles 0.2–1 cm long, pubescent like the stems. Inflorescences opposite the leaves or terminal, unbranched or rarely forked (on the same plant, e.g., *Brooke 6038*), 1–3 cm long, with 1–4(10) flowers clustered at the tips, almost glabrous to sparsely pubescent with white eglandular simple uniseriate trichomes 0.3–0.5 mm long, these stiff and antrorse; peduncle 0.8–2.5 cm long; pedicels 0.8–1 cm long, 0.4–0.5 mm in diameter at the base, 1–1.2 mm in diameter at the apex, filiform and spreading, sparsely pubescent with simple trichomes like the rest of the inflorescence, articulated at the base; pedicel scars closely spaced and clustered at the tips of the inflorescence axis or branches. Buds ellipsoid, the corolla strongly exserted from the calyx before anthesis. Flowers 5-merous, cosexual (hermaphroditic). Calyx tube 1.5–2 mm long, narrowly cup-shaped, the lobes 1.5–2 mm long, 1–1.2 mm wide, triangular, glabrous or with tiny simple uniseriate trichomes ca. 0.2 mm long, the sinuses transparent, drying white and scarious. Corolla 2–2.4 cm in diameter, 1–1.2 cm long, pale violet, campanulate, lobed ca. 1/4 of the way to the base, the lobes 2–4 mm long, 5–6 mm wide, slightly incurved, adaxially glabrous, abaxially densely puberulent with tiny white uniseriate trichomes ca. 0.2 mm long where exposed in bud especially along petal midveins, appearing less pubescent with flower age due to expansion, the interpetalar tissue glabrous. Stamens equal, completely hidden within the corolla tube; filament tube minute; free portion of the filaments 1–1.5 mm long, with tangled transparent simple uniseriate trichomes adaxially; anthers 2.5–3 mm long, ca. 1 mm wide, ellipsoid, yellow, poricidal at the tips, the pores lengthening to slits with age. Ovary conical, glabrous; style 5–7 mm long, straight, exserted beyond the anther cone, densely pubescent in the lower half, entirely within the corolla tube; stigma globose-capitate to somewhat clavate, the surface minutely papillate. Mature fruits and seeds not known. Chromosome number: not known.

**Figure 87.**
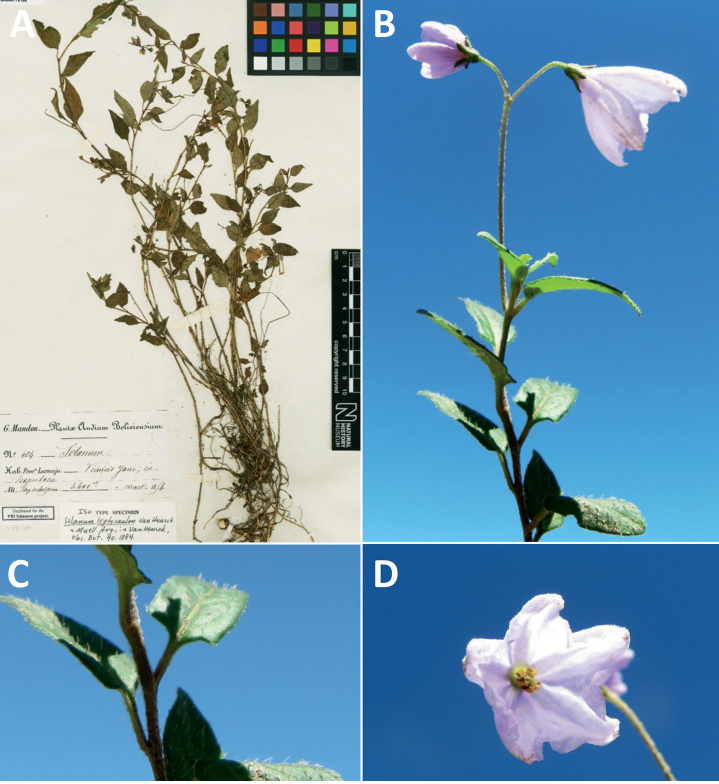
*Solanumleptocaulon***A** habit **B** flowering branch **C** leaves **D** flower at full anthesis (**A***Mandon 404* [BM000778198], reproduced with permission of the Trustees of the Natural History Museum **B–D***Nee et al. 55364*). Photos of live plants by S. Stern.

##### Distribution

**Figure 88.**
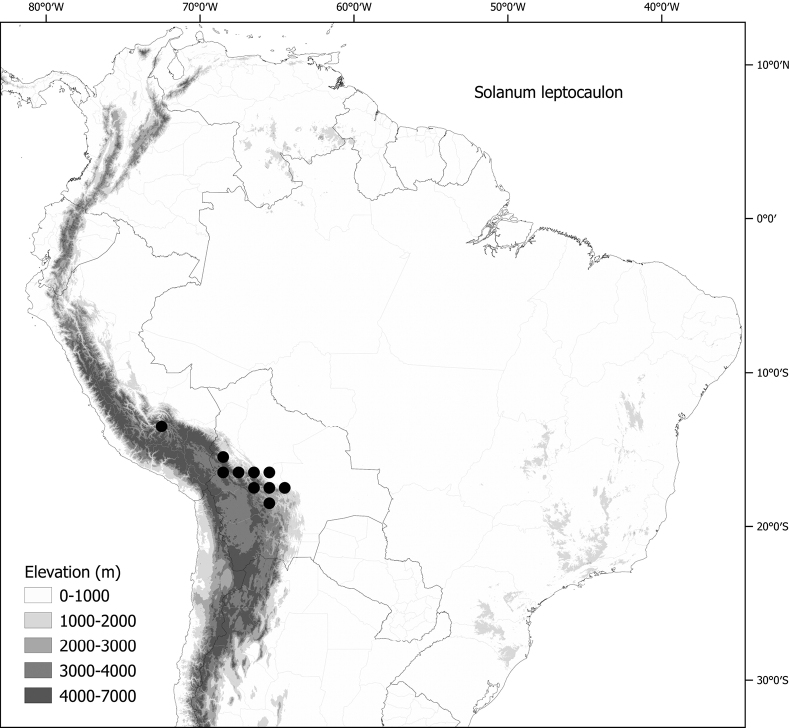
Distribution map of *Solanumleptocaulon*.

**(Fig. 88).***Solanumleptocaulon* is known from the Andes of Bolivia (Depts. Cochabamba, La Paz, Santa Cruz) and from a single collection in southern Peru (Dept. Cusco).

##### Ecology and habitat.

*Solanumleptocaulon* grows in high elevation grasslands or cloud forest margins, usually above timberline or in open areas of puna or pre-puna vegetation, from 1,870 to 3,950 m elevation.

##### Common names and uses.

None recorded.

##### Preliminary conservation status

**(IUCN 2022).** Least Concern [LC]. EOO = 66,386 km^2^ [LC]; AOO = 120 km^2^ [EN]. The relatively wide distribution of *S.leptocaulon* does not suggest it is of immediate conservation concern, but pressure from mining and grazing in the high elevation grassy areas where it occurs may impact the species in the future. It occurs near protected areas in Bolivia (e.g., Parque Nacional Amboró, Parque Nacional Tunari and Parque Nacional Carrasco) but we have seen no collections from within the parks.

##### Discussion.

*Solanumleptocaulon* is a semi-prostrate, straggly shrub of high elevations mostly from northern Bolivia; *Jardim 829* (Parque Nacional Tunari in Bolivia) is unusual in being recorded as a shrub 1 m high. It is similar to both *S.albescens* and *S.dianthum*, both also from the Bolivian Andes, and in the past specimens of these three taxa have been annotated as one or the other species somewhat chaotically. *Solanumleptocaulon* differs from *S.albescens* in its leaves with uniform short, stiff white pubescence (versus glabrous with longer curling trichomes confined to the stems and leaf margins), smaller corollas (1.1.2 cm long versus 1.5–1.8 cm long) and triangular calyx lobes (versus calyx lobes with somewhat fleshy expanded tips). Both species have campanulate flowers and anthers of more or less the same size, but the larger corollas of *S.albescens* make the anthers seem smaller.

*Solanumdianthum* is a shrub to 2 m high and often has geminate leaves, and like *S.leptocaulon* has even pubescence on stems and leaves. The most striking difference between *S.leptocaulon* and *S.dianthum* is the corolla shape; *S.leptocaulon* has campanulate corollas, while those of *S.dianthum* are stellate with distinct deltate lobes. The anthers of *S.leptocaulon* are shorter than those of *S.dianthum* (2.5–3 mm long versus 3.5–5 mm long). Fruits and seeds of *S.leptocaulon* are not known.

The protologue of *S.leptocaulon* (van Heurck and Müller Argoviensis 1870) cites a single collection, *Mandon 404* from two Herbaria “hb. Van Heurck et hb. DC.”. We have selected the duplicate from the De Candolle Herbarium (that not used for the *Prodromus* and bearing a label to that effect) as the lectotype (G00359948), as it is the best preserved of the cited specimens and is annotated by J. Müller (Müll.-Arg.). As is the case in the herbarium at Geneva, the specimen consists of two sheets, only the one without the barcode bears the original label (see Turland et al. 2018, Art. 8.3, Ex. 9).

Two collections were cited in the protologue of *S.rheithrocharis* (Bitter 1914b), *Kuntze s.n.* from “herb. Berol.” and *Herzog 806* from “herb. Turic.” [Z] (Bitter 1914b). The specimen of Kuntze’s collection in Berlin was destroyed (Zanoni 1980), the duplicate held in NY is not of good quality and is probably a specimen of *S.dianthum*. We thus select the other syntype, *Herzog 806* at Z (Z-000229529) as the lectotype; it bears an annotation label in Bitter’s hand.

Rusby (1927) stated in his introduction to descriptions of plants of the Mulford Expedition to Bolivia that “all type specimens are to be found in the herbarium of The New York Botanical Garden”, so even though a herbarium was not cited in the description of *S.pongoense* itself the specimen of *White 165* in NY (00172138) is the holotype.

#### ﻿29. Solanum longifilamentum

Taxon classificationPlantaeSolanalesSolanaceae

Särkinen & P.Gonzáles, PhytoKeys 44: 42. 2015.

Figs 89 , 90

##### Type.

Peru. Pasco: Prov. Oxapampa, Dist. Huancabamba, Parque Nacional Yanachaga-Chemillén, sector Tunqui, riberas del rio Muchuymayo, alrededores del hito PNYC, 1,790 m, 22 Oct 2008, *M. Cueva, A. Peña, R. Rivera & M. Moens 276* (holotype: USM [acc. # 00268971]; isotypes: HOXA, HUT, MO [MO-2507305, acc. # 6455431]).

**Figure 89.**
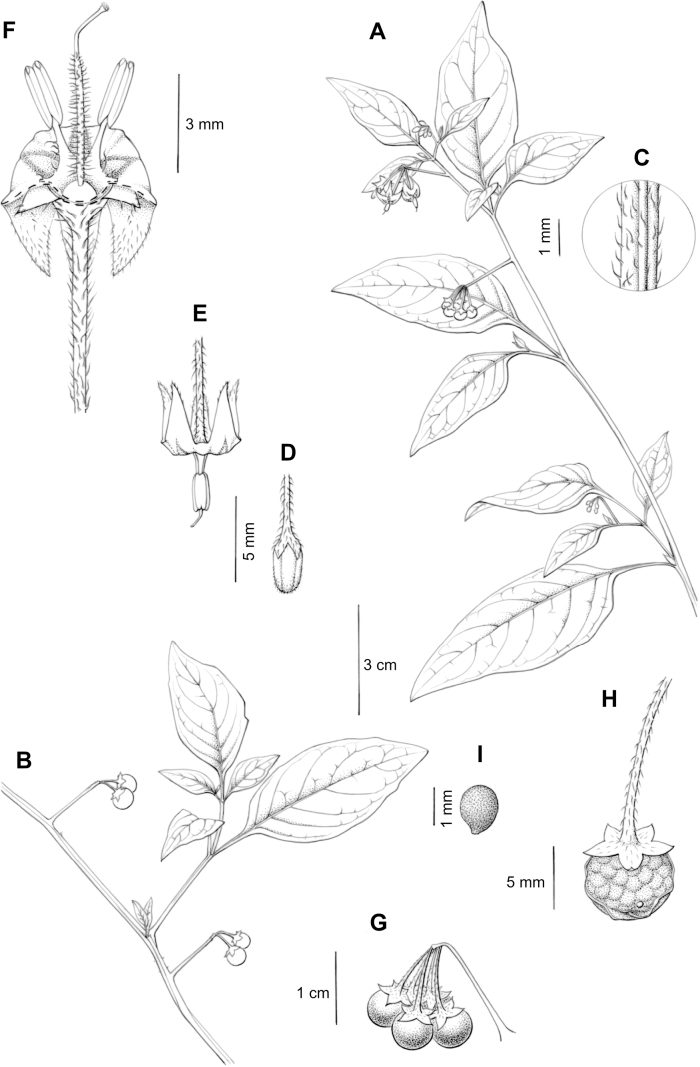
*Solanumlongifilamentum***A** flowering and fruiting branch **B** fruiting branch **C** stem detail with eglandular multi-cellular trichomes **D** flower bud **E** flower at full anthesis **F** dissected flower **G** infructescence **H** fruit **I** seed (**A–I***Särkinen et al. 4585*). Illustration by C. Banks.

##### Description.

Delicate herbs to small subwoody subshrubs, 0.2–1 m high, single stemmed or occasionally branching at the base. Stems terete to ridged, often tinged with purple, sparsely pubescent with appressed 1–2-celled simple uniseriate trichomes ca. 0.2 mm long. Sympodial units difoliate, not geminate. Leaves simple, the blades 2.5–12 cm long, 1–4 cm wide, ovate-lanceolate, membranous, somewhat discolorous; adaxial surface glabrous; abaxial surface with appressed 1–2-celled simple uniseriate trichomes like those of the stem along the veins; principal veins 4–8 pairs; base cuneate to attenuate, slightly unequal and oblique; margins entire; apex acuminate; petiole 0.5–1 cm long, sparsely pubescent with simple uniseriate trichomes like those of the stems and leaves, especially on young growth. Inflorescences internodal, unbranched, 1.5–3 cm long, with 3–5(6) flowers often all apparently arising from the same place, sparsely pubescent with simple uniseriate trichomes like those of the stems and leaves; peduncle 1–1.5 cm long, often tinged with purple; pedicels 0.5–0.6 cm long, ca. 0.4 mm in diameter at the base and 0.5 mm at apex, straight and spreading at anthesis, articulated at the base; pedicel scars closely spaced a maximum of 1 mm apart. Buds conical, white, occasionally purple-tinged towards the base, the corolla strongly exserted from the calyx tube long before anthesis. Flowers 5-merous, cosexual (hermaphroditic). Calyx tube ca. 1.5–2 mm long, the lobes 1–1.5 mm long, deltate to triangular with acute apices, slightly reflexed at anthesis, sparsely pubescent with simple uniseriate trichomes like those of the stems and leaves. Corolla 0.5–0.6 cm in diameter, stellate, white with a yellow, purple or black central star at the base, lobed 2/3 to nearly to the base, the lobes ca. 3–3.5 mm long, 1.5–2 mm wide, strongly reflexed at anthesis, later spreading, purple towards tips, densely pubescent abaxially with 1–2-celled simple uniseriate trichomes, these usually shorter than the trichomes of the stems and leaves. Stamens equal; filament tube minute, pubescent with a few scattered 3–5-celled trichomes at the base adaxially; free portion of the filaments ca. 1.1–1.4 mm long, pubescent like the tube; anthers (1.7-)3–3.4 mm long, 0.8–0.9 mm wide, ellipsoid, yellow, poricidal at the tips, the pores lengthening to slits with age. Ovary globose, glabrous; style 3.5–4 mm long, straight, short-exserted beyond the anther cone, densely pubescent in lower 1/4 with 2–3-celled simple uniseriate trichomes; stigma globose, minutely papillate, pale yellow in live plants. Fruit a globose berry, 0.6–0.7 cm in diameter, green at maturity or green and turning purplish black when ripe, the pericarp thin, shiny, somewhat translucent, glabrous; fruiting pedicels 1–1.2 cm long, ca. 0.6 mm in diameter at the base, 0.9 mm in diameter at the apex, spreading, not persistent; fruiting calyx lobes 1.8–3.5 mm long, spreading, the tips reflexed. Seeds 35–45 per berry, ca. 1.2 mm long, ca. 1.1 mm wide, tear-drop shaped, narrower at one end, brownish orange, the sub-lateral hilum positioned towards the narrower end of the seed, the testal cells pentagonal in outline. Stone cells 4–8 per berry, 0.4–0.5 mm in diameter, scattered throughout, white to cream-coloured. Chromosome number: not known.

**Figure 90.**
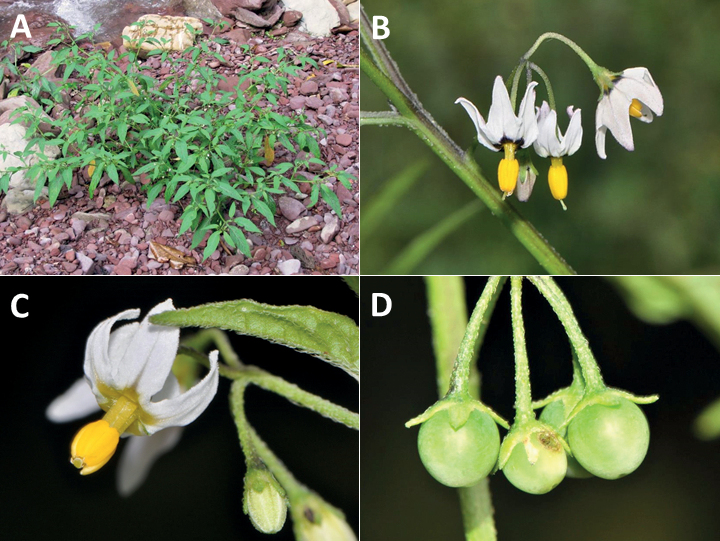
*Solanumlongifilamentum***A** habit **B** flowers at full anthesis **C** buds and flowers, floral type without a black central star **D** fruits with spreading calyx lobes (**A***Cueva et al. 276***B***Särkinen et al. 4030***C, D***Knapp et al. 10545*). Photos by S. Knapp, M. Cueva and T. Särkinen. Previously published in Särkinen et al. (2015c: 42).

##### Distribution

**Figure 91.**
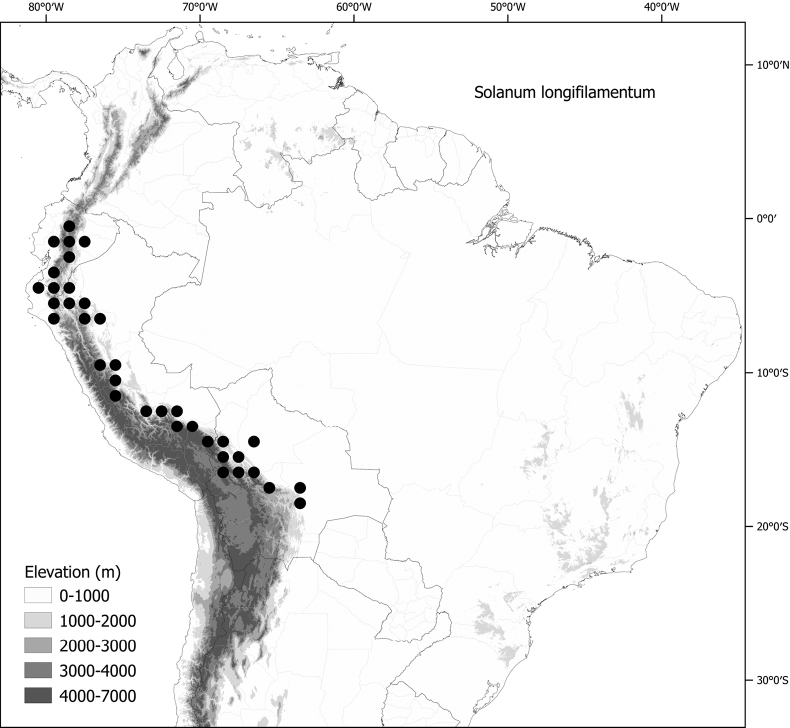
Distribution map of *Solanumlongifilamentum*.

**(Fig. 91).***Solanumlongifilamentum* is distributed from Ecuador (Provs. Azuay, Cañar, Chimborazo, Loja, Napo, Pastaza, Pichincha, Zamora-Chinchipe) to Peru (Depts. Amazonas, Ayacucho, Cajamarca, Cusco, Huánuco, Junín, Pasco, Puno, San Martín, Ucayali) and Bolivia (Depts. Beni, Cochabamba, La Paz, Santa Cruz) along the eastern slopes of the Andes.

##### Ecology and habitat.

*Solanumlongifilamentum* grows in mid-elevation montane forests in moist areas, along roadsides, often amongst mosses and small herbs; from (200-) 1,000 to 2,800 (-3,500) m elevation. In the Huancabamba depression in northern Peru (e.g., *Kujikat 104*), plants have often been collected at lower elevations.

##### Common names and uses.

Ecuador. Pastaza: wampishkúr (Shuar Jívaro, *Lewis 14172*). Peru. Cajamarca: mortiño (Spanish; *Särkinen et al. 4577*). Stems and leaves crushed and applied with achiote (*Bixaorellana* L., Bixaceae) warm to treat skin irritations (‘papera’) (*Lewis 14172*).

##### Preliminary conservation status

**(IUCN 2022).** Least Concern [LC]. EOO = 1,008,132 km^2^ [LC]; AOO = 468 km^2^ [EN]. *Solanumlongifilamentum*, since its recognition at the species level, has been shown to have a much wider distribution than originally thought by Särkinen et al. (2015c). Many recent collections exist, indicating that populations are not in decline and, as are most members of the Morelloid clade, *S.longifilamentum* is a weedy plant of disturbed areas.

##### Discussion.

*Solanumlongifilamentum* is most similar to *S.macrotonum* and *S.nigrescens* of northern South America. It can be distinguished from *S.macrotonum* by its longer calyx lobes (1–1.5 mm long versus 0.5–0.8(1) mm long) and filaments that are longer relative to the anthers (half the length of the anthers versus always much shorter than the anthers). The styles of *S.longifilamentum* are exserted to only 0.5–1 mm beyond the anther cone, but styles extend 1.5–3.5 mm beyond the anthers in *S.macrotonum*. *Solanumlongifilamentum* has consistently narrower, oblong-lanceolate leaves as compared to the more ovate leaves of *S.macrotonum*. *Solanumlongifilamentum* differs from *S.nigrescens* in its smaller flowers (0.5–0.6 cm in diameter versus 0.8–1 cm in diameter) with longer anthers (3–3.4 mm long versus 2–2.5 mm long), calyx lobes that are slightly reflexed at the tips at anthesis and strongly reflexed in fruit (versus tightly pressed to the berry in fruit) and its distribution in the Andes of Ecuador, Peru and Bolivia rather than Colombia and Venezuela (extending into Central America, Mexico, the Caribbean and the southern United States of America).

Other species with which *S.longifilamentum* could be confused include *S.americanum* and *S.pseudoamericanum* both of which have smaller anthers (1–1.5 mm long) and *S.interandinum* that is a larger, broadly spreading shrub up to 2 m high, with larger, violet corollas up to 2 cm in diameter and inflorescence axes that persist long after fruit drop.

#### ﻿30. Solanum macrotonum

Taxon classificationPlantaeSolanalesSolanaceae

Bitter, Repert. Spec. Nov. Regni Veg. 11: 222. 1912.

Figs 92 , 93

Solanum frutescens A.Braun & C.D.Bouché, Ind. Sem. Hort. Berol. App. 9. 1853, nom. utique rej. Type. Cultivated at Berlin Botanical Garden from seed sent from Caracas, Venezuela by J.W.K. Moritz, *Anon. s.n.* (possibly described from living material; if type material at B, destroyed; Knapp et al. 2018; Applequist 2022).
Solanum megalophyllum Bitter, Repert. Spec. Nov. Regni Veg. 11: 202. 1912. Type. Cultivated in England (?) ex Herb. A.B. Lambert “Villa Caracas cultum in hort. Boyton, Ph. Woodford”, *Anon. s.n.* (lectotype, designated by Knapp et al. 2019, pg. 75: W [acc. # 1889-0291427]; isolectotype: W [acc. # 1889-0291426 = F neg. 33091]).
Solanum diodontum Bitter, Repert. Spec. Nov. Regni Veg. 12: 552. 1913. Type. Panama. Chiriqui: around El Potrero Camp, 2,800–3,000 m, 10–13 Mar 1911, *H. Pittier 3104* (holotype: US [US00027551, acc. # 677494]; isotype: GH [GH00077485], NY [NY00138980], US [US00027550, acc. # 1405957]).
Solanum leonii Heiser, Ceiba 4: 298. 1955. Type. Costa Rica. Cartago: near Robert, Irazú [protologue -wooded ravine 1/2 mile below Finca Robert], 8,500 ft., 4 Oct 1953, *C.B. Heiser 3597* (holotype [two sheets]: IND [sheet 1, IND-0136009, acc. # 95138; sheet 2, IND-00136010, acc. # 95137]; isotype: F [V0073111F, acc. # 143245 = F neg. 49431]).
Solanum paredesii Heiser, Ci. & Naturaleza [Quito] 6: 55. 1963. Type. Ecuador. Pichincha: [Cantón Quito] laderas al norte de los terrenos de la Universidad Central, Ciudad Universitaria Quito, 24 May 1962, *C.B. Heiser 5001* (holotype: IND [IND-0136006, acc. # 106787]; isotype: Q [n.v.]).

##### Type.

Venezuela. Aragua: Colonia Tovar, Sep 1847, *J.W.K. Moritz 1643* (holotype: B, destroyed [F neg 2669]; lectotype, designated by D’Arcy 1974a, pg. 737: P [P00336967]; isolectotypes: BM [BM000617678], F [v0073325F, acc. # 612111], HBG [HBG511459], K [K000585559]).

**Figure 92.**
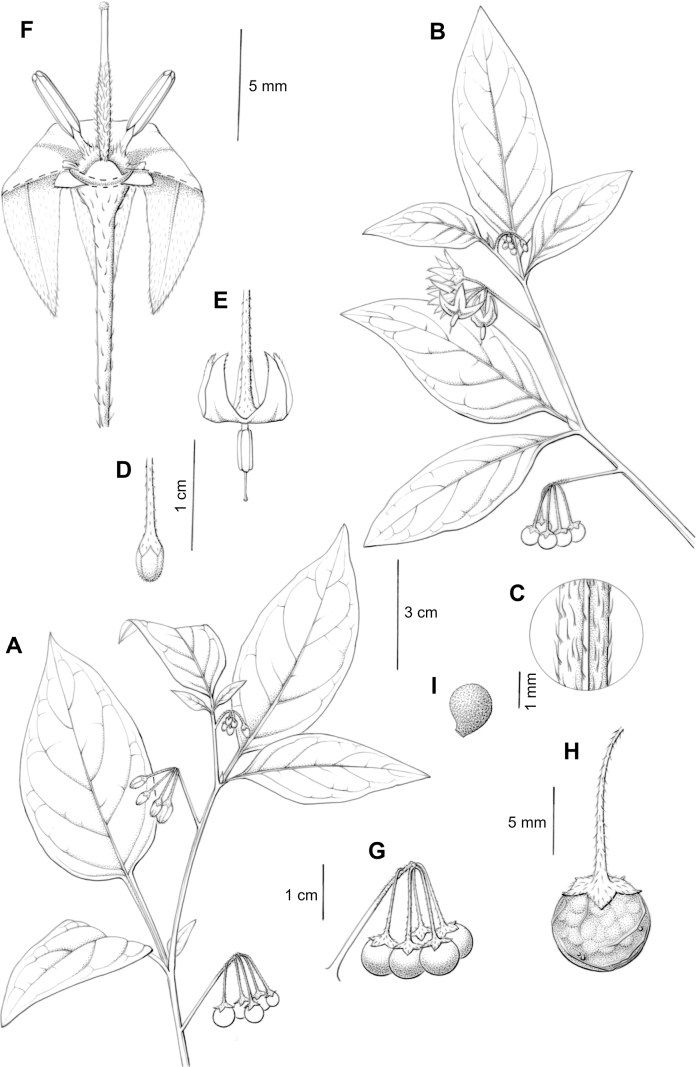
*Solanummacrotonum***A** fertile branch with flower buds and fruits **B** flowering and fruiting branch **C** stem detail with eglandular multi-cellular trichomes **D** flower bud **E** flower at full anthesis **F** dissected flower **G** infructescence **H** fruit **I** seed (**A–C, G–I***Ezedin & Särkinen 48*; **D–F***Balls 7528*). Illustration by C. Banks.

##### Description.

Perennial herbs to subwoody shrubs, 0.7–2 m high, perhaps occasionally annual or only persisting for a few years, often described as “viney”. Stems terete or angled with spinose processes, arching and scrambling over other vegetation, often drying blackish grey; young stems densely pubescent with somewhat antrorse, simple uniseriate eglandular trichomes 0.5–1 mm long, the trichomes drying white, soon glabrescent; new growth densely white pubescent like the young stems, glabrescent; bark of older stems green to greenish brown. Sympodial units difoliate or unifoliate, the leaves not geminate. Leaves simple, occasionally with a few dentate teeth near the base, the blades (2)4–10(12) cm long, (0.8)1.8–4.5(5.5) cm wide, elliptic to narrowly obovate, widest at the middle or in the upper half, sometimes thick (described as succulent), but more often membranous, concolorous; adaxial surfaces sparsely pubescent with simple 3–4-celled uniseriate trichomes or almost glabrous, the trichomes denser on veins and midrib; abaxial surfaces sparsely pubescent to glabrous like the adaxial surfaces, but the trichomes denser along the veins; principal veins 5–7 pairs, drying paler abaxially; base abruptly attenuate along the petiole; margins entire to sparsely toothed near the base; apex acute to narrowly acute; petiole 0.5–2.5 cm, sparsely pubescent with antrorse simple uniseriate trichomes like those of the stems and leaves. Inflorescences internodal or very occasionally opposite the leaves, unbranched or very occasionally forked (e.g., *Ruíz-Teran 14155*), 0.7–4 cm long, with 2–3(7) flowers clustered in the distal part of the axis (sub-umbelliform), sparsely pubescent with simple uniseriate trichomes like those of the stems and leaves; peduncle 0.5–4 cm long; pedicels 1–1.3 cm long, ca. 0.5 mm in diameter at the base, ca. 1 mm in diameter at the apex, tapering gradually and appearing relatively stout, often described as reddish purple or purple, spreading at anthesis, sparsely pubescent or glabrous, articulated at the base; pedicel scars tightly packed in the distal portion of the inflorescence, less than 0.5 mm apart or occasionally the lowermost scar to 2 mm apart. Buds broadly ellipsoid to subglobose, the corolla long-exserted from the calyx tube before anthesis. Flowers 5-merous, cosexual (hermaphroditic). Calyx tube 1–1.5 mm long, conical, the lobes 0.5–0.8(1) mm long, 0.5–1 mm wide, broadly deltate with acute apices, sparsely pubescent with simple uniseriate trichomes like those of the pedicel or almost glabrous. Corolla 1–2 cm in diameter, white to lilac or tinged with lilac, the central portion yellowish green, stellate, lobed halfway to 2/3 of the way to the base, the lobes 4–6 mm long, 1.5–3 mm wide, triangular, reflexed or spreading at anthesis, abaxially sparsely puberulent with tiny simple uniseriate trichomes. Stamens equal; filament tube minute and barely visible, the free portion of the filaments 1–2 mm long, pubescent with tangled simple uniseriate trichomes adaxially; anthers (2.7)3–4 mm long, 1–1.5 mm wide, ellipsoid, bright yellow, the surfaces smooth, poricidal at the tips, the pores elongating to slits with age. Ovary glabrous; style 5–6 mm long, straight, exserted beyond the anther cone, densely pubescent with tangled simple uniseriate trichomes in the basal half where included in the anther cone, markedly exserted from the anther cone; stigma capitate or minutely capitate, bright green, the surface densely papillate. Fruit a globose berry, 0.8–1 cm in diameter, green turning to black when ripe or occasionally green when ripe (*Nee & Whalen 16839*), the pericarp thin, more or less shiny but not brilliantly so, opaque, glabrous; fruiting pedicels 15–17 mm long, tapering from a base 0.7–1 mm in diameter to an apex 1.5–2 cm in diameter, somewhat woody, strongly deflexed (very occasionally appearing spreading due to herbarium specimen preparation), not persistent or occasionally remaining on the inflorescence axis; fruiting calyx not accrescent, the tube 1–1.5(2) cm long, appressed to the berry, the lobes 0.5–1 mm long, appressed or spreading at the tips. Seeds (10)30–50 per berry, 1.2–1.5 mm long, 0.8–1 mm wide, flattened and teardrop shaped, tan to reddish brown, the surfaces minutely pitted, the testal cells pentagonal, more elongate and rectangular near the hilum. Stone cells (2)4–5(6) per berry, 0.5–0.7 mm in diameter, white or cream-coloured. Chromosome number: 2n = 24 (Heiser 1955, as *S.leonii*); n = 36 (Heiser 1963, as. *S.paredesii*).

**Figure 93.**
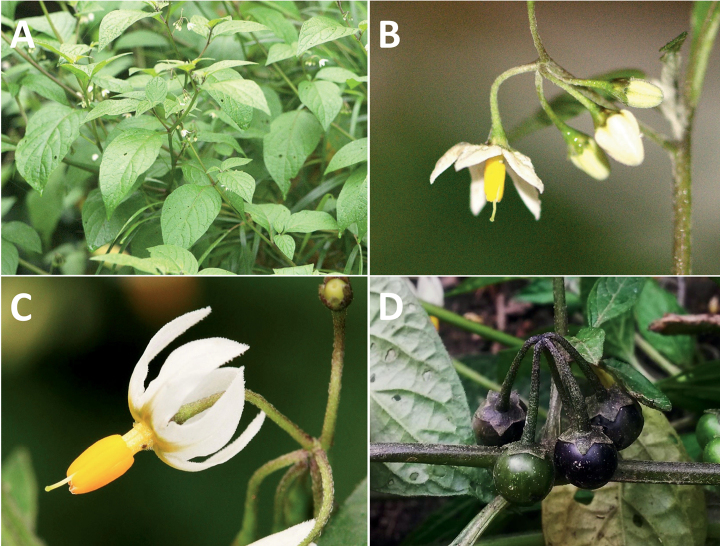
*Solanummacrotonum***A** habit **B** inflorescence **C** flower at full anthesis **D** maturing fruits (**A–D***Ezedin & Särkinen 48*). Photos by T. Särkinen. Previously published in Knapp et al. (2019: 77).

##### Distribution

**Figure 94.**
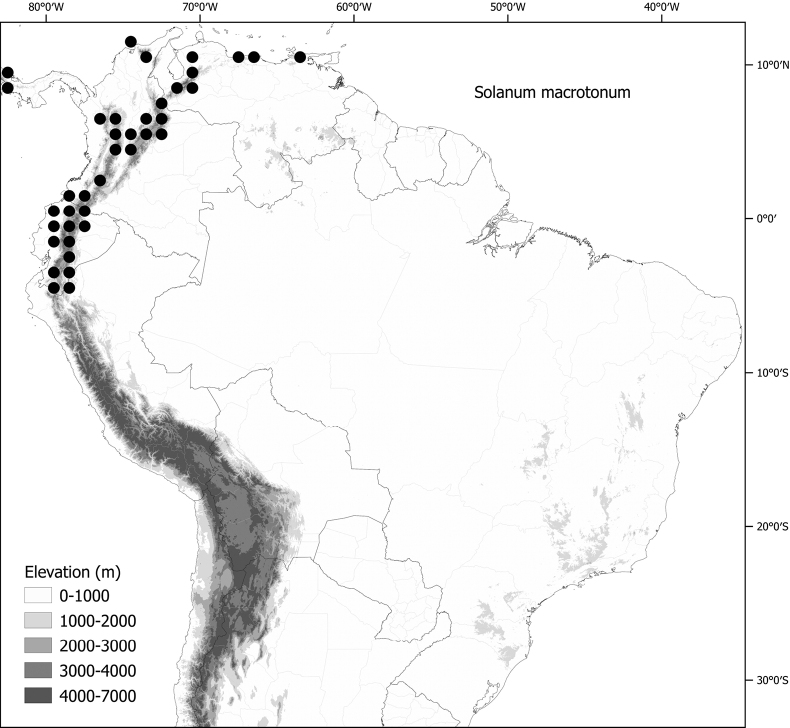
Distribution map of *Solanummacrotonum* in South America. For distribution in North and Central America and the Caribbean, see Knapp et al. (2019: 78).

**(Fig. 94).***Solanummacrotonum* is widely distributed from Guatemala to northern South America; Colombia (Depts. Antioquia, Boyacá, Cauca, Cundinamarca, Huila, Magdalena, Meta, Nariño, Norte de Santander, Putumayo, Quindío, Risaralda, Santander, Tolima, Valle de Cauca), Ecuador (Provs. Azuay, Bolívar, Carchi, Chimborazo, Cotopaxi, Imbabura, Loja, Morona-Santiago, Napo, Pichincha, Sucumbios, Tungurahua, Zamora-Chinchipe), Venezuela (States of Aragua, Lara, Mérida, Miranda, Sucre, Táchira, Trujillo, Vargas) and in the Antilles on the islands of Hispaniola and Jamaica.

##### Ecology and habitat.

*Solanummacrotonum* is a plant of open areas in cloud forests and premontane and montane forests, occurring in treefall gaps and along roads and other disturbances, from (200-)1,000 to 3,400 m elevation.

##### Common names and uses.

Colombia. Antioquia: hierba mora (*Kirkbride & Forero 1853*); Pasta: yerba mora (*Vogelmann 2006*); Cundinamarca: yerba mora (*Barragán-Fonseca 9*); Santander: yerba mora (*Combita et al. 114*). Ecuador. Chimborazo: hierba mora (*Cerón 15905 [a*]); Napo: hierba mora macho (*Baez et al. 32B*); Pichincha: papa de monte (*Mena V. 123*). Venezuela: Vargas: yerba mora (*González 17*). In Ecuador (Chimborazo, *Ceròn 15905 [a*]) an infusion of leaves is used as a bath for medicinal purposes.

##### Preliminary conservation status

**(IUCN 2022).** Least Concern [LC]. EOO = 4,218,133 km^2^ [LC]; AOO = 936 km^2^ [EN]; calculated on entire range in the Americas. *Solanummacrotonum* is widespread and is a weedy plant throughout its range. It occurs in several protected areas in Colombia (e.g., Selva de las Ventanas Natural Reserve), Ecuador (e.g., Bosque Protector de Pasochoa, Parque Nacional Llanganates) and Venezuela (e.g., Parque Nacional Chorro del Indio).

##### Discussion.

*Solanummacrotonum* is broadly sympatric with *S.nigrescens* across its entire range (see Knapp et al. 2019). It is similar to *S.nigrescens* in having usually 4 to 5 stone cells per berry and black fruits that are more or less shiny. It can be distinguished from *S.nigrescens* in having longer anthers (to 4 mm rather than to 2.5 mm) and in having more robust, longer fruiting pedicels that are strongly deflexed. Many annotations in herbaria have been done based on elevation (see comments in Bohs 2015) so care must be taken with determinations of these species. Measurement of anthers is the best way to determine specimens unambiguously. In general, *S.macrotonum* does occupy slightly higher elevations than does *S.nigrescens*, and appears to be confined to cloud forests, but *S.nigrescens* has a wide elevational range and ecological tolerance. The two species are sympatric throughout northern South America (Colombia and Venezuela).

*Solanummacrotonum* is also morphologically similar to *S.longifilamentum*, but as with *S.nigrescens*, differs from it in its longer anthers. *Solanummacrotonum* has larger corollas (1–2 cm in diameter versus 0.5–0.6 cm in diameter in *S.nigrescens*) and broadly deltate (rather than triangular) calyx lobes that do not split at the sinuses. The strongly deflexed fruiting pedicels of *S.macrotonum* are distinct from the spreading ones of *S.longifilamentum*.

*Solanummacrotonum* is one of few morelloids with differing chromosome counts across its range (but see also *S.interandinum*). D’Arcy (1974a) reported a chromosome number of “n = 36” for *S.macrotonum* as a personal communication from J.M. Edmonds; the chromosome count in Edmonds (1972) is not new and we presume it is a reference to the count (“número de cromosomas – 36”; Heiser 1963) given in the protologue of *S.paredesii*, which Edmonds (1972) placed in tentative synonymy with *S.macrotonum*. Some other chromosome vouchers of *S.macrotonum* at IND, however (e.g., *Heiser 4854*) are noted as having “n = 24” on the label; Heiser (1963) did not cite these in the description of *S.paredesii*. Chromosome counts for *S.leonii*, here treated in synonymy with *S.macrotonum*, indicate it is diploid, with 2n = 24 (Heiser 1955). Chromosome number variation within a species is known in *Solanum* (e.g., in the potatoes, see Spooner et al. 2014), and sometimes occurs sporadically at the edges of species ranges. It will be important to assess this across the range of *S.macrotonum*, because we cannot find any morphological characteristic that distinguishes vouchers with different chromosome counts.

Details of the typification of *S.macrotonum* and its synonyms can be found in Knapp et al. (2019). The earlier name *S.frutescens* A.Braun & C.D.Bouché was proposed (Knapp et al. 2018) and recommended for suppression (Applequist 2022).

#### ﻿31. Solanum marmoratum

Taxon classificationPlantaeSolanalesSolanaceae

Barboza & S.Knapp, PhytoKeys 164: 46. 2020.

Figs 95 , 96

##### Type.

Argentina. La Pampa: Dpto. Loventué, 10 km al W de Luan Toro, rumbo a Loventué, 297 m, 9 Feb 2020, *G.E. Barboza, S. Knapp, F. Chiarini & R. Fortunato 5099* (holotype: CORD [CORD00007007]; isotypes: BAB, BM [to be distributed]).

**Figure 95.**
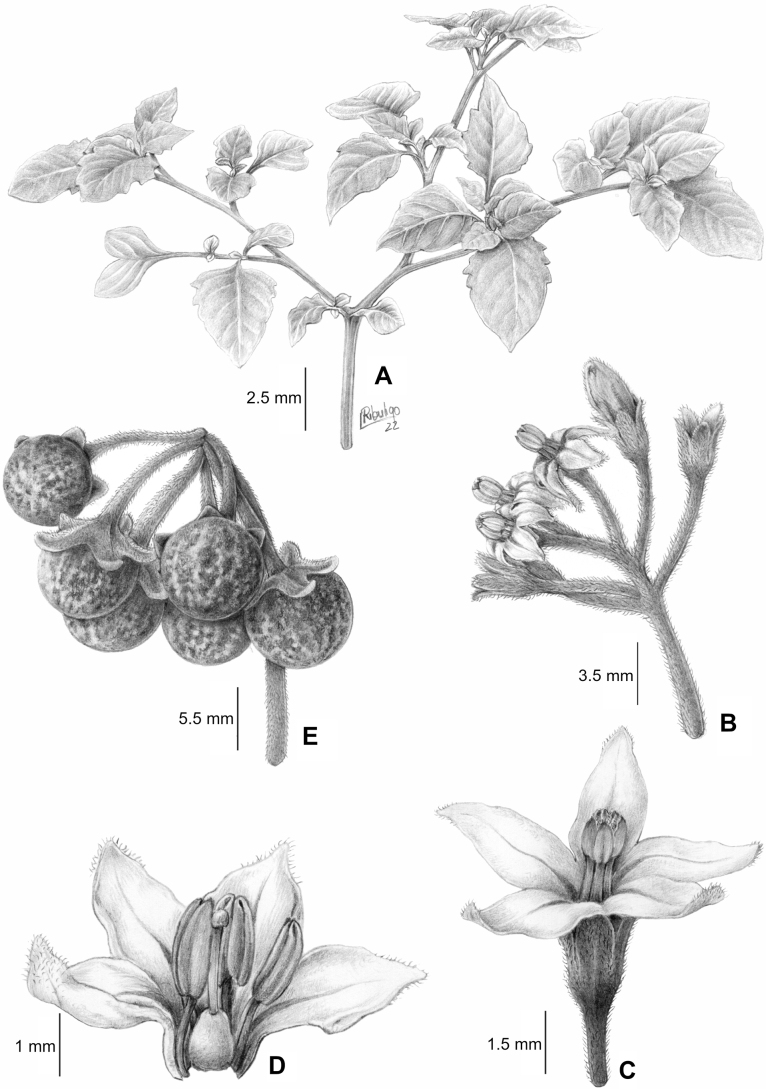
*Solanummarmoratum***A** habit, vegetative branch **B** inflorescence **C** flower at late anthesis **D** flower opened to show position of style at late anthesis **E** fruits (**A–E***Barboza et al. 5099*). Illustration by L. Ribulgo.

##### Description.

Watery annual herbs, 0.1–1 m high, sprawling and somewhat prostrate when large. Stems strongly winged, the wing to 1 mm wide, sometimes with spinose processes (old trichome bases), sparsely to moderately pubescent with spreading to appressed eglandular simple 5–8-celled uniseriate trichomes 0.5–1 mm long, these drying white; new growth densely pubescent with eglandular, white simple uniseriate trichomes 0.5–1 mm long; older stems greenish white, not woody. Sympodial units difoliate, the leaves not geminate. Leaves simple and shallowly toothed, the blades 2–10 cm long, 1.5–6 cm wide, much larger in older plants, ovate, widest in the lower third, membranous, watery and somewhat succulent, concolorous, very bright green on live plants; adaxial and abaxial surfaces evenly white-pubescent with eglandular simple 5–8-celled uniseriate trichomes 0.5–1 mm long, these longer and denser on the veins; principal veins 5–6 pairs; base attenuate onto the petiole; margins shallowly and irregularly toothed, the teeth 2–4 mm long, 2.4– mm wide, broadly deltate, with blunt tips; apex acute; petioles 0.5–2.5 cm long, somewhat winged from the attenuate leaf base, pubescent with simple uniseriate trichomes like the stems and leaves. Inflorescences internodal, unbranched, (1)2–3 cm long, with 5–7 flowers clustered at the tip, usually only 1–2 open at a time, sparsely and evenly pubescent with antrorse simple uniseriate trichomes 0.5–1 mm long like the stems and leaves; peduncle 1.4–2.5 cm long; pedicels 0.4 cm long, ca. 0.5 mm in diameter at the base, ca. 0.6 mm in diameter at the apex, slightly tapering, spreading, eglandular pubescent like the rest of the inflorescence, articulated at the base; pedicel scars tightly packed at the tip of the inflorescence, 0.5–1.5 mm apart. Buds broadly ellipsoid, the corolla included in the calyx tube until just before anthesis. Flowers 5-merous, cosexual (hermaphroditic). Calyx tube 1.2–1.5 mm long, cup-shaped, the lobes 1–1.5 mm, narrowly deltate-triangular, fleshy and recurved in live plants, sparsely pubescent with eglandular white trichomes on both surfaces like the rest of the plant. Corolla 0.5–0.8 cm in diameter, white with a green central star, stellate, lobed ca. halfway to the base, the lobes ca. 2.5 mm long, ca. 2 mm wide, spreading to slightly reflexed at anthesis (flowers closing daily and lasting for several days), adaxially glabrous, abaxially densely pubescent with tiny simple uniseriate trichomes especially at the tips. Stamens equal or slightly unequal with one anther marginally longer than the rest; filament tube ca. 0.1 mm long; free portion of the filaments 0.5–1 mm long, elongating through anthesis, with a few tangled transparent simple uniseriate trichomes adaxially; anthers 1–1.5 mm long 0.6–1 mm wide, ellipsoid, yellow, poricidal at the tips, the pores elongating with age. Ovary conical, glabrous; style 2–2.5 mm, straight, included within the anther cone or the stigma just visible, densely papillate in the lower 3/4; stigma large capitate, held at the level of the anthers when flowers first open, later included within the anther cone, bright green in live plants, the surfaces minutely papillate. Fruit a globose berry, 0.8–1.5 cm in diameter, dark green marbled with white at maturity, the pericarp surface thin, shiny, translucent, glabrous; fruiting pedicels 1.2–1.5 cm long, ca. 1 mm in diameter at the base, ca. 1.5 mm in diameter at the apex, fleshy and watery, tapering to the spreading calyx, strongly deflexed at maturity, with a distinct bend at the pedicel base, not persistent; fruiting calyx somewhat expanded, the tube 3–4 mm long, the lobes 4–5 mm long, ca. 3 mm wide, spreading and fleshy, the tips rounded. Seeds 50–70 per berry, ca. 2 mm long, ca. 1.7 mm wide, flattened teardrop shape with an apical hilum, pale tan to reddish brown, the surfaces minutely pitted, the testal cells mostly rectangular to pentagonal in outline, more sinuate towards the seed centre. Stone cells 1–2 per berry, 1–1.1 mm in diameter, randomly positioned in the berry. Chromosome number: not known.

**Figure 96.**
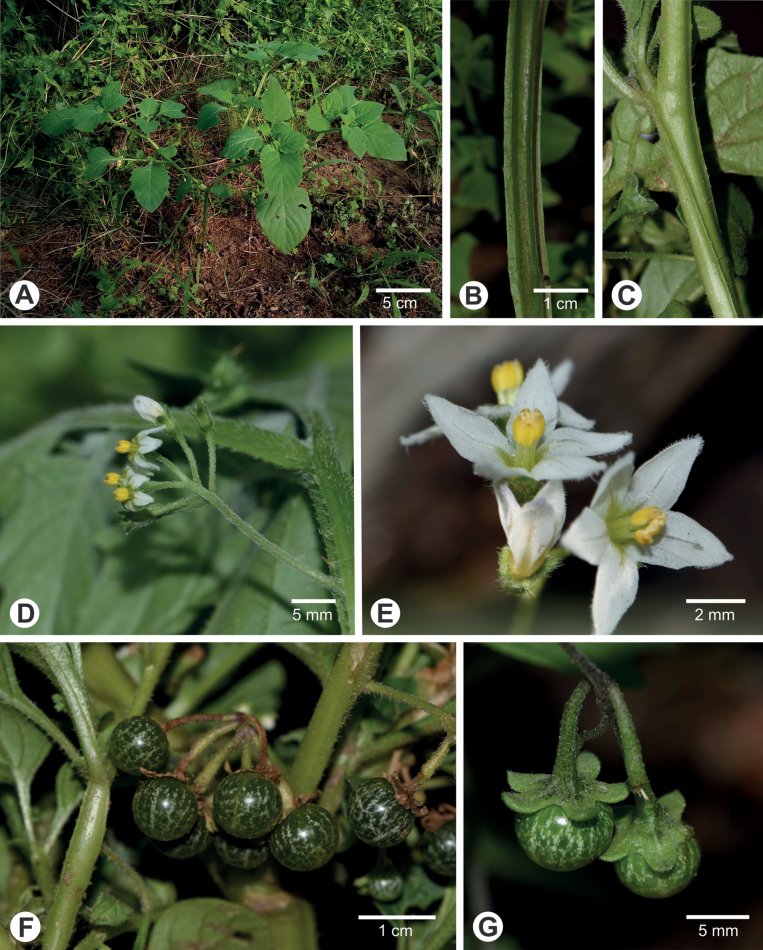
*Solanummarmoratum***A** habit **B, C** details of the winged stems **D** inflorescence **E** flowers, showing the included style and the filaments that elongate with flower age **F** mature fruits **G** detail of berries showing the spreading fleshy calyx in fruit (**A***Barboza et al. 5099***B, E***Barboza et al. 5136***C, F***Barboza et al. 5073*). Photos by S. Knapp. Previously published in Knapp et al. (2020: 47).

##### Distribution

**Figure 97.**
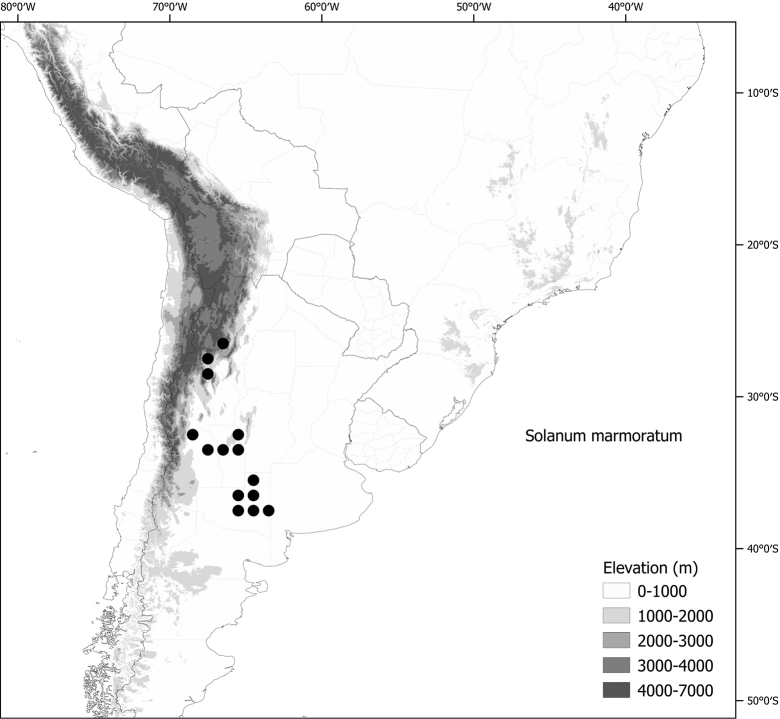
Distribution map of *Solanummarmoratum*.

**(Fig. 97).***Solanummarmoratum* is endemic to Argentina (Provs. Catamarca, La Pampa, La Rioja, San Luis); we expect it also to be found in Mendoza, because several collections are known from Desaguadero (San Luis), a locality very close to the provincial border that crosses through uniform habitat.

##### Ecology and habitat.

*Solanummarmoratum* is found in shady areas in *Prosopis* (Leguminosae) woodlands and at the edges of arable fields; it usually grows under trees and shrubs with a number of other herbaceous plants, from 200 to 1,400 m elevation.

##### Common names and uses.

None recorded.

##### Preliminary conservation status

**(IUCN 2022).** Least Concern [LC]. EOO = 266,502 km^2^ [LC]; AOO = 100 km^2^ [EN]. *Solanummarmoratum* is a relatively widespread species; the extent of occurrence suggests it should be given a status of least concern. The small area of occupancy perhaps reflects a lack of collecting in the dry forest and partially degraded habitats where *S.marmoratum* occurs. The number of localities (ca. 9) is probably an underestimate due to the widespread perception that these habitats are not interesting; most collections are quite old, and the species has not been collected recently (except by us). The large-scale conversion of land in the range of *S.marmoratum* to intensive monoculture of commercial crops such as maize, peanuts and sunflowers poses a risk for this and other species in these habitats; use of herbicides and elimination of patches of forest leave little room for even weedy species to persist. Widespread habitat conversion in central Argentina warrants further studies as to population status across the species’ historical range.

##### Discussion.

*Solanummarmoratum* had long confused botanists working with Argentinian solanums (see discussion in Knapp et al. 2020). We collected *S.marmoratum* in 2013 (*Barboza et al. 3668*) along with *S.tweedieanum*, and mistakenly noted the leaves of *S.marmoratum* as sticky; it was only examination of the dried specimens that alerted us to our error. Careful examination of all morelloid collections at CORD in early 2020 showed the distinctness of *S.marmoratum* and its relatively widespread but fragmented distribution.

The flowers of *S.marmoratum* are among the tiniest in the morelloid solanums rivalled only by the globally distributed *S.americanum* and *S.nitidibaccatum* and the North American *S.emulans* Raf. (see Knapp et al. 2019). *Solanumnitidibaccatum* also has somewhat marbled berries but is always extremely sticky and covered with glandular trichomes, in contrast to the eglandular pubescence of *S.marmoratum*. *Solanumamericanum* and *S.emulans* both have eglandular pubescence but have purplish black rather than green marbled berries. The fleshy spreading calyx lobes of *S.marmoratum* are distinct from those of all of these species with tiny flowers.

*Solanummarmoratum* appears to be highly autogamous and is perhaps entirely self-fertilising. Flowers stay open for several days (closing at night) and in cultivation the plant goes from bud to flower to fruit in 15–18 days with all flowers setting fruit (SK and GEB, pers. obs.). Over the course of anthesis the filaments elongate until the style becomes enclosed in the anther cone (Fig. 96E); as the anthers dehisce they leave pollen directly on the stigma. Ripe berries last more than two weeks after being gathered from desiccated plants, remaining unchanged as to colour or odour (G.E. Barboza, pers. obs.).

#### ﻿32. Solanum michaelis

Taxon classificationPlantaeSolanalesSolanaceae

Särkinen & S.Knapp, PhytoKeys 74: 22. 2016.

Figs 98 , 99

##### Type.

Bolivia. Tarija: Prov. Gran Chaco, 44.5 km (by rd) W from upper bridge over Rio Pilcomayo and 17.7 NE of Palos Blancos, on rd from Villa Montes to Palos Blancos, 815 m, 21 Mar 2007, *M. Nee & R. Flores S. 54821* (holotype: LPB; isotypes: BM [BM001211859], CORD [CORD00094450], MO [MO-2113149, acc. # 6073914], NY [00853628], SI [075094, acc. # 112169], US [02836465, acc. # 3595978], UT [acc. # 126715]; [records indicate that additional duplicates were sent to CAS, G, MEXU, NSW, USZ, WIS]).

**Figure 98.**
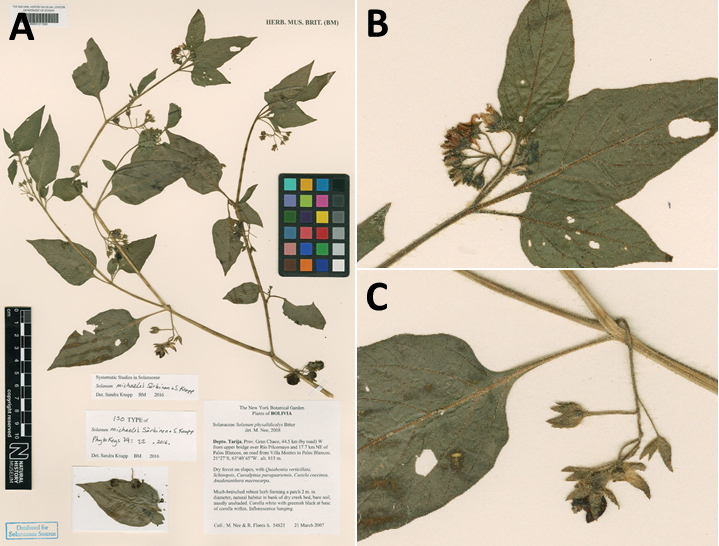
*Solanummichaelis***A** habit **B** inflorescence **C** maturing fruits (**A–C***Nee & Flores 54821* [BM001211859]). Reproduced with permission of the Trustees of the Natural History Museum.

##### Description.

Decumbent to erect subwoody herb to 1 m high, spreading to up to 2 m in diameter. Stems 3–4 mm in diameter at base, spreading or erect, terete, straw-coloured, glabrescent; new growth densely glandular-papillate and pubescent with a mixture of patent, simple, uniseriate eglandular and glandular trichomes, the trichomes of several lengths, 1-celled to 17-celled, 0.2–2 mm long, translucent, if glandular then with a terminal gland (this often breaking off). Sympodial units difoliate, not geminate. Leaves simple and shallowly lobed, the blades (2.4–)4–7.6 cm long, (1.4–)2.3–3(–4) cm wide, ovate, widest in the lower third, membranous, concolorous or slightly discolorous; adaxial surface moderately pubescent with both eglandular and glandular hairs along lamina and veins; abaxial surface more densely pubescent along veins; major veins 3–5 pairs; base truncate to rounded; margins entire to shallowly and unevenly lobed (mostly near the base); apex acute; petiole (0.7–)1.5–2 cm long, pubescent with spreading eglandular and glandular hairs like those on the stem. Inflorescences internodal or opposite the leaves, unbranched, 2.5–3.5 cm long, with (6–)7–10(–12) flowers, pubescent with both eglandular and glandular trichomes like those on stem; peduncle 1.4–3.3 cm long; pedicels spaced 0–1 mm apart, 6–10 mm long, ca. 0.2 mm in diameter at base and apex, straight and spreading at anthesis, articulated at the base. Buds ellipsoid, white or purple-tinged, densely pubescent with spreading, multicellular hairs (see description of calyx), the corolla not strongly exserted from the calyx, exceeding the calyx lobes by less than 1/2 of their lengths before anthesis. Flowers 5-merous, cosexual (hermaphroditic). Calyx tube 0.8–1.3 mm long, the lobes 1.4–3.7 mm long, 0.6–1 mm wide, triangular with long-acuminate apices, densely pubescent with both eglandular and glandular trichomes, the eglandular trichomes 1.5–3.5 mm long. Corolla 0.7–1.3 cm in diameter, white with a green-black basal central star, stellate, lobed halfway to the base, the lobes 2.5–3.2 mm long, 1.5–2.5 mm wide, reflexed at anthesis, later spreading, sparsely pubescent abaxially with multicellular simple spreading eglandular uniseriate trichomes to 0.5 mm long, densely papillate on the tips and margins. Stamens equal; filament tube 0.1–0.25 mm long; free portion of the filaments 0.2–0.3 mm long, adaxially pubescent with tangled eglandular simple uniseriate trichomes; anthers 2.5–3.2 mm long, 0.9–1.1 mm wide, ellipsoid, yellow, poricidal at the tips, the pores lengthening to slits with age. Ovary subglobose, glabrous; style 4–5 mm long, straight, exserted beyond the anther cone, densely pubescent with 4-celled simple uniseriate trichomes in the basal 1/2 or 3/5 where included in the anther cone; stigma capitate, the surface minutely papillate. Fruit a subglobose berry, slightly flattened, 0.5–1.2 cm in diameter, green and mottled with white vein-like reticulations (black when ripe fide *Fuentes & Navarro 2607*), the pericarp thin, shiny, translucent, glabrous; fruiting pedicels 1.6–2 cm long, ca. 0.5 mm in diameter at the base, ca. 1 mm in diameter at the apex, spaced 1–2 mm apart, strongly deflexed, not persistent, leaving raised pedicel scars to 0.1 mm high; fruiting calyx tube 2–2.5 mm long, the lobes 5–8 mm long and 3–3.5 mm wide, spreading to reflexed. Seeds 15–25 per berry, 1.7–2 mm long, 1.1–1.5 mm wide, teardrop shaped and somewhat flattened, pale brown, the surface minutely pitted, the hilum positioned subapically, the testal cells pentagonal in outline. Stone cells absent. Chromosome number: not known.

**Figure 99.**
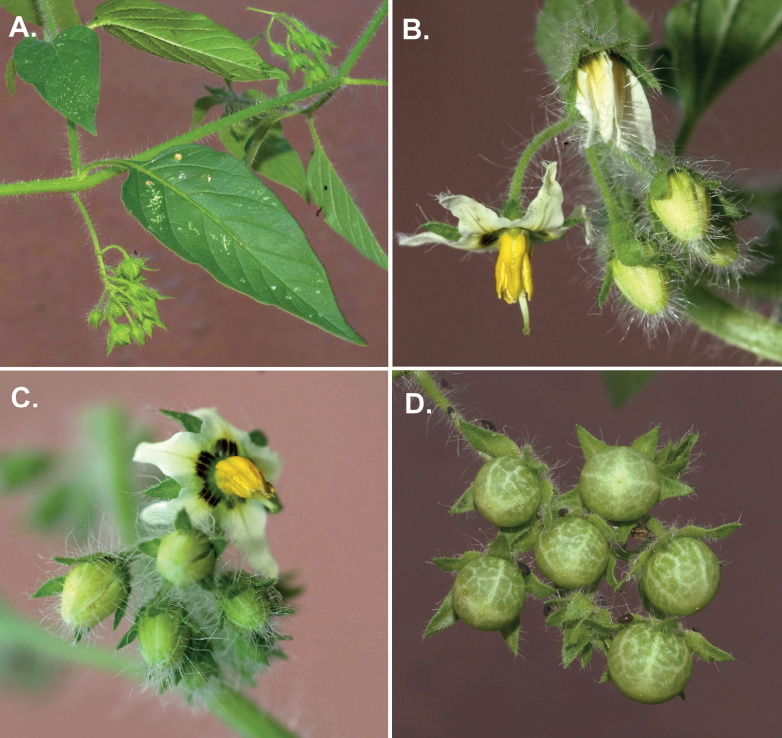
*Solanummichaelis***A** fruiting stem **B** inflorescence with details of mixed glandular and eglandular pubescence **C** flower at anthesis **D** maturing fruit (**A–D***Nee & Flores 54821*). Photos M. Nee. Previously published in Särkinen and Knapp (2016: 23).

##### Distribution

**Figure 100.**
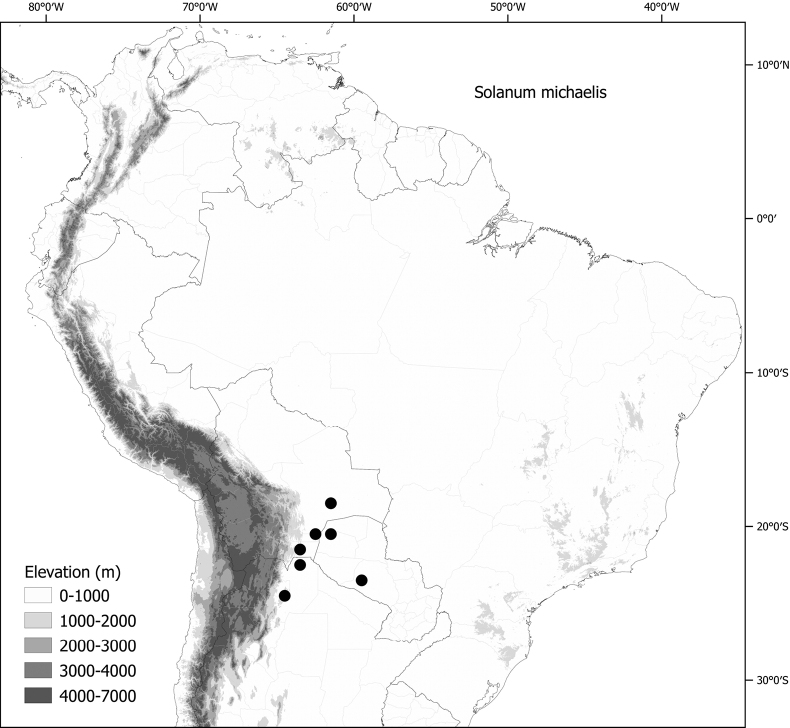
Distribution map of *Solanummichaelis*.

**(Fig. 100).***Solanummichaelis* occurs in Bolivia (Depts. Chuquisaca, Santa Cruz, Tarija), northern Argentina (Prov. Salta) and Paraguay (Dept. Presidente Hayes).

##### Ecology and habitat.

*Solanummichaelis* grows in dry Chaco vegetation and in lower inter-Andean valleys, along slopes in sandy soils in mostly unshaded dry creek beds on bare soil, often in areas that have been burned, or in more humid Chaco vegetation at the edge of “palmares” (stands of *Coperniciaalba* Morong, Arecaceae), between 300 and 900 m elevation.

##### Common names and uses.

None recorded.

##### Preliminary conservation status

**(IUCN 2022).** Least Concern [LC]. EOO = 1,008,132 km^2^ [LC]; AOO = 468 km^2^ [EN]. Särkinen and Knapp (2016) assigned a preliminary threat status of Endangered to *S.michaelis*. Although many more collections of this species have been found since its description, collection densities in the Chaco remain low, and *S.michaelis* is likely to be highly vulnerable to grazing pressure and changes in rainfall patterns due to its ephemeral ecology as an annual plant and its threatened habitat. The Chaco woodlands are highly threatened by land use change due to agricultural expansion and logging (Huang et al. 2009). Two populations are known to occur within the protected area network in Bolivia (e.g., Parque Nacional de Gran Chaco Kaa-lya along the border with Paraguay, and Parque Nacional de Serranía del Aguaragüe).

##### Discussion.

*Solanummichaelis* differs from the co-occurring and morphologically similar glandular-pubescent *S.sarrachoides* and *S.physalifolium* in having larger anthers (2.5–3.2 mm long); both *S.sarrachoides* and *S.physalifolium* have anthers less than 2.2 mm long. *Solanumphysalifolium* has similar shiny, green-mottled berries, but occurs at higher elevations (1,400 to 2,900 m) in ‘yungas’ or moist forest vegetation and has broadly ovate calyx lobes that partially enclose the fruit at maturity. *Solanumphysalidicalyx* has similarly sized anthers, but a longer calyx tube (ca. 1.5–2 mm in flower and to ca. 5 mm or more in fruit) which is inflated and fully encloses the berry both during development and at fruit maturity (see Knapp et al. 2020). *Solanummichaelis* has similarly long calyx lobes but a shorter calyx tube in both flower (0.8–1.3 mm) and fruit (2–2.5 mm) that does not enclose the fruit and appears to sometimes have reflexed calyx lobes at fruit maturity (e.g., *Fuentes & Navarro 2607*). The more widely distributed *S.tweedieanum* has much longer anthers (4–5 mm long), whitish green mature berries and the calyx tightly encloses the berry during development, but the berry can be somewhat exposed at maturity.

#### ﻿33. Solanum nigrescens

Taxon classificationPlantaeSolanalesSolanaceae

M.Martens & Galeotti, Bull. Acad. Roy. Sci. Bruxelles 12(1): 140. 1845.

Figs 101 , 102

Solanum nodiflorum Jacq. var. puberulum Dunal, Prodr. [A. P. de Candolle] 13(1): 46. 1852. Type. United States of America. Texas: [Bexar County] “Mexico, Bejar”, Oct 1828, *J.L. Berlandier 1904* (lectotype, designated by Edmonds 1972, pg. 103 [as holotype]: G-DC [G00144231]; isotypes: MO [acc. # 5481286], NY [00743232], P [P00319514], W [acc. # 0022313]).
Solanum caribaeum Dunal, Prodr. [A. P. de Candolle] 13(1): 48. 1852. Type. Jamaica. Sin.loc., [protologue – “In insulis Caribaeis, Jamaica, Guadalupâ”], no date, *Anon. s.n.* (lectotype, designated by D’Arcy 1974a, pg. 735: G-DC [G0000144199]).
Solanum crenatodentatum Dunal var. ramosissimum Dunal, Prodr. [A. P. de Candolle] 13(1): 54. 1852. Type. United States of America. Louisiana: “Basse Louisiane”, 1839, *G.D. Barbe 82* (holotype: P [P00362535]).
Solanum nigrum L. var. nigrescens (M.Martens & Galeotti) Kuntze, Revis. Gen. Pl. 2: 455. 1891. Type. Based on Solanumnigrescens M.Martens & Galeotti.
Solanum nigrum L. var. amethystinum Kuntze, Revis. Gen. Pl. 2: 455. 1891. Type. Costa Rica. San José/Cartago: “Irazu”, 24 Jun 1874, *O.E. Kuntze s.n.* (neotype, designated here: NY [00688134]).
Solanum prionopterum Bitter, Repert. Spec. Nov. Regni Veg. 11: 5. 1912. Type. Venezuela. Distrito Federal: “Caracas, in arena ad rivulum in valle loci dicti Valle”, 25 Mar 1854, *J. Gollmer s.n.* (holotype: B, destroyed [F neg. 2699], possibly the same original material as the type of S.gollmeri; no duplicates found).
Solanum gollmeri Bitter, Repert. Spec. Nov. Regni Veg. 11: 202. 1912. Type. Cultivated in Berlin (“horto bot. Berol.”) from seeds sent from Caracas, Venezuela by J. Gollmer, 1859, *Without collector s.n.* (holotype: B, destroyed [F neg. 2689]; lectotype, designated by Knapp et al. 2019, pg. 80: F [V0361922F, acc. # 621268], mounted on sheet with F neg. 2689).
Solanum pruinosum Dunal var. phyllolophum Bitter, Repert. Spec. Nov. Regni Veg. 12: 77. 1913. Type. Cultivated in Europe, seeds from Mexico from David Fairchild as USDA-32065 [protologue “sub. no. 32065, Mexico, S.nigrum”] (no specimens cited, probably described from living plants; original material at B?).
Solanum subelineatum Bitter, Repert. Spec. Nov. Regni Veg. 12: 79. 1913. Type. Cultivated at Bremen from seeds from Mexico sent by U. S. Dept. Agriculture, Bureau of Plant Industry, no. 32067 (original material in Bremen? [destroyed]; possibly described from living material).
Solanum oligospermum Bitter, Repert. Spec. Nov. Regni Veg. 12: 80. 1913. Type. Mexico. Oaxaca: Sierra de San Felipe, 7,500 ft., Oct 1894, *C.G. Pringle 4948* (lectotype, designated by Edmonds 1972, pg. 108 [as holotype]: Z [Z000033841]; isolectotypes: BM [BM001017184], BR [BR0000005537983], E [E00570141], GOET [GOET003559], HBG [HBG511469], KFTA [KFTA0000498], NDG [NDG45082], NY [NY00139012], PH [00030459], S [acc. # S-G5704], US [US00027711, acc. # 251984; US01014256, acc. # 1418095], W [acc. # 1895-0004424]).
Solanum durangoense Bitter, Repert. Spec. Nov. Regni Veg. 12: 82. 1913. Type. Mexico. Durango: “prope urbem Durango”, Apr 1896, *E. Palmer 101* (holotype: B, destroyed; lectotype, designated by D’Arcy 1974a, pg. 738: US [US00027556, acc. # 304231]; isolectotypes: BM [BM001034665], F [V0073093F, acc. # 51213, F. neg. 052464], K [K000063870], MO [MO-568723, acc. # 1718478], NY [00138982]).
Solanum purpuratum Bitter, Repert. Spec. Nov. Regni Veg. 13: 85. 1913. Type. Bahamas. Andros Island: Coppice, near Fresh Creek, Northern Section, 28–13 Jan 1910, *J.K. Small & J.J. Carter 8805* (holotype: P [P00369223]; isotypes: F [acc. # 283797], K [K001161011], NY [00111385], US [00027765, acc. # 758168]).
Solanum approximatum Bitter, Repert. Spec. Nov. Regni Veg. 13: 86. 1913. Type. Jamaica. Saint Andrew: Hardwar Gap, 4,000 ft., 17 Jun 1903, *G.E. Nichols 89* (holotype: B, destroyed; lectotype, designated by Knapp et al. 2019, pg. 80: NY [NY00111374]; isolectotypes: F [F0073167F, acc. # 147000], GH [GH00077545], MO [MO-503650, acc. # 1815480], US [US00027456, acc. # 429037], YU [YU065289]).
Solanum amethystinum (Kuntze) Heiser, Ceiba 4: 296. 1955. Type. Based on SolanumnigrumL.var.amethystinum Kuntze.
Solanum costaricense Heiser, Ceiba 4: 297. 1955. Type. Costa Rica. Heredia: La Paz, by waterfall, on road to Vara Blanca, about 29 mi. from Heredia, 1,400 m, 13 Sep 1953, *C.B. Heiser 3536* (holotype [two sheet holotype]: IND [IND1000067, acc. # 95105; IND1000068, acc. # 95106]; isotypes: CORD [CORD00004189], US [04064608, acc. # 2485189]).

##### Type.

Mexico. Oaxaca: “Cordillera” [“aux bords des ruiseaux de la cordillera de Yavezia”], Nov-Apr 1848, *H. Galeotti 1238* (lectotype, designated by D’Arcy 1974a, pg. 737: P [P00337261]; isolectotypes: BR [BR000000825045, BR0000008250483], W [acc. # 0022312, acc. # 1889-0291397]).

**Figure 101.**
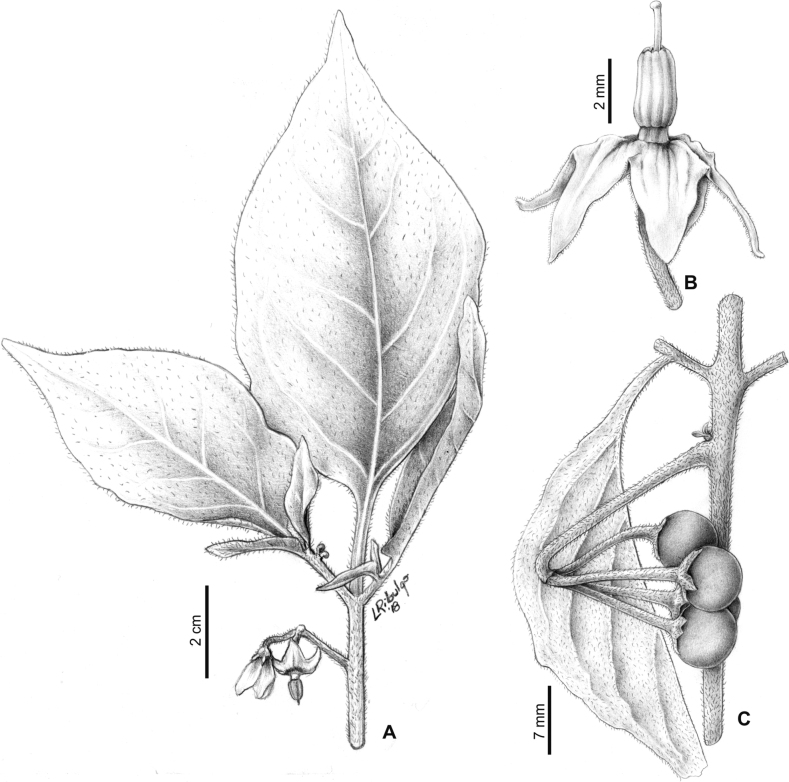
*Solanumnigrescens***A** new shoot **B** flower **C** inflorescence with mature fruit (**A–C***Ventura 672*). Illustration by L. Ribulgo. Previously published in Knapp et al. (2019: 81).

##### Description.

Perennial herbs to 3 m high, sometimes epiphytic. Stems terete or more usually angled to ridged, green or sometimes tinged purplish green, usually lax and somewhat scrambling, glabrescent to sparsely pubescent with antrorse simple eglandular uniseriate trichomes to 1 mm long, these white when dry and usually somewhat curved, occasionally on older stems the trichome bases enlarged and forming spinescent processes; new growth more densely pubescent. Sympodial units difoliate, geminate or not, the leaves if paired of similar size and shape. Leaves simple, often shallowly lobed, the blades (1.5)4–10.5(15) cm long, (0.5)2–5(7.5) cm wide, elliptic to elliptic ovate, widest at the middle, membranous, concolorous or somewhat discolorous; surfaces sparsely to moderately pubescent with simple eglandular uniseriate trichomes to 1 mm long, these denser on the veins and abaxially; principal veins 5–6 pairs; base abruptly attenuate, usually decurrent on the petiole; margins entire to sinuate or dentate, the teeth irregular and unevenly spaced, often larger in the basal half of the lamina; apex acute or occasionally acuminate; petiole 0.5–2 cm long, sparsely pubescent like the stems and leaves. Inflorescence internodal, unbranched to occasionally forked, 1–3.5 cm long, with (2)5–10 flowers clustered at the tip (sub-umbelliform) or spaced along the axis (depending on inflorescence age), sparsely pubescent with antrorse simple eglandular trichomes like the stems; flowering-bearing portion 0.3–1 cm long; peduncle 1–2.5 cm long, slender, spreading; pedicels 0.4–0.7 cm long, slender and threadlike, spreading at anthesis, ca. 1 mm in diameter at the base, ca. 0.5 mm in diameter at the apex, sparsely pubescent like the inflorescence axis, articulated at the base. Buds ellipsoid with blunt tips, the corolla strongly exserted from the calyx tube long before anthesis. Flowers 5-merous, cosexual (hermaphroditic). Calyx tube 1–1.2 mm, conical, the lobes 0.5–0.8(1) mm long, 0.5–1 mm wide, broadly deltate to deltate, the apices acute or occasionally somewhat rounded. Corolla 0.8–1 cm in diameter, white or less often pale purple, with a green or yellow-green (very occasionally dark purple) central portion near the base of the lobes, stellate, lobed ca. 3/4 of the way to the base, the lobes 3–4 mm long, 1.5–2 mm wide, narrowly triangular, reflexed or spreading, densely papillate abaxially, the papillae ca. 0.1 mm long, denser at the tips and margins. Stamens equal; filament tube minute; free portion of the filaments 0.5–2 mm long, densely pubescent adaxially with tangled simple trichomes; anthers 2–2.8(3) mm long, 1–1.1 mm wide, yellow, ellipsoid or narrowly ellipsoid, poricidal at the tips, the pores lengthening to slits with age. Ovary globose, glabrous; style 3.5–5 mm long, usually somewhat curved, often exserted from the bud before anthesis, exserted beyond the anther cone at anthesis, densely pubescent in the basal 2/3 (the portion inside the anther cone), exserted from the anther cone; stigma minutely capitate, the surface papillose. Fruit a globose berry, 0.6–0.8 cm in diameter, dull green to purplish black at maturity, the pericarp thin and usually matte but sometimes slightly shiny, opaque, glabrous; fruiting pedicels 1–1.2 cm long, ca. 0.5 mm in diameter at the base, ca. 1 mm in diameter at the apex, not markedly woody, spreading, not persistent or occasionally remaining on the inflorescence axis; fruiting calyx not accrescent, the tube ca. 1 mm long, the lobes 0.5–1.1 mm long, spreading and appressed to the berry, very occasionally somewhat reflexed. Seeds (5)10–50 per berry, 1.2–1.5 mm long, 1–1.1 mm wide, flattened and teardrop shaped, pale brown to yellow, the surfaces minutely pitted, the testal cells square or pentagonal in shape, becoming elongate and rectangular near the subapical hilum. Stone cells 4–13, mostly commonly 5 or 6, rather large to ca. 0.5 mm in diameter. Chromosome number: n = 12 (Heiser 1955, voucher *Heiser 3536* as *S.costaricense*; Heiser et al. 1965, voucher *Heiser S106* as *S.amethystinum*)..

**Figure 102.**
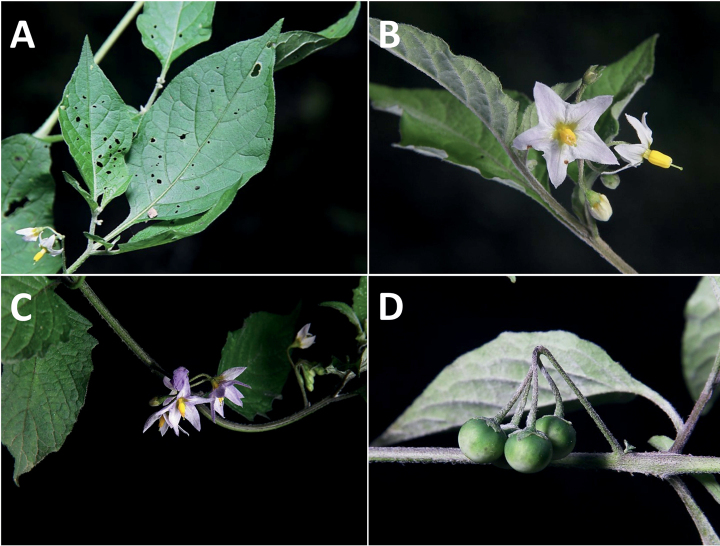
*Solanumnigrescens***A** leaves **B** flowering branch **C** inflorescence with flowers at full anthesis **D** developing fruits (**A–D***Amith et al. F0055*). Photos by M. Gorostiza Salazar. Previously published in Knapp et al. (2019: 82).

##### Distribution

**Figure 103.**
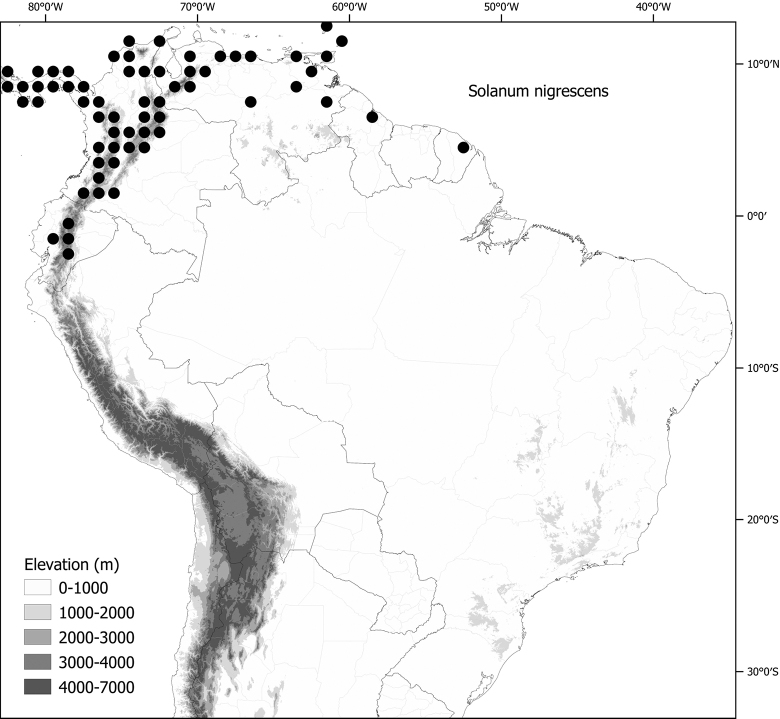
Distribution map of *Solanumnigrescens* in South America. For distribution in North and Central America and the Caribbean, see Knapp et al. (2019: 84).

**(Fig. 103).***Solanumnigrescens* is a widespread species ranging from the southeastern United States of America through Central America, northern South America, and the Caribbean; in South America it occurs in Colombia (Depts. Atlántico, Antioquia, Bolívar, Boyacá, Caldas, Caquetá, Cauca, Cesar, Chocó, Cundinamarca, Huila, La Guajira, Magdalena, Meta, Nariño, Quindio, Risaralda, Santander, Tolima, Valle de Cauca), Ecuador (Provs. Chimborazo, Pichincha, Tungurahua, Zamora-Chinchipe), Venezuela (States of Apure, Aragua, Bolívar, Capital, Carabobo, Delta Amacuro, Lara, Mérida, Miranda, Portuguesa, Sucre, Táchira, Trujillo, Vargas, Yaracuy, Zulia) and the Guianas (Guyana, Suriname, French Guiana).

##### Ecology and habitat.

*Solanumnigrescens* is most commonly collected from open areas in cloud forests, deciduous forests and pine forests between sea level and 3,000 m elevation in South America, but most common at lower elevations (ca. 1,500 m).

##### Common names and uses.

Colombia. Caldas: yerba mora (*Grisales 9*); Santander: yerba mora (*López & Gonzáles 31*). Ecuador. Azuay: mortiño negro (*Cerón 15905*); Cañar: mortiño blanco (*Kohn 1469*); Chimborazo: hierba mora (*Cerón 15905 [b*]). Leaves widely used a potherb (“quelite”) in Mexico and Central America, but we have not seen this use recorded on South American specimens.

##### Preliminary conservation status

**(IUCN 2022).** Least Concern [LC]. EOO = 21,536,739 km^2^ [LC]; AOO = 4,260 km^2^ [EN]; calculated on entire species range. *Solanumnigrescens* is widespread and weedy in the southern United States, throughout Mexico and Central America and in the Caribbean; it also occurs in northern South America. It has been registered as a noxious weed of agriculture in Louisiana (Orgeron et al. 2018).

##### Discussion.

*Solanumnigrescens* is one of the commonest and most widely distributed of all morelloid species in northern South America, Central America and the Caribbean. It is very variable morphologically, perhaps due to its wide ecological tolerance and occurrence in many different habitats. It is sympatric or occurs parapatrically with *S.americanum* and may hybridise with it in the southeastern United States (see Knapp et al. 2019). Putative hybrids have not been seen from South America.

Where *S.nigrescens* and *S.americanum* occur in sympatry, the matte berries with appressed to spreading calyx lobes of *S.nigrescens* are distinct from the shiny berries with strongly reflexed tiny calyx lobes of *S.americanum*; anther length also differs (0.7–1.5 mm in *S.americanum* versus 2–2.8(3) mm in *S.nigrescens*). *Solanumnigrescens* is also similar and sympatric with *S.macrotonum*. It differs from that species in its shorter anthers (1–8–2.5 mm long versus (2.7)3–4 mm long) and spreading (versus strongly deflexed) pedicels in fruit. Like most morelloid species, *S.nigrescens* is very weedy and occupies a wide range of disturbed and undisturbed habitats. *Solanumnigrescens* is a perennial and has been reported to be epiphytic in some situations (D’Arcy 1974a, b).

Material identified as *S.americanum* by Manoko et al. (2007) represents specimens of *S.nigrescens* (Särkinen et al. 2018: 61).

Bitter (1914a) reported large numbers of stone cells in the berries of many of the names we consider synonyms of *S.nigrescens*. In general, *S.nigrescens* has more stone cells in its berries than do other similar taxa, but these can be difficult to see as some of them are very tiny.

Details of typification of *S.nigrescens* and its many synonyms can be found in Knapp et al. (2019).

#### ﻿34. Solanum nitidibaccatum

Taxon classificationPlantaeSolanalesSolanaceae

Bitter, Repert. Spec. Nov. Regni Veg. 11: 208. 1912, nom cons. prop.

**Figure 104.**
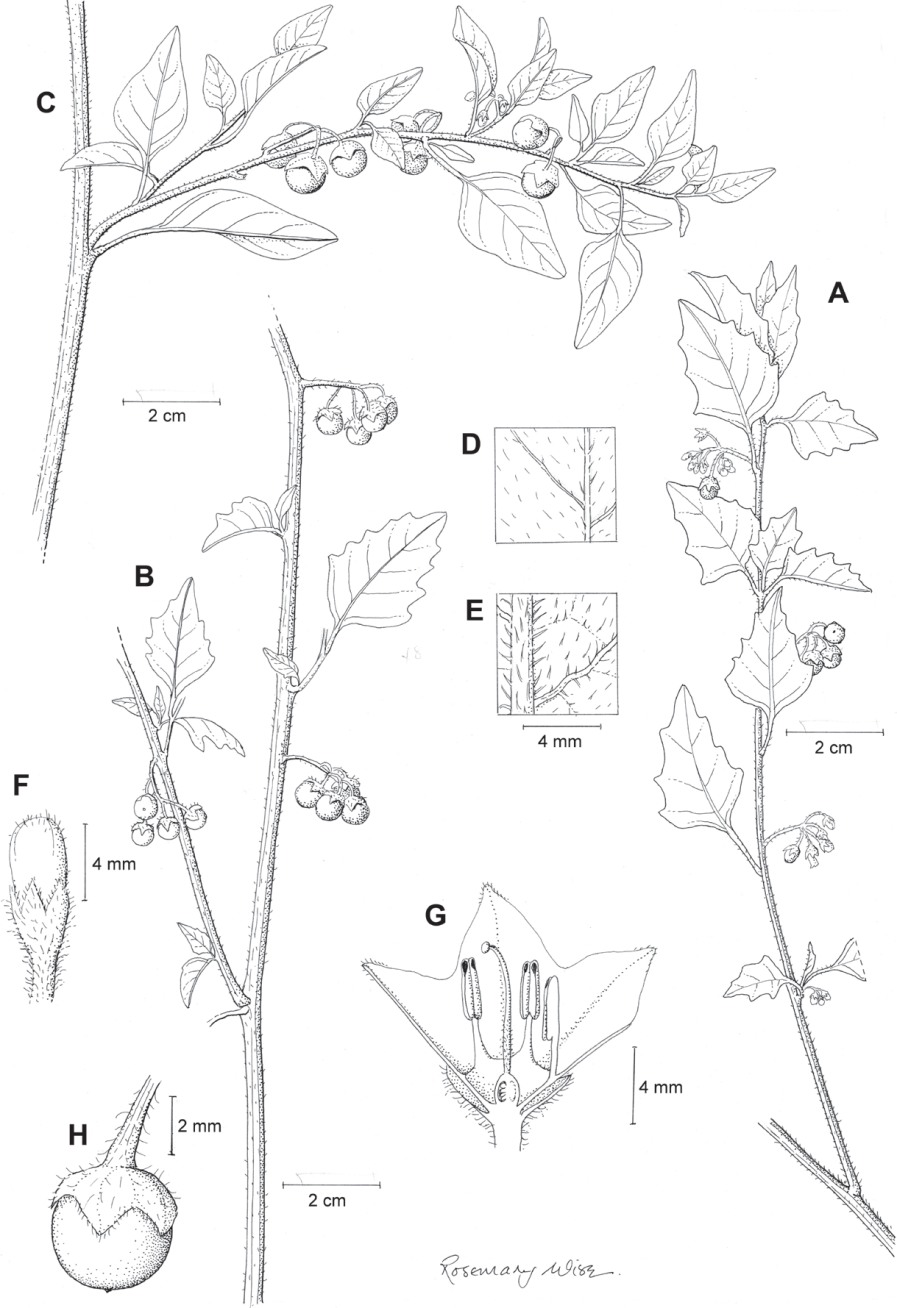
*Solanumnitidibaccatum***A** habit **B** fruiting habit **C** fruiting habit showing leaf variation **D** detail of adaxial leaf surface **E** detail of abaxial leaf surface **F** flower bud **G** dissected flower **H** fruit (**A, C, F***Henning 14***B, D, E, H***Blake 186***C***Arnow 740*). Illustration by R. Wise. Previously published in Särkinen et al. (2018: 107) and Knapp et al. (2019: 92).

**Figure 105.**
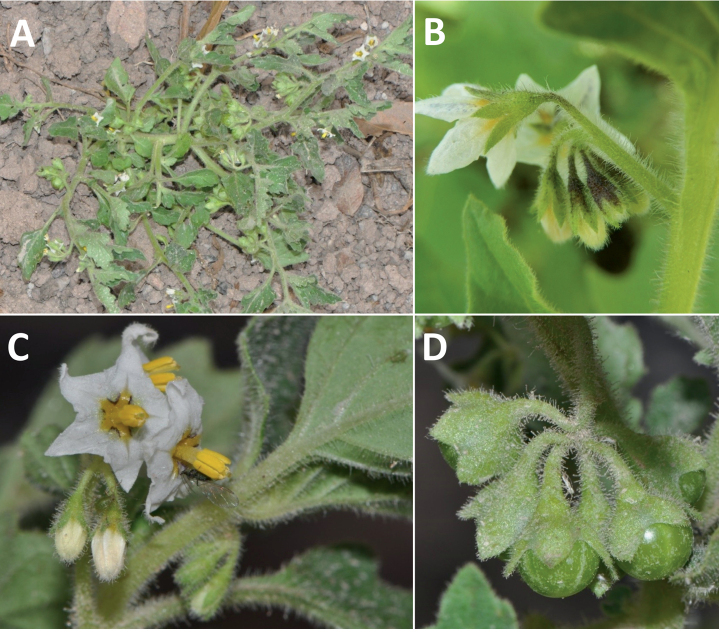
*Solanumnitidibaccatum***A** habit **B** young inflorescence with flower buds **C** flowers at anthesis **D** maturing fruits (**A–D***Särkinen et al. 4076*). Photos by T. Särkinen. Previously published in Särkinen et al. (2018: 108) and Knapp et al. (2019: 93).

Figs 104 , 105 Types based on American specimens only; for full synonymy, see Särkinen et al. (2018: 105–106)

Solanum stylesanum Dunal, Prodr. [A. P. de Candolle] 13(1): 44. 1852, nom rej. prop., as ‘*styleanum*’ Type. Chile. Sin. loc., *J. Styles s.n.* (holotype: G-DC [G00144016]).
Bosleria nevadensis A.Nelson, Proc. Biol. Soc. Washington 18(30): 175. 1905, nom rej. prop. Type. United States of America. Nevada: Washoe County, Pyramid Lake, 9 Jun 1903, *G.H. True s.n.* (holotype: RM [RM0004387]).
Solanum patagonicum C.V.Morton, Revis. Argentine Sp. Solanum 146. 1976. Type. Chile. Región XII (Magallanes): Río Paine, 100 m, 15 Jan 1931, *A. Donat 415* (holotype: BM [BM000617673]; isotypes: BA, BAF, GH [GH00077732], K, SI [003331, 003332], US [00027733, acc. # 2639758]).
Solanum physalifolium Rusby var. nitidibaccatum (Bitter) Edmonds, Bot. J. Linn. Soc. 92: 27. 1986. Type. Based on Solanumnitidibaccatum Bitter.

##### Type.

Chile. Sin. loc., 1829, *E.F. Poeppig s.n.* (lectotype, designated by Edmonds 1986, pg. 27: W [acc. # 0004151]; isolectotype: F [v0073346F, acc. # 875221]).

##### Description.

Annual herbs to 0.2 m high, prostrate and spreading to 0.3 m in diameter or more. Stems terete, green, not markedly hollow; new growth densely viscid-pubescent with translucent simple, uniseriate 2–8(10)-celled spreading trichomes 1.5–2 mm long with a glandular apical cell; older stems glabrescent. Sympodial units difoliate, the leaves not geminate. Leaves simple and shallowly toothed, the blades 2–5.5(–9.5) cm long, 1.5–5(–6.5) cm wide, ovate to broadly ovate, rarely elliptic, widest in the lower half to third, membranous, discolorous; adaxial surface sparsely pubescent with spreading 2–4-celled translucent, simple, uniseriate gland-tipped trichomes like those of the stem, these denser along the veins; abaxial surface more evenly densely pubescent on the lamina and veins; major veins 3–6 pairs, not clearly evident abaxially; base attenuate to cuneate, at times asymmetric, decurrent on the petiole; margins entire or sinuate-dentate; apex acute to obtuse; petioles 0.5–2.7(–4.5) cm long, sparsely pubescent with simple uniseriate glandular trichomes like those of the stems and leaves. Inflorescences generally internodal, but in new growth appearing to arise opposite the leaves, unbranched, 1–2 cm long, with 4–8(–10) flowers clustered at the tip (sub-umbelliform) or spread along a short flower-bearing portion of the axis, sparsely pubescent with spreading trichomes like those on stems and leaves; peduncle 0.6–1.3 cm long; pedicels 4–12 mm long, 0.1–0.2 mm in diameter at the base and 0.2–0.4 mm in diameter at the apex, straight and spreading, articulated at the base; pedicel scars spaced 0.3–1 mm apart. Buds subglobose, the corolla only slightly exserted from the calyx tube before anthesis. Flowers 5-merous, cosexual (hermaphroditic). Calyx tube 1–2 mm long, conical, the lobes 1.7–2.5 mm long, less than 1 mm wide, triangular with acute to obtuse apices, sparsely pubescent with 1–4-celled glandular trichomes like those of the pedicels. Corolla 0.4–0.6 cm in diameter, white with a yellow-green central eye with black “V” or “U” shaped edges in the lobe sinuses, rotate-stellate, lobed 1/3 of the way to the base, the lobes 2.3–3.2 mm long, 2.5–3.7 mm wide, spreading at anthesis, sparsely papillate-pubescent abaxially with 1–4-celled simple uniseriate trichomes, especially along tips and midvein. Stamens equal; filament tube minute; free portion of the filaments 1.5–2 mm long, adaxially sparsely pubescent with tangled uniseriate 4–6-celled simple trichomes; anthers 1–1.4 mm long, 0.5–0.8 mm wide, ellipsoid, yellow, poricidal at the tips, the pores lengthening to slits with age and drying. Ovary globose, glabrous; style 2.5–3 mm long, straight, exserted beyond the anther cone, densely pubescent with 2–3-celled simple uniseriate trichomes in the lower half where included in the anther cone; stigma capitate, minutely papillate, green in live plants. Fruit a globose berry, 0.4–1.3 cm in diameter, brownish green and marbled with white (this not easily visible in herbarium specimens) at maturity, translucent, the pericarp usually shiny, somewhat translucent, glabrous; fruiting pedicels 4–13 mm long, ca. 0.2 mm in diameter at the base, spaced 1–3 mm apart, reflexed and slightly curving, not persistent; fruiting calyx accrescent, becoming papery in mature fruit, the tube ca. 3 mm long, the lobes 2.5–3.5(-4) mm long and 3–4 mm wide, appressed against the berry, but the berry clearly visible. Seeds 13–24 per berry, 2–2.2 mm long, 1.2–1.4 mm wide, flattened and teardrop shaped with a subapical hilum, brown, the surfaces minutely pitted, the testal cells pentagonal in outline. Stone cells usually (1-)2–3 per berry, occasionally absent, ca. 0.5 mm in diameter. Chromosome number: n = 12 (Moscone 1992, voucher *Ambrosetti & Moscone 1478*; Moyetta et al. 2013, voucher *Chiapella et al. 1840*, as *S.sarrachoides*).

##### Distribution

**Figure 106.**
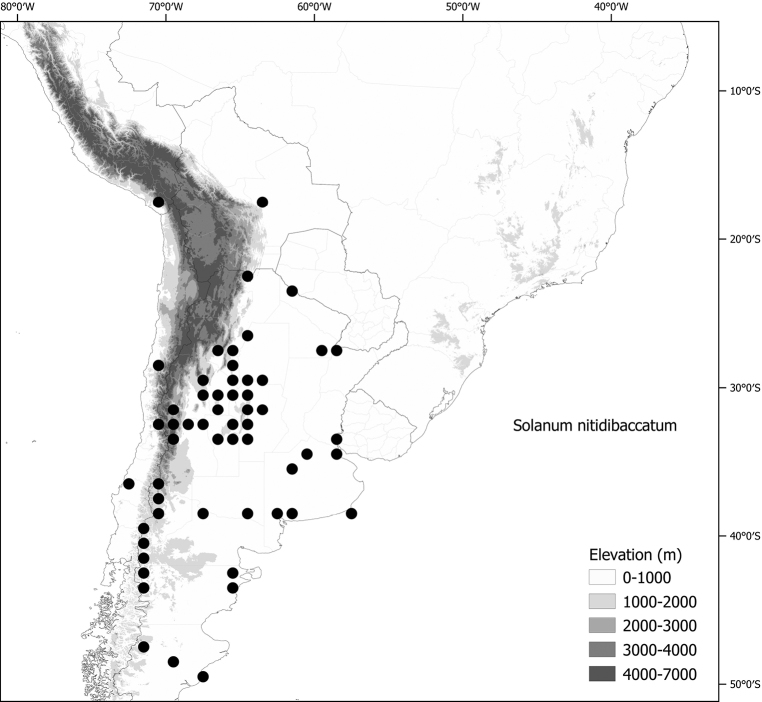
Distribution map of *Solanumnitidibaccatum* in South America. For distribution in North and Central America and the Caribbean, see Knapp et al. (2019: 95) and adventive distribution elsewhere, see Särkinen et al. (2018: 109).

**(Fig. 106).***Solanumnitidibaccatum* has an amphitropical distribution in temperate South America and temperate western North America, including northern Baja California (for distribution outside South America and a discussion of its native range see Särkinen et al. 2018, Knapp et al. 2019). In South America it appears to be native in Chile (Regions III [Atacama], V [Valparaiso], VI [O’Higgins], VIII [Bío-Bío], XII [Magellanes], XIII [Metropolitana], XIV [Los Ríos]), Argentina (Provs. Buenos Aires, Catamarca, Chubut, Córdoba, Entre Rios, La Rioja, Mendoza, Neuquén, Río Negro, Salta, San Juan, San Luis, Santa Cruz, Santiago del Estero, Tucumán) and coastal southern Peru (Depts. Moquegua, Tacna); it is perhaps adventive in Ecuador (Prov. Tungurahua).

##### Ecology and habitat.

*Solanumnitidibaccatum* is a species that inhabits disturbed areas, usually found growing along roadsides in the shade of trees and shrubs, and in rocky and sandy soils between (0-) 1,200 and 2,500 m elevation. It is a common weed of agriculture and is often found growing in sandy soil in seasonal washes (arroyos).

##### Common names and uses.

No common names or uses have been recorded from South American specimens (for common names in North America, see Knapp et al. 2019). The many uses of *S.nitidibaccatum* by indigenous peoples of North America are documented in Knapp et al. (2019). In Argentina, the Mapuche people use *S.nitidibaccatum* medicinally for treatment of gastrointestinal and liver complaints (Molares and Ladio 2012).

##### Preliminary conservation status

**(IUCN 2022).** Least Concern [LC]. EOO = 188,100,484 km^2^ [LC]; AOO = 1,824 km^2^ [EN]; calculated on the global range. *Solanumnitidibaccatum* is widespread and weedy throughout its range. In North America it is considered a noxious weed of agriculture (see Knapp et al. 2019).

##### Discussion.

*Solanumnitidibaccatum* is morphologically similar to and has been treated as *S.sarrachoides* in many previous treatments (e.g., Schilling 1981; Schilling and Heiser 1979); it is also often treated as *S.physalifolium*. *Solanumnitidibaccatum* has also sometimes been treated at infraspecific rank within *S.physalifolium*, an Andean endemic; the species are distinct and not closely related (Gagnon et al. 2022). *Solanumnitidibaccatum* is usually thought to be native to Patagonian South America, from which it has been introduced extensively to other parts of the world where it has become a prolific and successful weed of disturbed sites. The species is locally abundant throughout North America and is probably native there west of the Rockies (see Knapp et al. 2019), as evidenced by its extensive use by local people.

*Solanumnitidibaccatum* can be distinguished from *S.sarrachoides* in its shorter, plumper anthers, the blackish purple markings in the centre of the corolla on the margins of the central star, and in its fruits that are shiny at maturity, marbled with white (not usually visible on herbarium sheets) and not completely enclosed in the accrescent calyx. In addition, the mature inflorescences of *S.nitidibaccatum* are always internodal while those of *S.sarrachoides* are usually opposite the geminate leaves. Edmonds (1986) showed that *S.nitidibaccatum* and *S.sarrachoides* were distinct morphologically and phylogenetic results confirm this, showing these two species are not closely related despite their overall similarity (Särkinen et al. 2015b; Gagnon et al. 2022).

*Solanumnitidibaccatum* can be distinguished from other glandular-pubescent morelloids by its minute flowers, with anthers 1–1.5 mm long. *Solanummarmoratum*, with which *S.nitidibaccatum* is sympatric, has similarly tiny flowers, but lacks glandular pubescence completely and has much larger, more distinctly marbled berries.

Details of typification of the synonyms of *S.nitidibaccatum* can be found in Barboza et al. (2013) and Särkinen et al. (2018). The name *S.nitidibaccatum* has been proposed for conservation (Särkinen and Knapp 2022) due to its widespread usage in the weed literature.

#### ﻿35. Solanum palitans

Taxon classificationPlantaeSolanalesSolanaceae

C.V.Morton, Revis. Argentine Sp. Solanum 92. 1976.

Figs 107 , 108

##### Type.

Argentina. Tucumán: Dpto. Tafí del Valle, Yerba Buena, 19 Jan 1919, *S. Venturi 159* (holotype: US [00027724, acc. # 1548805]; isotypes: BA [acc. # 2463], LIL [LIL001454], LP [LP010926], MA, SI [003329]).

**Figure 107.**
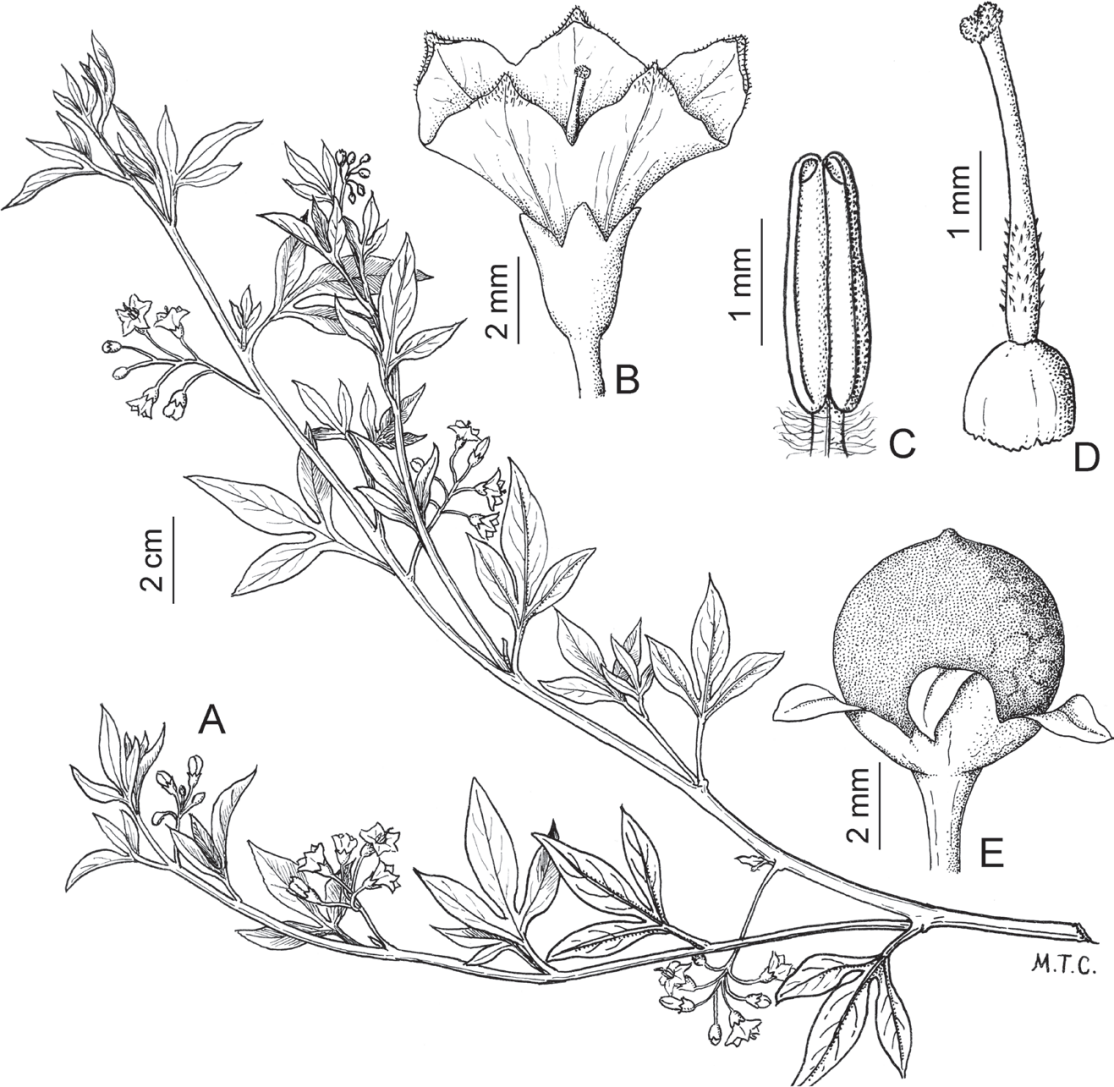
*Solanumpalitans***A** habit **B** flower **C** stamen, ventral view **D** gynoecium **E** fruit (**A–E** voucher details missing). Illustration by M.T. Cabrera. Previously published in Barboza et al. (2013: 252).

##### Description.

Annual, decumbent or prostrate herbs, the young plants sometimes erect, up to 0.2 m high often rooting at the lower nodes, forming dense patches, the branches to ca. 1 m long. Stems decumbent or ascending, terete or somewhat angled with ridges, green, older stems yellowish-brown, not markedly hollow; new growth pubescent with simple, spreading, uniseriate, translucent, eglandular trichomes, these 0.5–1 mm long, glabrous or nearly so; older stems glabrous. Sympodial units difoliate, the leaves not geminate. Leaves simple and strongly 3-lobed, the blades 2.5–9 cm long, 2.5–7.5 cm wide, broadly ovate, widest in the lower third, thinly membranous, concolorous, without smell; adaxial surfaces glabrous to sparsely pubescent with simple hairs to 0.5 mm on the major veins; abaxial surfaces glabrous; major veins 3–4 pairs; base long attenuate, decurrent on the petiole; margins 3-lobed nearly to the midrib, rarely the lateral lobes themselves lobed, the terminal lobe ovate, the lateral lobes asymmetrically ovate or lanceolate-ovate, acute at the tips, the sinuses sometimes sparsely ciliate; apex acute; petioles 0.5–2 cm, winged to the base, glabrous or sometimes sparsely ciliate near the base. Inflorescences internodal or often just below a node, unbranched or rarely forked, 1.2–2.5 cm long, with 4–9 flowers, glabrous to sparsely pubescent; peduncle 0.7–1.4 cm long, delicate; pedicels 3–5 mm long, 0.2–0.3 mm in diameter at the base and at the apex, filiform, spreading, articulated at the base; pedicel scars spaced 1–5 mm apart. Buds ellipsoid, the corolla completely covered by the calyx tube before anthesis. Flowers 5-merous, cosexual (hermaphroditic). Calyx tube 1.5–2 mm long, cup-shaped, the lobes ca. 0.75–1.5 mm long, lanceolate-oblong, glabrous, the tips acute. Corolla ca. 0.7 cm in diameter, white or rarely light violet, rotate-stellate, lobed ca. 1/2 way to the base, the lobes 1.5–2.5 mm long, 1–2 mm wide at the base, reflexed or spreading at anthesis, abaxially minutely white-puberulent on the tips of the lobes, glabrous adaxially. Stamens equal; filament tube minute; free portion of the filaments 0.5–1 mm long, adaxially pubescent with tangled uniseriate trichomes; anthers 1.6–2 mm long, 0.7–0.8 mm wide, oblong or ellipsoid, yellow, poricidal at the tips, the pores lengthening to slits with age and drying. Ovary glabrous; style 2.3–3.3 mm long, straight exserted beyond the anther cone, glabrous or sparsely pubescent in the lower part,; stigma capitate, the surface minutely papillate, green in live plants. Fruit a depressed-globose and bilobed (especially when young) berry, 0.6–0.8 cm in diameter, pale yellow, the pericarp thin and somewhat shiny, opaque to somewhat translucent, glabrous; fruiting pedicels 4–7 mm long, 0.5–0.7 mm in diameter at the base, 0.5–0.7 mm in diameter at the apex, spreading, recurved at the base to hold the fruit downwards, often in contact with the soil, not persistent; fruiting calyx not markedly accrescent but the lobes somewhat elongating in fruit, the tube 2–3 mm long, the lobes 2–3(-4) mm long, covering the basal 1/3 of the berry, the tips somewhat recurved. Seeds 20–30 per berry, 1.5–1.6 mm long, 1.2–1.6 mm wide, flattened reniform, light yellow, the surfaces pitted, the testal cells sinuate in outline. Stone cells 2(-4) per berry, 2 larger and apical (1–1.5 mm in diameter), the other 2 equatorial, smaller, 0.5–0.6 mm in diameter, all pale cream-coloured. Chromosome number: n = 12 (Moyetta et al. 2013, voucher *Barboza et al. 2228*, as *S.tripartitum*, *Barboza et al. 2178*).

**Figure 108.**
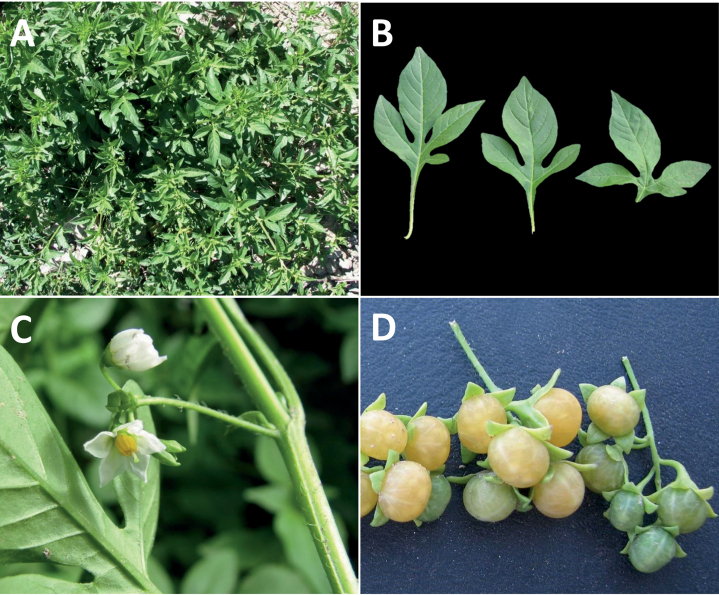
*Solanumpalitans***A** habit **B** leaves **C** flowers and inflorescence **D** fruits at different stages of maturity (**A–D***Barboza et al. 3471*). Photos by T. Särkinen.

##### Distribution

**Figure 109.**
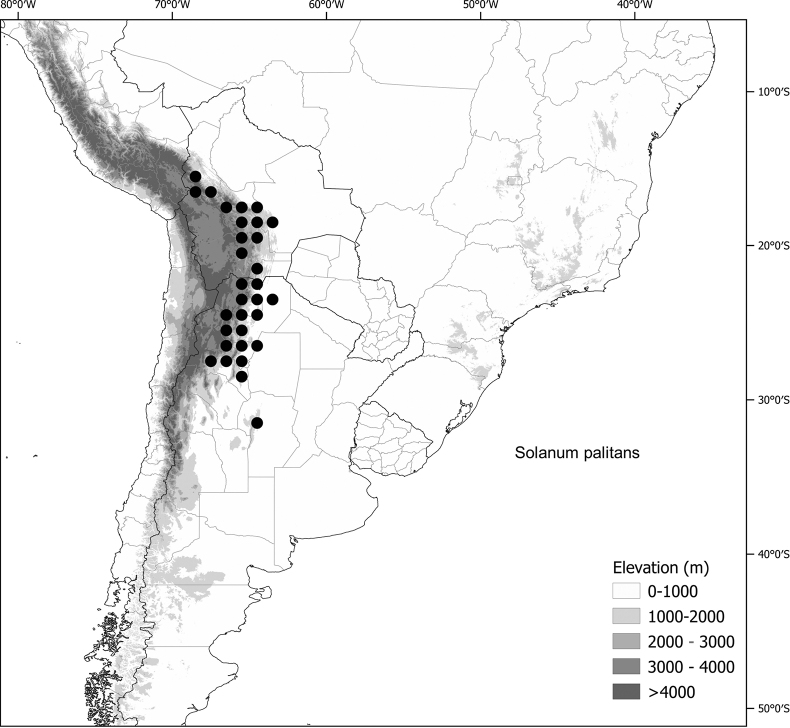
Distribution map of *Solanumpalitans*. For adventive distribution in Australia, see Särkinen et al. (2018).

**(Fig. 109).***Solanumpalitans* occurs in northwestern Argentina (Provs. Catamarca, Córdoba, Jujuy, Salta, Tucumán), northern Chile and Bolivia (Depts. Chuquisaca, Cochabamba, La Paz, Potosi, Santa Cruz); a very local naturalised population is known from New South Wales (Australia, see Särkinen et al. 2018).

##### Ecology and habitat.

*Solanumpalitans* grows in disturbed sites, along roadsides and field margins, on rocky, sandy, or clay soils; between (50–)1,400 and 3,000(–3,700) m elevation.

##### Common names and uses.

Argentina. Jujuy, Tucumán: ñusco (Hilgert and Gil 2006; Ceballos and Perea 2014; Acosta et al. 2018); Salta: ñusco blanco (Califano 2020). Leaves are used medicinally to soothe pain from blows and as a febrifuge (Ceballos and Perea 2014), and as animal fodder (Califano 2020).

##### Preliminary conservation status

**(IUCN 2022).** Least Concern [LC]. EOO = 1,008,132 km^2^ [LC]; AOO = 468 km^2^ [EN]; calculated excluding adventive Australian range. *Solanumpalitans* is a widespread species in its native range. It grows in open disturbed areas and has been collected in at least one protected area in Argentina (e.g., Parque Nacional Calilegua).

##### Discussion.

*Solanumpalitans* is morphologically similar to *S.tripartitum* and closely related to it (Särkinen et al. 2015b) The two species are sympatric and will apparently hybridise in the field (see below). With *S.corymbosum* and *S.radicans*, these two taxa form the distinct Radicans clade (Särkinen et al. 2015b), distinguished by their usually divided leaves and bright orange or red berries. *Solanumpalitans* has a creeping habit, with stems growing close to the ground extending up to 3 m and often rooting at nodes. *Solanumtripartitum* is an upright plant, the base not rooting if decumbent, with erect and branched inflorescences. *Solanumpalitans* has unbranched inflorescences, whereas those of *S.tripartitum* are usually branched several times. The berries of *S.palitans* are yellow or pale yellow and often held near the soil surface, while those of *S.tripartitum* are bright red when ripe and not so disposed. Both species have two large apical stone cells in the berries.

*Solanumpalitans* is very easily confused in the herbarium with *S.tripartitum* and the species are mixed under the same collection number in some cases. There are apparently hybrids, at least in Bolivia, between the two taxa. Michael Nee (pers. comm.) selected forty individual plants more or less at random from an area of ruderal vegetation on dry rocky slopes and gravelly stream beds in Achumani, a suburb of the City of La Paz, Bolivia; 25 proved to be *S.tripartitum* (*Nee 32057a–y*), 11 were *S.palitans* (*Nee 32058a–k*), and four seemed to be intermediate (*Nee 32058a–d*). The plants of *Nee 32058a–d* were similar to *S.palitans*, but had the branched inflorescences of *S.tripartitum*. *Nee & Solomon 34175* has also been suggested to be a hybrid plant by M. Nee (pers. comm.).

*Solanumradicans* differs from both *S.palitans* and *S.tripartitum* in its 5-lobed leaves, in its combination of a generally upright habit and orange or orange-yellow berries and its more northerly distribution. *Solanumcorymbosum* has entire leaves.

#### ﻿36. Solanum pallidum

Taxon classificationPlantaeSolanalesSolanaceae

Rusby, Mem. Torrey Bot. Club 4: 228. 1895.

Figs 2F , 110 , 111

Solanum lechleri Rusby, Bull. Torrey Bot. Club 26: 193. 1899. Type. Bolivia. La Paz: Prov. Larecaja, Guanai [Guanay], May 1886, *H.H. Rusby 790* (no herbaria cited; lectotype, designated here: NY [00172060]; isolectotypes: GH [00077701], NY [00172059, 00743694], PH [00030433], US [00027648, acc. # 1416231; 01014267, acc. # 32604]).
Solanum lilacinum Rusby, Bull. Torrey Bot. Club 26: 192. 1899. Type. Bolivia. La Paz: Prov. Nor Yungas, Unduavi, Oct 1885, *H.H. Rusby 779* (no herbaria cited; lectotype, designated here: NY [00172067]; isolectotypes: BM [BM000778229], NY [00172068, 00172069], US [00027653, acc. # 32597], WIS [v0256196WIS]).
Solanum rosulatum Rusby, Bull. New York Bot. Gard. 4: 418. 1907. Type. Bolivia. Sin. loc., [no date], *M. Bang 2518* (no herbaria cited; lectotype, designated here: NY [00172157]; isolectotype: US [00027779, acc. # 1324745]).
Solanum symmetrifolium Rusby, Bull. New York Bot. Gard. 4: 418. 1907. Type. Bolivia. Sin loc., [no date], *M. Bang 2870* (no herbaria cited; lectotype, designated here: NY [00172200]; isolectotypes: K [K000585654, K000585655], NY [00172201]).
Solanum sarachioides Rusby, Bull. New York Bot. Gard. 4: 420. 1907, nom illeg., non Solanumsarrachoides Sendtn. (1846). Type. Bolivia. Sin. loc., [no date], *M. Bang 2517* (no herbaria cited; lectotype, designated here: NY [00172168]; isolectotype US [00027789, acc. # 1416169]).
Solanum buchtienii Bitter, Repert. Spec. Nov. Regni Veg. 10: 558. 1912. Type. Bolivia. La Paz: Prov. Nor Yungas, Unduavi, 12 Feb 1907, *O. Buchtien 765* (no herbaria cited; lectotype, designated here: HBG [HBG-511410]).
Solanum subauriferum Bitter, Repert. Spec. Nov. Regni Veg. 10: 559. 1912. Type. Bolivia. La Paz: Prov. Sud Yungas, Sirupaya prope Yanacachi, 22 Nov 1906, *O. Buchtien 332* (lectotype, designated here: US [00027813, acc. # 1175818, as “322”]; isolectotypes: NY [00824366], WRSL).
Solanum scotinonectarium Bitter, Repert. Spec. Nov. Regni Veg. 10: 560. 1912. Type. Bolivia. La Paz: Prov. Sud Yungas, “Sirupay bei Yanacachi”, 22 Nov 1906, *O. Buchtien 332* (lectotype, designated here: US [00027813, acc. # 1175818]; isolectotype: NY [00824366], WRSL [not seen]).
Solanum planifurcum Bitter, Repert. Spec. Nov. Regni Veg. 11: 2. 1912. Type. Peru. Puno: Prov. Sandia, sin. loc., 2,100–2,500 m, 6 Apr 1902, *A. Weberbauer 685* (holotype: B, destroyed [F neg. 2631]; lectotype, designated here: F [v0076175F, acc. # 647966, fragment of holotype).
Solanum sandianum Bitter, Bot. Jahrb. Syst. 50, Beibl. 111: 62. 1913. Type. Peru. Puno: Prov. Sandia, supra Cuyocuyo, 3,800 m, *A. Weberbauer 930* (holotype B, destroyed [F. neg. 2636]; no duplicates found); Peru. Puno: Prov. Sandia, km 137 on road from Cuyocuyo to Quiscupunco, 3641 m, 21 Mar 2012, *T. Särkinen, A. Mathews & P. Gonzáles 4055* (neotype, designated here: USM [acc. # 00265491]; isoneotypes: BM [BM001120017, BM001120240, BM001120241]).

##### Type.

Bolivia. Vic. La Paz, *M. Bang 64* [a] (no herbaria cited; lectotype, designated here: NY [00172111]; isolectotypes: GH, LE, M [M-0165963], MO [MO-503708, acc. # 1815483], NY [00172112], PH [00030385], US [00027725, acc. # 58341).

**Figure 110.**
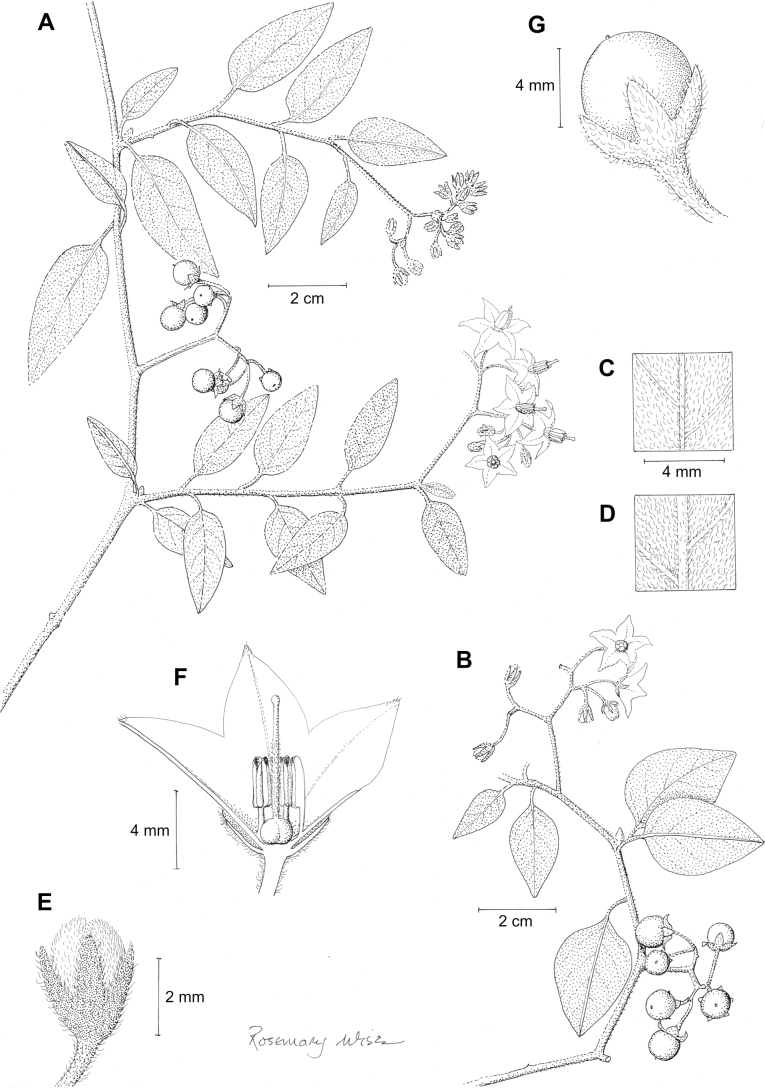
*Solanumpallidum***A** habit **B** habit with larger leaves **C** detail of adaxial leaf surface **D** detail of abaxial leaf surface **E** flower bud **F** dissected flower **G** fruit (**A, C–G***Knapp et al. 10445***B***Knapp et al. 10444*). Illustration by R. Wise.

##### Description.

Scandent or lax shrub 1–3 high, with elongate branches. Stems terete, densely pubescent with transparent eglandular dendritic uniseriate trichomes 0.5–1 mm long; new growth densely pubescent with transparent eglandular dendritic uniseriate trichomes 0.5–1 mm long, these drying white or yellowish white in herbarium specimens; bark of older stems greenish brown, somewhat glabrescent. Sympodial units difoliate to plurifoliate, the leaves not geminate. Leaves simple, the blades 2.5–10 cm long, 1–5.2 cm wide, elliptic to ovate-elliptic, widest at the middle or in the lower third, membranous, discolorous; adaxial surfaces moderately and evenly pubescent with transparent dendritic uniseriate trichomes to 0.5 mm long; abaxial surfaces sparsely to densely pubescent with transparent eglandular dendritic uniseriate trichomes to 0.5 mm long, thinner and more delicate than the trichomes of the adaxial surfaces; principal veins 6–8 pairs, often drying yellowish tan; base cuneate to acute; margins entire; apex acute to acuminate; petioles 0.8–1 cm long, adaxially pubescent like the upper leaf surfaces. Inflorescences internodal or terminal at branch tips, forked to several times branched, 3–9(12) cm long, with 20–40 flowers clustered at the branch tips, moderately to densely pubescent with transparent eglandular dendritic uniseriate trichomes to 0.5 mm long like those of the stems; peduncle 1.5–4(6) cm long; pedicels 1–1.5 cm long, ca. 0.75 mm in diameter at the base, ca. 1.5 mm in diameter at the apex, tapering, spreading at anthesis, moderate to densely pubescent with transparent eglandular dendritic uniseriate trichomes to 0.5 mm long, articulated at the base; pedicel scars tightly packed at the ends of the inflorescence branches, 1–1.5 mm apart. Buds ellipsoid, the corolla strongly exserted from the calyx before anthesis, usually darker than the corolla in flower. Flowers 5-merous, cosexual (hermaphroditic). Calyx tube 1.5–2 mm long, conical, the lobes 1.5–2.5 mm long, ca. 1 mm wide, triangular, sometimes somewhat reflexed at anthesis, moderately pubescent with transparent eglandular dendritic trichomes to 0.5 mm long, like those of the pedicels. Corolla 2–2.3 cm in diameter, pale to dark purple with a green eye, stellate, lobed ca. halfway to the base, the lobes 6–9 mm long, 4–6 mm wide, deltate, spreading at anthesis, adaxially glabrous except for the papillate lobe tips, abaxially puberulent with white eglandular simple and dendritic trichomes where exposed in bud, these denser on the midveins and tips, the interpetalar tissue more glabrous. Stamens equal; filament tube minute to 0.1 mm long; free portion of the filaments 1–1.5 mm long, very densely pubescent adaxially with tangled simple uniseriate trichomes. Ovary conical, glabrous; style 6–8 mm long, straight, exserted beyond the anther cone, densely pubescent in the lower part with transparent simple uniseriate trichomes to 0.5 mm long; stigma small-capitate, the surfaces minutely papillate. Fruit a globose berry, 0.8–1 cm in diameter, green when immature, ripening to blackish purple, the pericarp thin, matte, opaque, glabrous; fruiting pedicels 1.3–1.4 cm long, ca. 1.2 mm in diameter at the base and apex, not markedly woody, deflexed, not persistent; fruiting calyx not markedly accrescent, the lobes to 4 mm long and slightly reflexed at the tips, the tube appressed to the berry. Seeds ca. 20 per berry, ca. 2 mm long, ca. 1.5 mm wide, flattened and teardrop shaped, reddish gold, the surfaces minutely pitted, the testal cells sinuate in outline. Stone cells 6 per berry, scattered through the mesocarp, ca. 1 mm in diameter, cream-coloured, two of the inclusions slightly smaller. Chromosome number: 2n = 24 (Chiarini et al. 2017, voucher *Särkinen et al. 4014*).

**Figure 111.**
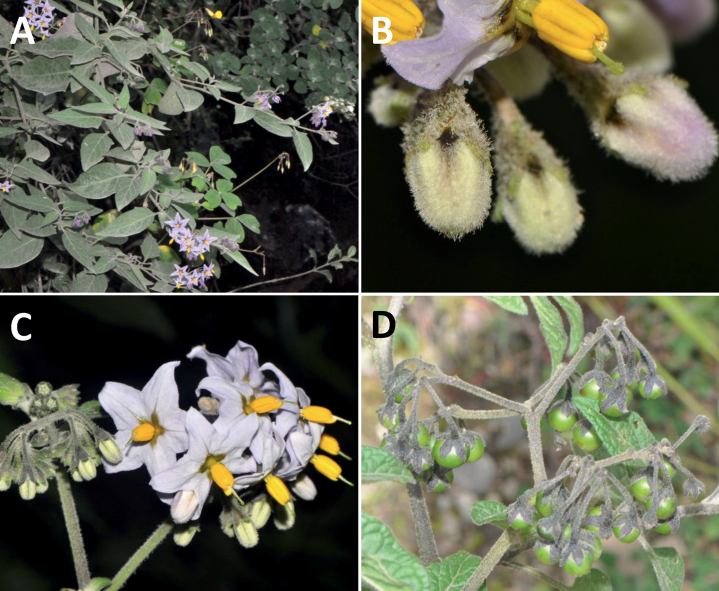
*Solanumpallidum***A** habit **B** flower buds with dense indumentum of dendritic trichomes **C** flowers at full anthesis **D** maturing fruits (**A***Särkinen et al. 4010***B***Knapp et al. 10433***C***Särkinen et al. 4042***D***Särkinen et al. 4010*). Photos by S. Knapp and T. Särkinen.

##### Distribution

**Figure 112.**
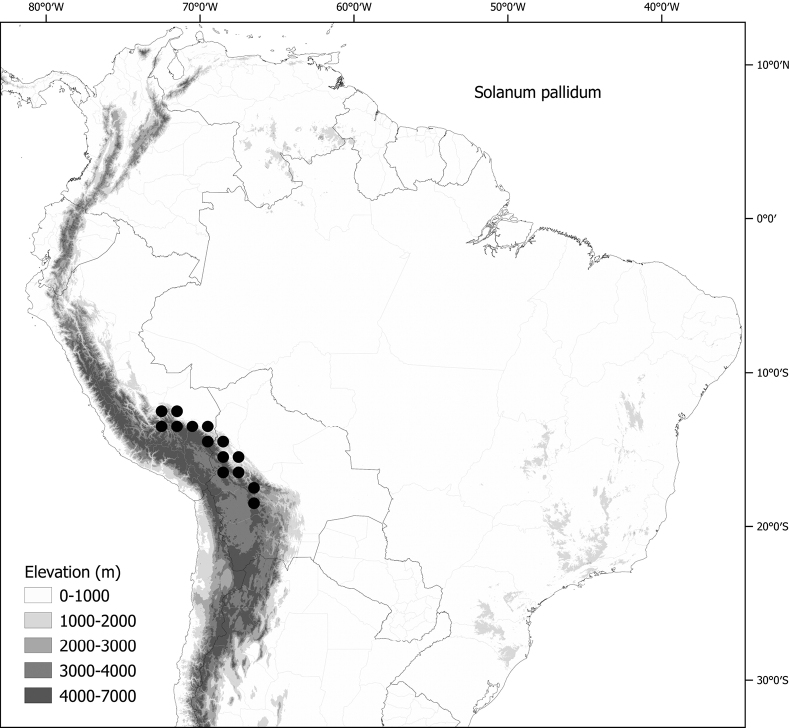
Distribution map of *Solanumpallidum*.

**(Fig. 112).***Solanumpallidum* occurs from south-central Peru (Depts. Huánuco, Ayacucho, Cusco, Puno) to northern Bolivia (Depts. Cochabamba, La Paz) on the eastern Andean slopes.

##### Ecology and habitat.

*Solanumpallidum* grows in cloud forests, cloud forest edges and clearings, roadsides and montane scrub, from (600-)1,200 to 4,000 m elevation. Most specimens have been collected between 2,000 and 2,800 m elevation.

##### Common names and uses.

Peru. Cusco: muyuqhaya (*Särkinen et al. 5284*). No uses recorded.

##### Preliminary conservation status

**(IUCN 2022).** Least Concern [LC]. EOO = 140,455 km^2^ [LC]; AOO = 340 km^2^ [EN]. *Solanumpallidum* is a weedy shrub of landslides and road edges and has a relatively wide distribution; it occurs around many of the protected archaeological sites in the region of Cusco, Peru.

##### Discussion.

*Solanumpallidum* is morphologically similar to *S.cochabambense* in its large flowers and highly branched inflorescences usually on long peduncles but is easily distinguished from it by often dense pubescence composed of branched (dendritic) trichomes on all plant parts. The two taxa are somewhat sympatric, but *S.pallidum* is confined to the eastern Andean slopes, while *S.cochabambense* occurs on both slopes of the Andes in Peru. Molecular sequence data suggest the species are closely related (Gagnon et al. 2022).

The collection used to describe *S.pallidum* (*Bang 64*; Rusby 1895) is a mixed gathering of *S.pallidum* and *S.gonocladum* (see *S.gonocladum*). We have selected the sheet at NY (barcode 00172111) that has the protologue attached and is annotated by Rusby as the lectotype for *S.pallidum*. Care must be taken with assigned duplicates of *Bang 64* as isolectotypes, some sheets correspond to *S.bangii*, a synonym of *S.gonocladum*.

In subsequent years Rusby (1899, 1907) described several names we here recognise as synonyms of *S.pallidum* but cited no herbaria in the protologues We have lectotypified all of these with the specimens in NY (where the specimens that Rusby used are held) that best correspond to the protologues and are most complete (see above).

Bitter (1912a) described *S.buchtienii* citing *Buchtien 765* with the date of 12 Feb 1907 but no herbarium; of the duplicates we have seen, only that in HBG (HBG-511410) has that number and the correct date; we designate it here as the lectotype. Other collections (e.g., NY barcode 00139077 and US barcode 00027486, acc. # 700088) do not have the same collecting date (date of collection Nov 1910) and so are not part of the same gathering and thus are not isolectotypes. In the same publication Bitter described *S.scotinonectarium* citing *Bang 31 pro parte* from “herb. Berol.” and *Buchtien 332 pro parte* from “herb. Buchtien! Vratisl.!”. He often used different duplicates of the same collection to describe different taxa based on minor leaf shape and size differences; he also cited *Buchtien 332 pro parte* as the basis for *S.subauriferum* without citing a herbarium but saying “una cum *S.scotinonectarium*”. *Bang 31* at “herb. Berol” also forms the basis for his *S.irenaeum* (Bitter 1912a), a synonym of *S.polytrichostylum*. A sheet in US (barcode 00027813, acc. # 1175818) bears both the numbers 322 and 332 and is annotated “S.subauriferum Bitt. n. sp.” by Buchtien; we are interpreting the number 322 as an error to be corrected. In view of Bitter’s practice of using different sheets of the same gathering to describe different species, the destruction of the WRSL sheet of *Buchtien 332* and the Buchtien Herbarium being held at US (Morton and Stern 1966), we have designated the US sheet as the lectotype of both *S.scotinonectarium* and *S.subauriferum* making these names homotypic.

*Solanumsandianum* was described (Bitter 1913) citing a collection made by August Weberbauer in southern Peru (*Weberbauer 930*) held in Berlin (F. neg. 2636). That specimen is destroyed and we have found no duplicates, even at MOL where Weberbauer’s original herbarium is held. We thus neotypify this name with a modern collection made near the type locality of Cuyocuyo in Sandia Province (*Särkinen et al. 4055*) held in the Peruvian National Herbarium (USM).

#### ﻿37. Solanum paucidens

Taxon classificationPlantaeSolanalesSolanaceae

Bitter, Repert. Spec. Nov. Regni Veg. 11: 226. 1912.

Figs 113 , 114

Solanum diffusum Vell., Fl. Flumin. 83. 1829 [1825], nom.illeg., non Solanumdiffusum Ruiz & Pav. (1799). Type. Brazil. [Rio de Janeiro]: “campis apricis mediterraneis”; (lectotype, designated by Knapp et al. 2015, pg. 829: [illustration] Original parchment plate of Flora Fluminensis in the Manuscript Section of the Biblioteca Nacional, Rio de Janeiro [cat. no.: mss1198651_101] and later published in Vellozo, Fl. Flumin. Icon. 2: tab. 98. 1831).
Solanum maracayuense Bitter, Repert. Spec. Nov. Regni Veg. 11: 227. 1912. Type. Paraguay. Canindeyú: “Sierra de Maracayú”, Nov, *É. Hassler 5278* (holotype: B, destroyed; lectotype, designated here: G [G00306843]; isolectotypes: BM [BM000074095], G [G00306845], GH [00105865], K [K000532494], NY [00172082], P[P00753766, P00753765, P00337048], UC [950199])
Solanum rojasii Chodat, Bull. Soc. Bot. Genève, sér. 2, 8: 150. 1916. Type. Paraguay. Paraguarí: [Cerro] Acahay, *R. Chodat & W. Vischer 67* (lectotype, designated here: G [G00449278]; isolectotype: G [G00449243]).
Solanum maioranthum L.B.Sm. & Downs, Phytologia 10: 425. 1964. Type. Brazil. Santa Catarina: Rio do Rastro, 20 km west of Lauro Müller, lower and middle slopes of Rio do Rastro, *L.B. Smith & R.M. Klein 12338* (holotype: US [00067554, acc. # 2423800]; isotypes: K [K000590017], NY [00172074], R [R000129993], US [03272136, acc. # 2492258]).

##### Type.

Brazil. Rio de Janeiro: Theresopolis, Nov-Dec 1888, *J.T. de Moura 578* (holotype: B, destroyed [F neg. 2839]; lectotype, designated here: F [v0073360F, acc. # 621340, fragment of holotype]).

**Figure 113.**
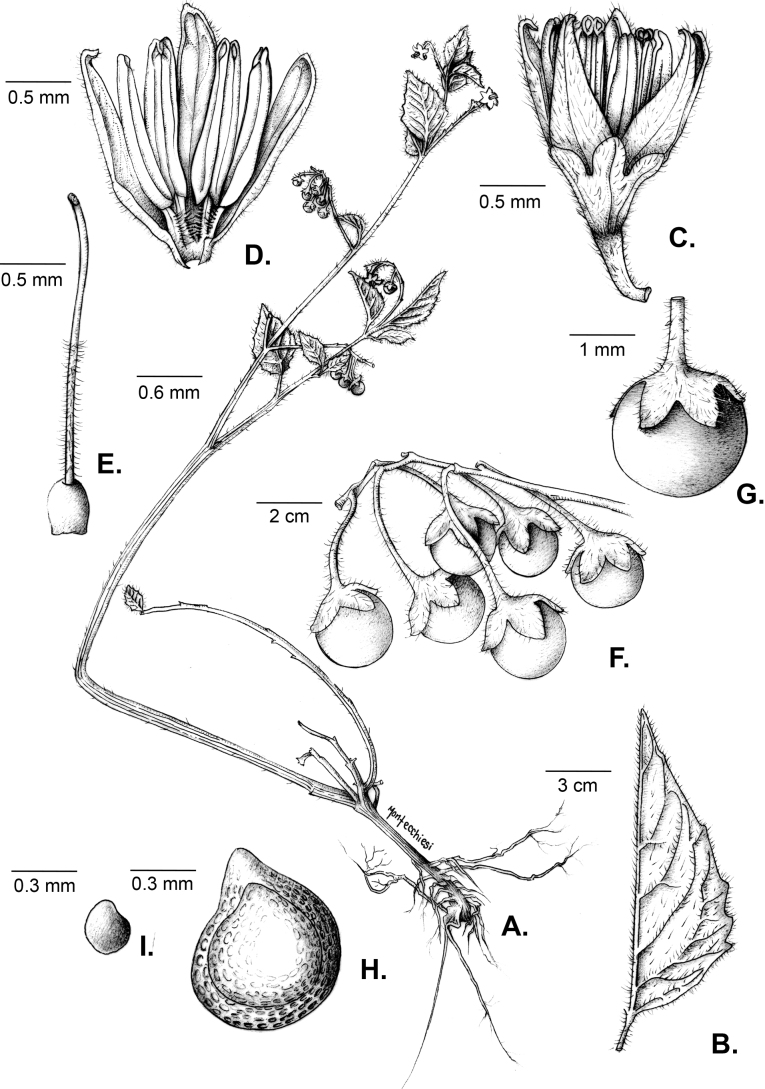
*Solanumpaucidens***A** habit **B** portion of a leaf, abaxial surface **C** flower **D** dissected flower **E** gynoecium **F** infructescence **G** fruit **H** seed **I** stone cell (**A–I***Gomes s.n.*). Illustration by S. Montecchiesi.

##### Description.

Herb to small subshrub with lax branches, 0.5–1.5 m high. Stems terete or slightly angled, sparsely pubescent with white eglandular 3–5-celled simple uniseriate trichomes ca. 0.5 mm long, also with a few spinescent processes along the angles; new growth densely white pubescent with eglandular 3–7-celled simple uniseriate trichomes ca. 0.5 mm long, these usually antrorse; bark of older stems pale greenish grey. Sympodial units difoliate, the leaves not geminate. Leaves simple or shallowly toothed, the blades 2–9(15) cm long, 1.5–3(5) cm wide, narrowly elliptic, widest at the middle or in the lower half, membranous, concolorous or slightly discolorous, very variable in size with lower leaves much larger; adaxial surfaces almost glabrous, with a few scattered white eglandular simple uniseriate trichomes ca. 0.5 mm long on the lamina, these somewhat denser along the veins; abaxial surfaces moderately and evenly pubescent with similar white eglandular simple uniseriate trichomes ca. 0.5 mm long; principal veins 4–6 pairs, more densely pubescent than the lamina; base acute; margins entire or with a few irregular teeth in the lower half; apex acute; petiole 0.5–0.9 cm long, pubescent with scattered white eglandular trichomes like those of the stem. Inflorescences internodal, unbranched or forked, 1.5–2.5 cm long, with 5–10 flowers not markedly clustered at the tips, moderately to sparsely pubescent with white eglandular simple uniseriate trichomes like those of the stems; peduncle 1.2–2.3 cm long; pedicels 0.6–0.7 cm long, 0.4–0.5 mm in diameter at the base, 1–1.2 mm in diameter at the apex, spreading or slightly deflexed and secund at anthesis, sparsely to moderately pubescent like the inflorescence axes, articulated at the base; pedicel scars more or less evenly spaced 0.5–1 mm apart along the axis, somewhat raised from the axis as small protuberances. Buds narrowly ellipsoid, the corolla strongly exserted from the calyx before anthesis. Flowers 5-merous, cosexual (hermaphroditic). Calyx tube 1–1.5 mm long, conical, the lobes 0.5–1 mm long, 0.5–1 mm wide, deltate to broadly triangular, often tearing irregularly, sparsely pubescent with white eglandular simple uniseriate trichomes like the inflorescence, but these sparser than on the pedicel. Corolla 0.8–1.2 cm in diameter, white with a central green star, lobed 2/3 to 3/4 of the way to the base, the lobes 3–4 mm long, 1.5–1.7 mm wide, spreading or reflexed at anthesis, glabrous adaxially, sparsely and evenly puberulent-papillate, more densely so on the tips and margins. Stamens equal; filament tube minute; free portion of the filaments 1–1.5 mm long, densely pubescent adaxially with tangled simple uniseriate trichomes; anthers 2.5–3.5 mm long, ca. 1 mm wide, ellipsoid, yellow, poricidal at the tips, the pores lengthening to slits with age. Ovary conical, glabrous; style 8–9 mm long, straight, exserted beyond the anther cone, densely pubescent with white simple uniseriate trichomes ca. 0.5 mm long in the lower half; stigma capitate, the surfaces minutely papillate. Fruit a globose berry, 0.8–1 cm in diameter, black or purple when ripe, the pericarp thin, somewhat shiny, translucent, glabrous; fruiting pedicels 0.7–1 cm long, ca. 0.7 mm in diameter at the base, ca. 1.2 mm at the apex, gradually tapering, not markedly woody, strongly deflexed at the base and the infructescence appearing secund in herbarium specimens, not persistent; fruiting calyx not enlarged or accrescent, appressed to the berry, the lobes tearing to become ca. 2 mm long. Seeds 40–80 per berry, 1.3–1.5 mm long, 1–1.2 mm wide, flattened and teardrop shaped, pale tan or yellow, the surfaces minutely pitted, the testal cells rectangular to slightly sinuate in outline, with short hair-like extensions of the lateral cell walls. Stone cells 2–6(8) per berry, 0.5–0.7 mm in diameter, cream-coloured. Chromosome number: not known.

**Figure 114.**
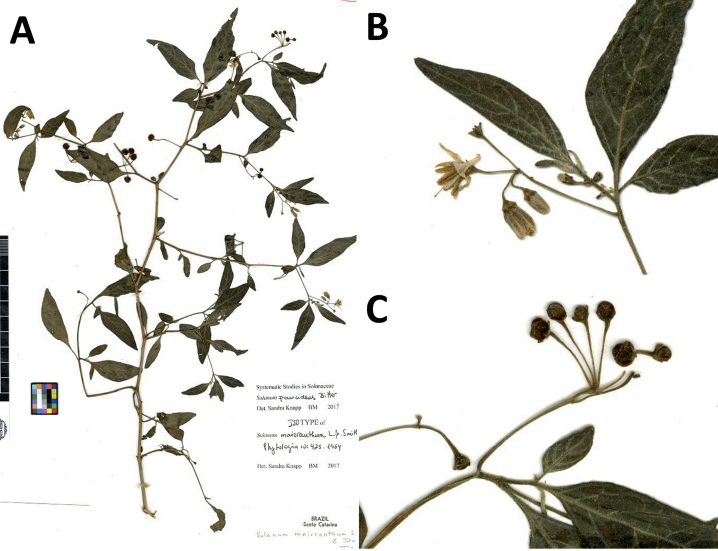
*Solanumpaucidens***A** habit **B** inflorescence **C** maturing fruits (**A–C***Smith & Klein 12338* [GH 01011894]). Reproduced with permission of the Gray Herbarium, Harvard University.

##### Distribution

**Figure 115.**
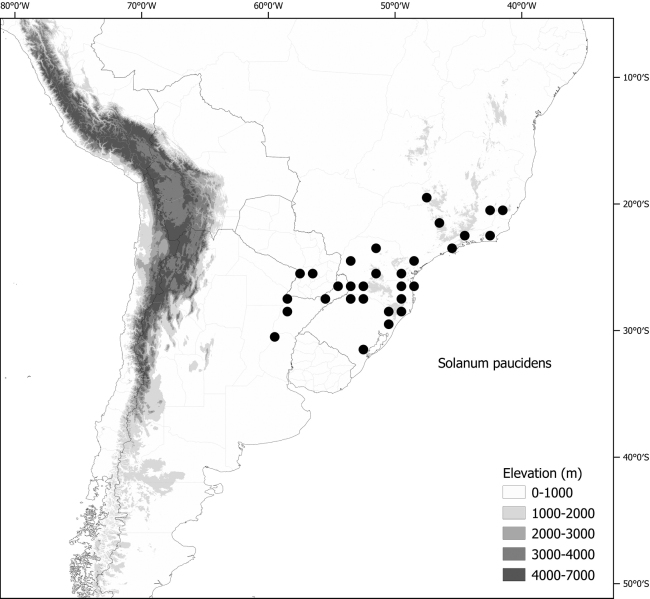
Distribution map of *Solanumpaucidens*.

**(Fig. 115).***Solanumpaucidens* occurs in southern Brazil (States of Espírito Santo, Mato Grosso, Minas Gerais, Paraná, Rio de Janeiro, Rio Grande do Sul, São Paulo, Santa Catarina), northeastern Argentina (Provs. Corrientes, Misiones) and eastern Paraguay (Depts. Alto Paraná, Central, Guairá).

##### Ecology and habitat.

*Solanumpaucidens* is a plant of middle to low elevations in the semideciduous and evergreen Mata Atlântica and Selva Paranense, growing in swampy areas, forest edges and clearings and forest understory, from near sea level to 2,000 m in the Atlantic forest mountains.

##### Common names and uses.

Brazil. Minas Gerais: erva-moura (*Souza et al. s.n.*), erva nome (*Andrade 1209*). Uses and common names attributed to *S.americanum* in Lorenzi and Abreu Matos (2021) and Ferreira Kinupp and Lorenzi (2022) may also apply to *S.paucidens*, although the photographs appear to be in large part *S.americanum*; there are no vouchers cited.

##### Preliminary conservation status

**(IUCN 2022).** Least Concern [LC]. EOO = 1,233,243 km^2^ [LC]; AOO = 196 km^2^ [EN]. *Solanumpaucidens* has a broad geographical distribution and is a weedy species of open areas and roadsides where it occurs. It occurs within protected areas in Argentina (e.g., Reserva Vida Silvestre Urugua-í in Misiones) and Brazil (e.g., Parque Nacional Itatiaia at the border of Minas Gerais, São Paulo and Rio de Janeiro, Parque Estadual Intervales in Santa Catarina, Parque Estadual Ibtipoca in Minas Gerais).

##### Discussion.

The name *S.paucidens* has not been in common use for this species, in part due to the poor quality of the type specimen (see below). Earlier treatments either treated this species as new (e.g., Smith and Downs 1966 as *S.maioranthum*) or as *S.nigrescens* (e.g., Mentz and Oliveira 2004), a species from North, Central and northern South America. The flowers of *S.paucidens* are usually evenly spaced along the elongate inflorescence axis, in contrast to *S.nigrescens* where they are clustered at the tip or not widely spaced. *Solanumpaucidens* is most similar to *S.enantiophyllanthum*, with which it is nearly sympatric in the mountain ranges of southern Brazil. *Solanumpaucidens* has smaller flowers (0.8–1.2 cm in diameter versus 1.9–2 cm in diameter) with shorter anthers (2.5–3.5 mm long versus 4.5–6 mm long) than *S.enantiophyllanthum* and grows at somewhat lower elevations where their distributions overlap. In herbarium specimens the inflorescences of *S.paucidens* appear secund, with flowers and fruits on one side of the inflorescence axis; this is most easily seen in fruiting specimens.

Bitter (1912b) described *S.paucidens* citing only a collection by “Julio” Moura (referring to Julia T. de Moura) from a specimen in the Berlin Herbarium. That specimen no longer exists, and we have looked for duplicates in other herbaria where collections might be found (e.g., BR), but have been unsuccessful. The fragment of the holotype at F mounted on the sheet with the photograph (F neg. 2839) taken by J.F. Macbride in Berlin (barcode v0073360F, acc. # 621340) is more or less adequate for identification and is here designated as the lectotype. Further duplicates of this gathering may be found at R that are better representations of the species, thus we do not at this stage designate an epitype.

In describing *S.maracayuense*Bitter (1912b) cited *Hassler 5278* from Berlin, but this specimen is no longer extant. Two sheets of *Hassler 5278* are held in the Hassler Herbarium at G, and we select the better of these with flowers and fruits (G00306843) as the lectotype for this name.

Chodat (1916) cited *Chodat & Visscher 67* from “herb. Univ. de Geneve” in the protologue of *S.rojasii*. This herbarium is now held at G, and we have selected the more complete of the two duplicates (G00449278) held there that is labelled “holotype” as the lectotype for *S.rojasii*.

#### ﻿38. Solanum pentlandii

Taxon classificationPlantaeSolanalesSolanaceae

Dunal, Prodr. [A. P. de Candolle] 13(1): 51. 1852.

Figs 3C , 116 , 117

Solanum coerulescens Bitter, Repert. Spec. Nov. Regni Veg. 10: 554. 1912. Type. Bolivia. La Paz: sin. loc., Apr 1910, *O. Buchtien 2965* (no herbaria cited; lectotype, designated here: US [00027517, acc. # 703363]; isolectotypes: GOET [GOET003565, GOET003566], NY [00139098, 00139099], US [00610913, acc. # 1175828]).
Solanum coerulescens Bitter var. manophyes Bitter, Repert. Spec. Nov. Regni Veg. 10: 554. 1912. Type. Bolivia. La Paz: Caminos, 4 Jan 1907, *O. Buchtien 769* (no herbaria cited; lectotype, designated here: US [00027518, acc. # 1175829]; isolectotypes: GH [00077600], GOET [GOET003568], NY [00139101], US [00610914, acc. # 1175823]).
Solanum coerulescens Bitter var. pycnophyes Bitter, Repert. Spec. Nov. Regni Veg. 10: 554. 1912. Type. Bolivia. La Paz: sin. loc., Apr 1910, *O. Buchtien 2966* (no herbaria cited; lectotype, designated here: US [00027519, acc. # 1175974]; isolectotypes: GOET [GOET003567]; NY [00139100], US [00610915, acc. # 703364]).
Solanum insulae-solis Bitter, Repert. Spec. Nov. Regni Veg. 10: 563. 1912. Type. Bolivia. La Paz: Lake Titicaca, Isla del Sol (“Sonneninsel”), Mar 1910, *O. Buchtien 5856* (no herbaria cited; lectotype, designated here: US [00650475, acc. # 1175976]).

##### Type.

Bolivia. “E of La Paz”, *J.B. Pentland s.n.* (holotype: G-DC [G00144345]; isotype: P [P00367413]).

**Figure 116.**
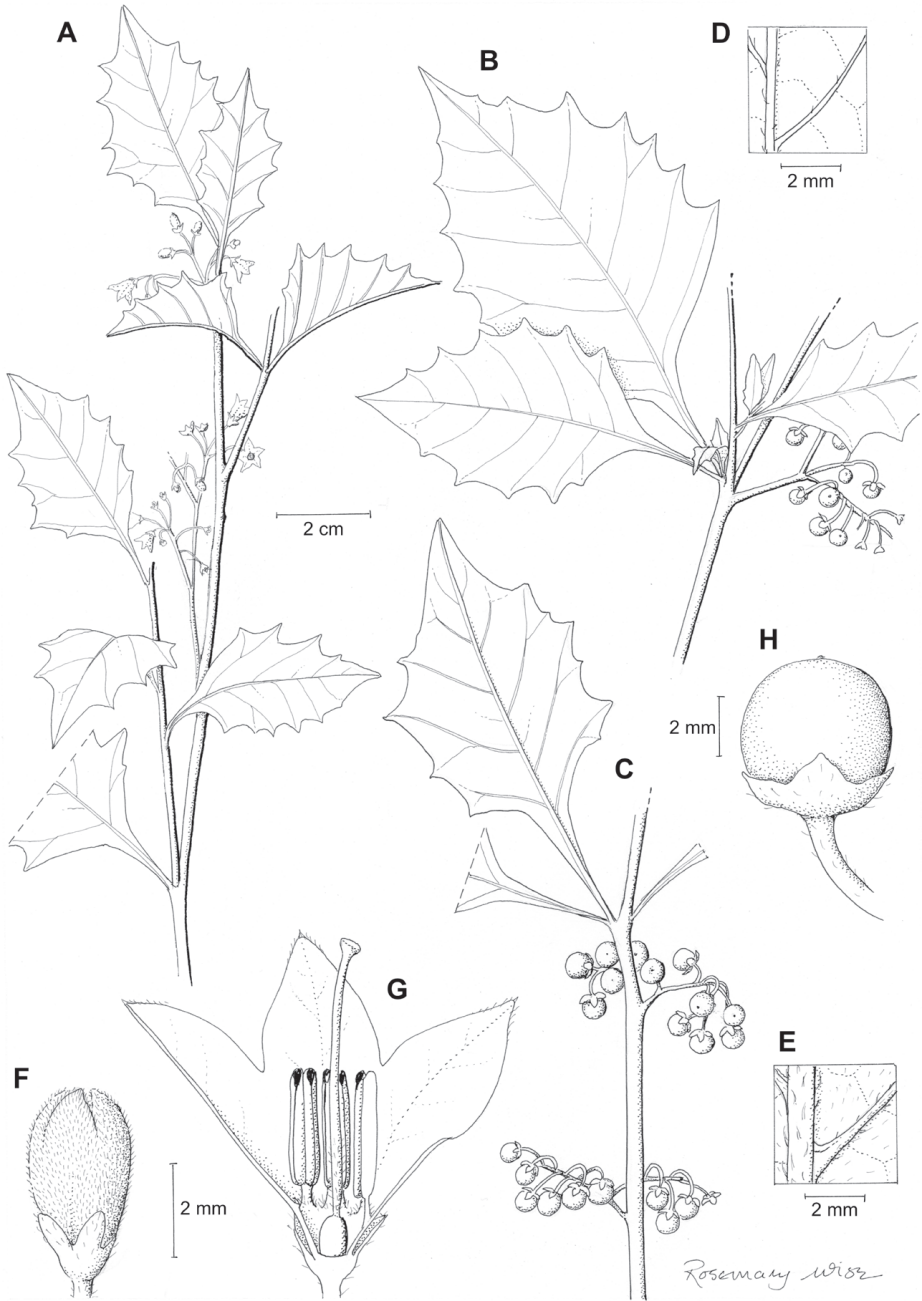
*Solanumpentlandii***A** flowering habit **B, C** fruiting habits **D** detail of adaxial leaf surface **E** detail of abaxial leaf surface **F** flower bud **G** dissected flower **H** fruit (**A, B**, **D, F***Knapp et al. 10267***C***Knapp et al. 10416*). Illustration by R. Wise.

##### Description.

Bushy small shrubs or herbs, 0.2–0.7 m high, to ca. 1 m spread, the branches more or less erect or spreading, slightly woody at the base. Stems strongly angled with wings to 1.5 mm wide and with abundant spinescent processes, glabrous to very sparsely pubescent with scattered white eglandular simple uniseriate 3–4-celled trichomes to 0.5 mm long, soon glabrescent; new growth moderately to densely pubescent with white eglandular simple uniseriate trichomes to 0.5 mm long; bark of older stems greenish brown, glabrescent. Sympodial units difoliate, the leaves usually not geminate. Leaves simple, usually more or less regularly toothed, the blades 2.1–10.5 cm long, 0.8–5.5 cm wide, ovate to broadly elliptic, occasionally narrowly elliptic, much larger on older stems, widest at the middle or just below, membranous to slightly rubbery, concolorous; adaxial and abaxial surfaces glabrous and shiny, with a few scattered white eglandular 3–4-celled simple uniseriate trichomes to 0.5 mm long like those of the stems; principal veins 7–9 pairs, usually slightly more pubescent that the lamina; base attenuate onto the petiole and the stem; margins usually strongly toothed, only occasionally entire or with few teeth near the base, the teeth 0.3–1.5 mm long, 0.4–1 mm wide, triangular with acute apices, the sinuses rounded, reaching ca. 1/8 to 1/5 of the way to the midrib; apex acute to acuminate; petioles winged from the decurrent leaf bases and then onto the stem, winged portion 0.5–3 cm long, glabrous. Inflorescences internodal, several times branched (occasionally only forked), 1–6 cm long, with 10–20 flowers at the tips of the branches or in the distal half, moderately to sparsely pubescent with white eglandular simple uniseriate trichomes to 0.5 mm long, usually more pubescent that the stems; peduncle 1–2 cm long; pedicels 0.9–1 cm long, ca. 0.5 mm in diameter at the base, ca. 0.75 mm in diameter at the apex, spreading at anthesis, sparsely pubescent with simple uniseriate trichomes like the rest of the inflorescence, articulated at the base, leaving a slight swelling on the axis; pedicel scars irregularly spaced 1–2 mm apart in the distal half of each inflorescence branch. Buds globose, the corolla ca. halfway exserted from the calyx before anthesis. Flowers 5-merous, cosexual (hermaphroditic). Calyx tube 1–1.5 mm long, conical or strongly cup-shaped, the lobes ca 1 mm long, 1–1.2 mm wide, broadly deltate, the tips obtuse to acute, strongly recurved in bud, sparsely pubescent with white eglandular simple uniseriate trichomes like the rest of the inflorescence. Corolla 0.9–1.2 cm in diameter, violet-blue, often edged white, with a green eye, stellate, lobed ca. 2/3 of the way to the base, the lobes 4–5 mm long, 3–4 mm wide, deltate, spreading to slightly reflexed at anthesis, adaxially glabrous, abaxially densely puberulent with white simple uniseriate trichomes ca. 0.2 mm long, these denser at the tips and margins. Stamens equal; filament tube minute; free portion of the filaments 0.6–1 mm long, sparsely pubescent with tangled transparent simple uniseriate trichomes adaxially; anthers 2–2.5 mm long, 1.2–1.5 mm wide, plump and ellipsoid, yellow, poricidal at the tips the pores lengthening to slits with age. Ovary conical, glabrous; style 5–6 mm long, straight (even in bud), markedly long-exserted beyond the anther cone, densely pubescent in the lower third with transparent, tangled simple trichomes; stigma ball-shaped and capitate, bright green in live plants, the surface minutely papillate. Fruit a globose or occasionally slightly ellipsoid berry, 0.8–1 cm in diameter, dark green with white striped mottling when ripe, the pericarp thin, shiny, translucent when ripe, glabrous; fruiting pedicels 1–1.1 cm long, ca. 1 mm in diameter at the base, ca. 1.2 mm in diameter at the apex, somewhat woody, deflexed, usually persistent; fruiting calyx not markedly enlarged, the tube and lobes to ca. 2 mm long, spreading and not markedly appressed to the berry. Seeds 20–30 per berry, 2–2.5 mm long, 1.5–1.7 mm wide, flattened and teardrop shaped, reddish brown, the surfaces minutely pitted, the testal cells sinuate in outline. Stone cells absent. Chromosome number: reported as 2n = 24 (Edmonds 1972, 1977, voucher *Hawkes ‘B*’, not verified).

**Figure 117.**
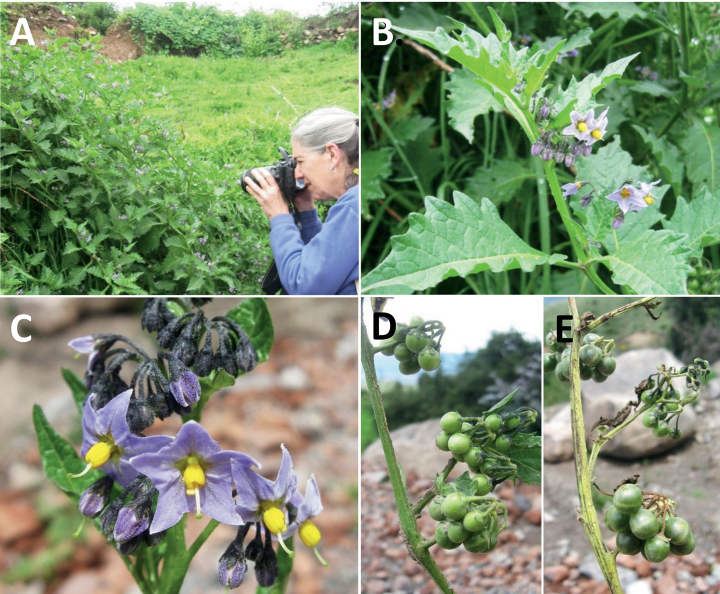
*Solanumpentlandii***A** habit **B** flowering branch **C** flowers at full anthesis **D** maturing fruits **E** fully mature fruits (**A, B***Knapp et al. 10308***C–E***Knapp et al. 10248*). Photos by S. Knapp.

##### Distribution

**Figure 118.**
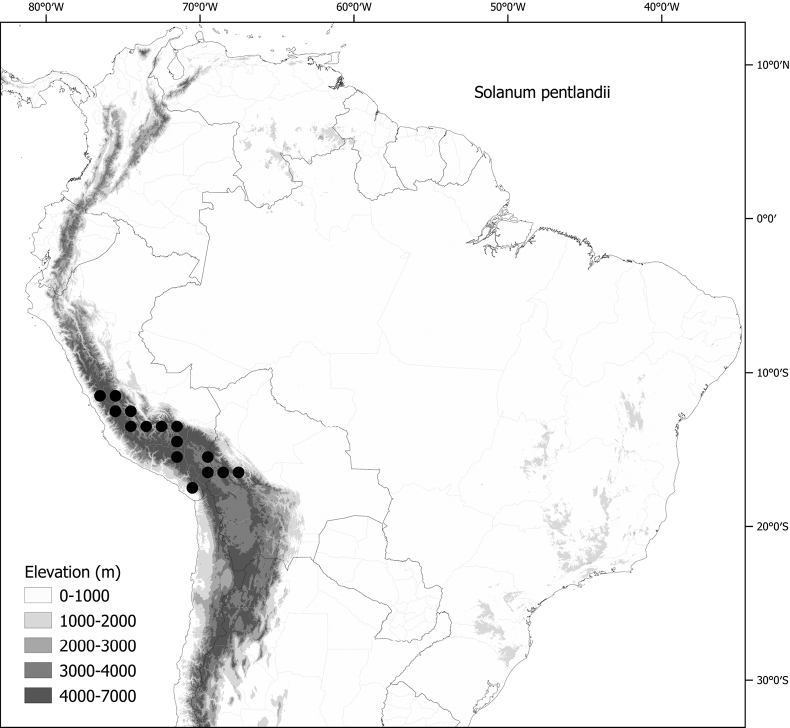
Distribution map of *Solanumpentlandii*.

**(Fig. 118).***Solanumpentlandii* occurs in the Andes from central Peru (Depts. Apurimac, Arequipa, Ayacucho, Cusco, Huancavelica, Junín, Lima, Puno, Tacna) to northern Bolivia (Dept. La Paz).

##### Ecology and habitat.

*Solanumpentlandii* occurs in open areas at high elevation, often in grassland and along roadsides; it appears to favour high nitrogen environments and is often collected near villages and cities, from 2,400 to 5,200 m elevation.

##### Common names and uses.

Peru. Cusco: chaja chaja (*Iltis & Iltis 867*), moyoccaya (*Cook & Gilbert 297*), qosmayllu (*Davis et al. 1348*). In the Quechua community of Chinchero (Cusco, Peru), the fruits are macerated and added to water to wash hair in the morning (Franquemont et al. 1990, as *S.arequipense*).

##### Preliminary conservation status

**(IUCN 2022).** Least Concern [LC]. EOO = 190,050 km^2^ [LC]; AOO = 228 km^2^ [EN]. *Solanumpentlandii* has a wide distribution, is a plant of disturbed areas and is common around the protected archaeological sites in the Sacred Valley near Cusco, Peru.

##### Discussion.

*Solanumpentlandii* is easy to confuse with *S.arequipense* and *S.furcatum*, with which it shares small flowers with short anthers, long-exserted styles and toothed leaf margins. It occurs at generally much higher elevations than either of those taxa, and is usually more glabrous, with shiny, often more deeply and regularly toothed, leaves. Flowers of *S.pentlandii* are usually dark violet, as compared to the normally white or pale violet flowers of *S.arequipense* and *S.furcatum*. The flowers of *S.pentlandii* are smaller than either of those two species (corolla 0.9 cm in diameter, anthers 2–2.5 mm long and style 5–6 mm long in *S.pentlandii*; more than 1.2 cm in diameter, anthers longer than 2.5 mm and style 6–9 mm long in *S.arequipense* and *S.furcatum*).

All of the names we here recognise as synonyms of *S.pentlandii* were coined by Bitter (1912a) using a number of collections made by Otto Buchtien in Bolivia; no herbaria other than “herb. Boliv.” were cited in any of the protologues. We have lectotypified all of these names with specimens from Buchtien’s Herbarium held in US. The lectotype we have selected for *S.coerulescens* is the more complete of the two sheets of *Buchtien 2965* held in US (00027517, acc. # 703363) and is annotated in an unknown hand “S.coerulescens Bitt. n. sp.”. Solanumpentlandiivar.manophyes and var. pycnophyes are lectotypified with sheets annotated as such by Buchtien (var. pycnophyes – *Buchtien 2966*, US barcode 00027519, acc. # 1175974; var. manophyes – *Buchtien 769* – US barcode 00027518, acc. # 1175829). Another specimen at US of a completely different plant numbered *Buchtien 769* (US barcode 00342204, acc. # 1498779) is a specimen of *Sennatrachypus* (Benth.) HS.Irwin & Barnaby (Leguminosae; Fabaceae); Buchtien appears to have used number series multiple times on different collecting trips. *Solanuminsulae-solis* was based on *Buchtien s.n.* collected on the Isla del Sol in Lago Titicaca (Bolivia); we select as the lectotype here the sheet of *Buchtien 5856* at US (barcode 00650475, acc. # 1175976) with the exact collecting locality and date cited by Bitter and annotated “S. insulae-solis Bitt. n. sp.” by Buchtien.

#### ﻿39. Solanum physalidicalyx

Taxon classificationPlantaeSolanalesSolanaceae

Bitter, Repert. Spec. Nov. Regni Veg. 11: 212. 1912.

Figs 119 , 120

Solanum physalidicalyx Bitter var. integrascens Bitter, Repert. Spec. Nov. Regni Veg. 11: 213. 1912. Type. Argentina. Salta: Pasaje del Rio Juramento, *P.G. Lorentz & G. Hieronymus s.n.* (no explicit type material located; likely homotypic with the species).
Solanum physalidicalyx Bitter var. plurilobulatum Bitter, Repert. Spec. Nov. Regni Veg. 11: 213. 1912. Type. Argentina. Salta: Pasaje del Rio Juramento, *P.G. Lorentz & G. Hieronymus s.n.* (no explicit type material located; likely homotypic with the species).

##### Type.

Argentina. Salta: Pasaje del Rio Juramento, Feb 1873, *P.G. Lorentz & G. Hieronymus 364* (lectotype, designated by Barboza et al. 2013, pg. 262: GOET [GOET003574]; isolectotypes: CORD [CORD00004269], DR [DR054234], US [00027741, acc. # 282274).

**Figure 119.**
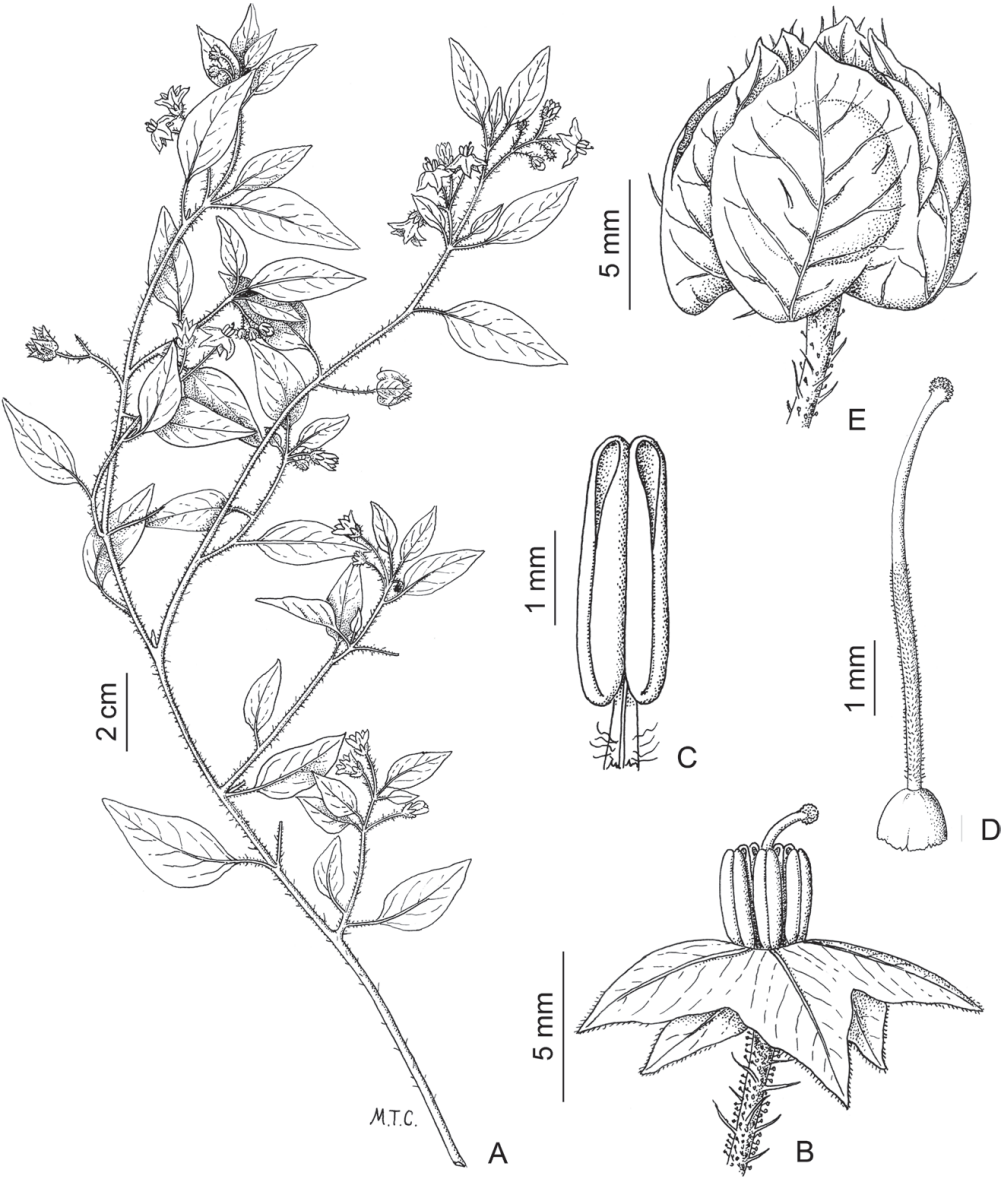
*Solanumphysalidicalyx***A** flowering and fruiting branch **B** flower **C** stamen, ventral view **D** gynoecium **E** fruit (**A–E***Cabrera 14409*). Illustration by M.T. Cabrera. Previously published in Barboza et al. (2013: 262), as * “S.tweedianum*”.

##### Description.

Annual (?) or perennial herbs, the branches 0.3–1.3 m long, spreading and sprawling when large, viscid to the touch, somewhat woody at the base. Stems terete, viscid, densely pubescent with transparent glandular simple 3–5-celled uniseriate trichomes of varying lengths to 2.5 mm long and shorter simple uniseriate glandular trichomes, the glands unicellular; new growth densely pubescent with glandular papillae and transparent glandular simple uniseriate trichomes to 5-celled and 2 mm long; bark of older stems pale yellow when dry, remaining viscid to the touch. Sympodial units difoliate, the leaves not geminate. Leaves simple or shallowly crenate, the blades (1.2)2.5–8(9) cm long, (0.7)1–4.5(7.5) cm wide, ovate to broadly elliptic, widest at the middle or in the lower half, membranous, concolorous, viscid to the touch, extremely variable in size within a plant; adaxial surfaces sparsely to moderately but evenly glandular-pubescent with transparent simple uniseriate trichomes ca. 1 mm long, these denser along the veins; abaxial surfaces sparsely and evenly pubescent with similar glandular simple uniseriate trichomes, or the trichomes only on the veins; principal veins 4–6 pairs, glandular-pubescent; base abruptly truncate; margins entire or irregularly crenate, the lobes 1–2 mm long; apex acute; petiole (0.5)1–3(5) cm long, densely glandular pubescent like the stems, the pubescence denser on adaxial surface. Inflorescences opposite the leaves or occasionally internodal (ca. 1 mm away from leaf), unbranched, 1–2.5(4) cm long, with 3–8(10) flowers in the distal half, densely glandular-pubescent with transparent simple uniseriate trichomes 1–1.5 mm long and shorter glandular papillae; peduncle 0.5–2 cm long; pedicels 0.8–1 cm long, ca. 0.4 mm in diameter at the base, ca. 0.5 mm in diameter at the apex, gradually tapering, spreading at anthesis, densely glandular-pubescent with transparent, simple uniseriate trichomes to 1 mm long, articulated at the base but leaving a small raised stump ca. 0.3 mm long; pedicel scars evenly spaced ca. 1.5 mm apart, more crowded distally. Buds ellipsoid, the corolla just exserted from the calyx lobe tips before anthesis. Flowers 5-merous, cosexual (hermaphroditic). Calyx tube 1.5–2 mm long, conical, the lobes 1.5–3 mm long, deltate to triangular, densely glandular-pubescent with transparent simple uniseriate trichomes to 1.5 mm long. Corolla 1.2–1.4 cm in diameter, white with a pale yellowish green central star, this sometimes edged with purple, stellate, lobed ca. halfway to the base, the lobes 4–5 mm long, 3–5 mm wide, deltate, spreading or slightly reflexed at anthesis, glabrous adaxially, sparsely glandular-pubescent over the entire surface abaxially, the trichomes denser at the tips. Stamens equal; filament tube minute; free portion of the filaments 0.25–0.5 mm long, pubescent with transparent, tangled eglandular simple uniseriate trichomes adaxially; anthers (2.6)3–3.5(4) mm long, 1–1.2 mm wide, ellipsoid, yellow, poricidal at the tips, the pores lengthening to slits with age. Ovary conical, glabrous or with a few glandular papillae apically; style 6–7 mm long, curved upwards distally, exserted beyond the anther cone, densely papillate in the lower third inside the anther cone; stigma globose, the surface minutely papillate, green in live plants. Fruit a globose berry, 0.6–0.8 cm in diameter, green or marbled green (*Hunziker 10997*) at maturity, completely enclosed in the accrescent, inflated calyx, the pericarp thin, shiny, translucent, glabrous; fruiting pedicels ca. 1.5 cm long, ca. 0.5 mm in diameter at the base, ca. 1.5 mm in diameter at the apex, strongly deflexed with a distinct bend at the base, not markedly woody, not persistent; fruiting calyx accrescent and inflated, loosely and completely covering the berry, the base invaginate, the tube to 1.5 cm long, the lobes ca. 5 mm long, ca. 3 mm wide, pointed at the tips, the tube expanding more than the lobes, remaining viscid pubescent. Seeds 10–25 per berry, 2–2.5 mm long, 1.5–2 mm wide, flattened and teardrop shaped, reddish brown, the surfaces minutely pitted, the testal cells sinuate in outline with elongate “hairy” lateral walls to 0.25 mm long at maturity. Stone cells absent or 2 apical, ca. 0.5 mm in diameter, creamy white. Chromosome number: not known.

**Figure 120.**
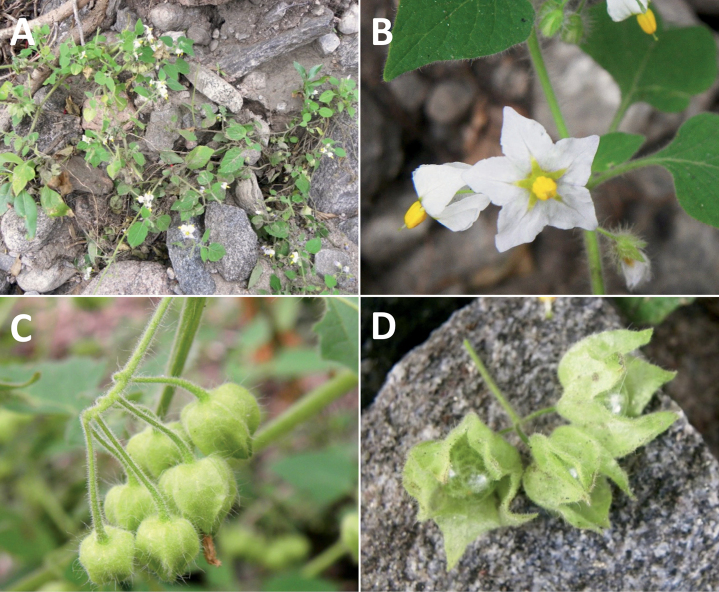
*Solanumphysalidicalyx***A** habit **B** flowering branch **C** developing infructescence **D** maturing fruits (**A–D***Barboza 4210*). Photos by G.E. Barboza. Previously published in part in Knapp et al. (2020: 39).

##### Distribution

**Figure 121.**
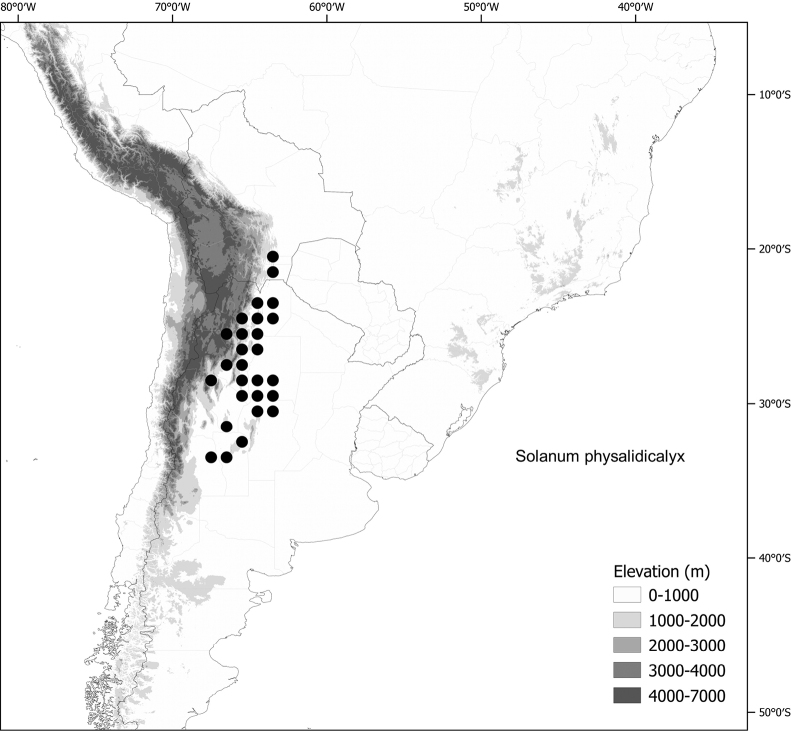
Distribution map of *Solanumphysalidicalyx*.

**(Fig. 121).***Solanumphysalidicalyx* occurs from southern Bolivia (Depts. Santa Cruz, Tarija) to central Argentina (Provs. Catamarca, Córdoba, Jujuy, La Rioja, Tucumán, Salta, San Luis, Santiago del Estero).

##### Ecology and habitat.

*Solanumphysalidicalyx* grows in dry forests and Chaco woodlands, often in the shade of trees or hedges, from 300 to 2,500 m elevation (two collections from Salta have elevations of 2,700 to 3,100 m).

##### Common names and uses.

None recorded.

##### Preliminary conservation status

**(IUCN 2022).** Least Concern [LC]. EOO = 605,225 km^2^ [LC]; AOO = 312 km^2^ [EN]. *Solanumphysalidicalyx* is not as common as the very similar *S.tweedieanum*, but it has a similarly wide distribution and is a plant of disturbed areas. It appears not to have been collected within protected areas in Argentina.

##### Discussion.

*Solanumphysalidicalyx* had long been recognised as a synonym of *S.tweedieanum* (Edmonds 1972; Barboza et al. 2013) a morphologically similar glandular-pubescent species, but Knapp et al. (2020) recognised it as distinct and unravelled the complex set of names surrounding these two species. *Solanumphysalidicalyx* differs from *S.tweedieanum* in its conspicuously inflated calyx in fruit (as opposed to merely accrescent and tightly investing the berry) and in its anthers 3–3.5 mm long (versus 4–5 mm in *S.tweedieanum*, although some overlap can occur). The two species are partly sympatric in Argentina and in the absence of mature fruit, can be very difficult to distinguish, although in general plants of *S.physalidicalyx* are more herbaceous and delicate (usually annuals?) than those of *S.tweedieanum*, which are woody and rhizomatous.

In *Flora Argentina*Barboza et al. (2013) recognised two species, *S.tweedieanum* (as “*S.tweedieanum*”, a misspelling) and *S.atriplicifolium*, both glandular-pubescent with ovate, shallowly toothed leaves. *Solanumphysalidicalyx* was erroneously put into synonymy with *S.tweedieanum*; the type of *S.tweedieanum* is a better match for those plants they called *S.atriplicifolium*, which is here treated as a synonym of *S.tweedianum* The type of *S.tweedieanum* comes from a plant cultivated at Kew that was collected in flower only; it lacks the diagnostic calyx characters that enable easy identification in this group, but anther length can also be used to distinguish those plants not in fruit. See Knapp et al. (2020) for a complete discussion and comparison of these glandular-pubescent plants.

The chromosome count of 2n = 24 reported by Edmonds (1972) is based on a voucher (*Hawkes et al. 3204*) we have been unable to locate. From the locality (between Mina Clavero and Villa Dolores in Córdoba, Argentina) this could represent either *S.tweedieanum* or *S.physalidicalyx*.

Details of typification of *S.physalidicalyx* and the issues with its synonyms are treated in Knapp et al. (2020).

#### ﻿40. Solanum physalifolium

Taxon classificationPlantaeSolanalesSolanaceae

Rusby, Mem. Torrey Bot. Club 6: 88. 1896.

Figs 4C , 122 , 123

Solanum nitidibaccatum Bitter var. robusticalyx Bitter, Repert. Spec. Nov. Regni Veg. 11: 209. 1912. Type. Bolivia. Cochabamba: Parotani, [2,400 m], 20 Mar 1892. *C.E.O. Kuntze s.n.* (lectotype, designated by Edmonds 1986, pg. 25: NY [00172105]; isolectotype: US [00027706, acc. # 702148]).

##### Type.

Bolivia. Cochabamba: vic. Cochabamba, 1891, *M. Bang 1159* (lectotype, designated by Edmonds 1986, pg. 25, second step designated here: NY [00172129]; isolectotypes: GH [00077734], K [K001390419], MO [MO-503737, acc. # 5579576], NDG [NDG45199], NY [00172128], PH [00030469], US [00027742, acc. # 1324703; 00650472, acc. # 3412820], WIS [v0256203WIS]).

**Figure 122.**
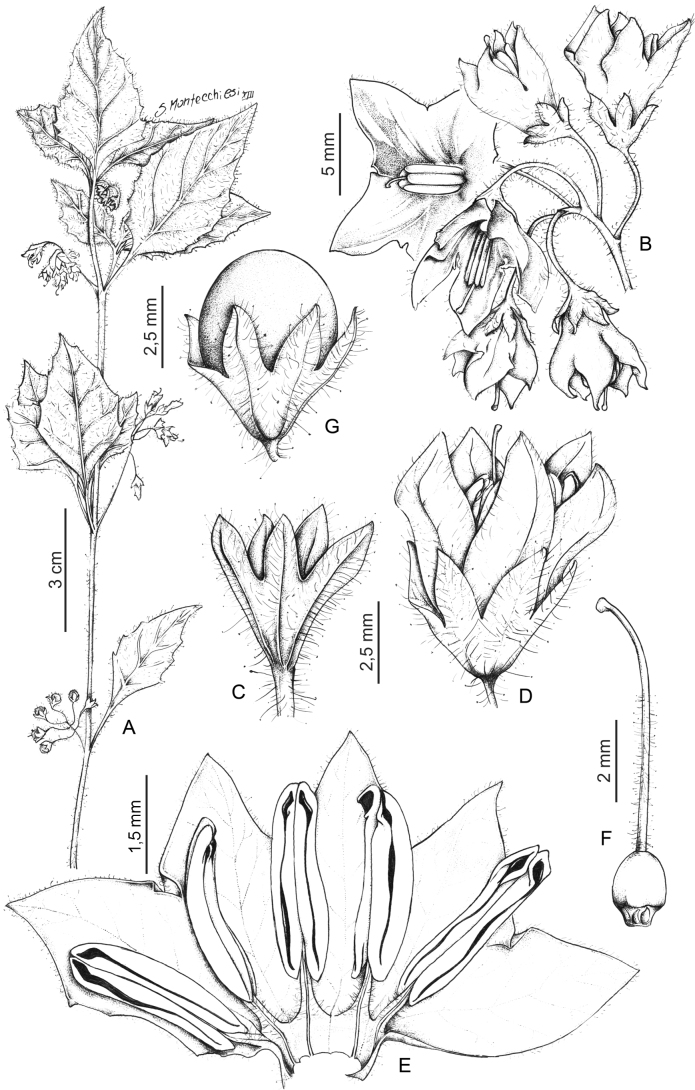
*Solanumphysalifolium***A** flowering branch **B** inflorescence **C** calyx **D** flower **E** dissected flower **F** gynoecium **G** fruit (**A–G***Barboza et al. 2229*). Illustration by S. Montecchiesi. Previously published in Barboza et al. (2013: 253).

##### Description.

Annual herbs to 0.5 m high, spreading to 1 m in diameter, from a strong taproot and occasionally somewhat woody at the base. Stems terete or occasionally winged from decurrent leaf bases, the wings if present to 1.5 mm wide, densely glandular-pubescent with transparent 6–10-celled simple uniseriate trichomes to 2 mm long, but most shorter than 2 mm, later glabrescent; new growth densely glandular-pubescent with transparent simple uniseriate trichomes like the young stems, the longest trichomes ca. 2 mm long; bark of older stems pale brown, glabrescent. Sympodial units difoliate or trifoliate, the leaves not usually geminate. Leaves simple, entire or shallowly toothed, the blades (1.4)2–6 cm long, (0.8)1.4–3.2 cm wide, ovate to elliptic-ovate, widest in the lower third, membranous, concolorous; adaxial surface sparsely to moderately and evenly glandular-pubescent with transparent 6–10-celled simple uniseriate trichomes 1.5–2 mm long; abaxial surfaces glandular-pubescent like the upper surfaces, but the trichomes denser along the veins; principal veins 3–5 pairs, drying somewhat yellowish green; base abruptly truncate then attenuate onto the petiole; margins entire to shallowly toothed, the teeth ca. 1.5 mm long, deltate with acute tips; apex acute; petiole 0.3–1.5 cm long, winged from the decurrent leaf bases. Inflorescences internodal or occasionally opposite the leaves, unbranched, 0.8–2.1 cm long, with 3–6 flowers clustered at the tips and the inflorescence more or less subumbellate, densely glandular-pubescent like the stems; peduncle 0.7–1.7 cm long; pedicels 0.6–0.8 cm long, ca. 0.25 mm in diameter at the base, ca. 0.5 mm in diameter at the apex, filiform, spreading at anthesis, glandular-pubescent like the rest of the inflorescence, articulated at the base; pedicel scars 0.5–3 mm apart, the lowermost flower more distant from the rest. Buds ellipsoid, the corolla just exserted from the calyx lobe tips before anthesis. Flowers 5-merous, cosexual (hermaphroditic). Calyx tube 1–1.2 mm long, conical, the lobes 1.5–2 mm long, 1–1.5 mm wide, triangular with slightly rounded tips, densely glandular-pubescent with simple uniseriate trichomes like those of the stems. Corolla 0.8–1 cm in diameter, white with a green central eye, stellate, lobed ca. 1/2 way to the base, the lobes 3–3.5 mm wide, 2.5–3 mm wide, broadly triangular to deltate, spreading to slightly reflexed at anthesis, adaxially glabrous, abaxially densely papillate at the tips and margins and with long transparent trichomes at tips and along the midvein, these a mixture of glandular and eglandular. Stamens equal; filament tube ca. 0.3 mm long; free portion of the filaments 1–1.2 mm long, adaxially pubescent with tangled eglandular simple uniseriate trichomes; anthers 2–3 mm long, 1–1.1 mm wide, ellipsoid, yellow, poricidal at the tips, the pores lengthening to slits with age. Ovary conical, glabrous; style 3.5–5 mm long, strongly hooked in the distal part, exserted beyond the anther cone, densely papillate in the lower half to 3/4 where enclosed in the anther cone; stigma capitate, the surface minutely papillate. Fruit a globose berry, 0.8–1 cm in diameter, green and strongly marbled with white when mature, the pericarp thin, shiny and translucent, glabrous; fruiting pedicels 0.8–1.2 cm long, ca. 0.75 mm in diameter at the base, not markedly woody, strongly deflexed at the base, not persistent; fruiting calyx accrescent and spreading, not enclosing or appressed to the berry, the tube 3.5–5.5 mm long, the lobes ca. 5.5 mm wide, 4.5–5.5 mm wide, the venation prominent in dry specimens. Seeds 30–40 per berry, ca. 2 mm long, ca. 1.5 mm wide, flattened and teardrop shaped, reddish brown, the surfaces minutely pitted, the testal cells rectangular in shape. Stone cells absent or present and 2 at the apex of the berry or 6 with 2 apical and 4 equatorially positioned, ca. 0.5 mm in diameter, creamy white. Chromosome number: not known.

**Figure 123.**
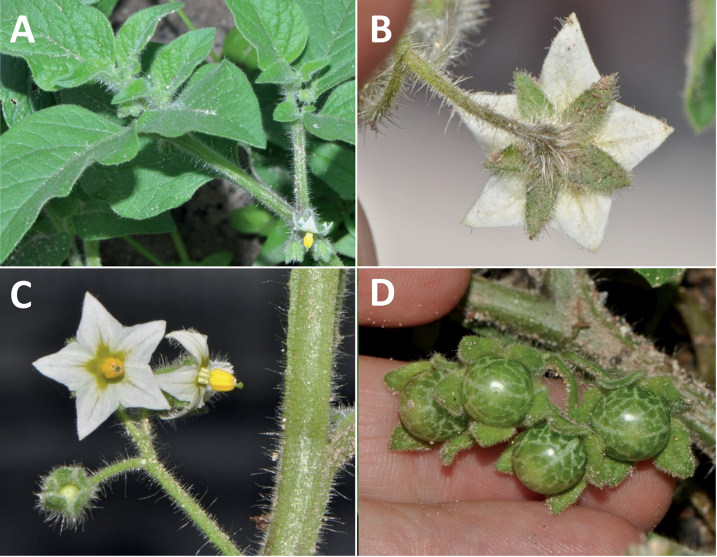
*Solanumphysalifolium***A** habit **B** abaxial surface of flower **C** flowers and buds **D** developing fruits (**A–D***Knapp et al. 10332*). Photos by S. Knapp.

##### Distribution

**Figure 124.**
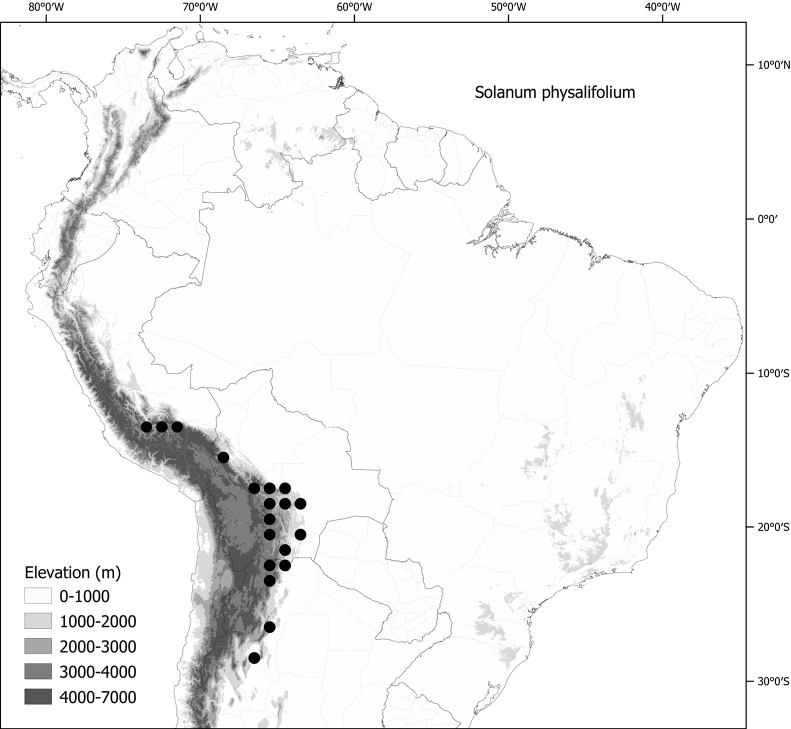
Distribution map of *Solanumphysalifolium*.

**(Fig. 124).***Solanumphysalifolium* is an Andean species, occurring from southern Peru (Depts. Apurímac, Cusco) through Bolivia (Depts. Chuquisaca, Cochabamba, La Paz, Potosí, Santa Cruz, Tarija) to northern Argentina (Provs. Catamarca, Jujuy, Salta).

##### Ecology and habitat.

*Solanumphysalifolium* grows in dry interAndean valleys, often along streams or in the shade of small trees, from 1,500 to 3,500 m elevation.

##### Common names and uses.

None recorded.

##### Preliminary conservation status

**(IUCN 2022).** Least Concern [LC]. EOO = 757,522 km^2^ [LC]; AOO = 172 km^2^ [EN]. *Solanumphysalifolium* is a plant of open disturbed areas and has a wide distribution along the Andes. It occurs in protected areas in Argentina (e.g., Parque Nacional Baritú) and Peru (around the ruins of Sacsayhuamán near Cusco).

##### Discussion.

*Solanumphysalifolium* is one of several glandular-pubescent morelloids from the southern Andes. Morphologically it is similar to both *S.physalidicalyx* and *S.profusum*; the three species all have anthers 2–3 mm long and long, sticky glandular trichomes. *Solanumphysalifolium* differs from *S.physalidicalyx* in mature fruits; the calyx is inflated and completely covering the whitish green to cream berry in *S.physalidicalyx* and only partially covering the dark green marbled berry in *S.physalifolium*. *Solanumphysalifolium* is an annual, while *S.profusum* is a rhizomatous perennial. Leaf shape also differs; *S.profusum* has more lanceolate to lance-elliptic leaves, while those of *S.physalifolium* and *S.physalidicalyx* are ovate to elliptic ovate. The distributions of the three species do not overlap. A key to the glandular-pubescent morelloids can be found in Knapp et al. (2020).

In lectotypifying *S.physalifolium*Edmonds (1986) only cited “NY” and did not specify which sheet of Bang *1159* she was referring to as the lectotype; we here select the better preserved of the two sheets held at NY (00172129) in a second step lectotypification.

#### ﻿41. Solanum pilcomayense

Taxon classificationPlantaeSolanalesSolanaceae

Morong, Ann. New York Acad. Sci. 7: 177. 1893.

Figs 125 , 126

Solanum nigrum L. var. pilcomayense (Morong) Chodat, Bull. Herb. Boissier, sér. 2, 2: 747. 1902. Type. Based on Solanumpilcomayense Morong.
Solanum pilcomayense Morong var. brevipetiolare Chodat, Bull. Herb. Boissier, sér. 2, 2: 811. 1902. Type. Paraguay. “in insula a Caprera [?]” [probably Isla Cabrera in Dpto. Ñeembucu], May 1885–1895, *É. Hassler 2524* (lectotype, designated by Morton 1976, pg. 141 [as holotype]: G [G00306739, Morton neg. 8661]).
Solanum nigrum L. forma brevipetiolare (Chodat) Chodat, Bull. Herb. Boissier, sér. 2, 4: 80. 1903. Type. Based on SolanumpilcomayenseMorongvar.brevipetiolare Chodat.
Solanum nigrum L. subsp. chacoense Hassl., Trab. Mus. Farmacol. 21: 104. 1909. Type. Paraguay. “Gran Chaco, ad ripam occidentalem flum.”, *T. Rojas in É. Hassler 2324* (lectotype, designated by Morton 1976, pg. 141 [as holotype]: G [G00306755, Morton neg. 8660]; isolectotypes: BM [BM000087562], G [G00306749, G00306750, G00306751, G00306753], GH [00105872], K [K000585693], W [acc. # 1906-0001034]).
Solanum nigrum L. forma floribundum Hassl., Trab. Mus. Farmacol. 21: 104. 1909, as “SolanumnigrumL.subsp.chacoenseHassl.var.genuinumHassl.formafloribundum Hassl.” Type. Paraguay/Argentina. [Presidente Hayes/Formosa]: “ad ripam fluminis, in regione cursus inferioris fluminis Pilcomayo [orillas de los ríos – ex protologue]”, Jul 1906, *T. Rojas 108d* (lectotype, designated by Morton 1976, pg. 141 [as type]: G [G00306748, Morton neg. 8662]; isolectotype: G [G00306753]).
Solanum nigrum L. var. subhastatum Hassl., Trab. Mus. Farmacol. 21: 104. 1909, as “SolanumnigrumL.subsp.chacoenseHassl.var.subhastatum Hassl.” Type. Paraguay/Argentina. [Presidente Hayes/Formosa]: “ad ripam fluminis, in regione cursus inferioris fluminis Pilcomayo [en los campos humedos – ex protologue]”, May 1906, *T. Rojas 108a* (lectotype, designated by Morton 1976, pg. 141 [as holotype]: G [G00306747]).
Solanum nigrum L. forma longepedunculatum Hassl., Trab. Mus. Farmacol. 21: 105. 1909, as “SolanumnigrumL.subsp.chacoenseHassl.var.genuinumHassl.formalongepedunculatum Hassl.” Type. Paraguay/Argentina. [Presidente Hayes/Formosa]: “ad ripam fluminis, in regione cursus inferioris fluminis Pilcomayo [orillas del río – ex protologue]”, May 1906, *T. Rojas 108* (lectotype, designated by Morton 1976, pg. 141 [as holotype]: G [G00306746]).
Solanum nigrum L. forma longepetiolatum Hassl., Trab. Mus. Farmacol. 21: 105. 1909, as “*langepetiolatum*”, as “SolanumnigrumL.subsp.chacoenseHassl.var.genuinumHassl.formalangepetiolatum Hassl.” Type. Paraguay/Argentina. [Presidente Hayes/Formosa]: “ad ripam fluminis, in regione cursus inferioris fluminis Pilcomayo [arenales en las orillas del río – ex protologue]”, May 1906, *T. Rojas 108c* (lectotype, designated Morton 1976, pg. 141 [as holotype]: G [G00306742, G00306743; two barcodes on same sheet]).
Solanum nigrum L. subforma sinuatodentatum Hassl., Trab. Mus. Farmacol. 21: 105. 1909, as “SolanumnigrumL.subsp.chacoenseHassl.var.subhastatumHassl.formalangepetiolatumHassl.subformasinuatodentatum Hassl.” Type. Paraguay/Argentina. [Presidente Hayes/Formosa]: “ad ripam fluminis, in regione cursus inferioris fluminis Pilcomayo [arenales en las orillas del río – ex protologue]”, May 1906, *T. Rojas 108b* (lectotype, designated by Morton 1976, pg. 141 [as holotype], second step designated here: G [G00306745, G00306744; two barcodes on same sheet]; isolectotype: G [G00306740, G00306741, two barcodes on the same sheet]).
Solanum nigrum L. var. brevipetiolare (Chodat) Chodat & Hassl., Trab. Mus. Farmacol. 21: 105. 1909, as “SolanumnigrumL.subsp.chacoenseHassl.var.brevipetiolare Chodat & Hassl.” Type. Based on SolanumpilcomayenseMorongvar.brevipetiolare Chodat.
Solanum nigrum L. forma pilcomayense (Morong) Hassl., Trab. Mus. Farmacol. 21: 105. 1909, as “SolanumnigrumL.subsp.chacoenseHassl.var.brevipetiolareHassl.formapilcomayense (Morong) Hassl.” Type. Based on Solanumpilcomayense Morong.
Solanum nigrum L. forma brevipetiolare (Chodat) Hassl., Trab. Mus. Farmacol. 21: 105. 1909, as “SolanumnigrumL.subsp.chacoenseHassl.var.brevipetiolareChodat & Hassl.formabrevipetiolare Hassl.”, nom. illeg. superfl. non Solanumnigrumformabrevipetiolare (Chodat) Chodat (1903). Type. Based on SolanumpilcomayenseMorongvar.brevipetiolare Chodat.
Solanum pulchrilobum Bitter, Repert. Spec. Nov. Regni Veg. 11: 4. 1912. Type. Paraguay/Argentina. [Presidente Hayes/Formosa]: “ad ripam fluminis, in regione cursus inferioris fluminis Pilcomayo [arenales en las orillas del río – ex protologue]”, May 1906, *T. Rojas 108b* (syntypes: B, destroyed [F neg. 2755]: lectotype, designated by Morton 1976, pg. 141, second step designated here: G [G00306745, G00306744; two barcodes on same sheet]; isolectotype:G [G00306740, G00306741, two barcodes on the same sheet]).
Solanum pulchrilobum Bitter var. paucilobum Bitter, Repert. Spec. Nov. Regni Veg. 11: 5. 1912. Type. Paraguay/Argentina. [Presidente Hayes/Formosa]: “ad ripam fluminis, in regione cursus inferioris fluminis Pilcomayo”, May 1906, *T. Rojas 108c* (holotype: B, destroyed; lectotype, designated by Morton 1976, pg. 141 [as isotype]: G [G00306742, G00306743; two barcodes on same sheet]).
Solanum basilobum Bitter, Repert. Spec. Nov. Regni Veg. 11: 215. 1912. Type. Argentina. Chaco: Barranqueras, 27 Aug 1892, *G. Niederlein 284* (holotype: B, destroyed [F neg. 2864]; lectotype, designated by Barboza et al. 2103, pg. 253: PH [00030388]).
Solanum syringoideum Bitter, Repert. Spec. Nov. Regni Veg. 11: 225. 1912. Type. Paraguay. “Gran Chaco, ad ripam occidentalem flum.”, *T. Rojas in É. Hassler 2324* (holotype: B, destroyed [F neg. 2758]; lectotype, designated here: BM [BM000087562]; isolectotypes: G [G00306749, G00306750, G00306751, G00306753, G00306755], GH [00105872], K [K000585693], W [acc. # 1906-0001034]).
Solanum syringoideum Bitter var. pycnostichanthum Bitter, Repert. Spec. Nov. Regni Veg. 11: 225. 1912. Type. Paraguay. “Gran Chaco, ad ripam occidentalem flum.” 1903, *T. Rojas in É. Hassler 2393* (holotype: B, destroyed [F neg. 2758]; lectotype, designated by Morton 1976, pg. 141: G [G00306737]; isolectotypes: BM [BM000087587], K [K000585694]).
Solanum pulchrilobum Bitter var. longepetiolatum (Hassl.) Parodi, Tomo Conmem. 25 Aniv. Fund. Fac. Agron. Vet. Buenos Aires 85. 1929. Type. Based on SolanumnigrumL.var.longepetiolatum Hassl.
Solanum deltaicum Cabrera, Fl. Prov. Buenos Aires 5a: 215. 1965. Type. Argentina. Buenos Aires: Delta, Paraná Miní, 18 May 1950, *A.L. Cabrera 10626* (holotype: LP [LP005356]).
Solanum pilcomayense Morong var. vicinum C.V.Morton, Revis. Argentine Sp. Solanum 143. 1976. Type. Argentina. Tucumán: Dpto. Leales: Chañar Pozo, 5 Nov 1919, *S. Venturi 624* (holotype: US [00027744, acc. # 1548361]; isotypes: A [00077736], SI [075135, acc. # 167305, 137336, acc. # 167305b]).

##### Type.

Paraguay. Pilcomayo River, 1888–1890, *T. Morong 898* (lectotype, designated here: NY [00172130]; isotypes: BM [BM000087584], E [E00106293], GH [00077735], MICH [1109928], MO [MO-503704, acc. # 3575651], PH [00030470], US [00027743, acc. # 48030; 00650476, acc. # 1324704], WIS [v0256204WIS]).

**Figure 125.**
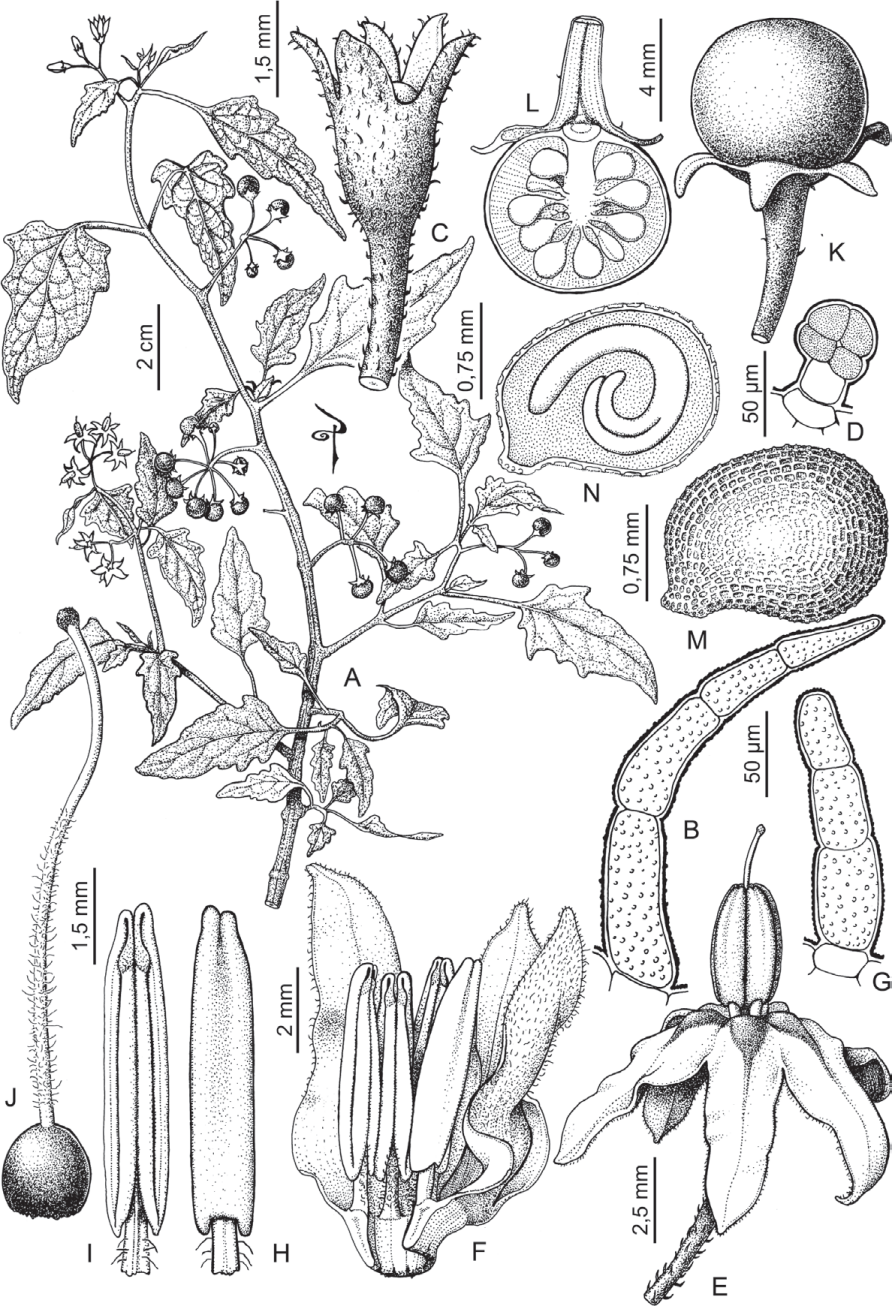
*Solanumpilcomayense***A** flowering and fruiting habit **B** eglandular leaf trichome **C** calyx **D** glandular trichome of the calyx **E** flower **F** dissected flower **G** eglandular trichome of the corolla **H** stamen, dorsal view **I** stamen, ventral view **J** gynoeciu **K** frui **L** fruit, longitudinal section **M** seed **N** seed, longitudinal section (**A–N***Barboza et al. 2287*). Illustration by P. Peralta. Previously published in Barboza et al. (2013: 254).

##### Description.

Perennial herbs or subshrubs to 1.5 m, woody at the base, the branches sprawling on other vegetation. Stems terete, distally thin and sprawling, minutely puberulent with eglandular, translucent simple uniseriate 2–5-celled trichomes 0.5–1 mm long, these usually antrorse; new growth densely to moderately pubescent with translucent simple uniseriate 2–5-celled trichomes 0.5–1 mm long; bark of older stems pale yellowish tan. Sympodial units difoliate, the leaves usually not, but occasionally, geminate. Leaves simple, entire or shallowly toothed, the blades 2–8(11) cm long, 1.5–5(7) cm wide, obovoid to oblanceolate, distinctly triangular in outline, widest in the lower quarter, membranous, concolorous; adaxial and abaxial surfaces sparsely and evenly pubescent with translucent simple uniseriate trichomes 0.5–1 mm long, these denser along the veins; principal veins 5–6 pairs, drying paler than the lamina; apex acute to acuminate; margins entire or shallowly toothed in the lower third, if present the sinuses ca. 1/4 of the way to the midrib (e.g., *Rojas 108b*, F neg. 2755, type of *S.pulchrilobum*), the teeth with acute to slightly rounded tips; base abruptly truncate; petiole 1–3(-4) cm long, sparsely pubescent like the stems. Inflorescences internodal or occasionally opposite the leaves, usually unbranched but occasionally forked (e.g., *Schinini et al. 10021*), 1.5–4 cm long, with 5–10(15) flowers, sparsely and evenly pubescent with simple uniseriate mostly antrorse trichomes ca. 0.5 mm long like the stems; peduncle 1.4–2.5(3) cm long; pedicels 0.8–1.3 cm long, ca. 0.5 mm in diameter at the base, ca. 1 mm in diameter at the apex, spreading at anthesis, articulated at the base with a somewhat swollen insertion point; pedicel scars closely spaced ca. 0.5 mm apart, mostly clustered at the tips of the inflorescence. Buds ellipsoid, the corolla strongly exserted from the calyx tube before anthesis. Flowers 5-merous, cosexual (hermaphroditic). Calyx tube ca. 1.5 mm long, conical, the lobes 1–2.5 mm long, long-triangular, often unequal in size, the sinuses rounded, the tips blunt, sparsely pubescent with simple uniseriate trichomes like the rest of the inflorescence. Corolla 1.2–1.8 cm in diameter, white with a green central eye, deeply stellate, lobed 3/4 of the way to the base, the lobes 4–5.5 mm long, 2–2.5 mm wide, reflexed then spreading at anthesis, adaxially glabrous, abaxially minutely puberulent with unicellular papillae, these denser on the tips and margins. Stamens equal; filament tube minute; free portion of the filaments ca. 0.5 mm long, densely pubescent adaxially with tangled, translucent simple uniseriate trichomes; anthers 3–4 mm long, 0.75–1.1 mm wide, ellipsoid, yellow, the abaxial surfaces sometimes somewhat papillate, poricidal at the tips, the pores lengthening to slits with age. Ovary conical, glabrous; style 5–6 mm long, straight, exserted beyond the anther cone, densely pubescent in the lower third inside the anther cone; stigma minutely capitate, the surface minutely papillose. Fruit a globose berry, 0.5–0.7 cm in diameter, green becoming black or dark purple when ripe (some collections from Paraguay mention “red” berries, e.g., *Zardini &Tilleria 35278*), the pericarp thin, matte to somewhat shiny, opaque, glabrous; fruiting pedicels 1.9–2.5 cm long, ca. 0.5 mm in diameter at the base, ca. 1.5 mm in diameter at the apex, spreading, somewhat woody, not persistent; fruiting calyx not accrescent, the lobes not enlarged. Seeds 40–60 per berry, ca. 1.5 mm long, ca. 1 mm wide, flattened and teardrop shaped, pale tan, the surfaces minutely pitted, the testal cells pentagonal to somewhat sinuate in outline. Stone cells 2–4 per berry, ca. 0.5 mm in diameter, usually found close together in the berry, cream-coloured. Chromosome number: n = 12 (Moscone 1992, voucher *Di Fulvio 806*; Moyetta et al. 2013, vouchers *Barboza et al. 2279, 2287*).

**Figure 126.**
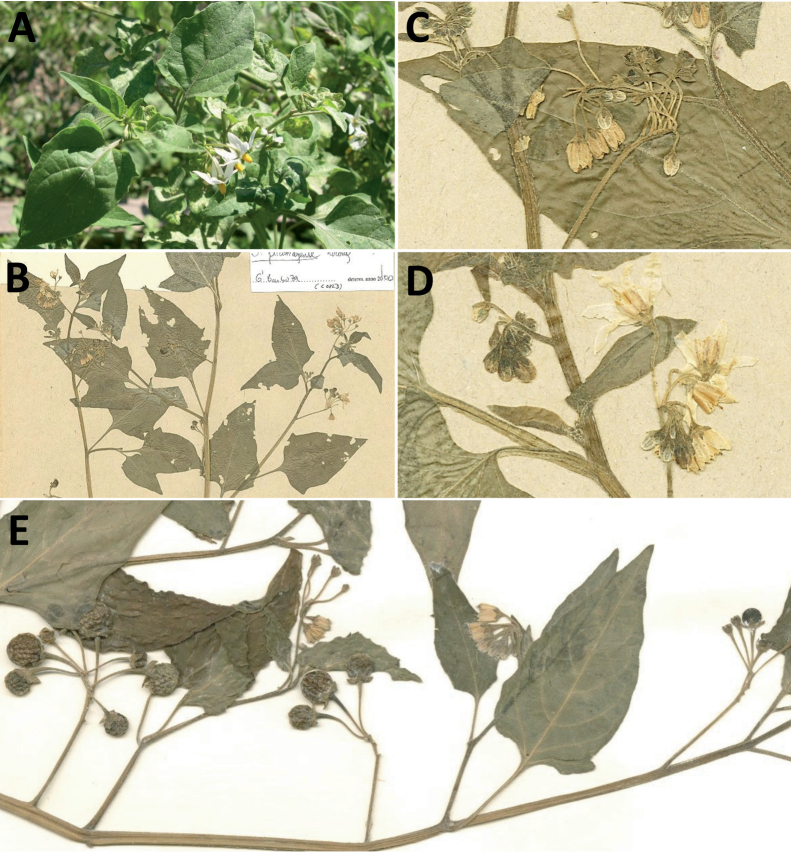
*Solanumpilcomayense***A** habit (live plant) **B** habit (herbarium sheet) **C** inflorescences with buds **D** flowers at full anthesis **E** fruiting branch with maturing fruits (**A***Peña-Chocarro et al. 1496***B–D***Rojas 108* [G00306746] **E***Morong 898* [PH 00030470]). Reproduced with permission of the Conservatoire et Jardins Botaniques de la Ville de Genéve and the Philadelphia Academy of Sciences (Drexel University).

##### Distribution

**Figure 127.**
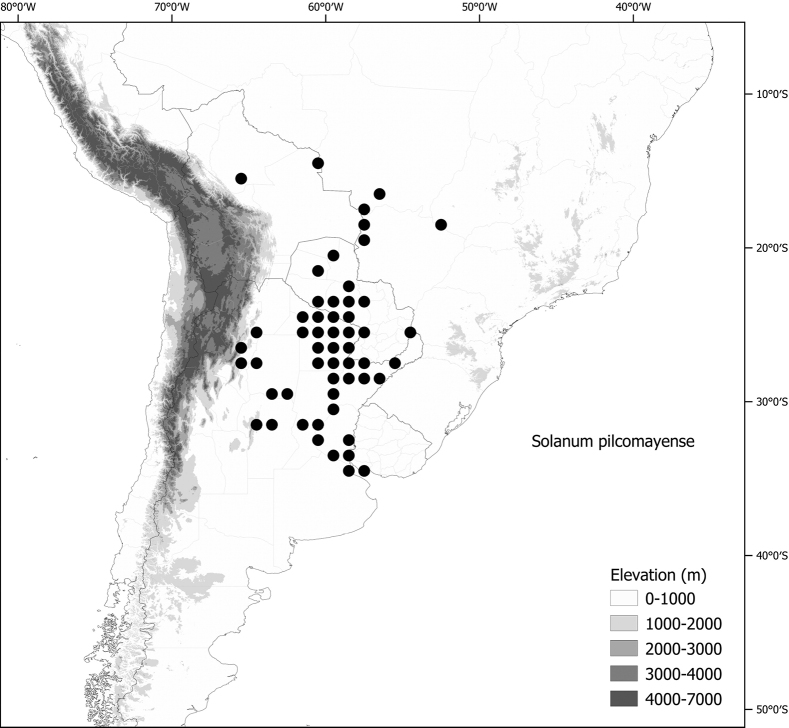
Distribution map of *Solanumpilcomayense*.

**(Fig. 127).***Solanumpilcomayense* occurs primarily in the Paraná River Basin, in Brazil (States of Mato Grosso, Mato Grosso do Sul, Pará, Paraná, Rio Grande do Sul), Bolivia (Depts. Bení, Santa Cruz), Paraguay (Depts. Alto Paraguay, Boquerón, Central, Concepción, Cordillera, Misiones, Presidente Hayes) and Argentina (Provs. Buenos Aires, Chaco, Corrientes, Entre Ríos, Formosa, Santa Fé, Santiago del Estero, Tucumán). It has been found sporadically outside of this native range in Europe (see Särkinen et al. 2018) and the United States of America (see Knapp et al. 2019), mostly associated with wool waste and sheep-related imports.

##### Ecology and habitat.

*Solanumpilcomayense* is a plant of wet areas in dry forests (Chaco and Chiquitano woodlands) and swampy areas along streams and rivers, from near sea level to 1,000 m elevation.

##### Common names and uses.

No common names recorded on specimens seen. *Solanumpilcomayense* is used to treat “cadillo” (corns and calluses) in folk medicine (Argentina, Corrientes; Martínez Crovetto 1981).

##### Preliminary conservation status

**(IUCN 2022).** Least Concern [LC]. EOO = 15,437,317 km^2^ [LC]; AOO = 768 km^2^ [EN]. *Solanumpilcomayense* is widely distributed along rivers in the Paraná Basin; it has been collected in protected areas in Argentina (e.g., Parque Nacional Iberá, Parque Nacional Río Pilcomayo) and Paraguay (e.g, Parque Nacional Ypoá).

##### Discussion.

*Solanumpilcomayense* is a distinctive species of the Paraná River Basin with broadly triangular leaves widest in the lower quarter with truncate to somewhat hastate bases, large corollas and dark purple berries with two apical stone cells. It often grows in flooded areas along rivers and streams and stems can be very long and sprawling over other vegetation. The calyx lobes are distinctly spathulate, in contrast to the sympatric *S.americanum* with deltate calyx lobes and much smaller (1–1.5 mm versus 3–4 mm long) anthers. *Solanumpilcomayense* also differs from *S.americanum* in its deciduous (versus persistent) fruiting pedicels. Two very old collections of *S.pilcomayense* have been seen in the United States of America, in coastal Texas and New Jersey, probably from 19^th^ century ship’s ballast, but the species has not persisted outside of its native range (see Knapp et al. 2019).

Barboza et al. (2013) cited the sheet of *Morong 898* in NY (barcode 00172130) as the holotype of *S.pilcomayense* in error, no herbaria were cited in the protologue. We here select this sheet explicitly as the lectotype for the name.

Bitter (1912b) cited specimens at B collected by Hassler and Rojas to coin names we here recognise as synonyms of *S.pilcomayense*, these specimens were exactly the same collections Hassler (1909) had previously used to coin his names (mostly at the infraspecific level). Most of these were inadvertently effectively lectotypified by Morton (1976) by citing “holotype” or” isotype” (see above). The only name he did not effectively lectotypify was *S.syringoideum* Bitter which we here lectotypify with the duplicate of *Rojas in Hassler 2324* in BM (BM000087562) so as not make it homotypic with S.nigrumvar.chacoense that Morton (1976) inadvertently lectotypified with the sheet of this number in Geneva.

The various collections of Teodoro Rojas used by Hassler (1909) to describe infraspecific taxa of *S.nigrum* treated here as synonyms of *S.pilcomayense* were also used by Bitter (1912b) to describe *S.pulchrilobum*. In the herbarium at G they are labelled in such a way that a single sheet has on it two barcodes, one for each name. We have lectotypified these names with the same set of specimens and have cited both barcodes that are on individual sheets (e.g. *Rojas 108b* – https://www.ville-ge.ch/musinfo/bd/cjb/chg/adetail.php?id=314895&base=img&lang=en). The way in which specimens are kept at G (see Turland et al. 2018: Art. 8.3, Ex. 9) means that despite the barcoding, these “duplicates” of *Rojas 108b* are all considered the same specimen, thus rendering S.nigrumsubformasinuatodentatum and *S.pulchrilobum* homotypic.

Hassler (1918) treated *S.pilcomayense* in Paraguay as a complex series of names at nested ranks of subspecies, variety and forma. He recognised *S.syringoideum* at the specific level, but in a very confusing paragraph listed all his own (Hassler 1909) and Bitter’s (1912b) previous infraspecific taxa under his S.nigrumvar.chacoense, apparently creating new names, but really only listing them in synonymy and equating taxon concepts.

#### ﻿42. Solanum polytrichostylum

Taxon classificationPlantaeSolanalesSolanaceae

Bitter, Repert. Spec. Nov. Regni Veg. 10: 550. 1912.

Figs 4G , 128 , 129

Solanum violaceistriatum Bitter, Repert. Spec. Nov. Regni Veg. 10: 550. 1912. Type. Bolivia. La Paz, Caminos, 14 May 1906, *O. Buchtien 119* (no herbaria cited; lectotype, designated here: US [00610902, acc. # 700077]; isolectotypes: NY [00172242], S [acc. # S04-2996], US [00027850, acc. # 1175820]).
Solanum irenaeum Bitter, Repert. Spec. Nov. Regni Veg. 10: 551. 1912. Type. Bolivia. La Paz: vic. La Paz, 1889, *M. Bang 31* [a] (holotype: B [destroyed, as “Bang 31 p.p.”]; lectotype, designated here: NY [00173050]; isotypes: BM [BM000617674], BR [BR0000005538546], CAL [acc. # 316753], E [E00279514], G [G00343341], MO [MO-503695, acc. # 1815479], NY [00172051], PH [00030429], US [00027629, acc. # 1324595], W [acc. # 1895-0000969, acc. # 1890-0001435], WIS [v0256195WIS]).
Solanum medianiviolaceum Bitter, Repert. Spec. Nov. Regni Veg. 10: 562. 1912. Type. Bolivia. La Paz: La Paz, *O. Buchtien 2968* (holotype: “herb. Buchtien”; lectotype, designated here: US [00027675, acc. # 1133299]; isolectotypes: GOET [GOET003549, GOET003550, GOET003551. GOET003552], NY [00172085, 00172086], US [00610903, acc. # 700120]).
Solanum aloysiifolium Dunal var. polytrichostylum (Bitter) Edmonds, Bot. J. Linn. Soc. 75: 171. 1977. Type. Based on Solanumpolytrichostylum Bitter.

##### Type.

Bolivia. La Paz: Nor Yungas, Unduavi, 3,200 m, 12 Dec 1907, *O. Buchtien 763* (holotype: “herb. Buchtien”; lectotype, designated here: US [00027753, acc. # 700087]; isolectotypes: M [M-0171818], NY [00172137]).

**Figure 128.**
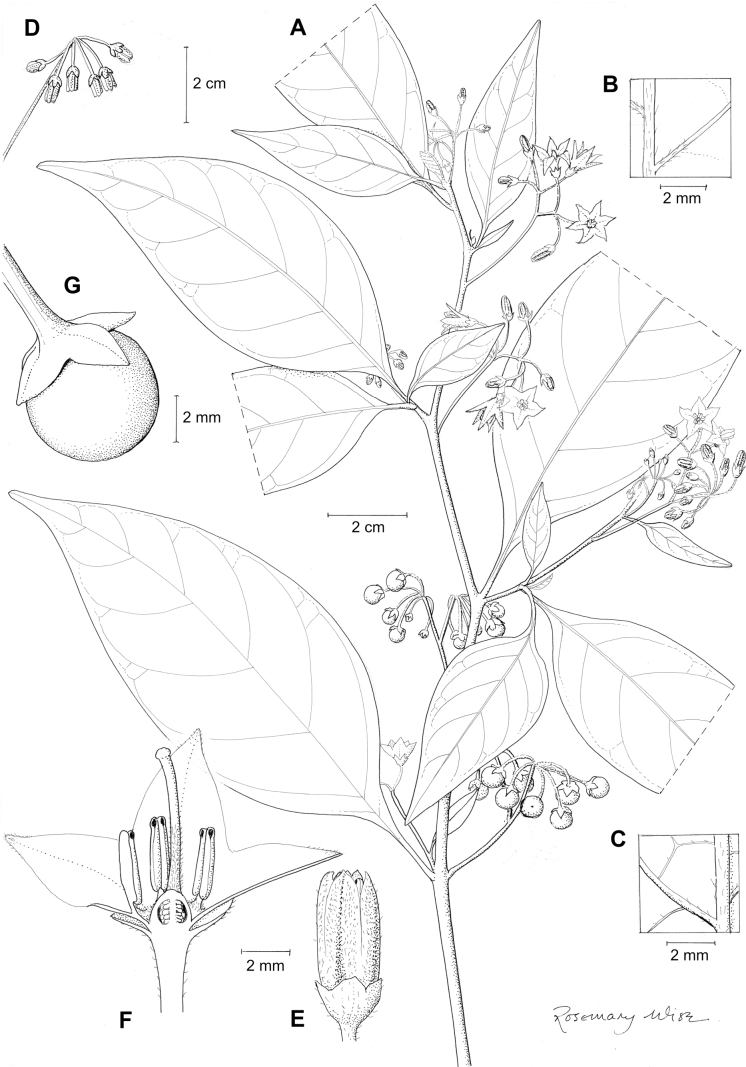
*Solanumpolytrichostylum***A** flowering and fruiting habit **B** detail of adaxial leaf surface **C** Detail of abaxial leaf surface **D** inflorescence in bud **E** flower bud **F** dissected flower **G** fruit (**A***Buchtien 8754***B, C, E–G***Monro et al. 3982***D***Knapp et al. 10274*). Illustration by R. Wise.

##### Description.

Erect herbs to single-stemmed shrubs 1–2.5 m high, the branches erect and ascending. Stems terete, sparsely pubescent with a few scattered white eglandular simple uniseriate 2–6-celled trichomes to 0.5 mm long, soon glabrescent; new growth densely appressed-pubescent with white eglandular simple uniseriate trichomes to 1 mm long; bark of older stems greenish brown, glabrescent. Sympodial units difoliate, the leaves not geminate. Leaves simple, the blades 3.3–18 cm long, 1.4–8 cm wide, elliptic to narrowly elliptic, widest at the middle, membranous, concolorous; adaxial surfaces almost glabrous with a few tiny eglandular simple uniseriate trichomes ca. 0.2 mm long, these more commonly found along the veins; abaxially glabrous on the lamina, moderately pubescent with eglandular simple uniseriate trichomes ca. 0.2 mm long on the veins; principal veins 8–9 pairs, moderately white-pubescent abaxially; base attenuate; margins entire or occasionally with a few small teeth to 2 mm long in the basal part of the blade; apex acuminate; petioles 0.6–2.5 cm long, not markedly winged, very sparsely pubescent with simple uniseriate trichomes like those of the leaf venation. Inflorescences internodal or opposite the leaves at branching points, many times branched, 5–10 cm long, with 10–50 flowers clustered at the tips of the branches, sparsely pubescent with white eglandular simple uniseriate 2–6-celled trichomes like those of the stems, but these weak and tangled; peduncle 2–5 cm long; pedicels 1–13 cm long, ca. 1 mm in diameter at the base, ca. 1.5 mm in diameter at the apex, slightly tapering, spreading at anthesis, sparsely pubescent with tangled trichomes like those of the rest of the inflorescence, articulated at the base; pedicel scars tightly spaced at the tips of inflorescence branches. Buds narrowly ellipsoid, ellipsoid to slightly ovate and flattened at the tip, wider in the lower third, the corolla strongly exserted from the calyx before anthesis, in live plants the buds markedly striped with purple and white. Flowers 5-merous, cosexual (hermaphroditic). Calyx tube 1–1.5 mm long, cup-shaped, the lobes 1–1.5 mm long, ca. 1 mm wide, deltate with a distinct mucro ca. 0.5 mm long from the obtuse tip, sparsely pubescent with white eglandular simple uniseriate 2–6-celled trichomes like the rest of the inflorescence. Corolla 1.9–2.2 cm in diameter, white with a dark purple petal midvein and a green eye, stellate to deeply stellate, lobed halfway to 3/4 of the way to the base, the lobes ca. 6 mm long, ca. 3 mm wide, narrowly deltate, spreading or reflexed, adaxially glabrous, abaxially sparsely papillate-puberulent, densely so on the tips and margins. Stamens equal; filament tube minute; free portion of the filaments 0.75–1 mm long, densely pubescent with tangled transparent simple uniseriate trichomes adaxially; anthers 4–4.5 mm long, ca. 1.2 mm wide, ellipsoid, yellow, poricidal at the tips, the pores lengthening to slits with age. Ovary conical, glabrous; style 6.5–9 mm long, straight, exserted beyond the anther cone, densely pubescent with tangled simple uniseriate trichomes to 0.4 mm long in the lower 2/3; stigma capitate to slightly bilobed, bright green in live plants, the surface minutely papillose. Fruit a globose or occasionally slightly flattened berry, 1–1.2 cm in diameter, green when immature, dark green when ripe, the pericarp thin, matte to slightly shiny, opaque but becoming slightly translucent on ripening, glabrous; fruiting pedicels 1.3–1.7 cm long, ca. 1 mm in diameter at the base, ca. 1.5 mm in diameter at the apex, slightly woody, deflexed, not persistent; fruiting calyx not enlarged in fruit, the lobes appressed to the berry. Seeds 40–80 per berry, 1.5–2 mm long, 1–1.5 mm wide, flattened and teardrop shaped, pale tan, the surfaces minutely pitted, the testal cells sinuate in outline. Stone cells 6 per berry, 2 apical and 4 equatorial, ca. 0.7 mm in diameter, cream-coloured. Chromosome number: 2n = 24 (Chiarini et al. 2017, voucher *Knapp et al. 10384*).

**Figure 129.**
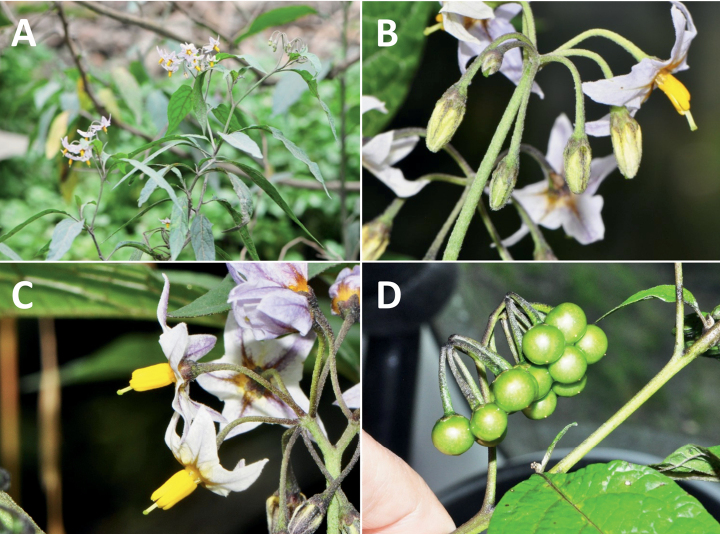
*Solanumpolytrichostylum***A** habit **B** inflorescence with buds **C** flowers at full anthesis **D** infructescence with maturing fruits (**A***Särkinen & Correa 5277***B***Knapp et al. 10439***C***Knapp et al. 10414***D***Knapp et al. 10437*). Photos by S. Knapp.

##### Distribution

**Figure 130.**
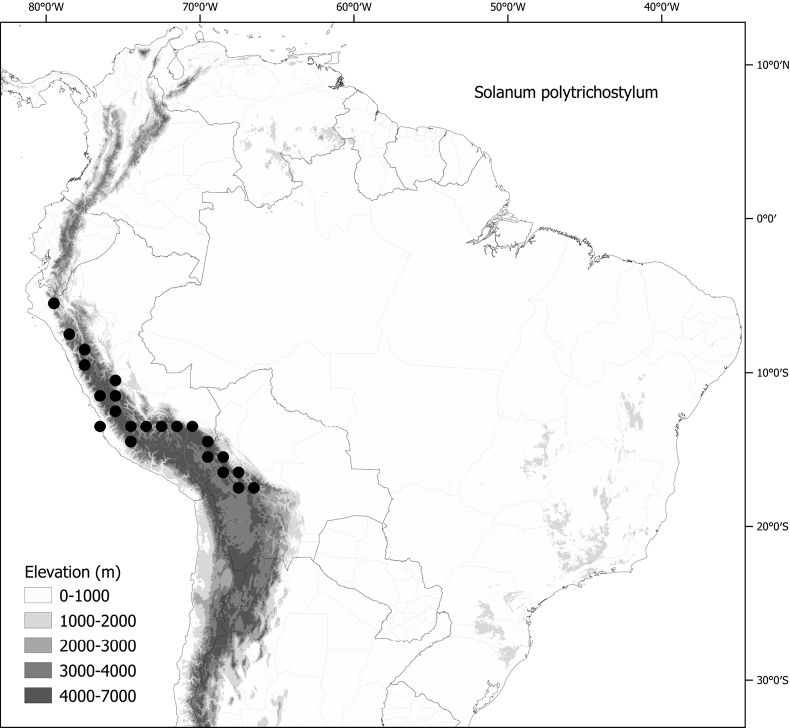
Distribution map of *Solanumpolytrichostylum*.

**(Fig. 130).***Solanumpolytrichostylum* is a plant of Andean slopes from central Peru (Depts. Ancash, Apurímac, Ayacucho, Cajamarca, Cusco, Huancavelica, Junín, Lima, Pasco, Piura) and Bolivia (Depts. Cochabamba, La Paz). In Peru it has been collected both on the eastern and western slopes of the Andes.

##### Ecology and habitat.

*Solanumpolytrichostylum* grows in wet forests and cloud forests (‘yungas’), often in marginal sites or landslides, from 2,000 to 4,000 m elevation.

##### Common names and uses.

Peru. Cusco: ccaya-ccaya (*Herrera 3022*). No uses recorded.

##### Preliminary conservation status

**(IUCN 2022).** Least Concern [LC]. EOO = 432,164 km^2^ [LC]; AOO = 244 km^2^ [EN]. *Solanumpolytrichostylum* is widely distributed and is a plant of disturbed areas wherever it occurs. It is found within the World Heritage Site of Machu Picchu (Peru) and is common among protected archaeological sites in the Sacred Valley of Cusco (e.g., Ollantaytambo [Ullantaytampu], Pisac).

##### Discussion.

**Figure 131.**
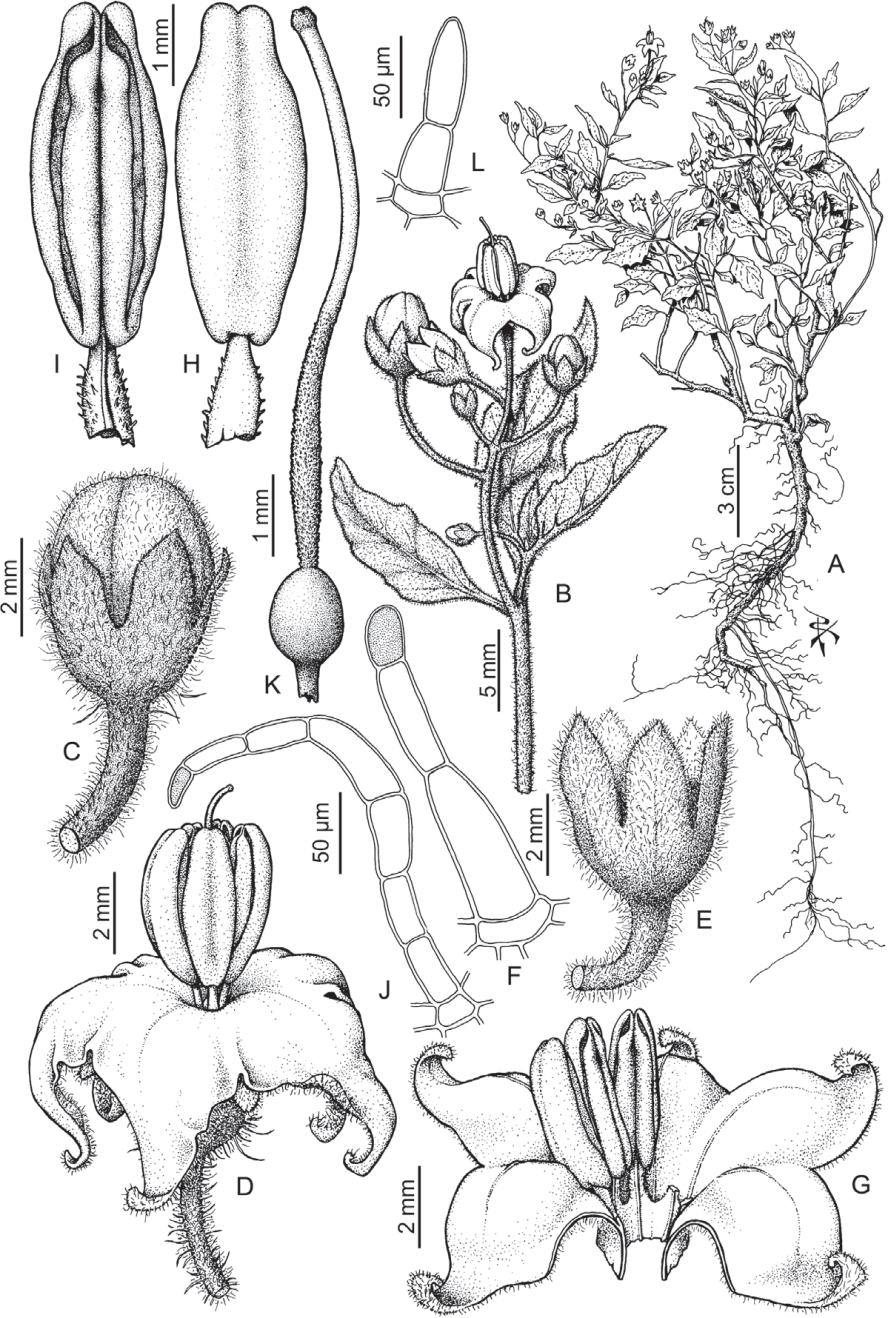
*Solanumprofusum***A** habit **B** flowering branch **C** flower bud **D** flower **E** calyx **F** glandular trichome of the calyx **G** dissected flower **H** stamen, dorsal view **I** stamen, ventral view **J** glandular trichome of the filament **K** gynoecium **L** eglandular trichome of the style (**A–L***Balls 5915*). Illustration by P. Peralta. Previously published in Barboza et al. (2013: 255).

*Solanumpolytrichostylum* is a coarse erect herb morphologically very similar to *S.antisuyo*, with which it is broadly sympatric. It differs from *S.antisuyo* in its more elliptic (rather than ovoid) buds that are prominently striped with purple and white in both live and dried plants (Fig. 131C, D), flowering pedicels without a strong taper (as opposed to strongly tapered), its longer calyx lobes (1–1.5 mm versus 0.6–0.9 mm) with a distinct mucro apically and the larger number of stone cells in mature berries (6 versus 0–2). The anthers of *S.polytrichostylum* are slightly longer (4–4.5 mm long) than those of *S.antisuyo* (3–3.4 mm long), but this can be difficult to measure. The most reliable characters for identification are the bud shape and pedicel tapering, although *S.polytrichostylum* generally has more branched inflorescences than does *S.antisuyo*. *Solanumpolytrichostylum* often grows in dense stands along river edges in otherwise cultivated areas.

Bitter (1912a) cited a specimen from “herb. Buchtien” as the sole element for *S.polytrichostylum*. Buchtien’s Herbarium was acquired by the US National Herbarium during Paul C. Standley’s curatorship (Morton and Stern 1966), so we have lectotypified *S.polytrichostylum* with the sheet in US (barcode 00027753, acc. # 700087).

In the same publication Bitter (1912a) described all of the rest of the names we here recognise as synonyms of *S.polytrichostylum*. *Solanumviolaceistriatum* was described citing *Buchtien 119*, but with no herbarium indicated; we lectotypify this with the sheet in US (barcode 00610902, acc. # 700077) with the date of collection cited in the protologue, and an annotation of this species name in Buchtien’s hand. The protologue of *S.irenaeum* cited *Bang 31 pro parte* from Berlin (“herb. Berol.”). This specimen is no longer extant and duplicates of *Bang 31* are a mixed collection of *S.polytrichostylum* and *S.pallidum* (see under *S.pallidum*). We select here as the lectotype for this name the duplicate of *Bang 31* in NY (barcode 00173050) with both flowers and fruits that has the original determination (“S. nudum HBK”) crossed out and “S.irenaeum Bitter sp. n.” written in Rusby’s hand. *Solanummedianiviolaceum* was based on *Buchtien 2968* (“herb. Boliv.”); we lectotypify this name with the sheet in US from Buchtien’s herbarium (US barcode 00027675, acc. # 1133299) annotated as “Solanummedianiviolaceum Bitt. n.sp.” in Buchtien’s hand.

#### ﻿43. Solanum profusum

Taxon classificationPlantaeSolanalesSolanaceae

C.V.Morton, Revis. Argentine Sp. Solanum 86. 1976.

Figs 131 , 132

##### Type.

Argentina. Jujuy: Dpto. Dr. Manuel Belgrano, near Jujuy, 2 May 1939, *E.K. Balls 5915* (holotype: US [00027757, acc. # 1779255]; isotypes: E [E00298913], UC [UC683471]).

**Figure 132.**
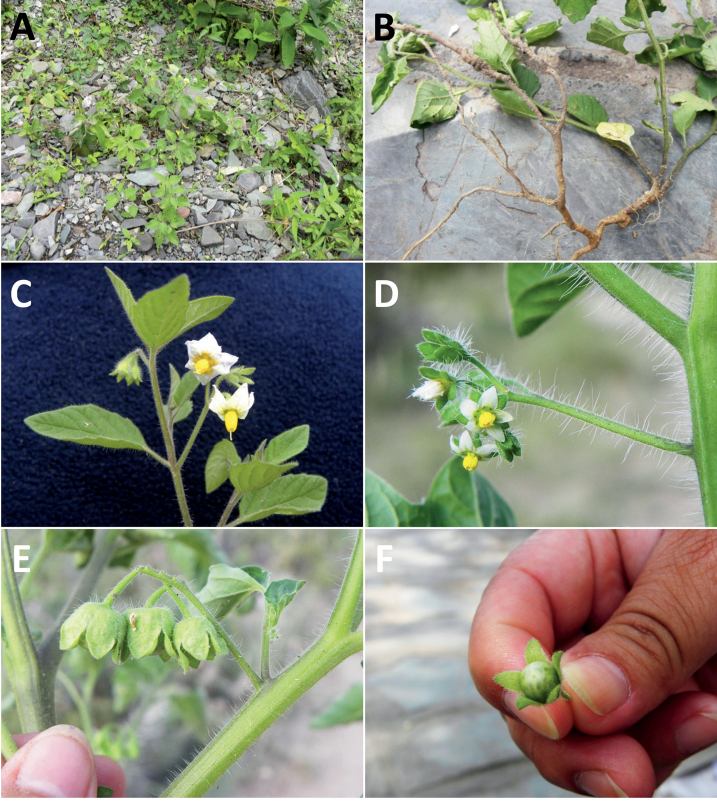
*Solanumprofusum***A** habit **B** underground rhizome **C** flowering branch **D** inflorescence with flowers at full anthesi **E** infructescence with maturing fruits **F** developing fruit inside the enlarged calyx (**A–F***Barboza 4928*). Photos by G.E. Barboza.

##### Description.

Prostrate, perennial herb to 0.2 m high, somewhat woody at the base, rooting along the nodes and from rhizomes and forming large populations. Stems terete, sprawling, densely glandular pubescent with transparent 2–3-celled, simple uniseriate trichomes mostly 0.5 mm long, some to 1.5 mm long; new growth densely glandular-pubescent with simple uniseriate trichomes like those of the stems, densely papillate with tiny glandular trichomes on leaf laminar surfaces; bark of older stems greenish brown. Sympodial units difoliate, the leaves not geminate. Leaves simple, entire or occasionally very shallowly toothed, the blades 3–4 cm long, 1.2–1.5 cm wide, narrowly elliptic, widest at the middle, membranous, concolorous; adaxial surfaces sparsely to moderately and evenly glandular-pubescent with 2–3-celled, simple, uniseriate trichomes 1–1.5 mm long, occasionally some shorter; abaxial surfaces similarly glandular-pubescent, but trichomes on the lamina somewhat longer than those on the veins; principal veins 3–4 pairs; base attenuate along entire petiole; margins entire or very shallowly toothed, the teeth if present ca. 1 mm long, very broadly deltate with rounded tips; apex acute, with the ultimate tip usually somewhat rounded; petiole 0.1–0.6 cm long, glandular-pubescent like the stems and leaves. Inflorescences opposite the leaves or internodal, unbranched, 0.6–2.3 cm long, with 3–6 flowers in the distal half, densely glandular-pubescent with transparent, simple uniseriate trichomes like those of the stems; peduncle 0.5–2 cm long; pedicels 0.9–1 cm long, ca. 0.5 mm in diameter at the base, ca. 1.2 mm in diameter at the apex, tapering, densely glandular-pubescent, spreading at anthesis and the flowers nodding, articulated at the base leaving small areas of darker tissue after abscission; pedicel scars irregularly spaced 1.5–2 mm apart. Buds globose to short-ellipsoid, the corolla ca. 1/4 exserted from the calyx lobes before anthesis. Flowers 5-merous, cosexual (hermaphroditic). Calyx tube 1.5–2 mm long, conical, the lobes 2.5–3 mm long, 1.5–2 mm wide, lanceolate with the tips acute or slightly rounded, densely glandular-pubescent like the pedicels. Corolla 1.5–1.8 cm in diameter, white or pale lilac (with age) with a green central eye, stellate, lobed halfway to 2/3 of the way to the base, the lobes 5–5.5 mm long, 3.5–4 mm wide, broadly triangular, strongly reflexed at anthesis, glabrous adaxially, densely glandular-pubescent on the midvein, tips and margins abaxially, the trichomes longer at the tips. Stamens equal; filament tube minute; free portion of the filaments 0.5–1 mm long, sparsely pubescent with tangled transparent simple uniseriate trichomes adaxially; anthers 3–3.5 mm long, 1.2–1.5 mm wide, ellipsoid, yellow, poricidal at the tips, the pores lengthening to slits with age. Ovary conical, glabrous; style 6–6.5 mm long, straight, exserted beyond the anther cone, densely pubescent in the lower third with transparent, eglandular simple uniseriate trichomes; stigma capitate to globose, the surface minutely papillate. Fruit a globose berry, 0.7–0.9 cm in diameter, green or pale green when mature, opaque, the pericarp thin, matte, glabrous; fruiting pedicels ca. 1 cm long, ca. 0.5 mm in diameter at the base, ca. 2 mm in diameter at the apex, not markedly woody, strongly deflexed at the base, not persistent; fruiting calyx accrescent, appressed to the berry, the tube 3.5–5 mm long, the lobes 4–5.5 mm long, 3.5–4 mm wide, enclosing the berry ca. halfway (approximately half of the berry visible beyond the calyx lobes). Seeds ca. 20 per berry, ca. 1.5 mm long, ca. 1.2 mm wide, flattened and teardrop shaped, tan to reddish gold, the surfaces minutely pitted, the testal cells rectangular-pentagonal, those near the margins longer and thinner. Stone cells absent. Chromosome number: not known.

##### Distribution

**Figure 133.**
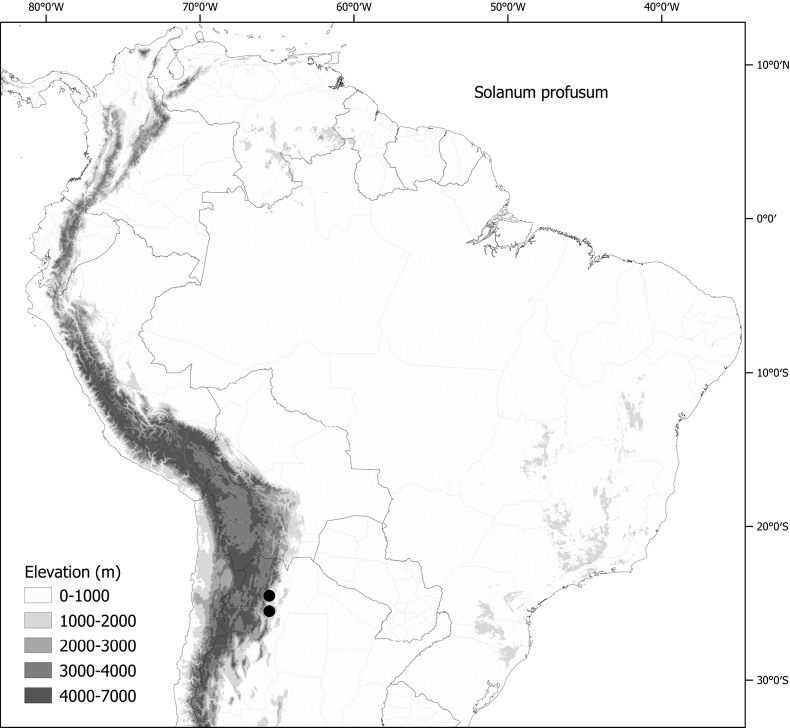
Distribution map of *Solanumprofusum*.

**(Fig. 133).***Solanumprofusum* is endemic to the Andes of northern Argentina (Provs. Jujuy, Salta).

##### Ecology and habitat.

*Solanumprofusum* is a plant of open rocky areas along streams or grasslands with patches of semideciduous forest, from 1,200 to 1,500 m elevation.

##### Common names and uses.

None recorded.

##### Preliminary conservation status

**(IUCN 2022).** Endangered [EN, B1, 2 a,b(ii)]. EOO = 4,852 km^2^ [EN]; AOO = 28 km^2^ [EN]. *Solanumprofusum* is known from only four sites (some of these with imprecise localities) and has a narrow geographic range. Some of its populations occur very near to the expanding urbanisation of Salta; subsequent searches for plants in historical collecting sites have been unsuccessful. *Solanumprofusum* may be more resilient to disturbance than we think though, as it is rhizomatous and is able to spread vegetatively; this, however, does not assist with genetic diversity. *Solanumprofusum* does not occur in any protected areas.

##### Discussion.

*Solanumprofusum* is one of several glandular-pubescent species of morelloids with accrescent to somewhat accrescent calyces occurring in north-central Argentina (e.g., *S.physalidicalyx*, *S.physalifolium*, *S.tweedieanum*). *Solanumprofusum* differs from *S.physalidicalyx* and *S.tweedieanum* in lacking a strongly accrescent calyx that completely covers the mature berry. It has narrower, less incised leaves than either of those two species and has shorter anthers than *S.tweedieanum* (3–3.5 mm long versus 4–6 mm long). Plants in flower can be difficult to identify. *Solanumprofusum* does not overlap in distribution with *S.physalifolium*, whose berries are not consistently covered by an accrescent calyx. *Solanumprofusum* is a rhizomatous perennial and possibly clonal, while *S.physalifolium* is an annual. Leaf shape also differs, *S.profusum* has more lanceolate to lance-elliptic leaves, while those of *S.physalifolium* and *S.physalidicalyx* are ovate to elliptic ovate. The distributions of the three species do not overlap. A key to the glandular-pubescent morelloids in Argentina can be found in Knapp et al. (2020).

#### ﻿44. Solanum pseudoamericanum

Taxon classificationPlantaeSolanalesSolanaceae

Särkinen, P.Gonzáles & S.Knapp, PhytoKeys 31: 10. 2013.

Figs 134 , 135

##### Type.

Peru. Cajamarca: Prov. Cajabamba, in town of Cajabamba, 7°36'43"S, 78°03'28"W, 2,649 m, 9 May 2013, *S. Knapp, T. Särkinen, H.M. Baden, P. Gonzáles & E. Perales 10575* (holotype USM; isotypes BM [BM001120840], CORD [CORD00006824], CPUN, E [E00700636], HUT).

**Figure 134.**
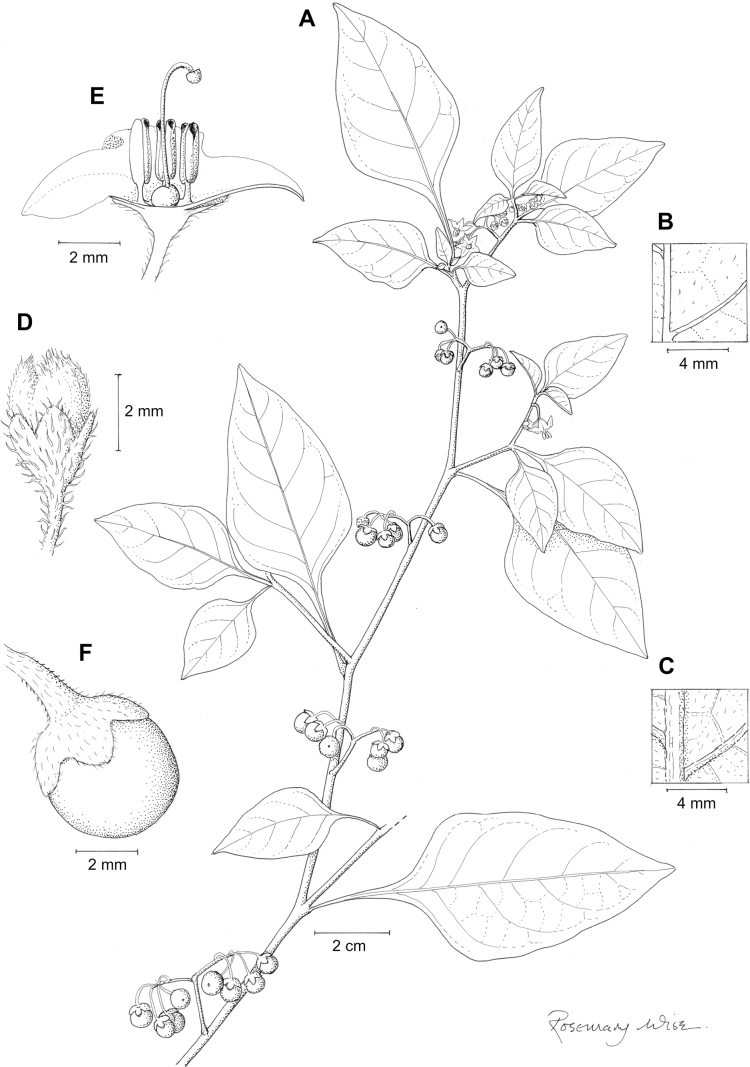
*Solanumpseudoamericanum***A** flowering and fruiting habit **B** detail of adaxial leaf surface **C** detail of abaxial leaf surface **D** flower bud **E** dissected flower **F** fruit (**A–F***Knapp et al. 10300*, *Knapp et al. 10351*). Illustration by R. Wise. Previously published in Särkinen et al. (2013: 13).

##### Description.

Herb with woody base, 0.2–0.6 m high, the individual stems to 1 m long and sprawling. Stems terete or somewhat angled with ridges, pubescent with simple, uniseriate 1–4-celled trichomes, these often clustered along the stem angles; new growth densely pubescent with appressed 1–4-celled simple, uniseriate trichomes 0.2–0.8 mm long. Sympodial units difoliate, not geminate. Leaves simple and shallowly toothed, the blades 4.5–12(–15) cm long, 1.8–8 cm wide, ovate to elliptic, widest near or just below the middle, membranous, somewhat discolorous; adaxial surface sparsely pubescent with more or less appressed 1–4-celled translucent simple, uniseriate trichomes, these denser along the veins; abaxial surface more densely pubescent with simple uniseriate trichomes like those of the upper surface; principal veins 5–8 pairs; base acute and decurrent on the petiole; margins entire or occasionally with shallow lobes in the basal third; apex acute; petiole 0.5–2.5(–5) cm long, occasionally narrowly winged, sparsely pubescent with simple uniseriate trichomes like those of the stems and leaves. Inflorescences internodal, unbranched or forked, 1–2.5 cm long, with 3–5(9) flowers, sparsely pubescent with appressed 1–2-celled simple uniseriate trichomes; peduncle 0.4–1.6 cm long, if the inflorescence branched, then the peduncle of each branch 0.4–0.6 cm long; pedicels 0.6–0.7 cm long, ca. 0.3 mm in diameter at the base and apex, straight and spreading, articulated at the base; pedicel scars spaced ca. 1 mm apart. Buds globose, the corolla only exserted from the calyx tube just before anthesis. Flowers 5-merous, cosexual (hermaphroditic). Calyx tube ca. 1 mm long, the lobes 0.5–0.7 mm long with rounded apices, sparsely pubescent with 1–4-celled translucent simple uniseriate trichomes. Corolla 0.5–0.6 cm in diameter, stellate, white with a yellow central portion near the base, lobed slightly less than halfway to the base, the lobes ca. 1.5 mm long, 2 mm wide, strongly reflexed at anthesis, later spreading, densely pubescent abaxially with 1–4-celled simple uniseriate trichomes, these usually shorter than the trichomes of the stems and leaves. Stamens equal; filament tube minute, pubescent with tangled uniseriate trichomes adaxially; free portion of the filaments ca. 1 mm long, pubescent like the tube; anthers 1–1.5 mm long, 0.7–0.8 mm wide, ellipsoid, yellow, poricidal at the tips, the pores lengthening to slits with age. Ovary conical, glabrous; style 3–4 mm long, straight, somewhat long-exserted beyond the anther cone, densely pubescent with 2–3-celled simple uniseriate trichomes at the base; stigma globose and capitate, minutely papillate, bright green in live plants. Fruit a globose berry, 0.4–0.9 cm in diameter, green at maturity or green and turning purplish black when ripe, the pericarp not markedly shiny, opaque, glabrous; fruiting pedicels 0.4–0.7 cm long, ca. 1 mm in diameter at the base, ca. 1.2 mm in diameter at the apex, spreading and becoming somewhat more woody in fruit, persistent and usually remaining on the plant after fruit drops; fruiting calyx lobes spreading or appressed to the berry, not reflexed. Seeds 35–45 per berry, 1.2–1.5 mm long, 0.9–1 mm wide, flattened-reniform, yellowish straw-coloured, the surfaces minutely pitted, the testal cells pentagonal in outline. Stone cells absent. Chromosome number: not known.

**Figure 135.**
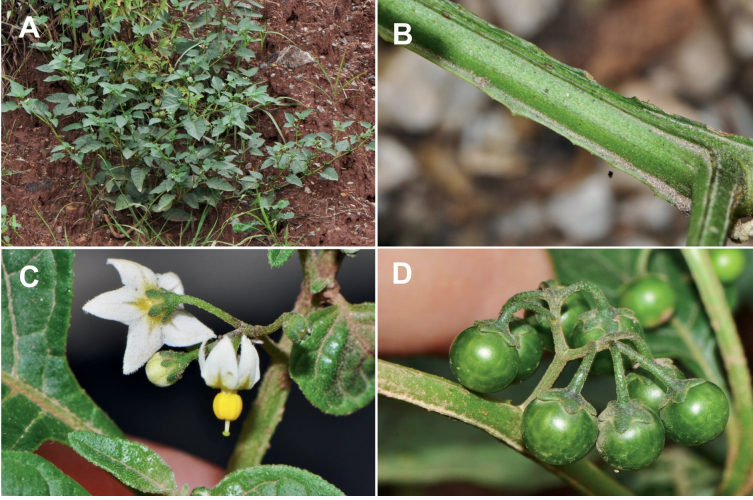
*Solanumpseudoamericanum***A** habit **B** ridged stem **C** inflorescence with buds and flowers at full anthesis **D** maturing fruits (**A***Särkinen et al. 4640***B***Knapp et al. 10357***C, D***Knapp et al. 10300*). Photos by S. Knapp and T. Särkinen. Previously published in Särkinen et al. (2013: 15).

##### Distribution

**Figure 136.**
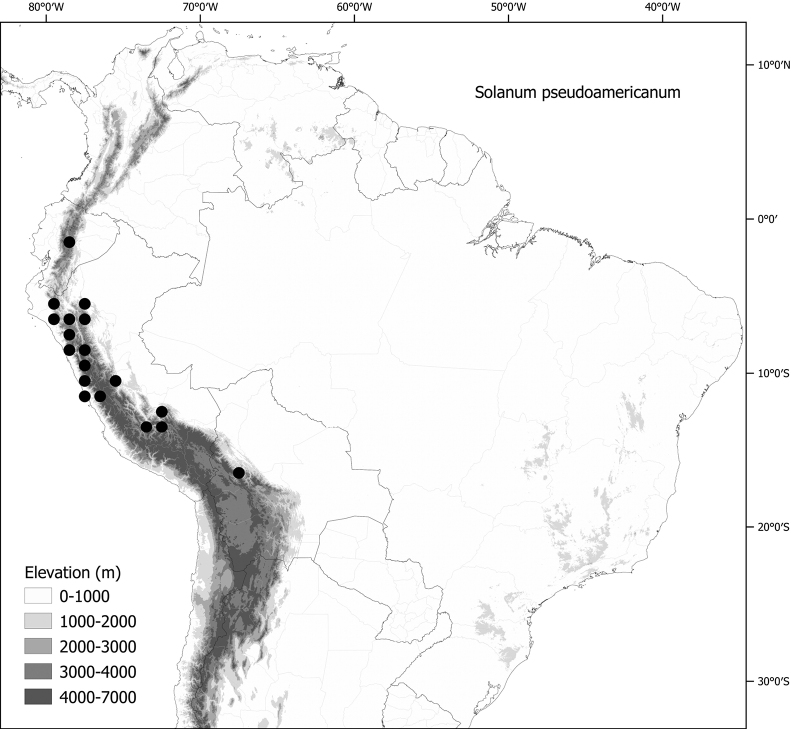
Distribution map of *Solanumpseudoamericanum*.

**(Fig. 136).***Solanumpseudoamericanum* occurs from southern Ecuador (Prov. Imbabura), throughout Andean Peru (Depts. Amazonas, Ancash, Apurímac, Ayacucho, Cajamarca, Cusco, Huánuco, La Libertad, Lima, Pasco, Piura) to northern Bolivia (Dept. La Paz).

##### Ecology and habitat.

*Solanumpseudoamericanum* grows in the upper zones of seasonally dry tropical forests to mid-elevation montane forests, commonly growing in sandy soils in full sun or partial shade in disturbed sites such as landslides and roadsides or cultivated areas, often in moist depressions in otherwise dry areas, from (930-)1,700 to 3,200(-3,735) m in elevation. A single anonymous collection recorded as occurring at 100 m elevation in the Department of Lima may be a label error.

##### Common names and uses.

Peru. Ancash: atoqpa papan (*Gamarra 416*); Amazonas: hierba mora (*García Llatas 8155*); Lima: hierba mora (*Vilcapoma 1649a, 5330*). No uses recorded.

##### Preliminary conservation status

**(IUCN 2022).** Least Concern [LC]. EOO = 668,293 km^2^ [LC]; AOO = 180 km^2^ [EN]. *Solanumpseudoamericanum*, previously unrecognised as distinct from the widespread *S.americanum*, has been collected more often since its description, increasing the range and number of populations greatly. *Solanumpseudoamericanum* occurs in at least one protected area in Peru (e.g., Lomas de Lachay).

##### Discussion.

*Solanumpseudoamericanum* can be distinguished from the similar *S.americanum* by the following suite of characters; berries that are matte or somewhat shiny at maturity, versus very shiny in *S.americanum*, styles that are always exserted to approximately equal to the length of the anther cone, versus styles almost included in the anther cone in *S.americanum* and the globose, bright green stigmas, versus a white or pale green stigmas that are merely a widening of the style tip in *S.americanum*. *Solanumpseudoamericanum* usually occurs above 2,000 m elevation, with only some overlap between the closely related *S.americanum* that occurs from sea level to 2,200 m in elevation.

Other members of the Morelloid clade in Peru without glandular trichomes which grow sympatrically with *S.pseudoamericanum* differ from it in being larger in growth form (reaching up to 2 m in height), having larger, violet flowers and fruits that are green at maturity (*S.cochabambense*, *S.interandinum*), or being smaller herbs up to 30 cm high with similarly sized flowers but red, orange or yellow berries (*S.corymbosum*, *S.palitans*, *S.radicans*). *Solanumlongifilamentum* is somewhat similar to *S.pseudoamericanum* but has longer anthers (2–3.4 mm long versus 1–1.5 mm long) and more ellipsoid buds.

#### ﻿45. Solanum pygmaeum

Taxon classificationPlantaeSolanalesSolanaceae

Cav., Icon. 5: 23, tab. 439. 1799.

**Figure 137.**
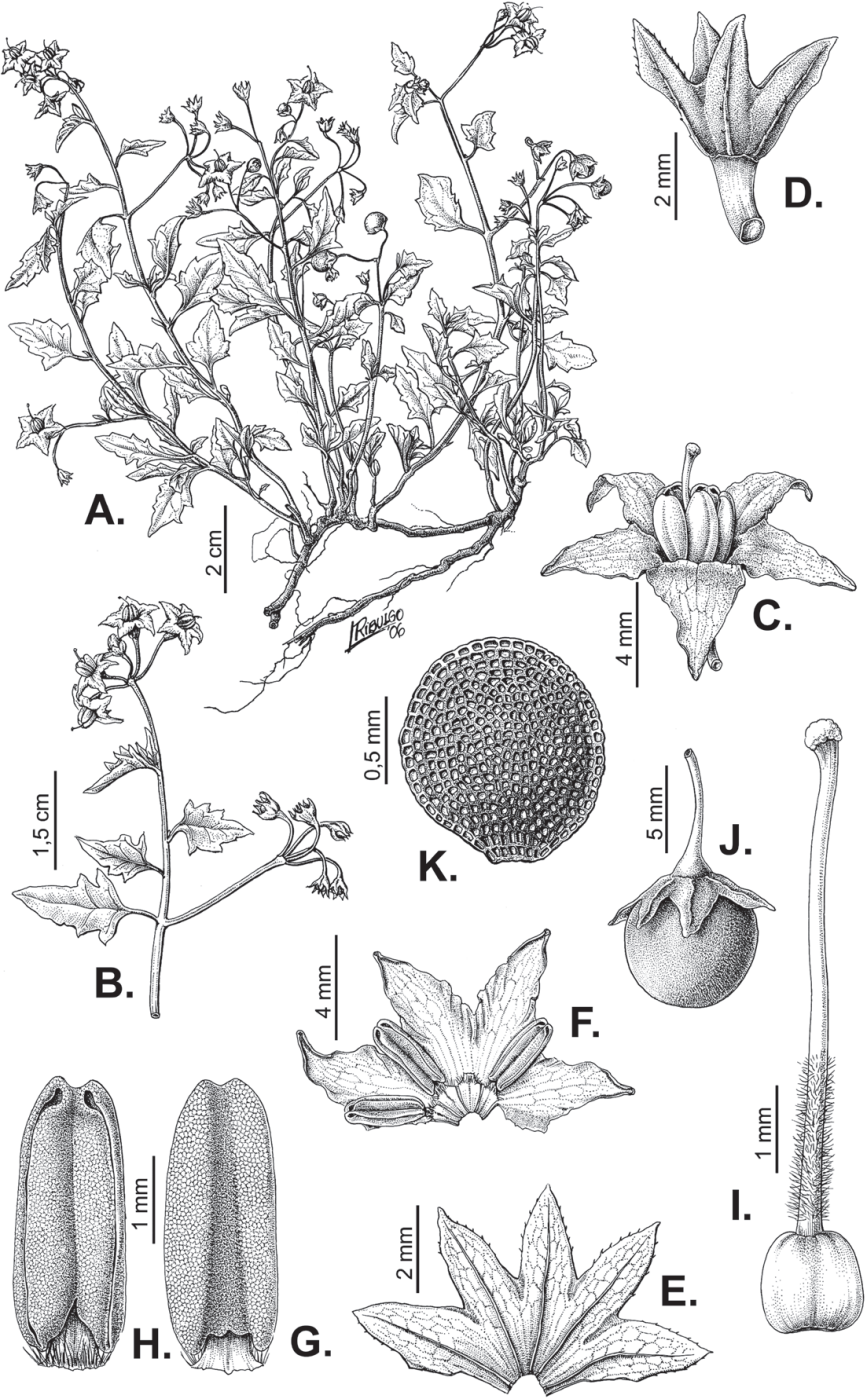
*Solanumpygmaeum***A** habit **B** flowering branch **C** flower **D** calyx **E** dissected calyx, adaxial surface **F** dissected flower **G** stamen, dorsal view **H** stamen, ventral view **I** gynoecium **J** fruit **K** seed (**A–K***Bernardello & DiFulvio 476*). Illustration by L. Ribulgo. Previously published in Barboza et al. (2013: 256) and Särkinen et al. (2018: 130).

**Figure 138.**
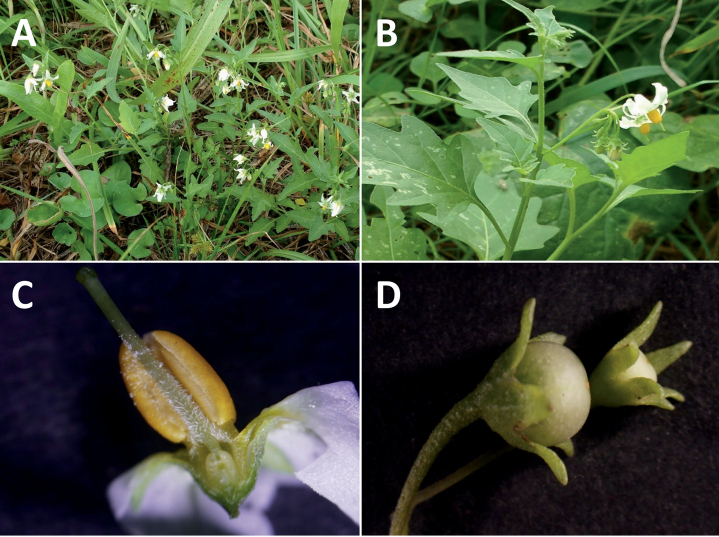
*Solanumpygmaeum***A** habit **B** flowering branch **C** dissected flower **D** developing fruits (**A–D***Chiarini 1341*). Photos by F. Chiarini. Previously published in Särkinen et al. (2018: 131).

Figs 137 , 138 Types based on American specimens only; for full synonymy, see Särkinen et al. (2018: 167–168)

Solanum pygmaeum Cav. var. hastatum Bonte ex Aellen, Ber. Schweiz. Bot. Ges. 50: 236. 1940. Type. Argentina. Buenos Aires: Pergamino, J.A. de la Peña, 14 Jan 1925, *L.R. Parodi 6107* (lectotype, designated here: BAA [BAA00004675]).
Solanum pygmaeum Cav. var. suspensum C.V.Morton, Revis. Argentine Sp. Solanum 138. 1976. Type. Argentina. Córdoba: Alta Córdoba, barrio de la ciudad de Córdoba, *T. Stuckert 4713* (holotype: G; isotypes: CORD [CORD00004273, CORD00004274]).
Solanum deterrimum C.V.Morton, Revis. Argentine Sp. Solanum 138. 1976. Type. Argentina. Buenos Aires: Sierra de la Ventana, 23 Feb 1944, *H. Ruíz de Huidrobo 1332* (holotype: A [00077613]; isotypes: NY [00139129], S [acc. # 12-27773], SI [003308, 003307]).

##### Type.

Argentina. Buenos Aires: “in Pampas de Buenos Ayres esquina de Ballesteros”, Sep, *L. Née, s.n.* (lectotype, designated by Knapp 2007, pg. 200: MA [MA-476361]; isolectotype: G [G00357891]).

##### Description.

Perennial small upright herbs to 0.3 m high, subwoody at base, perennating via underground rhizomes. Stems decumbent or ascending, delicate, terete or somewhat angled with ridges, not markedly hollow; new growth pubescent with simple, appressed, uniseriate, translucent, eglandular trichomes, these 1–6-celled, 0.2–0.5 mm long, or nearly glabrous; older stems glabrous or glabrescent. Sympodial units difoliate, the leaves not geminate. Leaves simple, occasionally lobed, the blades 1–5 cm long, 0.5–3 cm wide, ovate to narrowly elliptic, widest in the lower half or near the middle, membranous, concolorous; adaxial surface glabrous or sparsely pubescent along leaf lamina and margins with simple, uniseriate trichomes like those on stem; abaxial surface sparsely pubescent with similar trichomes but the pubescence denser along the midrib; major veins 3–4 pairs; base attenuate, decurrent on the petiole; margins sinuate to entire, if sinuate then teeth more common in lower part of the blade; apex acute to obtuse; petioles 0.5–1.7 cm long, with scattered simple, appressed, uniseriate eglandular trichomes like those on stem. Inflorescences generally internodal, unbranched or rarely forked, umbelliform to subumbelliform, 1–3 cm long, with (2-)4–6 flowers clustered at the tip, glabrous or with scattered simple, appressed, uniseriate eglandular trichomes like those on stem; peduncle (1.3-)1.5–2.6 cm long, delicate; pedicels 6–13 mm long, 0.5–1 mm in diameter at the base, ca. 1 mm in diameter at the apex, straight and spreading, articulated at the base; pedicel scars spaced ca. 0–2.5 mm apart. Buds globose to broadly ovoid, the corolla strongly exserted from the calyx tube but only halfway exserted beyond the elongate and reflexed calyx lobes before anthesis. Flowers 5-merous, cosexual (hermaphroditic). Calyx tube (0.5–)1.7–2(–2.2) mm long, conical, the lobes 1.5–1.8 mm long, 0.7–0.9 mm wide, narrowly elliptic with long-acuminate to acute apices, glabrous to sparsely pubescent with simple uniseriate eglandular trichomes like those on stem. Corolla 0.9–1.6 cm in diameter, white to pale lilac with a yellow-green central portion near the base, stellate, lobed halfway to the base, the lobes 5–6.7 mm long, ca. 3–3.5 mm wide, strongly reflexed at anthesis, later spreading, glabrous to sparsely pubescent abaxially with simple uniseriate trichomes like those of the stem but shorter. Stamens equal; filament tube minute; free portion of the filaments 1–1.2 mm long, adaxially pubescent with tangled uniseriate 4–9-celled eglandular trichomes to 0.5 mm long; anthers (3-)3.5–3.8 mm long, 0.7–1 mm wide, oblong-ellipsoid, yellow, poricidal at the tips, the pores lengthening to slits with age and drying. Ovary globose, glabrous; style ca. 6.3 mm long, straight, exserted beyond the anther cone, densely pubescent with (1-)2–3-celled simple uniseriate trichomes along 4/5 from the base; stigma capitate to clavate, bilobed, minutely papillate, green in live plants. Fruit a subglobose berry, 0.8–1 cm in diameter, greyish green at maturity, the pericarp opaque and glaucous, glabrous; fruiting pedicels 12–15 mm long, ca. 1 mm in diameter at the base and at the apex, deflexed and often somewhat curved, not persistent; fruiting calyx not accrescent, lobes 1.5–2 mm long, lobes appressed against the berry. Seeds 30-more than 50 per berry, 1.8–2 mm long, 1.2–1.4 mm wide, flattened and teardrop shaped with a subapical hilum, pale yellow, the surfaces minutely pitted, the testal cells irregularly quadrate in outline. Stone cells (4)6–8, the 2 apical ones 1.5–2 mm in diameter, usually very closely paired, the rest equatorial and 1–1.2 mm in diameter, pale whitish brown. Chromosome number: n = 12 (Moscone 1992, vouchers *Bernardello & Di Fulvio 476*, *Moscone 99*, *Subils 3382*; one individual from *Bernadello & Di Fulvio* 476 with n = 18 and chromosomal anomalies with supernumerary bivalents or univalents not segregating).

##### Distribution

**Figure 139.**
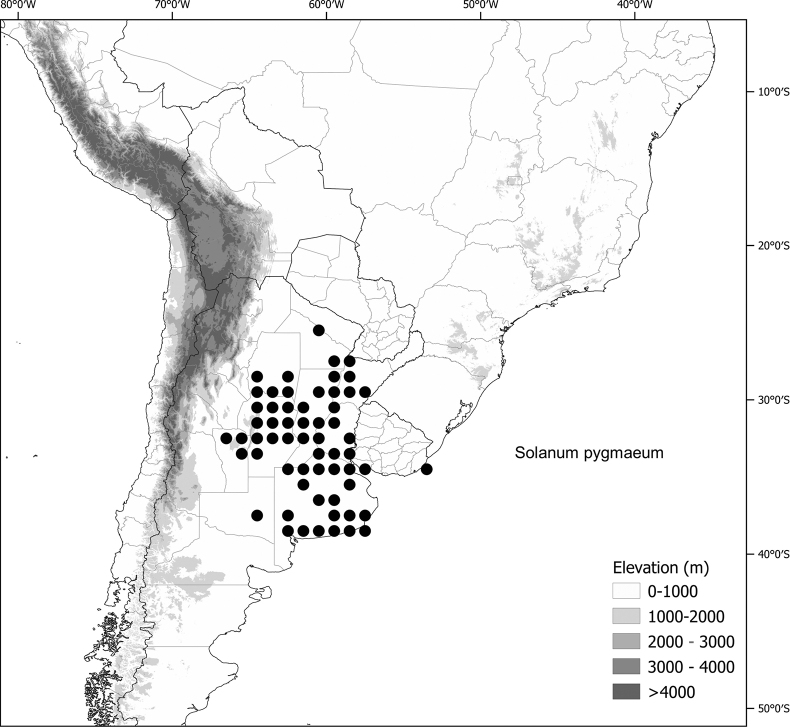
Distribution map of *Solanumpygmaeum*. For adventive distribution, see Särkinen et al. (2018: 133).

**(Fig. 139).***Solanumpygmaeum* is native to central and coastal Argentina (Provs. Buenos Aires, Chaco, Córdoba, Corrientes, Entre Ríos, La Pampa, San Luis, Santa Fé, Santiago del Estero, Tucumán) and Uruguay (Dept. Rocha); it is also adventive in Europe, arriving as seeds through wool shipments but not usually established as permanent populations. A specimen (*Gillies s.n.*, BM) cited in Särkinen et al. (2018) as being from Chile is almost certainly mislabelled and was collected somewhere in Argentina.

##### Ecology and habitat.

*Solanumpygmaeum* in South America grows in dry forests and grassland habitats, usually in sandy and clay soils, along railroad tracks and roadsides; from 100 to 1,000 m.

##### Common names and uses.

None recorded.

##### Preliminary conservation status

**(IUCN 2022).** Least Concern [LC]. EOO = 18,428,537 km^2^ [LC]; AOO = 596 km^2^ [VU]; calculated on the global range. *Solanumpygmaeum* is widespread in its native range and is a patch-forming species of open areas and many different habitat types.

##### Discussion.

*Solanumpygmaeum* is a plant that spreads by underground stems, often forming dense stands of small straggling plants along roads and in grassy vegetation. It is easy to distinguish by its large flowers (anthers more than 3.5 mm long), narrowly elliptic calyx lobes (1.5–1.8 mm long) , and rhizomatous habit. Leaves are quite variable in size, but are usually narrowly elliptic, less often wider in the lower half. It is most similar to *S.rhizomatum* of the Bolivian Andes but differs from that species in its unbranched (versus forked) inflorescences, larger anthers (those of *S.rhizomatum* are less than 3.5 mm long) and berries with 15–25 seeds (versus more than 50 seeds in *S.rhizomatum*). The two species are not sympatric.

Details of typification of the synonyms of *S.pygmaeum* can be found in Särkinen et al. (2018); we have still been unable to trace original material of var.latifolium.

#### ﻿46. Solanum radicans

Taxon classificationPlantaeSolanalesSolanaceae

L.f., Dec. Pl. Horti Upsal. 1. Apr-Jul 1762.

Figs 3D , 140 , 141

Witheringia ruderalis J.Rémy, Fl. Chil. [Gay] 5: 69. 1849. Type. Chile. Región IV (Coquimbo): Coquimbo, *C. Gay 297* (neotype, designated here: P [P00370543]; isolectotype: P [P00370544]).
Solanum ruderale (J.Rémy) F.Phil., Cat. Pl. Vasc. Chil. 229. 1881. Type. Based on Witheringiaruderale J.Rémy.

##### Type.

Cultivated in Uppsala, from Peru, *Anon. s.n.* (lectotype, designated by Knapp in Jarvis 2007, pg. 861: LINN [LINN 248.9]).

**Figure 140.**
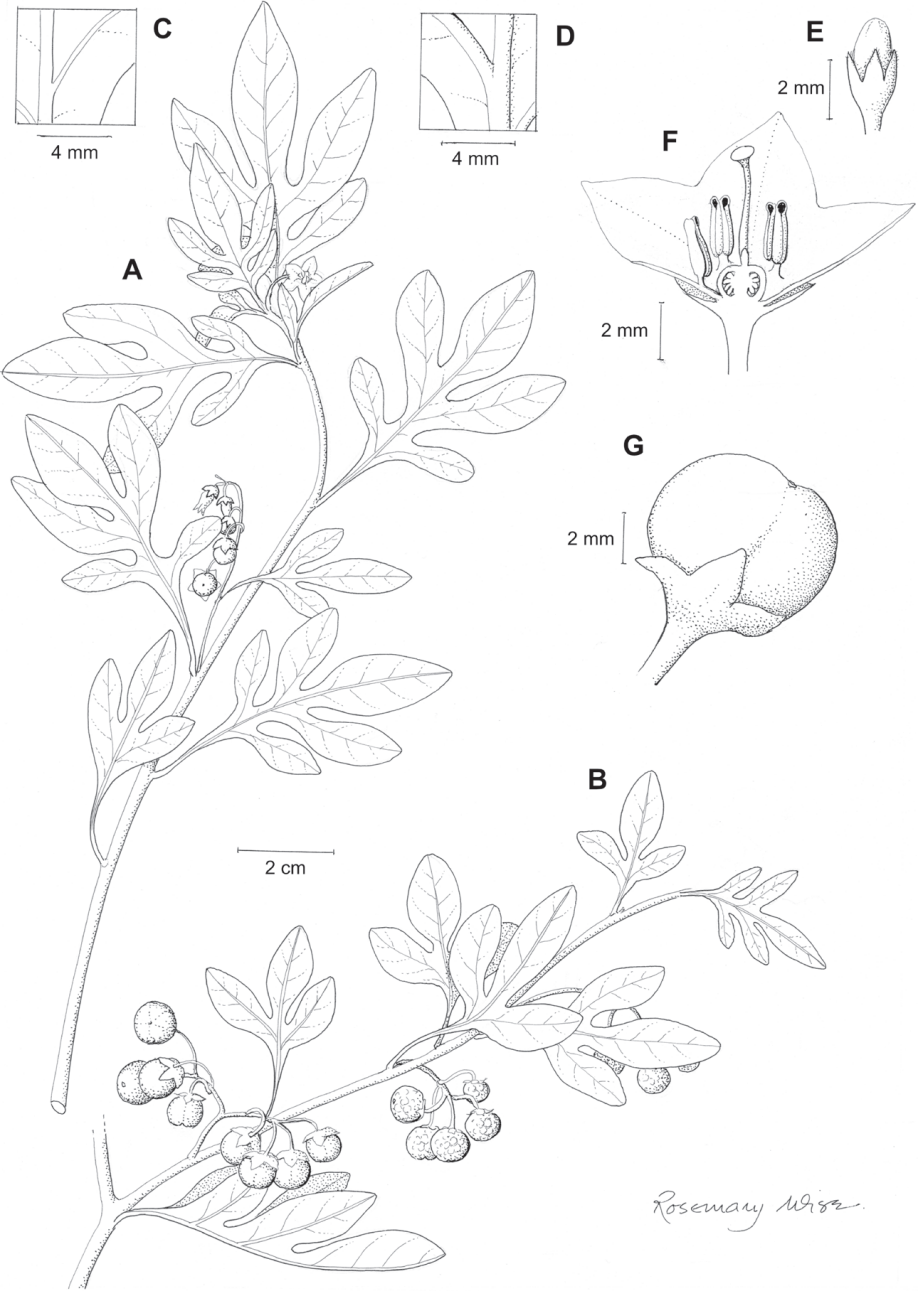
*Solanumradicans***A** flowering habit **B** fruiting habit **C** detail of adaxial leaf surface **D** detail of abaxial leaf surface **E** flower bud **F** dissected flower **G** fruit (**A, D–F***Knapp et al. 10267***B***Knapp et al. 10416*). Illustration by R. Wise.

##### Description.

Creeping herbs to sprawling subshrubs, 0.2–0.75 m high, branches occasionally rooting at the lower nodes. Stems strongly angled to winged from the decurrent leaf bases, with occasional spinescent process along the angles, sparsely pubescent with white eglandular 2–3-celled uniseriate trichomes ca. 0.5 mm long, glabrescent; new growth glabrous to sparsely pubescent with a few scattered white eglandular trichomes like those of the stems, the new leaves densely papillate. Sympodial units difoliate, the leaves not geminate. Leaves simple and deeply 5-lobed, the blades 2.5–14 cm long, 2.5–6 cm wide, elliptic to ovate in outline, widest at the middle or in the lower half, chartaceous, concolorous; adaxial surfaces glabrous or with a few scattered white eglandular simple uniseriate trichomes to 0.5 mm long along the midrib and principal veins, the midrib raised above; abaxial surfaces glabrous or with a few scattered white eglandular simple uniseriate trichomes to 0.5 mm long along the midrib and principal veins; principal veins 2–3 pairs, the midrib raised above; base attenuate onto the stem; margins deeply (3)5-lobed nearly to the midrib, the lobes 3–5 cm long, ca. 2 cm wide, widest in the distal third, asymmetrically elliptic, narrowed near the base, the terminal lobe the largest, occasionally the lateral lobes with minute secondary lobes, the sinuses 3/4 or more of the distance to the midrib, often sparsely ciliate; petiole 0–1 cm long, winged for most of its length. Inflorescences internodal to almost opposite the leaves, usually unbranched, occasionally forked, 2–7 cm long, with 10–20 flowers, sparsely to moderately pubescent with white simple uniseriate trichomes like those of the stems; peduncle 1–3 cm long; pedicels 0.5–1 cm long, 0.4–0.5 mm in diameter at the base, 0.4–0.6 mm in diameter at the apex, with a few scattered white simple trichomes near the base, distally glabrous, articulated at the base; pedicel scars irregularly spaced 1.5–2.5 mm apart. Buds globose, purple-tinged, the corolla halfway to strongly exserted from the calyx before anthesis. Flowers 5-merous, cosexual (hermaphroditic). Calyx tube 1.5–2 mm long, cup-shaped and abruptly narrowing to the pedicel, the lobes 0.75–2 mm long, 0.5–0.9 mm wide, triangular to long-triangular, slightly fleshy, glabrous. Corolla 1–1.2 cm in diameter, rotate-stellate, pale violet to purple, with a greenish yellow central star, lobed ca. halfway to the base, the lobes 2.5–4 mm long, 3–4 mm wide, spreading at anthesis, glabrous adaxially and abaxially, except for the densely papillate lobe tips. Stamens equal; filament tube minute; free portion of the filaments 0.3–0.4 mm long, pubescent with tangled white simple uniseriate trichomes adaxially; anthers 1.5–2 mm long, ca. 1 mm wide, plumply ellipsoid, yellow, poricidal at the tips, the pores lengthening to slits with age. Ovary globose, glabrous; style 4–5 mm long, straight, exserted beyond the anther cone, densely papillate in the lower third; stigma large-capitate and strongly bilobed, the surfaces minutely papillate, bright green in live plants. Fruit a globose to occasionally somewhat flattened-globose berry, 0.5–1 cm in diameter, orange-yellow or slightly greenish yellow when mature, the pericarp thin, matte to slightly shiny, translucent, glabrous; fruiting pedicels 0.6–1 cm long, ca. 1 mm in diameter at the base, ca. 1.5 mm in diameter at the apex, somewhat thickened and woody, deflexed and strongly bent at the base, not persistent; fruiting calyx not markedly accrescent, the tube appressed to the berry, the lobes to 3 mm long, spreading or reflexed in the distal half. Seeds 20–50 per berry, ca. 2 mm long, ca. 1.5 mm wide, teardrop shaped, not markedly flattened, reddish tan or pale tan, the surfaces minutely pitted, the testal cells sinuate in outline. Stone cells 5–6, with 2 larger, 1.2–1.5 mm in diameter and apically positioned, 3–4 smaller, 0.4–0.5 mm in diameter and throughout the berry flesh, all cream-coloured. Chromosome number: 2n = 24 (Chiarini et al. 2017; voucher *Särkinen et al. 4008*).

**Figure 141.**
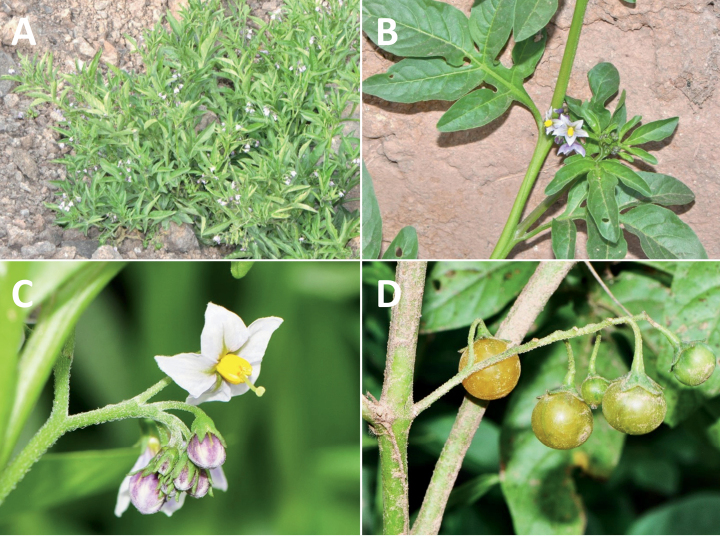
*Solanumradicans***A** habit **B** flowering branch **C** flowers at full anthesis with flower buds **D** maturing fruits (**A***Särkinen et al. 4065***B***Knapp et al. 10417***C***Gonzáles et al. 2877***D***Knapp et al. 10304*). Photos by S. Knapp.

##### Distribution

**Figure 142.**
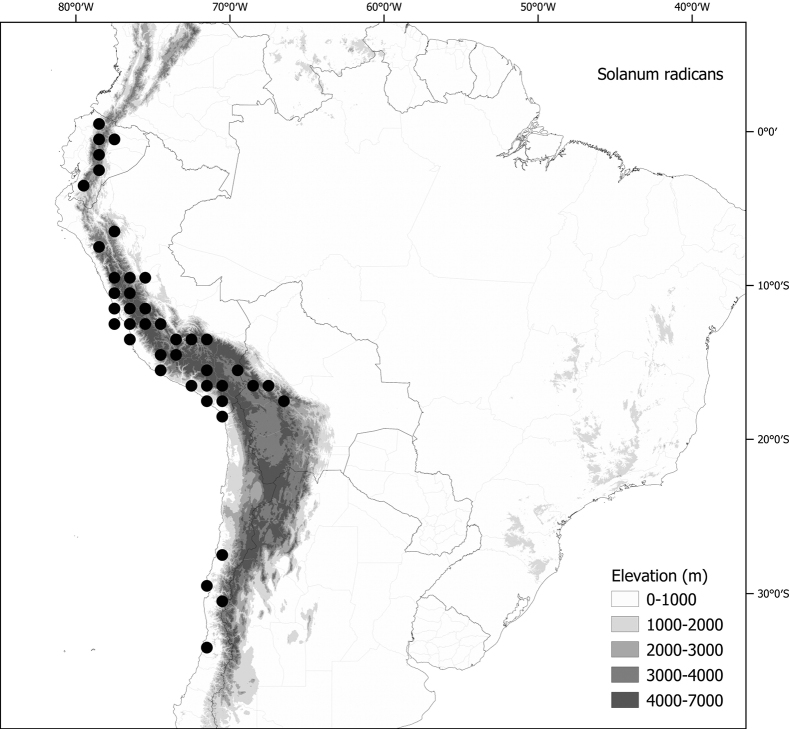
Distribution map of *Solanumradicans*.

**(Fig. 142).***Solanumradicans* occurs from Ecuador (Provs. Carchi, Chimborazo, Cotopaxi, El Oro, Esmeraldas, Imbabura, Pichincha, Tungurahua) and Peru (Depts. Arequipa, Apurímac, Ayacucho, Cajamarca, Cusco, Huancavelica, Huánuco, Ica, Junín, Lima, Moquegua, Piura, Tacna) to Bolivia (Depts. Cochabamba, La Paz) and central Chile (Regions I [Tarapacá], III [Atacama], IV [Coquimbo], XV [Arica y Parinacota]). Most collections are from the western Andean slopes.

##### Ecology and habitat.

*Solanumradicans* is a plant of open places usually at high elevations but can occur to almost sea level in the southern part of its range, from (40-) 1,500 to 3,700 m elevation. It is generally a weedy species and is found in disturbed areas, often associated with human habitation.

##### Common names and uses.

Peru. Arequipa: uva de sapo (*Gonzáles J. 26*); Cusco: cusmayllo (*Valenzuela et al. 6189*); Huánuco: bapichinga (*Woytkowski 738*); Lima: hierba mora (*Espinoza 39*); Moquegua: nucchu (*Blanchard et al. s.n.*), uva de sapo (*Núñez 5*). No uses recorded.

##### Preliminary conservation status

**(IUCN 2022).** Least Concern [LC]. EOO = 2,210,753 km^2^ [LC]; AOO = 484 km^2^ [EN]. *Solanumradicans* is widely distributed and is often found in conjunction with people (in villages and along roadsides and streams). It has been collected around protected archaeological sites in Cusco (Peru).

##### Discussion.

*Solanumradicans* is a member of a small clade (Radicans clade of Särkinen et al. 2015b) also containing the Andean species *S.corymbosum*, *S.palitans* and *S.tripartitum*. Like them, it is a small herbaceous species that often occurs in disturbed areas. It differs from *S.corymbosum* in its 5-parted leaves (versus entire) and orange (versus bright red) berries. From *S.palitans* and *S.tripartitum* it differs in berry colour (orange versus red or yellow), consistently 5-parted leaves (versus 3–4-parted leaves) and usually violet flowers with a prominent globose green stigma. The buds of *S.radicans* are often dark purple, but at anthesis the corolla is usually white (Fig. 3D).

No specimens or herbaria were cited in the protologue of *W.ruderalis* (Rémy 1849), but the publication was largely based on the collections of Claudio Gay. We have selected the one of the two specimens of *Gay 267* at Paris (P00370543) that has a label corresponding to the protologue text as the neotype of this name and combinations based upon it. The other specimen of *Gay 297* at P (P00370544) has no annotations and is clearly a duplicate.

#### ﻿47. Solanum rhizomatum

Taxon classificationPlantaeSolanalesSolanaceae

Särkinen & M.Nee, PhytoKeys 47: 102. 2015.

Figs 143 , 144

##### Type.

Bolivia. Santa Cruz: Prov. Vallegrande, 10 km (by air) NNW of Vallegrande, 18°23'S, 64°08'W, 1,850 m, 1 Feb 1987, *M. Nee 33947* (holotype: LPB; isotypes: CORD [CORD00082080], G, MO [MO-5894880, acc. # 5894880], NY [00824501], US [02836499, acc. # 3146806] and to be distributed).

**Figure 143.**
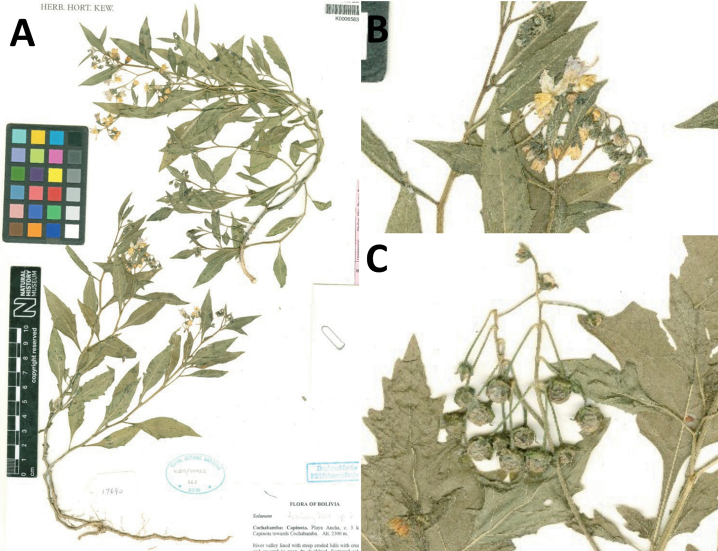
*Solanumrhizomatum***A** habit **B** inflorescence **C** infructescence with maturing fruits (**A, B***Wood 17690* [K000658383]; **C***Wood 11974* [K000658384]). Reproduced with permission of the Trustees of the Royal Botanic Gardens, Kew.

##### Description.

Perennial rhizomatous herbs with erect stems to 0.15–0.2 m high rising from an underground rhizome. Stems 1.5–2 mm in diameter at base, slightly flexuous, terete to ridged, often slightly winged, often purple-coloured, glabrous to sparsely pubescent with appressed 1–4-celled simple uniseriate trichomes ca. 0.5 mm long. Sympodial units difoliate, not geminate. Leaves simple or shallowly toothed or lobed, the blades 2.3–8 cm long, 1.2–4.3 cm wide, ovate-lanceolate, widest in the lower third, membranous, concolorous; adaxial surface glabrous or sparsely pubescent with 1–2-celled spreading hairs along lamina and veins; abaxial surface pubescent only along veins; principal veins 4–6 pairs; base attenuate to decurrent; margins shallowly toothed to entire, often purple-tinged, pubescent with short, 1-celled simple uniseriate trichomes, teeth, if present, most commonly only in the basal 1/3 of the blade; apex acute to acuminate; petiole 0.5–1.2 cm long, sparsely pubescent with spreading, simple uniseriate trichomes like those of the stems and leaves. Inflorescences internodal, forked or several times branched (rarely unbranched), 1.5–3.1 cm long, with 6–15 flowers, sparsely pubescent with simple 1–4-celled uniseriate appressed trichomes; peduncle 1–2.4 cm long and if the inflorescence branched, each branch with a flower-bearing axis 3–4 mm long; pedicels 4–6 mm long, ca. 0.3 mm in diameter at the base and ca. 0.4 mm in diameter at the apex, straight and spreading at anthesis, articulated at the base; pedicel scars spaced 1–2 mm apart. Buds ovoid, white or purple-tinged. Flowers 5-merous, cosexual (hermaphroditic). Calyx tube ca. 2–2.5 mm long, the lobes 1–1.5 mm long, triangular with acute apices, sparsely pubescent with simple 1–3-celled appressed uniseriate trichomes. Corolla 1.2–1.5 cm in diameter, white or flushed blue, with a yellow-green basal star, stellate, lobed halfway to 2/3 of the way to the base, the lobes 4–5 mm long, 2.5–3 mm wide, reflexed at anthesis, later spreading, densely pubescent abaxially with 1–2-celled simple uniseriate trichomes, these usually shorter than the trichomes of stems and leaves. Stamens equal; filament tube 1.2–1.5 mm long; free portion of the filaments 1–1.2 mm long, pubescent along internal side with spreading hairs like those of the stems and leaves; anthers 3.2–3.5 mm long, 0.9–1 mm wide, ellipsoid or rectangular in outline, yellow. Ovary globose, glabrous; style 6–7 mm long, straight, long-exserted beyond the anther cone, densely pubescent with 4-celled simple uniseriate trichomes in the basal 2/3; stigma globose, minutely papillate. Fruit a globose berry, 0.6–0.7 cm in diameter, pale green (mature ?), the pericarp thin, matte, glabrous; fruiting pedicels 1.2–1.4 cm long, ca. 0.6 mm in diameter at the base, ca. 0.8 mm in diameter at the apex, strongly deflexed, not persistent; fruiting calyx lobes 2.5–3.5 mm long, appressed to the berry with the tips slightly reflexed. Seeds 15–25 per berry, 1.7–1.8 mm long, 1.4–1.5 mm wide, tear-drop shaped, pale brown, the hilum positioned towards the narrower end of the seed, the testal cells pentagonal in outline. Stone cells 4–7 per berry, ca. 0.5 mm in diameter, pale tan or creamy white. Chromosome number not known.

**Figure 144.**
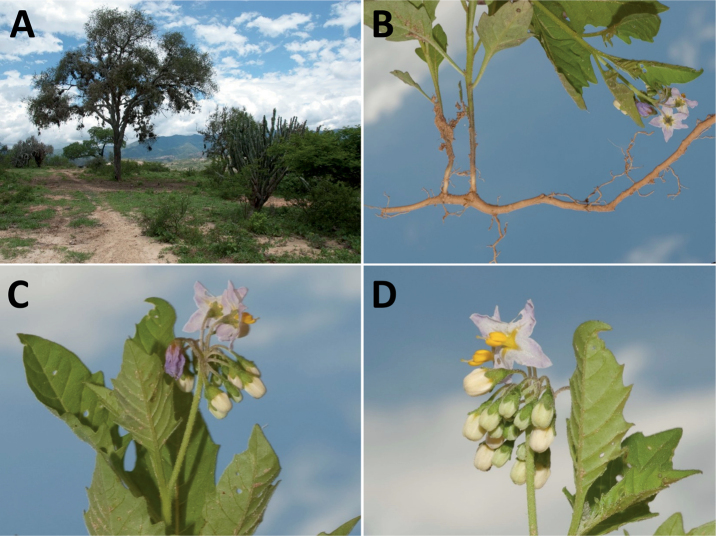
*Solanumrhizomatum***A** habitat of seasonally dry forests **B** rhizome **C, D** inflorescence with flowers at anthesis (**A–D***Nee & Mendoza 57594*). Photos by M. Nee. Previously published in Särkinen et al. (2015d: 105).

##### Distribution

**Figure 145.**
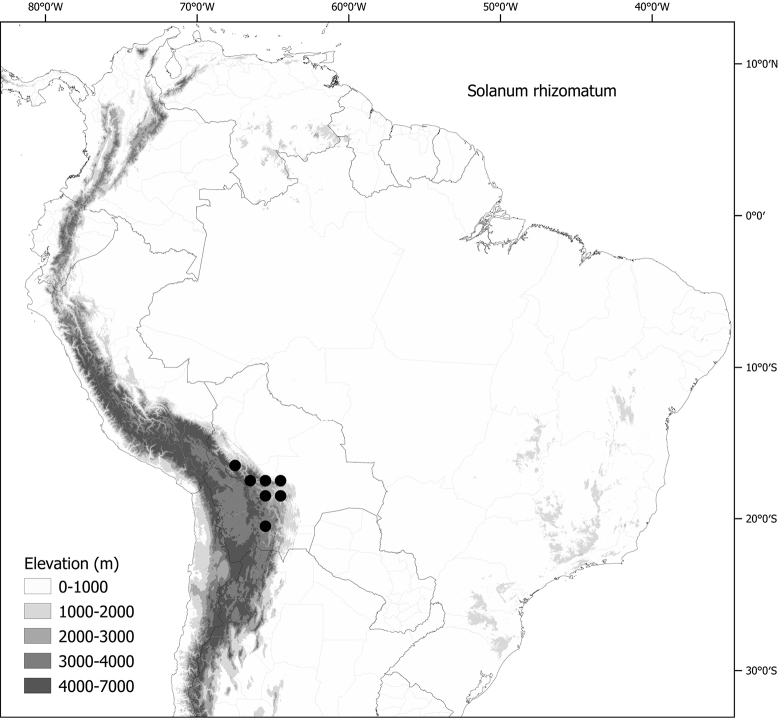
Distribution map of *Solanumrhizomatum*.

**(Fig. 145).***Solanumrhizomatum* is endemic to the arid interior valleys of the Bolivian Andes (Depts. Cochabamba, Potosí, Santa Cruz and also expected to occur in Chuquisaca).

##### Ecology and habitat.

*Solanumrhizomatum* grows in seasonally dry tropical forests and dry matorral vegetation, along slopes and on rocky and sandy soils, found often growing in moist depressions under the shade of larger trees and thickets; between 1,300 and 2,900 m elevation.

##### Common names and uses.

None recorded.

##### Preliminary conservation status.

Least Concern [LC]. EOO = 71,565 km^2^ [LC]; AOO = 80 km^2^ [EN]. Knowing that collection densities in the tropical Andes remain extremely low and considering that current collections of *S.rhizomatum* are from >10 different localities, we suggest this species is not of particular conservation concern. It is not known whether *S.rhizomatum* is similar in its biology and vegetative spread to *S.pygmaeum* and further studies may clarify this aspect for potential conservation assessments in the future. No populations are known thus far from the protected area network in Bolivia. The rhizomatous growth form that allows effective vegetative spreading would indicate that the species can withstand grazing pressures moderately well.

##### Discussion.

*Solanumrhizomatum* is most similar to *S.pygmaeum* from central and littoral Argentina (see Barboza et al. 2013; Särkinen et al. 2015d) and differs from *S.pygmaeum* in having mostly forked (or sometimes more highly branched) inflorescences with 6–15 flowers, anthers 3.2–3.5 mm long, strongly recurving pedicels in fruit, and berries with 15–25 seeds, while *S.pygmaeum* always has simple inflorescences with 2–6 flowers, anthers usually >3.5 mm long, pedicels that are broadly spreading in fruit and berries with > 50 seeds. Although these sets of characters to some extent overlap, *S.pygmaeum* individuals are generally smaller than those of *S.rhizomatum* (10–20 cm high), with smaller leaves 1–5 cm long and 0.5–2.2 cm wide, while *S.rhizomatum* grows 15–50 cm high, with larger leaves 2.3–8 cm long and 1.2–4.3 cm wide.

Like many species of *Solanum*, colour variation of the corolla based on herbarium labels is observed in *S.rhizomatum* where the corolla varies from white to pale lilac, even within individuals. *Nee & Mendoza 57954* note changes in the corolla colour during development, where the *c*orolla is white in bud, violet in anthesis (Fig. 144D), but darker after wilting.

#### ﻿48. Solanum riojense

Taxon classificationPlantaeSolanalesSolanaceae

Bitter, Repert. Spec. Nov. Regni Veg. 11: 481. 1913.

Figs 146 , 147

Solanum andicola C.V.Morton, Revis. Argentine Sp. Solanum 75. 1976. Type. Argentina. Tucumán: Dpto Tafí del Valle, Paso del Infiernillo, 3,000 m, 18 Feb 1924, *S. Venturi 7776* (holotype: US [00027450, acc. # 1549026]).

##### Type.

Argentina. La Rioja: [Dpto. Vinchina], al pie del Peñón, Cordillera de la Rioja, *G. Hieronymus & G. Niederlein 233* (lectotype, designated by Morton 1976, pg. 79: CORD [CORD00004290]).

**Figure 146.**
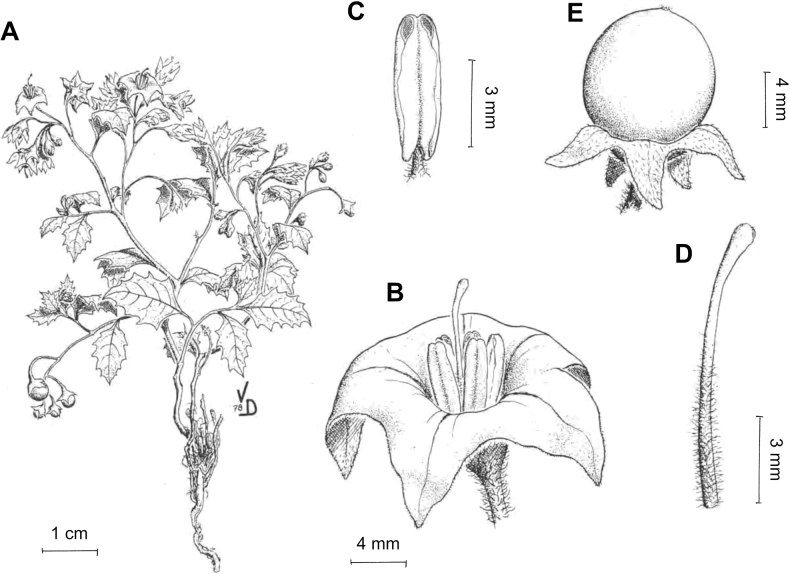
*Solanumriojense***A** habit **B** flower at anthesis **C** stamen **D** style with pubescence in basal half **E** fruit with slightly reflexed calyx lobes (**A–D***Cabrera et al. 21489*). Illustration by V. Dudas. Previously published in Cabrera (1983: 418) and in part in Barboza et al. (2013: 244), as *S.echegarayi*.

##### Description.

Small herbs from a woody rhizomatous base, 0.1–0.2 m high, the branches usually spreading. Stems terete, moderately pubescent with white eglandular 2–6-celled simple uniseriate trichomes to 0.5 mm long, these usually curled and somewhat tangled; new growth moderately to densely pubescent with tangled white eglandular simple uniseriate trichomes like those of the stems, these denser along the veins; bark of older stems glabrescent, pale greenish brown. Sympodial units plurifoliate, the leaves not geminate. Leaves simple and shallowly toothed, the blades 1.8–4.5 cm long, 0.8–2.4 cm wide, elliptic, widest at the middle, somewhat thick and coriaceous or fleshy, concolorous, very variable in size on individual plants; adaxial and abaxial surfaces sparsely and evenly pubescent with curled white eglandular simple uniseriate trichomes like the stems to 0.5 mm long or glabrous with only a few trichomes along the veins; principal veins 3–4(6) pairs, sparsely pubescent on both surfaces; base attenuate; margins shallowly toothed along entire length or less teeth only present near the base, the teeth 3–5 mm long, 4–6 mm wide, with rounded or pointed tips; apex acute or occasionally bluntly rounded; petiole absent to minute (ca. 0.1 mm), sparsely pubescent with curled white eglandular simple uniseriate trichomes ca. 0.5 mm long like those of the stems. Inflorescences terminal, unbranched, 1–2.5(3) cm long, with 5–7 flowers clustered at the tips, sparsely tangled white-pubescent like the stems and leaves; peduncle 0.8–2.3 cm long; pedicels 0.8–1.1 cm long, ca. 0.5 mm in diameter at the base, 2–2.5 mm in diameter at the base, strongly tapering, spreading at anthesis, sparsely pubescent with tangled white eglandular trichomes like those of the stems and rest of the inflorescence, articulated at the base; pedicel scars evenly spaced 1–3 mm apart. Buds broadly ellipsoid, the corolla ca. halfway exserted from the calyx tube before anthesis, the style often emerging from the unopened flowers in bud. Flowers 5-merous, cosexual (hermaphroditic). Calyx tube 1.5–2.5 mm long, conical, the lobes 2.5–3 mm long, broadly deltate with rounded tips, sparsely pubescent with tangled white eglandular trichomes to 0.5 mm long. Corolla 1.8–2 cm in diameter, white or pale lavender adaxially, purple abaxially, with a greenish yellow central star or eye, stellate, lobed halfway to 2/3 of the way to the base, the lobes 5–6 mm long, 3.5–5 mm wide, spreading at anthesis, adaxially glabrous, abaxially pubescent where exposed in bud with curled and tangled white eglandular simple uniseriate trichomes 0.5–0.75 mm long, these denser at tips and margins. Stamens equal; filament tube minute; free portion of the filaments ca. 0.5 mm long, sparsely pubescent with tangled weak simple uniseriate trichomes adaxially; anthers 3.5–4.5 mm long, 1.5–2 mm wide, ellipsoid, yellow, poricidal at the tips, the pores lengthening to slits with age. Ovary conical, glabrous; style 10–11 mm long, straight, exserted beyond the anther cone, moderately pubescent with transparent uniseriate papillae and trichomes in the lower half within the anther tube; stigma capitate and usually somewhat bilobed, the surface minutely papillate. Fruit a globose berry, 0.8–1.1 cm in diameter, mature colour not known, the pericarp thin, matte, opaque, glabrous; fruiting pedicels 1.3–1.5 cm long, ca. 0.5 mm in diameter at the base, ca. 2.5 mm in diameter at the apex, deflexed, somewhat woody, not persistent; fruiting calyx not accrescent, appressed to the berry surface or slightly reflexed, not enlarging from size in flower. Seeds ca. 20 per berry, 2.5–3 mm long, ca. 2.5 mm wide, flattened and teardrop shaped, reddish brown, the surfaces minutely pitted, the testal cells deeply sinuate in outline. Stone cells absent (*Barboza 3253*) or 4–10 per berry, 2–2.5 mm in diameter, pale cream. Chromosome number: n = 12 (Moyetta et al. 2013, voucher *Barboza et al. 3253*, as *S.echegarayi*).

**Figure 147.**
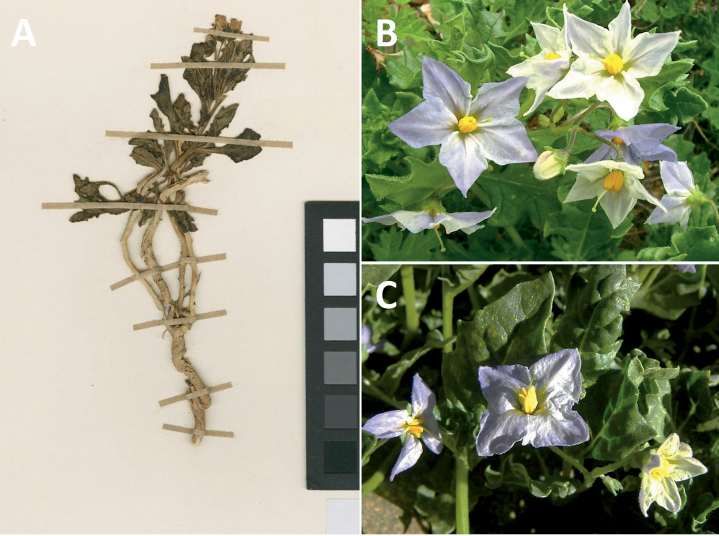
*Solanumriojense***A** habit **B** flowers at full anthesis **C** flower with 4-parted corolla (**A***Hieronymus & Niederlein 233* [CORD00004290], reproduced with permission of the Universidad Nacional de Córdoba **B, C***Barboza 3253*). Photos of live plants by G.E. Barboza.

##### Distribution

**Figure 148.**
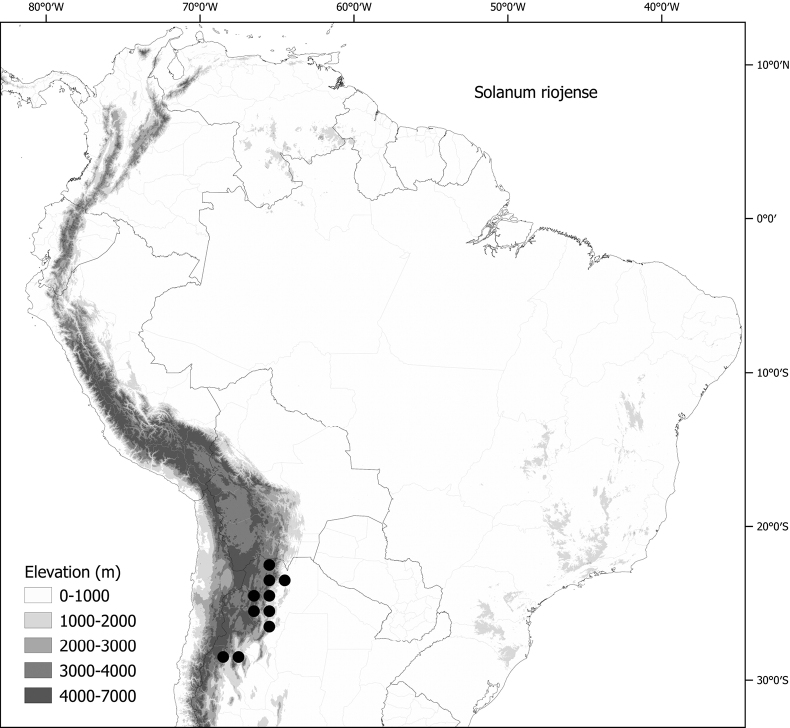
Distribution map of *Solanumriojense*.

**(Fig. 148).***Solanumriojense* is endemic to the Andes of northern Argentina (Provs. Jujuy, La Rioja, Salta, Tucumán).

##### Ecology and habitat.

*Solanumriojense* grows in prepuna and puna vegetation, usually in open rocky areas, often at the edges of fields or in other disturbed areas, from 2,000 to 3,800 m elevation.

##### Common names and uses.

None recorded.

##### Preliminary conservation status

**(IUCN 2022).** Least Concern [LC]. EOO = 128,085 km^2^ [LC]; AOO = 88 km^2^ [EN]. *Solanumriojense* occurs in approximately eight locations along the eastern Andean slope and has a relatively wide distribution. It has been collected in the higher elevations of the Yungas Biosphere Reserve, but most collections are outside of protected areas.

##### Discussion.

*Solanumriojense* is a member of the *Episarcophyllum* clade (Särkinen et al. 2015b), together with *S.echegarayi* and *S.sinuatirecurvum*. The identity of this species has been somewhat obscured by issues over the type specimen (see below), Barboza et al. (2013) placed it in synonymy *with S.echegarayi*, but Cabrera (1983) correctly identified it as distinct. It differs from *S.echegarayi* in the tangled, floccose pubescence of new growth (versus uniform, short erect, trichomes or absence of pubescence in *S.echegarayi*) and the calyx lobes with rounded rather than sharply acute tips. *Solanumriojense* is similar to *S.sinuatirecurvum* in the floccose pubescence of new growth, but differs in having smaller flowers (1.8–2 cm in diameter versus 2–2.6 cm in diameter in *S.sinuatirecurvum*) and berries (0.8–1.1 cm in diameter versus 1–1.3 cm in diameter in *S.sinuatirecurvum*). The rounded calyx lobe apices of *S.riojense* in both flower and fruit differentiate it from *S.sinuatirecurvum* (with sharply pointed calyx lobe tips). Mature fruit colour of *S.riojense* is not known.

A specimen in CORD (CORD00012856) labelled “Echegaray 472”, came to CORD from SI and undoubtably represents a mix-up of labels. The name *Solanumechegarayi* Hieron. was based on an un-numbered collection of Saale Echegaray from Leoncito (San Juan) that lacked pubescence (as noted by A.T. Hunziker on CORD00012858 in pencil); the protologue notes that the plant is completely glabrous and the lectotype of *S.echegarayi* (CORD00004197) matches the protologue. CORD00012856 has tangled white uniseriate trichomes and is therefore in conflict with the protologue.

The specimen CORD00012856 matches perfectly one of the syntypes of *Solanumriojense* Bitter, *Hieronymus & Neiderlein 472* (CORD00004291) a similar small, high elevation species that has tangled white trichomes on all new growth and inflorescences. What appears to have happened is a complex exchange of specimens from CORD to SI, then back to CORD, with the collector and locality being copied in error to this duplicate (and that at SI) of *Hieronymus & Niederlein 472*. Therefore, this sheet (CORD00012856) and the corresponding sheet in SI (003309) should be considered duplicates of *Hieronymus & Niederlein 472*, thus isosyntypes of *Solanumriojense*, not type material of *Solanumechegarayi*.

#### ﻿49. Solanum salamancae

Taxon classificationPlantaeSolanalesSolanaceae

Hunz. & Barboza, Lorentzia 7: 17, fig. 1. 1993.

Figs 149 , 150

##### Type.

Argentina. Salta: Dpto. Guachipas: La Salamanca, viniendo desde San Carlos, rumbo a La Vina, La Salamanca, *A.T. Hunziker & R. Subils 24049* (holotype: CORD [CORD00004293]; isotypes: CORD [CORD00004292], MA [MA771370]).

**Figure 149.**
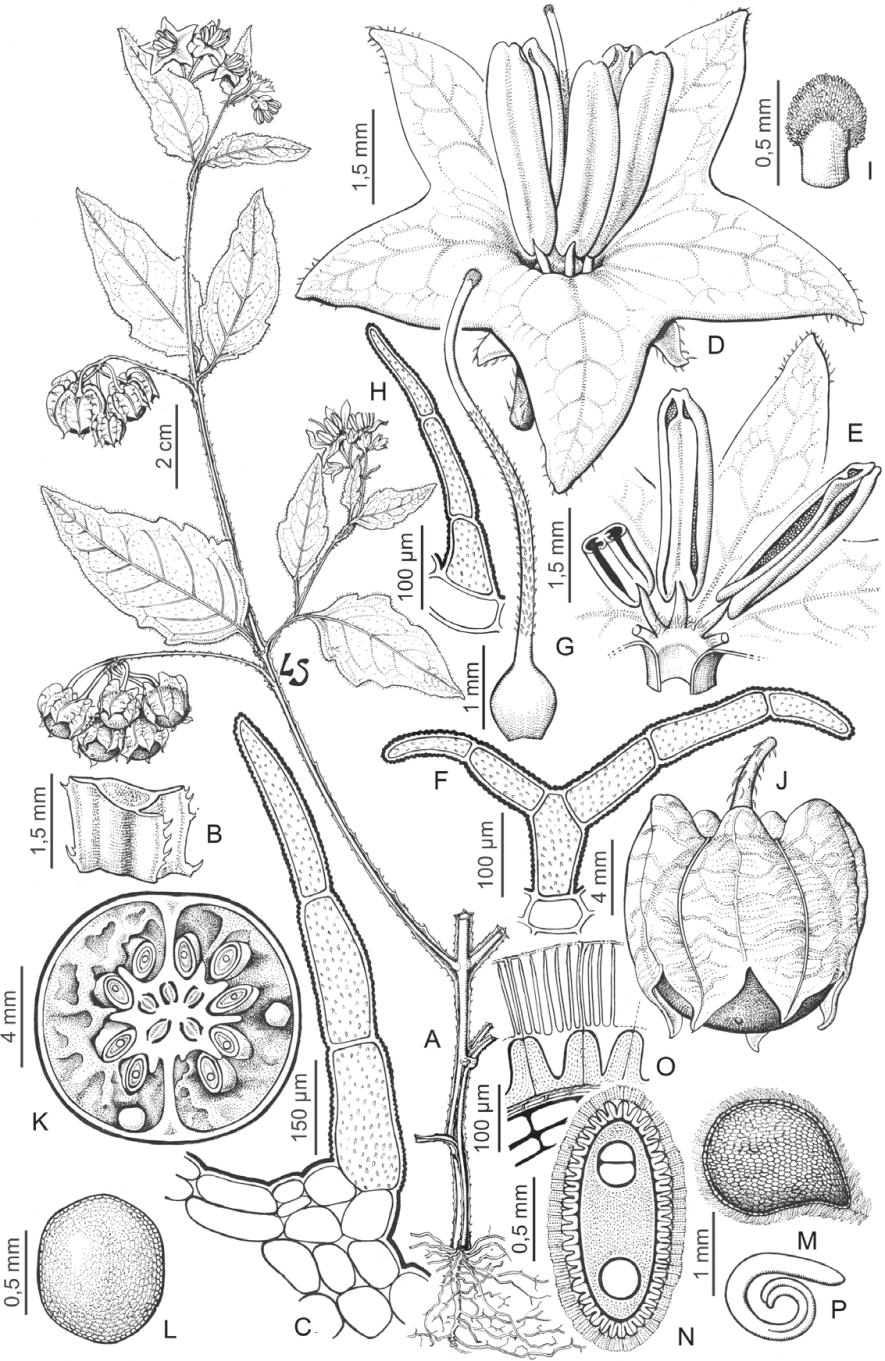
*Solanumsalamancae***A** habit **B** stem cross section **C** spinose process with an eglandular trichome **D** flower **E** dissected flower **F** forked trichome of the filament **G** gynoecium **H** eglandular trichome of the style **I** stigma **J** fruit **K** fruit cross section **L** stone cell **M** seed **N** seed cross section **O** detail of the episperm **P** embryo (**A–P***Hunziker & Subils 24049*). Illustration by L. Sánchez. Previously published in Barboza et al. (2013: 257).

##### Description.

Annual herbs 0.2–0.7 m high, often spreading and sprawling. Stems somewhat winged or with prominent spinose processes from the remnant, stiffened bases of trichomes, sparsely to moderately pubescent with eglandular white 6–8-celled simple uniseriate trichomes 0.5–2(–3.5) mm long, these usually spreading and tangled, but sometimes antrorse (collections from Metán, Salta with extremely long trichomes); new growth sparsely to densely pubescent with eglandular, 6–8-celled simple uniseriate trichomes 0.5–2 mm long; bark of older stems pale yellow, glabrescent, but spinose processes persistent. Sympodial units difoliate, the leaves more or less geminate, if geminate then more or less equal in size and shape. Leaves simple, entire or somewhat toothed, the blades (3)3.5–11 cm long, 2–7 cm wide narrowly ovate to narrowly elliptic, widest in the lower third, membranous, concolorous; adaxial surfaces almost glabrous to sparsely pubescent with eglandular, simple uniseriate trichomes to 1 mm long, these mostly on the veins; abaxial surfaces similarly almost glabrous to sparsely pubescent, but the trichomes denser along the midvein; principal veins 6–7 pairs, usually sparsely pubescent with eglandular, white, simple uniseriate trichomes; base truncate-attenuate to attenuate and decurrent along the petiole; margins entire or irregularly and shallowly toothed in the lower third of the blade or along the entire margin, the teeth 1–3 mm long; apex acute to acuminate; petiole (0.5)1–1.5(–2.6) cm long including the winged portion of the leaf base, pubescent with eglandular white simple uniseriate trichomes like those of the leaf surfaces. Inflorescences internodal, arising just below the geminate leaf pair, unbranched, 1.5–7.5 cm long, with 5–10 flowers in the distal third, these somewhat secund, sparsely pubescent with spreading, eglandular, simple uniseriate trichomes to 2 mm long like those of the stems; peduncle 1–4 cm long; pedicels 0.7–1 cm long at anthesis, ca. 0.5 mm in diameter at the base, ca. 0.5 mm in diameter at the apex, filiform and slightly widening at the base of the calyx tube, secund to somewhat spreading at anthesis, sparsely pubescent with simple uniseriate trichomes like the rest of the inflorescence, articulated near the base, leaving a distinct stump; pedicel scars evenly spaced 1–1.5 mm apart. Buds narrowly ellipsoid to ellipsoid, the corolla strongly exserted from the calyx tube before anthesis. Flowers 5-merous, cosexual (hermaphroditic). Calyx tube 1–1.5 mm long, conical, the lobes 1.2–3 mm long, long-triangular with acuminate tips, sparsely pubescent with eglandular, white simple uniseriate trichomes to 2 mm long like the rest of the inflorescence. Corolla ca. 1.5 cm in diameter, white with a pale green central star, this sometimes with purple margins, stellate, lobed ca. halfway to the base, the lobes 4.5–5 mm long, 2–2.5 mm wide, spreading to somewhat reflexed at anthesis, adaxially glabrous, abaxially with scattered eglandular, simple uniseriate trichomes ca. 0.5 mm long on the tips and midvein. Stamens equal; filament tube 0.1–0.5 mm long; free portion of the filaments 1–1.5 mm long, pubescent with translucent tangled simple uniseriate trichomes abaxially; anthers 3.5–5 mm long, 1–1.2 mm wide, ellipsoid, yellow, poricidal at the tips, the pores lengthening to slits with age. Ovary conical, glabrous; style 6–7 mm long, straight, exserted beyond the anther cone, densely pubescent in the lower 2/3 (within the anther cone) with unicellular papillae and tangled unicellular trichomes; stigma large-capitate, green in live plants, the surface minutely papillate. Flowers 5-merous, cosexual (hermaphroditic). Calyx tube 1–1.5 mm long, conical, the lobes 1.2–3 mm long, long-triangular with acuminate tips, sparsely pubescent with eglandular, white simple uniseriate trichomes to 2 mm long like the rest of the inflorescence. Corolla ca. 1.5 cm in diameter, white with a pale green central star, this sometimes with purple margins, stellate, lobed ca. halfway to the base, the lobes 4.5–5 mm long, 2–2.5 mm wide, spreading to somewhat reflexed at anthesis, adaxially glabrous, abaxially with scattered eglandular, simple uniseriate trichomes ca. 0.5 mm long on the tips and midvein. Stamens equal; filament tube 0.1–0.5 mm long; free portion of the filaments 1–1.5 mm long, pubescent with translucent tangled simple uniseriate trichomes abaxially; anthers 3.5–5 mm long, 1–1.2 mm wide, ellipsoid, yellow, poricidal at the tips, the pores lengthening to slits with age. Ovary conical, glabrous; style 6–7 mm long, straight, exserted beyond the anther cone, densely pubescent in the lower 2/3 (within the anther cone) with unicellular papillae and tangled unicellular trichomes; stigma large-capitate, green in live plants, the surface minutely papillate. Fruit a globose berry, 0.7–0.9 cm in diameter, green when mature, enclosed in the inflated calyx but the tip of the berry visible at fruit maturity, the pericarp thin, shiny, translucent, glabrous; fruiting pedicels 1.2–1.4 cm long, 0.5–0.7 mm in diameter at the base, strongly hooked at insertion point onto the inflorescence axis, 2–2.5 mm in diameter at the apex just below inflated calyx, not persistent; fruiting calyx accrescent and inflated, invaginate (saccate) at the base, the tube to 1 cm long, almost completely covering berry, the lobes ca. 3 mm long, ca. 3 mm wide, broadly triangular, apiculate. Seeds 20–40 per berry, 2–2.5 mm long, 1.5–2 m wide, flattened and teardrop shaped, brown or dark brown, the surfaces minutely pitted, the testal cells rectangular or slightly sinuate in outline. Stone cells 2–4 per berry, ca. 0.7 mm in diameter, cream-coloured. Chromosome number: not known.

**Figure 150.**
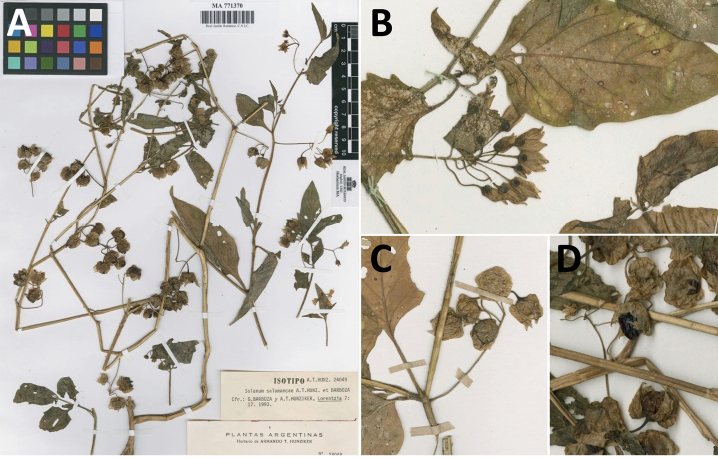
*Solanumsalamancae***A** habit **B** fruiting branch with developing fruits with large calyx lobes **C** fruiting branch with mature fruits **D** mature fruits inside inflated calyces (**A***Hunziker & Subils 24049* [MA771370] **B***Novara & Bruno 9637* [S-R-9196] **C***Novara & Bruno 9637* [CORD00004294] **D***Hunziker & Subils 24049* [CORD00004292]). Reproduced with permission of the Real Jardín Botánico de Madrid, Swedish Museum of Natural History and the Universidad Nacional de Córdoba.

##### Distribution

**Figure 151.**
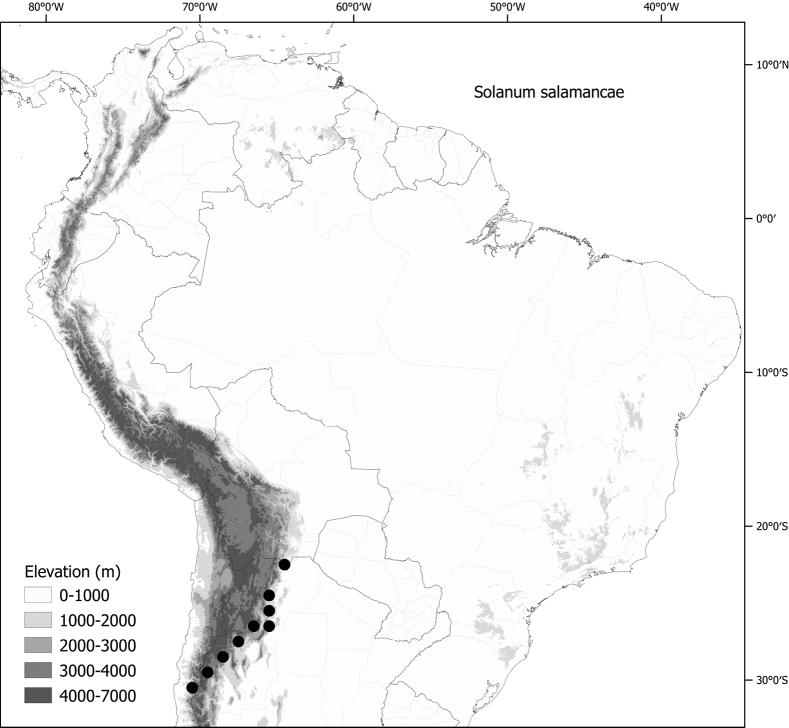
Distribution map of *Solanumsalamancae*.

**(Fig. 151).***Solanumsalamancae* is endemic to Argentina (Provs. Catamarca, Salta, Tucumán).

##### Ecology and habitat.

*Solanumsalamancae* grows in dry forests (“chaco serrano”), in the transition zone between forest and prepuna, often at seasonal stream margins in sandy soils or at field edges, from 1,200 to 3,000 m elevation.

**Common names and uses**. None recorded.

##### Preliminary conservation status

**(IUCN 2022).** Least Concern [LC]. EOO = 79,244 km^2^ [LC]; AOO = 84 km^2^ [EN]. *Solanumsalamancae* occurs all along the Andean slope in northern Argentina and is a weedy species, but because it grows in a narrow, transitional habitat, future studies might reveal habitat specialisation. Many recent collections are from areas around active mining operations (e.g., *Tolaba & Gutiérrez 4240*) and so these populations may be at risk.

##### Discussion.

*Solanumsalamancae* is a distinctive species with winged or strongly spinescent stems, lacking glandular pubescence and with accrescent, inflated calyces with invaginate bases that completely enclose the berry. Other taxa with similarly accrescent calyces (*S.hunzikeri*, *S.nitidibaccatum*, *S.physalidicalyx*, *S.sarrachoides*, *S.tweedieanum*) are densely viscid-glandular pubescent. The strongly inflated calyces of *S.salamancae* are most similar to those of *S.physalidicalyx*; accrescent calyces of these similar species only partially cover the berry or are tightly appressed to it (e.g., *S.tweedieanum*).

Populations of *S.salamancae* from the region of Metán (Prov. Salta) are consistently more long-pubescent than in other areas of the species range (e.g., *Tolaba & Gutiérrez 4238* and others collected around the same area) and the trichomes are often strongly antrorse.

#### ﻿50. Solanum salicifolium

Taxon classificationPlantaeSolanalesSolanaceae

Phil., Anal. Univ. Chile 36: 195. 1870.

**Figure 152.**
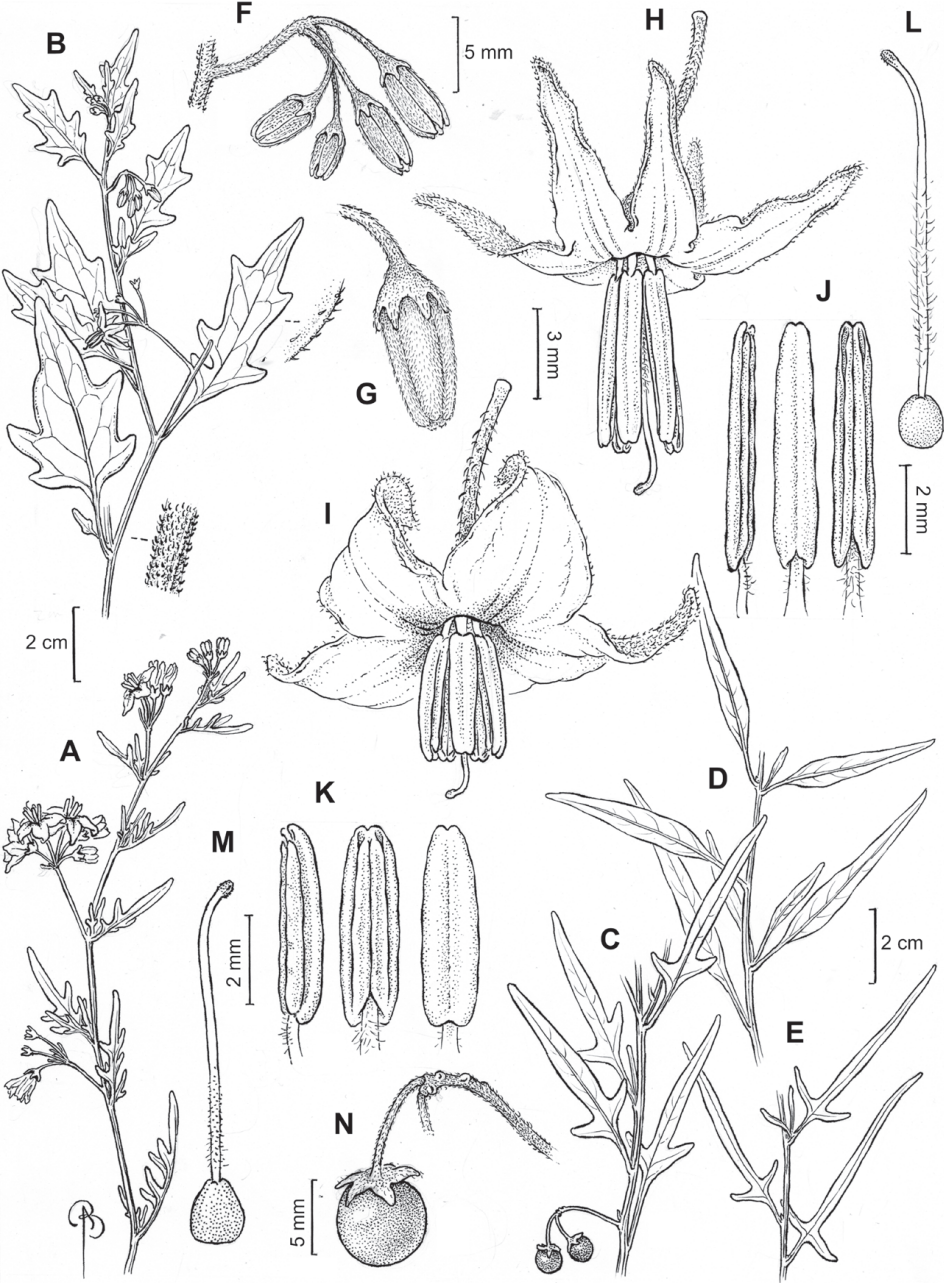
*Solanumsalicifolium***A, B** flowering habit **C** fruiting habit **D, E** sterile habit showing leaf shape variation **F** inflorescence in bud **G** flower bud **H, I** flower **J, K** stamens **L, M** gynoecium **N** maturing fruit (**A, I, K, M***Kiesling et al. 7929***B, F–H, J, L***Varela 633***C, N***Kuntze s.n*., Dec 1891 **D***King 151***E***Hieronymus s.n.*, collected in 1878). Illustration by B. Angell. Previously published in Knapp (2013: 240).

**Figure 153.**
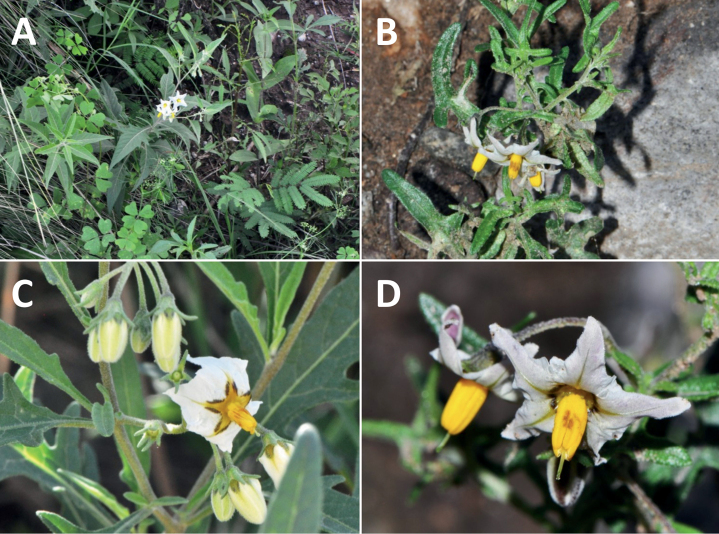
*Solanumsalicifolium***A** habit with less divided leaves **B** habit with more divided leaves **C** flowers and buds **D** flowers at anthesis (**A***Barboza et al. 3465***B, D***Barboza et al. 3494***C***Barboza et al. 3473*). Photos S. Knapp.

Figs 2H , 3F , 152 , 153

Solanum incisum Griseb., Abh. Königl. Ges. Wiss. Göttingen 24: 251. 1879. Type. Argentina. Córdoba: Sierra de Achala, 24–25 Mar 1874, *G. Hieronymus 220* (lectotype, designated by Morton 1976, pg. 99: GOET [GOET003582]; isolectotypes: B, destroyed [F neg. 2779], CORD [CORD00006112]).
Solanum sericeum Ruiz & Pav. var. strigillosum Griseb., Abh. Königl. Ges. Wiss. Göttingen 24: 252. 1879. Type. Argentina. Córdoba: Dpto. Las Minas, Cerro de Orcosu [Achala in protologue], 20 Feb 1876, *G. Hieronymus 812* (holotype: GOET [GOET003580]; isotypes: CORD [CORD00006115], US [00027795, acc. # 2678278]).
Solanum tenuisectum Kuntze, Revis. Gen. Pl. 3(2): 227. 1898. Type. Argentina. “western Pampas, 34 degrees”, Jan 1892, *O. Kuntze s.n.* (lectotype, designated by Knapp 2013, pg. 238: NY [00172207]; isolectotype: NY [00172206]).
Solanum incisum Griseb. var. septatopilosum C.V. Morton, Revis. Argentine Sp. Solanum 100. 1976. Type. Argentina. Catamarca: Dpto. Belén, Pozo de Piedra, 1,900 m, 25–31 Jan 1952, *H. Sleumer & F. Vervoorst 2375* (holotype: US [01049780, acc. #2173088]; isotype: LIL).
Solanum crebrum C.V.Morton & L.B.Sm., Revis. Argentine Sp. Solanum 80. 1976. Type. Argentina. Catamarca: Dpto. Andalgalá, Alto de las Juntas y alrededores, 1–16 Jan 1952, 2,700–2,830 m, *H. Sleumer 2166* (holotype: US [00027530, acc. # 2168362]; isotypes: CORD [CORD00012840], G [G00357861], LIL [LIL-394778]).
Solanum incisum Griseb. var. tenuisectum (Kuntze) C.V. Morton, Revis. Argentine Sp. Solanum 100. 1976. Type. Based on Solanumtenuisectum Kuntze.
Solanum vervoorstii C.V.Morton, Revis. Argentine Sp. Solanum 128. 1976. Type. Argentina. Catamarca: Dpto. Belén, Quebrada de los Potrerillos above El Rodeo, Granadillas, 26 Jan 1952, 2,700–2,830 m, *H. Sleumer & F. Vervoorst 2481* (holotype: US [00027846, acc. # 2168145]; isotypes: G, LIL [acc. # 394789]).
Solanum restrictum C.V.Morton, Revis. Argentine Sp. Solanum 128. 1976. Type. Argentina. Córdoba: Dpto. Punilla, Estancia El Rosario, east of La Cumbre, Sierra de Córdoba, 20 Mar 1943, *H.H. Bartlett 20171* (holotype: US [00027775, acc. # 2320061]).
Solanum ratum C.V.Morton, Revis. Argentine Sp. Solanum 130. 1976. Type. Argentina. Córdoba: Dpto. Punilla, El Durazno, 18 Mar 1944, *C.A. O’Donell & J.M. Rodriguez V. 805* (holotype: A [00077745]; isotype: LIL [acc. # 97232]).

##### Type.

Argentina. Mendoza: Villavicencio, *R.A. Philippi s.n.* (lectotype, designated by Knapp 2013, pg. 238: SI [acc. # 26577]; isolectotypes: G [G00070190, F neg. 23156], SGO [SGO acc. # 42739, acc. # 55501], W [acc. # 0001341]).

##### Description.

Suffrutescent herbs to small shrubs, 0.5–1.5 m high, arising from a woody rootstock. Stems slightly angled when young, sparsely to densely pubescent with simple uniseriate trichomes to 0.5 mm long, these strongly antrorse and all appressed to stem, occasionally (collections from Famatina in La Rioja Province, Argentina) more floccose, the trichome base enlarged and slightly bulbous; new growth glabrous or densely pubescent with simple white trichomes like those of the stems. Bark of older stems yellowish grey, glabrescent. Sympodial units difoliate to plurifoliate, if difoliate, the leaves not geminate. Leaves simple to variably pinnatifid, the blades 2.5–10 cm long, 1–7 cm wide, more or less lanceolate to narrowly elliptic in outline, widest at the middle, membranous to chartaceous, concolorous or slightly discolorous; adaxial surfaces glabrous or with scattered simple uniseriate trichomes at the base and along the veins, these all appressed and pointing distally; abaxial surfaces glabrous to uniformly pubescent with appressed and ascending simple uniseriate trichomes < 0.2 mm long; principal veins 10–20 pairs, drying yellowish grey; base attenuate, winged along the stem; margins entire to 3–5-lobed, the lobes 0.5–3.5 cm long, 0.2–0.7 cm wide, incised to the midrib or very shallowly, in the basal part of the leaf; apex acute to acuminate; petioles very short to apparently absent, sparsely pubescent with ascending appressed trichomes on all surfaces like those of the stems. Inflorescences internodal, occasionally opposite the leaves, unbranched or forked, 1–2.5 cm long, with 4–10 flowers in a pseudoumbel, glabrous to pubescent with ascending appressed simple uniseriate trichomes like those of the stems and leaves; peduncle 1–2.2 cm long; pedicels 0.7–1.2 cm long, filiform, ca. 0.5 mm in diameter, nodding at anthesis, pubescent like the rest of the inflorescence, articulated at the base in a very small sleeve; pedicel scars tightly packed at the tip of the inflorescence on a small platform. Buds ellipsoid to fusiform and elongate, the corolla strongly exserted from the calyx tube before anthesis. Flowers 5-merous, cosexual (hermaphroditic). Calyx tube 1–1.5 mm long, conical, the lobes 2.5–3 mm long, long-triangular to lanceolate, glabrous to pubescent with appressed white simple trichomes like those of the stems and leaves. Corolla 1–1.6 cm in diameter, violet or white, often with a green or yellowish green eye, stellate, lobed nearly to the base, the lobes 6–8 mm long, 3–4 mm wide, strongly reflexed at anthesis, densely and uniformly pubescent abaxially with minute simple uniseriate trichomes < 0.1 mm long, glabrous adaxially. Stamens equal; filament tube ca. 0.5 mm long; free portion of the filaments 0.5–1 mm long, densely pubescent adaxially with tangled simple trichomes 0.5–1 mm long; anthers (3–)5–5.5 mm long, ca. 1 mm wide, ellipsoid, loosely connivent to occasionally somewhat spreading, poricidal at the tips, the pores lengthening to slits with age. Ovary glabrous; style 9–11 mm long, straight, exserted beyond the anther cone, glabrous or pubescent with weak simple trichomes in the basal 2/3; stigma capitate, the surface minutely papillose. Fruit a globose berry, 0.5–0.7 cm in diameter, purple or reddish purple when ripe, the pericarp thin and somewhat shiny, opaque, glabrous; fruiting pedicels 1–1.5 cm long, ca. 1 mm in diameter at base and apex, not particularly woody, pendent from weight of fruit, not persistent. Seeds 20–50 per berry, ca. 1.5 mm long, ca. 1.5 mm wide, flattened reniform, yellowish brown, the surfaces minutely pitted, the testal cells rectangular. Stone cells ca. 10 per berry, 0.7–1 mm in diameter. Chromosome number: n = 12 (Moscone 1992, vouchers *Ambrosetti & Moscone 1477*, *Del Vitto & Moscone 852*; *Hunziker et al. 24876*, *24882*, *25043*, *Moscone et al. 91*, *92* as *S.incisum*; Moyetta et al. 2013, vouchers *Barboza et al. 3488*, *3158*, *Chiarini et al. 818*, *794*).

##### Distribution

**Figure 154.**
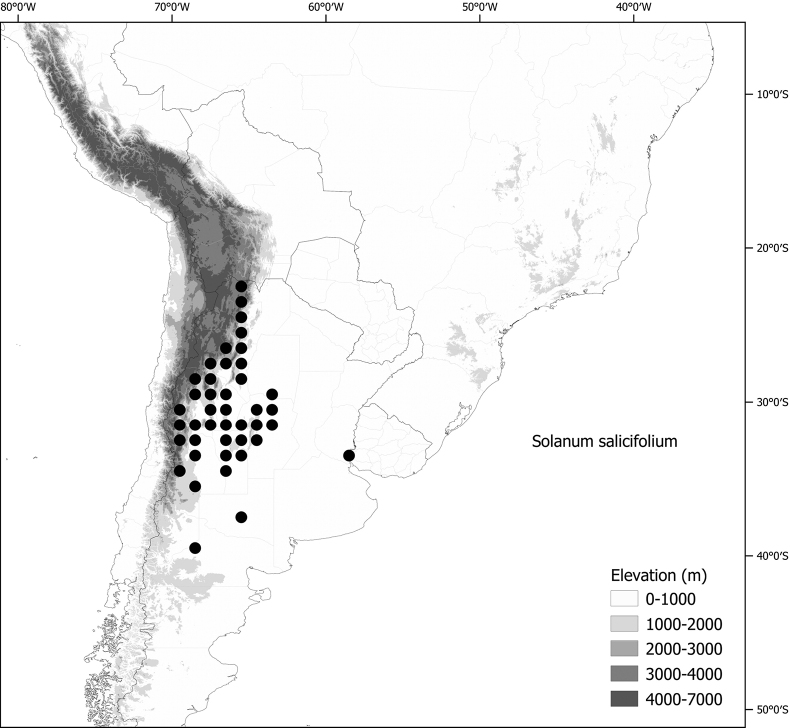
Distribution map of *Solanumsalicifolium*.

**(Fig. 154).***Solanumsalicifolium* occurs on the eastern slopes and foothills of the Andes in western Argentina (Provs. Catamarca, Córdoba, Entre Ríos, Jujuy, La Pampa, La Rioja, Mendoza, Salta, San Juan, San Luis, Tucumán) and Paraguay (Dept. Presidente Hayes). Nineteenth century collections labelled as being collected in “Chile” are almost certainly from adjacent Argentina near Mendoza (e.g., *Cuming s.n.*, *Gillies 32*, *Née s.n.*).

##### Ecology and habitat.

*Solanumsalicifolium* occupies a wide range of dry forested and open habitats, from Chaco woodlands to puna areas above treeline, often growing amongst rocks in grazed areas or on roadsides, from 600 to 4,100 m elevation.

##### Common names and uses.

Argentina. Córdoba: yerba mora (*Kurtz 8324*). No uses recorded.

##### Preliminary conservation status

**(IUCN 2022).** Least Concern [LC]. EOO = 1,063,580 km^2^ [LC]; AOO = 896 km^2^ [VU]. *Solanumsalicifolium* is common where it occurs, is widely distributed geographically and occurs in a number of different habitats. It is found in protected areas in the Argentine Provinces of Mendoza (e.g., Puente del Inca, Parque Provincial Aconcagua) and Córdoba (e.g., Pampa de Achala).

##### Discussion.

Knapp (2013) treated *S.salicifolium* as a member of the Dulcamaroid clade due to its possession of a small pedicel sleeve (swollen insertion point), a characteristic of the dulcamaroids. Molecular sequence data, however, show that *S.salicifolium* belongs to the Morelloid clade (Särkinen et al. 2015b; Gagnon et al. 2022). Possession of stone cells in the berries, noted as unusual in the dulcamaroids by Knapp (2013) also points to its morelloid affinities. *Solanumsalicifolium* is extremely (almost incredibly) variable in leaf shape (see Figs 2H, 152D, E, 153 A, B), ranging from simple and linear (the type of *S.salicifolium*) to deeply pinnatifid with very narrow lobes (the type of *S.tenuisectum*). This is an extreme of variation in leaf shape and has led to considerable confusion over the identity and synonymy of this species. Morton (1976) suggested that leaves with a single pair of lobes at the base might be a late season growth form, but we have seen all leaf shapes on a single plant.

The pinnatifid leaves of *S.salicifolium* are somewhat morphologically similar to those of members of the Radicans clade (*S.corymbosum*, *S.palitans*, *S.radicans* and*S.tripartitum*). These taxa have pedicels that are flush with the inflorescence axis, rather than inserted into a small sleeve, and the flowers are spaced along the inflorescence axis rather than being clustered at the tip on a small platform. The flowers of *S.salicifolium* are much larger than those of members of the Radicans clade, with anthers 3–5.5 mm long versus 1–2 mm long.

For details of typification of the many synonyms of *S.salicifolium* see Knapp (2013).

#### ﻿51. Solanum sarrachoides

Taxon classificationPlantaeSolanalesSolanaceae

Sendtn., Fl. Bras. (Martius) 10: 18, tab. 1, figs 1–8. 1846.

**Figure 155.**
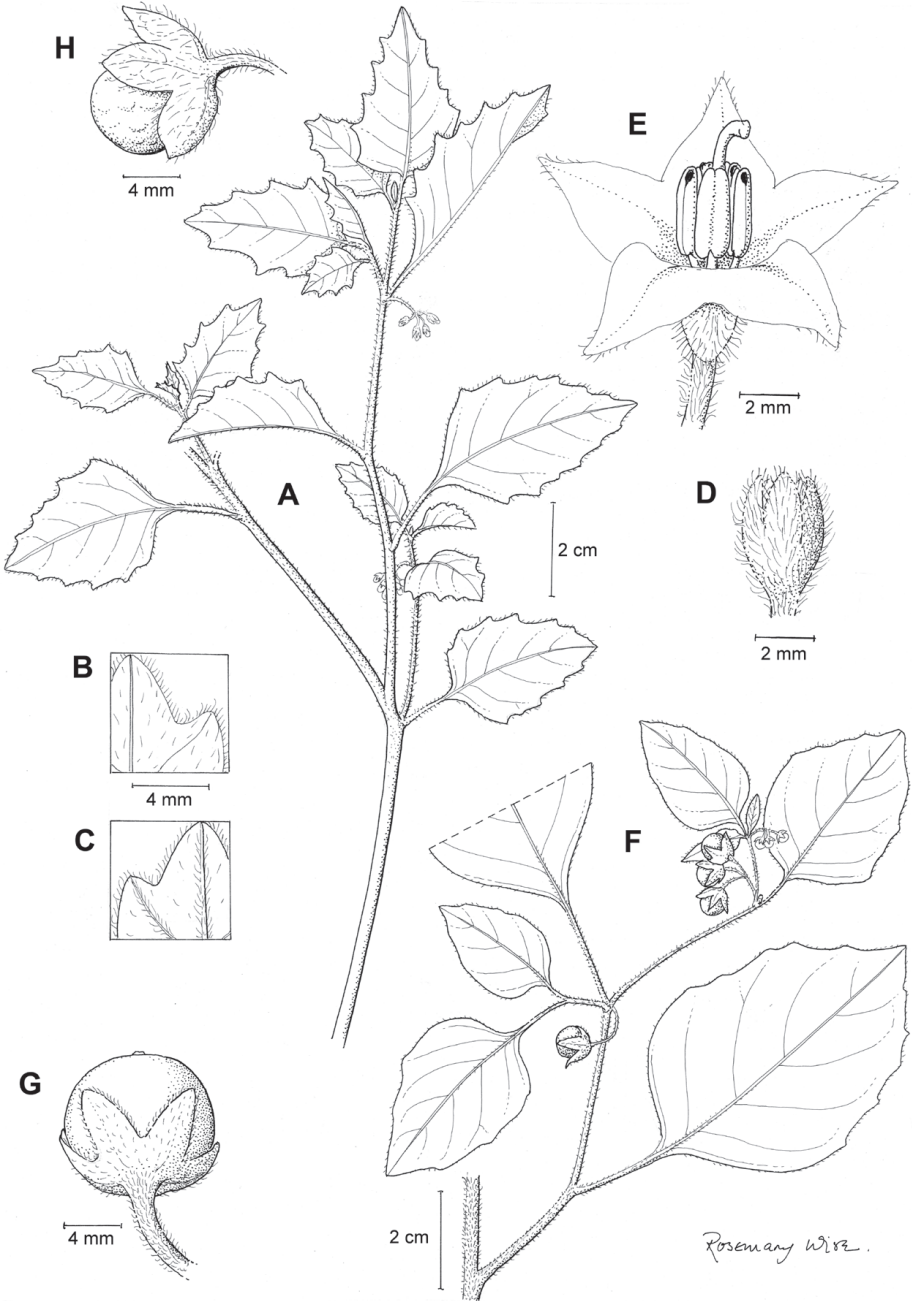
*Solanumsarrachoides***A** habit **B** detail of adaxial leaf surface **C** detail of abaxial leaf surface **D** bud **E** flower **F** fruiting habit **G** maturing fruit (**A–E***Macoun s.n.***F, G***Ahles 55038*). Illustration by R. Wise. Previously published in Särkinen et al. (2018: 142) and Knapp et al. (2019: 108).

**Figure 156.**
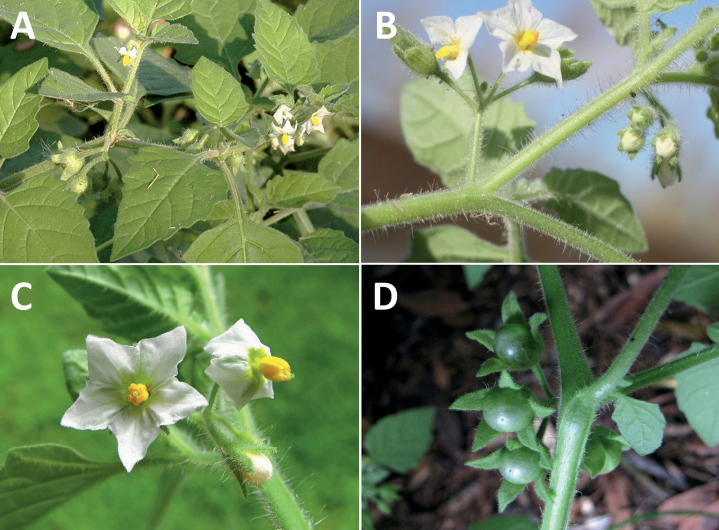
*Solanumsarrachoides***A** habit **B** inflorescence **C** flowers at full anthesis **D** developing fruits (unvouchered). Photos by D.G. Smith, S. Martín de la Vega, and B.W. Wells Association. Previously published in Särkinen et al. (2018: 143) and Knapp et al. (2019: 109).

Figs 155 , 156 Types based on American specimens only; for full synonymy, see Särkinen et al. (2018: 141)

Solanum sarachidium Bitter, Repert. Spec. Nov. Regni Veg. 11: 211. 1912. Type. Paraguay. Gran Chaco: Loma Clavel, Nov 1903, *T. Rojas 2493* (lectotype, designated by Edmonds 1986, pg. 17: BM [BM000087577]; isolectotype: G [G00306752]).
Solanum sarrachoides Sendtn. var. sarachidium (Bitter) C.V.Morton, Revis. Argentine Sp. Solanum 122. 1976. Type. Based on Solanumsarachidium Bitter.

##### Type.

Brazil. “Brasilia australis”, *F. Sellow s.n.* (lectotype, designated by Edmonds 1986, pg. 16: P [P00371162]).

##### Description.

Annual herbs to 0.7 m high, usually smaller (but very rarely to 1 m), spreading and decumbent with age. Stems terete, green, generally erect, branching and later spreading, not markedly hollow; new growth densely viscid-pubescent with simple, uniseriate, spreading trichomes with a glandular apical cell, the trichomes of two lengths, 1–4-celled trichomes to 0.5 mm long and 5–14-celled trichomes to 2 mm long; older stems glabrescent. Sympodial units difoliate, the leaves not geminate. Leaves simple and sinuate-dentate, the blades 3–7.5 cm long, 3–6 cm wide, broadly ovate, widest in the lower third, thinly membranous, concolorous; adaxial and abaxial surfaces sparsely to densely pubescent with spreading, simple, uniseriate glandular trichomes like those of the stem, evenly distributed on lamina and veins; major veins 3–4 pairs; base truncate to cordate, sometimes asymmetric; margins entire or regularly sinuate-dentate; apex acute; petioles 0.5–3.2 cm long, sparsely pubescent with trichomes like those of the stem and leaves. Inflorescences usually opposite the leaves but occasionally internodal (always very near the node), unbranched, 0.7–1.7 cm long, with 2–5(6–7) flowers clustered at the tip (sub-umbelliform), sparsely pubescent with spreading trichomes like those of the stems; peduncle 0.7–1 cm long; pedicels 5–7 mm long, 0.1–0.2 mm in diameter at the base, 0.3–0.4 mm in diameter at the apex, straight and spreading, articulated at the base; pedicel scars spaced ca. 0(–1) mm apart. Buds globose, the corolla only slightly exserted from the calyx tube before anthesis, almost completely included within the calyx lobes and only the tip of the corolla showing. Flowers 5-merous, cosexual (hermaphroditic). Calyx tube 0.5–1 mm long, the lobes 1.5–2 mm long, 1.3–1.5 mm wide, lanceolate to narrowly ovate with acute apices, sparsely pubescent with 1–4-celled spreading glandular trichomes like those on the pedicels but shorter. Corolla 0.5–0.8 cm in diameter, white with a yellow-green central eye, pentagonal-stellate, lobed 1/3 of the way to halfway to the base, the lobes 3–4.5 mm long, 5–7 mm wide, spreading at anthesis, sparsely papillate-pubescent abaxially with glandular 1–4-celled simple uniseriate trichomes and eglandular papillae, these denser along margins, tips and midvein. Stamens equal; filament tube minute; free portion of the filaments 1–1.5 mm long, adaxially sparsely pubescent with tangled uniseriate 4–6-celled simple trichomes; anthers 1.2–2 mm long, 0.4–0.8 mm wide, ellipsoid, yellow, poricidal at the tips, the pores lengthening to slits with age and drying. Ovary globose, glabrous; style 3–3.5 mm long, straight, not usually exserted beyond the anther cone, densely pubescent with 2–3-celled simple uniseriate trichomes in the lower half to 2/3 where included in the anther cone; stigma capitate, minutely papillate, green in live plants. Fruit a globose berry, 0.6–0.9 cm in diameter, green brownish grey at maturity, the pericarp usually matte, opaque, glabrous; fruiting pedicels 5–9 mm long, 0.2–0.3 mm in diameter at the base, ca. 1 mm in diameter at the apex, spaced 0–1 mm apart, reflexed, not persistent; fruiting calyx accrescent, becoming papery in mature fruit, the tube 3–4 mm long, the lobes 5.5–8 mm long and 3.5–4 mm wide, the tips slightly reflexed or spreading. Seeds (23-)59–69(-93) per berry, 1.3–1.7 mm long, 1–1.5 mm wide, flattened and teardrop shaped with a subapical hilum, pale yellow, the surfaces minutely pitted, the testal cells pentagonal in outline. Stone cells 4–6 per berry, (0.5) 0.8–1 mm in diameter. Chromosome number: 2n = 24 (see Särkinen et al. 2018).

##### Distribution

**Figure 157.**
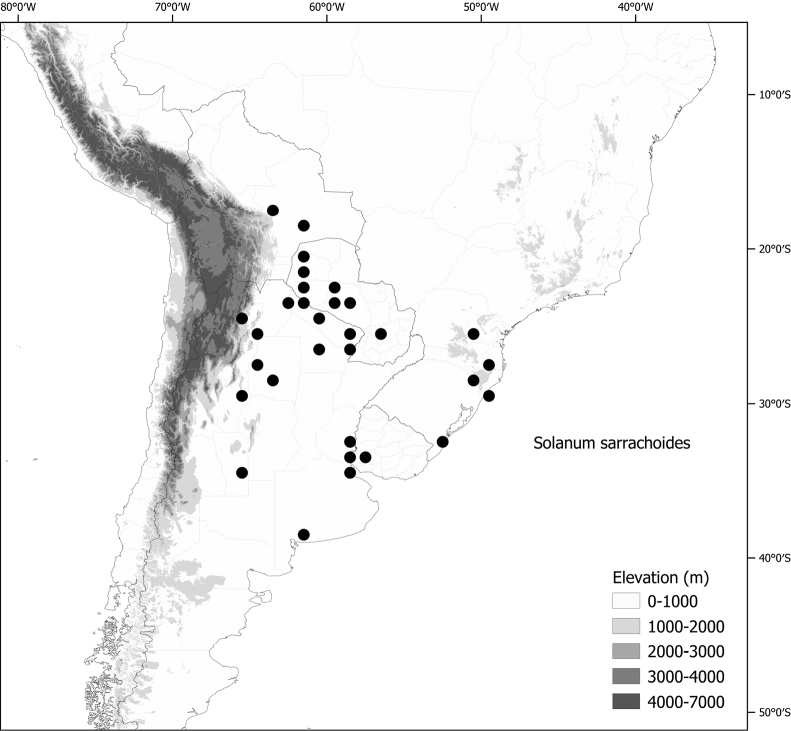
Distribution map of *Solanumsarrachoides* in South America. For adventive distribution in North and Central America and the Caribbean, see Knapp et al. (2019: 111) and Särkinen et al. (2018: 145) for the Eastern Hemisphere.

**(Fig. 157).***Solanumsarrachoides* is native to southern South America, occurring in Brazil (States of Paraná, Rio Grande do Sul, Santa Catarina, São Paulo,), Bolivia (Dept. Santa Cruz), Argentina (Provs. Buenos Aires, Catamarca, Chaco, Entre Ríos, Formosa, San Luis, Santiago del Estero), Paraguay (Depts. Boquerón, Nueva Asunción, Presidente Hayes) and Uruguay (Depts. Florida, Lavalleja, Montevideo, San José). It is sporadically introduced in the temperate zones of both Northern and Southern Hemispheres, where it is much less common than the morphologically similar *S.nitidibaccatum* (see Särkinen et al. 2018, Knapp et al. 2019 for details of extra-South American distribution of both these species).

##### Ecology and habitat.

*Solanumsarrachoides* occurs in a wide variety of dry and semi-humid habitats, often in open or disturbed areas such as the edges of agricultural fields, and sporadically occurs as a weed of cultivation in urban areas, from sea level to 1,000 m elevation.

##### Common names and uses.

Bolivia. Santa Cruz: huiraquillomi (Guaraní, *Michel et al. 2769*). No uses recorded.

##### Preliminary conservation status

**(IUCN 2022).** Least Concern [LC]. EOO = 2,194,057 km^2^ [LC]; AOO = 128 km^2^ [EN]; calculated on South American range only. *Solanumsarrachoides* is widespread in the Paraná Basin and is a weedy somewhat ephemeral species in a wide variety of habitats; it is introduced elsewhere as an agricultural weed (see Särkinen et al. 2018; Knapp et al. 2019 for details).

##### Discussion.

Many accounts of morelloid solanums from outside South America have treated as *Solanumsarrachoides* specimens of the species whose correct name is *S.nitidibaccatum* (see references in Särkinen et al. 2018; Knapp et al. 2019). Records of *S.sarrachoides* in the literature should therefore be dealt with care due to common misidentification of voucher material. The two species can be distinguished based using the following suite of characters: *S.sarrachoides* has generally truncate leaf bases, umbellate to sub-umbellate mature inflorescences opposite the leaves (Fig. 156B) with fewer flowers (2–5), shorter calyx lobes 1–1.4 mm long and a corolla with yellow-green central eye. *Solanumnitidibaccatum* has cuneate leaf bases, usually internodal mature inflorescences with an elongate flower-bearing axis with more flowers (4–8), longer calyx lobes 1.8–2.5 mm long, and corolla with black-purple edged central eye. The accrescent calyx almost completely encloses the matte-surfaced mature berry in *S.sarrachoides*, while the shiny, marbled berry of *S.nitidibaccatum* is always ca. halfway exserted from the calyx lobes. *Solanumsarrachoides* usually has more stone cells in each berry (4–6) than does *S.nitidibaccatum* (1–2, or absent). Though morphologically very similar, data from both nuclear and plastid DNA sequences suggests the two species are not closely related (Gagnon et al. 2022).

Typification details of the synonyms of *S.sarrachoides* can be found in Barboza et al. (2013) and Särkinen et al. (2018).

#### ﻿52. Solanum scabrum

Taxon classificationPlantaeSolanalesSolanaceae

Mill., Gard. Dict. ed. 8, no. 6. 1768.

**Figure 158.**
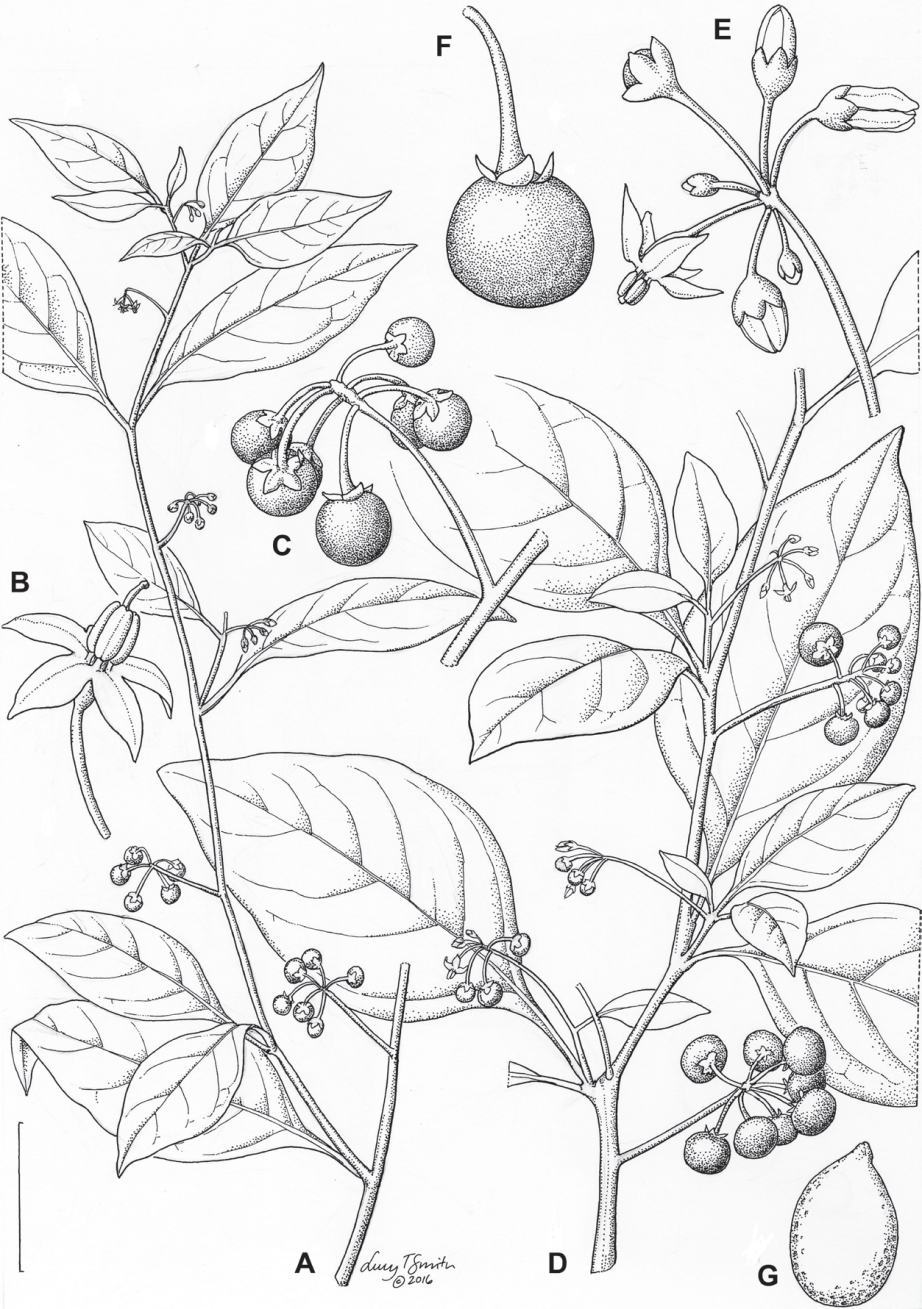
*Solanumscabrum***A** habit of wild form **B** flower of wild form **C** infructescence of wild form **D** habit of cultivated form **E** inflorescence of cultivated form **F** fruit of cultivated form **G** seed (**A–C***Pilz 2108***D–G***Nee 16088*). Illustration by L. Smith. Previously published in Särkinen et al. (2018: 148) and Knapp et al. (2019: 113).

**Figure 159.**
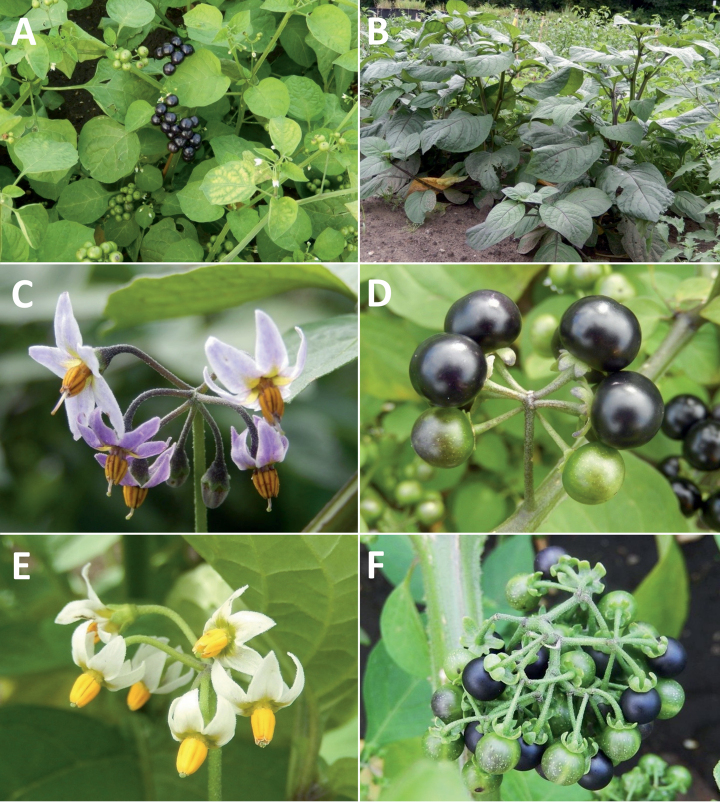
*Solanumscabrum***A** common habit **B** habit in taller varieties **C** flowers of the larger berried variety at full anthesis **D** fruits of a large-berried morph **E** flowers of the small-berried morph at full anthesis **F** fruits of a small-berried morph (**A** Nijmegen acc. # BG13 **B** Nijmegen acc. # A34750072 **C** Nijmegen acc. # GB22 **D** Nijmegen accession H065 **E** Nijmegen acc. # A34750067 **F** Nijmegen acc. # 2010/3). Photos by S. Knapp. Previously published in Särkinen et al. (2018: 149) and Knapp et al. (2019: 114).

Figs 158 , 159 Types based on American specimens only; for full synonymy, see Särkinen et al. (2018: 146–147)

Solanum fistulosum Dunal, Encycl. [J. Lamarck & al.] Suppl. 3: 749. 1814. Type. “Originaire de l’Isle de France [Mauritius], est cultivée en Amerique [Brazil]”, *Herb. Richard s.n.* (lectotype, designated by D’Arcy 1974a, pg. 735: P [P00335259]).
Solanum oleraceum Dunal var. macrocarpum Dunal, Prodr. [A. P. de Candolle] 13(1): 50. 1852. Type. Brazil. Bahia: Ilheus, 1841, *C.F.P. Martius 1255* (lectotype, designated by Edmonds 1972, pg. 108 [as holotype]: G-DC [G00144295]; isolectotype: P [P00366815]).

##### Type.

Cultivated in Chelsea Physic Garden, said in protologue to “grow naturally in North America”, *Herb. Miller s.n.* (lectotype, designated by Henderson 1974, pg. 61 [as type]: BM [BM000847083]).

##### Description.

Annual or short-lived perennial herbs to 1.5 m high, often woody at the base. Stems terete, ridged, or winged, green to purple, erect or ascending, if ridged or winged the stems later with spinose processes, usually somewhat hollow; new growth puberulent with simple spreading uniseriate 2–8-celled eglandular trichomes 0.3–0.8 mm long; older stems glabrescent, with or without prominent spinose processes. Sympodial units difoliate, the leaves usually not geminate, but if leaves paired, then one is usually smaller. Leaves simple to rarely shallowly sinuate, the blades 4–15(20) cm long, 3–10(16) cm wide, broadly ovate to elliptic, widest in the lower half, very variable in size depending on cultivars and growth conditions, membranous, usually discolorous; adaxial and abaxial surfaces glabrous or sparsely pubescent with simple uniseriate trichomes like those on the stem mainly along veins and scattered along lamina; principal veins 3–6(–8) pairs, paler green or often purple tinged; base abruptly acute or truncate, narrowly winged onto the petiole; margins entire or rarely shallowly sinuate; apex rounded to acute; petioles 1–5(8) cm long, glabrous or sparsely pubescent with simple uniseriate trichomes like those of the stem. Inflorescences internodal, unbranched, forked or many times branched (in cultivars), 1–2 (–4) cm long, with 4–10(30+) flowers clustered towards the tips (sub-umbelliform) or spread along the axis, glabrous or sparsely pubescent with simple uniseriate trichomes like those on the stem; peduncle 1–5(–8) cm long, erect and thick, much thickened at the apex, subwoody, green or purple-tinged; pedicels 0.4–1 cm long, 0.3–0.5 mm in diameter at the base, 0.75–0.9 mm in diameter at the apex and abruptly expanding to the calyx tube, stout, erect and/or spreading, green or purple-tinged, glabrous or minutely pubescent like the peduncle, articulated at the base; pedicel scars tightly clustered near the tip of the axis, spaced 0–2 mm apart, sometimes with short stumps ca. 0.5–1 mm long. Buds globose to subglobose, the corolla exserted 1/2–1/3 from the calyx tube before anthesis. Flowers 5-merous or occasionally fasciate and 6–7-merous in cultivars, cosexual (hermaphroditic). Calyx tube 0.9–1.1 mm long, abruptly cup-shaped with a broad base, the lobes slightly unequal, 0.9–1.5 mm long, 0.5–1.5 mm wide, broadly deltate with a rounded tip, green or purple-tinged, glabrous or sparsely pubescent with simple uniseriate trichomes like those of the pedicels, the margins often drying scarious and white. Corolla 0.7–1.2 cm in diameter, white, purple-tinged or occasionally lilac to dark purple, with a yellow basal star, stellate, lobed ca. 1/2 of the way to the base, the lobes 2.5–4 mm long, 1.5–3 mm wide, spreading or reflexed, densely papillate on tips and margins. Stamens equal; filament tube very short, to 0.1 mm long; free portion of the filaments 0.5–0.8 mm long, glabrous or pubescent with tangled uniseriate simple trichomes; anthers 2–3 mm long, ellipsoid or slightly tapering towards the tips, yellow, orange or brown, poricidal at the tips, the pores lengthening to slits with age and drying, the connective often becoming brownish black in dry specimens. Ovary rounded, glabrous; style 2.5–5 mm long, straight, exserted beyond the anther cone, densely pubescent with simple uniseriate trichomes 0.2–0.5 mm long in the basal 1/2 where included in the anther cone; stigma capitate, the surface minutely papillate. Fruit a globose to slightly flattened berry, 1–2 cm in diameter, purplish black at maturity, the pericarp thick, shiny, opaque, glabrous; fruiting pedicels 0.7–1.5(2) cm long, 0.5–1 mm in diameter at the base, 1.1–1.5 mm in diameter at the apex, stout, erect and spreading, purple or brown, usually not falling with the fruit, persistent, remaining on the plant on older inflorescences; fruiting calyx not accrescent, the tube 1.5–2 mm long, usually tearing unevenly, the lobes 2–3 mm long, usually with thicker white margins in dry material, appressed or spreading to slightly reflexed. Seeds (20-)100–150 per berry, 2–2.8 mm long, 1.5–1.8 mm wide, flattened and teardrop shaped with a subapical hilum, yellow-brown or purple, the surfaces minutely pitted, thin and the embryo clearly visible, the testal cells rectangular to pentagonal in outline. Stone cells absent. Chromosome number: *2*n = 72 (see Särkinen et al. 2018).

##### Distribution.

*Solanumscabrum* is native to tropical Africa and has been introduced worldwide as a cultivated plant as a result of trafficking in enslaved peoples. In South America, apart from the type of *S.fistulosum*, we have only seen two collections, both from Brazil (States of Bahia, Rio de Janeiro), one of these (*Amorim 21*) cultivated in the Jardim Botânico do Rio de Janeiro and the other (*Martius 1255*) of uncertain origin. A map of the native distribution of *S.scabrum* can be seen in Särkinen et al. (2018: 151, Fig. 48).

##### Ecology and habitat.

*Solanumscabrum* is only known from cultivation in South America, although plants could persist in subtropical areas.

##### Common names and uses.

In its native range *S.scabrum* is a prized plant for its juicy berries and its nutritious leaves that are used as a potherb (see Särkinen et al. 2018 for a summary of the uses of *S.scabrum*).

##### Preliminary conservation status

**(IUCN 2022).** We have not assessed *S.scabrum* for South America since it is only cultivated here; for conservation status in its native range in Africa see Särkinen et al. (2018).

##### Discussion.

*Solanumscabrum* is a species known only from cultivation in the Americas. It is the mostly commonly cultivated morelloid species in Africa, and there is used for both its leaves (eaten as a potherb) and its fruits. Specimens of *S.scabrum* occasionally have been collected from areas where enslaved people were brought from western Africa (e.g., Bahia, *Martius 1255*), so it is possible it could occur elsewhere in the region.

*Solanumscabrum* can be distinguished from the somewhat similar *S.americanum* by the larger anthers (2.5–3 mm long versus 0.8–1.5 mm long) that usually dry a dirty brownish tan. In both these species the berries drop off without the pedicels at maturity and lack stone cells except in some populations of *S.americanum* where up to four stone cells have been observed (other populations lacking stone cells completely). Both *S.scabrum* and *S.americanum* have purple-black, shiny berries.

Material seen from South America represents only a fraction of the diversity of *S.scabrum* across its native range in Africa (see Olet 2004; Olet et al. 2006; Manoko 2007) and are specimens of cultivars with simple inflorescences, possibly originally brought for use as vegetables or fruits.

Typification details for the synonyms of *S.scabrum*, and a complete discussion of its morphological variability and many uses in its native range can be found in Särkinen et al. (2018).

#### ﻿53. Solanum sinuatiexcisum

Taxon classificationPlantaeSolanalesSolanaceae

Bitter, Repert. Spec. Nov. Regni Veg. 10: 558. 1912.

Figs 160 , 161

Solanum hyoscyamoides Bitter, Repert. Spec. Nov. Regni Veg. 11: 236. 1912. Type. Bolivia. La Paz: Prov. Larecaja, “viciniis Sorata, colle Catarguata”, *G. Mandon 395* (lectotype, designated by Barboza and Hunziker 2005, pg. 63: W [acc. # 0001339]; isolectotypes: BM [BM000617679], F [v0073294F, acc. # 976770, fragment of P isolectotype], G [G00343478], K [K000585618], P [P00335950]).
Solanum deltoideum Rusby, Descr. S. Amer. Pl. 115. 1920. Type. Bolivia. “Yungas”, 1890, *M. Bang 740* (lectotype, designated by Barboza et al. 2013, pg. 258: NY [00139127]; isolectotypes BM [BM000778125], GH [00077611], MO [MO-2099411, acc. # 2056613], NY [00139128], PH [00030402], US [00027544, acc. # 57454], W [acc. # 1890-001426]).

##### Type.

Bolivia. La Paz: Unduavi, Sudyungas, Nov 1920, *O. Buchtien 2962* (lectotype, designated by Barboza and Hunziker 2005, pg. 63 [first stage], second stage designated by Barboza et al. 2013, pg. 258: US [00650477, acc. # 703362]; isolectotypes: GOET [GOET003591], K [K000585617], LD, M, NY [00172175, 00172176], SI [003342], US [00027799, acc. # 1175816).

**Figure 160.**
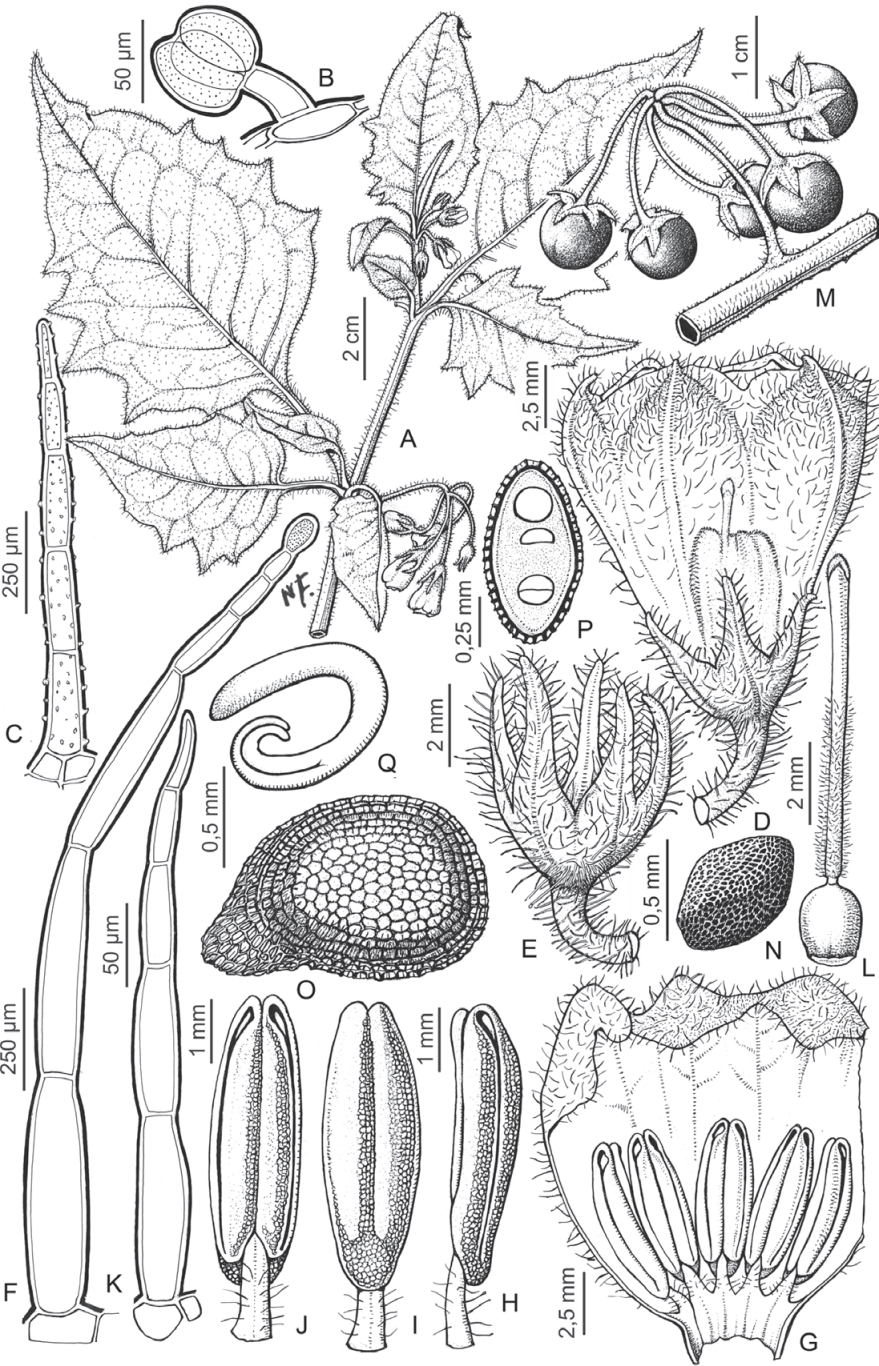
*Solanumsinuatiexcisum***A** flowering branch **B** glandular trichome of the leaf **C** eglandular trichome of the leaf **D** flower **E** calyx **F** glandular trichome of the calyx **G** dissected flower **H** stamen, lateral view **I** stamen, dorsal view **J** stamen, ventral view **K** eglandular trichome of the filament **L** gynoecium **M** infructescence **N** stone cell **O** seed **P** seed cross section **Q** embryo (**A–G***Solomon 13073***M–Q***Nee & Solomon 36671*). Illustration by N. de Flury. Previously published in Barboza et al. (2013: 259).

##### Description.

Robust herbs or subwoody shrubs, 0.75–2.5 m high, erect. Stems terete or somewhat angled with longitudinal ridges, densely pubescent with transparent glandular and eglandular 5–10-celled simple, uniseriate trichomes 1–4 mm long, the terminal gland, if present, unicellular, somewhat glabrescent with age; new growth densely pubescent with mixed glandular and eglandular trichomes like those of the stems, viscid to the touch; bark of older stems pale greenish brown. Sympodial units difoliate, the leaves geminate, members of a pair equal in size and shape. Leaves simple and shallowly toothed, the blades (4.5–) 10–16 (–22.5) cm long, (2.3–) 6–12 cm wide, ovate to ovate-elliptic, widest at the middle or in the lower half, membranous, concolorous; adaxial surfaces evenly and moderately pubescent with transparent glandular and eglandular 6–10-celled simple uniseriate trichomes 1–2.5 mm long, the glands if present unicellular; abaxial surfaces with similar mixed glandular and eglandular pubescence of transparent simple uniseriate trichomes, but sparser on the lamina and denser on the midrib and main veins than on adaxial surfaces; principal veins 4–6, densely pubescent abaxially; base cuneate to attenuate onto the petiole; margins coarsely and irregularly serrate or dentate, with 4–10 teeth mostly in the lower third of the blade, directed upwards or outwards, the sinuses broad and somewhat deep, reaching ca. 1/10 of the way to the midrib; apex acuminate; petioles 1–4.5 (–6) cm long, moderately pubescent with transparent glandular and eglandular trichomes like those of the stems. Inflorescences internodal, unbranched, 2–4.5 cm long, with 4–8 flowers clustered in the distal third to quarter, densely pubescent with transparent glandular and eglandular simple uniseriate trichomes 1–4 mm long; peduncle 2–3.5 cm long; pedicels 0.7–1.2(–1.4) cm long, 0.5–0.6 mm in diameter at the base, ca. 1 mm in diameter at the apex, nodding at anthesis, densely pubescent with mixed glandular and eglandular transparent simple uniseriate trichomes like those of the rest of the inflorescence, articulated at the base; pedicel scars ca. 1 mm apart at the tip of the inflorescence. Buds elliptic to obelliptic, the corolla strongly exserted from the calyx before anthesis. Flowers 5-merous, cosexual (hermaphroditic). Calyx tube 1.8–2 mm long, conical, the lobes 3.5–6 mm long, ca. 0.5 mm wide, narrowly triangular, densely pubescent with transparent glandular and eglandular simple uniseriate trichomes 1–4 mm long like the rest of the inflorescence, denser at the tips and margins. Corolla 1.5–1.7 cm in diameter, light purple or blue-violet, with dark purple or yellow ring at base within at anthesis, openly campanulate, lobed less than 1/10 of the way to the base, the lobes minute, 1–1.5 mm long, 3–4 mm wide, cucullate at the tips, loosely and sparsely pubescent abaxially with simple uniseriate trichomes. Stamens equal; filament tube minute; free portion of the filaments 2–2.5 mm long, glabrous or with a few tangled simple uniseriate trichomes adaxially; anthers 3.5–4 (5) mm long, 1.2–1.5 mm wide, ellipsoidal, yellow, poricidal at the tips, the pore lengthening to slits with age. Ovary subglobose, glabrous; style 6–9 mm, straight, exserted beyond the anther cone, densely pubescent in the lower half with papillate trichomes; stigma broadly capitate to saddle-shaped and somewhat bilobed, the surfaces minutely papillate, bright green in live plants. Fruit a globose berry, 0.7–1.2 cm in diameter, green at maturity, the pericarp thin, matte and opaque, glabrous; fruiting pedicels 1.2–1.5 cm long, ca. 1 mm in diameter at the base, ca. 2 mm in diameter at the apex, not markedly woody, strongly deflexed and curving at the base, not persistent; fruiting calyx slightly accrescent, appressed to the berry, the tube ca. 3 mm long, the lobes to 6 mm long. Seeds 30–70 per berry, 1.5–1.7 mm long, 1.1–1.2 mm wide, teardrop shaped but not markedly flattened, pale yellow brown, the surfaces minutely pitted, the testal cells pentagonal to polygonal in outline, with distinct strands of hair-like thickenings from the lateral cell walls. Stone cells 2–8, 0.5–0.8 mm in diameter, small and scattered throughout the berry, cream-coloured. Chromosome number: 2n = 24 (reported in Edmonds and Chweya 1997 cannot be verified, no voucher cited).

**Figure 161.**
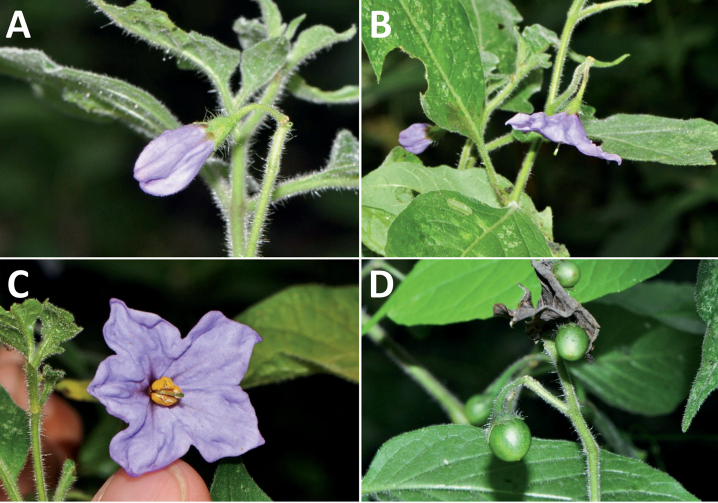
*Solanumsinuatiexcisum***A** flower bud **B** flower at full anthesis (side view) **C** flower at full anthesis (front view) **D** maturing fruits (**A–D***Barboza et al. 3497*). Photos by S. Knapp.

##### Distribution

**Figure 162.**
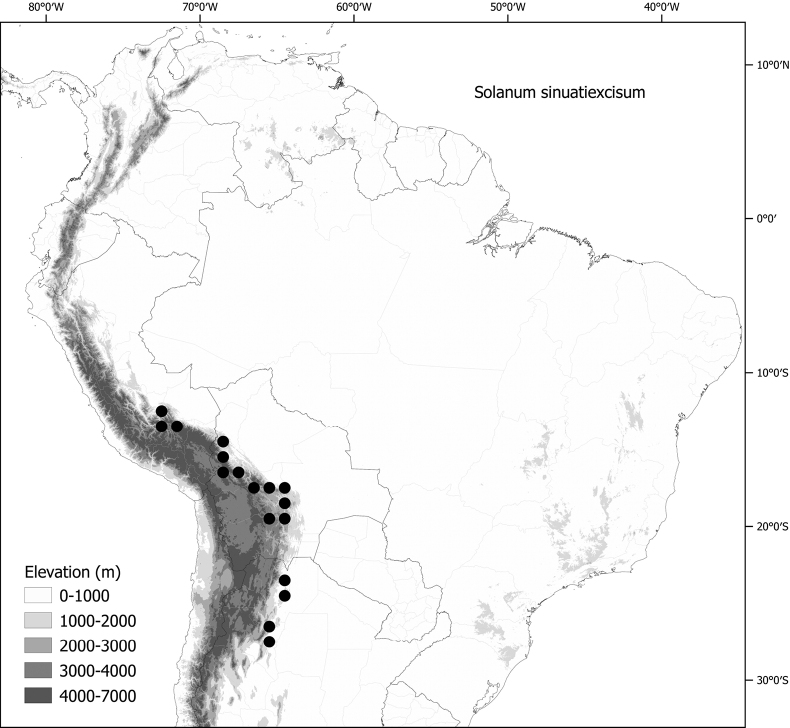
Distribution map of *Solanumsinuatiexcisum*.

**(Fig. 162).***Solanumsinuatiexcisum* occurs from southern Peru (Dept. Cusco) and central Bolivia (Depts. Cochabamba, La Paz, Santa Cruz) to northern Argentina (Provs. Catamarca, Jujuy, Salta).

##### Ecology and habitat.

*Solanumsinuatiexcisum* grows in montane and premontane forests (‘yungas’), often at the edges of open areas along streams and light gaps in the forest, at elevations from 500 to 3,200 m elevation.

##### Common names and uses.

None recorded.

##### Preliminary conservation status

**(IUCN 2022).** Least Concern [LC]. EOO = 625,487 km^2^ [LC]; AOO = 148 km^2^ [EN]. *Solanumsinuatiexcisum* is widely distributed but not often collected; the low AOO is most likely due to collection deficit. It has been collected in protected areas in Argentina (Parque Nacional El Rey) and Bolivia (e.g., Parque Nacional Carrasco).

##### Discussion.

*Solanumsinuatiexcisum* is a member of what was previously recognised as section Campanulisolanum (Barboza and Hunziker 2005), together with the very similar *S.fiebrigii*. They are resolved as sister taxa with molecular data (Särkinen et al. 2015b; Gagnon et al. 2022). Both species are large lax herbs with campanulate flowers and long, simple eglandular pubescence. *Solanumsinuatiexcisum* differs from *S.fiebrigii* in having strictly unbranched, usually subumbellate inflorescences with reflexed fruiting pedicels (versus forked inflorescences with spreading pedicels in fruit in *S.fiebrigii*) and in its narrowly triangular calyx lobes that are always longer than the calyx tube (versus deltate calyx lobes that are usually shorter than the calyx tube). *Solanumfiebrigii* is more widely distributed and more commonly collected than *S.sinuatiexcisum* where their ranges overlap.

#### ﻿54. Solanum sinuatirecurvum

Taxon classificationPlantaeSolanalesSolanaceae

Bitter, Repert. Spec. Nov. Regni Veg. 11: 241. 1912.

Figs 163 , 164

Solanum pulchellum Phil., Anales Univ. Chile 523. 1873., nom. illeg., non Solanumpulchellum F.Muell. (1855). Type. Chile. Región II (Antofagasta): Salitreras de Antofagasta en el desierto de Atacama, *G. Döll s.n.* (lectotype, designated by Barboza et al. 2013, pg. 259: SGO [SGO000004589, acc. # 042726]; isolectotypes: K [K000005316], SI [003353]).
Solanum sinuatirecurvum Bitter subsp. crispatellum Bitter, Repert. Spec. Nov. Regni Veg. 11: 242. 1912. Type. Argentina. Sin. loc., *R. Hauthal 58* (holotype: B, destroyed [F neg. 2790]; lectotype, designated by Barboza et al. 2013, pg. 259: F [v0073407F, acc. # 621148 fragment of B holotype]).
Solanum metarsium C.V.Morton, Revis. Argentine Sp. Solanum 72. 1976. Type. Based on (replacement name for) Solanumpulchellum Phil.

##### Type.

Bolivia. [Oruro]: Puna Patanca, 6 Jan 1904, *K. Fiebrig 2471* (holotype: B [destroyed, F. neg. 2722]; lectotype, designated by Barboza et al. 2103, pg. 259: F [v0073406F, acc. # 621223, fragment of B holotype]).

**Figure 163.**
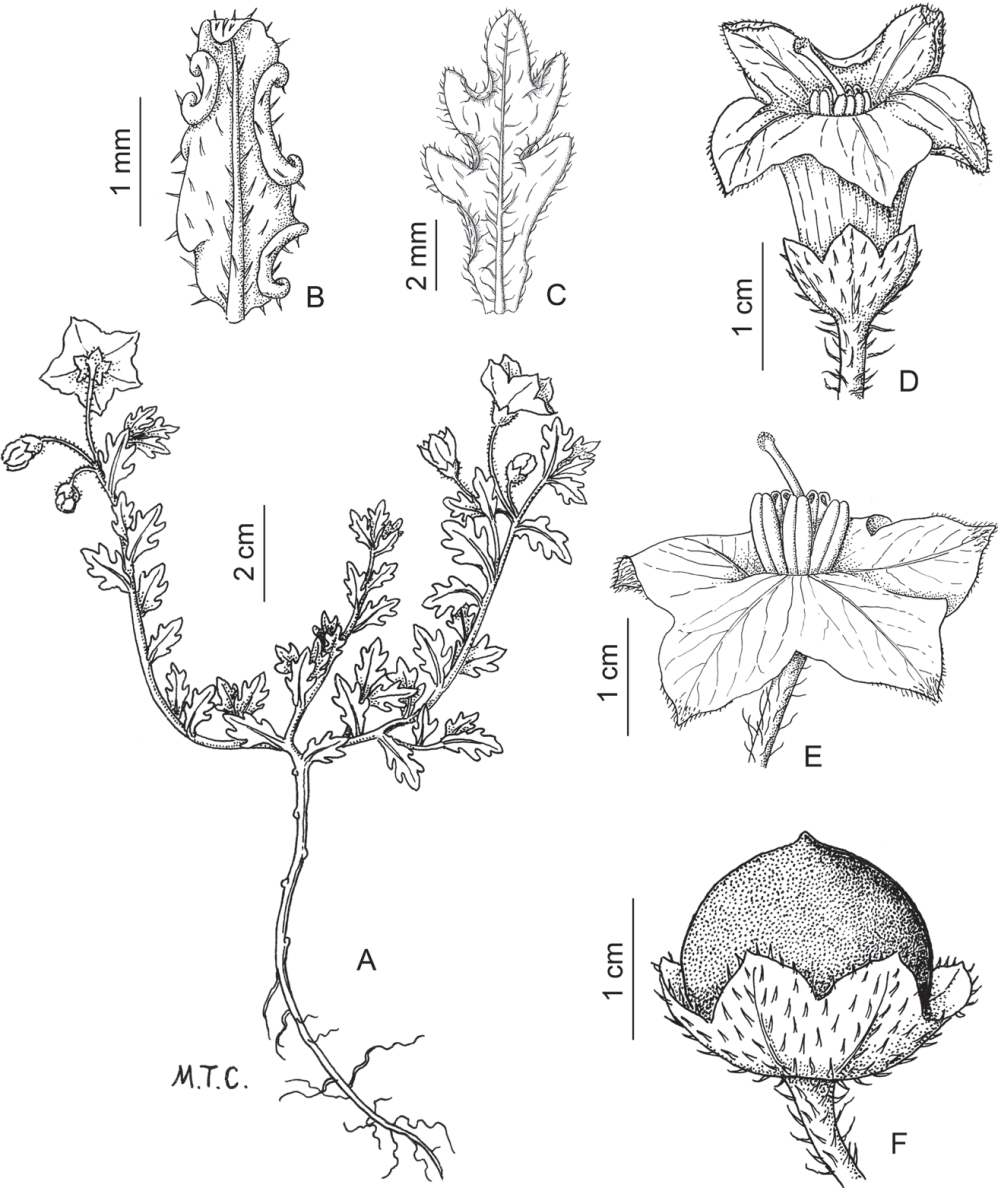
*Solanumsinuatirecurvum***A** habit **B** leaf, abaxial surface **C** leaf, adaxial surface **D, E** flowers **F** fruit (**A–F** voucher details missing). Illustration by M.T. Cabrera. Previously published in Barboza et al. (2013: 259).

##### Description.

Perennial herbs from deep woody rhizomes or tap roots (to 15 cm below soil surface), 0.05–0.2 m high, the branches spreading, woody at the base. Stems angled and winged from the decurrent leaf bases, moderately to densely pubescent with tangled white eglandular 5–10-celled simple uniseriate trichomes 1–1.5 mm long, these occasionally gland-tipped, occasionally very small sessile glands also present on stems; new growth densely pubescent with tangled white eglandular 5–10-celled simple uniseriate trichomes 1–1.5 mm long, these occasionally gland-tipped, occasionally a dense covering of very small sessile glands also present; bark of older stems greenish white. Sympodial units plurifoliate, the leaves not geminate. Leaves simple, shallowly lobed to pinnatifid, the blades 0.6–3.5(5) cm long, 0.2–1.5(2) cm long, narrowly elliptic in outline, widest at the middle, thick and coriaceous or somewhat fleshy, concolorous, variable in size between populations; adaxial and abaxial surfaces sparsely to densely pubescent with tangled white eglandular 5–10-celled simple uniseriate trichomes 1–1.5 mm long like those of the stems; principal veins usually not visible in small-leaved plants, if visible then 3–4 pairs corresponding to the number of leaf lobes; base attenuate onto the petiole and the leaves sessile; margins irregularly moderately to deeply lobed or erose, the lobes 1–2(–4) pairs, 0.5–2.5 mm long with acute to rounded or blunt tips, always oriented pointed to leaf apex, the sinuses reaching ca. 1/3–1/2 of the way to the midrib, strongly revolute between the lobes; apex acute to rounded; petioles absent, the leaves sessile. Inflorescences terminal, unbranched, 0.5–2 cm long, with 2–5 flowers clustered at the tips, sparsely to moderately pubescent with tangled white eglandular simple uniseriate trichomes 1–1.5 mm long like those of the stems; peduncle 0.5–1.9 cm long; pedicels 1.5–2 cm long, ca. 0.5 mm in diameter at the base, ca. 1.5 mm in diameter at the apex, filiform, spreading at anthesis, articulated at the base, often dark purple or at least darker than the leaves; pedicel scars irregularly spaced 0–5 mm apart, sometimes overlapping. Buds ellipsoid, ca. halfway exserted from the calyx before anthesis. Flowers 5-merous, cosexual (hermaphroditic). Calyx tube 1.5–2 mm long, conical to deeply cup-shaped, the lobes 3–3.5 mm long, 1.5–2 mm wide, triangular with sharply pointed tips, the margins sometimes shallowly lobed, thick and leathery, sparsely pubescent with tangled white eglandular simple uniseriate trichomes 1–1.5 mm long like those of the rest of the inflorescence. Corolla 2–2.6 cm in diameter, deep purple with a greenish brown central star that is shinier than the rest of the corolla, shallowly stellate, lobed 1/3 to halfway to the base, the lobes 5–7 mm long, 5–7 mm wide, broadly deltate, spreading to reflexed, glabrous adaxially, sparsely to densely pubescent along the midveins, tips and margins abaxially with eglandular, simple uniseriate trichomes ca. 0.5 mm long, the lobe tips cucullate early in anthesis, the corolla apparently expanding over the course of flowering. Stamens slightly unequal, 3 lower ones longer due to filament length difference; filament tube less than 0.5 mm long; free portion of the filaments 0.5–1.1 mm long, glabrous or with a few tangled weak simple uniseriate trichomes adaxially, the lower 3 longer than the upper 2; anthers 4–5 mm long, 1–1.5 mm wide, ellipsoid, yellow, apparently unequal in size but this due to filament length difference, poricidal at the tips, the pores lengthening to slits with age. Ovary conical, glabrous; style ca. 9 mm long, slightly curved so it emerges from between the lower 3 anthers, exserted beyond the anther cone, papillate in the lower third within the anther cone; stigma capitate to clavate, bright green in live plants, the surface minutely papillate. Fruit a globose berry, 1–1.3 cm in diameter, bright yellow when mature, green to greenish purple when immature, the pericarp somewhat leathery, matte, opaque, glabrous; fruiting pedicels 2–2.5 cm long, ca. 1 mm in diameter at the base, ca. 2 mm in diameter at the apex, strongly deflexed and directing the berry towards the soil, the entire pedicel curving, somewhat woody at fruit maturity, persistent; fruiting calyx not accrescent, the lobes ca. 5 mm long, ca. 2.5 mm wide, becoming woody and brittle with fruit age. Seeds (5)10–20 per berry, 3–3.5 mm long, 2–3 mm wide, flattened and teardrop shaped, pale yellowish tan to brown, the surfaces minutely pitted, the testal cells deeply sinuate in outline. Stone cells absent. Chromosome number: n = 12 (Moyetta et al. 2013; voucher *Barboza et al. 3551*).

**Figure 164.**
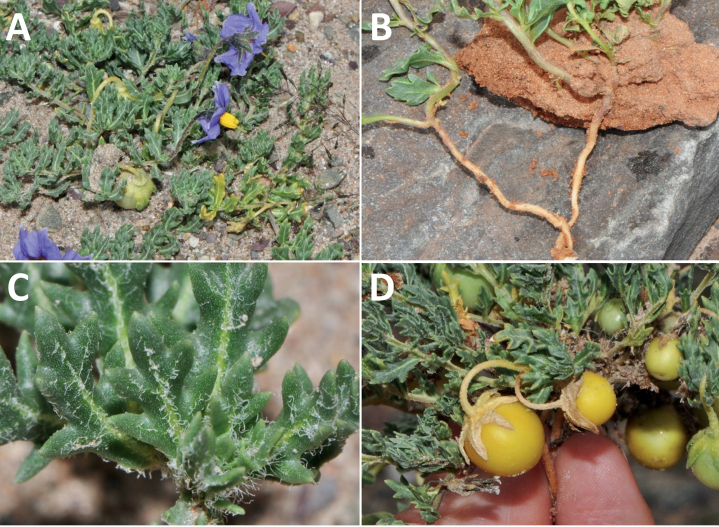
*Solanumsinuatirecurvum***A** habit **B** underground rhizome **C** leaf indumentum (top surface) **D** maturing fruits (**A, C, D***Barboza et al. 3557***B***Barboza et al. 3551*). Photos by S. Knapp.

##### Distribution

**Figure 165.**
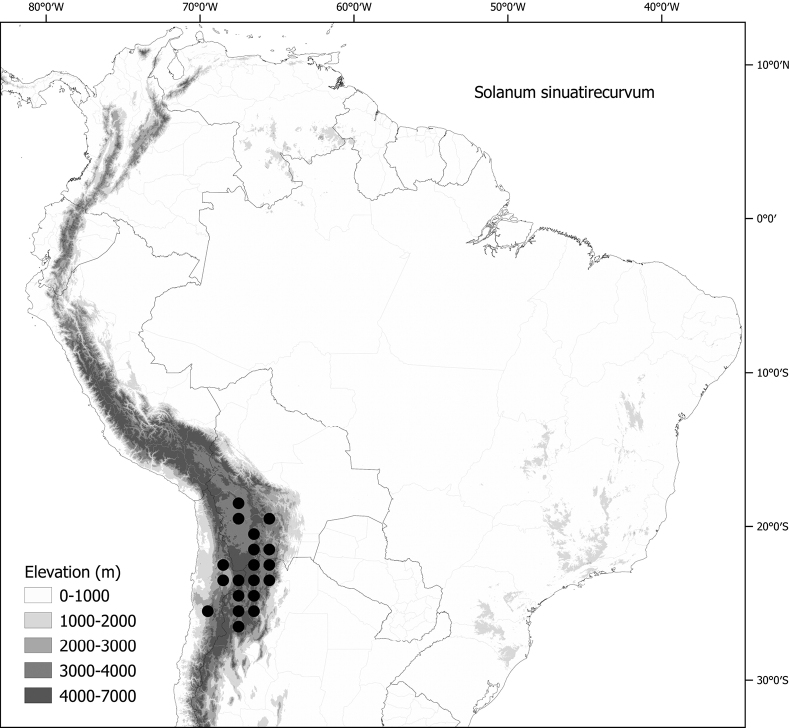
Distribution map of *Solanumsinuatirecurvum*.

**(Fig. 165).***Solanumsinuatirecurvum* occurs in the high Andes from Bolivia (Depts. Oruro, Potosí) to northwestern Argentina (Provs. Catamarca, Jujuy, Salta) and adjacent Chile (Region II [Antofagasta]).

##### Ecology and habitat.

*Solanumsinuatirecurvum* is a species of open, very high elevation dry habitats above treeline (puna or high elevation deserts), usually growing in sandy or gravelly soils, often amongst grasses, from 3,000 to 5,000 m elevation.

##### Common names and uses.

Argentina. Catamarca: chuschalin (*Hueck 504*); Jujuy: ají (*Claren 11369*), porotillo (*Cabezas 49*); Salta: cora cora (*Krapovickas 3192*). Chile. Region II (Antofagasta): salvilla (*Wickens et al. 11*). No uses recorded.

##### Preliminary conservation status

**(IUCN 2022).** Least Concern [LC]. EOO = 231,683 km^2^ [LC]; AOO = 344 km^2^ [EN]. *Solanumsinuatirecurvum* occurs in large populations and is widely distributed in high elevation habitats. It is found in protected areas in Argentina (e.g., Quebrada de Humahuaca World Heritage site, although there are conservation concerns around that site, https://whc.unesco.org/en/soc/4176/).

##### Discussion.

*Solanumsinuatirecurvum* is a member of the small *Episarcophyllum* clade along with *S.echegarayi* and *S.riojense* (Särkinen et al. 2015b) The clade consists of perennial herbs with woody underground rhizomes and slightly thick and fleshy leaves that appear succulent when compared to membranous species of the Morelloid clade. All species of the *Episarcophyllum* clade are distributed in dry habitats in Argentina and neighbouring Chile, generally above 2,000 m elevation.

*Solanumsinuatirecurvum* is similar to *S.riojense* in the floccose pubescence of new growth, but differs in its large yellow berries (always over 1 cm in diameter; Fig. 165D) and flowers (2–2.6 cm in diameter with acute calyx lobe tips versus 1.8–2 cm in diameter with rounded calyx lobe tips in *S.riojense*). Leaves of *S.sinuatirecurvum* are usually smaller and more deeply dissected than those of *S.riojense* but considerable variation exists.

#### ﻿55. Solanum subtusviolaceum

Taxon classificationPlantaeSolanalesSolanaceae

Bitter, Repert. Spec. Nov. Regni Veg. 11: 207. 1912.

Figs 166 , 167

##### Type.

Bolivia. “Yungas”, 22 Aug 1894, M. *Bang 2392* (lectotype, designated here: NY [00172194]; isolectotypes BM [BM000617681], E [E00190737], F [v0073422F, acc. # 163942], G [2 sheets], GH [00077769], K [K000585515], M [M0166060], MO [MO-503622, acc. # 1815481], NY [00172192, 00172193], PH [00030484], US [00650473, acc. # 32986; 00027817, acc. # 1324786], W [acc.# 1895-001067], WIS [v0256269WIS]).

**Figure 166.**
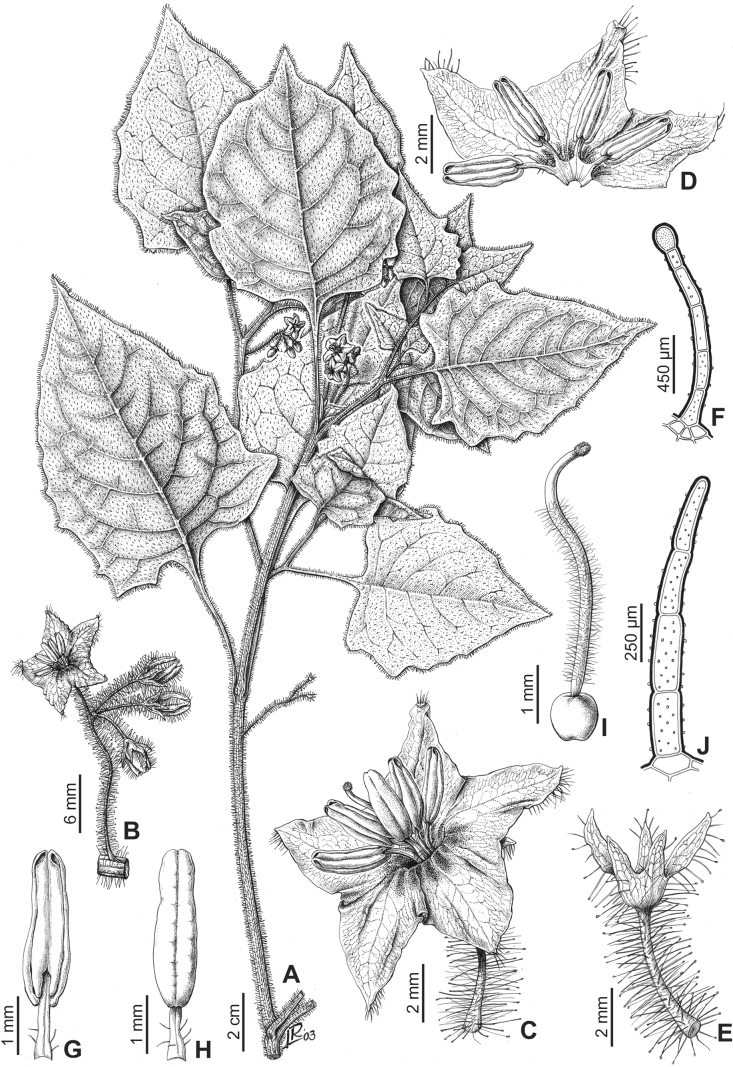
*Solanumsubtusviolaceum***A** flowering branch **B** inflorescence **C** flower at anthesis **D** flower opened **E** calyx **F** glandular trichome **G** stamen adaxial view **H** stamen abaxial view **I** style **J** eglandular trichome (**A–J***Krukoff 10378*). Illustration by L. Ribulgo.

##### Description.

Sprawling herbs to small shrubs to 0.45 m high, the branches erect or somewhat lax. Stems terete, densely pubescent with spreading transparent glandular simple uniseriate ca. 10-celled trichomes 3(4) mm long, the gland single-celled and globose or with more than one cell and slightly elongate; new growth densely pubescent with transparent glandular simple uniseriate trichomes to 4 mm long like those of the stems, these spreading; bark of older stems somewhat glabrescent, brownish red or pale tan. Sympodial units unifoliate or difoliate, the leaves not geminate. Leaves simple and variously irregularly toothed, the blades 2.5–13 cm long, 1.5–9 cm wide, larger on lower branches, ovate or slightly rhomboidal, widest in the lower third, membranous, discolorous; adaxial surfaces moderately and evenly pubescent on veins and lamina with transparent glandular 6–10-celled simple uniseriate trichomes to 3 mm long, these spreading and somewhat weak and collapsing in dry specimens, the glands usually single-celled; abaxial surfaces similarly pubescent with transparent glandular trichomes on veins and lamina, often purplish in both live plants and dried specimens; principal veins 6–8 pairs, drying yellowish or pale green; base truncate then abruptly attenuate and somewhat decurrent onto the petiole; margins irregularly toothed, the teeth to 10 mm long, ca. 7 mm wide, with acute apices, the sinuses rounded, reaching to 1/4 of the way to the midrib; apex acuminate; petioles 1–3.5 cm long, glandular-pubescent like the stems and leaves. Inflorescences opposite the leaves or borne just below the leaf node, unbranched (occasionally forked), 2–3 cm long, with 4–6 flowers in the distal third of the axis, densely pubescent with spreading, transparent glandular 6–10-celled simple uniseriate trichomes to 3 mm long, the glands usually single-celled; peduncle 1.5–2 cm long; pedicels (0.5)0.7–1 cm long, ca. 0.5 mm in diameter at the base, ca. 1 mm in diameter at the apex, tapering, spreading to deflexed at anthesis, densely pubescent with spreading, transparent glandular 6–10-celled simple uniseriate trichomes like the rest of the inflorescence, articulated at the base; pedicel scars 0.5–2 mm apart, more closely spaced distally. Buds ellipsoid to globose-ellipsoid, the corolla barely exceeding the calyx lobes before anthesis. Flowers 5-merous, cosexual (hermaphroditic). Calyx tube 1.5–2 mm long, conical, the lobes (2)2.5–3.5 mm long, ca. 1 mm wide, long-triangular (lobes on type triangular), distinctly different in texture to the tube, densely pubescent with spreading, transparent glandular 6–10-celled simple uniseriate trichomes to 3 mm long, the glands usually single-celled. Corolla (1.5)1.8–2 cm in diameter, white or white tinged or striped with violet, with a darker yellow-green or purple eye (the eye drying dark), stellate, lobed ca. 2/3 of the way to the base, the lobes 4–7 mm long, 2–3 mm wide, triangular, spreading to strongly reflexed at anthesis, adaxially glabrous, abaxially densely papillate at the tips and margins, sparsely pubescent with transparent eglandular simple uniseriate trichomes to 2.5 mm long at lobe tips and along petal midveins. Stamens equal; filament tube minute; free portion of the filaments 1–1.5 mm long, glabrous or with a few tangled transparent eglandular simple uniseriate trichomes adaxially; anthers 3–4 mm long, 1–1.2 mm wide, ellipsoid, yellow, poricidal at the tips the pores lengthening to slits with age. Ovary conical, glabrous; style 5–6(7) mm long, straight, exserted beyond the anther cone, densely papillate and pubescent with eglandular transparent tangled simple uniseriate trichomes to 0.5 mm in the lower 2/3; stigma small capitate, the surface minutely papillate. Fruit a globose berry, 0.7–0.8 cm in diameter, green (immature ?), drying pale whitish grey, the pericarp thin, matte, opaque, glabrous; fruiting pedicels 1–1.2 cm long, ca. 0.75 mm in diameter at the base, ca. 1.5 mm in diameter at the apex, not markedly woody, deflexed or spreading, not persistent; fruiting calyx slightly accrescent, the tube ca. 2 mm long, the lobes to 5 mm long, appressed or the tips spreading. Seeds ca. 20 per berry, ca. 1.5 mm long, ca. 1 mm wide, flattened and teardrop shaped, tan, the surfaces minutely pitted, the testal cells pentagonal in outline or the walls somewhat sinuate. Stone cells 4, scattered through the mesocarp or sometimes 2 more apically (*fide*Bitter 1912c), ca. 0.5 mm in diameter, cream-coloured. Chromosome number not known.

**Figure 167.**
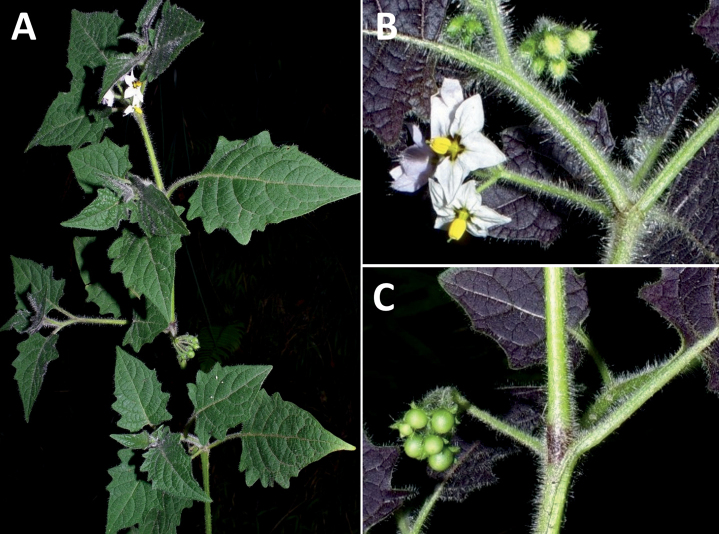
*Solanumsubtusviolaceum***A** habit **B** flowering branch with flowers at full anthesis **C** maturing fruits (**A–C***Orejuela 2833*). Photos by A. Orejuela.

##### Distribution

**Figure 168.**
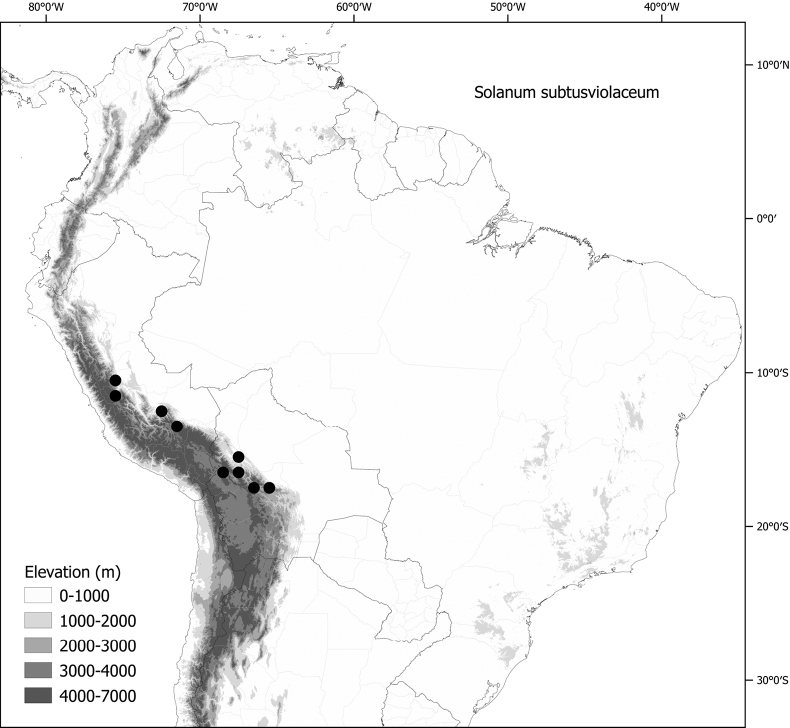
Distribution map of *Solanumsubtusviolaceum*.

**(Fig. 168).***Solanumsubtusviolaceum* occurs along the eastern slopes of the Andes in Peru (Depts. Cusco, Junín, Pasco) and northern Bolivia (Depts. Cochabamba, La Paz).

##### Ecology and habitat.

*Solanumsubtusviolaceum* grows in premontane, montane and cloud forests, at forest gap edges and along roadsides, from 750 to 4,100 m elevation.

##### Common names and uses.

None recorded.

##### Preliminary conservation status

**(IUCN 2022).** Least Concern [LC]. EOO = 163,921 km^2^ [LC]; AOO = 100 km^2^ [EN]. *Solanumsubtusviolaceum* has a broad geographical distribution and is a plant of forest edges and borders. It has been collected in protected areas in Bolivia (Parque Nacional Carrasco) and Peru (Parque Nacional Yanachaga-Chemillén).

##### Discussion.

*Solanumsubtusviolaceum* is one of the glandular-pubescent species from the Andes without accrescent calyces (although *Nee 55287* from Dept. Cochabamba in Bolivia is an aberrant glabrous individual). It is morphologically most similar to *S.juninense*, with which it shares shallowly toothed leaves, long (to 2 mm long) glandular trichomes and corollas with a dark central eye. *Solanumsubtusviolaceum* has longer, more narrowly triangular (2.5–3.5 mm long versus 1.5–2 mm long) calyx lobes and more stone cells per berry (4 versus 1–2) than *S.juninense*, and the inflorescences are usually unbranched (rather than consistently forked) although some specimens of *S.subtusviolaceum* have some forked inflorescences. *Solanumjuninense* has a more northerly distribution in Peru than *S.subtusviolaceum*, which occurs from central Peru to northern Bolivia. Leaves of *S.subtusviolaceum* are often tinged with purple beneath and usually more truncate at the base than those of *S.juninense*. In *S.subtusviolaceum*, the corolla eye is markedly dark in dry material, although this can also be the case in some specimens of *S.juninense*. *Solanumsubtusviolaceum* differs from the lower elevation glandular-pubescent *S.arenicola* in its larger flowers (1.5–2 cm in diameter versus 0.8–1.2 cm in diameter), its more deeply divided calyx and the slightly larger (0.7–0.8 cm in diameter versus 0.3–0.7 cm in diameter) with fewer (ca. 20 versus 35–45) seeds.

Bitter (1912b) cited *Bang 2392* from “herb. Berol.!, Vratisl.!” in the protologue of *S.subtusviolaceum*, also stating that the specimens had been determined as “*S.atriplicifolium* Gill.” We have not found either of these two duplicates of this widely distributed Miguel Bang gathering and so designate the best preserved of the three duplicates of *Bang 2392* at NY (barcode 00172194) as the lectotype for *S.subtusviolaceum*; this sheet bears Bang’s original field label and has both flowers and immature fruits.

#### ﻿56. Solanum tiinae

Taxon classificationPlantaeSolanalesSolanaceae

Barboza & S.Knapp, PhytoKeys 164: 52. 2020.

Figs 2D , 169 , 170

##### Type.

Argentina. Tucumán: Dpto. Tafí del Valle, El Infiernillo, en el parador, 3,042 m, 13 Feb 2012, *G.E. Barboza, S. Knapp & T. Särkinen 3496* (holotype: CORD [CORD00013848]; isotypes: BM [BM001115408, BM001115409], others to be distributed).

**Figure 169.**
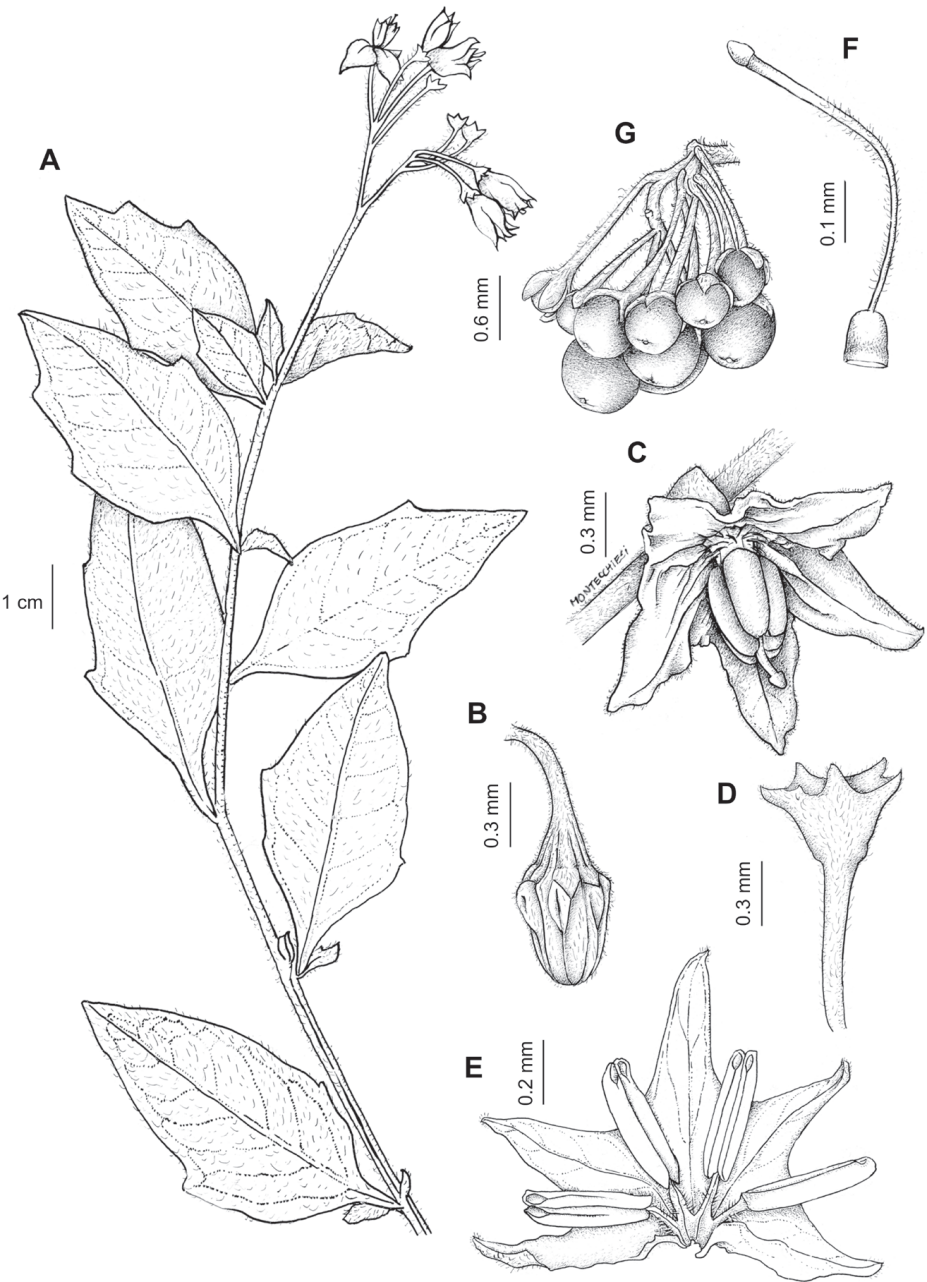
*Solanumtiinae***A** flowering branch **B** flower bud **C** flower **D** calyx **E** dissected flower **F** gynoecium **G** infructescence (**A, C–G***Barboza et al. 3491***B***Barboza et al. 3496*). Illustration by S. Montecchiesi.

##### Description.

Perennial herbs or subshrubs to 0.5 m high, usually sprawling from a woody base. Stems narrowly winged, the wing to 0.5 mm wide, often invested with spinose processes (enlarged trichome bases), sparsely pubescent with appressed, antrorse eglandular, simple uniseriate trichomes, 6–10-celled, ca. 0.5 mm long, these white when dry; new growth densely to moderately pubescent with antrorse eglandular, simple 2–8-celled uniseriate trichomes, ca. 0.5 mm long; bark of older stems pale greenish brown, glabrescent. Sympodial units plurifoliate, the leaves not geminate. Leaves simple, the blades 2–5 cm long, 0.6–2 cm wide, narrowly elliptic to almost lanceolate in some individuals, widest at the middle, membranous, concolorous; adaxial surfaces sparsely and evenly pubescent with antrorse eglandular simple 2–4-celled uniseriate trichomes to 0.5 mm long, the trichomes slightly longer on the veins, white when dry; abaxial surfaces with similar, but denser eglandular antrorse pubescence; principal veins 4–6 pairs, drying yellow, especially abaxially; base attenuate and decurrent onto the winged stem and the leaves sessile or nearly so; margins entire or with a few teeth ca. 2 mm long, ca. 2 mm wide with blunt tips in the lower third to half; apex acute to slightly blunt-tipped; petiole absent to 0.2 mm long, eglandular pubescent like the stems and leaves. Inflorescences opposite the leaves or internodal, forked with 2 short branches, 2.5–5 cm long, with 10–20 flowers clustered at the tips of the inflorescence branches, sparsely pubescent with antrorse eglandular simple uniseriate trichomes like those of the stems; peduncle 1.2–2.5 cm long; pedicels 0.8–1 cm long, ca. 0.5 mm in diameter at the base, ca. 1 mm in diameter at the apex, strongly tapering, spreading to somewhat deflexed at anthesis, sparsely to moderately pubescent with antrorse eglandular simple uniseriate trichomes like the rest of the inflorescence, articulated at the base; pedicel scars clustered at the tips of the inflorescence branches, ca. 0.5 mm apart. Buds ellipsoid to somewhat turbinate (widest in lower third), the corolla strongly exserted from the calyx tube before anthesis, the style sometimes exserted from the bud before anthesis. Flowers 5-merous, cosexual (hermaphroditic). Calyx tube 1.5–2 mm long, conical, the lobes (0.5)1–2 mm long, deltate with lanceolate tips, the sinuses rounded, sparsely pubescent with antrorse eglandular trichomes like the pedicels. Corolla 1.2–2.2 cm in diameter, white, pale violet or white tinged with violet, sometimes changing colour through anthesis, with a brownish yellow to yellow-green central star edged with brownish purple, stellate, lobed halfway to the base, the lobes 5–8 mm long, 4–5 mm wide, deltate to triangular, spreading or slightly reflexed at anthesis, adaxially glabrous, abaxially densely pubescent with eglandular papillae and simple uniseriate trichomes to 0.2 mm long. Stamens equal; filament tube minute; free portion of the filaments 0.5–1 mm long, adaxially densely pubescent with tangled transparent simple uniseriate trichomes; anthers 4–5 mm long, 1–1.25 mm wide, ellipsoid, yellow, the abaxial surfaces occasionally papillate, poricidal at the tips, the pores lengthening to slits with age. Ovary conical, glabrous; style 7–10 mm long, straight, more or less long-exserted beyond the anther cone, pubescent along almost the entire length, more densely in the lower half with tangled transparent simple trichomes to 0.5 mm long; stigma capitate to clavate, bright green in live plants, the surface minutely papillose. Fruit a globose berry, 0.8–0.9 cm in diameter, green with tiny white spots (immature?), the pericarp thin, matte, opaque, glabrous; fruiting pedicels 0.8–1 cm long, ca. 0.75 mm in diameter at the base, ca. 1.5 mm in diameter at the apex, thickened but not woody, strongly deflexed with a distinct bend at the pedicel base, persistent; fruiting calyx not enlarged or accrescent, the lobes appressed to the surface of the berry. Seeds 10–30 per berry, 1.7–2 mm long, 1–1.5 mm wide, not markedly flattened, teardrop shaped with an apical hilum, pale tan, the surfaces minutely pitted, the testal cells sinuate in outline. Stone cells 4–9 per berry, 0.7–1.5 mm in diameter, 2 usually larger than the rest. Chromosome number: n = 12 (Moscone 1992, as S.lorentziiBittervar.montigenum C.V.Morton).

**Figure 170.**
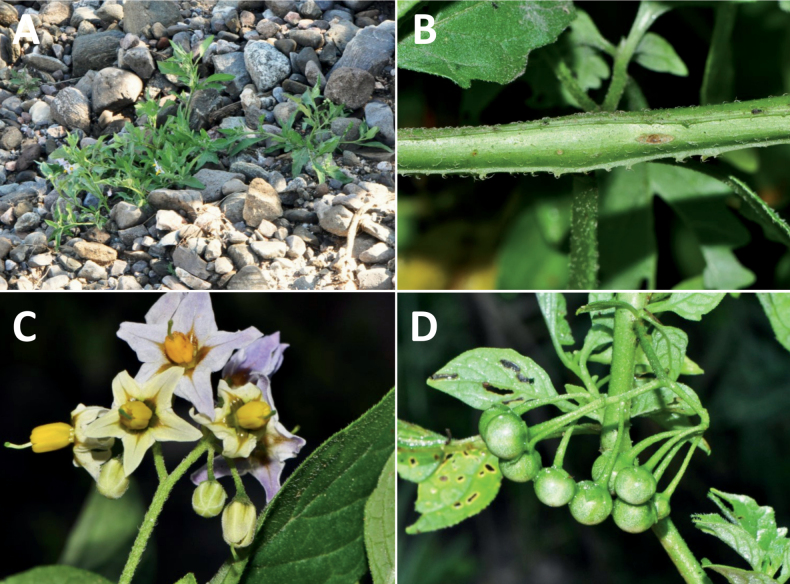
*Solanumtiinae***A** habit **B** stem with spinose processes and enlarged trichome bases **C** inflorescence with flowers and buds **D** maturing fruits **E** flower at anthesis **F** mature and nearly mature berries showing strongly deflexed pedicels (**A–F***Barboza et al. 3491*). Photos by S. Knapp. **A–D** previously published in Knapp et al. (2020: 53).

##### Distribution

**Figure 171.**
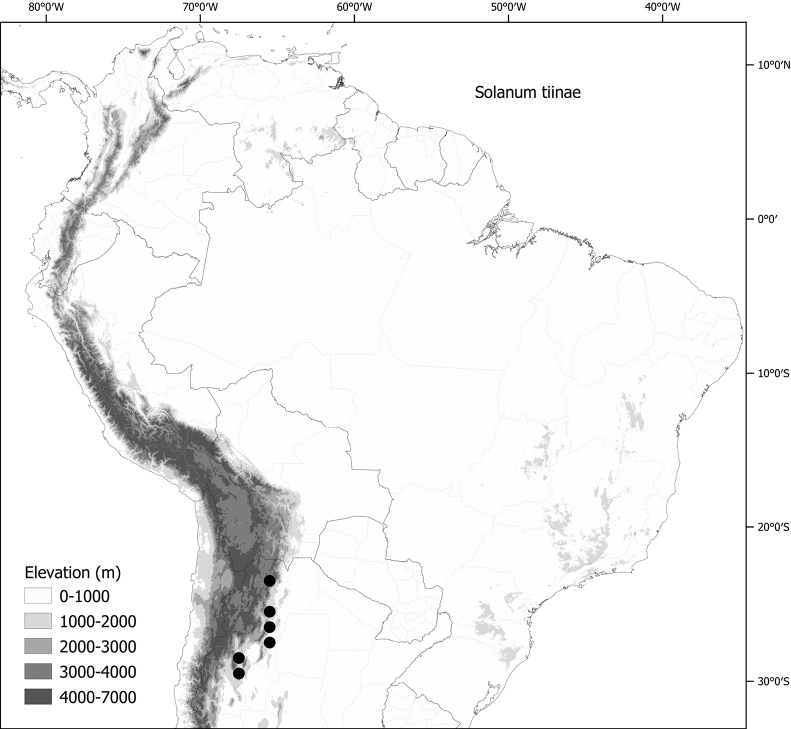
Distribution map of *Solanumtiinae*.

**(Fig. 171).***Solanumtiinae* is endemic to Argentina (Provs. Jujuy, Salta, Tucumán) with most collections from the area around the type locality at El Infiernillo. The distribution coincides with the Jujuy-Tucumán area of endemism of Aagensen et al. (2012).

##### Ecology and habitat.

*Solanumtiinae* grows among rocks and in open areas in pre-puna habitats in the Andes, from 2,400 to 4,000 m elevation.

##### Common names and uses.

None recorded.

##### Preliminary conservation status

**(IUCN 2022).** Near Threatened [NT]. EOO = 40,977 km^2^ [LC]; AOO = 84 km^2^ [EN]. Most collections of *S.tiinae* are from a very few commonly visited localities and the main road between Tafí del Valle and Amaicha del Valle in the Province of Tucumán. *Solanumtiinae* to date has not been collected in protected areas; based on the number of localities (ca. 5), the area of occupancy and the extent of occurrence it may be of conservation concern. Knapp et al. (2020) assessed it as Vulnerable. Where it occurs *S.tiinae* is not common or weedy, although it does grow in open areas.

##### Discussion.

*Solanumtiinae* is often identified in herbaria as *S.aloysiifolium* (and its synonyms, see Barboza et al. 2013) or *S.cochabambense*. It is similar to those species in its forked inflorescence with a long peduncle, but differs from *S.aloysiifolium* in its larger, less deeply stellate purple or purplish cream (rather than usually white) corollas, and from *S.cochabambense* in its smaller habit and winged stems. The strongly antrorse pubescence of *S.tiinae* is distinctive and not found in either *S.aloysiifolium* or *S.cochabambense*.

Cabrera (1983) cited specimens of what we recognise as *S.tiinae* as *S.bangii*, a synonym of *S.gonocladum* of high elevation Bolivia and southern Peru. Both these species are subshrubs that are markedly woody at the base with anthers ca. 5 mm long, but *S.tiinae* differs from *S.gonocladum* in its deltate calyx lobes with lanceolate tips (versus spathulate in *S.gonocladum*), capitate to clavate stigma (versus large capitate in *S.gonocladum*) and in its strongly antrorse stem pubescence (versus spreading in *S.gonocladum*).

*Solanumtiinae* also resembles the highly variable species *S.salicifolium*, from which it can be distinguished by its shorter (1–2 mm versus 2.5–3 mm long) calyx lobes, the appressed strongly antrorse pubescence (Fig. 170B), the strictly forked (versus only occasionally once-branched) inflorescences with more flowers (10–20 versus 4–10) and the calyx lobes (Fig. 170F) that are tightly appressed to the berry (versus spreading and slightly recurved in *S.salicifolium*, Fig. 153C). These two species have been collected growing side by side in the same habitat (e.g., *Barboza et al. 3491*, *S.tiinae* and *Barboza et al. 3494*, *S.salicifolium* from km 92 on the Amaicha del Valle to Tafí del Valle road) and can be easily distinguished in the field using corolla shape – those of *S.salicifolium* are deeply stellate with relatively narrow lobes (Fig. 153D), while those of *S.tiinae* are less deeply and more broadly lobed (Fig. 170C).

#### ﻿57. Solanum triflorum

Taxon classificationPlantaeSolanalesSolanaceae

Nutt., Gen. N. Amer. Pl. 1: 128. 1818.

**Figure 172.**
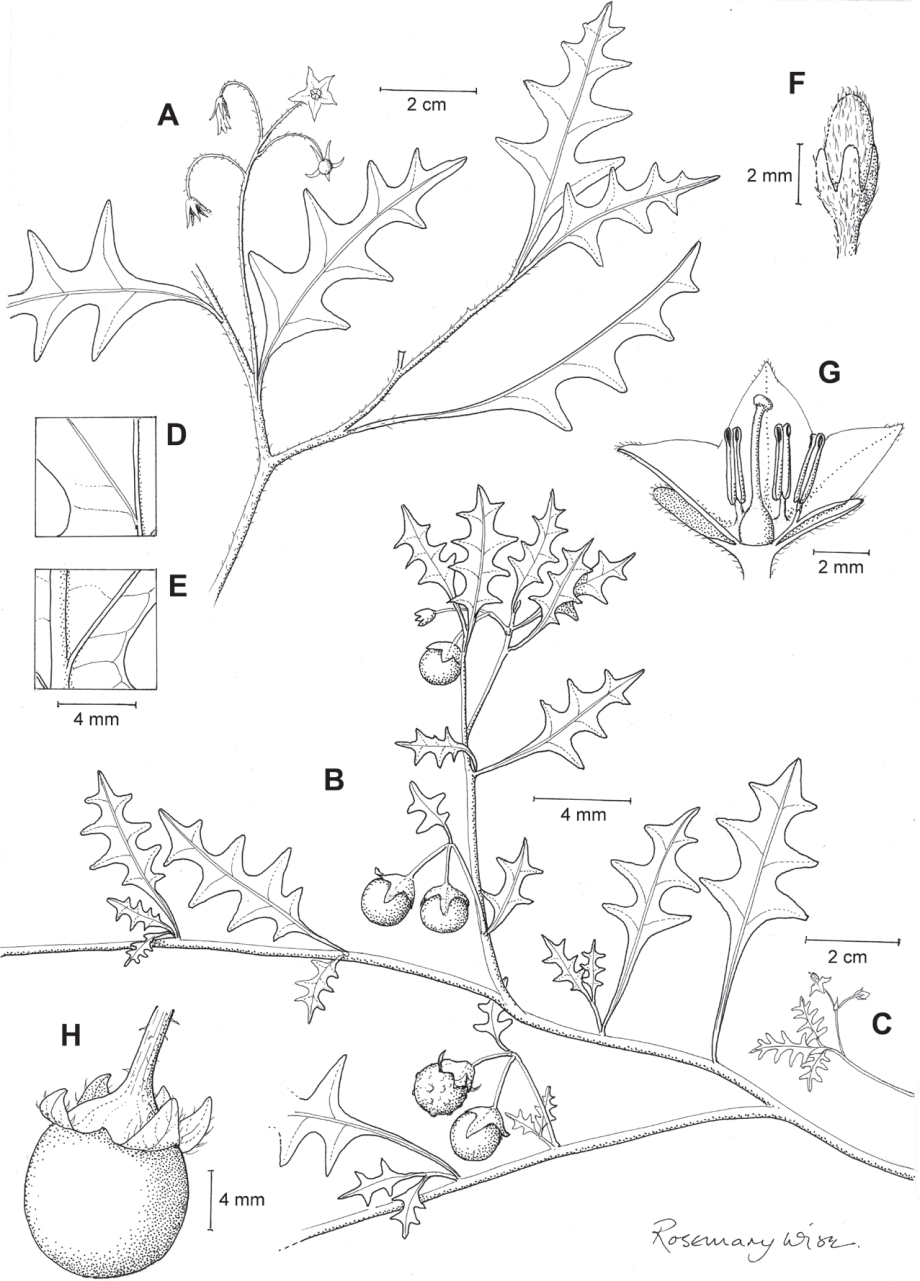
*Solanumtriflorum***A** flowering habit **B** fruiting habit **C** flowering branch **D** detail of adaxial leaf surface **E** detail of adaxial leaf surface **F** flower bud **G** flower **H** fruit (**A, C, F, G***Donat 55***B, D, E, H***Baker 577*). Illustration by R. Wise. Previously published in Särkinen et al. (2018: 169) and Knapp et al. (2019: 118).

**Figure 173.**
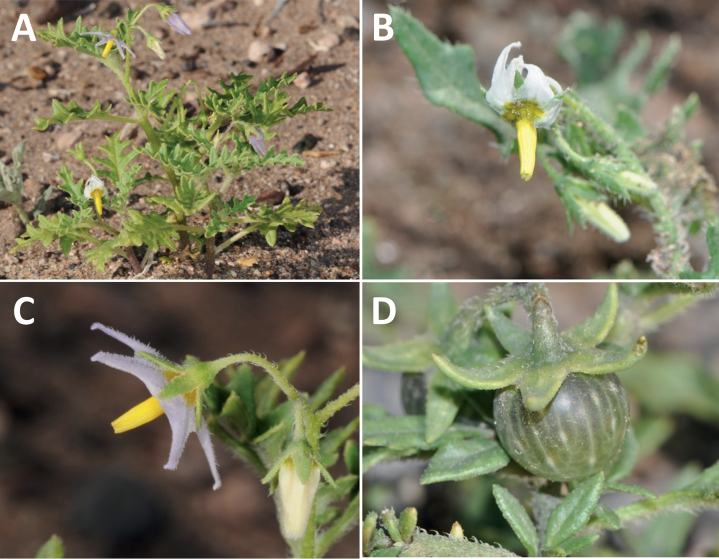
*Solanumtriflorum***A** habit **B** flowering habit **C** flower bud and flower **D** flower (**A***Sérsic 5040***B, D** Knapp et al. 10488 **C***Barboza et al. 2345*). Photos by G. Barboza, S. Knapp and A. Sérsic. Partly previously published in Särkinen et al. (2018: 170) and Knapp et al. (2019: 119).

Figs 4I , 172 , 173 Types based on American specimens only; for full synonymy, see Särkinen et al. (2018: 167–168)

Solanum triflorum Nutt. var. majus Hook., Fl. Bor.-Amer. 2: 90. 1837, as “*major*”. Type. Canada. Saskatchewan: “Carleton House Fort, Saskatchewan River”, *J. Richardson s.n.* (lectotype, designated by Särkinen et al. 2018, pg. 167: BM [BM000934745]; isolectotype: K [K001159656, large plants]).
Solanum triflorum Nutt. var. minus Hook., Fl. Bor.-Amer. 2: 90. 1837, as “*minor*”. Type. Canada. Saskatchewan: “In the Garden (a weed) of Carleton House Fort, entrance of Badger’s Hole, and Saskatchewan River to Edmonton House” [protologue], *T. Drummond s.n.* (lectotype, designated by Särkinen et al. 2018, pg. 167: E [E00526685]; isolectotypes: BM [BM000934744], K [K001159656]).
Solanum mendocinum Phil., Anales Univ. Chile 21(2): 403. 1862. Type. Argentina. Mendoza: Mendoza, 1860–1861, *W. Díaz s.n.* (lectotype, designated by Hunziker 1989, pg. 184 [superfluously by Barboza et al. 2013, pg. 260]: SGO [SGO000004580, acc. # 055499]).
Solanum calophyllum Phil., Anales Univ. Chile 21(2): 403. 1862. Type. Argentina. Mendoza: Mendoza, 1860–1861, *R. Philippi s.n.* (lectotype, designated by Hunziker 1989, pg. 184 [superfluously by Särkinen et al. 2018, pg. 167; cited as holotype in Barboza et al. 2013]: SGO [SGO000004552]; isolectotype: G [G00343450]).
Solanum pyrethrifolium Griseb., Abh. Königl. Ges. Wiss. Göttingen 24: 250. 1879. Type. Argentina. Tucumán: Lules, Dec 1873, *P. G. Lorentz & G. Hieronymus 1132* (lectotype, designated by Morton 1976, pg. 102: CORD [CORD00006111]; isolectotype: GOET [GOET003594]).
Solanum gaudichaudii Dunal var. pyrethrifolium (Griseb.) Kuntze, Revis. Gen. Pl. 3(3): 226. 1898. Type. Based on Solanumpyrethrifolium Griseb.
Solanum triflorum Nutt. var. calophyllum (Phil.) Bitter, Abh. Naturwiss. Vereine Bremen 23: 144. 1914. Type. Based on Solanumcalophyllum Phil.
Solanum triflorum Nutt. var. pyrethrifolium (Griseb.) Bitter ex Probst, Mitteil. Naturfor. Gesellsch. Solothurn 9: 41. 1932. Type. Based on Solanumpyrethrifolium Griseb.

##### Type.

United States of America. North Dakota [McLean County]: Near Fort Mandan, *Anon. [Lewis & Clark] s.n.* (lectotype, designated by Hunziker 1989, pg. 189 [superfluously by Barboza et al. 2013, pg. 260]: PH [00030496]).

##### Description.

Annual herbs to 0.4 m high, much branched at the base, to 0.7 m in diameter. Stems terete, green, decumbent and prostrate, forming adventitious roots at the nodes, not markedly hollow; new growth glabrous to sparsely pubescent with eglandular simple, uniseriate (3–)4–10-celled spreading trichomes 0.5–2 mm long, occasionally with a few glandular trichomes with a 1-many-celled apical gland; older stems glabrescent. Sympodial units difoliate or trifoliate, the leaves not geminate. Leaves simple and shallowly lobed to deeply pinnatifid, the blades (1–)2–4(–5) cm long, 0.2–2.9 cm wide, narrowly elliptic to oblong or ovate-elliptic, widest in the lower half, membranous to somewhat fleshy, discolorous; adaxial surface glabrous to sparsely pubescent with simple, uniseriate trichomes like those on stem, scattered along lamina and more densely along the veins; abaxial surface more densely pubescent on veins and lamina; major veins 3–6 pairs, not clearly evident abaxially; base cuneate, decurrent on the petiole; margins almost entire to sinuate-lobate to deeply pinnatifid to near-pinnate, with 3–6 linear to triangular pairs of lobes; apex acute; petioles (0.5–)1–2(–2.4) cm long, pubescent with simple uniseriate trichomes like those of the stems. Inflorescences internodal, unbranched, 1–2 cm long, with 1–5(–6) flowers clustered near the tips (sub-umbelliform), glabrous to sparsely pubescent with spreading trichomes like those of the stems; peduncle 0.8–3.5 cm long, often with apical leafy “bracteoles” (small, leaf-like structures amongst the pedicels); pedicels 3–12 mm long, 0.4–0.5 mm in diameter at the base and 0.4–0.5 mm in diameter at the apex, straight and spreading, articulated at the base; pedicel scars spaced 0(-0.5) mm apart. Buds narrowly ellipsoid or occasionally narrowly ovoid, the corolla exserted 1/5–2/5 from the calyx tube before anthesis. Flowers 5-merous, cosexual (hermaphroditic). Calyx tube 1–1.5 mm long, conical, the lobes 2.5–3.5(–7) mm long, 0.8–1(–4) mm wide, triangular-oblong with acute apices, densely pubescent with simple, uniseriate eglandular trichomes like those of the stem. Corolla 1–1.4 cm in diameter, white to lilac with a yellow-green central eye with black-purple colouration at the base, deeply stellate, lobed halfway to 3/4 of the way to the base, the lobes 4–5 mm long, 1.8–2.2 mm wide, reflexed at anthesis, densely pubescent abaxially with short simple uniseriate eglandular trichomes like those on stems and leaves. Stamens equal; filament tube minute; free portion of the filaments 0.6–1 mm long, adaxially sparsely pubescent with tangled simple, uniseriate trichomes; anthers 2.8–3.1(–4) mm long, 0.4–0.5 mm wide, narrowly ellipsoid, pale yellow, poricidal at the tips, the pores lengthening to slits with age and drying. Ovary globose, glabrous; style 2.5–3.5 mm long, straight, not exserted beyond the anther cone, densely pubescent with 2–3-celled simple uniseriate trichomes to 1/2 from the base; stigma capitate, minutely papillate, green in live plants. Fruit a globose berry, 0.8–1(–2) cm in diameter, dark green at maturity, the pericarp thin, usually shiny, opaque, glabrous; fruiting pedicels 12–17 mm long, 0.5–1 mm in diameter at the base, 1–1.5 mm in diameter at the apex, spaced 0–0.5(–1) mm apart, reflexed and becoming woody, not persistent; fruiting calyx somewhat accrescent in fruit, but not becoming papery nor covering the berry, the tube 2.5–3 mm long, the lobes (4–)4.5–5.5(–8) mm long and 2.2–3.5 mm wide, strongly reflexed to spreading. Seeds 40–60 per berry, 2–2.5 mm long, 1.7–2 mm wide, subglobose, yellow, the surfaces minutely pitted, the testal cells pentagonal in outline. Stone cells 13–30, 1–1.5 mm in diameter, creamy white or pale tan. Chromosome number: *n* = 12 (Moyetta et al. 2013, voucher *Chiapella et al. 1839*).

##### Distribution

**Figure 174.**
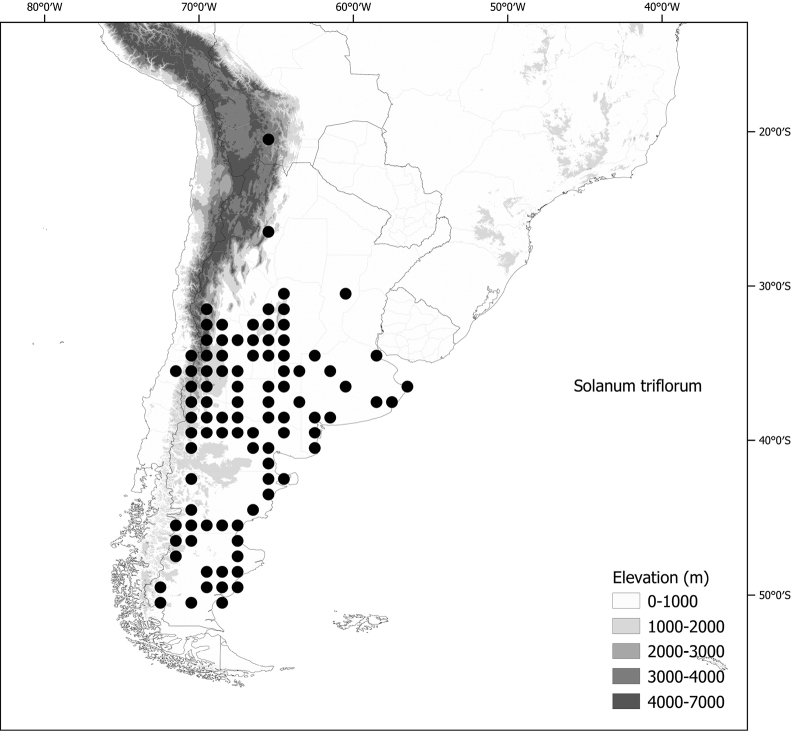
Distribution map of *Solanumtriflorum* in South America. For distribution in North America and the Eastern Hemisphere, see Knapp et al. (2019: 120) and Särkinen et al. (2018: 171), respectively.

**(Fig. 174).***Solanumtriflorum* is native to the Americas with a disjunct (amphitropical) distribution between temperate South and North America (see Knapp et al. 2019). In South America it is only known from Argentina (Provs. Buenos Aires, Chubut, Córdoba, La Pampa, Mendoza, Neuquén, Río Negro, San Juan, San Luis, Santa Cruz), largely in Patagonia. The species has been introduced outside its native range in temperate areas of Europe, South Africa and Australia (see Särkinen et al. 2018).

##### Ecology and habitat.

*Solanumtriflorum* shows broad ecological lability, growing along roadsides, sandy soils, in cultivation, and in salt plains (salinas) between (0-)700 and 2,900 m elevation..

##### Common names and uses.

Argentina. Córdoba: meloncillo (*Kurtz 4612*). In North America berries eaten in times of famine and used medicinally (see Knapp et al. 2019); no uses have been recorded from South American specimens.

##### Preliminary conservation status

**(IUCN 2022).** Least Concern [LC]. EOO = 92,225,775 km^2^ [LC]; AOO = 3,708 km^2^ [EN]; calculated on global range. *Solanumtriflorum* is weedy and common where it occurs (Särkinen et al. 2018, Knapp et al. 2019). In Patagonia it is common along roads and in highly disturbed sites.

##### Discussion.

*Solanumtriflorum* is a distinctive species with a prostrate habit, fleshy, usually pinnatifid, leaves, and deeply stellate flowers with long, thin anthers. The inflorescences usually have a small bracetole at the apex and berry size varies from small (ca. 10 mm) to very large (ca. 20 mm), but usually a given plant has either small or large berries. Numerous stone cells are found in the berries, sometimes almost outnumbering seeds, and large berries can have as many as 30 stone cells. *Solanumtriflorum* is difficult to confuse with any other morelloid solanum. It was thought to be related to members of the Radicans clade based on morphology (Child 1994) but molecular data refute this and place the species as the first branching species of the Black nightshade clade (sensu Särkinen et al. 2015b).

Leaf shape can be quite variable in *S.triflorum* although not within individual plants. Most plants have deeply dissected leaves, but some (e.g., *Knapp et al. 10488* and *Kurtz 5534b* from Prov. Mendoza, Argentina, *Chiapella et al. al. 1809* from Prov. Neuquén, Argentina) have leaves that are only shallowly toothed. The glandular trichomes reported on leaves of *S.triflorum* (Subils 1989) are very sparse and never give the plants a viscid, sticky feel.

*Solanumtriflorum* has a classic American Amphitropical Distribution (Gray and Hooker 1880; Raven 1963; AAD sensu Simpson et al. 2017), with populations occuring in North and South America, but not between (see also *S.nitidibaccatum*). Due to its weedy nature, it is often assumed to be introduced to North America (see discussion in Knapp et al. 2019), but the amphitropical distribution pattern is found in other Solanaceae native to both regions such as *Lycium* L. (Levin et al. 2007) and groups of solanums such as the Carolinense (subsection Lathyrocarpum G.Don, Wahlert et al. 2015, as “section”) and Elaeagnifolium (Knapp et al. 2017) clades. *Solanumelaeagnifolium* Cav. (Elaeagnifolium clade, Knapp et al. 2017) has an almost identical amphitropical distribution (AAD sensu Simpson et al. 2017), and is similarly weedy; it has also been assumed to be introduced to North America. Distribution of these disjunct group is more likely to be the result of long distance dispersal than of vicariance (Guilliams et al. 2017), with dispersal after being eaten and passing through an animal’s gut (endozoochory) being less common than disperal via attachment to an animal fur or feathers (epizoochory) (Schenk and Saunders 2017). In the case of Solanaceae, soft juicy berries make endozoochory more likely as a distribution mechanism, although there is no information on frugivores or fruit dispersal for *S.triflorum*. The distribution of *S.triflorum* in temperate areas, but also at higher elevations in deserts and into the more boreal regions of North America places it in the temperate AAD category of Simpson et al. (2017); annuals like *S.triflorum* predominate in this category. Amongst temperate AAD species the most common direction for distribution is from North to South America, but we suspect that like Verbenaceae (Frost et al. 2017) and *Lycium* (Levin et al. 2007), most *Solanum* disjunctions will have a South America to North America directionality. To date, only North American populations of *S.triflorum* have been included in molecular phylogenetic studies (Särkinen et al. 2015b).

Hunziker (1989) inadvertently lectotypifed (sensu Prado et al. 2015) *S.triflorum* in a note added in proof referring to a photocopy of the holotype from PH. Barboza et al. (2013) and Särkinen et al. (2018) superfluously lectotypified the synonyms *S.mendocinum* and *S.calophyllum* respectively; these names had also been inadvertently lectotypified by Hunziker (1989) via citation of a single specimen in a single herbarium.

#### ﻿58. Solanum tripartitum

Taxon classificationPlantaeSolanalesSolanaceae

Dunal, Prodr. [A. P. de Candolle] 13(1): 72. 1852.

Figs 4A , 175 , 176

Solanum quadripartitum Dunal, Prodr. [A. P. de Candolle] 13(1): 72. 1852. Type. Bolivia. Circa Miraflor, *A.D.’Orbigny 1346* (holotype: P [P00369233]; isotype: MPU [MPU830309]).

##### Type.

Bolivia. La Paz: La Paz, 1842, *A. D’Orbigny 1537* (lectotype, designated by Barboza et al. 2013, pg. 261: P [P00445317]; isolectotypes: BR [BR0000005570737], G [G00343534], P [P00445318, P00445319], W [acc. # 1889-127572]).

**Figure 175.**
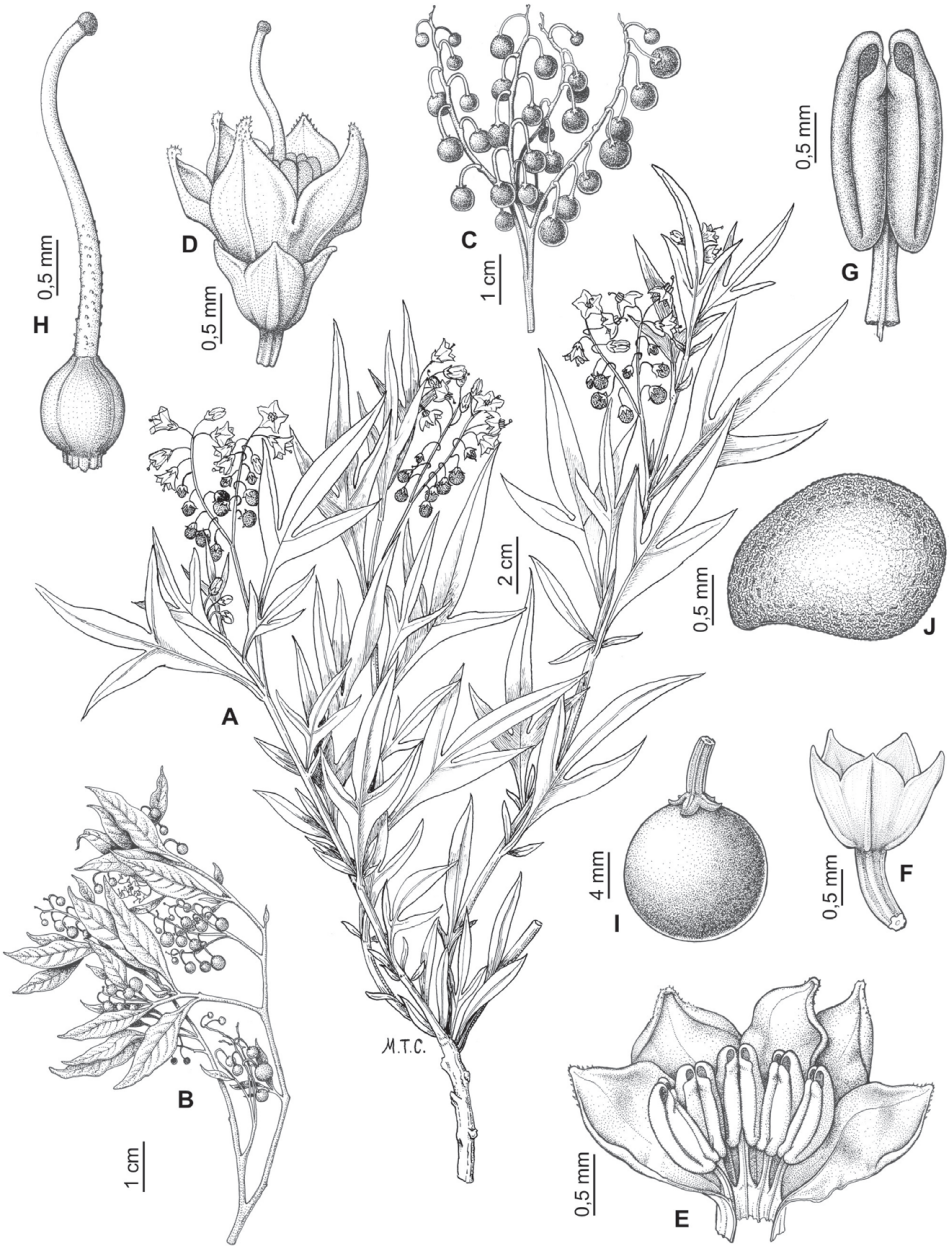
*Solanumtripartitum***A** habit **B** habit of simple leaved population from Salta, Argentina **C** infructescence **D** flower **E** flower opened **F** calyx **G** stamen, lateral view **H** gynoecium **I** fruit **J** seed (**A–F***Cabrera 19880*). Illustration by M.T. Cabrera. Previously published in Barboza et al. (2013: 261).

##### Description.

Erect to spreading or decumbent perennial herbs or subshrubs, occasionally prostrate, if erect to 1.2 m high with several branches, the stems generally not rooting even where in contact with the soil, but sometimes rooting at the lowermost nodes. Stems terete or with small angles from the decurrent leaf bases, completely glabrous to sparsely pubescent with simple uniseriate eglandular trichomes to 0.4 mm long from the ciliate lower margin of the petiole; new growth glabrous or moderately pubescent with simple uniseriate eglandular trichomes to 0.4 mm long, these denser near the stems; bark of older stems pale greenish grey. Sympodial units difoliate, the leaves not geminate. Leaves deeply lobed or occasionally simple, the blades (2.5)3.5–11 cm long, (2.5)3–8 cm wide, broadly elliptic to ovate, widest at the middle or in the lower half, membranous to chartaceous, concolorous; adaxially and abaxially glabrous; principal veins 2(–4) pairs, the terminal leaf lobe with an additional 2–4 pairs of veins; base long-attenuate; margins occasionally entire (some populations in Salta, Argentina), more often lobed nearly to the midrib, the lobes usually 3, occasionally 5, rarely one of the lateral lobes with minute secondary lobes, the terminal lobe lanceolate, the lateral lobes asymmetrically lanceolate with more laminar tissue basiscopically, all narrower at the base and widest at the middle, the lobe tips acute; apex acute to slightly rounded; petioles 0.5–1.5 cm long, 1/10 to 1/5 the length of the blades, winged to the base, glabrous or sometimes sparsely ciliate with simple uniseriate trichomes near the base. Inflorescences extra-axillary, often just above the bifurcation of a stem, forked or many-branched, (1.5–) 2–4(–7) cm long, with 5–10 flowers per branch, glabrous or rarely minutely puberulent; peduncle 0.5–1 (–1.2) cm; pedicels 2–5 mm long, occasionally slightly angled at the apex from the base of the calyx, deflexed and nodding at anthesis, articulated at the base; pedicel scars irregularly spaced 2–4 mm apart. Buds elliptic to obelliptic, the corolla strongly exserted from the calyx tube before anthesis, buds purple-tinged to dark purple. Flowers 5-merous, cosexual (hermaphroditic). Calyx tube 1–1.5 mm long, cup-shaped and abruptly narrowing to the pedicel, the lobes 0.5–1.2 mm long, 0.6–1 mm wide, triangular with obtuse to acute tips, glabrous or rarely minutely puberulent, the sinuses somewhat scarious. Corolla 0.9–1.1 cm in diameter, shallowly stellate, white, violet or light violet, with a pale green or yellowish green central eye, lobed ca. halfway to the base, the lobes 2–3.5 mm long, 2–3 mm wide, broadly triangular, spreading, adaxially glabrous, abaxially minutely white-puberulent at least on the tips. Stamens equal; filament tube minute; free portion of the filaments 0.2–0.6 mm long, pubescent with tangled simple uniseriate trichomes abaxially; anthers 1.8–2.3 mm long, 0.5–0.9 mm wide, elliptic-oblong and wider in the distal third, somewhat connivent, yellow, poricidal at the tips, the pores lengthening to slits with age. Ovary conical, glabrous; style 3–5 mm long, straight, exserted beyond the anther cone, glabrous or minutely puberulent near the base; stigma capitate, the surfaces minutely papillate, pale green in live plants. Fruit a depressed-globose, flattened berry, 0.6–0.7 cm in diameter, markedly bilobed when immature, nearly globose when ripe, passing from green to orange to red when fully ripe, the pericarp thin, shiny, opaque, glabrous; fruiting pedicels 0.7–0.8 cm long, ca. 0.5 mm in diameter at the base, ca. 1 mm in diameter at the apex, strongly recurved at the base to hold the fruit downwards, but always well above the soil level, not persistent; fruiting calyx not accrescent, appressed to the berry surface or the tips slightly reflexed, not enlarging from size in flower. Seeds ca. 40 per berry, 1.4–2 mm long, 1.3–1.5 mm wide, flattened-reniform, light yellow or pale tan-brown, the surfaces minutely pitted, the testal cells sinuate in outline. Stone cells 2(-6) per berry, 2 of these apically positioned and 2–2.2 mm in diameter, occasionally with 1–4 additional smaller stone cells ca. 0.5 mm in diameter scatted throughout berry, all pale cream. Chromosome number: 2n = 24 (Acosta et al. 2005, voucher *Hunziker et al. 24745*).

**Figure 176.**
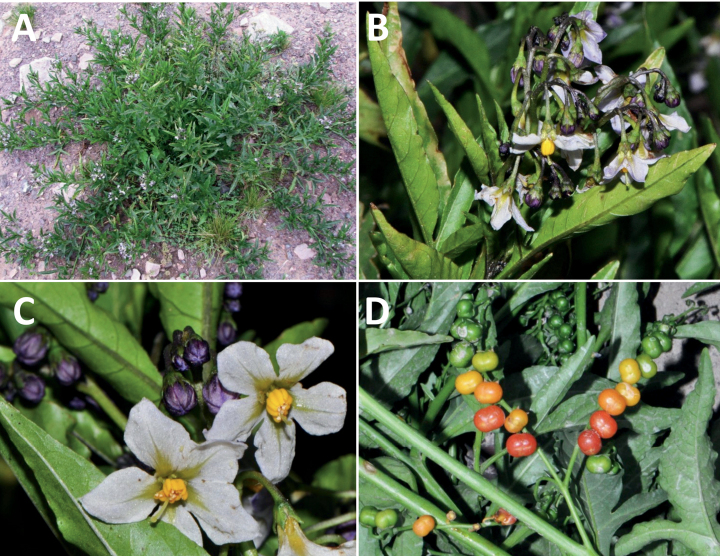
*Solanumtripartitum***A** habit **B** flowering branch **C** flowers at full anthesis **D** maturing fruits (**A–C***Barboza et al. 3561***D***Barboza et al. 3563*). Photos by S. Knapp.

##### Distribution

**Figure 177.**
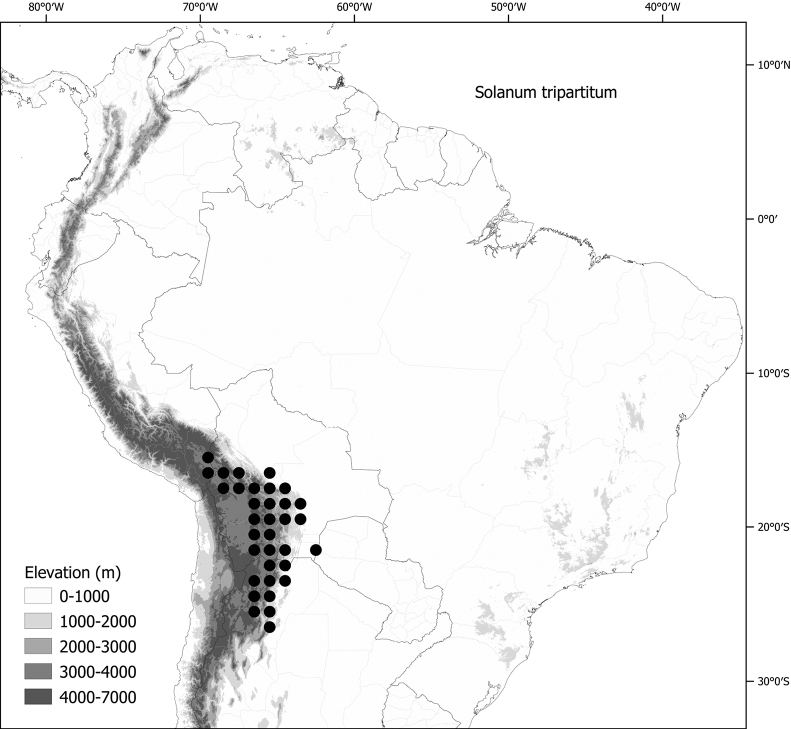
Distribution map of *Solanumtripartitum*.

**(Fig. 177).***Solanumtripartitum* is known from northern Argentina (Provs. Jujuy, Salta) and Bolivia (Depts. Chuquisaca, Cochabamba, La Paz, Oruro, Potosí, Santa Cruz, Tarija).

##### Ecology and habitat.

*Solanumtripartitum* occurs on steep stony hillsides with low, rather poor vegetation with scattered shrubs and along roadsides in gravelly areas and in association with disturbed ground near habitations, from 600 to 4,270 m elevation.

##### Common names and uses.

Argentina. Jujuy: mora mora (*Giberti et al. s.n.*, *Arenas et al. 822*, *Claren 11548*), ñusco (*Burkart et al. s.n.*, *Claren 11730*, *Ambrosetti 38*, *Budin s.n.*, *Hunziker 1290*), tomatillo (*Biloni 6555*). Bolivia. Chuquisaca: ñuschuchu (*Barboza 84 bis*). In Argentina (Prov. Jujuy) the entire plant (excluding the root) is used medicinally (Hurrel 1991; Lupo and Echenique 1997); the flowers are also reported to be ornamental (Lupo and Echenique 1997).

##### Preliminary conservation status

**(IUCN 2022).** Least Concern [LC]. EOO = 410,690 km^2^ [LC]; AOO = 564 km^2^ [EN]. *Solanumtripartitum* is a weedy plant of open areas and has a wide distribution. It has been collected in protected areas in Argentina (e.g., Humahuaca World Heritage site) and in the area of Parque Nacional Carrasco in Bolivia.

##### Discussion.

*Solanumtripartitum* is a member of the Radicans clade (Särkinen et al. 2015b), together with *S.corymbosum*, *S.palitans* and *S.radicans*. It is largely sympatric with and often is found growing with *S.palitans* in the same sort of weedy habitats. Michael Nee (pers. comm.) has suggested they hybridise in Bolivia (see discussion of *S.palitans*). Both taxa have deeply lobed leaves with mostly three leaflets, although *S.tripartitum* occasionally has five leaflets. Poorly prepared herbarium specimens and those in young flowering condition can be difficult to identify; mixed collections are common. In the field the two species are distinct, with *S.tripartitum* being an upright plant, the base often decumbent but not rooting, and with erect and branched inflorescences and red ripe berries, while *S.palitans* is a prostrate plant, rooting at the nodes, and with the simple inflorescences holding the yellow-orange berries at the surface of the soil.

Variation in leaf and inflorescence morphology is very local in *S.tripartitum*. In the Department of Potosí (Bolivia) leaflets are very narrow and the inflorescences are usually many times branched, and in the area of Salta (Argentina) a population is often collected that has undivided leaves (e.g., *Varela & del Castillo 1332*; Fig. 175B). In other parts of the range the basal-most divisions of leaves of *S.tripartitum* are occasionally further divided into secondary lobes.

#### ﻿59. Solanum tweedieanum

Taxon classificationPlantaeSolanalesSolanaceae

Hook., Bot. Mag. 62: tab. 3385. 1835, as “Tweedianum”.

Figs 178 , 179

Solanum atriplicifolium Gillies ex Nees, Nov. Act. Acad. Caes. Leop. 19, Suppl. 1: 386. 1843. Type. Argentina. Mendoza: El Diamante, [no date], *J. Gillies s.n.* (lectotype, designated by Barboza et al. 2013, pg. 239): E [E00112916]; isolectotypes: E [E00057545], K[K000585737], NY [00139057]).
Solanum nigrum L. subsp. atriplicifolium (Gillies ex Nees) Sendtn., Fl. Bras. (Martius) 10: 17. 1846. Type. Based on S.atriplicifolium Gillies ex Nees.
Solanum haarupii Bitter, Repert. Spec. Nov. Regni Veg. 11: 210. 1912. Type. Argentina. Mendoza: Estancia Santa Rosa, 1904, *A.C. Jensen-Haarup s.n.* (holotype: UPS; isotype: US [00027594, acc. # 1081085]).
Solanum meizonanthum Bitter, Repert. Spec. Nov. Regni Veg. 11: 214. 1912. Type. Argentina. Entre Ríos: Paraná, 16 Aug 1892, *G. Niederlein 270* (holotype: B, destroyed [F neg. 2783]; lectotype, designated by Knapp et al. 2020, pg. 40: F [V0361924F, acc. # 621142]).
Solanum atriplicoides Herter, Rev. Sudamer. Bot. 7: 226. 1943, nom. illeg. superfl. Type. Based on Solanumatriplicifolium Gillies ex Nees.

##### Type.

Cultivated [Glasgow Botanical Garden, protologue] from seeds sent by J. Tweedie from “near Buenos Ayres”, *Anon. s.n.* (lectotype, designated by Edmonds 1972, pg. 102 [as “holotype”], second step designated by Knapp et al. 2020, pg. 40: K [K000585739]; isolectotype: K [K000585738]).

**Figure 178.**
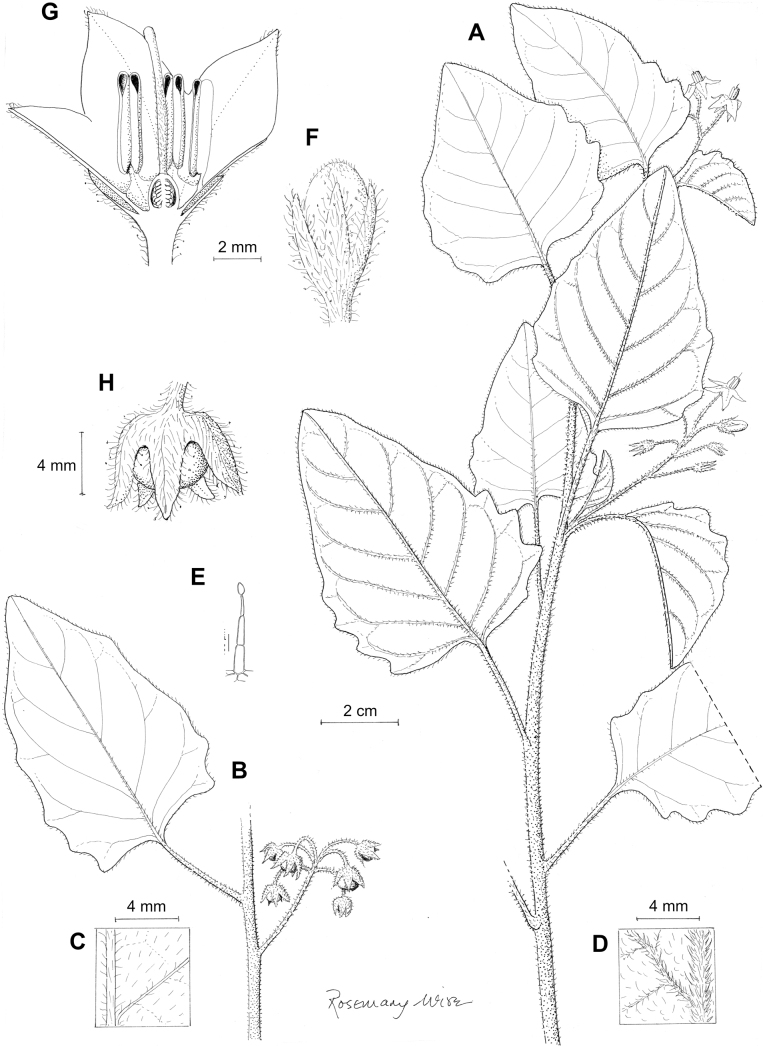
*Solanumtweedieanum***A** flowering habit **B** fruiting habit **C** detail of adaxial leaf surface **D** detail of abaxial leaf surface **E** glandular trichome of the stem **F** flower bud **G** dissected flower **H** maturing fruit (**A–D, F, G***Wood & Goyder 16818***E** voucher details missing). Illustration by R. Wise and V. Dudas. Previously published in part in Cabrera (1983: 404) and Barboza et al. (2013: 239) as *S.atriplicifolium*.

##### Description.

Perennial herbs or subshrubs woody at the base, rhizomatous, 0.1–0.75 m high, viscid to the touch, the branches erect to spreading. Stems terete, densely pubescent with glandular transparent simple uniseriate trichomes mostly 0.5 mm long and 1–2-celled, but some scattered trichomes 6–10-celled, 1–1.5 mm long, the glandular tips unicellular; new growth viscid-pubescent with glandular simple uniseriate trichomes like those of the stems; bark of older stems pale tan, the longer trichomes deciduous, but stems remaining viscid with shorter glandular trichomes. Sympodial units difoliate, the leaves not geminate, but occasionally arising very near each other. Leaves simple and usually shallowly toothed, the blades (1.5)4–6 cm long, (0.8)2–5 cm wide, ovate to elliptic, widest at or just below the middle, membranous, concolorous, viscid to touch, extremely variable in size both between and within plants; adaxial surfaces evenly and more or less densely pubescent on the veins and lamina with transparent glandular simple uniseriate trichomes 0.2–0.5(–1) mm long; abaxial surfaces similarly viscid-pubescent, the glandular trichomes denser along the veins; principal veins (4)4–7 pairs, sometimes drying yellowish; base truncate, then slightly decurrent along the petiole as a wing less than 0.5 mm wide; margins usually shallowly toothed, occasionally almost entire, the teeth to 2.5 mm long, broadly deltate with acute to slightly rounded apices, the sinuses reaching less than 1/4 of the way to the midrib; apex acute; petiole 0.5–2.5 cm long, with a narrow wing of leaf tissue along most of its length. Inflorescences opposite the leaves, usually unbranched (forked in *Wood et al. 18764* from Bolivia), 1.5–6 cm long, with 4–8 flowers clustered in the distal portion, densely glandular pubescent with transparent, simple uniseriate trichomes to 1 mm long; peduncle 1–4 cm long; pedicels 0.7–1 cm long, ca. 0.5 mm in diameter at the base, gradually tapering to an apex ca. 1 mm in diameter, nodding or somewhat spreading at anthesis, densely glandular pubescent like the rest of the inflorescence, articulated at the base; pedicel scars clustered at the tips of the inflorescence 1–2 mm apart. Buds ellipsoid, the calyx ca. halfway exserted from the tips of the calyx lobes before anthesis. Flowers 5-merous, cosexual (hermaphroditic). Calyx tube (0.5)1–1.5 mm long, cup-shaped to somewhat urceolate, the lobes 3.5–5 mm long, 2–3 mm wide, narrowly triangular with pointed tips, densely glandular pubescent with transparent simple uniseriate trichomes. Corolla 1.2–1.6 cm in diameter, white or lavender with a pale greenish yellow central eye, stellate, lobed ca. 2/3 of the way to the base, the lobes 4–5 mm long, 3–5 mm wide, triangular, spreading or reflexed at anthesis, glabrous adaxially, densely glandular-papillate abaxially especially along the midvein and at lobe tips, with a few longer glandular simple uniseriate trichomes at the lobe tips. Stamens equal, or sometimes the lowermost apparently very slightly longer; filament tube minute; free portion of the filaments 0.5–1 mm long, slightly unequal, glabrous or sparsely pubescent adaxially with eglandular tangled simple uniseriate trichomes; anthers (3.6)4–5(6) cm long, 1–1.2 mm wide, ellipsoid, yellow, poricidal at the tips, the pores lengthening to slits with age. Ovary conical, glabrous; style ca. 9 mm long, slightly curved upwards, exserted beyond the anther cone, densely pubescent in the lower third with 2–3-celled simple uniseriate trichomes to 0.5 mm long; stigma large-capitate to somewhat bilobed, yellow-green in live plants, the surface minutely papillate. Fruit a globose berry, 0.4–0.8 cm in diameter, greenish white to cream at maturity, tightly enclosed in the accrescent calyx, the pericarp thin, matte, opaque, glabrous; fruiting pedicels 0.8–1.1 cm long, ca. 0.75 mm in diameter at the base, ca. 1.1 mm in diameter at the apex, nodding, strongly deflexed at the base with a distinct bend, not markedly woody, not persistent; fruiting calyx accrescent and tightly investing the berry, the tube 3–3.5 mm long, the lobes 2.5–8 mm long, the lobes expanding more than the tube, remaining viscid-pubescent. Seeds 10–20 per berry, ca. 2 mm long, ca. 1.5 mm wide, flattened and teardrop shaped, pale tan to reddish brown, the surfaces minutely pitted, the testal cells sinuate in outline. Stone cells 8–10 per berry, 2 apical (*fide*Bitter 1914a), to 1.2 mm in diameter, the rest scattered through the mesocarp, 0.7–1 mm in diameter, all creamy white. Chromosome number: n = 12 (Moscone 1992, vouchers *Del Vitto & Moscone 854*, *Di Fulvio 783*, *Hunziker 24883, 25036*, all as *S.atriplicifolium*).

**Figure 179.**
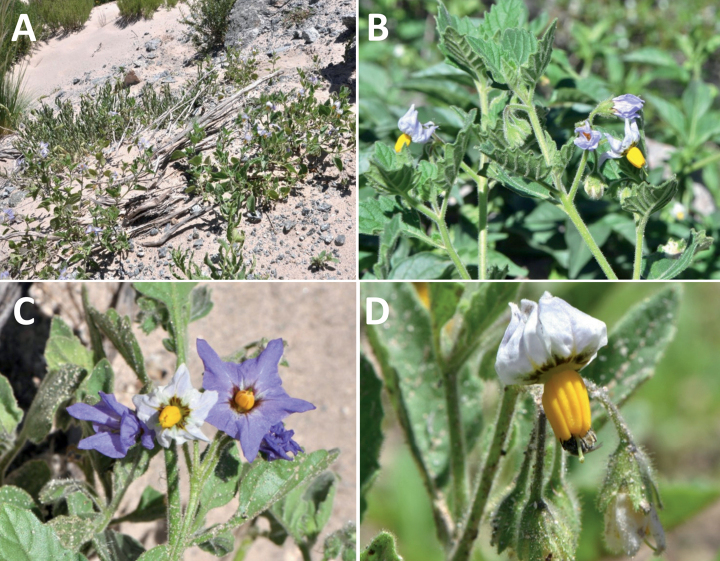
*Solanumtweedieanum***A** habit **B** flowering branch **C** inflorescence with corolla colour variation **D** flower at anthesis (**A***Barboza et al. 3483***B***Barboza et al. 3472***C***Barboza et al. 3486***D***Barboza et al. 3474*). Photos by S. Knapp.

##### Distribution

**Figure 180.**
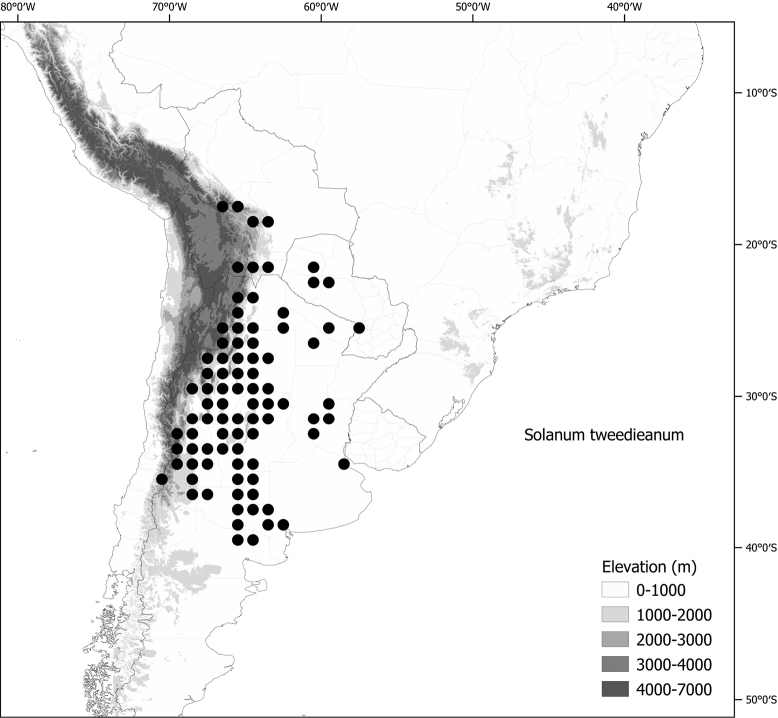
Distribution map of *Solanumtweedieanum*.

**(Fig. 180).***Solanumtweedieanum* occurs on the eastern Andean slopes and foothills and into the littoral (Argentina) in Bolivia (Depts. Santa Cruz, Tarija), Paraguay (Depts. Boquerón, Central), and across Argentina (Provs. Buenos Aires, Catamarca, Córdoba, Entre Ríos, Formosa, Jujuy, La Pampa, La Rioja, Mendoza, Río Negro, Salta, San Juan, San Luis, Santiago del Estero, Tucumán).

##### Ecology and habitat.

*Solanumtweedieanum* grows in a very wide range of habitats from the littoral of Argentina, Chaco woodlands and high elevation open areas above tree line in the Andes, from near sea level to 3,500 m elevation. It often is found in the shade of trees in loose soil or in the cracks of rocks, often in large patches connected with underground rhizomes.

##### Common names and uses.

None recorded.

##### Preliminary conservation status

**(IUCN 2022).** Least Concern [LC]. EOO = 2,010,678 km^2^ [LC]; AOO = 1,420 km^2^ [VU]. *Solanumtweedieanum* is a widespread species that reproduces clonally by rhizomes and occupies a wide range of habitats. It is found within protected areas in a variety of habitats in Argentina (e.g., Pampa de Achala, Ernesto Tornquist Provincial Park) and probably also occurs in the Paraguayan Parque Nacional Defensores del Chaco.

##### Discussion.

*Solanumtweedieanum* is one of the mostly widely distributed of the glandular-pubescent morelloid species with accrescent calyces in fruit. The name *S.atriplicifolium* was formerly applied to this species (e.g., Barboza et al. 2013) but re-evaluation of taxon circumscription and types revealed that the names for glandular-haired species with accrescent calyces were previously incorrectly applied (Knapp et al. 2020). Previously Barboza et al. (2013) recognised *S. “tweedianum*” Hook. (a mis-spelling of *S.tweedieanum*, see below) and *S.atriplicifolium* as distinct species and placed *S.physalidicalyx* in synonymy with *S.tweedieanum*. The type of *S.tweedieanum* does not match these specimens but is a better match for the plants called *S.atriplicifolium* in Barboza et al. (2013). The type of *S.tweedieanum* comes from a plant cultivated at Kew that was collected in flower only; it lacks the diagnostic calyx characters (see fig. 1 in Knapp et al. 2020) that enable easy identification in this group, but anther length can also be used to distinguish those plants not in fruit. Plants with inflated calyces have shorter anthers than do those with calyces that are merely accrescent and tightly investing the berry; the types of both *S.tweedieanum* and *S.atriplicifolium* have longer (to 6 mm) anthers and belong to the same species, for which the oldest name is *S.tweedieanum*.

*Solanumtweedieanum* is most similar to *S.physalidicalyx*, from which it differs in having longer anthers (4–6 mm long versus 3–4 mm long in *S.physalidicalyx*) and a fruiting calyx that is accrescent but tightly invests the pale cream berry rather than the inflated accrescent calyx of *S.physalidicalyx* that is somewhat invaginate at the base. In mature fruit, the calyx lobes are longer than the tube in *S.tweedieanum*, whereas in *S.physalidicalyx* the inflated tube is longer than the lobes, but this can be difficult to see in herbarium specimens, and in the absence of mature fruit, determination can be difficult.

The chromosome count of 2n = 24 reported by Edmonds (1972) for *S.tweedieanum* (as *tweedianum*) is based on a voucher (*Hawkes et al. 3204*) we have been unable to locate. From the locality (between Mina Clavero and Villa Dolores in Córdoba, Argentina), this could represent either *S.tweedieanum* or *S.physalidicalyx*.

Details of the orthography of the name and typification of *S.tweedieanum* and its synonyms are treated in Knapp et al. (2020) as is the confusion over the application of this name.

#### ﻿60. Solanum weddellii

Taxon classificationPlantaeSolanalesSolanaceae

Phil., Anales Mus. Nac. Chile, Segunda Secc., Bot. 1891: 65. 1891.

Figs 2A , 181 , 182

Chamaesaracha boliviensis Dammer, Bot. Jahrb. Syst. 49: 215. 1913. Type. Bolivia. La Paz: Between Palca and La Paz, *K. Pflanz 145* (holotype: B, destroyed [F neg. 2710]). Bolivia. La Paz: Prov. Ingavi, cantón Jesus de Machaca, comunidad Titicani-Tacaca, a 20 km de Guaqui, 3820 m, 22 Mar 1989, *X. Villavencio L. 318* (neotype, designated here: LPB; isoneotype: CORD [CORD00101735]).
Solanum chamaesarachidium Bitter, Repert. Spec. Nov. Regni Veg. 15: 94. 1917. Type. Based on Chamaesarachaboliviensis Dammer.

##### Type.

Chile. Región I (Tarapacá): Prov. Tarapacá, Calcalhuay, Jan 1886, *C. Rahmer s.n.* (no herbaria cited; lectotype, designated here: SGO [SGO000004605]; isolectotype: WU [acc. # 1903-0010229]).

**Figure 181.**
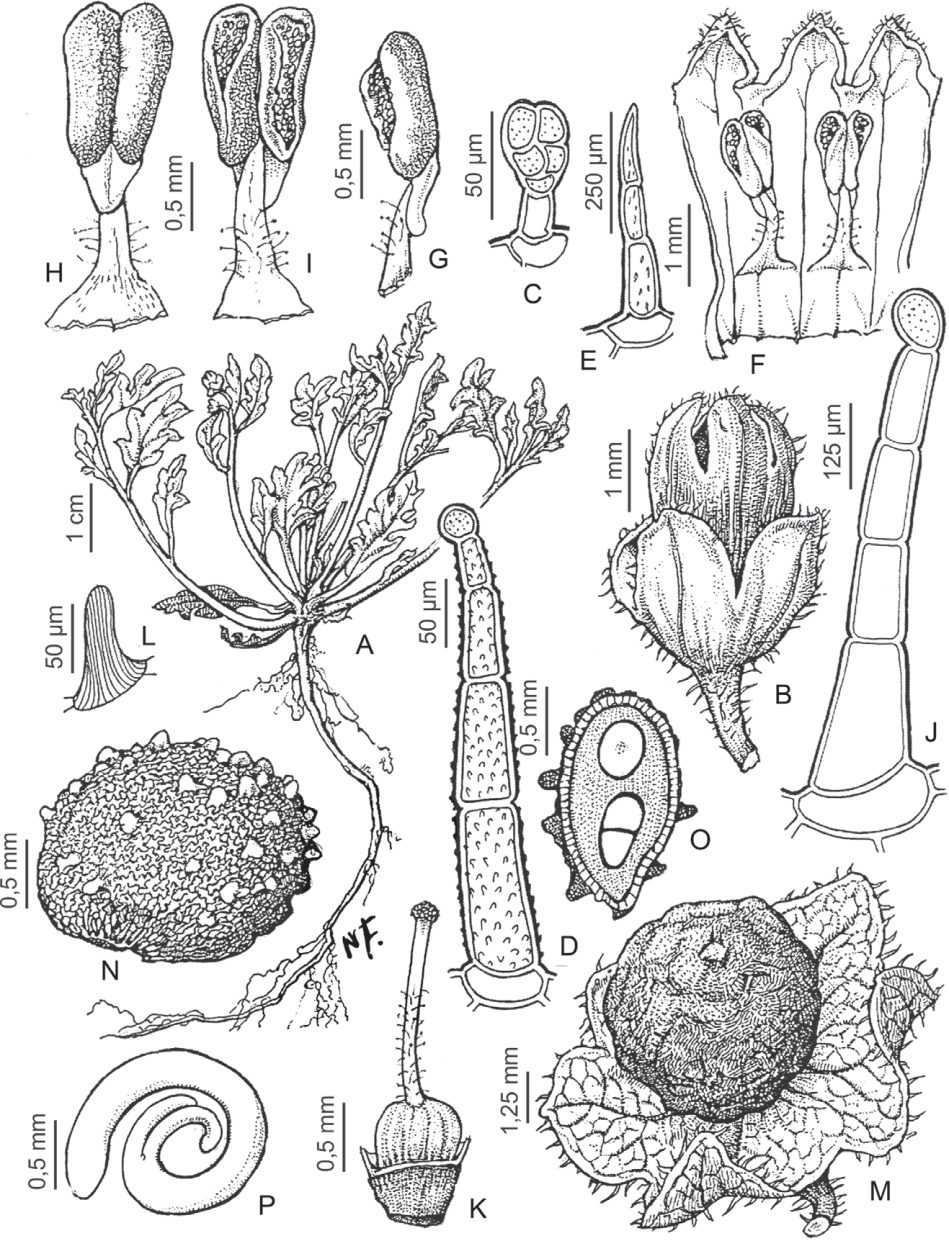
*Solanumweddellii***A** habit **B** flower bud **C, D** glandular trichomes of the calyx **E** eglandular trichome of the calyx **F** section of a dissected flower **G** stamen, lateral view **H** stamen, dorsal view **I** stamen, ventral view **J** glandular trichome of the filament **K** gynoecium **L** papilla of the style **M** fruit **N** seed **O** seed cross section **P** embryo (**A–P***Krapovickas 6219*). Illustration by N. de Flury. Previously published in Barboza et al. (2013: 241) as *S.chamaesarachidium*.

##### Description.

Tiny annual herbs to 0.2 m high, usually appearing as a prostrate rosette. Stems terete, sparsely pubescent with eglandular, 2–4-celled simple uniseriate trichomes to 0.5 mm long, these antrorse and slightly verrucose, and shorter 1–2-celled glandular trichomes to 0.2 mm long; new growth sparsely to densely pubescent with tangled, weak-walled eglandular simple uniseriate trichomes; older stems green. Sympodial units plurifoliate, the leaves not geminate. Leaves simple, shallowly to deeply lobed, the blades 1–3(4) cm long, 0.5–1.5(2) cm wide, elliptic to narrowly elliptic in outline, widest at the middle, thick and somewhat fleshy in live plants, concolorous; adaxial and abaxial surfaces sparsely pubescent with tangled, eglandular simple uniseriate trichomes to 0.5 mm long, these denser on the veins; principal veins 3–4 pairs, not clearly visible (except as lobes) in live plants; base attenuate onto the winged petiole; margins shallowly to deeply lobed, revolute and undulate, the sinuses reaching 1/4–3/4 of the distance the midrib, the lobes triangular with deltate, rounded tips; petiole 0.4–1.5 cm long, winged from the attenuate leaf base. Inflorescences internodal, sometimes very near the nodes and then appearing almost opposite the leaves, unbranched, 0.3–1 cm long, with 3–5 flowers clustered at the tip, a single flower open at a time, sparsely to moderately pubescent with tiny glandular papillae and longer eglandular simple uniseriate trichomes to 0.5 mm long like those of the stems; peduncle 0.25–0.9 cm long; pedicels 0.2–0.4 cm long, ca. 0.25 mm in diameter at the base, ca. 0.25 mm in diameter at the apex, erect at anthesis, sparsely to moderately pubescent with a mixture of glandular papillae and tangled simple uniseriate trichomes like the rest of the inflorescence, articulated at the base; pedicel scars clustered or to ca. 1 mm apart, occasionally to as much as 4 mm apart in larger inflorescences. Buds ellipsoid, the corolla ca. halfway exserted from the calyx before anthesis (only just surpassing the tips of the calyx lobes). Flowers 5-merous, cosexual (hermaphroditic). Calyx tube 1–1.5 mm long, conical, the lobes 1.1.5 mm long, 1–1.5 mm wide, deltate with rounded tips, sparsely pubescent with a mixture of glandular papillae and 2–4-celled eglandular simple uniseriate trichomes. Corolla ca. 0.6 cm in diameter, purple or white (fading with flower age through anthesis) with a large greenish yellow, purple-edged central eye, rotate, lobed less than 1/4 of the way to the base, the lobes (acumens) ca. 1 mm long, ca. 1 mm wide, spreading at anthesis, adaxially glabrous except for the papillate lobe tips, abaxially with scattered eglandular simple uniseriate trichomes over the entire surface. Stamens equal; filament tube minute; free portion of the filaments 0.5–0.7 mm long, densely pubescent with tangled simple uniseriate trichomes adaxially; anthers ca. 1 mm long, ca. 1 mm wide, globose to broadly ellipsoid, yellow, poricidal at the tips, the pores lengthening to slits with age. Ovary conical, glabrous; style ca. 1.5 mm long, straight, only just exceeding the anther cone, densely papillate in the lower 3/4 of its length; stigma large-capitate and globose, ca. 1 mm in diameter, the surface minutely papillate, bright green in live plants. Fruit a globose berry, 0.5–0.7 cm in diameter, pale green to pale whitish green at maturity, the pericarp thin, matte, opaque, glabrous; fruiting pedicels 0.6–0.7 cm long, ca. 0.5 mm in diameter at the base, ca. 1 mm in diameter at the apex, not markedly woody, deflexed with the berry pointing down to rest on the soil, not persistent; fruiting calyx accrescent and inflated, the calyx tube ca. 0.5 cm long strongly angled between the lobes, the lobes ca. 0.5 cm long, 0.5–0.6 cm wide, more or less halfmoonshaped to deltate, the margins somewhat overlapping, the venation prominent and slightly purplish black or dark green in live plants, darker in dry specimens, the berry always visible inside the inflated calyx. Seeds 2–7(17) per berry, 2–3 mm long, 1–2 mm wide, irregularly shaped to somewhat teardrop shaped, not markedly flattened, black to dark brown, the surface tuberculate, the testal cells pentagonal in outline. Stone cells absent. Chromosome number: 2n = 24 (Chiarini et al. 2017, voucher *Särkinen et al. 4038*).

**Figure 182.**
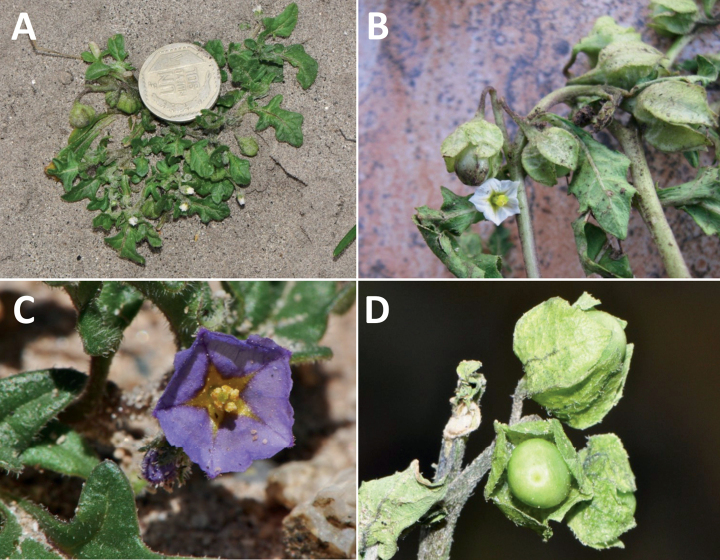
*Solanumweddellii***A** habit **B** flowering and fruiting branch **C** flower at full anthesis **D** maturing fruits (**A, D***Särkinen et al. 4038***B***Cano et al. 20615***C***Barboza et al. 3475*). Photos by A. Cano, S. Knapp and T. Särkinen.

##### Distribution

**Figure 183.**
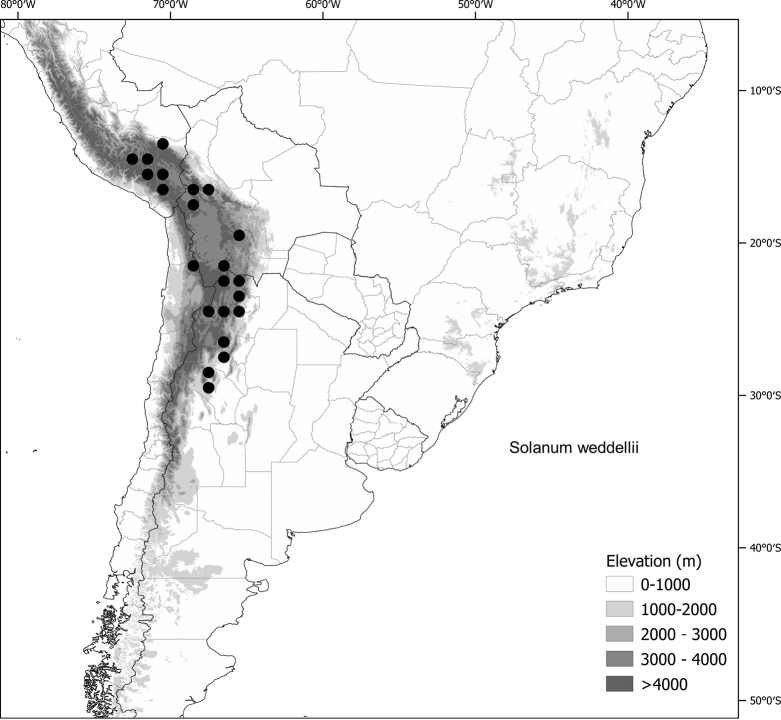
Distribution map of *Solanumweddellii*.

**(Fig. 183).***Solanumweddellii* occurs in the high Andes of Peru (Depts. Arequipa, Cusco, Huancavelica, Moquegua, Puno), Bolivia (Depts. La Paz, Potosí), Argentina (Provs. Catamarca, La Rioja, Jujuy, Salta, Tucumán) and Chile (Regions I [Tarapacá], II [Antofagasta]).

##### Ecology and habitat.

*Solanumweddellii* is a plant of open sandy areas above treeline in the puna or high elevation semi-desert (xerophytic) habitats, growing in loose sandy soil among gravel and with other small herbs, from 2,300 to 4,550 m elevation.

##### Common names and uses.

None recorded.

##### Preliminary conservation status

(IUCN 2022) Vulnerable [VU, B 2a,b(iv), D2]. EOO = 603,950 km^2^ [LC]; AOO = 220 km^2^ [EN]. *Solanumweddellii* has a large extent of occurrence but grows only in very loose sandy soils in widely dispersed populations. It is known from fewer than ten sites. Given these parameters we would suggest it is of some conservation concern. It is known to occur within protected areas in Argentina (e.g., Humahuaca World Heritage site) but is often collected in areas around mining operations; whether this is an access issue or more related to the soil type specificity of *S.weddellii* is not known.

##### Discussion.

*Solanumweddellii* is morphologically very similar to *S.gilioides* and it can sometimes be difficult to distinguish them from fragmentary herbarium specimens. The most striking differences are in calyx shape through anthesis and fruit development. In flower, the calyx lobes of *S.gilioides* are linear or narrowly triangular and grow considerably in length when fruiting (Fig. 62G, H), while those of *S.weddellii* are more ovate-deltate and expand in width in fruit, forming a broadly open, somewhat frilly-looking cup (Fig. 182D). Leaves of *S.weddellii* are more densely pubescent and usually less deeply divided than those of *S.gilioides* and flowers are smaller (ca. 0.6 cm in diameter with anthers ca. 1 mm long in *S.weddellii* versus ca. 1.6 cm in diameter with anthers 1–3 mm long in *S.gilioides*). These two species were previously recognised as section Chamaesarachium Bitter (Barboza 2003), along with *S.annuum*. Molecular data confirm the close relationship of *S.weddellii* and *S.gilioides* as the monophyletic Chamaesarachidium clade (Särkinen et al. 2015b), but *S.annuum* is not related to them (Gagnon et al. 2022).

Plants of *S.weddellii* vary enormously in size even in similar habitats; this may be due to local microenvironmental differences. The patchy distribution of *S.weddellii* may be due to its preference for loose sandy soils; we have only collected it in high elevation areas with sand soil or dune-like habitats; *S.gilioides* in contrast is found in a wide variety of rocky and sandy areas.

No herbaria were cited in the original description of *S.weddellii*; we have selected a specimen in SGO (SGO000004605) corresponding to the collector, locality and date from the protologue as the lectotype.

We have found no duplicates of the Pflanz collection used to describe *Chamaesarachaboliviensis* and it is likely to have been a sheet in Berlin that was destroyed (B, F neg. 2710), but no herbaria were cited in the protologue (Dammer 1913). Barboza et al. (2013) did not neotypify this name, but we here select a fruiting collection gathered in March from the vicinity of La Paz, Bolivia (*Villavicencio L. 318*, LPB) as the neotype for this name.

#### ﻿61. Solanum woodii

Taxon classificationPlantaeSolanalesSolanaceae

Särkinen & S.Knapp, PhytoKeys 74: 26. 2016.

Figs 184 , 185

##### Type.

Bolivia. Santa Cruz: Prov. Valle Grande, pasando el puente Santa Rosa, a 78 km desde Serrano hacia Valle Grande, 1,169 m, 4 Apr 2003, *J.R.I. Wood 19616* (holotype: LPB).

**Figure 184.**
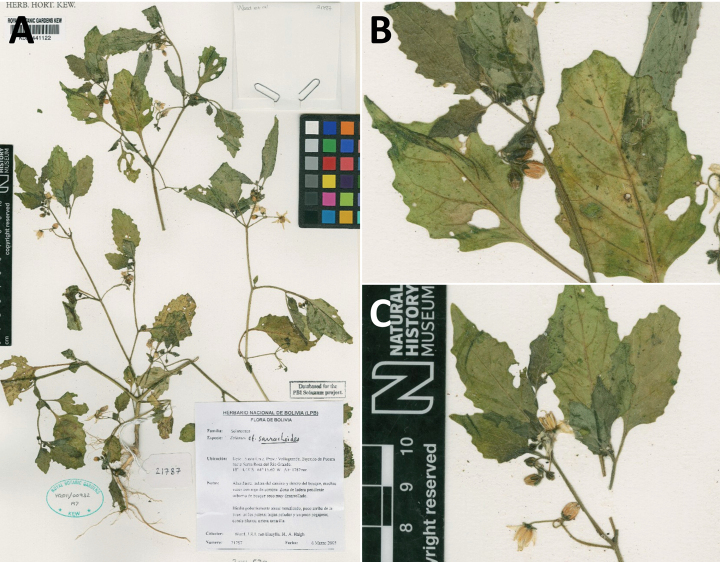
*Solanumwoodii***A** habit **B** inflorescence in bud **C** inflorescence with flowers at anthesis (**A–C***Wood 21787* [K000441122]). Reproduced with permission of the Trustees of the Royal Botanic Gardens, Kew.

##### Description.

Decumbent, slender annual (*fide* labels) herbs to 0.3–0.4 m high, much branching. Stems terete, pale yellow or greenish beige, glabrescent; new growth densely pubescent with spreading translucent 5–8-celled simple uniseriate glandular trichomes ca. 0.5 mm long, some to 1 mm. Sympodial units difoliate, not geminate. Leaves simple and often shallowly toothed, the blades (2.3–)4.5–8 cm long, (1.5–)2.2–4.3 cm wide, elliptic to ovate, widest at the middle or in the lower third, thin-membranous, slightly discolorous; adaxial surface moderately pubescent with spreading hairs as on stem evenly spaced along lamina and veins; abaxial surface more densely pubescent along veins; major veins 5–7 pairs; base attenuate to decurrent; margins entire to shallowly and unevenly toothed, the lobes narrow; apex acute; petiole 0.8–4.5 cm long, sparsely pubescent with simple 5–8-celled uniseriate trichomes like those of the stems. Inflorescences unbranched, opposite the leaves, 1.5–3 cm long, with (2–)3–7 flowers, sparsely pubescent with simple 5–8-celled uniseriate trichomes like those of the stems; peduncle 0.9–1.8 cm long, ca. 0.3 mm in diameter at the apex and ca. 0.5 mm in diameter at the base; pedicels spaced 0–1 mm apart, 0.7–1.1 cm long, ca. 0.2 mm in diameter at the base and ca. 0.3 mm in diameter at the apex, straight and spreading at anthesis, articulated at the base. Buds ovoid, white, the corolla strongly exserted from the calyx before anthesis, exceeding the lobes by up to two times their length. Flowers 5-merous, cosexual (hermaphroditic). Calyx tube 0.6–0.7 mm long, the lobes 1.2–2.1 mm long, 0.8–1 mm wide, ovate to elliptic in outline with acute apices, somewhat spreading at anthesis, sparsely pubescent with simple 5–8-celled uniseriate glandular trichomes like those of the stems. Corolla 1–1.5 cm in diameter, white with a greenish-purple central star at the base, stellate, lobed to the middle, the lobes 4–6 mm long, 2–3 mm wide, reflexed at anthesis, sparsely pubescent abaxially with very short 1–2-celled simple uniseriate eglandular trichomes. Stamens equal; filament tube ca. 0.5 mm long; free portion of the filaments 0.1–0.4 mm long, adaxially pubescent with 4–7-celled uniseriate eglandular trichomes; anthers (2.5–)3–3.8 mm long, 1.2–1.4 mm wide at base, ca. 0.5 mm at tip, tapering and narrowly triangular to triangular in outline, yellow, poricidal at the tips, the pores lengthening to slits with age. Ovary globose, glabrous; style 4.5–5 mm long, curved at the very tip, exserted beyond the anther cone, densely pubescent with 2–3-celled simple uniseriate trichomes in the basal 1/3 where included in the anther cone; stigma minutely capitate, the surface papillate. Fruit a globose berry, 0.5–0.9 cm in diameter, green (immature), the pericarp thick and shiny, opaque, glabrous; fruiting pedicels 0.7–1 cm long, ca. 0.5 mm in diameter at the base, ca. 0.6 mm in diameter at the apex, spaced 0–1 mm apart, spreading to recurved, not persistent; fruiting calyx tube ca. 1 mm long, the lobes 2–3.5 mm long, spreading to reflexed. Seeds 15–30 per berry, 1.6–2 mm long, 1–1.5 mm wide, flattened, teardrop-shaped with a subapical hilum, yellow, the surface minutely pitted, the testal cells pentagonal in outline with the lateral cell walls elongate and the seeds from mature fruits appearing hairy. Stone cells absent. Chromosome number: not known.

**Figure 185.**
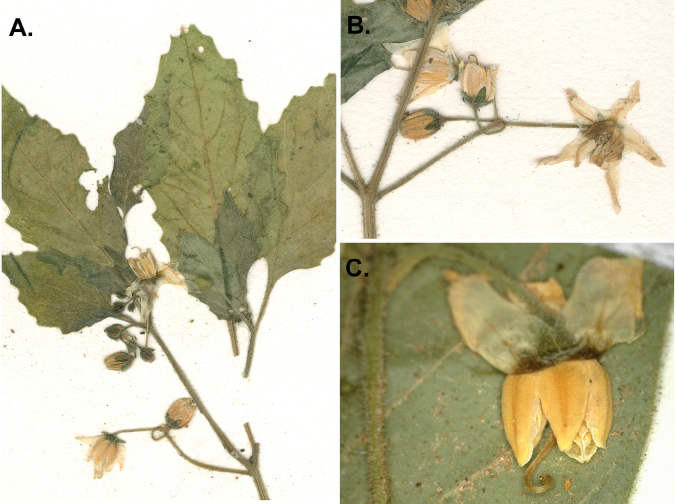
*Solanumwoodii***A** flowering stem **B** inflorescence with details of buds, calyx and corolla **C** flower at anthesis (**A, B***Wood 21787* [K000441122] **C***Nee et al. 51967* [BM001211468]). Photos by G. Davis. Previously published in Särkinen and Knapp (2016: 27). Reproduced with permission of the Trustees of the Natural History Museum and the Royal Botanic Gardens, Kew.

##### Distribution

**Figure 186.**
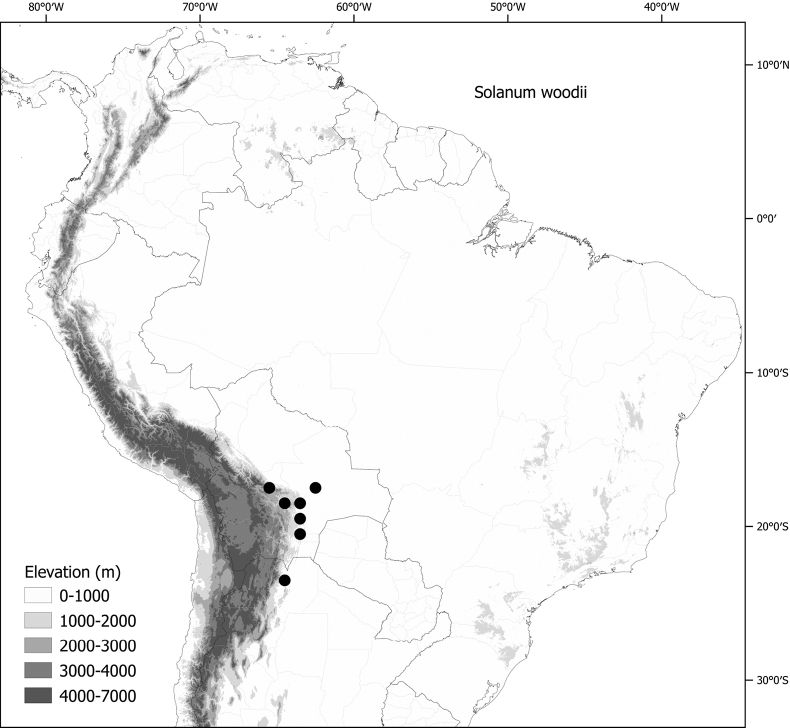
Distribution map of *Solanumwoodii*.

**(Fig. 186).***Solanumwoodii* occurs in Bolivia (Depts. Chuquisaca, Santa Cruz) and in northern Argentina (Prov. Jujuy). When originally described (Särkinen and Knapp 2016), it was thought to be a Bolivian endemic.

##### Ecology and habitat.

*Solanumwoodii* grows in Chaco and Chaco forests of inter-Andean valleys in Bolivia and northern Argentina, in dry Chaco woodlands on sandy and clay soils near water sources, rivers and in moist depressions in partial or full shade; between 300 and 1,800 m elevation.

##### Common names and uses.

None recorded.

##### Preliminary conservation status

**(IUCN 2022).** Least Concern [LC]. EOO = 122,138 km^2^ [LC]; AOO = 64 km^2^ [EN]. The preliminary threat status of *S.woodii* was assessed as Vulnerable (VU, B1) by Särkinen and Knapp (2016); recent collections from Argentina have revealed it to be more widely distributed than previously thought but is rare where it occurs. No occurrences are known within protected areas thus far.

##### Discussion.

*Solanumwoodii* is unusual in South American morelloids having tapering, somewhat cone-shaped anthers with a beak-like tip (see Fig. 185C); this character, however, can be difficult to see in older flowers with dehisced anthers. Among other glandular-viscid herbaceous solanums, it could be confused with *S.tweedieanum* and *S.physalifolium*. *Solanumwoodii* is sympatric with *S.tweedieanum* but the latter species has longer calyx lobes in flower (3.5–5(–7) mm) and fruit (>5 mm) and slightly larger ellipsoid anthers (3–)4–4.5 mm long that are rectangular in outline (equally wide along their entire length) rather than broadest at the base; the calyx of *S.tweedieanum* is accrescent and completely covers the berry at maturity, while that of *S.woodii* is spreading and does not become accrescent.

The unusual anther shape in *S.woodii* resembles that of *S.anomalostemon* from the dry inter-Andean valley of the Rio Apurimac in southern Peru (Knapp and Nee 2009). Despite the similarity in anther shape, preliminary molecular data suggest *S.woodii* is a member of the morelloid lineage, whereas *S.anomalostemon* is morphologically unique within *Solanum* in having cordate anthers and has been resolved as an independent lineage not closely related to the Morelloid clade (Gagnon et al. 2022).

#### ﻿62. Solanum zuloagae

Taxon classificationPlantaeSolanalesSolanaceae

Cabrera, Hickenia 1(41): 225. 1980.

Figs 187 , 188

##### Type.

Argentina. Jujuy: Dpto. Palpalá, Mina 9 de Octubre, Sierra de Zapla, subida a la antenna, 24 Jan 1975, *F.O. Zuloaga & N.B. Deginani 225* (holotype: SI [003663, acc. # 074662]).

**Figure 187.**
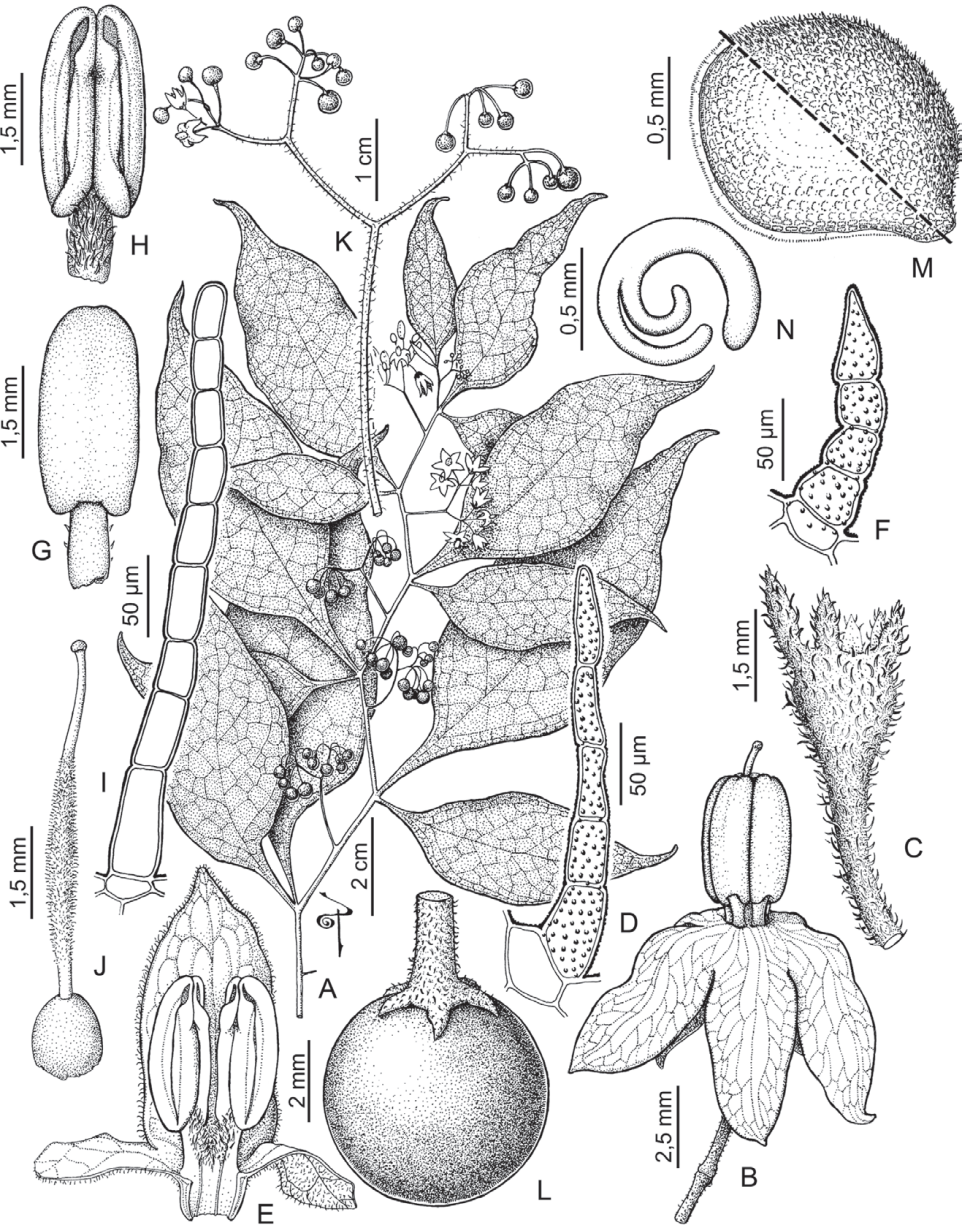
*Solanumzuloagae***A** flowering and fruiting branch **B** flower **C** calyx **D** eglandular trichome of the calyx **E** sector of the dissected flower **F** eglandular trichome of the corolla **G** stamen, dorsal view **H** stamen, ventral view **I** eglandular trichome of the filament **J** gynoecium **K** infructescence **L** fruit **M** seed **N** embryo (**A–N***Barboza et al. 2208*). Illustration by P. Peralta. Previously published in Barboza et al. (2013: 263)

##### Description.

Erect or spreading perennial herbs or subwoody shrubs, to 2 m high or spreading to 2 m diameter. Stems terete, sparsely pubescent with spreading eglandular 2–8-celled simple uniseriate trichomes to 1.5 mm long, these drying white; new growth densely to moderately pubescent with simple uniseriate eglandular trichomes like those of the stems, on the leaves these mostly along the veins; bark of older stems green to pale brown. Sympodial units difoliate, the leaves more or less geminate, those of a pair equal in size and shape. Leaves simple, entire or rarely shallowly toothed, the blades 3.5–16 cm long, 2.5–7.5 cm wide, elliptic to narrowly elliptic, widest at the middle, membranous, concolorous; adaxial surfaces very sparsely and evenly pubescent with 4–6-celled eglandular simple uniseriate trichomes to 1.5 mm long, appearing glabrous to the naked eye; abaxial surfaces similarly sparsely pubescent, but with slightly denser pubescence along the veins; principal veins 5–7 pairs, drying yellowish green or brown, more pubescent than the lamina; base attenuate and somewhat decurrent onto the petiole; margins entire or shallowly toothed, if toothed the teeth to 2 mm long, all margins ciliate-pubescent with antrorse 2–6-celled eglandular simple uniseriate trichomes to 1 mm long, these drying white; petiole 0.5–1 cm long, sparsely pubescent like the stems and leaves. Inflorescences internodal, forked to several times branched, 4–8 cm long, with 20–40 flowers, moderately to densely pubescent with weak, spreading eglandular simple uniseriate trichomes to 1 mm long; peduncle 1.5–4 cm long; pedicels 0.8–1.1 cm long, ca. 0.5 mm in diameter at the base, ca. 1 mm in diameter at the apex, tapering, spreading at anthesis, sparsely to moderately pubescent like the stems and inflorescence axis, articulated at the base, leaving small stumps along the axis; pedicel scars irregularly spaced 1.5–5 mm apart, slightly raised from the axis. Buds globose to broadly ellipsoid, the corolla strongly exserted from the calyx before anthesis. Flowers 5-merous, cosexual (hermaphroditic). Calyx tube ca. 0.5 mm long, conical, the lobes 1.1–5 mm long, 0.5–1 mm wide, sometimes unequal in length in the same flower, narrowly triangular, sparsely to moderately pubescent like the rest of the inflorescence, the trichomes eglandular, simple, uniseriate and drying white. Corolla 1.2–1.8 cm in diameter, white, stellate, lobed ca. 3/4 of the way to the base, the lobes 3–7 mm long, 3–5 mm wide, deltate, spreading to reflexed at anthesis, adaxially glabrous, abaxially densely papillate with 1–3-celled trichomes over entire surface. Stamens equal; filament tube ca. 0.5 mm long; free portion of the filaments ca. 0.5 mm long, pubescent with transparent, tangled eglandular simple uniseriate trichomes adaxially; anthers 3–3.5 mm long, 1.25–1.5 mm wide, broadly ellipsoid, yellow, poricidal at the tips, the pores lengthening to slits with age. Ovary conical, glabrous; style 5–6 mm long, straight, exserted beyond the anther cone, densely pubescent in the lower half where included in the anther cone; stigma small-capitate, the surface minutely papillose. Fruit a globose berry, 0.4–0.5 cm in diameter, green (?) when mature, the pericarp thin, matte, opaque, glabrous; fruiting pedicels 0.8–1.1 cm long, ca. 0.5 mm in diameter at the base, ca. 0.5 mm in diameter at the apex, not markedly woody, spreading, not persistent; fruiting calyx not accrescent, the lobes appressed to the berry to slightly reflexed. Seeds ca. 30 per berry, ca. 1.2 mm long, ca. 1.2 mm wide, not markedly flattened, round or somewhat teardrop shaped, pale yellowish tan, the surfaces minutely pitted, the testal cells sinuate in outline near the centre of the seed, more rectangular at the margins. Stone cells 8 per berry, 4 larger ca. 1 mm in diameter, 4 smaller ca. 0.5 mm in diameter, cream-coloured, the surfaces occasionally ornamented (*Barboza 2202*). Chromosome number: n = 12 (Moyetta et al. 2013, voucher *Barboza et al. 3569*).

**Figure 188.**
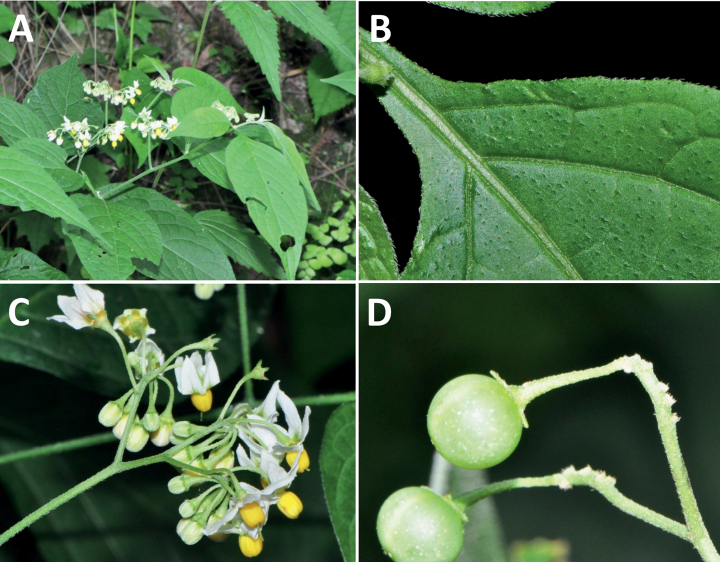
*Solanumzuloagae***A** habit **B** ciliate leaf margins **C** inflorescence with buds and flowers at full anthesis **D** maturing fruits (**A–D***Barboza et al. 3569*). Photos by S. Knapp.

##### Distribution

**Figure 189.**
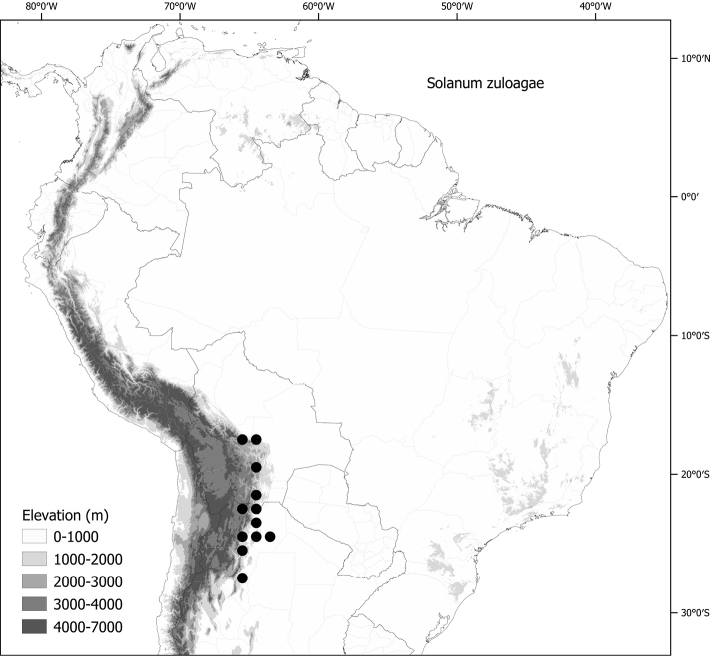
Distribution map of *Solanumzuloagae*.

**(Fig. 189).***Solanumzuloagae* occurs in the Andes of northern Argentina (Provs. Jujuy, Salta, Tucumán) and Bolivia (Depts. Cochabamba, Chuquisaca, Santa Cruz).

##### Ecology and habitat.

*Solanumzuloagae* grows in understorey of moist forests or ‘yungas’, often at clearing edges or scrambling over other vegetation in treefalls or roadside, from 640 to 2,940 m elevation.

##### Common names and uses.

None recorded.

##### Preliminary conservation status

**(IUCN 2022).** Least Concern [LC]. EOO = 144,608 km^2^ [LC]; AOO = 108 km^2^ [EN]. *Solanumzuloagae* is a plant of roadsides and disturbed areas and occurs over a relatively wide range. The small AOO may be a result of collecting deficit but bears consideration. It has been found within protected areas in Argentina (e.g., Yungas Biosphere Reserve, Parque Nacional Baritú) and in the region of Parque Nacional Carrasco in Bolivia.

##### Discussion.

*Solanumzuloagae* is most similar morphologically to *S.huayavillense*, with which it is broadly, but not locally, sympatric. The species share large, membranous leaves with ciliate margins, lax stems that zig-zag over other vegetation, small flowers with stubby anthers and small fruits. *Solanumzuloagae* can be distinguished from *S.huayavillense* by calyx morphology (tube shorter than lobes versus longer than lobes in *S.huayavillense*), corolla colour (white versus yellow) and slightly longer (3–3.5 mm long versus 2.5–3 mm long) anthers. Berries of *S.zuloagae* consistently have eight stone cells of two sizes, while those of *S.huayavillense* either have four or the stone cells are completely absent.

## ﻿Doubtful names

SolanumnigrumL.var.aspergilliflorum Sendtn., Fl. Bras. (Martius) 10: 16. 1846. Type. Sin. loc. [Brazil?] (no specimens or collectors cited). Might be *S.chenopodioides* Lam. based on Sendtner’s notes “*S.chenopodioides* Lam.?” beneath description in the original publication, but the description mentions stellate hairs on the leaf undersides, a character that does not occur in the Morelloid clade.

*Solanumaurantium* Larrañaga, Escritos Damaso Antonio Larrañaga 2: 88. 1923. Type: 29 May 1812, *Larrañaga s.n.* (no type material located). The original herbarium of Damaso Larrañaga was destroyed and no specimens of these names and descriptions exist at MVM in Montevideo, Uruguay, where Larrañaga was based (Manuel Garcia, MVM, pers. communication, 9 July 2014).

*Solanumcremastanthemum* Werderm., Notizbl. Bot. Gart. Berlin-Dahlem 12: 378. 1935. Type: Ecuador: Tal des Rio Pastaza bei Rio Negro, Wald, ca. 1,250m, 11 Sep 1934, *L. Diels 885* (holotype B, destroyed, no duplicates found). The description specifies a plant with interaxillary inflorescence typical of the Morelloids, but large anthers up to 5–6 mm long. Morphological similarity to *S.probolospermum* (= *S.cochabambense*) is noted – this latter species has smaller anthers (ca. 3.5 mm), and no species of the Morelloids known to occur in Ecuador or nearby areas of Peru or Colombia has larger than 4 mm long anthers.

*Solanumhyemale* Biroli ex Colla, Herb. Pedem. 4: 275. 1835. Type: Of unknown origin, *Herb. G. Biroli* (holotype TO?) = *Physalis* sp. (ex. descr.)

*Solanumpigmaeum* Larrañaga, Escritos Damaso Antonio Larranaga 2: 88. 1923. Type: 15 May 1814, *Larrañaga s.n.* (no type material located). The original herbarium of Damaso Larrañaga was destroyed and no specimens of these names and descriptions exist at MVM in Montevideo, Uruguay, where Larranaga was based (Manuel Garcia, MVM, pers. communication, 9 July 2014). This is almost certainly a redescription of *S.pygmaeum* Cav.

## ﻿“Names” (designations) not validly published

Here we only list designations that can be referred to species native to the Americas. For the many designations associated with cultivated species outside of the Americas (e.g., *S.scabrum*) please see Särkinen et al. (2018). Article numbers refer to the Shenzhen Code (Turland et al. 2018).

*Solanumamaranthifolium* Gillies ex Rusby, Bull. Torrey Bot. Club 26: 152. 1899, nomen nudum; based on a Gillies manuscript name at Kew; two specimens collected by Gillies (K001166701, K001166704) are annotated “ S.amaranthifolium Gill.” in Gillies’ hand = *S.chenopodioides* Lam.

*Solanumasperum* Hornem. ex Walp., Repert. Bot. Syst. (Walpers) 3: 49. 1844, pro syn. *Solanumrumphii* Dunal = *S.americanum* Mill.

SolanumatriplicifoliumGillies ex Neesvar.minus Dunal, Prodr. [A. P. de Candolle] 13(1): 55. 1852, not intended as a new name, direct reference to Nees and Walpers publication of var. minus = *S.fragile* Wedd.

*Solanumchenopodioides* Hort. ex Dunal, Prodr. [A. P. de Candolle] 13(1): 55. 1852., pro syn. *Solanumatriplicifolium* Gillies ex Nees = *S.atriplicifolium* Gillies ex Nees.

Solanumchousboevar.merrillianum (T.N.Liou) C.Y.Wu & S.C.Huang. This citation from Flora of China (Zhang et al. 1994) that also appears on Tropicos (www.tropicos.org, accessed 12 August 2017) is a misprint and confounding of the attribution to Schousboe (Schousboe ex Willd.) with a specific epithet.

*Solanumdasystichanthum* Bitter, Herbarium name on an annotation label in Bitter’s hand on destroyed specimen of *Niederlein 262* from B [F neg. 2771] = *S.pilcomayense* Morong.

*Solanumdecurrens* Wall. ex Dunal, Prodr. [A. P. de Candolle] 13(1): 50. 1852, pro syn. *Solanumrhinozerothis* Blume = *S.americanum* Mill.

*Solanumgracile* Otto ex W.Baxter, in Loudon, Hort. Brit. 2, Suppl.: 639 1850, nomen nudum, also later homonym of *S.gracile* Sendtn. (1846) = identity uncertain.

*Solanumhastatum* Mattos & Guaranha, herbarium name on annotation slip on sheet at SP (*A.S. Costa & A.P. Viegas s.n.* SP025911) = *S.paucidens* Bitter.

*Solanumjahnii* Bitter ex Pittier, Cat. Fl. Venez. 2: 380. 1947, not validly published; no diagnosis or description in Latin (Art. 39.1) = *S.nigrescens* M.Martens & Galeotti.

*Solanummonttianum* Bitter: Herbarium name on an annotation label of Bitter’s in Vienna = *S.furcatum* Dunal.

*Solanummuricatum* Bertero ex Dunal, Prodr. [A. P. de Candolle] 13(1): 150. 1852, pro syn. *Solanumrancaguense* Dunal = *S.furcatum* Dunal.

SolanumnigrumL.subsp.chinense Filov, Kult. Fl. SSSR (Zhukovskii) 10: 382. 1958, not validly published; no diagnosis or description in Latin (Art. 39.1) = *S.americanum* Mill.

SolanumnigrumL.var.frutescens Macloskie, Rep. Princeton Univ. Exped. Patagonia 8: 707. 1905, nomen nudum = *S.nitidibaccatum*, *S.triflorum*, *S.chenopodioides* or *S.pygmaeum* all of which occur in northern Patagonia.

SolanumnigrumL.var.merrillianum (Liou) Filov, Kult. Fl. SSSR (Zhukovskii) 10: 383. 1958, as “merrilianum”, not validly published; no direct citation of basionym (Art. 38.1) = *S.americanum* Mill.

SolanumnigrumL.var.violaceum Chen ex Wessely, Feddes Repert. Spec. Nov. Regni Veg. 63(3): 293. 1960, nomen nudum; not intended as a new name, listed as one of the taxa accepted by Filov (1958, Kult. Fl. SSSR 20: 382) = *S.americanum* Mill.

*Solanumnodiflorum* Desv. ex Dunal, Prodr. [A. P. de Candolle] 13(1): 46. 1852, pro syn. *Solanumdesvauxii* Ham. = *S.americanum* Mill.

SolanumnodiflorumJacq.var.acuminatum Chodat, Bull. Herb. Boissier, sér. 2, 2: 811. 1902, not intended as a new name, as “*acuminatum* (?)”, with no specimen cited. In the rest of this work the new taxa are clearly indicated with “nob.” and a specimen (or several) cited = *S.americanum* Mill.

SolanumphoteinocarpumNakam. & Odash.var.violaceum C.Y.Wu & S.C.Huang, Acta Phytotax. Sin. 16(2): 72. 1978, nomen nudum; incorrectly cited in this publication as *violaceum* Chen ex Wessely but Wessely (1960) did not formally publish this name, instead she cited Filov’s (1958) list of accepted taxa. Filov did not provide Latin descriptions or diagnoses (Art. 38.1) and so all names in that work are not validly published. The varietal epithet should be attributed only to Wu and Huang (1978), but they do not provide a Latin diagnosis either (Art. 39.1) = *S.americanum* Mill.

*Solanumpasudodulcamaroides* Schaffer in Child, Feddes Repert. 95: 145. 1994, nomen nudum, no description or diagnosis; the word holotype not used (Art. 40.6). Based on “Schulte 1655 Valle de Mojiro herb. BM!” (= *Schmitz 1655*, Valle de Mejico, BM) = *S.corymbosum* Jacq.

*Solanumvirgatum* Endl. ex Sendtn., Fl. Bras. (Martius) 10: 13. 1846, pro syn. *Solanumgracile* Sendtn. = *S.chenopodioides* Lam.

## Supplementary Material

XML Treatment for Solanum albescens

XML Treatment for Solanum alliariifolium

XML Treatment for Solanum aloysiifolium

XML Treatment for Solanum americanum

XML Treatment for Solanum annuum

XML Treatment for Solanum antisuyo

XML Treatment for Solanum arenicola

XML Treatment for Solanum arequipense

XML Treatment for Solanum caatingae

XML Treatment for Solanum caesium

XML Treatment for Solanum chenopodioides

XML Treatment for Solanum cochabambense

XML Treatment for Solanum corymbosum

XML Treatment for Solanum dianthum

XML Treatment for Solanum echegarayi

XML Treatment for Solanum enantiophyllanthum

XML Treatment for Solanum fiebrigii

XML Treatment for Solanum fragile

XML Treatment for Solanum furcatum

XML Treatment for Solanum gilioides

XML Treatment for Solanum glandulosipilosum

XML Treatment for Solanum gonocladum

XML Treatment for Solanum grandidentatum

XML Treatment for Solanum huayavillense

XML Treatment for Solanum hunzikeri

XML Treatment for Solanum interandinum

XML Treatment for Solanum juninense

XML Treatment for Solanum leptocaulon

XML Treatment for Solanum longifilamentum

XML Treatment for Solanum macrotonum

XML Treatment for Solanum marmoratum

XML Treatment for Solanum michaelis

XML Treatment for Solanum nigrescens

XML Treatment for Solanum nitidibaccatum

XML Treatment for Solanum palitans

XML Treatment for Solanum pallidum

XML Treatment for Solanum paucidens

XML Treatment for Solanum pentlandii

XML Treatment for Solanum physalidicalyx

XML Treatment for Solanum physalifolium

XML Treatment for Solanum pilcomayense

XML Treatment for Solanum polytrichostylum

XML Treatment for Solanum profusum

XML Treatment for Solanum pseudoamericanum

XML Treatment for Solanum pygmaeum

XML Treatment for Solanum radicans

XML Treatment for Solanum rhizomatum

XML Treatment for Solanum riojense

XML Treatment for Solanum salamancae

XML Treatment for Solanum salicifolium

XML Treatment for Solanum sarrachoides

XML Treatment for Solanum scabrum

XML Treatment for Solanum sinuatiexcisum

XML Treatment for Solanum sinuatirecurvum

XML Treatment for Solanum subtusviolaceum

XML Treatment for Solanum tiinae

XML Treatment for Solanum triflorum

XML Treatment for Solanum tripartitum

XML Treatment for Solanum tweedieanum

XML Treatment for Solanum weddellii

XML Treatment for Solanum woodii

XML Treatment for Solanum zuloagae

## ﻿Excluded taxa

*Solanumconcarense* Hunz., Kurtziana 20: 190, fig. 2. 1989. Type. Argentina. San Luis: Chacabuco: Rumbo a Santa Rosa, Cerca de Concarán, viniendo desde Santa Rosa, 14 Jan 1960, *A.T. Hunziker & A.E. Cocucci 14547* (lectotype, designated by Barboza et al. 2013, pg. 243: CORD [CORD00004184]; isolectotype: CORD [CORD00004183]).

Barboza et al. (2013) included *S.concarense* in the morelloid solanums in Flora Argentina based on the few specimens available. Subsequent inclusion of this species in a molecular phylogenetic study (Gagnon et al. 2022) shows it is nested within the Dulcamaroid clade and a closer analysis of its morphology shows it shares many of the synapomorphies of that clade (e.g., pedicel sleeves, green “pseudo-nectaries”). A complete description of *S.concarense* can be found on Solanaceae Source (http://solanaceaesource.org/solanaceae/solanum-concarense).

## ﻿Additional information

### Author ORCIDs

Sandra Knapp https://orcid.org/0000-0001-7698-3945

Tiina Sarkinen https://orcid.org/0000-0002-6956-3093

Gloria E. Barboza https://orcid.org/0000-0003-1085-036X

### Data availability

All of the data that support the findings of this study are available in the main text or Supplementary Information and on the NHM Data Portal (https://doi.org/10.5519/3fh6f88q).

## Supplementary materials

Supplementary material 1 Index to numbered collections Data type collections by collector and number (exsiccatae list) Explanation note Here we only cite numbered collections examined for this monograph from plants collected in South America (incl. the Juan Fernández islands). First collectors in collections made by two or more collectors are listed here rather than complete collector strings. Collections by anonymous collectors without date or other identifying features are not included. Morelloids are prone to being found in mixed collections, so where we have identified these, or have identified the same number used for a different taxon, we have added [a] or [b] in square brackets. Any suffixes not in square brackets are part of the original collection. All collections seen and full collector strings can be found in the searchable csv files provided as Supplementary materials (SM 2 for South American collections; SM 3 for all collections of morelloids examined worldwide). These files are also available on the NHM Data Portal (https://doi.org/10.5519/3fh6f88q). https://binary.pensoft.net/file/897651This dataset is made available under the Open Database License (http://opendatacommons.org/licenses/odbl/1.0/). The Open Database License (ODbL) is a license agreement intended to allow users to freely share, modify, and use this Dataset while maintaining this same freedom for others, provided that the original source and author(s) are credited.Sandra Knapp, Tiina Sarkinen, Gloria E. Barboza

Supplementary material 2 Searchable csv file of South American specimens Data type specimens Explanation note Searchable csv file of South American (incl. the Juan Fernández Islands) morelloid specimens examined for this monograph. Column headings are: **Herbarium** = herbarium acronym according to Index Herbariorum; **Category** = specimen or photograph of specimen; **SpecID** = brahms specimen ID; **Brahms** = Brahms collection event ID; **Accession** = accession number of specimen; **Barcode** = barcode of specimen; **Collector** = principal collector; **Prefix** = collection number prefix (if applicable); **Number** = collection number; **Suffix** = collection number suffix (if applicable); **AddColl** = additional collectors; **Type** = type of type; **Type of** = type of what species; **Day** = day of collection; **Month** = month of collection; **Year** = year of collection; **DateRes** = date resolution (if applicable); **Family** = plant family; **Genus** = genus name; **Species** = species epithet; **Author** = species author name(s); **Continent** = TDWG continent; **Region** = TDWG region; **Country** = country of collection; **Majorarea** = first political division; **Minorarea** = second political division; **Gazetteer** = nearby town or place; **Locnotes** = locality; **Plantdesc** = description of the plant; **Habitattxt** = vegetation characteristics; **Lat** = latitude; **NS** = north or south; **Long** = longitude; **EW** = east or west; **LLunit** = coordinate units (DD = decimal degrees; DM = decimal minutes; DMS = degrees, minutes, seconds); **LLres** = coordinate resolution; **LLorig** = origin of coordinates; **LatLong** = coordinates in DMS; **LatDec** = decimal latitude; **LongDec** = decimal longitude; **Alt** = elevation minimum; **AltMax** = elevation maximum; **AltRes** = elevation resolution; **AltUnit** = elevation units (all metres); **AltRange** = elevational range; **Vernacular** = common name; **Language** = language of common name (if known). https://binary.pensoft.net/file/897652This dataset is made available under the Open Database License (http://opendatacommons.org/licenses/odbl/1.0/). The Open Database License (ODbL) is a license agreement intended to allow users to freely share, modify, and use this Dataset while maintaining this same freedom for others, provided that the original source and author(s) are credited.Sandra Knapp, Tiina Sarkinen, Gloria E. Barboza

Supplementary material 3 Searchable csv file of all specimens Data type specimens Explanation note Searchable csv file of all specimens examined for this monograph, including Africa, Asia, Australia, Europe, the Caribbean, North and Central America, the Pacific and South America. Column headings are: **Herbarium** = herbarium acronym according to Index Herbariorum; **Category** = specimen or photograph of specimen; **SpecID** = brahms specimen ID; **Brahms** = Brahms collection event ID; **Accession** = accession number of specimen; **Barcode** = barcode of specimen; **Collector** = principal collector; **Prefix** = collection number prefix (if applicable); **Number** = collection number; **Suffix** = collection number suffix (if applicable); **AddColl** = additional collectors; **Type** = type of type; **Type of** = type of what species; **Day** = day of collection; **Month** = month of collection; **Year** = year of collection; **DateRes** = date resolution (if applicable); **Family** = plant family; **Genus** = genus name; **Species** = species epithet; **Author** = species author name(s); **Continent** = TDWG continent; **Region** = TDWG region; **Country** = country of collection; **Majorarea** = first political division; **Minorarea** = second political division; **Gazetteer** = nearby town or place; **Locnotes** = locality; **Plantdesc** = description of the plant; **Habitattxt** = vegetation characteristics; **Lat** = latitude; **NS** = north or south; **Long** = longitude; **EW** = east or west; **LLunit** = coordinate units (DD = decimal degrees; DM = decimal minutes; DMS = degrees, minutes, seconds); **LLres** = coordinate resolution; **LLorig** = origin of coordinates; **LatLong** = coordinates in DMS; **LatDec** = decimal latitude; **LongDec** = decimal longitude; **Alt** = elevation minimum; **AltMax** = elevation maximum; **AltRes** = elevation resolution; **AltUnit** = elevation units (all metres); **AltRange** = elevational range; **Vernacular** = common name; **Language** = language of common name (if known). https://binary.pensoft.net/file/897653This dataset is made available under the Open Database License (http://opendatacommons.org/licenses/odbl/1.0/). The Open Database License (ODbL) is a license agreement intended to allow users to freely share, modify, and use this Dataset while maintaining this same freedom for others, provided that the original source and author(s) are credited.Sandra Knapp, Tiina Sarkinen, Gloria E. Barboza
