# Supplementary material for: ﻿A revision of the South American species of the Morelloid clade (Solanum L., Solanaceae)
Source: PhytoKeys. 2023 Aug 29;231:1–342. doi: 10.3897/phytokeys.231.100894 (PMC10481398; doi:10.3897/phytokeys.231.100894)
Supplement: Supplementary material 1 — Index to numbered collections [file phytokeys-231-001_article-100894__-s001.docx]

**Version**: 1 June 2023

# **Supplementary Material 1. Index to numbered collections**

Here we only cite numbered collections from plants collected in South America (incl. the Juan Fernández islands). First collectors in collections made by two or more collectors are listed here rather than complete collector strings. Collections by anonymous collectors without date or other identifying features are not included. Morelloids are prone to being found in mixed collections, so where we have identified these, or have identified the same number used for a different taxon, we have added [a] or [b] in square brackets. Any suffixes not in square brackets are part of the original collection. All collections seen and full collector strings can be found in the Supplementary Material searchable files (Suppl. Mat. 2 and 3). These files are also available on the NHM Data Portal (doi provided on acceptance).

Abbott, J.R. 15442 (radicans); 16181 (americanum).

Abrão, H.R.S. 5 (americanum).

Abruzzi, M.L. 1104 (americanum).

Acevedo Rodríguez, P. 4487 (palitans).

Achá, S. 162 (dianthum).

Ackermann, M. 414 (fragile).

Acosta, C. 69 (salamancae); 126 (palitans).

Acosta, J. 4 (radicans).

Acosta-Solís, M. 5351 (americanum); 5970 (interandinum); 6055 (americanum); 7020 (interandinum); 7139 (interandinum); 7314 (macrotonum); 7538 (grandidentatum); 7598 (grandidentatum); 7940 (macrotonum); 8022 (interandinum); 8655 (interandinum); 9152 (grandidentatum); 9207 (grandidentatum); 11201 (macrotonum); 12502 (radicans); 12919 (radicans); 14985 (interandinum).

Acra, L.A. 137 (americanum).

Adolfo 325 (physalifolium).

Adsersen, A. 174 (americanum); 506 (americanum).

Aedo, C. 10790 (americanum); 10806 (arequipense); 10979 (radicans); 10986 (grandidentatum); 11237 (grandidentatum); 14558 (polytrichostylum); 14576 (pallidum); 14693 (cochabambense); 14714 (sinuatiexcisum); 15299 (cochabambense).

Agostini, G. 00024 (nigrescens); 1031 (americanum).

Agra, M.F. 96 (americanum); 786 (americanum); 790 (americanum); 792 (americanum); 1108 (americanum); 1110 (americanum); 1114 (americanum); 1116 (americanum); 1245 (americanum); 1292 (americanum); 2111 (americanum); 3138 (americanum); 3892 (americanum); 5784 (americanum); 7232 (paucidens).

Agreda, D. 136 (gonocladum).

Agudelo, C.A. 1566 (macrotonum); 1703 (macrotonum).

Aguirre C, J. 483 (interandinum).

Aguirre F, P.H. 13 (interandinum).

Aguirre G, L.E. 192 (macrotonum).

Aguirre, E. 449 (physalidicalyx).

Ahumada, O. 350 (chenopodioides); 386 (pilcomayense); 861 (glandulosipilosum); 1085 (americanum); 1161 (paucidens); 4427 (aloysiifolium); 4773 (physalidicalyx); 4849 (physalidicalyx); 4908 (aloysiifolium); 5181 (physalidicalyx); 5758 (physalidicalyx); 7166 (fiebrigii); 8214 (zuloagae); 8275 (zuloagae); 8321 (aloysiifolium); 8551 (fiebrigii); 8560 (huayavillense); 8608 (aloysiifolium); 8705 (huayavillense); 8773 (fiebrigii); 8776 (huayavillense); 8781 (caesium); 8831 (aloysiifolium); 9045 (aloysiifolium); 9048 (aloysiifolium); 9093 (zuloagae); 9112 (aloysiifolium).

Alba, A. 19 (nigrescens).

Albán, J. 10240 (arequipense).

Albuquerque, J.M. de INPA-143090 (americanum); INPA-143091 (americanum).

Alexiades, M. 850052 (americanum).

Allard, H.A. 22042 (americanum); 22459 (americanum).

Allart, A. 313 (macrotonum).

Almeida Conceição, C. de 1415 (pilcomayense).

Almeida de Jesus, J. 1495 (americanum).

Almeida, J.A. 451 (americanum); 1969 (americanum).

Almeida, S.S. de 665 (americanum).

Almeida, T.E. 2302 (paucidens).

Alonso G, P. 19 (americanum).

Alonso, J.M. 250 (pygmaeum); 324 (pygmaeum); 350 (pygmaeum); 509 (chenopodioides).

Alston, A.H.G. 7118 (interandinum).

Altamirano, N. 987 (gonocladum).

Altamirano, S. JA 932C (dianthum); JA1080 (antisuyo); 3075 (leptocaulon); 3089 (leptocaulon).

Álvarez, A. 442 (antisuyo).

Álvarez, R. 28 (radicans); 343 (americanum); 437 (pilcomayense).

Alvear-P, M. 615 (nigrescens).

Alves, E.M. 493 (americanum).

Alvítez I, E. 1070 (cochabambense).

Amaral, A. 418 (americanum).

Amaral, I.L. 808 (americanum).

Ambrosetti, J.A. 38 (tripartitum); 160 (echegarayi); 1402 (triflorum); 1403 (triflorum); 1474 (triflorum); 1477 (salicifolium); 1478 (nitidibaccatum); 1485 (salicifolium); 1574 (echegarayi).

Amorim, A.M. 21 (scabrum); 8020 (americanum).

Amparo M, L. 83 (nigrescens).

Ana Virginia, L. 15 (macrotonum).

Ancuach, E. 686 (americanum).

Ancuash Atsut, E. 447 (americanum).

Anderson, B. 1920 (americanum).

Anderson, D.L. 1336 (pygmaeum); 1463 (triflorum); 1490 (salicifolium); 1511 (marmoratum); 1748 (pygmaeum); 1771 (salicifolium); 1902 (tweedieanum); 1914 (echegarayi); 1972 bis (tweedieanum); 2098 (tweedieanum); 2464 (nitidibaccatum); 2573 (tweedieanum); 2658 (tweedieanum); 2858 (tweedieanum); 2867 (pygmaeum); 2877 (tweedieanum); 2987 (tweedieanum); 3095 (physalidicalyx); 3126 (pygmaeum); 3168 (salicifolium); 3265 (nitidibaccatum); 3268 (salicifolium); 3271 (triflorum); 3300 (sarrachoides); 3416 (salicifolium); 3427 (marmoratum); 3428 (triflorum); 3475 (tweedieanum); 3476 (physalidicalyx); 3559 (tweedieanum); 3574 (salicifolium); 3592 (tweedieanum); 3594 (nitidibaccatum); 3609 (triflorum); 3643 (marmoratum); 3749 (triflorum); 3769 (salicifolium); 3793 (salicifolium); 4006 (tweedieanum).

Anderson, J. 10 (furcatum); 140 (furcatum).

Anderson, W.R. 9935 (americanum); 12349 (sinuatirecurvum); 36930 (chenopodioides).

Andrade, B.O. 147 (americanum); 302 (americanum).

Andrade, P. 1209 (paucidens).

Andrade, S.V. 274 (enantiophyllanthum); 1393 (enantiophyllanthum).

André, E. 638 (nigrescens); K-702 (grandidentatum); 715 (macrotonum); 1588 (macrotonum).

Andreas, C.H. 866 (furcatum).

Andreata, R. 180 (americanum).

Angulo, N. 1984 (radicans).

Antezana, C. 482 (fiebrigii).

Antezano, S. 093 (cochabambense).

Antúnez De Mayolo, K. 81 (arequipense).

Anzótegui, L. 373 (tweedieanum).

Apac, J. 04837 (radicans).

Aparecida da Silva, M. 2753 (americanum).

Aparecida, M. 2753 (americanum).

Apaza, O. 54 (cochabambense); 274 (cochabambense); 308 (fragile).

Apochian, C. 40 (echegarayi).

Apolo, D. 62493 (nitidibaccatum).

Aponte, H. 256 (americanum).

Arakaki, M. 34 (arequipense); 70 (corymbosum); 85 (americanum); 151 (radicans); 184 (corymbosum); 763 (grandidentatum).

Aranda, D. 11 (americanum).

Araque Molina, J. 19Ar506 (aloysiifolium); 882 (tweedieanum).

Araújo, F. 333-AE (americanum).

Araújo-M, A. 1977 (americanum); 2071 (longifilamentum); 3107 (americanum); 3130 (arenicola); 3787 (cochabambense).

Arbeláez, E.P. 6002 (interandinum).

Arbo, M.M. 87 (pilcomayense); 1019 (americanum); 7044 (pilcomayense); 7894 (pilcomayense); 7920 (americanum); 7990 (pilcomayense); 9398 (pilcomayense); 9436 (pilcomayense).

Árbocz, G.F. 594 (chenopodioides).

Årbocz, G.F. 2834 (enantiophyllanthum).

Archer, W.A. 44 (americanum); 1284 (nigrescens); 4197 (americanum).

Archibald, J.C. 14162 (echegarayi).

Arenas P, G. 13 (grandidentatum); 59 (arequipense); 117 (grandidentatum); 145 (grandidentatum).

Arenas T, J. 4 (cochabambense).

Arenas, J.M. 494 (nigrescens).

Arenas, P. 822 (tripartitum).

Argañaras, J.L. 120 (tweedieanum).

Argent, G.C.G. 6362 (americanum).

Argüello, A. 118 (interandinum).

Ariste-Joseph [Brother] B11 (nigrescens).

Ariza E, L. 3621 (chenopodioides).

Ariza Espinar, L. 223 (chenopodioides); 572 (tweedieanum); 1559 (chenopodioides); 2563 (aloysiifolium); 2679 (tweedieanum); 2798 (salicifolium); 3289 (tweedieanum); 3621 (chenopodioides).

Arroyo P, L. 4328 (aloysiifolium); 4407 (fiebrigii); 4435 (fiebrigii); 4650 (fiebrigii).

Arroyo R, F. 018 (arequipense); 040 (grandidentatum); 043 (grandidentatum).

Arroyo, L. 1823 (fiebrigii); 1843 (aloysiifolium); 3211 (aloysiifolium); 3568 (dianthum).

Arroyo, S.C. 27585 (aloysiifolium).

Arteta B, M.C. 330 (gonocladum).

Articó, L. 240 (triflorum); 249 (triflorum).

Asplund, E. 615 (pentlandii); 4080 (cochabambense); 5283 (americanum); 5837 (gonocladum); 5850 (fragile); 5901 (interandinum); 5982 (interandinum); 6240 (interandinum); 6552 (macrotonum); 8310 (grandidentatum); 8941 (americanum); 11448 (fragile); 15390 (americanum); 15448 (longifilamentum); 16264 (interandinum); 16600 (americanum); 16911 (macrotonum); 17889 (interandinum); 18053 (americanum); 18633 (macrotonum); 19851 (americanum); 20203 (interandinum); 20534 (macrotonum).

Astegiano, M.E. 113 (chenopodioides).

Atchison, G. 18 (aloysiifolium); 19 (glandulosipilosum); 20 (huayavillense); 24 (annuum); 25 (palitans).

Autran, E.J.B. 4 (pygmaeum).

Avilán, L. 19 (nigrescens).

Ayerbe, J.R. 194 (longifilamentum).

Aymard, G. 4942 (nigrescens).

Baar, R. 236a (cochabambense).

Badcock, W.J. 587 (tripartitum); 717 (leptocaulon); 846 (fiebrigii).

Baer, G.A. 16 (aloysiifolium).

Baez, C. 104 (enantiophyllanthum).

Báez, J.R. 31 (chenopodioides);106 (triflorum).

Baez, S. 31 (interandinum); 32B (macrotonum).

Bailetti, E. 145 (glandulosipilosum); 201 (physalidicalyx).

Bailey, L.H. 564 (nigrescens).

Bailey, P. 73 (interandinum).

Baines, R. 333 (grandidentatum).

Balcázar S, J. 355 (rhizomatum).

Balcázar, J. 368 (woodii).

Baldeón Malpartida, S. 7514 (cochabambense).

Baldi, J. 24 (tweedieanum).

Balech, E. 49 (pygmaeum).

Balls, E.K. 37 (cochabambense); 57 (tweedieanum); B5891 (gonocladum); 5892 (gonocladum); B5906 (sinuatirecurvum); 5915 (profusum); B5949 (cochabambense); 6057 (cochabambense); B6071 (fiebrigii); 6077 (weddellii); 6111 (hunzikeri); B6203 (cochabambense); B6231 (gonocladum); B6247 (leptocaulon); B6782 (pallidum); B6783 (pallidum); 6793 (polytrichostylum); 6806 (cochabambense); 6815 (cochabambense); 6901 (cochabambense); 7113 (interandinum); B7516 (macrotonum); 7528 (macrotonum).

Balslev, H. 1493 (interandinum); 2070 (macrotonum); 2172 (interandinum); 4650 (interandinum); 69247 (interandinum).

Bang, M. 1 (tripartitum); 10 (tripartitum); 31[a] (polytrichostylum); 31[b] (pallidum); 64[a] (pallidum); 64[b] (gonocladum); 539 (americanum); 740 (sinuatiexcisum); 938 (gilioides); 1151 (cochabambense); 1159 (physalifolium); 1462 (americanum); 1575 (albescens); 1781 (sinuatiexcisum); 1914 (leptocaulon); 2023 (cochabambense); 2024 (leptocaulon); 2062 (palitans); 2392 (subtusviolaceum); 2492 (dianthum); 2515 (gonocladum); 2517 (pallidum); 2518 (pallidum); 2870 (pallidum).

Barbosa-Silva, R.G. 397 (enantiophyllanthum).

Barbour, P. 3958 (longifilamentum); 4088 (antisuyo).

Barbour, P.J. 4088 (antisuyo).

Barboza, G.E. 62 (chenopodioides); 69 (triflorum); 73 (triflorum); 84 bis (tripartitum); 95 (palitans); 111 (aloysiifolium); 114 (aloysiifolium); 126 (salicifolium); 136 (physalidicalyx); 137 (physalidicalyx); 138 (physalidicalyx); 139 (tiinae); 140 (tiinae); 142 (physalidicalyx); 143 (physalidicalyx); 150 (tiinae); 151 (tiinae); 152 (tiinae); 185 (salicifolium); 188 (americanum); 190 (nitidibaccatum); 197 (nitidibaccatum); 204 (echegarayi); 221 (salicifolium); 230 (salicifolium); 273 (aloysiifolium); 278 (aloysiifolium); 280 (aloysiifolium); 286 (cochabambense); 287 (zuloagae); 305 (aloysiifolium); 308 (huayavillense); 311 (zuloagae); 315 (tweedieanum); 325 (tweedieanum); 336 (salicifolium); 343 (chenopodioides); 348 (americanum); 352 (pilcomayense); 360 (pilcomayense); 425 (americanum); 530 (americanum); 553 (salicifolium); 554 (tweedieanum); 561 (salicifolium); 566 (chenopodioides); 570 (tweedieanum); 584 (riojense); 588 (salicifolium); 605 (palitans); 610 (tweedieanum); 615 (tweedieanum); 619 (tweedieanum); 622 (tweedieanum); 628 (aloysiifolium); 642 (aloysiifolium); 646 (aloysiifolium); 656 (aloysiifolium); 657 (tripartitum); 692 (aloysiifolium); 696 (physalidicalyx); 703 (cochabambense); 729 (riojense); 731 (sinuatirecurvum); 740 (physalidicalyx); 745 (aloysiifolium); 779 (aloysiifolium); 840 (pilcomayense); 844 (tweedieanum); 848 (cochabambense); 866 (aloysiifolium); 877 (salicifolium); 882 (aloysiifolium); 884 (aloysiifolium); 897 (americanum); 994 (americanum); 998 (pilcomayense); 1037 (americanum); 1055 (caesium); 1071 (aloysiifolium); 1072 (aloysiifolium); 1124 (cochabambense); 1139 (cochabambense); 1151 (tweedieanum); 1170 (chenopodioides); 1176 (tweedieanum); 1184 (triflorum); 1227 (triflorum); 1258 (tweedieanum); 1271 (aloysiifolium); 1276 (aloysiifolium); 1306 (salicifolium); 1356 (sinuatirecurvum); 1434 (sinuatirecurvum); 1542 (pilcomayense); 1560 (chenopodioides); 1574 (macrotonum); 1575 (macrotonum); 1579 (nigrescens); 1598 (americanum); 1603 (americanum); 1644 (paucidens); 1658 (americanum); 1705 (tiinae); 1709 (tweedieanum); 1715 (physalidicalyx); 1721 (aloysiifolium); 1769 (physalifolium); 1774 (zuloagae); 1776 (huayavillense); 1785 (sinuatirecurvum); 1788 (weddellii); 1792 (sinuatirecurvum); 1809 (physalidicalyx); 1812 (americanum); 1829b (polytrichostylum); 1830 (subtusviolaceum); 1831 (sinuatiexcisum); 1839 (dianthum); 1840 (antisuyo); 1846 (sinuatiexcisum); 1848 (longifilamentum); 1855 (cochabambense); 1864 (sinuatiexcisum); 1865 (polytrichostylum); 1880 (cochabambense); 1890 (cochabambense); 1908 (aloysiifolium); 1917 (chenopodioides); 1929 (salicifolium); 1938 (salicifolium); 1998 (tweedieanum); 2017 (americanum); 2045 (enantiophyllanthum); 2046 (enantiophyllanthum); 2054 (enantiophyllanthum); 2091 (americanum); 2128 (chenopodioides); 2137 (aloysiifolium); 2139 (americanum); 2142 (aloysiifolium); 2152 (cochabambense); 2153 (cochabambense); 2158 (physalidicalyx); 2163 (tweedieanum); 2167 (tiinae); 2172 (tiinae); 2176 (annuum); 2177 (tweedieanum); 2178 (palitans); 2182 (salamancae); 2191 (salamancae); 2194 (tripartitum); 2202 (zuloagae); 2208 (zuloagae); 2210 (aloysiifolium); 2216 (aloysiifolium); 2219 (aloysiifolium); 2223 (physalifolium); 2228 (palitans); 2229 (physalifolium); 2234 (sinuatirecurvum); 2240 (caesium); 2243 (aloysiifolium); 2249 (caesium); 2250 (caesium); 2252 (huayavillense); 2253 (aloysiifolium); 2255 (huayavillense); 2256 bis (fiebrigii); 2279 (pilcomayense); 2282 (pilcomayense); 2287 (pilcomayense); 2291 (huayavillense); 2292 (chenopodioides); 2309 (pygmaeum); 2316 (chenopodioides); 2317 (chenopodioides); 2343 (triflorum); 2345 (triflorum); 2351 (triflorum); 2399 (furcatum); 2429 (triflorum); 2463 (tweedieanum); 2489 (physalidicalyx); 2491 (physalidicalyx); 2513 (americanum); 2531 (americanum); 2695 (salicifolium); 2704 (salicifolium); 2709 (salicifolium); 2715 (riojense); 2740 (salicifolium); 2784 (aloysiifolium); 2811 (salicifolium); 2812 (salicifolium); 2845 (salicifolium); 2960 (salicifolium); 2962 (echegarayi); 3001 (fragile); 3006 (cochabambense); 3007 (cochabambense); 3014 (tiinae); 3017 (annuum); 3019 (tiinae); 3023 (annuum); 3027 (annuum); 3043 (annuum); 3058 (americanum); 3082 (pilcomayense); 3089 (echegarayi); 3148 (salicifolium); 3158 (salicifolium); 3224 (salicifolium); 3225 (salicifolium); 3245 (salicifolium); 3253 (riojense); 3269 (salicifolium); 3333 (tweedieanum); 3363 (tweedieanum); 3368 (interandinum); 3375 (arequipense); 3378 (grandidentatum); 3379 (arequipense); 3384 (interandinum); 3389 (interandinum); 3390 (interandinum); 3394 (arequipense); 3396 (arequipense); 3398 (grandidentatum); 3400 (pentlandii); 3402 (cochabambense); 3403 (cochabambense); 3407 (polytrichostylum); 3413 (pallidum); 3416 (pallidum); 3421 (pallidum); 3422 (antisuyo); 3426 (cochabambense); 3427 (cochabambense); 3441 (tweedieanum); 3447 (echegarayi); 3448 (tweedieanum); 3450 (echegarayi); 3456 (tweedieanum); 3458 (tweedieanum); 3460 (tweedieanum); 3462 (palitans); 3465 (salicifolium); 3466 (salicifolium); 3471 (palitans); 3472 (tweedieanum); 3473 (salicifolium); 3474 (tweedieanum); 3475 (weddellii); 3478 (salicifolium); 3483 (tweedieanum); 3485 (salicifolium); 3486 (tweedieanum); 3488 (salicifolium); 3491 (tiinae); 3492 (tweedieanum); 3494 (salicifolium); 3495 (annuum); 3496 (tiinae); 3497 (sinuatiexcisum); 3500 (cochabambense); 3504 (glandulosipilosum); 3505 (aloysiifolium); 3506 (aloysiifolium); 3508 (aloysiifolium); 3510 (glandulosipilosum); 3513 (aloysiifolium); 3515 (aloysiifolium); 3520 (glandulosipilosum); 3521 (aloysiifolium); 3523 (aloysiifolium); 3526 (aloysiifolium); 3527 (aloysiifolium); 3528 (aloysiifolium); 3530 (caesium); 3531 (huayavillense); 3532 (aloysiifolium); 3536 (huayavillense); 3539 (aloysiifolium); 3541 (caesium); 3544 (palitans); 3546 (glandulosipilosum); 3548 (fiebrigii); 3550 (weddellii); 3551 (sinuatirecurvum); 3557 (sinuatirecurvum); 3561 (tripartitum); 3563 (tripartitum); 3564 (tweedieanum); 3565 (glandulosipilosum); 3566 (aloysiifolium); 3569 (zuloagae); 3570 (palitans); 3596 (aloysiifolium); 3601 (sinuatirecurvum); 3604 (riojense); 3611 (tripartitum); 3613 (weddellii); 3617 (aloysiifolium); 3623 (cochabambense); 3625 (cochabambense); 3628 (cochabambense); 3668 (marmoratum); 3682 (triflorum); 3687 (triflorum); 3826 (salicifolium); 3839 (salicifolium); 3881 (riojense); 3936 (pentlandii); 3983 (physalidicalyx); 4026 (nitidibaccatum); 4210 (physalidicalyx); 4211 (nitidibaccatum); 4359 (sinuatirecurvum); 4383 (sinuatirecurvum); 4451 (triflorum); 4626 (aloysiifolium); 4647 (salicifolium); 4703 (hunzikeri); 4739 (echegarayi); 4753 (sinuatirecurvum); 4763 (hunzikeri); 4769 (tweedieanum); 4783 (echegarayi); 4798 (tweedieanum); 4799 (weddellii); 4834 (longifilamentum); 4905 (alliariifolium); 4928 (profusum); 4936 (sarrachoides); 5051 (aloysiifolium); 5059 (chenopodioides); 5060 (tweedieanum); 5066 (tweedieanum); 5072 (tweedieanum); 5073 (marmoratum); 5079 (marmoratum); 5080 (tweedieanum); 5087 (tweedieanum); 5094 (marmoratum); 5099 (marmoratum); 5106 (chenopodioides); 5121 (salicifolium); 5128 (salicifolium); 5130 (marmoratum); 5134 (pygmaeum); 5136 (marmoratum); 5140 (echegarayi); 5141 (physalidicalyx); IM-5142 (pygmaeum); 5156 (echegarayi); 5157 (echegarayi); 5158 (echegarayi); 5159 (tweedieanum).

Barclay, A.S. 3235 (macrotonum).

Barclay, G.W. 2301 (pentlandii).

Barclay, H.G. 7288 (interandinum); 8038 (interandinum); 8706 (interandinum).

Barfod, A. 41234 (interandinum); 41284 (interandinum).

Barker, P.R. 69 (leptocaulon); 326 (tripartitum).

Barkley, F.A. 189 (salicifolium); 1623 (nigrescens); 1775 (nigrescens); 1775 (nigrescens); 38774 (interandinum).

Barra, N. De La 640 (radicans).

Barragán-Fonseca, K. 9 (macrotonum).

Barros, A.A.M. de 2267 (americanum); 3504 (americanum); 5160 (americanum).

Barroso, R.M. 131 (americanum); 135 (americanum).

Barth, O.M. I-104 (enantiophyllanthum); I-207 (paucidens).

Bartlett, H.H. 19404 (salicifolium); 19427 (salicifolium); 19715 (tweedieanum); 19969 (tweedieanum); 20314 (aloysiifolium); 20623 (salicifolium); 20944 (chenopodioides).

Bascopé, F. 429 (dianthum).

Bassam, M.J. 8202 (americanum).

Bassignana, F. 1904 (triflorum); 9131 (pygmaeum).

Bastián, E. 204 (palitans); 815 (cochabambense); 964 (physalifolium); 1289 (palitans).

Bastos, B.C. 134 (americanum).

Batista, T.A. 264 (enantiophyllanthum).

Bauen, I. 37 (americanum).

Baxter, P.R. 24 (furcatum).

Bayma, I.A. 285 (americanum).

Bazzi, R. 248 (pilcomayense).

Beck, S.G. 685 (tripartitum); 786 (cochabambense); 1390 (palitans); 2130a (pallidum); 2270 (palitans); 2374 (gonocladum); 3508 (palitans); 3963 (gonocladum); 4580 (weddellii); 4592 (pentlandii); 4979 (leptocaulon); 5479 (arenicola); 6048 (tripartitum); 6119 (pallidum); 6330 (aloysiifolium); 7765 (pallidum); 7980 (gonocladum); 8386 (antisuyo); 8865 (tripartitum); 11375 (sinuatiexcisum); 11469 (aloysiifolium); 11788 (fragile); 11900 (gonocladum); 11961 (pallidum); 12935 (dianthum); 14091 (fiebrigii); 14145 (sinuatirecurvum); 14153 (fragile); 14347 (gonocladum); 14928 (dianthum); 14955a (subtusviolaceum); 17234 (pallidum); 17695 (dianthum); 17932 (gonocladum); 22252 (subtusviolaceum); 22982 (pallidum); 23733 (tripartitum); 26056 (cochabambense); 26822 (physalifolium); 26881 (aloysiifolium); 27781 (cochabambense); 29206 (dianthum).

Becker, B. 1158 (cochabambense).

Bellard, E.P. 240 (interandinum).

Bello, M.A. 498 (nigrescens); 613 (nigrescens).

Beltrán, G.D. 21 (americanum); 65 (nigrescens); 71 (macrotonum); 72 (macrotonum); 84 (nigrescens); 94 (nigrescens); 176 (macrotonum); 189 (americanum); 195 (americanum); 196 (longifilamentum).

Beltrán, H. 205 (arequipense); 6453 (grandidentatum); 016642 (polytrichostylum).

Bender, K. 28 (tripartitum).

Benítez de Rojas, C.E. 2[a] (nigrescens); 104 (nigrescens); 232 (nigrescens); 309 (nigrescens); 409 (nigrescens); 430 (nigrescens); 453 (nigrescens); 558 (nigrescens); 700 (nigrescens); 721 (nigrescens); 1561 (nigrescens); 2506 (macrotonum); 2513 (nigrescens); 2981 (americanum); 4190 (nigrescens); 4441 (macrotonum).

Bennet, B. 1942 (pallidum); 1948 (pallidum).

Benoist, R. 2287 (macrotonum).

Benol, D.M. 3 (americanum).

Bensman, R. 177 (americanum); 396 (interandinum); 491 (longifilamentum).

Bentley, P.S. 55 (americanum); 169 (americanum); 306 (americanum).

Bento, M.S. 54 (americanum).

Berg, C. 117 (chenopodioides); 137 (pygmaeum).

Berg, C.C. P-19836 (americanum).

Berlin, B. 1682 (americanum); 1965 (americanum); 3671 (americanum).

Bernacci, L.C. 25855 (americanum); 25856 (americanum); 25857 (americanum).

Bernal G, C.A. 300 (macrotonum); 525 (macrotonum); 551 (macrotonum); 707 (interandinum); 871 (interandinum).

Bernal, R. 1718 (nigrescens); 1732 (macrotonum); 2670 (americanum).

Bernardello, G.L. 167 (pilcomayense); 171 (pilcomayense); 459 (chenopodioides); 476 (pygmaeum); 478 (chenopodioides); 502 (americanum); 730 (chenopodioides); 731 (chenopodioides); 794 (americanum); 819 (pilcomayense).

Bernardi, L. 6112 (macrotonum).

Berro, M.B. 1458 (sarrachoides); 7206 (sarrachoides).

Bertero, C.L.G. 633 (furcatum); 636 (nitidibaccatum); 637 (nitidibaccatum); 638 (furcatum); 1324 (furcatum).

Bertoncini, A.P. 1091 (americanum).

Betancourt A, C. 28 (americanum).

Betancur, J.C. 1405 (americanum); 5768 (nigrescens); 9533 (macrotonum).

Bettfreund, C. 21 (chenopodioides); 207 (fiebrigii).

Beuther, A. 58 (nigrescens).

Bianco, C.A. 298 (triflorum).

Bicudo, L.R.H. 19 (chenopodioides).

Biganzoli, F. 130 (americanum).

Billiet, F. 5526 (furcatum); 6073 (huayavillense).

Biloni, J.S. 6206 (tweedieanum); 6206 (tweedieanum); 6555 (tripartitum); 6602 (aloysiifolium).

Binder, M. 1999/356 (radicans).

Binot, A. 34 (americanum).

Biurrun, F. 728 (tweedieanum); 959 (salicifolium); 1711 (nitidibaccatum); 1767 (nitidibaccatum); 1889 (nitidibaccatum); 2045 (tweedieanum); 2160 (nitidibaccatum); 2669 (tweedieanum); 2672 (salicifolium); 2682 (tweedieanum); 2862 (tweedieanum); 3133 (tweedieanum); 3211 (nitidibaccatum); 3216 (tweedieanum); 3564 (tweedieanum); 3605 (tweedieanum); 4261 (triflorum); 4281 (nitidibaccatum); 4628 (tweedieanum); 4739 (echegarayi); 4844 (physalidicalyx); 5038 (echegarayi); 5045 (salicifolium); 5121 (salicifolium); 5398 (tweedieanum); 5780 (echegarayi); 5822 (tweedieanum); 5997 (echegarayi); 6086 (nitidibaccatum); 6155 (nitidibaccatum); 6163 (chenopodioides); 6234 (tweedieanum).

Blake, A.L. 175 (triflorum); 186 (nitidibaccatum).

Blake, M.E. 116 (nitidibaccatum).

Blanchard, M. 46 (arequipense).

Blanchet, J.S. 183 (americanum); 869 (americanum); 2322 (paucidens).

Blanco, N. 26 (nigrescens).

Böcher, T.W. 200 (americanum).

Boeke, J.D. 1359 (pentlandii).

Boelcke, O. 56a (chenopodioides); 87 (riojense); 138 (chenopodioides); 248 (furcatum); 275 (furcatum); 286 (furcatum); 1225 (chenopodioides); 1226 (chenopodioides); 3009 (pygmaeum); 3653 (furcatum); 3915 (furcatum); 4201 (triflorum); 6383 (chenopodioides); 6464 (furcatum); 6558 (furcatum); 6645 (chenopodioides); 8709 (chenopodioides); 9873 (salicifolium); 9925 (echegarayi); 10423 (triflorum); 11974 (pygmaeum); 12262 (triflorum); 12670 (triflorum); 12954 (triflorum); 15540 (tweedieanum); 16088 (triflorum); 16250 (triflorum); 16853 (furcatum).

Bohs, L. 1954 (interandinum); 1986 (gonocladum); 2088 (tripartitum); 2095 (aloysiifolium); 2099 (sarrachoides); 2101 (aloysiifolium); 2135 (pentlandii); 2769 (aloysiifolium); 2784 (fiebrigii); 2794 (gonocladum); 2796 (palitans); 3016 (aloysiifolium); 3021 (aloysiifolium); 3023 (physalifolium); 3025 (aloysiifolium); 3034 (aloysiifolium); 3042 (cochabambense); 3109 (corymbosum); 3113 (interandinum); 3148 (radicans); 3189a (americanum); 3309 (americanum); 3312 (interandinum); 3321 (antisuyo); 3354 (longifilamentum); 3408 (longifilamentum); 3409 (longifilamentum); 3434 (longifilamentum); 3617 (macrotonum).

Boldrini, I. 277 (americanum).

Bonifácino, J.M. 475 (nitidibaccatum).

Bonilha, C. 313 (americanum).

Bonpland, A. 386 (pilcomayense).

Boone, W. 199 (americanum); 510 (americanum).

Borges, R. 1080 (pilcomayense).

Bornmüller, A. 565 (americanum).

Borsini, O.H. 1154 (tweedieanum).

Bortagaray, J. 99 (aloysiifolium).

Boto, M.M.P. 70 (americanum).

Bourdy, G. GB-3710 (polytrichostylum).

Bovini, M.G. 1056 (americanum); 1827 (americanum); 2207 (americanum); 2884 (americanum); 3646 (americanum).

Brack, P. 1658 (americanum); 1789 (americanum).

Brade, A.C. 9337 (paucidens); 14647 (enantiophyllanthum); 16429 (paucidens); 16952 (enantiophyllanthum); 20315 (enantiophyllanthum); 21276 (enantiophyllanthum).

Braga, J.M.A. 11 (americanum); 293 (americanum); 636 (americanum); 3056 (americanum); 3191 (enantiophyllanthum); 3701 (enantiophyllanthum).

Brandbyge, J.S. 42414 (nitidibaccatum); 42417 (radicans).

Brasil, I. 105 (americanum).

Bravo, E. 338 (interandinum); 632 (interandinum).

Bridges, T. 400 (nitidibaccatum).

Bristol, M.L. 921 (interandinum).

Britez, R.M. 2046 (americanum).

Brizuela, A. 543 (nitidibaccatum); 1042 (sarrachoides); 1077 (nitidibaccatum); 1158 (tweedieanum); 1319 (tweedieanum); 1339 (tweedieanum); 1621 (chenopodioides).

Brizuela, J. 345 (tweedieanum); 619 (tweedieanum); 1146 (physalidicalyx); 1216 (tweedieanum).

Broadway, W.E. 258 (nigrescens); 791 (nigrescens).

Brollo, M.E. 448 (americanum); 482 (pilcomayense).

Brooke, W.M.A. 5011 (palitans); 5015 (tripartitum); 5062 (gonocladum); 5063 (gonocladum); 5125 (cochabambense); 5148 (cochabambense); 5234 (fragile); 5434 (cochabambense); 5454 (cochabambense); 5851 (fiebrigii); 6038 (leptocaulon); 6590 (dianthum); 6802 (leptocaulon); 6881 (dianthum); 6905 (albescens); 6944 (leptocaulon).

Brooks, R.R. MS-128 (salicifolium); MS-178 (salicifolium).

Brown, ? [Argentina] 1757 (sinuatiexcisum).

Bruch, C. 38944 (tweedieanum).

Brunel, G.R. 181 (pentlandii); 410 (cochabambense).

Brunner, D.R. 859 (americanum); 1084 (pilcomayense); 1230 (pilcomayense); 1272 (americanum); 1298 (americanum); 1639 (sarrachoides).

Buchtien, O. 117 (gonocladum); 119 (polytrichostylum); 150 (echegarayi); 181 (leptocaulon); 290 (polytrichostylum); 331 (pallidum); 332 (pallidum); 463 (antisuyo); 465 (pallidum); 466 (pentlandii); 467 (gonocladum); 468 (polytrichostylum); 537 (gonocladum); 559 (pallidum); 601 (tripartitum); 618 (tripartitum); 659[a] (palitans); 659[b] (pallidum); 763 (polytrichostylum); 765[b] (pallidum); 765 (pallidum); 768 (dianthum); 769 (pentlandii); 772 (weddellii); 1443 (americanum); 2411 (cochabambense); 2412 (cochabambense); 2960 (polytrichostylum); 2962 (sinuatiexcisum); 2963 (gonocladum); 2964 (gonocladum); 2964[a] (gonocladum); 2964[b] (gonocladum); 2965 (pentlandii); 2966 (pentlandii); 2968 (polytrichostylum); 2969 (gonocladum); 2971 (pallidum); 3247 (americanum); 3249 (polytrichostylum); 3250 (polytrichostylum); 3253 (polytrichostylum); 3258 (pallidum); 3259 (pallidum); 3260 (pallidum); 3885 (dianthum); 3886 (polytrichostylum); 3887 (antisuyo); 3888 (dianthum); 3889 (radicans); 3890 (americanum); 3891 (arenicola); 3980 (tripartitum); 3981 (gonocladum); 3983 (gonocladum); 3986 (gonocladum); 3987 (pentlandii); 3988 (pentlandii); 4027[a] (longifilamentum); 4027[b] (americanum); 4383 (americanum); 4384 (americanum); 4387 (pallidum); 4450 (gonocladum); 4451 (palitans); 4452 (gonocladum); 4453 (gonocladum); 4458 (tripartitum); 5548 (americanum); 5549 (longifilamentum); 5550 (americanum); 5838 (gonocladum); 5846 (polytrichostylum); 5856 (pentlandii); 5859 (leptocaulon); 8426 (gonocladum); 8517 (gonocladum); 8650 (gonocladum); 8651 (gonocladum); 8652 (gonocladum); 8654 (gonocladum); 8665 (gonocladum); 8754 (polytrichostylum); 9022 (albescens); 9028 (polytrichostylum); 9029 (antisuyo); 9033 (antisuyo).

Budin, E. 6498 (sinuatirecurvum); 7471 (tiinae).

Buendía S, M.C. 23 (americanum).

Bueno, O. 356 (americanum); 680 (paucidens); 896 (americanum); 976 (americanum); 1685 (americanum); 2726 (americanum); 4284 (americanum).

Bufrem, A. 318 (americanum).

Bunting, G.S. 5572 (americanum); 11364 (nigrescens).

Burkart, A. 5015 (pygmaeum); 6303 (nitidibaccatum); 7490 (salicifolium); 7693 (chenopodioides); 7695 (nitidibaccatum); 8272 (pilcomayense); 8276 (sarrachoides); 8284 (salicifolium); 8891 (pygmaeum); 8912 (sarrachoides); 9062 (pygmaeum); 9306 (echegarayi); 9875 (triflorum); 9963 (triflorum); 9966 (tweedieanum); 10557 (chenopodioides); 10932 (triflorum); 11077 (physalidicalyx); 11176 (palitans); 11943 (tripartitum); 12559 (tweedieanum); 12571 (nitidibaccatum); 12572 (tweedieanum); 13220 (profusum); 13227 (fiebrigii); 13953 (triflorum); 15559 (fiebrigii); 15574 (pygmaeum); 15929 (triflorum); 15973 (triflorum); 17868 (pygmaeum); 18056 (pygmaeum); 18214 (pygmaeum); 19874 (nitidibaccatum); 20934 (pilcomayense); 21902 (chenopodioides); 22108 (sinuatiexcisum); 22721 (pygmaeum); 23822 (tweedieanum); 24253 (pygmaeum); 24931 (pygmaeum); 25444 (tweedieanum); 26043 (pygmaeum); 26360 (pygmaeum); 26602 (aloysiifolium); 27086 (nitidibaccatum); 28046 (tweedieanum); 28197 (pygmaeum); 28858 (chenopodioides); 30618 (fiebrigii); 30621 (caesium); 30621 (caesium).

Bussmann, R.W. 15721 (antisuyo); 15823 (longifilamentum); 16773 (juninense); 16908 (juninense); 17047 (interandinum); 17048 (interandinum); 17052 (cochabambense); 18201 (juninense); 18372 (juninense); 168800 (juninense).

Bye, R.A. 16922 (chenopodioides); 26929 (nitidibaccatum).

Caballero, L.M. 101 (macrotonum).

Cabanillas Soriano, J.M. 466 (interandinum); 562 (interandinum).

Cabezas, V. 49 (sinuatirecurvum).

Cabral, A. 569 (pilcomayense).

Cabrera R, I. 3694 (americanum); 7893 (nigrescens).

Cabrera, A.L. 37 (sinuatirecurvum); 82 (weddellii); 89 (sinuatirecurvum); 3132 (aloysiifolium); 4099 (aloysiifolium); 5024 (furcatum); 5267 (chenopodioides); 6065 (pygmaeum); 9332 (sinuatirecurvum); 9402 (weddellii); 9403 (weddellii); 10626 (pilcomayense); 10706 (chenopodioides); 11089 (pygmaeum); 11976 (tripartitum); 12021 (palitans); 12052 (tripartitum); 12905 (tweedieanum); 13188 (cochabambense); 13903 (aloysiifolium); 13926 (tripartitum); 13964 (riojense); 14026 (gilioides); 14078 (weddellii); 14262 (aloysiifolium); 14409 (physalidicalyx); 14572 (aloysiifolium); 14614 (aloysiifolium); 15098 (salicifolium); 15142 (tripartitum); 15160 (sinuatirecurvum); 15736 (aloysiifolium); 15797 (aloysiifolium); 16123 (fiebrigii); 16292 (fiebrigii); 16381 (aloysiifolium); 16611 (salicifolium); 16615 (salicifolium); 16633 (salicifolium); 16814 (aloysiifolium); 18274 (aloysiifolium); 18534 (weddellii); 18803 (salicifolium); 18833 (caesium); 18894 (aloysiifolium); 18953 (sinuatirecurvum); 19783 (weddellii); 20096 (aloysiifolium); 20793 (aloysiifolium); 20795 (cochabambense); 20803 (aloysiifolium); 20813 (aloysiifolium); 20980 (caesium); 21012 (fiebrigii); 21071 (glandulosipilosum); 21085 (cochabambense); 21226 (fiebrigii); 21489 (riojense); 21608 (aloysiifolium); 21732 (annuum); 21761 (tweedieanum); 21762 (tripartitum); 22366 (huayavillense); 22640 (aloysiifolium); 23384 (aloysiifolium); 23393 (palitans); 23663 (aloysiifolium); 23736 (salamancae); 23868 (aloysiifolium); 23993 (palitans); 24022 (caesium); 24106 (cochabambense); 24129 (aloysiifolium); 24216 (fiebrigii); 24665 (tweedieanum); 24728 (tweedieanum); 24791 (tweedieanum); 24912 (aloysiifolium); 25460 (aloysiifolium); 25576 (aloysiifolium); 25706 (caesium); 25730 (aloysiifolium); 26412 (aloysiifolium); 26483 (aloysiifolium); 27012 (echegarayi); 27046 (salicifolium); 27239 (tweedieanum); 27249 (tweedieanum); 27397 (sinuatirecurvum); 27441 (sinuatirecurvum); 27585 (aloysiifolium); 27827 (aloysiifolium); 27906 (aloysiifolium); 27912 (aloysiifolium); 27970 (aloysiifolium); 27988 (aloysiifolium); 28016 (aloysiifolium); 29559 (echegarayi); 29584 (nitidibaccatum); 29591 (tweedieanum); 29788 (fiebrigii); 29883 (caesium); 29896 (aloysiifolium); 30142 (echegarayi); 30191 (aloysiifolium); 30434 (physalifolium); 30546 (weddellii); 30709 (tripartitum); 30947 (caesium); 31070 (echegarayi); 31237 (echegarayi); 31302 (echegarayi); 31730 (sinuatirecurvum); 31825 (sinuatirecurvum); 32065 (fiebrigii); 32071 (zuloagae); 32095 (aloysiifolium); 32121 (huayavillense); 32140 (tripartitum); 32574 (tweedieanum); 33163 (triflorum); 33182 (triflorum); 33217 (triflorum); 33476 (triflorum); 33479 (triflorum); 33696 (woodii); 33776 (fiebrigii); 33861 (aloysiifolium); 33913 (aloysiifolium); 33915 (aloysiifolium); 34082 (aloysiifolium); 34121 (salicifolium); 34181 (nitidibaccatum); 34742 (tripartitum); 34840A (sinuatirecurvum); 34840 (sinuatirecurvum); 37741 (sinuatirecurvum).

Cabrera, V. 1 (chenopodioides); 2 (chenopodioides); 3 (chenopodioides); 4 (aloysiifolium); 5 (chenopodioides).

Cáceres, F. 597 (arequipense); 602 (arequipense); 619 (fragile); 621 (fragile); 760 (arequipense); 766 (radicans); 766a (arequipense); 858 (arequipense); 2892 (radicans); 2912 (arequipense); 5280 (arequipense).

Cadena G, A.L. 153 (nigrescens).

Calatayud, G. 999 (cochabambense); 1281 (cochabambense); 1626 (antisuyo); 1683 (antisuyo); 2391 (physalifolium); 2880 (longifilamentum); 2902 (longifilamentum); 3209 (antisuyo); 3647 (longifilamentum); 4062 (pseudoamericanum); 4350 (americanum).

Calcagnini, C. 100 (aloysiifolium); 537 (tweedieanum); 697 (caesium).

Callejas, R. 3144 (macrotonum); 7859 (macrotonum); 11325 (macrotonum).

Camargo, C. de 122 (americanum).

Camargo, E. 50 (americanum).

Camargo, O.R. 65 (americanum); 782 (chenopodioides); 962 (paucidens); 982 (chenopodioides); 1054 (paucidens); 1100 (paucidens); 1191 (paucidens); 1288 (chenopodioides); 1803 (paucidens); 2006 (paucidens); 2184 (americanum); 3274 (americanum).

Camp, W.H. E-2158 (macrotonum); E-2467 (interandinum); E-2915 (grandidentatum).

Campbell, D.G. P 22040 (americanum).

Campos Porto, P. 3241 (paucidens).

Campos, A.L. 20 (americanum).

Campos, J. 2097 (americanum); 2755 (americanum); 3796 (longifilamentum); 3908 (longifilamentum); 5379 (longifilamentum); 6472 (longifilamentum); 6474 (americanum); 6925 (americanum).

Cañigueral C, J. 80 (palitans).

Cano, A. 566 (pseudoamericanum); 1130 (grandidentatum); 2190 (americanum); 2265 (americanum); 2625 (radicans); 3636 (antisuyo); 4327 (antisuyo); 4593 (antisuyo); 4699 (pallidum); 4711 (pentlandii); 4735 (americanum); 5402 (longifilamentum); 5455 (longifilamentum); 5516 (longifilamentum); 5523 (pallidum); 5774 (radicans); 5776 (americanum); 5819 (radicans); 7223 (juninense); 7418 (juninense); 7915 (arequipense); 7988 (arequipense); 8040 (fragile); 8208 (grandidentatum); 8272 (fragile); 8320 (fragile); 8632 (pseudoamericanum); 8688 (americanum); 8777 (pseudoamericanum); 8862 (corymbosum); 10609 (interandinum); 11127 (interandinum); 11231 (interandinum); 11345 (corymbosum); 11589 (interandinum); 12052 (gonocladum); 12319 (interandinum); 12450 (interandinum); 12494 (interandinum); 12631 (americanum); 12857 (interandinum); 12886 (interandinum); 13104 (interandinum); 13909 (pseudoamericanum); 15380 (grandidentatum); 20615 (weddellii); 21036 (interandinum); 21073 (polytrichostylum); 21130 (cochabambense); 21151 (grandidentatum); 21283 (arequipense).

Cano, C. 741 (tweedieanum).

Cano, E. 79 (pygmaeum); 129 (pygmaeum); 365 (triflorum); 716 (triflorum); 741 (pilcomayense); 960 (marmoratum); 1724 (triflorum); 2580 (tweedieanum); 2831 (triflorum); 2832 (triflorum); 4017 (chenopodioides); 4019 (triflorum); 4060 (triflorum); 4061 (triflorum); 4520 (salicifolium); 4708 (triflorum); 4721 (tweedieanum).

Cano, M. 79 (interandinum).

Canqui M, F. 342 (cochabambense).

Cantero, J.J. 6289 (nitidibaccatum); 6355 (tweedieanum); 6434 (tweedieanum); 6865 (tweedieanum); 7046 (palitans); 7220 (tweedieanum).

Cantino, P.D. 447 (salicifolium); 448 (tweedieanum); 479 (tweedieanum); 729 (nitidibaccatum); 746 (nitidibaccatum); 762 (tweedieanum).

Capparelli, P.N. 257 (americanum).

Caranqui, J. 430 (antisuyo); 848 (antisuyo); 1244 (interandinum).

Carauta, J.P.P. 310 (americanum); 1236 (americanum); 3227 (americanum).

Cárdenas, M. 94 (tripartitum); 184 (fragile); 378 (gonocladum); 603 (gilioides); 644 (aloysiifolium); 733 (cochabambense); 749 (pallidum); 761 (antisuyo); 1175 (pallidum); 1179 (polytrichostylum); 2053 (americanum); 2109 (fiebrigii); 2154 (longifilamentum); 2264 (gonocladum); 2290 (woodii); 2331 (rhizomatum); 2377 (gonocladum); 2486 (physalifolium); 3043 (cochabambense); 3258 (gonocladum); 3263 (sinuatiexcisum); 3409 (cochabambense); 3470 (gonocladum); 3571 (fiebrigii); 3618 (leptocaulon); 3729 (tripartitum); 4356 (americanum); 4635 (caesium); 4636 (caesium); 4663 (alliariifolium); 5184 (aloysiifolium); 5447A (cochabambense); 5577 (cochabambense); 5620 (aloysiifolium); 5716 (alliariifolium); 5929 (aloysiifolium); 5942 (alliariifolium); 5968 (cochabambense); 6066 (cochabambense); 6087 (fiebrigii); 7417 (fiebrigii); 7418 (cochabambense); 7617 (gilioides).

Cárdenas, V. 12275 (corymbosum).

Cardona Naranjo, F.A. 238 (macrotonum).

Cardoso, L.J.T. 79 (americanum).

Cardozo, A. 974 (macrotonum).

Carenzo, V. 1306 (salamancae); 1318 (annuum); 1332 (physalifolium); 3636 (chenopodioides).

Carette, E. 72 (tweedieanum); 108 (tweedieanum); 114 (nitidibaccatum); 155 (tweedieanum); 197 (tweedieanum).

Carlier, I. 86 (grandidentatum).

Carolina, M. 17 (americanum).

Carpio, C. del 131 (arequipense).

Carriazo, E. 3 (interandinum).

Carrito, A.C. 8 (pilcomayense).

Carter, G.F. 82 (pilcomayense).

Carvalho, B. 16 (americanum); 26 (americanum).

Carvalho, D.M.G. 5 (americanum).

Carvalho, L. ASE 8047 (americanum).

Carvalho-Silva, M. 1487 (americanum); 1562 (americanum).

Carvalo, G.P. de 80 (americanum).

Casas, J. 6603 (antisuyo).

Castaño A, N. 1660 (americanum).

Castellanos, A. 8 (nitidibaccatum); 35 (triflorum); 253 (tweedieanum); 804 (chenopodioides); 811 (americanum); 814 (chenopodioides); 817 (chenopodioides); 818 (chenopodioides); 820 (pygmaeum); 821 (pilcomayense); 3108 (tweedieanum); 3223 (salicifolium); 6187 (triflorum); 11689 (tweedieanum); 16935 (chenopodioides); 17860 (chenopodioides).

Castillón, L. 1033 (salicifolium); 2596 (annuum); 13127[p.p.] (annuum).

Castro, R. 17690 (americanum).

Castroviejo, S. 10694 (interandinum).

Catalano, L. 15 (sinuatirecurvum).

Cavalcante de Lima, H. 528 (enantiophyllanthum).

Cavalcanti, A.C.S. 136 (americanum); 163 (americanum).

Cavalcanti, F. JPB 18718 (americanum).

Cavero B, M. 125 (polytrichostylum).

Cayola, L.E. 1435 (cochabambense); 3232 (antisuyo); 3233 (antisuyo); 3988 (antisuyo).

Cazalet, P.C.D. 5523 (antisuyo).

Cazzaniga, M.G. 3400 (chenopodioides).

Ceballos, A. 47 (weddellii); 133 (fragile); 2662 (aloysiifolium).

Censo, ? 12 (salicifolium).

Cerana, M.M. 135 (aloysiifolium); 327 (zuloagae); 328 (aloysiifolium); 331 (aloysiifolium); 332 (aloysiifolium); 358 (cochabambense); 392 (cochabambense); 417 (cochabambense); 503 (aloysiifolium); 532 (aloysiifolium); 533 (aloysiifolium); 534 (huayavillense); 558 (fiebrigii); 588 (aloysiifolium); 731 (aloysiifolium); 757 (aloysiifolium); 758 (aloysiifolium); 845 (americanum); 857 (pilcomayense); 927 (tripartitum); 1259 (salicifolium); 1824 (chenopodioides); 1936 (chenopodioides); 1984 (aloysiifolium).

Cerceau, E. 2396a (tweedieanum); 2416 (tweedieanum).

Cerón, C.E. 1764 (interandinum); 1764 (interandinum); 1838 (radicans); 6841 (interandinum); 6917 (interandinum); 6974 (interandinum); 7079 (interandinum); 7087 (interandinum); 11617 (interandinum); 12213 (interandinum); 12527 (interandinum); 13526 (interandinum); 14476 (interandinum); 14505 (radicans); 14629 (interandinum); 14651 (interandinum); 14661 (radicans); 14716 (interandinum); 14834 (interandinum); 14957 (interandinum); 15001 (nigrescens); 15133 (interandinum); 15198 (interandinum); 15252 (interandinum); 15277 (interandinum); 15323 (interandinum); 15377 (interandinum); 15513 (americanum); 15550 (interandinum); 15580 (longifilamentum); 15617 (interandinum); 15684 (radicans); 15748 (interandinum); 15769 (interandinum); 15905[b] (nigrescens); 15905[a] (macrotonum); 15909 (grandidentatum); 15909 (grandidentatum); 16051 (interandinum); 16076 (interandinum); 16295 (interandinum); 16328 (interandinum); 16351 (interandinum); 16398 (radicans); 16459 (interandinum); 16478 (interandinum); 16478 (interandinum); 17458 (interandinum); 17617 (macrotonum); 17636 (interandinum); 18038 (antisuyo); 18038 (macrotonum); 18813 (radicans); 18870 (pseudoamericanum); 18909 (americanum); 19626 (interandinum); 20545 (americanum); 29283 (americanum).

Cerrate [de Ferreyra], E. 259 (corymbosum); 264 (arequipense); 1218 (arequipense); 2302 (interandinum); 3886 (interandinum); 4255 (arequipense); 4257 (grandidentatum); 4398 (grandidentatum); 5064 (americanum); 6117 (interandinum); 6982 (fragile); 7105 (fragile); 7234 (interandinum); 7419 (interandinum); 7773 (corymbosum);7888 (interandinum); 9215 (fiebrigii); 9215 (fiebrigii).

Cervantes, E. 34 (aloysiifolium); 146 (cochabambense); 203 (aloysiifolium).

Céspedes, A. 78 (interandinum).

Céspedes, F.N. 1207 (pilcomayense).

Cevallos, D. hih-2 (aloysiifolium).

Chacra Experimental 26 (tweedieanum).

Chagas, F. 1099 (paucidens).

Chambi, P. 21 (longifilamentum).

Chardou, C.Z. 87 (americanum).

Charpin, A. 20139 (pilcomayense); 20579 (fiebrigii); 20659 (salicifolium); 20803 (sinuatirecurvum); 21516 (sarrachoides); 23128 (sinuatirecurvum); 23148 (cochabambense); 23168 (cochabambense); 23575 (sinuatirecurvum).

Chávez A, E. 46 (americanum).

Chávez, A. 15 (americanum).

Chávez, R. 3428 (sinuatiexcisum); 3501 (pallidum).

Chiapella, J. 1643 (furcatum); 1656 (furcatum); 1806 (nitidibaccatum); 1809 (triflorum); 1839 (triflorum); 1840 (nitidibaccatum); 1888 (triflorum); 2032 (salicifolium); 2167 (physalidicalyx); 2517 (salicifolium); 2524 (salicifolium); 2630 (echegarayi); 2814 (tripartitum); 2828 (tripartitum); 2877 (palitans); 2908 (tripartitum).

Chiar, P. 105 (chenopodioides).

Chiarini, F. 106 (americanum); 340 (chenopodioides); 448 (tweedieanum); 463 (salicifolium); 552 (tweedieanum); 568 (physalidicalyx); 639 (americanum); 650 (nitidibaccatum); 687 (tweedieanum); 705 (pygmaeum); 707 (salicifolium); 737 (pygmaeum); 762 (tweedieanum); 788 (salicifolium); 794 (salicifolium); 795 (nitidibaccatum); 805 (nitidibaccatum); 807 (salicifolium); 818 (salicifolium); 819 (salicifolium); 820 (nitidibaccatum); 891 (pilcomayense); 909 bis (pilcomayense); 910 (chenopodioides); 914 (pilcomayense); 920 (salicifolium); 942 (sinuatirecurvum); 951 (tripartitum); 959 (cochabambense); 971 (nitidibaccatum); 1012 (chenopodioides); 1052 (americanum); 1088 (pygmaeum); 1168 (riojense); 1227 (fiebrigii); 1229 (tripartitum); 1341 (pygmaeum).

Chiriboga Q, B. 27 (interandinum).

Christian, T. 6 (furcatum); 31 (furcatum).

Cialdella, A.M. 243 (tripartitum); 462 (sinuatirecurvum).

Cifuentes R, J.C. 2 (interandinum).

Claren, F. 11299 (sinuatirecurvum); 11347 (sinuatirecurvum); 11369 (sinuatirecurvum); 11393 (sinuatirecurvum); 11427 (sinuatirecurvum); 11526 (sinuatirecurvum); 11535 (weddellii); 11548 (tripartitum); 11677 (weddellii); 11706 (sinuatirecurvum); 11730 (tripartitum).

Clark, J.L. 9088 (interandinum); 9444 (antisuyo); 9534 (antisuyo); 10252 (interandinum).

Clarke, O.F. 10707 (tripartitum).

Claude-Joseph 595 (furcatum); 1165 (pentlandii).

Claussen, P. 180 (americanum); 16846 (americanum).

Cleef, A.M. 3927 (macrotonum).

Clemants, S.E. 2079 (interandinum).

Clements, R. 59 (interandinum).

Clos, E.C. 169 (chenopodioides); 228 (pygmaeum); 238 (chenopodioides); 439 (chenopodioides); 1136 (chenopodioides); 1139 (chenopodioides); 1813 (chenopodioides); 1843 (chenopodioides); 2871 (chenopodioides); 2878 (chenopodioides); 3357 (chenopodioides); 3491 (tweedieanum); 3840 (chenopodioides); 4152 (chenopodioides); 4170 (triflorum); 5336 (chenopodioides); 6570 (tripartitum).

Coca, M. 1 (grandidentatum).

Cocucci, A.A. 24 (echegarayi); 30 (salicifolium); 106 (salicifolium); 108 (salicifolium); 109 (salicifolium); 110 (salicifolium); 111 (salicifolium); 112 (salicifolium); 113 (salicifolium); 252 (aloysiifolium); 284 (cochabambense); 293 (tiinae); 298 (aloysiifolium); 399 (triflorum); 467 (triflorum); 480 (tweedieanum); 831 (triflorum); 886 (gonocladum); 888 (gonocladum); 890 (palitans); 890b (pentlandii); 900 (cochabambense); 914 (tripartitum); 975 (physalidicalyx); 981 (tweedieanum); 983 (salicifolium); 989 (tiinae); 993 (palitans); 994 (riojense); 1003 (cochabambense); 1004 (cochabambense); 1006 (tweedieanum); 2037 (cochabambense); 2039 (fiebrigii); 2040 (cochabambense); 2073 (palitans); 2266 (echegarayi); 2589 (nitidibaccatum); 2635 (cochabambense); 2636 (cochabambense); 2646 (tripartitum); 2647 (tripartitum); 2649 (tweedieanum); 2691 (sinuatirecurvum); 2903 (americanum); 3265 (tripartitum); 3293 (palitans); 3318 (palitans); 3348 (palitans); 3354 (cochabambense); 3357 (hunzikeri); 3365 (tripartitum); 3371 (tripartitum); 4637 (triflorum); 5040 (triflorum); 5124 (tweedieanum); 5307 (chenopodioides); 5772 (chenopodioides); 5798 (tweedieanum).

Cocucci, A.E. 13 (aloysiifolium); 90 (aloysiifolium); 91 (aloysiifolium); 327 (pygmaeum); 331 (pygmaeum).

Cocucci, A.M. 59 (echegarayi).

Coeli, R. 116 (americanum).

Coello Arguello, D. 3 (interandinum).

Cogollo, A. 451 (americanum); 601 (americanum).

Comber, H.F. 736 (furcatum).

Combita, S. 114 (macrotonum); 114[a] (macrotonum).

Commerson, P. 45 (chenopodioides); 46 (chenopodioides).

Conceição, A.A. 473 (americanum).

Conceição, S.F. 310 (americanum).

Conrad, J. 1998 (americanum); 2120 (americanum); 2487 (salicifolium); 2551 (americanum); 2580 (aloysiifolium); 2637 (aloysiifolium); 2653 (chenopodioides); 2716 (interandinum); 2744 (grandidentatum).

Conway, W.M. 43 (pallidum).

Cook, O.F. 128 (pentlandii); 297 (pentlandii).

Cordeiro, J. 1938 (americanum).

Córdoba, W.A. 422 (americanum).

Core, E.L. 1011 (macrotonum).

Cornejo, M. 849 (dianthum).

Cornell Tropical Botany Field Trip 61 (macrotonum); 71 (macrotonum).

Coro-Rojas 1440 (caesium); 1604 (physalifolium).

Correa Luna, H. 1817 (zuloagae); 9147 (zuloagae).

Correa, A. 172 (aloysiifolium).

Correa, M.N. 4313 (salamancae); 4484 (tripartitum); 4516 (tripartitum); 5041 (americanum); 5046 (americanum); 5125 (americanum); 5138 (chenopodioides); 6080 (fiebrigii); 7528 (aloysiifolium); 7716 (aloysiifolium); 8467 (americanum); 9735 (tweedieanum); 9748 (triflorum); 9768 (triflorum); 9815 (triflorum); 10174 (triflorum).

Correia, C. 367 (caatingae).

Cortés S, S.P. 533 (interandinum); 580 (interandinum).

Cosa, M.T. 36 (chenopodioides); 110 (chenopodioides); 111 (tweedieanum); 127 (chenopodioides); 148 (pygmaeum); 155 (tweedieanum); 164 (tweedieanum); 201 (chenopodioides); 202 (chenopodioides); 203 (chenopodioides); 214a (chenopodioides); 214b (chenopodioides); 214 (chenopodioides); 229 (chenopodioides); 247a (tweedieanum); 247b (tweedieanum); 255 (salicifolium); 346 (tweedieanum); 398 (americanum).

Costa Sacco, J. da 148 (chenopodioides); 163 (chenopodioides); 360 (americanum).

Costa, D. 242 (americanum).

Costa, R. 104 (chenopodioides).

Costa-Lima, J.L. 1406 (caatingae); 1605 (americanum); 1837 (americanum); 1862 (caatingae).

Costich, D.E. 841 (americanum).

Couto, D.R. 677 (americanum).

Covas, G. 663 (tweedieanum); 912 (echegarayi); 1235 (echegarayi); 3065 (tweedieanum); 3222 (echegarayi); 3309 (triflorum); 15059 (echegarayi); 15113 (tweedieanum); 15114 (tweedieanum); 18223 (tweedieanum).

Cowan, R.S. 38176 (americanum).

Cremers, G. 8084 (americanum).

Crespo, S. 1769 (triflorum).

Criollo, E. 16 (interandinum).

Cristóbal, C.L. 1076 (pilcomayense); 1546 (pilcomayense); 1547 (pilcomayense).

Croat, T.B. 7618 (nigrescens); 19668 (americanum); 20836 (americanum); 21488 (macrotonum); 21544 (nigrescens); 21777 (americanum); 21810 (nigrescens); 38415 (americanum); 38473 (nigrescens); 38843 (macrotonum); 51125 (americanum); 51453 (longifilamentum); 51503 (sinuatiexcisum); 51537 (americanum); 51705 (antisuyo); 54460 (nigrescens); 54826 (macrotonum); 55237 (nigrescens); 55251 (macrotonum); 57681 (cochabambense); 57771 (longifilamentum); 57786 (americanum); 57853 (cochabambense); 57854 (cochabambense); 58383 (americanum); 58409 (corymbosum); 60534 (macrotonum); 70552 (americanum); 72219 (macrotonum); 72852 (interandinum); 74460 (americanum); 74531 (americanum); 81568 (antisuyo); 81916 (americanum); 85407 (americanum); 86179 (interandinum); 86293 (macrotonum); 86355 (antisuyo); 86468 (macrotonum); 88133 (interandinum); 88968 (interandinum); 89114 (interandinum); 89211 (macrotonum); 89882 (macrotonum); 89999 (macrotonum); 90777 (macrotonum); 92009 (longifilamentum); 92283 (americanum); 92589 (interandinum); 92622 (longifilamentum); 93223 (macrotonum); 93228 (macrotonum); 95074 (interandinum); 96314 (antisuyo); 97419 (nigrescens); 98274 (nigrescens); 98275 (longifilamentum); 99171 (macrotonum); 99253 (interandinum); 99281 (macrotonum); 99634 (macrotonum).

Cruz A, S.P. 612 (nigrescens).

Cruz P, N. 16 (interandinum).

Cruz, J.S. de la 1664 (americanum); 4237 (americanum); 4442 (americanum); 4602 (americanum).

Cuatrecasas, J. 278 (macrotonum); 1703 (nigrescens); 5374 (macrotonum); 6002 (interandinum); 7897 (macrotonum); 11810 (macrotonum); 12017 (macrotonum); 12379 (macrotonum); 12491 (macrotonum); 14750 (macrotonum); 18456 (macrotonum); 18884 (macrotonum); 20235 (macrotonum); 20955 (interandinum); 22491 (nigrescens); 22978 (americanum); 24503 (interandinum); 25030 (interandinum); 27658 (macrotonum).

Cuello, N. 1376 (macrotonum).

Cueva, M. 26 (arequipense); 98 (sinuatiexcisum); 136 (sinuatiexcisum); 216 (longifilamentum); 276 (longifilamentum); 418 (longifilamentum); 444 (longifilamentum); 474 (longifilamentum); 482 (subtusviolaceum); 492 (antisuyo); 495 (juninense); 612 (longifilamentum); 625 (antisuyo); 626 (juninense).

Cuezzo, A.R. 1554 (tweedieanum); 1658 (salicifolium); 1851 (echegarayi); 1902 (salicifolium); 1952 (salicifolium); 2037 (echegarayi); 2566 (tweedieanum); 2586 (salicifolium); 5486 (glandulosipilosum).

Cuming, H. 164 (aloysiifolium); 266[b] (furcatum); 266[a] (furcatum); 783[b] (nitidibaccatum); 999 (americanum); 21263 (nitidibaccatum).

Cunha, L.S. 72 (americanum).

Curran, H.M. 188 (chenopodioides).

Cusato, L. 1140 (glandulosipilosum); 1904 (caesium); 4159 (furcatum).

Custódio Filho, A. 94 (americanum).

Cutler, H. 7697 (gonocladum).

Czermak, J. 381 (paucidens).

D'Arcy, W.G. 13716 (interandinum); 13758 (cochabambense); 13813 (pallidum); 13848 (subtusviolaceum); 13852 (pallidum); 13878 (dianthum); 13879 (dianthum); 13933 (longifilamentum); 13971 (radicans); 14041 (americanum); 14083 (interandinum); 14108 (antisuyo); 14825[a] (radicans); 14825[b] (interandinum); 14831 (interandinum); 14834 (macrotonum); 14838 (interandinum); 14841 (interandinum); 14919 (radicans); 14924 (interandinum); 15607 (nigrescens); 15623 (macrotonum); 15657 (nigrescens); 15667 (macrotonum); 15708 (longifilamentum); 15728 (antisuyo); 15734 (antisuyo); 15746 (interandinum); 15763 (interandinum); 15764 (interandinum); 16452A (longifilamentum); 16453 (longifilamentum); 16468 (macrotonum); 16476 (longifilamentum); 16498 (macrotonum); 16501A (macrotonum); 16507 (longifilamentum); 18243 (macrotonum).

D'Orbigny, A. 302 (tripartitum); 1208 (aloysiifolium); 1346 (tripartitum); 1536 (gonocladum); 1537 (tripartitum); 1541 (gonocladum).

Da Ros, ? 132 (nigrescens).

Daly, D.C. 17 (aloysiifolium); 37 (rhizomatum); 43 (aloysiifolium); 6634 (americanum); 9161 (americanum).

Damasceno Jr, G.A. 972 (pilcomayense).

Damasso, A.D. 747 (echegarayi).

Dantas, M.M. 18 (americanum).

Davidse, G. 4104 (americanum); 5089 (nigrescens); 5472 (americanum); 10549 (americanum); 11202 (americanum); 11819 (americanum); 13450 (nigrescens); 14511 (americanum); 18475 (nigrescens); 20926A (nigrescens); 28073 (nigrescens); 28147 (nigrescens).

Davidson, C. 3852 (dianthum); 4976 (longifilamentum); 10577 (americanum).

Dávila, M. 19 (nigrescens).

Davis, E.W. 557 (pallidum); 1348 (pentlandii); 1440 (cochabambense); 1756 (cochabambense); 1799 (polytrichostylum).

Davis, P.H. 2460 (chenopodioides).

Dawe, M.T. 289 (nigrescens).

Dawson, G. 1501 (salicifolium); 1555 (salicifolium); 1561 (salicifolium); 1947 (palitans); 2457 (nitidibaccatum); 2716 (nitidibaccatum).

DCI (Darwin Chilean Initiative 2002-2005) 1815 (furcatum).

de Mera, G. 15 10 (nitidibaccatum).

Deanna, R. 54 (americanum); 163 (americanum); 180 (salicifolium); 186 (aloysiifolium); 189 (salicifolium); 334 (americanum); 369 (physalidicalyx); 381 (salicifolium); 386 (sinuatirecurvum); 390 (tweedieanum); 442 (salicifolium); 443 (salicifolium).

Degen, R. 831 (americanum); 2203 (americanum); 2733 (pilcomayense); 3132 (sarrachoides).

Deginani, N.B. 279 (tripartitum); 499 (sinuatirecurvum); 1179 (americanum); 1281 (americanum); 1321 (americanum); 1825 (americanum); 2166 (nitidibaccatum); 2187 (tweedieanum); 2250 (triflorum); 2255 (echegarayi).

Del Aguila, M. 162 (americanum).

del Carpio, C. 2537 (arequipense).

Del Castillo, A. 677 (physalidicalyx); 1108 (tweedieanum).

Del Vitto, L.A. 819 (echegarayi); 821 (echegarayi); 851 (triflorum); 852 (salicifolium); 854 (tweedieanum); 2182 (huayavillense); 3103 (echegarayi); 3149 (physalidicalyx); 3411 (nitidibaccatum); 3455 (marmoratum); 3895 (tweedieanum); 4071 (nitidibaccatum); 4527 (chenopodioides); 4708 (echegarayi); 4994 (tweedieanum).

Delgado, L. 87 (interandinum); 177 (interandinum); 288 (interandinum); 331 (interandinum); 449 (interandinum); 508 (interandinum).

Delgado, L.B.S. 2773 (americanum).

Dell'Arciprete, A.C. 2337 (tripartitum).

Delnatte, C. 1544 (nigrescens).

Demaio, P. 294 (chenopodioides).

Dematteis, M. 728 (aloysiifolium); 2575 (aloysiifolium); 2694 (aloysiifolium).

Denham, S.S. 352 (chenopodioides).

Devoto, F.E. 36442 (aloysiifolium).

Di Fulvio, T.E. 29 (tweedieanum); 66 (tweedieanum); 386 (pygmaeum); 479 (pygmaeum); 483 (pygmaeum); 539 (salicifolium); 741 (sinuatirecurvum); 758 (chenopodioides); 782 (tiinae); 783 (tweedieanum); 795 (chenopodioides); 801 (americanum); 806 (pilcomayense); 849 (pygmaeum); 1058 (tripartitum); 1062 (gilioides); 1094 (tweedieanum); 1102 (chenopodioides).

Dias, R.S. 02 (americanum).

Díaz Gonzáles, J. 791 (americanum).

Díaz M, D. 1010 (nigrescens); 1057 (americanum).

Díaz P, S. 502 (macrotonum).

Díaz S, C. 2354 (americanum); 2701 (antisuyo).

Díaz, C. 2701 (polytrichostylum); 3539 (americanum); 9478 (americanum); 9503 (americanum); 9972 (longifilamentum); 10555 (longifilamentum).

Diáz, W. 5295 (nigrescens).

Diem, J. 3555 (triflorum).

Diers, L. 90 (palitans); 176 (aloysiifolium); 285 (tiinae); 311 (cochabambense).

Dik, A. 1310 (americanum).

Dillenburg, L.R. 134 (paucidens).

Dillon, M.O. 887 (cochabambense); 1115 (longifilamentum); 1115 (antisuyo); 3155 (interandinum); 3155 (interandinum); 3275 (fragile); 3275 (arequipense); 3307 (radicans); 3641 (americanum); 3643 (radicans); 4059 (interandinum); 4499 (interandinum); 4539 (arequipense); 6503 (arequipense).

Dimitri, M.J. 65 (tweedieanum); 202 (aloysiifolium); 364 (tweedieanum).

Dinelli, E. 593 (physalidicalyx).

Dittrich, V.A.O. 159 (chenopodioides).

Döbbeler, P. 4068 (interandinum).

Döbereiner, J. 756 (americanum).

Dodson, C.H. 1817A (macrotonum); 11523 (americanum); 12067 (macrotonum).

Dombey, J. 63 (corymbosum); 343 (arequipense).

Dombrowski, L.T. 143 (americanum); 221 (paucidens); 237 (americanum); 310 (americanum); 477 (americanum); 1228 (paucidens); 3564 (americanum); 3689 (americanum); 4153 (americanum); 4798 (americanum); 7776 (americanum); 9767 (americanum).

Domingo, M.A. 42 (arequipense); 57 (nitidibaccatum).

Donadío, S. 134 (echegarayi); 219 (aloysiifolium).

Donat, A. 55 (triflorum); 55a (triflorum); 415 (nitidibaccatum).

Dorr, L.J. 5400 (nigrescens); 6539 (antisuyo); 6727 (fiebrigii); 6731 (polytrichostylum); 6952 (cochabambense); 8414 (macrotonum); 8971 (macrotonum).

Dottori, N.M. 42 (chenopodioides); 43 (chenopodioides); 44 (chenopodioides); 153 (chenopodioides); 155 (chenopodioides); 173 (chenopodioides); 200 (echegarayi); 214 (chenopodioides); 220 (chenopodioides); 222 (chenopodioides).

Dreveck, S. 498 (americanum).

Drouet, F.E. 2412 (americanum).

Dryander, E. 2769 (nigrescens).

Duarte, M. 3334 (paucidens).

Dubugnon, N. 187 (chenopodioides).

Dudley, T.R. 10312 (arenicola).

Dueñas, R. 100 (interandinum).

Dugand, A. 1103 (americanum); 4537 (nigrescens); 5024 (americanum); 7080 (nigrescens).

Dunaski, A. 89 (americanum).

Duno de Stefano, R. 1203 (macrotonum).

Duque Jaramillo, J.M. 2476 (americanum); 2699 (interandinum); 2833 (interandinum); 2834 (interandinum); 3649 (nigrescens); 3665 (americanum); 4047A (americanum); 4100 (nigrescens).

Duré, R. 208 (americanum).

Dusén, P. 386 (furcatum); 663 (enantiophyllanthum); 3567 (paucidens); 5465 (triflorum); 6278 (pygmaeum); 9380 (paucidens); 9512 (americanum); 10201 (americanum); 11800 (paucidens); 15747 (paucidens); 17296 (paucidens).

Edmonds, J.M. T 360 (echegarayi).

Edwin, G. 3799 (grandidentatum).

Egea, J. de 12 (pilcomayense); 807 (americanum); 844 (pilcomayense); 935 (pilcomayense); 1320 (paucidens).

Ehrich, R. 6 (tweedieanum); 39 (tripartitum); 133 (cochabambense); 156 (gilioides); 254 (cochabambense); 385 (hunzikeri).

Eisinger, S. 37 (americanum); 38 (americanum); 40 (americanum).

Eiten, G. 1596 (americanum); 1895 (americanum); 4753 (americanum); 6184 (americanum); 6239 (americanum); 6670 (enantiophyllanthum); 6673 (enantiophyllanthum); 7211 (americanum); 7876 (americanum); 10152 (americanum).

Ekman, E.L. 813 (paucidens).

Elías, H. 219 (americanum); 853 (nigrescens).

Elias, S.I. 178 (americanum).

Eliasson, U. 100 (interandinum).

Ellenberg, H. 77 (nitidibaccatum); 120 (radicans); 200 (grandidentatum); 202 (radicans); 403 (cochabambense); 2692 (arequipense); 2977 (weddellii); 3251 (interandinum); 3759 (interandinum); 4004 (juninense); 4281 (sinuatirecurvum); 4810 (cochabambense); 7016 (cochabambense); 7124 (fragile); 8706 (corymbosum).

Elliot, C. 160 (echegarayi).

Emperaire, L. 577 (caatingae).

Emshwiller, E. EE-312 (subtusviolaceum); EE-334 (pallidum); EE-383 (fragile).

Emygdio, L. 3401 (paucidens).

Erickson, H.T. 13 (americanum); 29 (enantiophyllanthum); 37 (pilcomayense).

Escobar A, N.; H. 10 (palitans).

Escobar, L.A. 6459 (macrotonum).

Espinal T, S. 2961 (interandinum).

Espínola, M.C. JPB-666 (americanum).

Espinosa, P. 27 (interandinum).

Espinosa, R. E-799 (interandinum); 809 (americanum); 2903 (interandinum).

Espinoza, R. 39 (radicans).

Espinoza, Y. 33 (americanum).

Essi, L. 303 (americanum).

Esteban, E. 50 (tweedieanum).

Estela D, M.A. 45 (interandinum).

Estelrich 7 (triflorum).

Estrada, J. 149 (nigrescens).

Estudiantes Espoch 789 (radicans).

Eugenio, J. 1089 (americanum).

Eupunino, E. 489 (americanum).

Evrard, C. 10603 (furcatum).

Ewan, J.A. 15691 (americanum).

Eyerdam, W.J. 10741 (grandidentatum); 22151 (radicans); 22152 (arequipense); 22304 (aloysiifolium); 22389 (aloysiifolium); 22469 (sinuatiexcisum); 22476 (huayavillense); 22596 (caesium); 22617 (fiebrigii); 23162 (chenopodioides); 23555 (triflorum); 23682 (chenopodioides); 23905 (triflorum); 23954 (triflorum); 23956 (triflorum); 24449 (triflorum); 24671 (cochabambense); 24741 (gonocladum); 24944 (cochabambense); 24969 (cochabambense); 25029 (aloysiifolium).

Eynden, V. van den 463 (americanum); 701 (americanum); 980 (americanum).

Fabris, H.A. 1777 (sinuatirecurvum); 2294 (echegarayi); 2939 (fiebrigii); 3058 (aloysiifolium); 3088 (aloysiifolium); 3175 (nitidibaccatum); 3570 (aloysiifolium); 3928 (cochabambense); 4075 (weddellii); 4092 (gilioides); 4119 (palitans); 5153 (glandulosipilosum); 5224 (aloysiifolium); 5398 (gilioides); 5927 (fiebrigii); 6017 (tripartitum); 6477 (palitans); 6932 (gilioides); 7453 (tripartitum); 7681 (aloysiifolium); 7847 (aloysiifolium); 7848 (fiebrigii); 7947 (salicifolium); 8225 (fiebrigii).

Fagerlind, F. 517 (americanum); 607 (americanum); 696 (americanum); 770 (macrotonum); 1039 (interandinum); 1436 (macrotonum); 1536 bis (interandinum); 2022 (interandinum).

Falcão, M. 133 (americanum).

Falconí, G. 79 (interandinum).

Fallen, M.E. 710 (interandinum).

Farfán, J. 346 (cochabambense); 1196 (americanum); 1721 (americanum); 1771 (pallidum).

Farruggia, F. 2637 (americanum); 2646 (americanum); 2713 (americanum); 2720 (longifilamentum); 2737 (americanum); 2753 (longifilamentum); 2756 (cochabambense); 2780 (cochabambense); 2781 (americanum); 2801 (juninense); 2819 (interandinum).

Felisbino, ? 25 (americanum).

Fendler, A. 1021 (nigrescens).

Feres, F. 0796 (americanum).

Fernandes, D. 235 (chenopodioides); 291 (americanum).

Fernandes, I. 839 (chenopodioides).

Fernández Alonso, J.L. 5845A (americanum); 6290 (interandinum); 6409 (interandinum); 6498 (americanum); 6746 (americanum); 6772 (americanum); 6911 (americanum); 6934 (nigrescens); 6957 (nigrescens); 7111 (interandinum); 8289 (interandinum); 13247 (nigrescens); 15973 (americanum); 16625 (interandinum); 16864 (americanum); 19069 (interandinum); 19093 (interandinum); 19978 (macrotonum); 20782 (nigrescens); 20790 (americanum); 21674 (americanum); 21802 (nigrescens); 23186 (interandinum); 23259 (interandinum); 24755 (nigrescens); 24816 (americanum); 25172 (interandinum).

Fernández T, E. 3245 (leptocaulon); 4212 (aloysiifolium).

Fernández, Á. 25275 (macrotonum).

Fernández, E. EF-2038 (dianthum); EF-2241 (dianthum); EF-2242 (dianthum); EF-2243 (dianthum); 4018 (cochabambense); 4055 (cochabambense).

Fernández, P. 49 (chenopodioides); 62 (salicifolium); 108 (chenopodioides); 128 (chenopodioides).

Fernández-Casas, J. 7290 (americanum); 7389 (americanum); 7570 (paucidens); 7741 (cochabambense); 8015 (fragile); 8591 (arenicola).

Ferreira, A.P. 236 (americanum).

Ferreira, P.P.A. 544 (chenopodioides).

Ferreira, V.F. 3045 (americanum); 4086 (pilcomayense).

Ferreyra, R. 452 (cochabambense); 533 (radicans); 716 (corymbosum); 1021 (longifilamentum); 1486 (arequipense); 1990 (pseudoamericanum); 2634 (cochabambense); 3238 (cochabambense); 3672 (cochabambense); 4055 (aloysiifolium); 4402 (americanum); 5389 (interandinum); 6058 (polytrichostylum); 6074 (arequipense); 6081a (polytrichostylum); 6081b (arequipense); 6506 (interandinum); 6607 (juninense); 8164 (antisuyo); 8255 (arequipense); 8952 (arequipense); 9718 (arequipense); 9781 (grandidentatum); 9798 (cochabambense); 11622 (radicans); 11625 (americanum); 12038 (arequipense); 12065 (arequipense); 12836 (americanum); 12858 (aloysiifolium); 13498 (radicans); 13760 (corymbosum); 15675 (juninense); 16127 (cochabambense); 16616 (pallidum); 16721 (longifilamentum); 16756 (cochabambense); 17231 (americanum); 17683 (americanum); 18050 (grandidentatum); 18319 (arequipense); 18361 (arequipense); 18361 (arequipense); 18866 (americanum); 19959 (americanum).

Ferrucci, M.S. 1720 (caesium); 1758 (aloysiifolium); 1964 (dianthum).

Feuerer, T. 5492 (palitans); 6841a (pallidum); 6897a (cochabambense); 7024a (polytrichostylum); 7792a (grandidentatum); 7933a (grandidentatum); 7958a (cochabambense); 8364a (antisuyo); 8367a (cochabambense); 8509a (longifilamentum); 8786a (cochabambense); 9107a (cochabambense); 9358a (cochabambense); 22078a (polytrichostylum); 22877 (fragile); 23343 (dianthum).

Fiebrig, K. 2131 (aloysiifolium); 2421 (fiebrigii); 2439 (cochabambense); 2471 (sinuatirecurvum); 2507 (huayavillense); 3428 (aloysiifolium).

Fierro, A.F. 644 (radicans).

Figueroa C, Y. 541 (interandinum); 632 (interandinum); 667 (interandinum).

Figueroa Romero, M.R. 556 (americanum); 841 (tweedieanum); 1100 (physalidicalyx).

Figueroa, S. 3 (aloysiifolium); 4 (aloysiifolium).

Filippa, E.M. 37 (americanum); 56 (americanum); 86 (salicifolium).

Filskov, P. 37152 (interandinum); 37440 (radicans).

Firmin, G. 3 (interandinum); 611 (macrotonum).

Fischer, W. 100 (triflorum); 215 (triflorum).

Fisher, P.B. 28 (pallidum).

Flite, V. 32369 (enantiophyllanthum).

Flores, S. 2 (radicans).

Flores, S.P. 8 (americanum); 61 (americanum); 133 (americanum).

Flores, T.B. 231 (paucidens); 1319 (paucidens).

Flossdorf, A. 55 (tiinae); 56 (tiinae).

Floyer [Mrs E.A.] 8 (pygmaeum).

FLSP 480 (arequipense); 759 (americanum); 760 (radicans); 913 (arequipense); 949 (americanum); 1116 (americanum); 1149 (arequipense); 1240 (arequipense); 1291 (arequipense); 1325 (americanum); 1415 (americanum); 1460 (arequipense); 1464 (radicans).

Fogaça, J. 02 (americanum).

Fonnegra, R. 2452 (americanum); 2670 (macrotonum); 2722 (americanum); 5148 (nigrescens); 5193 (nigrescens); 6167 (macrotonum).

Fonseca B, M.H. 12 (macrotonum).

Fonseca Vaz, A. 303 (paucidens).

Fontana, A.P. 811 (americanum); 4837 (americanum); 5764 (americanum).

Forero P, L.E. 426 (nigrescens); 428 (americanum); 466 (americanum); 1620 (americanum).

Forero, E. 15 (macrotonum); 1974 (americanum).

Fortunato, R.H. 2017 (pilcomayense); 2356 (pilcomayense); 2612 (pilcomayense); 3213 (pilcomayense); 4685 (pilcomayense); 4688 (pygmaeum); 5275 (triflorum); 5652 (echegarayi); 6041 (americanum); 6063 (americanum); 7480 (triflorum); 7601 (riojense); 7606 (salicifolium); 7617 (tripartitum); 7749 (aloysiifolium); 10003 (triflorum).

Forzza, R.C. 4309 (americanum).

Fosberg, F.R. 20503 (americanum); 20559 (macrotonum); 20565 (interandinum); 22404 (interandinum); 22440 (grandidentatum); 27528 (macrotonum); 27930 (americanum); 27961[a] (corymbosum); 27961[b] (americanum); 28236 (americanum); 28399 (radicans); 29282 (americanum); 44774 (americanum); 44785 (americanum); 44788 (americanum); 44823 (americanum); 57408 (americanum).

Foster, R.B. 3021 (arenicola); 9756 (americanum); 10334 (americanum); 11365 (arenicola).

Fournet, A. 419 (fiebrigii); 532 (arenicola); 539 (arenicola); 557 (cochabambense).

França, F. 5548 (americanum).

Francia, P. 4 (americanum).

Franquemont, C. 297 (cochabambense); 348 (cochabambense).

Freeman, J.D. 15 (americanum).

Freire de Carvalho, L d'A 148 (americanum); 153 (americanum); 570 (americanum).

Freitas, E. 285 (americanum).

Freitas, L. 4 (paucidens); 5 (chenopodioides); 617 (enantiophyllanthum).

Frey, R. 495 (pilcomayense); 572 (pilcomayense).

Fries, R.E. 761 (sinuatirecurvum); 779 (tripartitum); 890 (grandidentatum); 996 (aloysiifolium); 1030 (gilioides); 1147 (tripartitum); 1162 (cochabambense); 1384 (physalidicalyx).

Fróes, R.L. 11663 (americanum).

Fuentes, A.F. 2607 (michaelis); 8489 (antisuyo); 8519 (pallidum); 9989 (antisuyo); 10457 (antisuyo); 12764 (cochabambense); 12777 (polytrichostylum); 13261 (longifilamentum); 13913 (dianthum); 13921 (dianthum); 14388 (antisuyo); 15548 (longifilamentum); 16026 (grandidentatum); 16141 (longifilamentum).

Funez, L.A. 129 (americanum); 542 (paucidens); 2819 (chenopodioides); 2891 (paucidens).

Funk, V.A. 3410 (pallidum).

Gadelha Neto, P.C. 736 (americanum).

Galander, C. 18 (salicifolium).

Galeano, G. 2156 (macrotonum); 2176B (macrotonum); 7602 (macrotonum).

Galeano, M.P. 239 (macrotonum); 495 (macrotonum); 2270 (macrotonum).

Galiano, W. 4162 (cochabambense); 5172 (physalifolium); 6003 (cochabambense).

Galindo T, R. 1237 (macrotonum).

Gallegos, S. 488 (aloysiifolium).

Gallinal H, J.P. 721 (chenopodioides); 3710 (chenopodioides).

Gamarra, P. 416 (pseudoamericanum); 502[a] (gonocladum); 662 (cochabambense).

Garcia A, S. 149 (interandinum).

García Llatas, L. 8155 (pseudoamericanum).

Garcia, A.L. 1013 (pilcomayense).

García, E. 47 (gonocladum); 124 (polytrichostylum); 152 (pentlandii); 194 (pentlandii).

García, E.M. 360 (salicifolium).

Garcia, I. 70 (palitans).

Garcia, M.A. 12681 (chenopodioides); 22891 (chenopodioides).

García, P. 210 (americanum); 253 (pilcomayense); 679 (tweedieanum); 932 (pilcomayense).

García, R.C.A. 541 (palitans).

Garcia, R.J.F. 464 (americanum).

García-Barriga, H. 17 C 751 (macrotonum); 6308 (americanum); 7784 (macrotonum); 11700 (macrotonum); 12321 (nigrescens); 12684 (macrotonum); 12875 (macrotonum); 12931 (macrotonum); 13091 (interandinum); 13251 (interandinum); 18415 (americanum); 20736 (macrotonum); 20977 (macrotonum).

Gardner, G. 838 (americanum); 1788 (americanum); 2266 (americanum).

Gardner, M.F. 63 (radicans); 82 (furcatum); 94 (furcatum); 174 (furcatum); 4034 (furcatum); 6206 (radicans); 6252 (gonocladum); 6516 (grandidentatum); 6738 (furcatum); 6963 (furcatum); 8322 (furcatum); 8356 (furcatum); 8462 (furcatum); 8478 (furcatum); 8649 (furcatum).

Gasper, A.L. de 3194 (americanum).

Gaudichaud, C. 112 (furcatum); 520 (chenopodioides); 521 (americanum); 522 (americanum); 1847 (furcatum).

Gautier, E.D. 16 (aloysiifolium).

Gavilanes, M. 17 (interandinum); 136 (interandinum); 798 (antisuyo).

Gay, C. 2 (furcatum); 297 (radicans); 1199 (radicans); 2000 (arequipense).

Ge, X. 242 (americanum).

Gehriger, W. 15 (interandinum); 260 (interandinum).

Gentry, A.H. 9040 (nigrescens); 9375 (americanum); 9479 (interandinum); 12371 (interandinum); 15129 (macrotonum); 16416 (americanum); 16464 (americanum); 17066 (macrotonum); 17098 (interandinum); 17944 (americanum); 19198 (interandinum); 19333 (interandinum); 19419 (cochabambense); 25381 (americanum); 27256 (americanum); 27384 (americanum); 28535 (americanum); 30379 (macrotonum); 30667 (interandinum); 35737 (arequipense); 36111 (arequipense); 37391 (interandinum); 39745 (interandinum); 41286 (macrotonum); 43245 (radicans); 44135 (pseudoamericanum); 47738 (nigrescens); 54148 (nigrescens); 60329 (macrotonum); 61585 (interandinum); 63561 (americanum); 63701 (americanum); 64370 (radicans).

Gérique, A. 390 (longifilamentum).

Gérold, R. 3 (tripartitum).

Giacomin, L.L. 9 (sinuatirecurvum); 15 (americanum); 236 (americanum); 366 (enantiophyllanthum); 873 (paucidens); 938 (enantiophyllanthum); 952 (enantiophyllanthum); 1074 (paucidens); 1128 (enantiophyllanthum); 1227 (paucidens); 1504 (americanum); 1646 (americanum); 1750 (dianthum); 1764 (polytrichostylum); 1766 (pallidum); 1802 (interandinum); 1971 (americanum); 1974 (americanum); 2009 (americanum); 2013 (americanum); 2021 (americanum); 2036 (enantiophyllanthum); 2039 (enantiophyllanthum); 2103 (americanum).

Giardelli, M.L. 52 (pygmaeum); 326 (pygmaeum); 326[a] (tweedieanum); 1056 (pygmaeum); 1165 (nitidibaccatum).

Gibert, E.J. 55 (tweedieanum); 147 (sarrachoides).

Giberti, G.C. 879 (tripartitum).

Gil, M. 21 (americanum).

Gil, R.R. 503 (aloysiifolium).

Gillett, J.M. 16487 (americanum).

Gilli, A. 97 (interandinum); 222 (americanum); 373 (macrotonum).

Gillies, J. 3 (nitidibaccatum); 7 (tweedieanum); 10 (salicifolium); 11(41) (tweedieanum); 18 (triflorum); 20 (salicifolium); 21 (tweedieanum); 32 (salicifolium); 33 (pygmaeum); 35 (triflorum); 36 (echegarayi); 38 (chenopodioides); 40 (tweedieanum); 43 (triflorum); 44 (triflorum); 151 (chenopodioides); 600 (tweedieanum); 625 (tweedieanum); 1431x (pygmaeum); 1434 (triflorum); 1436 (palitans).

Gilmartin, A.J. 581 (americanum).

Gines, H. 1572 (interandinum); 2072 (americanum).

Giordano, L.C. 748 (americanum); 1737 (paucidens).

Giordano, O. 1 (tweedieanum); 2 (nitidibaccatum); 3 (nitidibaccatum); 8 (triflorum); 10 (triflorum); 22 (nitidibaccatum); 23 (marmoratum).

Giorgis, M. 76 (salicifolium).

Giorgis, M.A. 55 (tweedieanum); 62 (chenopodioides); 1359 (tweedieanum).

Giraldo, G. 979 (americanum).

Girault, L. 27 (cochabambense).

Glaumann, F. 105 (americanum).

Glaziou, A.F.M. 8867 (enantiophyllanthum).

Glenn, A. 370 (juninense); 474 (juninense).

Glocker, E.F. von 85 (americanum).

Godoy, S.A.P. de 2024 (americanum); 2033 (americanum); 2047 (americanum); 2063 (americanum).

Goës, C.C. 596 (americanum).

Góes, O.C. 97 (americanum).

Gomes, E. 279 (americanum).

Gomes, J.M.L. 150 (americanum).

Gomes, L.A. 495 (americanum).

Gomez, A. 5 (macrotonum); 75 (macrotonum).

Gómez, J. 51 (corymbosum).

Gómez, J.C. 5 (macrotonum).

Gómez, P. 9 (interandinum).

Goncalves, A.C. PSACF_EX 4342 (americanum).

Gonto, R. 3288 (interandinum); 3739 (macrotonum).

Gonzales J, V. 26 (radicans).

Gonzáles, P. 6 (corymbosum); 17 (arequipense); 234 (pseudoamericanum); 313 (longifilamentum); 343 (subtusviolaceum); 348 (antisuyo); 349 (antisuyo); 494 (arequipense); 531 (arequipense); 663A (pentlandii); 663B (arequipense); 960 (pseudoamericanum); 1017 (pseudoamericanum); 1426 (grandidentatum); 1560 (grandidentatum); 1561 (gonocladum); 1577 (pseudoamericanum); 1665 (fragile); 1667 (gonocladum); 1668 (interandinum); 1669 (grandidentatum); 1738 (americanum); 1776 (pseudoamericanum); 1818 (corymbosum); 1819 (pseudoamericanum); 1847 (pseudoamericanum); 1853 (arequipense); 1877 (fragile); 1991 (polytrichostylum); 1992 (arequipense); 2016 (interandinum); 2088 (interandinum); 2092 (pseudoamericanum); 2104 (interandinum); 2108 (longifilamentum); 2112 (interandinum); 2139 (interandinum); 2193 (interandinum); 2332 (cochabambense); 2333 (interandinum); 2380 (cochabambense); 2851 (pseudoamericanum); 2852 (grandidentatum); 2853 (arequipense); 2858 (pseudoamericanum); 2859 (arequipense); 2860 (corymbosum); 2867 (pseudoamericanum); 2870 (arequipense); 2874 (arequipense); 2875 (arequipense); 2876 (pseudoamericanum); 2877 (radicans); 2894 (interandinum); 2895 (interandinum); 2900 (pseudoamericanum); 2906 (pseudoamericanum); 2928 (arequipense); 2929 (corymbosum); 2930 (pseudoamericanum); 2937 (corymbosum); 2946 (interandinum); 2948 (interandinum); 2952 (interandinum); 2962 (grandidentatum); 2964 (americanum); 2972 (americanum).

Gonzales, R. 518 (macrotonum).

González, C.E. 3466 (nigrescens).

González, F. 1656 (nigrescens).

González, H. 17 (macrotonum).

Goodspeed, T.H. 4612 (echegarayi).

Gouvêa, Y.F. 222 (enantiophyllanthum).

Graf, K. 520 (cochabambense).

Graham, J.G. 231 (americanum); 4146 (americanum).

Granados-Tochoy, J.C. 756 (interandinum);892 (americanum).

Grau, A. 4 (aloysiifolium).

Graywood Smyth, E. 19 (americanum).

Grifo, F.T. 1032 (albescens).

Grisales, A. 9 (nigrescens).

Groenendijk, J.P. 1302 (interandinum).

Grondona, E.M. 3014 (americanum); 3058 (americanum); 6373 (pygmaeum).

Grubb, P.J. 562 (macrotonum).

Grupo Pedra do Cavalo 80 (americanum); 771 (caatingae).

Guaglianone, E.R. 485 (pilcomayense); 1443 (nitidibaccatum); 1647 (echegarayi); 3115 (sinuatirecurvum).

Gualteros, F. 3 (nigrescens).

Guánchez M, F.J. 2122 (americanum).

Guedes, R. 2537 (paucidens).

Güerere, I. 42 (nigrescens).

Guerreiro, E. 15 (salicifolium);16 (aloysiifolium).

Guerrero, W. 336 (americanum).

Guevara, R. 6 (salicifolium).

Guillén, R. 53 (americanum).

Guimarães, E.A. 1 (americanum).

Guimarães, J.G. 14 (americanum).

Guinena, A. 42 (americanum).

Günther, E. 5840 (pallidum); 5843 (pallidum); 5844 (pallidum); 5845 (pallidum).

Gurini, L. 60 (pilcomayense); 301 (chenopodioides).

Gutiérrez de Sanguinetti, M.M. 378 (aloysiifolium).

Gutiérrez R, J. 506 (aloysiifolium); 1135 (aloysiifolium).

Gutiérrez V, G. StaRsa-1 (macrotonum); Sibundoy-3 (macrotonum); 430 (nigrescens); 17C-669 (chenopodioides probs nigrescens).

Gutiérrez, J. 14 (chenopodioides); 155 (palitans); 506 (cochabambense).

Gutiérrez, R. SRB-43 (nigrescens); SRB-98 (nigrescens).

Gutte, P. 16 (palitans); 8149 (americanum); 8619 (americanum); 9536a (arequipense).

Haene, E. 359 (tweedieanum).

Haenke, T. 2105 (echegarayi).

Hage, J.L. 485 (americanum); 927 (americanum); 989 (americanum).

Hagelund, K. 132 (americanum); 1024 (americanum); 1469 (americanum); 4548 (americanum); 4693 (paucidens); 5110 (americanum); 5452 (americanum); 7361 (americanum); 7649 (americanum); 7690 (americanum); 7790 (americanum); 7820 (americanum); 8237 (americanum); 9494 (americanum); 10565 (americanum); 10749 (americanum); 10885 (americanum); 10894 (americanum); 11737 (americanum); 14520 (americanum); 15291 (americanum); 15996 (americanum).

Hahn, W.J. 766 (americanum); 2056 (americanum); 2470 (americanum); 4818 (americanum); 5010 (nigrescens); 5021 (macrotonum).

Hamilton, A.C. 42 (juninense); 1431 (cochabambense); 1432 (cochabambense).

Hammel, B. 5895 (furcatum); 5970 (sinuatirecurvum); 5981 (sinuatirecurvum).

Hammen, T. van der 916 (nigrescens).

Hanbury-Tracy, J. 432 (interandinum); 454 (nigrescens).

Harley, R.M. 17271 (americanum); 19125 (caatingae); 22191 (americanum); 22926 (americanum); 27292 (americanum).

Harling, G. 209 (americanum); 310 (americanum); 372 (americanum); 618 (interandinum); 747 (interandinum); 5761 (macrotonum); 6766 (grandidentatum); 6882 (grandidentatum); 8355 (interandinum); 9142 (americanum); 9537 (interandinum); 10276 (interandinum); 13120 (macrotonum); 21596 (americanum); 22061 (macrotonum); 22194 (americanum); 22478 (longifilamentum); 22644 (longifilamentum); 25021 (americanum); 26240 (americanum).

Harling, G.W. 7933 (interandinum); 12049 (interandinum).

Harrison, C.G. 502 (americanum).

Hart, J.A. 1283 (americanum).

Hassler, É. 91 (pilcomayense); 474 (americanum); 622 (americanum); 2324 (pilcomayense); 2393 (pilcomayense); 2524 (pilcomayense); 3104 (americanum); 5278 (paucidens); 6400 (americanum); 10271 (chenopodioides); 12197 (americanum).

Hastings, G.T. 47 (furcatum); 337 (furcatum).

Hatschbach, G.G. 9814 (paucidens); 10036 (paucidens); 30325 (paucidens); 33604 (paucidens); 35846 (enantiophyllanthum); 41445 (chenopodioides); 45226 (pilcomayense); 63010 (americanum); 67490 (paucidens); 68816 (paucidens); 71691 (sarrachoides); 72692 (americanum).

Hattori, E.K.O. 918 (enantiophyllanthum); 1119 (paucidens).

Haught, O. 138 (americanum); 5002 (macrotonum); 5692 (macrotonum); 5729 (interandinum); 5771 (interandinum); 6529 (interandinum); 6530 (interandinum); 6590 (americanum).

Hauthal, R. 31 (pygmaeum);58 (sinuatirecurvum); 77 (sinuatirecurvum); 269 (fragile).

Hawkes, J.G. 79 (interandinum); 80 (macrotonum); 106 (macrotonum); 3343 (echegarayi); 6403 (cochabambense); 6406 (gonocladum); 6507 (fiebrigii); 6579 (cochabambense).

Hayward, K. 2060 (aloysiifolium).

Heiser, C.B. S 128 (americanum); 4854 (macrotonum); 4863 (grandidentatum); 4910 (grandidentatum); 4920 (interandinum); 4922 (interandinum); 4926 (grandidentatum); 4941 (interandinum); 4962a (interandinum); 4973 (interandinum); 4978 (interandinum); 4990 (interandinum); 4993 (interandinum); 5001 (macrotonum); 5002 (grandidentatum); 5015 (interandinum); 5024 (macrotonum); 5033 (americanum); 5052 (macrotonum); 5055 (nigrescens); 5062 (macrotonum); 5064 (interandinum); 5068 (radicans); 5081 (interandinum); 5083 (macrotonum); 5084 (interandinum); 5085 (interandinum); 5093 (interandinum); 6017 (interandinum); 6020 (interandinum); 6021 (interandinum); 6022A (macrotonum); 6022B (macrotonum); 6025 (macrotonum); 6035 (grandidentatum); 6036 (grandidentatum); 6037 (interandinum); 6042 (macrotonum); 6043 (interandinum); 6051 (interandinum); 6061 (interandinum); 6062 (grandidentatum); 6068 (interandinum); 6070 (interandinum); 6081 (interandinum); 6098 (americanum); 6146 (interandinum); 6188 (interandinum); 6197 (interandinum); 6809 (radicans); 7074 (interandinum); 8311 (radicans).

Henning, T. 6 (cochabambense).

Henschen, S.E. 970 (americanum).

Herb. Kew K-225 (nigrescens).

Herb. Lamarck 8366 (radicans).

Herb. Monteiro 3855 (radicans).

Herb. Willdenow 2910 (radicans); 4336 (chenopodioides).

Heringer, E.P. 723 (americanum); 3403 (americanum); 4928 (americanum); 15204 (americanum).

Hermann, M. 417 (chenopodioides); 758 (cochabambense); 759 (cochabambense); 880 (tripartitum).

Hernandes-Bicudo, L.R. 1405 (chenopodioides).

Hernández P, L.A. 186 (nigrescens).

Hernández S, M. 108 (macrotonum); 707 (macrotonum); 746 (interandinum).

Hernández, A. 35 (nigrescens).

Hernández, C. 63 (americanum); 153 (americanum); 240 (americanum).

Hernández, J. 91 (macrotonum).

Hernández-Ramos, J.F. 30 (nigrescens).

Hernani A, L. 968 (antisuyo); 1033 (antisuyo); 1037 (antisuyo); 1207 (arequipense).

Herrera, A. 438 (americanum).

Herrera, E. 864 (macrotonum).

Herrera, F.L. 819 (cochabambense); 2178 (gonocladum); 2674 (polytrichostylum); 3022 (polytrichostylum); 3092 (cochabambense); 3236 (polytrichostylum); 3427 (pentlandii).

Herter, W.G.F. 186 (chenopodioides); 17039 (sarrachoides); 17060 (chenopodioides); 31076 (chenopodioides);70323 (chenopodioides).

Herzog, T. 806 (leptocaulon).

HHV FLSP 759 (americanum); 001474 (cochabambense); 001519 (antisuyo); FLSP 1723a (americanum); 3050 (antisuyo); 03570 (americanum); 04175 (corymbosum).

Hicken, C.M. 71 (triflorum); 107 (furcatum); 238 (tweedieanum); 266 (nitidibaccatum); 267 (nitidibaccatum); 272 (furcatum); 372 (pygmaeum); 448 (pygmaeum); 509 (pygmaeum); 727 (triflorum); 943 (pygmaeum); 13704 (pilcomayense); 14575 (pilcomayense).

Hicken, R. 38 (pygmaeum).

Hieronymus, G. 6 (tweedieanum); 50 (salicifolium); 53[a] (tweedieanum); 53b (aloysiifolium); 55 (salicifolium); 56 (aloysiifolium); 62 (aloysiifolium); 90 (salicifolium); 220 (salicifolium); 233 (riojense); 248 (tweedieanum); 416 (aloysiifolium); 429 (salicifolium); 441 (tweedieanum); 472 (riojense); 473 (salicifolium); 622 (salicifolium); 632 (tweedieanum); 638 (tweedieanum); 642 (salicifolium); 710 (triflorum); 710 (triflorum); 722 (tripartitum); 812 (salicifolium); 813 (salicifolium).

Hilgert, N. 1691 (aloysiifolium); 2165 (palitans); 2251 (aloysiifolium); 2519 (aloysiifolium).

Hitchcock, A.S. 19974 (americanum); 20357 (americanum); 21006 (interandinum).

Hjerting, J.P. 621 (radicans); 625 (juninense); 626 (pentlandii); 645 (americanum); 1060 (radicans); 6235 (interandinum).

Hoehne, W. SPF-12502 (americanum); JPB-17122 (americanum); JPB-17127 (americanum).

Hoenicka, H. 55 (nigrescens); 56 (nigrescens).

Hoff, M. 6245 (americanum).

Hoffmann, W. 305 (radicans).

Hoffmann, W.A. 245 (americanum).

Hollermayer, A. 42 (furcatum).

Holliday, L.G. 9 (americanum).

Holm-Nielsen, L.B. 2027 (americanum); 2048 (americanum); 2735 (americanum); 3462 (macrotonum); 3547 (macrotonum); 4956 (interandinum); 5212 (interandinum); 5831 (macrotonum); 6206 (macrotonum); 6281 (interandinum); 6670 (interandinum); 6773 (macrotonum); 6798 (macrotonum); 6813 (macrotonum); 16661 (radicans); 18084 (interandinum); 18830 (americanum); 24728 (grandidentatum); 26412 (macrotonum); 27946 (americanum); 27985 (americanum); 28899 (americanum); 28998 (interandinum); 29039 (interandinum); 29053 (interandinum); 29597 (interandinum); 29624 (interandinum).

Holmberg, E.L. 10469 (tweedieanum).

Holmgren, I. 33 (americanum); 480 (macrotonum); 727 (interandinum).

Holt, E.G. 177 (nigrescens).

Holt, P. 143 (radicans).

Holway, E.W.D. 63 (furcatum); 557 (pallidum).

Hondelmann, W. 71 (sinuatirecurvum).

Hoogte, L. van der 3444 (pallidum); 3836 (interandinum).

Hoover, W.S. 1583 (macrotonum).

Hosseus, C.C. 6 (chenopodioides); 15 (chenopodioides); 32 (tweedieanum); 39 (pilcomayense); 62 (tweedieanum); 147 (nitidibaccatum); 249 (pygmaeum); 262 (chenopodioides); 284 (chenopodioides); 291 (tweedieanum); 316 (pygmaeum); 330 (pilcomayense); 503 (echegarayi); 616 (salicifolium); 627 (chenopodioides); 673 (tweedieanum); 743 (chenopodioides); 795 (tweedieanum); 930 (tweedieanum); 1009 (salicifolium); 1009 bis (salicifolium); 1023 (physalidicalyx); 1958 (salicifolium); 2064 (tweedieanum); 2467 (triflorum); 2567 (salicifolium).

Hostmann, F.W.R. 992 (americanum).

Hoyos, D. 22 (interandinum).

Huamantupa, I. 2258 (antisuyo); 3185 (antisuyo); 3527 (cochabambense); 3617 (cochabambense); 3670 (antisuyo); 4280 (pseudoamericanum); 4287 (pseudoamericanum); 6608 (cochabambense); 7063 (cochabambense); 7095 (antisuyo); 7536 (pentlandii); 8323 (cochabambense); 9258 (longifilamentum).

Huapalla, J. 001407 (corymbosum); 002496 (corymbosum); 2550 (corymbosum); 2921 (radicans); 2935 (corymbosum); 03259 (corymbosum); 04298 (radicans).

Huashikat, V. 52 (americanum); 206 (americanum).

Huaylla, H. 649 (fiebrigii); 670 (aloysiifolium); 1004[a] (aloysiifolium); 1033 (tripartitum); 1338 (longifilamentum); 2069 (physalifolium).

Hudson, J. 964 (americanum); 1102 (radicans).

Hueck, J. 504 (sinuatirecurvum).

Huertas P, G. 5918 (macrotonum).

Huertas, G. 871 (interandinum).

Huidobro, A.M.R. 1332 (pygmaeum); 1410 (triflorum); 1410 (triflorum); 1449 (chenopodioides); 1614 (pygmaeum); 3133 (pilcomayense); 3267 (pilcomayense); 3470 (tweedieanum); 3524 (tweedieanum); 3555 (tweedieanum); 3569 (tweedieanum); 3652 (tweedieanum); 4198 (americanum).

Humbert, H. 26180 (macrotonum); 26268 (nigrescens).

Humbles, J. 6059 (radicans).

Hunziker, A.T. 92 (nitidibaccatum); 229 (chenopodioides); 285 (chenopodioides); 289 (chenopodioides); 339 (chenopodioides); 588 (pygmaeum); 614 (sarrachoides); 646 (chenopodioides); 832 (pilcomayense); 923 (pilcomayense); 1182 (fiebrigii); 1190 (palitans); 1193 (aloysiifolium); 1228 (aloysiifolium); 1279 (palitans); 1290 (tripartitum); 1388 (aloysiifolium); 1631 (physalidicalyx); 1875 (palitans); 1926 (aloysiifolium); 1932 (aloysiifolium); 2011 (aloysiifolium); 2141 (pygmaeum); 2216 (chenopodioides); 2234 (chenopodioides); 2242 (pygmaeum); 2270 (chenopodioides); 2286 (chenopodioides); 2315 (chenopodioides); 2623 (tiinae); 2957 (pilcomayense); 2965 (pilcomayense); 3156 (chenopodioides); 3301 (chenopodioides); 3440 (chenopodioides); 3441 (chenopodioides); 3449 (chenopodioides); 3574 (tweedieanum); 3589 (chenopodioides); 3665 (pilcomayense); 3705 (pilcomayense); 3712 (pilcomayense); 3876 (chenopodioides); 4420 (pygmaeum); 4468 (triflorum); 4522 (chenopodioides); 4748 (aloysiifolium); 4854 (tweedieanum); 5193 (salicifolium); 5217 (salicifolium); 5294 (aloysiifolium); 5302 (tweedieanum); 5548 (americanum); 5729 (pilcomayense); 6237 (pygmaeum); 6282 (chenopodioides); 6533 (pilcomayense); 6702 (pilcomayense); 6703 (pilcomayense); 6808 (cochabambense); 6809 (aloysiifolium); 6872 (chenopodioides); 6942 (salicifolium); 7025 (chenopodioides); 7140 (aloysiifolium); 7183 (pygmaeum); 7216 (tweedieanum); 7374 (chenopodioides); 7503 (pygmaeum); 7541 (pygmaeum); 7549 (pygmaeum); 7554 (tweedieanum); 7559 (salicifolium); 7665 (tweedieanum); 7666 (chenopodioides); 7783 (tweedieanum); 7860 (chenopodioides); 7861 (tweedieanum); 7863 (salicifolium); 7903 (salicifolium); 7926 (pygmaeum); 7963 (tweedieanum); 8455 (salicifolium); 8477 (salicifolium); 8543 (salicifolium); 8692 (pygmaeum); 8829 (nitidibaccatum); 8880 (echegarayi); 9243 (physalidicalyx); 9741 (tweedieanum); 9764 (chenopodioides); 9781 (triflorum); 9787 (echegarayi); 9836 (salicifolium); 9949 (chenopodioides); 9952 (tweedieanum); 10093 (fiebrigii); 10359 (pygmaeum); 10445 (pygmaeum); 10488 (tweedieanum); 10557 (echegarayi); 10765 (tweedieanum); 10832 (tweedieanum); 10956 (chenopodioides); 10959 (chenopodioides); 10974 (chenopodioides); 10997 (physalidicalyx); 11288 (pygmaeum); 11309 (chenopodioides); 11415 (chenopodioides); 11474 (tweedieanum); 11629 (triflorum); 11638 (salicifolium); 11667 (salicifolium); 11693 (chenopodioides); 11697 (tweedieanum); 11712 (tweedieanum); 11798 (salicifolium); 11868 (nitidibaccatum); 12213 (chenopodioides); 12244 (salicifolium); 12353 (chenopodioides); 12544 (triflorum); 12602 (salicifolium); 12654 (tweedieanum); 13037 (triflorum); 13039 (tweedieanum); 13044 (triflorum); 13045 (tweedieanum); 13088 (tweedieanum); 13233 (chenopodioides); 13237 (chenopodioides); 13250 (pygmaeum); 13434 (salicifolium); 13661 (americanum); 13668 (pseudoamericanum); 13669 (corymbosum); 13673 (americanum); 13678 (arequipense); 13681 (radicans); 13692 (pseudoamericanum); 13719 (aloysiifolium); 13798 (chenopodioides); 13826 (salicifolium); 13970 (tweedieanum); 13991 (nitidibaccatum); 14025 (tweedieanum); 14078 (nitidibaccatum); 14114 (nitidibaccatum); 14126 (nitidibaccatum); 14266 (echegarayi); 14270 (echegarayi); 14398 (triflorum); 14430 (tweedieanum); 14545 (nitidibaccatum); 14619 (tweedieanum); 14685 (salicifolium); 14936 (physalidicalyx); 14949 (tweedieanum); 14980 (tweedieanum); 15028 (triflorum); 15171 (tweedieanum); 15172 (marmoratum); 15200 (tweedieanum); 15204 (tweedieanum); 15206 (marmoratum); 15230 (tweedieanum); 15286 (tweedieanum); 15317 (aloysiifolium); 15449 (americanum); 15643 (aloysiifolium); 15661 (aloysiifolium); 15702 (palitans); 15722 (tweedieanum); 16077 (tweedieanum); 16357 (tweedieanum); 16705 (tweedieanum); 16956 (tweedieanum); 17004 (tweedieanum); 17242 (tweedieanum); 17540 (aloysiifolium); 17583 (aloysiifolium); 17829 (pilcomayense); 18022 (salicifolium); 18361 (palitans); 18411 (aloysiifolium); 18543 (palitans); 18693 (chenopodioides); 18905 (aloysiifolium); 18927 (chenopodioides); 19073 (hunzikeri); 19165 (aloysiifolium); 19411 (tweedieanum); 19552 (americanum); 19869 (aloysiifolium); 20141 (triflorum); 20319 (hunzikeri); 20329 (hunzikeri); 20358 (aloysiifolium); 20421 (aloysiifolium); 20472 (aloysiifolium); 20476 (salicifolium); 20477 (salicifolium); 20514 (salicifolium); 20533 (salicifolium); 20658 (aloysiifolium); 21113 (aloysiifolium); 21928 (palitans); 21967 (pygmaeum); 22205 (hunzikeri); 22206 (hunzikeri); 22787 (tweedieanum); 23521 (americanum); 23669 (salicifolium); 23759 (salicifolium); 23796 (echegarayi); 23806 (salicifolium); 23865 (salicifolium); 24049 (salamancae); 24188 (salicifolium); 24228 (sarrachoides); 24587 (nitidibaccatum); 24645 (chenopodioides); 24691 (aloysiifolium); 24693 (aloysiifolium); 24711 (aloysiifolium); 24743 (tripartitum); 24745 (tripartitum); 24749 (sinuatirecurvum); 24785 (sarrachoides); 24805 (triflorum); 24807 (triflorum); 24809 (triflorum); 24813 (echegarayi); 24826 (aloysiifolium); 24833 (aloysiifolium); 24843 (palitans); 24844 (salicifolium); 24872 (cochabambense); 24876 (salicifolium); 24878 (tiinae); 24879 (tiinae); 24882 (salicifolium); 24883 (tweedieanum); 24886 (annuum); 24901 (annuum); 24941 (tweedieanum); 24952 (pygmaeum); 25036 (tweedieanum); 25043 (salicifolium); 25075 (salicifolium); 25313 (aloysiifolium); 25321 (salicifolium); 25322 (nitidibaccatum); 25330 (pygmaeum); 25331 (pygmaeum); 25333 (tweedieanum); 25335 (marmoratum); 25494 (palitans); 25546 (tiinae); 25625 (echegarayi).

Hunziker, J.H. 25 (pilcomayense); 35 (chenopodioides); 84 (pygmaeum); 115 (pilcomayense); 121 (pilcomayense); 175 (chenopodioides); 228 (pilcomayense); 401 (triflorum); 506 (nitidibaccatum); 525 (nitidibaccatum); 556 (nitidibaccatum); 561 (chenopodioides); 614 (tweedieanum); 694 (pilcomayense); 832 (americanum); 1022 (chenopodioides); 1193 (chenopodioides); 1624 (chenopodioides); 1888 (salicifolium); 1963 (salicifolium); 2087 (salicifolium); 2478 (chenopodioides); 3314 (pygmaeum); 3816 (chenopodioides); 3847 (americanum); 3904 (aloysiifolium); 4038 (echegarayi); 4342 (salicifolium); 4558 (americanum); 4712 (echegarayi); 7254 (americanum); 7509 (pygmaeum); 10374 (caesium); 10484 (sinuatirecurvum); 11022 (americanum); 11284 (echegarayi); 11682 (physalidicalyx); 12028 (chenopodioides); 12298 (tripartitum); 12305 (tripartitum); 12438 (tweedieanum); 12706 (aloysiifolium); 12840 (aloysiifolium).

Hunziker, J.J. 10515 (weddellii); 11638 (salicifolium).

Hurrell, J. 6431 (pilcomayense); 6432 (pilcomayense).

Hurrell, J.A. 6037 (chenopodioides); 6439 (chenopodioides).

Hutchison, P.C. 3458 (americanum); 3603 (corymbosum); 3853 (americanum); 5469 (cochabambense); 5931 (antisuyo); 7171 (radicans).

Huttel, C. 599 (americanum); 882 (radicans).

Ibáñez, M. 33 (arequipense).

Ibarrola, T.S. 2847 (paucidens); 3219 (paucidens).

Ibisch, P.L. 347 (tripartitum).

Idrobo, J.M. 3230 (macrotonum).

Ihue U, Y. 2004 25 (arequipense); 2004 75 (arequipense).

Illin, N. 273 (nitidibaccatum).

Iltis, H.H. P 2 (interandinum); 42 (interandinum); 217 (cochabambense); 218 (cochabambense); 324 (cochabambense); 355 (americanum); 388 (americanum); E-409 (interandinum); 457a (pallidum); 464 (cochabambense); 481a (fragile); 483 (fragile); 578 (cochabambense); 593 (pentlandii); 594 (radicans); 646 (cochabambense); 744 (americanum); 774 (cochabambense); 779 (cochabambense); 866 (grandidentatum); 867 (pentlandii); 869 (pentlandii); 954 (polytrichostylum); 955 (pentlandii); 984 (pentlandii); 986 (cochabambense); 1035 (pentlandii); 1037 (cochabambense); 1038 (cochabambense); 1135 (pentlandii); 1136 (pentlandii); 1138 (cochabambense); 1157 (grandidentatum); 1175 (grandidentatum); 1179 (cochabambense); 1189b (cochabambense); 1189a (physalifolium); 1190 (cochabambense); 1191 (cochabambense); 1343 (fragile); 1517 (radicans); 1554 (americanum); 1593 (americanum); 1621 (interandinum).

Imaguire, N. 105 (americanum); 317 (americanum).

Infantes Vera, J.G. 5242 (americanum); 5402 (grandidentatum).

Inostroza, ? CONC-35154 (furcatum).

Irwin, H.S. 230 (nigrescens); 2069 (americanum); 5865 (americanum); 9151 (chenopodioides); 9186 (americanum); 10990 (americanum); 13755 (americanum); 17893 (americanum); 18095 (americanum); 18123 (americanum); 19170 (americanum); 19812a (americanum); 20328 (americanum); 20958 (americanum); 23142 (americanum); 23766 (americanum); 24177 (americanum); 28500 (americanum); 29614 (americanum); 30413a (americanum); 34909 (americanum); 55947 (americanum).

Isern, J. 2060 (radicans); 2535 (arequipense); 6302 (americanum).

Isler, ? 64 (tweedieanum).

Iter Patagonicum 8 (nitidibaccatum); 733 (triflorum).

Izabella 200 (americanum).

Jacintho, S.F. 2 (americanum).

Jacobson, V. 18 (furcatum).

Jácome, J. 877 (dianthum).

Jacques, E.L. 65 (enantiophyllanthum).

Jahn, A.J. 588 (interandinum); 1179 (interandinum).

Jaimes, M.S. 10 (americanum).

Jameson, W. 262 (interandinum); 266 (interandinum).

Jangoux, J. 166 (americanum).

Jansen-Jacobs, M.J. 5503 (americanum).

Jara-Muñoz, A. 287 (americanum); 483 (americanum).

Jaramillo, J. 3 (interandinum); 396 (interandinum); 1151 (interandinum); 2319 (radicans); 2321 (interandinum); 7061 (grandidentatum); 8183 (macrotonum); 8237 (nigrescens); 8786 (macrotonum); 8926 (longifilamentum); 9647 (interandinum); 10614 (antisuyo); 13151 (americanum); 15214 (americanum); 15258 (longifilamentum); 18539 (longifilamentum); 20925 (interandinum); 20940 (americanum); 21139 (interandinum); 26699 (macrotonum); 26870 (americanum); 27983 (interandinum).

Jardim, A. 289 (leptocaulon).

Jarenkow, J.A. 324 (chenopodioides).

Játiva, C.D. 153 (americanum).

Jeffrey, C. 2326 (americanum); 2521 (nigrescens).

Jelski, C. de 46 (interandinum); 49 (americanum).

Jenman, G.S. 4510 (americanum); 4573 (americanum); 5131 (americanum).

Jiménez E, N.D. 315 (americanum).

Jiménez, B. 107 BJ (chenopodioides); 1339 (americanum).

Jiménez, M. 603 (alliariifolium); 616 (fiebrigii); 620 (aloysiifolium); 703 (aloysiifolium); 815 (aloysiifolium).

Job, M.M. 728 (pilcomayense).

Johns, T. 82-16 (fragile); 83-43 (fragile); 83-48 (sinuatirecurvum); 82-70 (weddellii); 82-59 (tripartitum); 82-60 (cochabambense); 82-61 (aloysiifolium).

Johnson, A.E. 843 (paucidens).

Johnston, I.M. 6129 (echegarayi).

Joly, A.B. JPB-17125 (americanum).

Jönsson, G. 841a (paucidens); 893a (americanum).

Jordán, C.G. 187 (leptocaulon); 195 (leptocaulon); 249 (dianthum); 352 (dianthum); 523 (dianthum); 526 (cochabambense).

Jörgensen, P. 91 (americanum); 114 (americanum); 529 (triflorum); 555 (nitidibaccatum); 657 (caesium); 978 (annuum); 1064 (tweedieanum); 1309 (aloysiifolium); 1473 (cochabambense); 1474 (aloysiifolium); 1635 (aloysiifolium); 1636 (echegarayi); 1638 (weddellii); 2214 (pilcomayense); 2621 (pilcomayense); 3340 (sarrachoides); 10709 (weddellii).

Jørgensen, P.M. 56161 (interandinum); 56275 (americanum); 56403 (macrotonum); 61338 (interandinum); 61460 (interandinum); 61466 (macrotonum).

Jouvin, P.P. 179 (americanum); 533 (americanum).

Juárez, F. 240 (aloysiifolium); 352 (aloysiifolium); 1652 (profusum); 1718 (aloysiifolium); 1776 (tweedieanum); 1840 (aloysiifolium); 1901 (huayavillense); 2021 (fiebrigii).

Julio [Brother] II-31 (gonocladum); 128 (polytrichostylum); II 240 (cochabambense).

Juncosa, A. 751 (americanum); 2220 (americanum).

Junge, C. 1271 (furcatum); 2611 (furcatum); 2861 (nitidibaccatum); 2862 (furcatum); 3083 (furcatum); 3087 (furcatum).

Junk, W.J. 245 (americanum).

Juzepczuk, S.V. 10049 (pentlandii); 10051 (radicans); 10642 (juninense); 11030 (pallidum); 11031 (tripartitum); 11032 (polytrichostylum); 11033 (radicans); 11050 (gonocladum); 11075 (tripartitum).

Jørgensen, P.M. 56275 (americanum).

Kahatt Soto, N. 014 (interandinum); 156 (americanum).

Kahn, F. 2980 (americanum); 2991 (juninense).

Kalenborn, M. 26 (pentlandii).

Kappler, A. 1663 (nigrescens).

Kausel, E.M.L. 3236 (furcatum); 3260 (furcatum); 4554 (furcatum); 4655 (furcatum).

Kayap, R. 1182 (americanum); 1436 (americanum).

Kegler, A. 1511 (americanum).

Keller, H.A. 1363 (paucidens); 4227 (americanum); 7196 (tweedieanum); 7211 (sarrachoides); 8556 (chenopodioides); 8606 (chenopodioides); 8940 (tweedieanum); 9040 (tweedieanum); 9041 (tweedieanum); 10153 (tweedieanum); 10176 (chenopodioides); 10253 (tweedieanum); 11103 (aloysiifolium); 11135 (fiebrigii).

Kessler, M. 18 (americanum); 179 (polytrichostylum); 252 (aloysiifolium).

Kiesling, R. 228 (tripartitum); 1116 (aloysiifolium); 1169 (tripartitum); 1619 (palitans); 1633 (fiebrigii); 2971 (echegarayi); 3096 (salicifolium); 3406 (aloysiifolium); 3434 (tripartitum); 3514 (weddellii); 3734 (hunzikeri); 3813 (cochabambense); 3845 (hunzikeri); 3861 (tripartitum); 3866 (gilioides); 3873 (weddellii); 4110 (salicifolium); 4112 (salicifolium); 4199 (salicifolium); 4237 (echegarayi); 4537 (echegarayi); 5201 (tripartitum); 5203 (tripartitum); 5252 (sinuatirecurvum); 5275 (grandidentatum); 5376 (aloysiifolium); 6094 (echegarayi); 6500 (triflorum); 6560 (echegarayi); 6562 (triflorum); 6642 (salicifolium); 6678 (salicifolium); 6717 (salicifolium); 7064 (tripartitum); 7425 (echegarayi); 7751 (echegarayi); 7824 (echegarayi); 7855 (echegarayi); 7929 (salicifolium); 8249 (huayavillense); 8259 (huayavillense); 8354 (michaelis); 8960 (tweedieanum); 10398 (echegarayi); 10537 (americanum).

Killip, E.P. 2001 (macrotonum); 9744 (nigrescens); 14060 (americanum); 15569 (macrotonum); 15692 (interandinum); 16681 (macrotonum); 16688 (interandinum); 16879 (interandinum); 16948 (interandinum); 16957 (interandinum); 16970 (macrotonum); 17011 (nigrescens); 17192 (interandinum); 17408 (interandinum); 17915 (interandinum); 17996 (interandinum); 18090 (macrotonum); 18132 (macrotonum); 18490 (nigrescens); 18731 (macrotonum); 18896 (macrotonum); 18956 (macrotonum); 19920 (macrotonum); 20011 (macrotonum); 20723 (macrotonum); 20795 (nigrescens); 20911 (nigrescens); 21540 (fragile); 21630 (grandidentatum); 21752 (pentlandii); 21791 (interandinum); 21862 (interandinum); 21871 (grandidentatum); 21912 (cochabambense); 22243 (cochabambense); 22970 (americanum); 23116 (longifilamentum); 23318 (cochabambense); 23360 (juninense); 23424 (americanum); 24058 (americanum); 24181 (cochabambense); 24430 (arequipense); 25218 (americanum); 28827 (americanum).

King, D.O. 24 (triflorum); 149B (echegarayi); 149A (tweedieanum); 150 (tweedieanum); 151 (salicifolium); 266 (chenopodioides); 285 (pygmaeum); 301 (pygmaeum); 401B (furcatum); 401A (nitidibaccatum); 462 (pygmaeum); 535 (chenopodioides); 537 (triflorum); 538 (pygmaeum); 630 (pygmaeum); 651 (echegarayi); 720 (pygmaeum).

King, R.M. 9105 (cochabambense); 9178 (cochabambense).

Kinoshita, L.S. 33 (americanum).

Kirizawa, M. 1048 (americanum).

Kirkbride Jr, J.H. 1853 (macrotonum); 2143 (americanum); 2314 (macrotonum); 2564 (nigrescens); 2684 (americanum).

Klein, R.M. 657 (americanum).

Klein, V.L.G. 673 (paucidens); 700 (americanum); 1192 (americanum).

Klevens, M.J. 17 C 318 (nigrescens).

Klitgaard, B.B. 1405 (americanum).

Knapp, G. 31 (interandinum).

Knapp, S. 6360 (cochabambense); 6467 (radicans); 7626a (americanum); 7627 (americanum); 7738 (americanum); 7802 (americanum); 9052 (macrotonum); 9054 (americanum); 9067 (interandinum); 9092 (longifilamentum); IM-10079 (chenopodioides); IM-10161 (americanum); 10205 (americanum); 10206 (americanum); 10210 (americanum); 10214 (grandidentatum); 10217 (arequipense); 10219 (grandidentatum); 10220 (interandinum); 10223 (interandinum); 10231 (fragile); 10233 (fragile); 10234 (interandinum); 10237 (interandinum); 10239 (interandinum); 10241 (interandinum); 10245 (interandinum); 10247 (polytrichostylum); 10248 (pentlandii); 10252 (pentlandii); 10253 (interandinum); 10254 (pentlandii); 10258 (pentlandii); 10259 (fragile); 10263 (interandinum); 10265 (pentlandii); 10266 (polytrichostylum); 10267 (pentlandii); 10271 (pentlandii); 10273 (pentlandii); 10274 (polytrichostylum); 10277 (radicans); 10279 (polytrichostylum); 10284 (cochabambense); 10287 (cochabambense); 10289 (polytrichostylum); 10291 (pentlandii); 10293 (pseudoamericanum); 10294 (americanum); 10297 (polytrichostylum); 10300 (pseudoamericanum); 10302 (americanum); 10304 (radicans); 10306 (radicans); 10307 (pseudoamericanum); 10308 (pentlandii); 10318 (interandinum); 10319 (pentlandii); 10320 (polytrichostylum); IM-10321 (polytrichostylum); 10324 (grandidentatum); 10325 (cochabambense); 10327 (cochabambense); 10332 (physalifolium); 10334 (physalifolium); 10335 (cochabambense); 10337 (cochabambense); 10339 (cochabambense); 10340 (cochabambense); 10341 (cochabambense); 10343 (cochabambense); 10344 (pentlandii); 10348 (radicans); 10349 (polytrichostylum); 10351 (pseudoamericanum); 10352 (pentlandii); 10357 (pseudoamericanum); 10360 (americanum); 10362 (cochabambense); 10363 (cochabambense); 10365 (physalifolium); 10368 (polytrichostylum); 10373 (pentlandii); 10374 (radicans); 10376 (pentlandii); 10378 (grandidentatum); 10380 (cochabambense); 10383 (polytrichostylum); 10384 (polytrichostylum); 10391 (cochabambense); 10392 (cochabambense); 10393 (cochabambense); 10399 (antisuyo); 10400 (cochabambense); 10401 (antisuyo); 10404 (antisuyo); 10405 (polytrichostylum); 10406 (antisuyo); IM-10408 (polytrichostylum); 10412 (cochabambense); 10413 (grandidentatum); 10414 (polytrichostylum); 10416 (pentlandii); 10417 (radicans); 10419 (polytrichostylum); 10423 (pallidum); 10424 (pallidum); 10425 (pallidum); 10427 (pallidum); 10430 (pallidum); 10431 (pallidum); 10433 (pallidum); 10434 (pallidum); 10435 (antisuyo); 10437 (polytrichostylum); 10438 (pentlandii); 10439 (polytrichostylum); 10440 (cochabambense); 10442 (pallidum); 10443 (pallidum); 10444 (pallidum); 10445 (pallidum); 10446 (pallidum); 10450 (pallidum); 10453 (sinuatiexcisum); 10454 (longifilamentum); 10457 (pallidum); 10461 (polytrichostylum); 10462 (pallidum); 10464 (cochabambense); 10485 (echegarayi); 10488 (triflorum); 10489 (tweedieanum); 10490 (tweedieanum); 10492 (salicifolium); 10504 (triflorum); 10513 (triflorum); 10540 (echegarayi); 10566 (grandidentatum); 10567 (interandinum); 10568 (radicans); 10575 (pseudoamericanum); 10578 (polytrichostylum); 10579 (americanum); 10580 (cochabambense); 10585 (interandinum); 10590 (pseudoamericanum); 10591 (grandidentatum); 10599 (pseudoamericanum); 10603 (corymbosum); 10604 (pseudoamericanum); 10606 (polytrichostylum); 10607 (interandinum); 10608 (americanum); 10614 (polytrichostylum); 10616 (pseudoamericanum); 10624 (polytrichostylum); 10628 (interandinum); 10629 (interandinum); 10634 (corymbosum); 10636 (grandidentatum); 10637 (corymbosum); 10640 (grandidentatum); 10648 (americanum); 10650 (pseudoamericanum); 10654 (grandidentatum); 10656 (grandidentatum); 10658 (cochabambense); 10659 (grandidentatum); 10669 (cochabambense); 10715 (interandinum); 10721 (cochabambense); 10739 (longifilamentum); 10740 (interandinum); 10754 (americanum).

Knight, D.H. 386 (longifilamentum); 756 (americanum); 1062 (macrotonum); 1145 (macrotonum).

Kohn, E. 1319 (interandinum); 1419 (grandidentatum); 1469 (nigrescens).

Korte, A. 5244 (chenopodioides).

Kranz, W.M. 93 (americanum).

Krapovickas, A. 400 (triflorum); 1267 (pilcomayense); 2144 (nitidibaccatum); 2249 (pilcomayense); 2683 (pilcomayense); 2729 (triflorum); 2824 (chenopodioides); 3192 (sinuatirecurvum); 3275 (chenopodioides); 3616 (tweedieanum); 4348 (triflorum); 4566 (palitans); 4729 (palitans); 5032 (chenopodioides); 5091 (tweedieanum); 5097 (salicifolium); 5158 (tweedieanum); 5415 (riojense); 5425 (salicifolium); 5498 (riojense); 5503 (salicifolium); 5871 (riojense); 6036 (nitidibaccatum); 6098 (nitidibaccatum); 6209 (salicifolium); 6219 (weddellii); 6539 (pilcomayense); 6950 (tripartitum); 6951 (tripartitum); 7373 (chenopodioides); 8395 (longifilamentum); 8645 (longifilamentum); 12738 (pilcomayense); 13193 (pilcomayense); 14575 (salicifolium); 14602 (echegarayi); 16853 (chenopodioides); 17711 (zuloagae); 18617 (aloysiifolium); 18998 (aloysiifolium); 19000 (aloysiifolium); 19504 (aloysiifolium); 20006 (americanum); 20715 (tripartitum); 21885 (tiinae); 21900 (annuum); 22192 (pilcomayense); 22308 (triflorum); 22358 (tweedieanum); 24483 (tweedieanum); 24505 (tweedieanum); 25161 (americanum); 25658 (pilcomayense); 26156 (chenopodioides); 26554 (glandulosipilosum); 26584 (zuloagae); 26621 (huayavillense); 26622 (aloysiifolium); 26692 (aloysiifolium); 26740 (pilcomayense); 26900 (pilcomayense); 26983 (pilcomayense); 27060 (pygmaeum); 27124 (aloysiifolium); 27208 (enantiophyllanthum); 27213 (enantiophyllanthum); 27361 (pilcomayense); 27444 (pygmaeum); 27529 (chenopodioides); 27720 (chenopodioides); 27772 (tweedieanum); 27861 (aloysiifolium); 27876 (physalidicalyx); 27931 (tweedieanum); 27943 (aloysiifolium); 27969 (aloysiifolium); 27988 (physalidicalyx); 28006 (aloysiifolium); 28007 (physalidicalyx); 28364 (aloysiifolium); 28490 (aloysiifolium); 29326 (pilcomayense); 30220 (physalidicalyx); 30304 (aloysiifolium); 30524 (aloysiifolium); 30733 (tripartitum); 30856 (glandulosipilosum); 30961 (aloysiifolium); 31088 (michaelis); 31136 (michaelis); 31137 (michaelis); 31399 (aloysiifolium); 33562 (americanum); 35748 (tripartitum); 35875 (aloysiifolium); 36060 (physalidicalyx); 37363 (physalidicalyx); 37403 (tweedieanum); 39064 (aloysiifolium); 39304 (physalidicalyx); 40639 (pygmaeum); 44353 (sarrachoides); 44650 (americanum); 47316 (aloysiifolium); 47364 (caesium); 47399 (tweedieanum); 47506 (physalidicalyx); 47724 (sinuatirecurvum); 47749 (sarrachoides); 47818 (physalidicalyx).

Krause, L. 139 (americanum).

Kreibohm, E. 114 (triflorum).

Krieger, L. 3311 (americanum); CESJ-7199 (americanum); CESJ-15365 (americanum); 19452 (americanum).

Krukoff, B.A. 10365 (longifilamentum); 10378 (subtusviolaceum); 10470 (longifilamentum); 10485 (subtusviolaceum); 22067 (radicans).

Kubo, R.R. 101 (americanum).

Kuhlmann, J.G. 2602 (americanum).

Kujikat, A. 104 (longifilamentum); 457 (americanum).

Kuniyoshi, Y.S. 74 (americanum); 120 (americanum); 327 (americanum); 3880 (paucidens).

Kunkel, G.W.H. 1689 (120. (furcatum); 1690 -313 (furcatum); 5550a (cochabambense); 5555 (arequipense); 5556 (grandidentatum); 5560 (radicans); 5561a (radicans); 5561 (radicans); 5562 (radicans); 5563 (interandinum); 5614 (polytrichostylum); 5616 (radicans); 5680 (americanum); 5690 (polytrichostylum); 5693 (arenicola); 5694 (americanum); 5695 (americanum); 5696 (arequipense); 5698 (juninense).

Kuntze, O. 1657 (nigrescens); 2295 (nigrescens).

Kurtz, F. 30 (pilcomayense); 129 (echegarayi); 322 bis (tweedieanum); 376 (pilcomayense); 418 (pilcomayense); 418 (pilcomayense); 912 (pygmaeum); 918 (triflorum); 922 (triflorum); 923 (pygmaeum); 924 (chenopodioides); 954 (chenopodioides); 963 (chenopodioides); 1430 (pilcomayense); 1626 (pilcomayense); 1782 (pilcomayense); 1782 (pilcomayense); 2055 (pilcomayense); 2216 (chenopodioides); 2381 (chenopodioides); 2384 (tweedieanum); 2564[A] (chenopodioides); 3337 (tweedieanum); 3338 (triflorum); 3354 (triflorum); 3436b (echegarayi); 3437 (salicifolium); 3446 (echegarayi); 3463 (salicifolium); 3542 (echegarayi); 3542 bis (echegarayi); 3544 (echegarayi); 4012 (aloysiifolium); 4023 (aloysiifolium); 4612 (triflorum); 4685 (pygmaeum); 4707 (triflorum); 4923 (aloysiifolium); 5239 (pilcomayense); 5265 (pygmaeum); 5269 (pygmaeum); 5534b (triflorum); 5534a (triflorum); 5564 (tweedieanum); 6510 (tweedieanum); 6994a (nitidibaccatum); 7019 (salicifolium); 7051a (triflorum); 7053a (tweedieanum); 7261a (tweedieanum); 7315 (fiebrigii); 7577 (triflorum); 7674 (triflorum); 8172 (caesium); 8324 (salicifolium); 8509 (physalidicalyx); 8509[A] (tweedieanum); 8786 (salicifolium); 8822 (chenopodioides); 9090 (chenopodioides); 9245 (pygmaeum); 9322 (salicifolium); 9346 (echegarayi); 9389 (salicifolium); 9533 (echegarayi); 9757 (echegarayi); 9827 (salicifolium); 10276 (aloysiifolium); 10318 (chenopodioides); 10887 (salicifolium); 10900 (salicifolium); 10946 (tweedieanum); 11066 (echegarayi); 11160 (triflorum); 11754 (aloysiifolium); 11803 (aloysiifolium); 11877 (chenopodioides); 12841 (tweedieanum); 13058 (tweedieanum); 13366 (salicifolium); 13367 (echegarayi); 13427 (salicifolium); 13430 (tweedieanum); 13475 (salicifolium); 13659 (weddellii); 13739 (weddellii); 13806 (weddellii); 14179 (tweedieanum); 14180 (tweedieanum); 14181 (echegarayi); 14183 (salicifolium); 14200 (tweedieanum); 14205 (triflorum); 14427 (tweedieanum); 14471 (tweedieanum); 14518 (salicifolium); 14724 (weddellii); 14792 (weddellii); 14889 (chenopodioides); 14961 (aloysiifolium); 15008 (salicifolium); 15062 (salicifolium); 15143 (aloysiifolium); 15190 (aloysiifolium); 15191 (aloysiifolium); 15283 (echegarayi); 15416 (salicifolium); 15417 (aloysiifolium); 15421 (salicifolium); 15626 (aloysiifolium); 15727 (pygmaeum); 16057 (pygmaeum); 16149 (pygmaeum).

La Torre Cuadros, M.A. 007 (cochabambense).

La Torre, M.I. 417 (corymbosum); 426 (corymbosum); 1791 (arequipense); 1808 (arequipense); 1890 (arequipense); 2002 (grandidentatum); 2096 (grandidentatum); 3200 (fragile); 3346 (grandidentatum).

Labiak, P. 5940 (americanum).

Lagiglia, H.A. 1838 (triflorum); 2283 (tweedieanum); 2462 (tweedieanum); 2476 (triflorum); 3097 (salicifolium); 3107 (triflorum); 3323 (tweedieanum).

Lahitte, R. 293 (chenopodioides); 49660 (tweedieanum).

Landrum, L.R. 1506 (furcatum); 2594 (americanum); 4416 (furcatum); 4427 (nitidibaccatum); 5809 (aloysiifolium); 7429 (furcatum); 8888 (grandidentatum); 11508 (furcatum).

Lanfranchi, A.E. 931 (pilcomayense); 1635 (triflorum).

Langenheim, J.H. 3336 (americanum); 3546 (macrotonum).

Lanjouw, J. 3122 (americanum).

Lanstyak, L. 270 (enantiophyllanthum).

Lasser, T. 739 (americanum).

Lasso, S. 10 (interandinum); 11 (interandinum).

Lawrance, A.E. 168 (americanum).

Lazeo, F.S.M. 5 (americanum).

Lechler, W. 1939 (pallidum).

Ledezma, N. 719 (dianthum).

Legname, P.R. 94 (weddellii); 142 (salicifolium); 355 (physalidicalyx); 3282 (tweedieanum); 5656C (tweedieanum); 9716 (zuloagae); 10021 (zuloagae); 10418 C. (aloysiifolium); 10452C (aloysiifolium).

Legname, V. 516 (physalidicalyx).

Legrand, C.D. 419 (chenopodioides); 1917 (sarrachoides); 1918 (sarrachoides); 1961 (sarrachoides).

Leitão Filho, H.F. 13124 (chenopodioides).

Leitão, F. 111 (americanum); 133 (americanum).

Leite, J.E. 270 (americanum).

Leiva, S. 77 (grandidentatum); 697 (americanum); 699 (corymbosum); 707 (corymbosum); 740 (interandinum); 785 (polytrichostylum); 795 (interandinum); 987 (interandinum); 1390 (longifilamentum); 1396 (cochabambense); 5653 (americanum).

Lemes, F.O.A. 603 (americanum).

León G, J. 48 (americanum).

León, B. 5192 (grandidentatum); 5574 (grandidentatum); 5643 (juninense).

León, H. 17 (nigrescens).

León, R.J. 4071 (pygmaeum).

Leoni, L.S. 108 (americanum); 6554 (americanum).

Lero, G.C. 56 (longifilamentum).

Lescure, J.P. 630 (americanum).

Leveque, R. 36 (americanum); 200A (americanum).

Lewis, M.A. V-114 (aloysiifolium); 35007 (cochabambense); 35076 (antisuyo); 35077 (cochabambense); 35146 (gonocladum); 35154 (cochabambense); 35159 (cochabambense); 35233 (gonocladum); 35358 (cochabambense); 37053 (antisuyo); 37114 (palitans); 38709 (antisuyo); 39147 (pallidum); 39249 (antisuyo); 39356 (cochabambense); 39732 (antisuyo); 88105 (americanum); 88119 (antisuyo); 88375 (gonocladum); 88380 (gonocladum); 88592 (gonocladum); 88696 (sinuatiexcisum); 88950 (cochabambense); 88963 (cochabambense); 88996 (dianthum); 871047 (gonocladum); 871048 (cochabambense); 871070 (cochabambense); 871684 (tripartitum); 871740 (tripartitum); 871749 (pallidum); 881019 (cochabambense); 881031a (cochabambense); 881659 (cochabambense); 881761 (cochabambense); 881769 (fiebrigii); 881787 (cochabambense).

Lewis, W.H. 10197 (americanum); 14172 (longifilamentum).

Liane, ? 3661 (americanum).

Liberman, M. 434 (cochabambense); 819 (fragile); 1291 (gonocladum); 1722 (aloysiifolium).

Libermann, M. 19 (gonocladum).

Licata, A. 383 (americanum).

Lieberg, S.A. 23247 (americanum).

Liesner, R.L. 5388 (americanum); 7988 (macrotonum); 10147 (nigrescens); 12623 (nigrescens); 12908 (nigrescens); 12911 (macrotonum); 13443 (americanum).

Lillo, M. 3851 (glandulosipilosum); 8691 (tiinae); 10884 (caesium); 13245 (glandulosipilosum).

Lima, J.R. 84 (americanum).

Lima-Verde, L.W. 360 (americanum); 921 (americanum).

Limbach, C.F. 145 (americanum).

Lindeman, J.C. 1373 (americanum); 2614 (americanum); 4156 (enantiophyllanthum); 5339 (paucidens).

Linden, J.J. 2204 (nigrescens).

Linneo, I.I. 831 (tripartitum); 951 (cochabambense); 955 (tripartitum); 1184 (aloysiifolium).

Lisakowski, ? 26 (americanum).

Llanos, F. 1785 (nigrescens).

Llatas Quiroz, S. 635 (radicans); 639 (interandinum); 642 (americanum); 645 (corymbosum); 645 (corymbosum); 702 (pseudoamericanum); 708 (cochabambense); 733 (americanum); 866 (pseudoamericanum); 869 (interandinum); 878 (interandinum); 1021 (interandinum); 1027 (radicans); 2348 (pseudoamericanum); 3597 (grandidentatum); 3672 (grandidentatum).

Lleras, E. 1943 (americanum); 1943 (americanum).

Lligado, S.J. 51 (interandinum).

Lliully, A.E. 17 (cochabambense); 24 (fiebrigii); 349 (aloysiifolium); 411 (tripartitum); 627 (aloysiifolium); 1010 (caesium); 1280 (aloysiifolium); 1331 (aloysiifolium); 1510 (fiebrigii); 1518 (aloysiifolium); 1736 (aloysiifolium); 1760 (aloysiifolium); 1804 (tripartitum).

Lobão, A.Q. 13 (americanum); 45 (americanum).

Lojtnant, B. 14431 (interandinum); 14880 (interandinum); 15448 (interandinum).

Lombardi, J.A. 8258 (paucidens).

Lombardo, A. 983[a] (chenopodioides); 983[b] (chenopodioides).

Londoño U, R. 131 (macrotonum).

Longhi-Wagner, H. 2833 (enantiophyllanthum).

Loos, A. 14 (echegarayi); 41 (echegarayi); 3663 (salicifolium).

Looser, G. 99 H (furcatum); 141 H (furcatum); 900 (furcatum); 4451 (furcatum).

López C, R. 7017 (nigrescens); 7828 (americanum).

López R, C.A. 48 (macrotonum).

López T, E. 035 (arequipense).

López, ? [Chaco, Argentina] 8194 (pilcomayense).

López, A. 8 (nigrescens).

López, H. 578 (grandidentatum); 580 (pentlandii); 581 (grandidentatum); 584 (interandinum).

López, H.A. 123 (tweedieanum); 197 (chenopodioides); 235 (chenopodioides); 304 (tweedieanum); 421 (fiebrigii).

López, L. 31 (nigrescens).

López, N. de 73 (nigrescens); 417 (nigrescens); 462 (nigrescens); 463 (nigrescens); 924 (americanum).

López-Palacios, S. 1478 (interandinum); 2042 (nigrescens).

Lorentz, P.G. 26 (chenopodioides); 83 (pygmaeum); 84 (chenopodioides); 100 (chenopodioides); 101 bis (chenopodioides); 106[b] (pygmaeum); 121 (aloysiifolium); 190 (chenopodioides); 193 (palitans); 194 (palitans); 215 (aloysiifolium); 351 (caesium); 364 (physalidicalyx); 418 (salicifolium); 440 (aloysiifolium); 440[b] (aloysiifolium); 441 (tweedieanum); 550 (pygmaeum); 565 (tiinae); 584 (aloysiifolium); 706 (aloysiifolium); 722 (tripartitum); 732 (aloysiifolium); 812 (tripartitum); 818 (fiebrigii); 899[a] (huayavillense); 899[b] (fiebrigii); 914 (glandulosipilosum); 999 (aloysiifolium); 1035 (glandulosipilosum); 1036 (aloysiifolium); 1074 (aloysiifolium); 1132 (triflorum); 1155 (cochabambense).

Lorenzi, H. 5747 (paucidens).

Lourteig, A. 716 (salicifolium); 1135 (americanum); 1912 (americanum).

Loveless, L. 1852 (interandinum).

Lowe, J. 4153 (americanum).

Loza, M.I. 1086 (antisuyo); 1626 (longifilamentum).

Lozano C, G. 2899 (nigrescens).

Lozano, R. 1257 (aloysiifolium); 1462 (huayavillense); 1530 (fiebrigii); 1671 (aloysiifolium); 1703 (aloysiifolium); 1787 (alliariifolium); 2028 (glandulosipilosum); 3138 (aloysiifolium).

Lucas, C.I.S. 43 (americanum).

Lucas, E.J. 495 (paucidens).

Luetzelburg, P. 28641 (caatingae).

Lugo S, H. 1266 (interandinum); 1297 (interandinum); 6082 (americanum); 6094 (americanum).

Lujan, M.C. 11 (salicifolium).

Luna Risso, J. 14 (tweedieanum).

Luna, A. 70 (pseudoamericanum).

Luna, F.E. 4[b] (physalidicalyx); 148 (pilcomayense); 363 (physalidicalyx); 507 (salicifolium); 914 (tweedieanum); 1014 (aloysiifolium); 1477 (pilcomayense).

Lund, P.W. 59 (americanum).

Lurvey, E. 480 (americanum).

Luteyn, J.L. 4816 (macrotonum); 5443 (cochabambense); 6431 (pallidum); 12126 (nigrescens); 13815 (pallidum); 13822 (pallidum).

Luz, ? 9 (americanum).

Lyra-Lemos, R.P. 605 (americanum); 1310 (americanum).

Løjtnant, B. 15485 (radicans); 15898 (radicans); 15988 (radicans).

Macbride, J.F. 380 (arequipense); 665 (interandinum); 1213 (juninense); 1630 (cochabambense); 1865 (antisuyo); 2079 (interandinum); 2966 (arequipense); 3089 (juninense); 3385 (cochabambense); 3879 (interandinum); 5314 (arenicola).

MacBryde, B. 1033 (americanum); 1061 (americanum); 1111 (americanum); 1269 (macrotonum); 1570 (interandinum).

MacDougal, J.M. 3754 (americanum).

Machado, B. 1826 (americanum).

Machado, D.N.S. 337 (americanum).

Maciel, A.A. 40 (americanum).

Macmillan, H.G. 22 (furcatum).

Madison, M.T. 10362-70 (cochabambense).

Madrigal, B. 540 (americanum).

Madsen, J.E. 36541 (radicans); 50034 (radicans); 50044 (interandinum); 63299 (americanum); 63358 (americanum); 63457 (americanum); 75741 (antisuyo).

Mahu, M. 7617 (furcatum).

Maioli, V. 168 (americanum).

Maldonado, C. 3077 (longifilamentum).

Malme, G.A. 2956 (echegarayi).

Malme, G.O.A. 1544 (americanum).

Malvárez, M.R. 101 (aloysiifolium).

Mandon, G. 395 (sinuatiexcisum); 396 (cochabambense); 398 bis (physalifolium); 402 (physalifolium); 404 (leptocaulon); 405ter (pallidum); 406 (pallidum); 407 (antisuyo); 409 (pallidum); 410 (cochabambense); 410[b] (pallidum).

Manetti, L. 2105 (glandulosipilosum).

Mansano, V.F. 06-357 (paucidens).

Mantílla, A. 41 (interandinum).

Mantuano, M. 64 (americanum).

Marcelo Peña, J.L. 1895 (juninense).

Marcelo, J. 62 (americanum); 63 (americanum).

Marchett, F. 327 (americanum).

Marchioretto, M.S. 80 (chenopodioides).

Marcolino, F. 163 (americanum).

Marcondes-Ferreira, W. 1752 (americanum).

Marelli, C.A. 20 (triflorum); 21 (nitidibaccatum).

Marin, F. 35 (pentlandii).

Marín, F. 242 (grandidentatum); 1456 (cochabambense); 1949 (cochabambense).

Marín, M.F. 66 (nigrescens).

Marmol, L.A. 9237 (zuloagae); 9273 (zuloagae).

Marodin, S.M. 368 (americanum).

Marquete, N. 535 (americanum).

Marquete, R. 3357 (enantiophyllanthum); 4312 (americanum); 4555 (americanum).

Marticorena, C. 1077 (furcatum).

Martin, J.S. 17 (americanum).

Martinelli, G. 2444 (enantiophyllanthum); 2452 (enantiophyllanthum); 2454 (enantiophyllanthum); 2470 (enantiophyllanthum); 2482 (paucidens); 2492[A] (paucidens); 2492 (enantiophyllanthum); 3579 (americanum); 3969 (americanum).

Martinet, J.P. 135 (arequipense); 233 (antisuyo); 774 (arequipense); 988 (antisuyo).

Martínez Carretero, E. 105 (echegarayi); 126 (echegarayi); 135 (echegarayi); 1519 (echegarayi).

Martínez Crovetto, R. 6071 (aloysiifolium).

Martínez, A.J. 4854 -A (tweedieanum); 4854 -B (tweedieanum).

Martínez, E. 698 (antisuyo).

Martínez-Crovetto, R. 1508 (chenopodioides); 1750 (triflorum); 1923 (triflorum); 1975 (chenopodioides); 2507 (triflorum); 4201 (americanum); 4444 (americanum); 4762 (chenopodioides); 4954 (americanum); 5068 (chenopodioides); 6242 (aloysiifolium); 6261 (palitans); 9278 (chenopodioides).

Martínez-Ochenbach, S. 2068 (pilcomayense).

Martins, S.A. 28 (americanum).

Martius, C.F.P. 1255 (scabrum).

Maruñak, V. 209 (tiinae); 507 (aloysiifolium).

Matesevach, A.M. 6 (tweedieanum); 10A (nitidibaccatum); 10C (marmoratum); 10B (tweedieanum); 38 (triflorum); 41 (sinuatirecurvum); 43 (sinuatirecurvum); 49 (sinuatirecurvum); 50 (tripartitum); 52 (tripartitum).

Mathews, A. 144 H (corymbosum); 270 (furcatum); 730 (americanum); 1509 (juninense).

Matos, E.C.A. 7 (americanum).

Matthei, O. 73 (furcatum); 138 (nitidibaccatum); 214 (sinuatirecurvum); 400 (radicans); 510 (americanum).

Mattos Silva, L.A. 3509 (americanum).

Mattos, J.R. 50 (americanum); 74 (americanum).

Matzenbacher, N.I. 3064 (americanum).

Mauad, L.P. 29 (americanum); 443 (americanum).

Mautone, L. D 23 (americanum); 554 (americanum).

Mazzucconi, V.J. 1547 (triflorum).

McCook, L. 1118 (macrotonum).

McDaniel, S. 13861 (americanum); 22404 (americanum); 23804 (americanum); 25378 (americanum); 30219 (americanum); 32880 (americanum).

McDowell, T. 2604 (americanum); 3207 (americanum).

Medeiros, E.V.S. 395 (americanum).

Medina, A. 4 (macrotonum).

Medri, C. 303 (americanum).

Meglioli, C. 52 (salicifolium).

Meier, W. 3769 (macrotonum); 4865 (macrotonum); 6425 (nigrescens); 8465 (nigrescens); 8674 (nigrescens); 9345 (nigrescens); 12349 (nigrescens); 12442 (nigrescens); 16386 (americanum).

Meireles, L.D. 455 (enantiophyllanthum); 586 (enantiophyllanthum); 643 (enantiophyllanthum); 690 (enantiophyllanthum); 1857 (enantiophyllanthum); 1883 (enantiophyllanthum); 2312 (enantiophyllanthum); 2872 (chenopodioides); 3089 (enantiophyllanthum).

Mejía P, F. 278 (nigrescens).

Mejía, A. 109 (interandinum).

Melampy, M. 904 (macrotonum).

Mello-Silva, R. 2058 (cochabambense); 2110 (cochabambense); 2126 (sinuatiexcisum); 2603 (enantiophyllanthum).

Melo, P.H.A. 875 (americanum).

Mena, P. 123 (macrotonum); 4066 (interandinum).

Méndez C, V. 170 (nigrescens).

Méndez, E. 9923 (echegarayi);10512 (triflorum); 10544 (triflorum); 10559 (triflorum).

Mendoza, M. 449 (rhizomatum); 491 (fiebrigii); 529 (rhizomatum); 872 (fragile); 1041 (cochabambense); 1282 (aloysiifolium); 1483 (aloysiifolium); 1600 (aloysiifolium); 2737 (physalidicalyx).

Meneghel, R. 34 (americanum).

Mentz, L.A. 33 (americanum); 51 (americanum); 82 (paucidens); 164 (paucidens); 173 (americanum); 353 (americanum); 359 (paucidens); 434 (americanum); 438 (americanum); 498 (americanum); 2888 (paucidens).

Mereles, F. 3420 (pilcomayense); 3938 (salicifolium); 5421 (pilcomayense); 6028 (pilcomayense); 6057 (pilcomayense); 6078 (pilcomayense); 6105 (pilcomayense); 9992 (tweedieanum).

Metcalf, R.D. 30293 (pentlandii); 30373 (pentlandii); 30431 (pallidum); 30698 (interandinum); 30725 (pallidum).

Mexia, Y. 4251 (interandinum); 4336 (americanum); 4529 (americanum); 5854 (americanum); 5930 (americanum); 6298 (americanum); 7408 (interandinum); 8079 (polytrichostylum).

Meyer, T. 102 (pilcomayense); 16-371 (fiebrigii); 2164 (glandulosipilosum); 3649 (aloysiifolium); 3659 (aloysiifolium); 4318 (aloysiifolium); 4544 (cochabambense); 5424 (glandulosipilosum); 12248 (tweedieanum); 12856 (tweedieanum); 13951 (pilcomayense); 15287 (salicifolium); 15555 (salicifolium); 20523 (huayavillense); 20747 (cochabambense); 22321 (aloysiifolium); 23339 (tweedieanum); 23584 (physalidicalyx).

Meza Torres, E.I. 856 (triflorum).

Miccis Peralta, L.R. 711 (nitidibaccatum).

Michel, B. 375 (caesium).

Michel, R. de 97 (aloysiifolium); 2532 (arenicola); 2677 (michaelis); 2721 (arenicola); 2769 (sarrachoides).

Miers, J. 276 (furcatum); 644 (salicifolium); 1235 (chenopodioides); 1412 (chenopodioides); 1427 (chenopodioides); 1428 (chenopodioides); 1435 (salicifolium); 1436 (palitans); 1815 (americanum); 3540 (paucidens); 4540[a] (paucidens); 4540[b] (americanum).

Millan, B. 01 (americanum).

Mille, L. 977 (americanum).

Minga, D. 453 (radicans).

Mintzer, M. 1 (palitans).

Miranda, A.M. 2859 (americanum); 3483 (americanum); 3641 (americanum).

Miranda, T. 580 (cochabambense).

Mocquerys, A. 163 (americanum); 763 (nigrescens); 1164 (nigrescens).

Molas, L. 1391 (sarrachoides).

Molina M, A. 7 (nigrescens).

Molina, A. 209 (americanum).

Molina, A.M. 1..561 (tweedieanum); 133 (tweedieanum); 257 (nitidibaccatum); 1210 (tweedieanum); 1402 (tweedieanum); 1544 (pygmaeum); 1601 (aloysiifolium); 1770 (aloysiifolium); 1802 (aloysiifolium); 2132 (pilcomayense); 2323 (nitidibaccatum); 2427 (pilcomayense); 2429 (pilcomayense); 2734 (tweedieanum); 3495 (salicifolium); 3501 (echegarayi); 3502 (echegarayi); 3681 (tweedieanum); 3759 (annuum).

Molinari, E. 186 (chenopodioides).

Molliner, N. 23 (radicans).

Monetti, L. 55 (aloysiifolium); 127 (aloysiifolium); 1838 (hunzikeri); 2248 (physalidicalyx).

Monro, A.K. 3953 (americanum); 3979 (radicans); 3982 (polytrichostylum); 4006 (subtusviolaceum); 4043 (cochabambense).

Monteagudo, A. 3820 (longifilamentum); 7326 (antisuyo); 13936 (longifilamentum); 14140 (antisuyo); 15072 (subtusviolaceum); 15724 (longifilamentum); 15798 (longifilamentum); 16161 (juninense); 16477 (antisuyo); 16710 (longifilamentum).

Monteiro, O.P. 76-886 (americanum).

Monteiro, T. dos PSACF_EX 3733 (americanum).

Montenegro, ? 451 (aloysiifolium).

Montero O, G. 212 (furcatum); 5618 (furcatum).

Montes, J.E. 943 (americanum); 1226 (americanum); 1545[b] (pilcomayense); 2128 (americanum); 2143 (americanum); 2324 (americanum); 12297 (americanum); 16065 (americanum).

Montesinos, D. 920 (gonocladum); 2620 (grandidentatum); 3627 (fragile); 3725 (fragile); 3730 (weddellii); 3833 (fragile).

Montesinos, D.B. 2144 (weddellii).

Moonlight, P.W. 94 (antisuyo); 141 (interandinum); 266 (juninense); 267 (longifilamentum); 306a (longifilamentum).

Mora G, R.A. 119 (interandinum).

Mora O, L.E. 666 (nigrescens); 7521 (interandinum); 7527 (interandinum).

Moraes, M. 116 (tripartitum); 207 (americanum); 1198 (arenicola).

Morales, ? 25 (macrotonum).

Morales, G. 622 (interandinum); 1420 (interandinum).

Morales, M. 3733 (interandinum); 3985 (corymbosum).

Moreira, D.M. 199 (americanum).

Moreira, E. 384 (americanum).

Moreira, H. 239 (chenopodioides).

Morel, I. 87 (pilcomayense); 254 (pilcomayense); 1393 (pilcomayense); 1623 (pilcomayense); 1904 (pilcomayense); 2740 (americanum); 2857 (americanum); 2930 (americanum); 2957 (pilcomayense); 3466 (americanum); 3740 (pilcomayense); 3749 (americanum); 4098 (pilcomayense); 4349 (pilcomayense); 4956 (pilcomayense); 5074 (pilcomayense); 5296 (pilcomayense); 5782 (americanum); 5903 (pilcomayense); 5984 (pilcomayense); 6218 (pilcomayense); 6307 (pilcomayense); 6512 (pilcomayense); 6656 (pilcomayense); 6991 (pilcomayense); 7669 (pilcomayense); 8047 (pilcomayense); 8247 (pilcomayense); 8313 (pilcomayense); 8579 (pilcomayense); 8618 (pilcomayense); 8711 (pilcomayense); 8731 (pilcomayense); 8853 (pilcomayense); 9405 (pilcomayense).

Morello, J. 959 (zuloagae).

Moreno B, L.M. 343 (nigrescens).

Moreno, P. 301 (macrotonum).

Morero, R.E. 75b (chenopodioides); 110 (chenopodioides).

Moretti, C. 1168 (americanum).

Mori, S.A. 10992 (americanum); 11624 (americanum).

Moritz, J.W.K. 1643 (macrotonum).

Morong, T. 9 (pygmaeum); 27 (paucidens); 262 (americanum); 898 (pilcomayense); 1104 (furcatum); 1117 (radicans).

Morrone, O. 646 (americanum); 1286 (americanum); 2366 (aloysiifolium); 2379 (tweedieanum); 2460 (cochabambense); 2748 (tripartitum); 3114 (cochabambense); 3261 (fiebrigii); 3489 (aloysiifolium); 3494 (aloysiifolium); 3840 (cochabambense); 4010 (caesium).

Moscone, E.A. 50 (chenopodioides); 59 (chenopodioides); 65 (chenopodioides); 84 (chenopodioides); 92 (salicifolium); 99 (pygmaeum); 105 (chenopodioides); 228 (americanum); 232 (americanum); 233 (americanum); 241 (chenopodioides); 244 (chenopodioides).

Mosén, C.W.H. 4483 (americanum).

Mostacedo, B. 155 (arenicola); 380 (michaelis).

Mostacero L, J. 941 (cochabambense); 3016 (interandinum).

Motta, J.T. 98 (americanum); 101 (americanum); 1115 (americanum); 1198 (americanum).

Moura, T.M. 654 (chenopodioides).

Moura-Júnior, E.G. 8 (americanum).

Moya, A. 39 (interandinum).

Múlgura, M.E. 199 (echegarayi); 740 (tweedieanum); 1228 (tripartitum); 1238 (physalifolium); 1254 (sinuatirecurvum); 2455 (paucidens); 3179 (americanum).

Müller, G.K. 3639 (nitidibaccatum); 3661 (radicans); 9544 (arequipense).

Müller, J. 6458 (gonocladum).

Müller, N. von 308 (chenopodioides).

Muñoz, E. 301 (nigrescens); 327 (nigrescens).

Muñoz, L. 223 (interandinum).

Murcia, C. 475 (nigrescens).

Murphy, H. 314 (americanum); 475 (americanum).

Musch, P. 67 (cochabambense).

Mutis D, E. 6 (interandinum).

Mutis, J.C. 3562 (interandinum); 3569 (nigrescens); 4650 (interandinum).

Nadruz, M. 2184 (paucidens).

Naessany, L. 37 (tripartitum).

Naganuma, R. 01 (americanum).

Nájera, J.J. 43 (triflorum).

Naranjo, C. 907 (salicifolium); 921 (tweedieanum).

Narváez, E. 628 (interandinum).

Nee, M. 3321 (gonocladum); 3397 (americanum); 3407 (americanum); 3435 (americanum); 3722 (interandinum); 3785 (nigrescens); 3870 (macrotonum); 3923 (nigrescens); 3966 (americanum); 4021 (macrotonum); 4043 (americanum); 4138 (macrotonum); 4176 (americanum); 16839 (macrotonum); 16872 (nigrescens); 16902 (nigrescens); 17003 (macrotonum); 30176 (tripartitum); 30178 (gonocladum); 30179 (tripartitum); 30180 (tripartitum); 30188 (antisuyo); 30191 (pallidum); 30208 (albescens); 30215 (antisuyo); 30217 (dianthum); 30224 (longifilamentum); 30237 (americanum); 30257 (americanum); 30275 (americanum); 30332 (antisuyo); 30336 (gonocladum); 30342 (fiebrigii); 30349 (palitans); 30362 (tripartitum); 30392 (cochabambense); 30396 (fiebrigii); 30440 (tripartitum); 30443 (tripartitum); 30484 (aloysiifolium); 30488 (americanum); 30522 (gonocladum); 31258 (americanum); 31331 (americanum); 31350 (americanum); 31398 (arenicola); 31415 (americanum); 31483 (arenicola); 31497 (arenicola); 31508 (americanum); 31578 (americanum); 31724 (arenicola); 31757 (arenicola); 31760 (arenicola); 31813 (americanum); 31821 (arenicola); 31899 (americanum); 31952 (pallidum); 31956 (dianthum); 32011 (americanum); 32050 (longifilamentum); 32053 (sinuatiexcisum); 32057 (tripartitum); 32058 (tripartitum); 32059 (palitans); 33282 (tripartitum); 33315 (tripartitum); 33321 (gonocladum); 33342 (americanum); 33461 (aloysiifolium); 33520 (americanum); 33880 (aloysiifolium); 33889 (aloysiifolium); 33891 (aloysiifolium); 33909 (fiebrigii); 33934 (tripartitum); 33936 (palitans); 33940 (palitans); 33947 (rhizomatum); 33955 (tweedieanum); 33959 (tripartitum); 33961 (aloysiifolium); 33972 (tripartitum); 34044 (leptocaulon); 34052 (dianthum); 34055 (sinuatiexcisum); 34072 (leptocaulon); 34074 (dianthum); 34085 (palitans); 34089 (tripartitum); 34097 (cochabambense); 34107 (rhizomatum); 34108 (gonocladum); 34112 (cochabambense); 34162 (palitans); 34163 (tripartitum); 34164 (cochabambense); 34174 (tripartitum); 34175 (tripartitum); 34176 (gonocladum); 34965 (americanum); 35134 (caesium); 35436 (arenicola); 35614 (caesium); 36087 (aloysiifolium); 36149 (palitans); 36201 (cochabambense); 36252 (rhizomatum); 36450 (cochabambense); 36461 (tripartitum); 36462 (palitans); 36464 (cochabambense); 36465 (tripartitum); 36473 (leptocaulon); 36506 (dianthum); 36518 (aloysiifolium); 36536 (rhizomatum); 36555 (aloysiifolium); 36609 (leptocaulon); 36631 (alliariifolium); 36635 (cochabambense); 36643 (cochabambense); 36668 (tripartitum); 36671 (sinuatiexcisum); 36676 (cochabambense); 36714 (cochabambense); 36737 (interandinum); 36738 (interandinum); 37163 (americanum); 37245 (arenicola); 37350 (longifilamentum); 37436 (aloysiifolium); 37477 (fiebrigii); 37561 (cochabambense); 37562 (cochabambense); 37592 (polytrichostylum); 37594 (cochabambense); 37600 (tripartitum); 37603 (pentlandii); 37773 (arenicola); 38303 (aloysiifolium); 38557 (huayavillense); 38763 (americanum); 38952 (aloysiifolium); 39727 (aloysiifolium); 39728 (aloysiifolium); 40315 (alliariifolium); 40843 (aloysiifolium); 42104 (caesium); 42350 (americanum); 42564 (americanum); 43054 (aloysiifolium); 43057 (physalifolium); 43058 (palitans); 43068 (palitans); 43069 (fiebrigii); 44688 (aloysiifolium); 44742 (woodii); 45983 (americanum); 46527 (tripartitum); 46692 (woodii); 47396 (sarrachoides); 47615 (cochabambense); 47673 (tweedieanum); 49056 (woodii); 50081 (antisuyo); 50082 (americanum); 50096 (aloysiifolium); 50153 (aloysiifolium); 50529 (tripartitum); 50626 (dianthum); 50741 (aloysiifolium); 50753 (aloysiifolium); 50793 (glandulosipilosum); 50823 (woodii); 50827 (physalidicalyx); 50829 (physalidicalyx); 50843 (riojense); 50846 (sinuatirecurvum); 50847 (salicifolium); 51080 (nitidibaccatum); 51732 (caesium); 51737 (cochabambense); 51752 (cochabambense); 51759 (pallidum); 51760 (dianthum); 51766 (sinuatiexcisum); 51768 (antisuyo); 51774 (longifilamentum); 51788 (pallidum); 51794 (longifilamentum); 51801 (cochabambense); 51805 (sinuatiexcisum); 51807 (antisuyo); 51808 (sinuatiexcisum); 51817 (pentlandii); 51821 (cochabambense); 51829 (cochabambense); 51837 (cochabambense); 51846 (cochabambense); 51847 (cochabambense); 51850 (dianthum); 51867 (cochabambense); 51967 (woodii); 52136 (cochabambense); 52148 (leptocaulon); 52191 (physalifolium); 52420 (dianthum); 52539 (dianthum); 52802 (aloysiifolium); 52806 (aloysiifolium); 52945 (physalifolium); 52962 (tripartitum); 52967 (americanum); 53230 (aloysiifolium); 53255 (aloysiifolium); 53579 (cochabambense); 53580 (cochabambense); 53585 (radicans); 53590 (americanum); 53599 (radicans); 53600 (radicans); 53774 (aloysiifolium); 53903 (alliariifolium); 53930 (cochabambense); 54063 (caesium); 54821 (michaelis); 54866 (aloysiifolium); 54867 (americanum); 54876 (michaelis); 54919 (palitans); 54923 (aloysiifolium); 54931 (aloysiifolium); 54935 (caesium); 54938 (tweedieanum); 54940 (aloysiifolium); 54947 (aloysiifolium); 54978 (aloysiifolium); 55034 (physalifolium); 55069 (physalifolium); 55081 (aloysiifolium); 55194 (arenicola); 55229 (americanum); 55273 (longifilamentum); 55287 (subtusviolaceum); 55288 (antisuyo); 55311 (leptocaulon); 55317 (dianthum); 55325 (dianthum); 55330 (gonocladum); 55331 (gonocladum); 55333a (cochabambense); 55360 (dianthum); 55364 (leptocaulon); 55367 (dianthum); 55397 (aloysiifolium); 56015 (physalifolium); 56231 (interandinum); 56526 (subtusviolaceum); 56545 (subtusviolaceum); 57594 (rhizomatum); 57674 (aloysiifolium); 59918 (aloysiifolium); 60861 (longifilamentum).

Negritto, M.A. 290 (palitans); 292 (zuloagae); 310 (tripartitum); 314 (tripartitum); 352 (sinuatirecurvum); 353 (weddellii); 372 (tripartitum); 394 (weddellii); 395 (sinuatirecurvum); 428 (weddellii); 429 (gilioides); 432 (sinuatirecurvum).

Nelson, B.W. 782 (americanum).

Nelson, E. 12509 (cochabambense); 12516 (cochabambense).

Nelson, J. 52775 (triflorum).

Neto, L. 14 (americanum).

Neves, R. 222 (americanum); 226 (americanum).

Nicora, E.G. 3731 (triflorum); 4330 (salicifolium); 4447 (echegarayi); 8866 (sinuatirecurvum).

Niederlein, G. 262 (pilcomayense); 270 (tweedieanum); 284 (pilcomayense).

Noblick, L.R. 4260 (americanum).

Nores, M. 330 (caesium).

Novara, L.J. 285 (aloysiifolium); 569 (aloysiifolium); 588 (cochabambense); 595 (palitans); 652 (salamancae); 653 (palitans); 668 (salamancae); 670 (salamancae); 733 (aloysiifolium); 958 (aloysiifolium); 972 (aloysiifolium); 1588 (palitans); 2642[a] (palitans); 2642[b] (annuum); 3373 (physalidicalyx); 3397 (aloysiifolium); 3433 (zuloagae); 3552 (aloysiifolium); 3580 (aloysiifolium); 3581 (caesium); 3594 (aloysiifolium); 3601 (michaelis); 3688 (aloysiifolium); 3815 (fiebrigii); 3915 (aloysiifolium); 3951 (pilcomayense); 4153 (palitans); 4552 (tweedieanum); 4807 (palitans); 4823 (fiebrigii); 4885 (salamancae); 5247 (fiebrigii); 5441 (tripartitum); 5560 (aloysiifolium); 5875 (tweedieanum); 5882 (aloysiifolium); 5886 (aloysiifolium); 6061 (zuloagae); 6090 (tweedieanum); 6185 (physalidicalyx); 6199 (physalidicalyx); 6226 (aloysiifolium); 6231 (aloysiifolium); 6235 (aloysiifolium); 6284 (aloysiifolium); 6390 (aloysiifolium); 6472 (tripartitum); 6545 (aloysiifolium); 7194 (tweedieanum); 7375 (palitans); 7415 (aloysiifolium); 7461 (profusum); 7501 (aloysiifolium); 7699 (aloysiifolium); 7738 (tweedieanum); 7786 (aloysiifolium); 7860 (aloysiifolium); 7904 (palitans); 7937 (aloysiifolium); 8023 (physalidicalyx); 8106 (aloysiifolium); 8125 (palitans); 8186 (tripartitum); 8247 (aloysiifolium); 8276 (palitans); 8585 (zuloagae); 8700 (palitans); 8746 (physalifolium); 8805 (aloysiifolium); 8876 (aloysiifolium); 9223 (salicifolium); 9246 (aloysiifolium); 9509 (tweedieanum); 9533 (palitans); 9534 bis (aloysiifolium); 9566 (tweedieanum); 9594 (salamancae); 9637 (salamancae); 9830 (aloysiifolium); 10271 (tripartitum); 10273 (salamancae); 10441 (cochabambense); 10581 (fiebrigii); 10897 (riojense); 10961 (zuloagae); 11212 (aloysiifolium); 11631 (aloysiifolium); 11632 (aloysiifolium); 11822 (tweedieanum); 11912 (aloysiifolium); 12202 (aloysiifolium); 12208 (aloysiifolium); 13025 (aloysiifolium).

Nugteren, Y.A. 41 (grandidentatum).

Nunes, T.S. 1398 (americanum).

Nunes, V.F. 156 -7 (americanum); 386 (chenopodioides).

Núñez V, P. 6017 (americanum); 6490 (americanum); 6667 (pallidum); 7015 (pentlandii); 7040 (cochabambense); 7074 (cochabambense); 7293 (radicans); 7311 (cochabambense); 7339 (cochabambense); 7362 (pentlandii); 7370 (cochabambense); 7460 (radicans); 7579 (longifilamentum); 7746 (dianthum); 7865 (fragile); 7943 (weddellii); 7962 (pentlandii); 8116 (grandidentatum); 8474 (antisuyo); 8601 (americanum); 8650 (polytrichostylum); 8666 (pentlandii); 8764 (americanum); 9079 (pallidum); 9158 (fiebrigii); 9191 (fiebrigii); 9199 (cochabambense); 9227 (cochabambense); 9229 (cochabambense); 9297 (radicans); 9514 (americanum); 12319 (americanum); 13106 (pallidum); 20824 (americanum).

Núñez, E.M. 5 (radicans); 6 (arequipense); 66 (radicans).

Núñez, T. 045 (radicans); 45 (radicans).

Núñez, V. 335 (zuloagae); 524 (palitans).

O'Donell, C.A. 313 (salicifolium); 805 (salicifolium); 909 (tweedieanum); 1015 (salicifolium); 1016 (salicifolium); 1140 (nitidibaccatum); 3150 (aloysiifolium); 3902 (triflorum); 3913 (sinuatirecurvum); 4490 (chenopodioides); 5152 (echegarayi).

Oberti, J.C. CORD 297 (triflorum).

Obregón B, R. 17C 911 (americanum).

Ocanto, N. 9 (salicifolium).

Occhioni, E.M. 526 (americanum).

Occhioni, P. 459 (americanum).

Ochoa, C.M. 53 (pallidum); 54 (gonocladum); 225 (gonocladum); 292 (cochabambense); 336 (cochabambense); 2688 (furcatum); 5079 (fragile); OCH 11161 (macrotonum); OCH 11781 (macrotonum); 12022 (alliariifolium); 13254 (longifilamentum); OCH 14244 (fragile); 14528 (interandinum); 14544 (longifilamentum); 14545 (cochabambense); OCH 14625 (corymbosum); 14627 (arequipense); 14648 (interandinum); 14662 (arequipense); 14901 (arequipense); 14997 (pallidum); 15025 (arenicola); 15043 (americanum); 15047 (pallidum); 15048 (pallidum); 15058 (pallidum); 15107 (cochabambense); 15457 (pentlandii); 15464 (cochabambense); 15485 (cochabambense); 15531 (rhizomatum); 15535 (cochabambense); 15548 (rhizomatum); 16093 (cochabambense); 16133 (longifilamentum).

Ohashi, D. 65 (americanum).

Oliveira de Britto, Y.L. 242 (americanum).

Oliveira Filho, N.E. 74 (americanum).

Oliveira, A.A. 2883 (americanum).

Oliveira, D.G. 292 (americanum).

Oliveira, D.M. 142 (americanum).

Oliveira, J.S. 36 (americanum).

Olmstead, R.J. 2007 -95 -95 (triflorum).

Olsen, J. 437 (radicans); 443 (radicans).

Onishi, E. 33 (americanum).

Ordóñez, M.T. 14 (americanum).

Ordoñez, O. 14 (americanum).

Orejuela, A. 221 (nigrescens); 694 (nigrescens); 696 (macrotonum); 701 (macrotonum); 762 (macrotonum); 2699 (americanum); 2707 (americanum); 2713 (longifilamentum); 2714 (longifilamentum); 2754 (americanum); 2757 (longifilamentum); 2819 (americanum); 2833 (subtusviolaceum); 2835 (americanum); 2842 (polytrichostylum); 2846 (sinuatiexcisum); 2863 (longifilamentum); 2870 (pallidum); 2886 (cochabambense); 2896 (americanum); 2903 (pseudoamericanum).

Orellana, R. 328 (arenicola).

Orlandini, P. 182 (americanum).

Orozco, C.I. 1833 (macrotonum); 2045 (nigrescens); 3374 (nigrescens); 3699 (macrotonum); 3786 (macrotonum); 3821 (macrotonum); 3869 (macrotonum); 3907 (longifilamentum); 3911 (longifilamentum); 3914 (nigrescens); 3941 (antisuyo); 3959 (interandinum).

Orsini V, G. 27 (nigrescens).

Ortega, L.C. de 123 (americanum); 1164 (americanum); 1528 (americanum); 1828 (americanum); 2096 (americanum).

Ortiz Adrián, C. 52 (interandinum).

Ortiz V, E. 140 (americanum); 935 (subtusviolaceum); 1215 (americanum); 1239 (americanum).

Ortiz, E. 1728 (cochabambense).

Ortiz, E.M. 1215 (americanum); 1239 (americanum).

Ortiz, M. 1121 (americanum).

Ortiz, N. 29 (nigrescens).

Ortiz, P. 179 (interandinum).

Ortuño, T. 204 (tripartitum); 290 (cochabambense).

Osten, C. 4506 (sarrachoides); 5105 (tweedieanum); 9052 (pilcomayense); 13448 (salicifolium); 22526 (sarrachoides).

Ostenfeld, C.H. 5298 (chenopodioides).

Pabón, G. 282 (interandinum).

Pabst, G. 4251 (americanum).

Pachano, A. 169 (nitidibaccatum).

Paci, O. 151 (echegarayi); 841 (tweedieanum).

Paciornik, E.F. 414 (americanum).

Páez V, J.A. 13 (americanum).

Paixão, J.L. 1542 (americanum).

Palacio, M. 23 (macrotonum).

Palacios, M.A. 20Mz 313 (echegarayi); 749 (chenopodioides).

Palacios, R. 1811 (pilcomayense).

Paniagua Zambrana, N. 644 (americanum); 8364 (interandinum); 8584 (interandinum); 8926 (cochabambense).

Parada, G.A. 1004 (americanum); 1635 (americanum); 1652 (arenicola); 2501 (caesium); 2506 (arenicola).

Parada-Gutierrez, G.A. 1635 (americanum).

Paredes, P. 145 (interandinum).

Paredes, S. 23 (macrotonum).

Parodi, L.R. 11130 (pilcomayense); 11875 (furcatum).

Parra O, C. 632 (macrotonum).

Pastore, A.L. 1195 (pygmaeum).

Pastore, F. 85 (nitidibaccatum).

Pastore, G.J. 1185 (chenopodioides).

Patiño, D. 4 (americanum).

Patzi, C. 217 (alliariifolium); CP 225 (dianthum).

Patzlaff, R. 55 (americanum).

Paula, C.H.R. de 277 (americanum); 621 (americanum).

Paula-Souza, J. 5884 (paucidens); 6076 (americanum); 7104 (americanum); 7178 (americanum); 7538 (tripartitum); 7557 (fiebrigii); 7685 (sinuatirecurvum); 7892 (tweedieanum); 7894 (tweedieanum); 7902 (tweedieanum); 7912 (tiinae); 8163 (pilcomayense).

Paz, S.M. 116 (interandinum).

Pedersen, C. 1586 (gonocladum).

Pedersen, E. 233 (physalifolium).

Pedersen, T.M. 321 (nitidibaccatum); 385 (americanum); 1054 (pygmaeum); 2767 (pygmaeum); 3079 (americanum); 4644 (pilcomayense); 5508 (pilcomayense); 5841 (pilcomayense); 6065 (pygmaeum); 6641 (pilcomayense); 7253 (sarrachoides); 8252 (chenopodioides); 8288 (tweedieanum); 10288 (pygmaeum); 10890 (americanum); 11828 (tweedieanum); 11851 (physalidicalyx); 12513 (pygmaeum); 12514 (chenopodioides); 12848 (caesium); 12893 (sarrachoides); 13330 (triflorum); 13868 (americanum); 14418 (triflorum); 14578 (pilcomayense); 15379 (nitidibaccatum); 16133 (aloysiifolium).

Pedra do Cavalo, G. 80 (americanum).

Pedraza F, M. 10 (nigrescens).

Pedraza, P. 1535 (cochabambense).

Pedraza-Peñalosa, P. 2550 (nigrescens).

Peláez, C. 99 (physalidicalyx); 205 (chenopodioides); 238 (tripartitum); 239 (palitans).

Pellegrini, M.O.O. 273 (enantiophyllanthum).

Pelosi, E. 44 (pygmaeum); 60 (pygmaeum).

Peña-Chocarro, M.C. 1486 (pilcomayense); 1489 (chenopodioides); 1529 (chenopodioides).

Peñafiel, M. 83 (interandinum); 368 (interandinum).

Peñaloza J, G. 27 (interandinum).

Peñaranda, J.A. 275 (glandulosipilosum); 456 (aloysiifolium); 932 (woodii).

Penland, C.W. 470 (interandinum).

Pennell, F.W. 2350 (interandinum); 3388 (nigrescens); 6611 (macrotonum); 6955 (macrotonum); 12663 (furcatum); 13048 (arequipense); 13464 (interandinum); 13575 (cochabambense); 13631 (polytrichostylum); 13689 (pentlandii); 13795 (pallidum); 13824 (dianthum); 13949 (longifilamentum); 13950 (pallidum); 14077 (grandidentatum); 14147 (pallidum); 14261 (tripartitum); 14460 (americanum); 14538 (arequipense); 14708 (interandinum).

Pennington, M.S. 71 (pilcomayense).

Pensiero, J.F. 2640 (pilcomayense); 2741 (americanum); 4167 (aloysiifolium); 4169 (aloysiifolium); 4232 (aloysiifolium); 4347 (aloysiifolium); .4521 (aloysiifolium); 4621 (aloysiifolium); 7341 (tweedieanum); 7500 (tweedieanum).

Pentland, J.B. 3 (gonocladum).

Percyner, ? 65 (sinuatirecurvum).

Perdomo, R. 22 (americanum).

Perea, J. 3413 (americanum).

Peredo, I. 182 (woodii); 432 (woodii); 442 (woodii).

Pereira, E. 358 (pilcomayense); 1810 (americanum); 4098 (americanum).

Pereira, S.C. 1511A (americanum); 1511 (paucidens).

Pérez Arbeláez, E. 88 (interandinum); 6002 (interandinum); 6336 (americanum).

Pérez Moreau, R.L. 3058 (triflorum); 3715 (pygmaeum); 4382 (tweedieanum); 4918 (pilcomayense); 45453 (furcatum).

Pérez, A.J. 8163 (macrotonum); 8281 (macrotonum).

Pérez, B. 19 (americanum); 86 (americanum); 207 (sarrachoides); 263 (americanum); 402 (nitidibaccatum); 675 (americanum).

Pérez, K. 27 (interandinum).

Pérez, L. 196 (americanum).

Pestalozzi, H.U. 989 (subtusviolaceum).

Petenatti, E.M. 323 (physalidicalyx).

Petersen, E. 20 (salicifolium); 561 (aloysiifolium); 1952 (fiebrigii).

Petetin, C.A. 1331 (pilcomayense); 1336 (pilcomayense); 1575 (americanum).

Pettersson, U. 239 (grandidentatum).

Pflanz, K. 69 (gonocladum); 145 (weddellii); 387 (tripartitum); 2066 (caesium).

Phadenhauer, J 324 (chenopodioides).

Philcox, D. 3986 (americanum); 4647 (americanum).

Philippi, F. 742 (furcatum).

Philippi, R.A. 25 (weddellii); 278 (furcatum).

Piccinini, B.G. 1888 (triflorum); 2000 (nitidibaccatum); 2016 (tweedieanum); 2048 (triflorum); 2104 (triflorum); 2122 (triflorum); 2448 (pilcomayense); 3270 (tweedieanum); 3275 (tweedieanum); 3430 (americanum).

Pierotti, S.A. 4091 (pilcomayense); 5083 (tweedieanum); 98932 (tweedieanum).

Pietrellini, F. 285 (arequipense); 286 (arequipense).

Pinto, G.C.P. 186 (americanum); 196/84 (americanum); 0275 (americanum).

Pipoly, J.J. 6509 (macrotonum); 7319 (americanum).

Pire, S.M. 350 (pilcomayense); 2054 (chenopodioides).

Pires, J.M. 9223 (americanum).

Pittier, H.F. 313 (macrotonum); 6087 (americanum); 6092 (nigrescens); 6253 (macrotonum); 6333 (nigrescens); 8281 (nigrescens); 9948 (nigrescens); 10453 (macrotonum); 10453 (macrotonum); 11634 (nigrescens); 11790 (nigrescens); 11793 (nigrescens); 12887 (macrotonum); 12902 (interandinum); 13172 (interandinum); 13201 (interandinum); 13544 (nigrescens); 14681 (nigrescens).

Pivetta, J [Father] 930 (americanum); 943 (paucidens).

Pizziolo, W. 47 (americanum).

Plaumann, F. 503 (paucidens).

Plowman, T.C. 2206 (interandinum); 2403 (americanum); 2621 (furcatum); 2622 (furcatum); 2861 (paucidens); 3858 (interandinum); 3871 (macrotonum); 4346 (macrotonum); 4447 (interandinum); 4636 (cochabambense); 5135 (interandinum); 5513 (americanum); 8013 (macrotonum); 8395 (americanum); 8930 (americanum); 10981 (americanum); 11141 (antisuyo).

Poeppig, E.F. 156 (furcatum); 538 (nitidibaccatum); 1366 (pseudoamericanum); 1366 (americanum).

Pohl, J.B.E. 600 (americanum); 2393 (caatingae); 2489 (americanum); 5408 (americanum).

Poloni, M. 1789 (paucidens); 1827 (paucidens).

Pombal, E.C. 26510 (chenopodioides).

Ponce, M. 22 (interandinum).

Ponce, M.M. 89 (aloysiifolium).

Pontual, I. 396 (enantiophyllanthum); 428 (enantiophyllanthum).

Popovkin, A.V. 317 (americanum).

Porto, M.L. 2871 (americanum).

Pott, A. 3728 (pilcomayense); 4757 (pilcomayense); 5421 (pilcomayense).

Pott, V.J. 4287 (pilcomayense).

Pozner, R. 523 (echegarayi); 584 (triflorum).

Prácticas de Recolección 32 (interandinum).

Prado, A.L. 2231 (pilcomayense).

Prado, D. 568 (chenopodioides); 629 (pygmaeum); 684 (chenopodioides); 716 (pilcomayense); 736 (pilcomayense); 743 (pilcomayense); 745 (pilcomayense).

Prado, M. 34 (americanum).

Prance, G.T. 6261 (americanum); 6797 (americanum); 10131 (americanum); 26084 (pilcomayense); 58736 (americanum).

Prata, A.P. 2388 (americanum); 2593 (americanum).

Prévost, M.F. 3911 (americanum).

Prina, A.O. 1645 (tweedieanum); 1854 (triflorum).

Pring, G.H. 105 (interandinum).

Proaño, E. 18 (interandinum).

Pujupet, J. 1047 (americanum).

Putcher, J. 55 (interandinum); 128 (interandinum); 251 (interandinum).

Puttemanns, A. CGG-5883 (paucidens).

Quarín, C. 357 (americanum); 1129 (pilcomayense); 1148 (americanum); 1427 (pygmaeum); 2369 (americanum); 2561 (pilcomayense).

Queiroz, E.P. 1517 (americanum); 2161 (americanum).

Queiroz, L.P. 13449 (tripartitum).

Quevedo G, E. 33 (americanum).

Quevedo, F.L. 1794 (americanum).

Quinn, V. 157 (cochabambense).

Quintana, C. 1072 (radicans); 1333 (interandinum).

Quintero 29 (aloysiifolium).

Quipuscoa S, V. 513 (interandinum); 923 (interandinum); 1583 (arequipense); 1737 (fragile); 2868 (fragile).

Quiroz, S.L. 3734 (juninense); 3797 (radicans); 3840 (corymbosum).

Raes, N. 59 (americanum).

Ragonese, A.E. [A.M.] 8251 (tweedieanum); 8715 (triflorum); 8721 (tweedieanum); 8750 (triflorum); 8751 (triflorum); 8785 (triflorum); 8794 (triflorum); 8809 (triflorum); 8818 (tweedieanum); 9017 (triflorum); 9449 (pygmaeum).

Rahn, K. 4817 (tweedieanum).

Raimondi, A. 11929 (radicans).

Rambo, B. 29020 (americanum); 31281 (paucidens); 39916 (americanum); 42637 (americanum); 43100 (americanum); 45745 (chenopodioides); 46421 (paucidens); 47208 (americanum); 52112 (paucidens).

Ramírez P, R.R. 13078 (nigrescens).

Ramírez, G. 20 (macrotonum).

Ramírez, M.C. 52 (tripartitum).

Ramírez, N. 993 (macrotonum); 2095 (nigrescens); 2670 (americanum).

Ramos, J.E. 442 (americanum); 5972 (macrotonum); 6139 (antisuyo); 6211 (macrotonum); 6447 (antisuyo); 6602 (macrotonum); 7304 (macrotonum); 7471 (macrotonum).

Ramos, M.E.M. 4822 (chenopodioides).

Raña, E. 149 (pygmaeum).

Rangel, O. 2496 (nigrescens); 11269 (nigrescens); 12351 (macrotonum); 12405 (nigrescens).

Ranta, P. 2077 (pilcomayense).

Rasp, A.E. 67 (triflorum).

Ratter, J.A. R.4262 (americanum).

Raute-Horscher, W. P-72 (arequipense).

Raynal, A. 18654 (americanum).

Reales, A. 1257 (tweedieanum); 1264 (marmoratum); 1719 (tweedieanum); 1773 (tweedieanum); 1906 (physalidicalyx); 1933 (tweedieanum).

Reed, E.C. 17 (interandinum).

Regnell, A.F. III-970 (americanum); III-970[b] (paucidens); III-970a (paucidens); III-971B (paucidens); III-971a (paucidens).

Rego, L.N.A.A. 6 (americanum).

Reiche, C.F. 69 (furcatum).

Reichlen, S. 10 (interandinum).

Reijenga, T.W. 995 (americanum).

Reina, G. 406 (macrotonum).

Reitz, R. [Padre] c72 (americanum); 464 (americanum); C693 (americanum); 871 (americanum); 2173 (americanum); 4424 (paucidens); 5077 (chenopodioides); 5597 (paucidens); 6066 (corymbosum); 6907 (americanum); 14800 (paucidens); 17346 (paucidens).

Renjifo, L.M. 227 (nigrescens).

Rentería, E. 714 (nigrescens); 1624 (americanum).

Renvoize, S.A. 3457 (aloysiifolium); 3477 (sinuatiexcisum); 3513 (sinuatirecurvum).

Resgate, E. 196 (americanum).

Revilla, J. 11 (americanum); 8667 (pilcomayense).

Reynel, C. 204 (pentlandii).

Ribas, O.S. 250 (paucidens); 6012 (paucidens); 6840B (americanum).

Ribeiro, B.G.S. 1391 (americanum).

Ribeiro, K.T. 236 (enantiophyllanthum); 237 (enantiophyllanthum); 238 (enantiophyllanthum).

Ricardi, M. 201 (grandidentatum); 5048 (furcatum).

Riccio, F 3712 (interandinum).

Richardson, A. 2059 (pentlandii).

Rico Arce, L. 1178 (aloysiifolium); 1217 (tripartitum); 1248 (palitans).

Rico, V.M. 352 (nigrescens); 355 (nigrescens).

Ridley, H.N. 77 (americanum).

Ridoutt, C.A. 11183a (corymbosum); 11520 (corymbosum).

Riedel, L. 402[b] (americanum); 506a (enantiophyllanthum); 7407 (paucidens).

Rilke, S. 561 (palitans).

Rimachi Y, M. 518 (americanum); 4134 (americanum); 7077 (americanum); 8340 (americanum); 8897 (americanum); 10277 (americanum); 10760 (americanum); 11116 (americanum); 11804 (americanum).

Rimbach, A. 657 (interandinum).

Ríos, J. 2723 (interandinum).

Risso, J. 964 (aloysiifolium).

Ritter, N. 532 (tripartitum); 644 (gonocladum); 703 (tripartitum); 766 (cochabambense); 815 (pallidum); 884 (cochabambense); 944 (subtusviolaceum); 967 (dianthum); 986 (subtusviolaceum); 1124 (americanum); 1231 (antisuyo); 1504 (leptocaulon); 1942 (physalifolium); 1967 (cochabambense); 3407 (dianthum); 4218 (arenicola); 4615 (pilcomayense).

Ritter, N.P. 1882 (tripartitum); 1965 (tripartitum).

Rivas T, G. 19 (antisuyo).

Rivera D, O. 1130 (interandinum); 3234 (nigrescens).

Rivera, D. 27 (aloysiifolium); DANA 28 (dianthum); 30 (aloysiifolium); DANA 36 (dianthum); DANA 56 (dianthum).

Robert, A. 760 (pilcomayense).

Robertson, K.R. 105 (corymbosum).

Robles, S.T. 584 (chenopodioides); 1860 (chenopodioides); 1898 (chenopodioides); 2096 (chenopodioides); 2139 (chenopodioides); 2277 (chenopodioides).

Rocabado, D. 128 (leptocaulon); 243 (cochabambense).

Rocha e Silva, I. 63 (americanum).

Rocha, E.A. 1722 (americanum).

Rocha, R. 1062 (aloysiifolium); 3668 (aloysiifolium).

Rodarte, A. 2 E (americanum).

Rodrigo, A.P. 492 (salicifolium).

Rodrigues V, ? 893 (tweedieanum).

Rodríguez C, A. 594 (nigrescens).

Rodríguez M, G.M. 317 (nigrescens); 962 (nigrescens).

Rodríguez P, D. 160 (interandinum).

Rodríguez R, E. 1909 (longifilamentum); 2307 (interandinum).

Rodríguez, A. 768 (pilcomayense).

Rodríguez, D. 14 (cochabambense); 71 (aloysiifolium); 372 (palitans); 419 (aloysiifolium); 1280 (tweedieanum); 1345 (palitans); 1391 (tweedieanum); 1421 (hunzikeri).

Rodríguez, E. 1909 (longifilamentum).

Rodríguez, F.M. 1280 (tweedieanum); 1391 (tweedieanum).

Rodríguez, M.E. 87 (americanum); 183 (americanum).

Rodríguez, R. 4 (americanum).

Rodschied, E.C. 31 (americanum).

Roig, F.A. 4969 (chenopodioides).

Roivainen, H. 75 (chenopodioides); 147 (chenopodioides); 2479 (triflorum); 2646 (triflorum); 2773 (triflorum); 2789 (furcatum).

Rojas M, M. 10 (americanum).

Rojas, R. 882 (americanum); 1793 (subtusviolaceum); 3268 (longifilamentum); 7335 (arenicola).

Rojas, T. 108[a] (pilcomayense); 108b (pilcomayense); 108c (pilcomayense); 108 d (pilcomayense); 193 (salicifolium); 1984 (pilcomayense); 2493 (sarrachoides); 5235 (michaelis); 5975 (americanum); 6165 (pilcomayense); 6953 (pilcomayense); 7289 (michaelis); 11322 (aloysiifolium).

Roldan, F.J. 1293 (americanum); 1704 (nigrescens); 2227 (macrotonum); 3276 (nigrescens).

Romanczuk, C. 37 (chenopodioides).

Romanos, A.E. 53 (arequipense).

Romanutti, A. 37 (americanum); 192 (salicifolium).

Rombouts, H.E. 718 (americanum).

Romero-Castañeda, R. 743 (americanum); 891 (macrotonum); 2474 (macrotonum); 6386 (americanum); 6389 (nigrescens); 6579 (nigrescens); 6856 (interandinum); 6930 (nigrescens); 7446 (macrotonum); 8937 (nigrescens); 8961 (macrotonum); 9172 (nigrescens); 10669 (nigrescens); 11066 (nigrescens); 11304 (nigrescens).

Rondeau, R. 134 (americanum).

Roque, J. 22 (americanum); 87 (americanum); 208 (americanum); 295 (cochabambense); 296 (corymbosum); 396 (americanum); 693 (americanum); 978 (arequipense); 985 (radicans); 1493[a] (grandidentatum); 1937 (arequipense); 4471 (cochabambense); 5484 (longifilamentum).

Rosa, N.A. 434 (americanum); 2189 (americanum); 3548 (americanum).

Rosário, C.S. 1878 (americanum).

Rosas, A. 30 (americanum); 310 (americanum).

Rose, J.N. 22532 (americanum).

Rosengurtt, B. B638 (chenopodioides); 775 (sarrachoides); B2642 (chenopodioides); 5783 (chenopodioides).

Rossato, M. 3339 (americanum); 3480 (americanum).

Rossoni, M.G. 133 (americanum); 633 (americanum).

Rossow, R.A. 2485 (triflorum); 3093 (nitidibaccatum); 4455 (triflorum); 5721 (chenopodioides).

Rotman, A.D. 373 (salicifolium); 774 (zuloagae); 803 (palitans); 899 (aloysiifolium); 933 (zuloagae).

Ruddle, K. 15 (nigrescens).

Ruiz Leal, R.A. 56 (echegarayi); 129 (echegarayi); 3322 (tweedieanum); 6420 (echegarayi); 9191 (marmoratum).

Ruiz, E. 1007 (furcatum).

Ruiz, T.V. 10633 (caesium).

Ruiz-Terán, L.E. 278 (interandinum); 511 (macrotonum); 1557 (macrotonum); 3545 (nigrescens); 7329 (macrotonum); 8545 (nigrescens); 11959 (americanum); 12390 (macrotonum); 14155 (macrotonum); 15502 (macrotonum).

Rusby, H.H. 24 (cochabambense); 779 (pallidum); 787 (pallidum); 790 (pallidum); 806 (gonocladum); 807 (tripartitum); 808 (palitans); 2564 (albescens).

Ruthsatz, B. 108 (weddellii); 1491 (tripartitum); 1961 (sinuatirecurvum); 2226 (sinuatirecurvum); 2422 (sinuatirecurvum); 2717 (weddellii); 3534 (weddellii); 3958 (tripartitum).

Rutile, A. 36 (chenopodioides); 65 (pygmaeum); 66 (americanum); 108 (chenopodioides); 111 (chenopodioides); 137 (chenopodioides); 218 (chenopodioides).

Sacco, J.C. 360 (paucidens).

Sagástegui, A. 7826 (interandinum); 7828 (corymbosum); 7854 (corymbosum); 7965 (interandinum); 8053 (interandinum); 8411 (interandinum); 9616 (cochabambense); 10363 (interandinum); 10712 (cochabambense); 10837 (cochabambense); 11416 (arequipense); 11556 (interandinum); 11607 (interandinum); 11946 (interandinum); 12289 (corymbosum); 12468 (grandidentatum); 12594 (interandinum); 14169 (arequipense); 14254a (interandinum); 14404 (interandinum); 14411 (interandinum); 14502 (polytrichostylum); 15668 (polytrichostylum); 16128 (americanum); 17235 (corymbosum); 17447 (interandinum).

Sagot, P.A. 453 (americanum); 493 (americanum).

Salama, A.M. 68 (nigrescens); 343 (macrotonum).

Salariato, D. 289 (echegarayi).

Saldías, E. 59 (chenopodioides).

Saldías, M. 172 (palitans).

Salgado, C. 14 (macrotonum).

Salgado, D.F. 551 (sinuatirecurvum); 570 (sinuatirecurvum).

Salians, I. 731 (subtusviolaceum).

Salinas R, N. 7257 (subtusviolaceum).

Salinas, I. 336 (longifilamentum); 1144 (longifilamentum).

Salm, H. 1 (americanum).

Salomón, L. 117 (salicifolium).

Salvador, J.L.G. 74 (chenopodioides).

Salzmann, P. 391 (americanum).

Sampaio, A.F. 8955 (americanum).

Sánchez Vega, I. 269 (interandinum); 3646 (radicans); 4600 (corymbosum); 4602 (grandidentatum); 4605 (radicans); 5472 (interandinum); 5827 (cochabambense); 10435 (cochabambense).

Sánchez, G. 73 (macrotonum).

Sanchez, J.H. 116 (paucidens).

Sandeman, C. 3519 (pseudoamericanum); 3755 (radicans); 3781 (arequipense); 4074 (interandinum); 4626 (cochabambense); 4655 (arequipense); 5343 (radicans); 5361 (corymbosum).

Sandoya-Sanchez, C.V. 563 (macrotonum).

Santos, L.A.S. 310 (americanum).

Santos, T.S. dos 3379 (americanum).

Sanzin, R. 176 (triflorum).

Saravia Toledo, C. 42 (physalidicalyx); 257 (nigrescens); 836 (physalidicalyx); 890 (physalidicalyx); 908 (nigrescens); 1019 (physalidicalyx); 1249 (macrotonum); 1375 (physalidicalyx); 1435 (nigrescens); 1883 (nigrescens); 1960 (aloysiifolium); 2000 (tweedieanum); 2398 (americanum); 2562 (interandinum); 3018 (interandinum); 3428 (americanum); 3692 (nigrescens); 11012 (aloysiifolium); 11099 (aloysiifolium); 11605 (aloysiifolium); 11868 (tripartitum); 12943 (tweedieanum); 12973 (tweedieanum); 13058 (physalidicalyx); 13320 (aloysiifolium); 13320 (aloysiifolium); 13628 (tweedieanum).

Sargent, C.S. 35 (pentlandii).

Särkinen, T.E. 4000 (cochabambense); 4001 (pentlandii); 4002 (cochabambense); 4003 (grandidentatum); 4004 (cochabambense); 4005 (cochabambense); 4007 (pentlandii); 4008 (radicans); 4010 (pallidum); 4011 (pallidum); 4014 (pallidum); 4015 (americanum); 4017 (americanum); 4018 (americanum); 4023 (americanum); 4027 (americanum); 4029 (longifilamentum); 4030 (longifilamentum); 4031 (pallidum); 4033 (americanum); 4034 (pallidum); 4035 (antisuyo); 4036 (cochabambense); 4038 (weddellii); 4040 (cochabambense); 4041 (americanum); 4042 (pallidum); 4045 (pallidum); 4047 (pallidum); 4048 (antisuyo); 4049 (antisuyo); 4050 (pallidum); 4051 (pallidum); 4053 (antisuyo); 4054 (cochabambense); 4055 (cochabambense); 4057 (pentlandii); 4058 (fragile); 4059 (fragile); 4065 (radicans); 4066 (corymbosum); 4071 (radicans); 4072 (americanum); 4075 (corymbosum); 4076 (nitidibaccatum); 4077 (radicans); 4078 (corymbosum); 4080 (americanum); 4081 (arequipense); 4082 (arequipense); 4083 (arequipense); 4084 (arequipense); 4085 (nitidibaccatum); 4087 (radicans); 4089 (corymbosum); 4090 (arequipense); 4095 (arequipense); 4097 (radicans); 4099 (arequipense); 4104 (fragile); 4105 (fragile); 4106 (pentlandii); 4107 (grandidentatum); 4108 (gonocladum); 4111 (grandidentatum); 4112 (radicans); 4113 (americanum); 4116 (americanum); 4117 (chenopodioides); 4505 (americanum); 4509 (corymbosum); 4511 (americanum); 4514 (americanum); 4520 (americanum); 4522 (interandinum); 4528 (americanum); 4534 (americanum); 4535 (pseudoamericanum); 4537 (arequipense); 4543 (americanum); 4555 (americanum); 4574 (americanum); 4575 (longifilamentum); 4577 (longifilamentum); 4579 (americanum); 4581 (longifilamentum); 4585 (longifilamentum); 4591 (cochabambense); 4598 (cochabambense); 4604B (corymbosum); 4607 (cochabambense); 4611 (longifilamentum); 4618 (cochabambense); 4619 (americanum); 4620 (longifilamentum); 4622 (americanum); 4624 (pseudoamericanum); 4627 (interandinum); 4632 (americanum); 4637 (cochabambense); 4640 (pseudoamericanum); 4643 (cochabambense); 4649 (americanum); 4650 (cochabambense); 4667 (grandidentatum); 4669 (radicans); 4670 (pseudoamericanum); 4676 (cochabambense); 4678 (pseudoamericanum); 4686 (pseudoamericanum); 4687 (corymbosum); 4692 (interandinum); 4693 (interandinum); 4695 (interandinum); 4698 (interandinum); 4699 (grandidentatum); 4704 (gonocladum); 4714 (grandidentatum); 4719 (interandinum); 4720 (interandinum); 4730 (pseudoamericanum); 4731 (interandinum); 4734 (interandinum); 4737 (pseudoamericanum); 4743 (grandidentatum); 4746 (interandinum); 4754 (juninense); 4757 (polytrichostylum); 4758 (interandinum); 4762 (interandinum); 4768 (juninense); 4769 (juninense); 4774 (interandinum); 4778 (pseudoamericanum); 4779 (radicans); 4780 (pseudoamericanum); 4781 (grandidentatum); 4791 (pseudoamericanum); 4792 (corymbosum); 4793 (corymbosum); 4794 (pseudoamericanum); 4795 (corymbosum); 4802 (corymbosum); 4804 (cochabambense); 4818 (americanum); 4820 (longifilamentum); 4823 (americanum); 4831 (arenicola); 4834 (longifilamentum); 4839 (polytrichostylum); 4841 (grandidentatum); 4842 (cochabambense); 4856 (longifilamentum); 4862 (americanum); 4866 (arenicola); 4867 (americanum); 4873 (arequipense); 5277 (polytrichostylum); 5284 (pallidum); 5285 (sinuatiexcisum); 5286 (polytrichostylum); 5287 (radicans); 5296 (longifilamentum); 5301 (pallidum); IM-5307 (pallidum); 5311 (radicans); 5312 (pentlandii); IM-5321 (pentlandii); 5323 (radicans); 5325 (grandidentatum); 5327 (arequipense); 5328 (cochabambense); 5332 (pentlandii); 5333 (pallidum); 5334 (gonocladum); IM-5339 (pallidum); 5341 (pentlandii); 5343 (pentlandii).

Sartori, M. 97 (chenopodioides).

Sás, B. 90 (cochabambense).

Sauerssig, D. 1909 (americanum).

Saunders, S.G.E. 335 (arequipense); 365 (arequipense); 990 (interandinum); 1297 (interandinum); 1382 (interandinum).

Savatier, L. 425 (grandidentatum); 506 (grandidentatum); 1760 (arequipense); 1779 (interandinum).

Say, A. 2506 (salicifolium).

Sayago, M. 103 (pygmaeum); 475b (tweedieanum); 476b (tweedieanum); 478b (tweedieanum); 547 (tweedieanum); 554 (palitans); 588 (palitans); 622 (tweedieanum); 637 (aloysiifolium); 796 (nitidibaccatum); 1252 (salicifolium); 1503 (salicifolium); 2000 (tweedieanum); 2010 (tweedieanum); 2054 (physalidicalyx); 2114 (tweedieanum); 2128 (pygmaeum).

Sayre, M. 20 (corymbosum).

Scala, A.C. 118 (americanum).

Scaldaferro, M. 55 (cochabambense).

Scarda, F.M. 27 (americanum).

Scatigna, A.V. 863 (americanum).

Schaller, G. 277 (pilcomayense).

Schiavone, M.M. 11655 (aloysiifolium); 11837 (zuloagae).

Schickendantz, F. 8 (tweedieanum); 20 (tweedieanum); 45 (salicifolium); 91 (salicifolium); 93 (tweedieanum); 111 (tweedieanum); 113 (marmoratum); 124 (tweedieanum); 135 (aloysiifolium); 139 (tweedieanum); 145 (tweedieanum); 214 (salicifolium); 329 (salicifolium).

Schiefer, H. 553 (interandinum).

Schimpff, H.J.F. 218 (americanum); 229 (interandinum); 658 (interandinum).

Schinini, A. 3971 (pilcomayense); 4745 (pilcomayense); 4746 (chenopodioides); 5174 (americanum); 5380 (pygmaeum); 5427 (americanum); 6420 (pygmaeum); 6806 (americanum); 6845 (americanum); 9387 (pilcomayense); 9633 (pilcomayense); 10021 (pilcomayense); 10125 (fiebrigii); 10196 (profusum); 10254 (zuloagae); 10256 (zuloagae); 10257 (zuloagae); 10258 (aloysiifolium); 11010 (pilcomayense); 11102 (americanum); 11276 (americanum); 16377 (sarrachoides); 17370 (americanum); 18656 (pygmaeum); 19013 (pygmaeum); 22275 (zuloagae); 22295 (zuloagae); 22536 (sinuatirecurvum); 24241 (pilcomayense); 25849 (pilcomayense); 26293 (pilcomayense); 26691 (pilcomayense); 30532 (pilcomayense); 34657 (glandulosipilosum); 34690 (glandulosipilosum); 34720 (tweedieanum); 36471 (pilcomayense).

Schlim, L.J. 194 (americanum).

Schmitt, G. 27 (rhizomatum); 27A (rhizomatum); 155 (pilcomayense); 163 (americanum).

Schmitt, J.P. 140 (longifilamentum); 268 (pallidum); 277 (pallidum).

Schnell, C.E. 8293 (paucidens).

Schnetter, M.L. 162 (nigrescens).

Schott, H.W. 5410 (americanum).

Schreiter, R. 761 (physalidicalyx); 1062 (aloysiifolium); 1214 (riojense); 1503 (fiebrigii); 1507 (fiebrigii); 1928 (glandulosipilosum); 2419 (physalidicalyx); 3794 (glandulosipilosum); 4828 (annuum); 4935 (weddellii); 5429 (annuum); 5990 (echegarayi); 6031 (echegarayi); 6183 (salicifolium); 7410 (chenopodioides); 7415 (physalidicalyx); 9967 (aloysiifolium); 10595 (salicifolium); 11199 (sinuatirecurvum); 11202 (caesium); 11203 (tripartitum); 12143 (physalidicalyx); 35084 (salicifolium).

Schroeber, C. 59 (americanum).

Schulte, M. 14 (tripartitum).

Schultes, R.E. 27 (macrotonum); 4037 (interandinum); 5139 (americanum); 5700 (macrotonum); 7265 (macrotonum); 7266 (macrotonum); 7420 (interandinum); 7439 (interandinum); 7458 (interandinum); 7566 (macrotonum); 7582 (macrotonum); 7767 (macrotonum); 7911 (interandinum).

Schultz, A.R. 212 (americanum); 1071 (americanum); 4019 (americanum); 7802 (americanum).

Schulz, A.G. 5477 (glandulosipilosum); 5508 (caesium); 12124 (pilcomayense); 17535 (pilcomayense).

Schunke Vigo, J. 1448 (americanum); 3668 (juninense); 3799 (fragile); 4958 (americanum); 6077 (americanum); 6992 (americanum).

Schwabe, H. 388 (nitidibaccatum).

Schwacke, A. 3810 (paucidens).

Schwarz, G.J. 1138 (americanum); 1212 (americanum); 1436 (paucidens); 4632 (americanum); 4697 (americanum); 6745 (paucidens); 8145 (americanum); 8455 (americanum); 8538 (pilcomayense); 8838 (chenopodioides); 8877 (americanum).

Schwindt, E. 1859 (americanum); 4696 (americanum); 4792 (americanum).

Scoffield, R.L. 105 (triflorum).

Scolnik, R. 21Tu-120 (fiebrigii); 464 (americanum); 474 (nigrescens); 598 (fiebrigii); 815 (americanum); 989 (americanum).

Scott-Elliot, G.F. 173 (furcatum); 416 (furcatum).

Scrivanti, L.R. 220 (pilcomayense); 385 (americanum).

Scur, L. 280 (paucidens); 962 (paucidens).

Sede, S. 514 (salicifolium).

Seger, G.D.S. 451 (chenopodioides).

Sehnem, A. 1392 (chenopodioides); 2070 (paucidens); 4163 (chenopodioides); 13986 (chenopodioides).

Seibert, R.J. 601 (nigrescens).

Seidel, R. 2717 (americanum); 6608 (arenicola).

Seidenschwarz, F.G. 11 /1 (americanum).

Seijo, G.J. 1079 (aloysiifolium); 1856 (salicifolium); 1869 (chenopodioides); 2039 (salicifolium); 2315 (triflorum); 4076 (weddellii); 4103 (weddellii).

Seillant, ? 27 (palitans).

Seler, C. 204 (arequipense).

Sellow, F. 225 (chenopodioides); 280 (chenopodioides).

Semper, J. 269 (salicifolium); 386 (tweedieanum); 397 (salicifolium); 15672 (tweedieanum); 16104 (tweedieanum).

Sendulsky, T. 472 (americanum).

Senn, H.A. 4068 (tripartitum).

Serrano, M. 1018 (zuloagae); 4634 (fiebrigii); 4692 (aloysiifolium); 4703 (aloysiifolium); 5099 (fiebrigii); 5283 (cochabambense); 5493 (fiebrigii); 5647 (aloysiifolium); 5921 (aloysiifolium); 6016 (zuloagae); 6121 (gilioides); 7127 (cochabambense).

Sérsic, A.N. 68 (echegarayi); 5040 (triflorum).

Shepard, R.S. 166 (tripartitum).

Shiki, D. 143 (americanum).

Silva Costa, D. 242 (americanum).

Silva Filho, F.A. 54 (paucidens).

Silva Filho, P.J.S. 716 (americanum); 1326 (chenopodioides).

Silva Neto, S.J. 981 (americanum); 1021 (americanum); 1734 (enantiophyllanthum); 1743 (enantiophyllanthum).

Silva, ? 60 (americanum).

Silva, A.C.C. 134 (americanum).

Silva, C.A.S. 18 (americanum).

Silva, E.M.G.B. 20 (americanum).

Silva, G.A. 452 (interandinum).

Silva, G.P. da 2259 (americanum).

Silva, J.M. 147 (americanum).

Silva, J.R. 4000 (paucidens).

Silva, J.S. 32 (americanum).

Silva, L.A.M. 3509 (americanum).

Silva, N.C.B. 47 (americanum).

Silva, S.J. 981 (americanum).

Silva, S.M. 1646 (americanum).

Silveira, G.H. 493 (americanum).

Silveira, M. 4176 (americanum).

Silveira, N. 8387 (americanum).

Silverstone Sopkin, P.A. 756 (nigrescens); 2041 (americanum); 3239 (americanum).

Simão Bianchini, R. 721 (enantiophyllanthum).

Simon, P.M. 165 (triflorum).

Simonis, J.E. 11 (pilcomayense); 125 (americanum).

Simpson, B.B. 8566 (arequipense).

Simpson, D.R. 439 (americanum).

Siñani, R. RS-288 (longifilamentum).

Sinca C, F. 47 (fragile).

Skottsberg, C. 198 (furcatum); 260 (furcatum); 363 (furcatum).

Slanis, A.C. 85 (tweedieanum); 139 (palitans); 169 (tweedieanum).

Sleumer, H.O. 8 (salicifolium); 410 /2 (weddellii); 505 (salicifolium); 731 (glandulosipilosum); 732 (glandulosipilosum); 733 (glandulosipilosum); 736 (glandulosipilosum); 755 (glandulosipilosum); 1610 (hunzikeri); 1627 (aloysiifolium); 1628 (salicifolium); 1633 (salicifolium); 1634 (salicifolium); 1827 (salicifolium); 1828 (salicifolium); 1833 (salicifolium); 1834 (hunzikeri); 1835 (salicifolium); 1836 (salicifolium); 1837 (salicifolium); 1841 (annuum); 1992 (glandulosipilosum); 2087 (glandulosipilosum); 2091 (americanum); 2132 (hunzikeri); 2134 (salicifolium); 2166 (salicifolium); 2196 (aloysiifolium); 2198 (tweedieanum); 2199 (salicifolium); 2208 (salicifolium); 2259 (hunzikeri); 2281 (salicifolium); 2311 (hunzikeri); 2354 (tweedieanum); 2375 (salicifolium); 2399 (salicifolium); 2400 (tweedieanum); 2481 (salicifolium); 2483 (salicifolium); 2484 (salicifolium); 2487 (salicifolium); 2491 (aloysiifolium); 2492 (salicifolium); 2495 (salicifolium); 2519 (salicifolium); 2551 (aloysiifolium); 2650 (tiinae); 2685 (salicifolium); 2690 (hunzikeri); 2691 (hunzikeri); 2692 (hunzikeri); 2748 (weddellii); 2768 (zuloagae); 2769 (salicifolium); 2976 (aloysiifolium); 3019 (weddellii); 3033 (zuloagae); 3089 (tripartitum); 3214 (riojense); 3235 (sinuatirecurvum); 3520 (aloysiifolium); 3574 (weddellii); 3767 (aloysiifolium); 3803 (physalifolium); 3922 (glandulosipilosum); 3997 (salamancae); 4102 (weddellii); 4108 (sinuatirecurvum); 4110 (sinuatirecurvum).

Smith, C.E. 4912 (corymbosum).

Smith, D.N. 2182B (cochabambense); 3501 (interandinum); 5857 (longifilamentum); 5921 (cochabambense); 6253 (cochabambense); 6691 (americanum); 7158 (cochabambense); 8774 (interandinum); 8795 (interandinum); 8953 (interandinum); 9336 (interandinum); 9673 (interandinum); 10061 (interandinum); 10372 (interandinum); 10556 (interandinum); 10735 (interandinum); 10857 (cochabambense); 11693 (interandinum); 11929A (interandinum); 11929 (grandidentatum); 11997 (grandidentatum); 12135 (cochabambense); 12178 (interandinum).

Smith, E.E. S&S 85 (furcatum).

Smith, H.H. 1165 (nigrescens); 1167 (macrotonum); 1168 (nigrescens); 1170 (nigrescens).

Smith, L.B. 1745 (enantiophyllanthum); 9725 (americanum); 10517 (paucidens); 12338 (paucidens); 12573 (americanum); 12575 (paucidens); 12735 (paucidens); 12900 (paucidens); 13848 (chenopodioides).

Smith, N. B-19 (americanum).

Smith, S.D. 380 (tripartitum); 392 (tripartitum).

Smith, S.F. 743 (americanum); 1354 (arenicola).

Smith, S.G. 1141 (americanum).

Sneidern, K. von 298 (nigrescens); 4833 (americanum); 5641 (nigrescens).

Soares Nunes, ? 41 (enantiophyllanthum).

Soares, E.L.C. 244 (paucidens).

Sobral, M. 1780 (americanum); 2260 (chenopodioides); 2276 (chenopodioides); 2296 (chenopodioides); 2300 (chenopodioides); 2850 (paucidens); 2852 (paucidens); 2854 (paucidens); 2891 (paucidens); 3212 (pilcomayense); 3334 (americanum); 3941 (chenopodioides); 7621a (paucidens); 7666 (paucidens); 8917 (paucidens); 9418 (paucidens); 14254 (paucidens).

Sobrinho, F.A. 62 (americanum).

Sodiro, A. 11 (macrotonum); 114/ 12 (interandinum); 114/ 13 (grandidentatum).

Solano, F. 15 (macrotonum).

Solbrig, O.T. 202 (tweedieanum).

Solomon, J.C. 2801 (radicans); 2878 (fragile); 4037 (tweedieanum); 4039 (salicifolium); 4080 (salicifolium); 4099 (nitidibaccatum); 4120 (chenopodioides); 4665 (triflorum); 4802 (gonocladum); 4826 (polytrichostylum); 4890 (pallidum); 5981 (pallidum); 6043 (dianthum); 6628 (gonocladum); 6662 (gonocladum); 7175 (palitans); 7220 (polytrichostylum); 7297 (dianthum); 8349 (pallidum); 8587 (arenicola); 8629 (subtusviolaceum); 10132 (caesium); 10363 (glandulosipilosum); 10514 (fiebrigii); 10806 (longifilamentum); 10901 (cochabambense); 10973 (aloysiifolium); 11285 (aloysiifolium); 11374 (pallidum); 11764 (tripartitum); 11861 (pallidum); 11945 (cochabambense); 11963 (antisuyo); 12731 (polytrichostylum); 12754 (tripartitum); 12756 (polytrichostylum); 13043 (gonocladum); 13050 (polytrichostylum); 13054 (cochabambense); 13073 (sinuatiexcisum); 13125 (pallidum); 13273 (cochabambense); 13277 (grandidentatum); 13283 (cochabambense); 13296 (tripartitum); 13339 (polytrichostylum); 13351 (cochabambense); 13691 (dianthum); 13809 (palitans); 13854 (dianthum); 13907 (longifilamentum); 13967 (arenicola); 14504 (gonocladum); 15088 (cochabambense); 15090 (gonocladum); 15098 (gonocladum); 15152 (pallidum); 15176 (gonocladum); 15701 (fiebrigii); 15881 (tripartitum); 15884 (gonocladum); 15910 (tripartitum); 15925 (cochabambense); 15929 (cochabambense); 16151 (antisuyo); 16237 (pentlandii); 16326 (gonocladum); 16329 (gonocladum); 16345 (tripartitum); 16463 (juninense); 16651 (antisuyo); 17049 (arenicola); 17422 (pallidum); 17521 (pallidum); 17538 (sinuatiexcisum); 17784 (gonocladum); 17846 (longifilamentum); 17861 (pallidum); 17901 (cochabambense); 17946 (rhizomatum); 18185 (cochabambense); 18227 (dianthum); 18230 (antisuyo); 18272 (pallidum); 18434 (arenicola); 18869 (longifilamentum).

Soria, N. 1514 (americanum); 1816 (pilcomayense); 2671 (americanum).

Soriano, A. 617 (sinuatirecurvum); 680 (weddellii); 832 (nitidibaccatum); 953 (aloysiifolium); 984 (aloysiifolium); 1259 (triflorum); 2606 (triflorum); 2640 (triflorum); 3257 (triflorum).

Sota, A.V. de la 65 (pilcomayense); 1546 (salicifolium); 1559 (salicifolium); 2295 (tweedieanum); 3129 (tweedieanum); 3131 (aloysiifolium); 3310 (tweedieanum); 4577 (americanum).

Sota, E. de la 236 (tiinae); 974 (arenicola); 1105 (glandulosipilosum); 2906 (cochabambense); 3023 (glandulosipilosum); 3059 (aloysiifolium).

Soukup, J. 50 (pentlandii); 95 (cochabambense); 975 (arequipense); 1933 (interandinum); 2550 (americanum); 2701 (interandinum); 2703 (interandinum); 2829 (polytrichostylum); 2832 (grandidentatum); 3233 (interandinum); 3242 (interandinum); 3317 (interandinum); 3618 (polytrichostylum); 3653 (interandinum); 3661 (interandinum); 3953 (arequipense); 4220 (interandinum); 4553 (interandinum); 4589 (corymbosum); 4611 (pseudoamericanum); 4616 (americanum); 4618 (fragile); 4622 (arequipense); 5544 (juninense); 5611 (interandinum); 6092 (juninense).

Souza, G.R. 1686 (americanum).

Souza, J.P. 1011 (paucidens).

Souza, L.O.F. de 102 (americanum).

Souza, M.A.D. de 848 (americanum).

Souza, R.S. 470 (americanum).

Souza, V.C. 3289 (americanum); 4305 (americanum); 11427 (chenopodioides); 12207 (paucidens); 12295 (americanum); 32004 (americanum).

Sparre, B. 733 (aloysiifolium); 756 (pilcomayense); 3696 (tweedieanum); 5302 (pygmaeum); 5472 (chenopodioides); 9232 (tiinae); 9233 (tiinae); 9594 (tweedieanum); 13011 (americanum); 13014 (americanum); 13212 (americanum); 13218 (americanum); 13264 (interandinum); 13328 (interandinum); 13389 (interandinum); 13417 (interandinum); 13420 (interandinum); 13984 (interandinum); 14268 (radicans); 14319 (interandinum); 14321 (interandinum); 14622 (interandinum); 14930 (macrotonum); 14934 (macrotonum); 15262 (americanum); 15423 (americanum); 15561 (americanum); 15562 (americanum); 15652 (interandinum); 15814 (interandinum); 15947 (macrotonum); 15996 (interandinum); 16900 (macrotonum); 16902 (macrotonum); 16980 (macrotonum); 16986 (macrotonum); 17059 (interandinum); 17375 (macrotonum); 17442 (interandinum); 17695 (macrotonum); 17697 (macrotonum); 17726 (interandinum); 17945 (americanum); 17994 (americanum); 18107 (americanum); 18112 (americanum); 18401 (interandinum); 18407 (interandinum); 18765 (antisuyo); 18949 (macrotonum); 19055 (americanum); 19454 (americanum); 19816 (americanum); 19833 (americanum).

Spegazzini, A. 50 (pygmaeum).

Spegazzini, C.L. 141 (chenopodioides); BAB-15689 (aloysiifolium).

Spegazzin, R.A. BAB-56646 (nitidibaccatum).

Sperling, C.R. 5653 (americanum).

Spichiger, R. 2105 (pilcomayense); 2394 (pilcomayense).

Spósito, T.C. 26367 (chenopodioides).

Spruce, R. 3983 (americanum); 3984 (americanum); 5819 (grandidentatum).

Stachino, M.S. 48 (aloysiifolium).

Stafford, D. 455 (weddellii); 815 (fragile).

Stehmann, J.R. 180 (americanum); 192 (americanum); 270 (americanum); 271 (chenopodioides); 384 (chenopodioides); 915 (americanum); 1522 (paucidens); 1553 (americanum); 1616 (chenopodioides); 1734 (paucidens); 1794 (paucidens); 1978 (americanum); 2129 (americanum); 3418 (enantiophyllanthum); 4274 (paucidens); 4329 (paucidens); 4384 (paucidens); 4414 (americanum); 4843 (paucidens); 6162 (americanum); 6340 (americanum).

Steibel, P.E. 2057 (tweedieanum); 2061 (triflorum); 3289 (chenopodioides); 3489 (tweedieanum); 3501 (triflorum); 3819 (tweedieanum); 3991 (salicifolium); 4017 (tweedieanum); 4098 (chenopodioides); 4619 (tweedieanum); 4671 (triflorum); 5474 (triflorum); 5478 (tweedieanum); 6765 (tweedieanum); 7509 (chenopodioides); 7701 (chenopodioides); 7702 (chenopodioides); 7735 (chenopodioides); 7946 (chenopodioides); 7959 bis (tweedieanum); 7960 (marmoratum); 7963 (marmoratum); 7976 (chenopodioides); 8003 (chenopodioides); 8004 bis (chenopodioides); 8011 (tweedieanum); 8035 (marmoratum); 8042 (tweedieanum); 8662 (pygmaeum); 8676 (pygmaeum); 9552 (chenopodioides); 10111 (marmoratum); 10118 (marmoratum).

Steinbach, J. 5750 (subtusviolaceum); 5840 (sinuatiexcisum); 5884 (leptocaulon); 5952 (albescens); 5972 (palitans); 8026 (leptocaulon); 8388 (dianthum); 8432 (aloysiifolium); 8525 (alliariifolium); 8526 (alliariifolium); 8546 (longifilamentum); 8605 (fiebrigii); 8614 (palitans); 8661 (palitans); 8707 (fiebrigii); 8763 (cochabambense); 8790 (palitans); 8803 (fiebrigii); 9201 (sinuatiexcisum); 9717 (tripartitum); 14844 (arenicola).

Steinbach, R.F. 12 (cochabambense); 32 (cochabambense); 34 (cochabambense); 121 (fiebrigii); 124 (palitans); 231 (dianthum); 529 (longifilamentum); 579 (antisuyo); 648 (leptocaulon); 659 (physalifolium); 721 (rhizomatum).

Stellfeld, C. 1065 (americanum); 1202 (americanum).

Stergios, B. 3723 (nigrescens); 20559 (macrotonum).

Stern, S. 88 (americanum); 98 (corymbosum); 104 (interandinum); 110 (interandinum); 117 (cochabambense); 124 (cochabambense); 140 (cochabambense); 301 (americanum); 343 (interandinum).

Stevens, W.D. 21955 (interandinum); 22067 (radicans).

Stevenson, P. 1940 (americanum).

Stewart, A. 3406 (americanum); 3407 (americanum); 3409 (americanum).

Steyermark, J.A. 13206 (macrotonum); 35953 (macrotonum); 55529 (macrotonum); 88968 (americanum); 96948 (macrotonum); 96970 (macrotonum); 104828 (macrotonum); 110480 (americanum); 118311 (nigrescens); 118569 (nigrescens); 121839 (macrotonum); 122055 (nigrescens); 124737 (nigrescens); 125438 (macrotonum); 127922 (macrotonum); 130943 (americanum).

Stiefkens, L.B. 6 (chenopodioides).

Stiles, G. 750 (interandinum).

Stork, H.E. 9372 (radicans); 9898 (antisuyo); 10264 (cochabambense); 10584 (physalifolium).

Straube, F. 66 (americanum).

Straw, R.M. 2266 (fragile); 2315 (fragile); 2344 (fragile).

Stuckert, T. 36 (salicifolium); 40 (pygmaeum); 261 (salicifolium); 404 (pygmaeum); 578 (pygmaeum); 870 (pygmaeum); 1747 (salicifolium); 1798 (salicifolium); 2207 (salicifolium); 2762 (tweedieanum); 3690 (salicifolium); 4555 (salicifolium); 4713 (pygmaeum); 4743 (salicifolium); 4798 (pygmaeum); 5022 (triflorum); 5848 (salicifolium); 6232 (pilcomayense); 6977 (salicifolium); 7019 (salicifolium); 7029 (echegarayi); 7184 (salicifolium); 7725 (salicifolium); 8654 (salicifolium); 8669 (salicifolium); 8672 (salicifolium); 8770 (salicifolium); 8963 (salicifolium); 9039 (pygmaeum); 9257 (salicifolium); 9265 (tweedieanum); 10372 (salicifolium); 10698 (salicifolium); 10883 (salicifolium); 10947 (salicifolium); 12199 (salicifolium); 12272 (pygmaeum); 12322 (salicifolium); 12520 (salicifolium); 13199 (salicifolium); 13438 (pygmaeum); 14029 (tweedieanum); 15161 (pygmaeum); 15584 (pygmaeum); 17188 (tweedieanum); 17713 (salicifolium); 17713a (nitidibaccatum); 19589 (salicifolium); 20771 (salicifolium); 20970 (salicifolium); 20976 (salicifolium); 21228 (salicifolium); 23314 (tweedieanum); 23341 (pygmaeum); 23661 (chenopodioides); 23806 (chenopodioides); 24012 (salicifolium); 24020 (chenopodioides); 24046 (pygmaeum).

Suárez, M.C. 174 (nigrescens).

Subils, R. 35 (triflorum); 38 (triflorum); 49 (aloysiifolium); 50 (aloysiifolium); 90 (aloysiifolium); 270 (fiebrigii); 285 (tiinae); 568 (pygmaeum); 994 (pygmaeum); 1051 (chenopodioides); 1057 (chenopodioides); 1059 (chenopodioides); 1615 (pygmaeum); 1944 (americanum); 2105 (chenopodioides); 2110 (chenopodioides); 2333 (nitidibaccatum); 2615 (palitans); 2627 (tweedieanum); 2653 (aloysiifolium); 2663 (tweedieanum); 2665 (annuum); 2667 (annuum); 2670 (tiinae); 2674 (annuum); 2682 (annuum); 3041 (aloysiifolium); 3050 (physalidicalyx); 3349 (tweedieanum); 3382 (pygmaeum); 3398 (pygmaeum); 3434 (chenopodioides); 3478 (aloysiifolium); 3483 (aloysiifolium); 3496 bis (aloysiifolium); 3497 (aloysiifolium); 3499 (aloysiifolium); 3523 (cochabambense); 3609 (glandulosipilosum); 3673 (palitans); 3675 (tweedieanum); 3680 (aloysiifolium); 3695 (palitans); 3701 (tweedieanum); 3762 (tweedieanum); 3815 (chenopodioides); 3816 (chenopodioides); 3825 (pygmaeum); 3848 (glandulosipilosum); 3848 bis (aloysiifolium); 3860 (physalidicalyx); 3867 (zuloagae); 3916 (nitidibaccatum); 3937 (chenopodioides); 3941 (nitidibaccatum); 4024 (tweedieanum); 4059 (triflorum); 4076 (triflorum); 4108 (triflorum); 4111 (triflorum); 4113 (triflorum); 4116 (triflorum); 4122 (americanum); 4125 (americanum); 4136 (americanum); 4144 (americanum); 4163 (americanum); 4168 (americanum); 4269 (americanum); 4281 (americanum); 4283 (americanum); 4434 (aloysiifolium); 4585b (weddellii); 4668 (chenopodioides); 4690 (pilcomayense); 4691 (chenopodioides); 4692 (pilcomayense); 4693 (chenopodioides); 4695 (pilcomayense); 4696 (americanum).

Suclli, E. 885 (cochabambense); 1140 (americanum); 1345 (cochabambense); 1535 (polytrichostylum); 1556 (polytrichostylum); 2292 (cochabambense); 2727 (pallidum).

Sucre, D. 1976 (americanum); 4952 (americanum); 5161 (enantiophyllanthum); 6464 (americanum); 7842 (americanum); 9644 (americanum); 9680 (americanum).

Sugden, A.M. 170 (americanum).

Sugiyama, M. 293 (chenopodioides).

Sullivan, G.A. 964 (pseudoamericanum); 980 (radicans); 991 (interandinum).

Sydow, H. 54 (interandinum).

Taborda, I. 4 (americanum).

Tamashiro, J.Y. 143 (americanum); 146 (americanum); 1009 (americanum).

Tameirão Neto, E. 186 (chenopodioides).

Tangaru, ? 12 (americanum).

Taroda, N. 18536 (americanum).

Tate, G.H.H. 663 (pallidum).

Tatter, O. 4238 (tweedieanum).

Tavares, S. 167 (americanum).

Taylor, C. 11307 (weddellii).

Taylor, C.M. 33 (salicifolium); 10187 (furcatum); 11307 (weddellii).

Teixeira, L.O.A. 724 (americanum).

Teixeira, W.A. 25084 (americanum).

Tejada M, J. X-26 (interandinum).

Tejada, N. 14580 (salicifolium).

Tepe, E.J. 140 (cochabambense); 2225 (subtusviolaceum); 2272 (americanum); 2291 (subtusviolaceum); 2579 (macrotonum); 2929 (macrotonum); 2930 (interandinum); 3013 (interandinum); 3053 (macrotonum); 3067 (macrotonum); 3329 (longifilamentum); 3335 (interandinum); 3338 (antisuyo); 3350 (macrotonum); 3361 (longifilamentum).

Terán, J. 233 (dianthum); 502 (leptocaulon); 825 (dianthum); 916 (aloysiifolium); 1106 (antisuyo); 1132 (dianthum); 1220 (leptocaulon); 1227 (leptocaulon); 1282 (dianthum); 1364 (antisuyo); 1532 (cochabambense); 1540 (cochabambense); 1757 (antisuyo); 1796 (longifilamentum); 1827 (antisuyo); 1889 (longifilamentum); 2094 (antisuyo); 2192 (sinuatiexcisum); 3140 (aloysiifolium); 3453 (longifilamentum).

Tessmann, G. 1217 (americanum); 2759 (americanum).

Theissen, S.J. 408A (paucidens).

Thode, V. 91 (americanum); 131 (paucidens).

Thomas, M.B. MT-488 (americanum); MT 551 (americanum); MT 569 (americanum); MT-595 (americanum).

Thomas, P.I. 109 (sinuatirecurvum).

Thomas, S.M. 39/1 (pentlandii).

Timaná, M. 2008 (americanum); 2203 (americanum); 2214 (americanum); 2226 (americanum); 2678 (americanum); 3251 (americanum).

Tipaz, G. 1585 (macrotonum).

Tirel, C. 33 (salicifolium); 225 (salicifolium).

Todzia, C.A. 2604 (interandinum).

Tolaba, J.A. 1112 (tripartitum); 1127 (physalifolium); 2024 (aloysiifolium); 2330 (aloysiifolium); 2341 (aloysiifolium); 2351 (aloysiifolium); 2712 (zuloagae); 2713 (zuloagae); 2739 (aloysiifolium); 2850 (aloysiifolium); 2878 (zuloagae); 3161 (physalidicalyx); 4232 (physalidicalyx); 4233 (tweedieanum); 4234 (salamancae); 4235 (salamancae); 4236 (tweedieanum); 4237 (salamancae); 4238 (salamancae); 4239 (tweedieanum); 4240 (salamancae); 4261 (aloysiifolium); 4267 (aloysiifolium); 4270 (profusum); 4275 (aloysiifolium); 4288 (aloysiifolium); 4289 (aloysiifolium); 4323 (tweedieanum); 4340 (tweedieanum); 4341 (aloysiifolium); 4388 (tweedieanum); 4390 (physalidicalyx); 4493 (tweedieanum); 4619 (salamancae).

Toledo, J.M. 45 (pilcomayense); 292 (aloysiifolium); 293 (aloysiifolium).

Toro, R. 23 (interandinum).

Torreggiani, ? 13 (chenopodioides).

Torres R, J.H. 19 (macrotonum); 61 (macrotonum).

Torres, R.B. 344A (americanum).

Torres, Z. 19 (radicans).

Torrez, V. 545 (cochabambense); 559 (cochabambense).

Torrico C, L.S. 464 (tripartitum).

Tortosa, R.D. 37/1 (nitidibaccatum).

Toscani, H.L. 27 (physalidicalyx); 45 (tweedieanum); 50 (chenopodioides).

Tovar T, D. 5 (antisuyo).

Tovar, J.D. 4 (americanum).

Tovar, O. 100 (cochabambense); 196 (polytrichostylum); 254 (arequipense); 268 (arequipense); 635 (arequipense); 646 (arequipense); 1317 (pentlandii); 3125 (pentlandii); 3790 (juninense); 4281 (juninense); 4551 (juninense); 4901 (radicans); 5369 (weddellii); 7042 (radicans); 7780 (pentlandii).

Trancano, A. 3 (caatingae).

Treacy, J. 666 (interandinum); 728 (pentlandii).

Tressens, S.G. 6401 (americanum).

Treviño, I. 296 (cochabambense).

Triana M, L.A. 266 (americanum).

Triana, J.J. 3855 (americanum).

Troiani, H.O. 540 (salicifolium); 3431 (triflorum); 3586 (tweedieanum); 3648A (triflorum); 3855 (tweedieanum); 4688 (marmoratum); 6820 (marmoratum); 7516 (chenopodioides); 8077 (tweedieanum); 8175 (tweedieanum); 8564 (marmoratum); 8565 (tweedieanum); 8572 (tweedieanum); 9895 (tweedieanum).

Troncoso, N.S. 1312 (pygmaeum); 2261 (chenopodioides); 2288 (americanum).

Trujillo, B. 18996 (macrotonum).

Tsugaru, S. B-12 (americanum); B-2383 (americanum).

Tuomisto, H. 62 (americanum).

Tupayachi, A. 116 (cochabambense); 753 (cochabambense); 775 (cochabambense); 912 (cochabambense); 1201 (cochabambense); 3185 (gonocladum).

Türpe, A.M. 4954 (zuloagae); 5007 (zuloagae).

Tweedie, J. 34 (pygmaeum); 38 (chenopodioides); 39 (chenopodioides); 50 (palitans); 51 (pygmaeum); 432 (sarrachoides); 441 (palitans); 1229 (physalidicalyx).

Udulutsch, R.G. 196 (americanum).

Ugent, D. 687 (americanum); 3784 (pentlandii); 3799 (cochabambense); 3802 (grandidentatum); 3857 (interandinum); 3992 (pentlandii); 4402 (pallidum); 4404 (polytrichostylum); 4540 (interandinum); 4559 (dianthum); 4605 (cochabambense); 4623 (fiebrigii); 4651 (cochabambense); 5040 (dianthum); 5066 (leptocaulon); 5118 (aloysiifolium); 5302 (arequipense); 5303 (radicans); 5304 (corymbosum); 5325 (cochabambense); 5579 (interandinum).

Ule, E. 3746 (enantiophyllanthum); 4310 (chenopodioides).

Ulibarri, E.A. 457 (nitidibaccatum); 470 (tweedieanum).

Ungaretti, I. 545 (americanum).

Urdampilleta, J.D. 651 (nitidibaccatum); 671 (tweedieanum); 717 (palitans); 719 (aloysiifolium); 760 (tiinae).

Uribe Uribe, L. 5366 (interandinum).

Urrea, G. 37 (macrotonum).

Vaca, A.A. 6629 (pilcomayense).

Valdemarin, K.S. 686 (americanum).

Valduga, E. 406 (chenopodioides).

Vale, G.D. 101 (americanum).

Valencia, N. 051 (arequipense); 052 (arequipense); 623 (grandidentatum); 995 (grandidentatum).

Valenzuela, E. 87 (pentlandii); 154 (gonocladum); 173 (gonocladum); 180 (gonocladum); 213 (gonocladum); 340 (gonocladum).

Valenzuela, L. 236 (juninense); 248 (radicans); 332 (tripartitum); 468 (americanum); 786 (pallidum); 1218 (pallidum); 1437 (cochabambense); 1587 (pallidum); 1735 (antisuyo); 1741 (cochabambense); 2015[b[ (interandinum); 2015[a] (pentlandii); 2441 (cochabambense); 3690 (cochabambense); 3729 (cochabambense); 4532 (antisuyo); 4544 (pallidum); 4739 (cochabambense); 4909 (sinuatiexcisum); 4965 (antisuyo); 4990 (leptocaulon); 5148 (americanum); 5344 (cochabambense); 5665 (pallidum); 5894 (antisuyo); 5908 (antisuyo); 5933 (dianthum); 6010 (cochabambense); 6064 (cochabambense); 6091 (cochabambense); 6117 (cochabambense); 6180 (polytrichostylum); 6189 (radicans); 6199 (pentlandii); 6257 (cochabambense); 6399 (cochabambense); 6650 (americanum); 6785 (americanum); 7019 (pallidum); 7282 (antisuyo); 7284 (antisuyo); 8098 (cochabambense); 8209 (sinuatiexcisum); 8964 (cochabambense); 9676 (antisuyo); 10353 (longifilamentum); 12648 (americanum); 28615 (cochabambense); 28718 (cochabambense).

Válka, R.J. 1477 (americanum).

Valla, J.J. 26 (pilcomayense).

van der Werff, H. 7685 (macrotonum); 14657 (corymbosum); 14803 (interandinum); 15742 (americanum); 20483 (fragile); 20592 (fragile); 20801 (fragile); 20822 (radicans); 20880 (grandidentatum); 20953 (weddellii).

Vanegas, M. 1 (nigrescens).

Vanni, R. 679 (americanum); 804 (americanum); 1895 (tweedieanum); 1926 (tweedieanum); 2063 (tweedieanum); 2100 (tweedieanum); 2304 (sarrachoides); 4194 (aloysiifolium); 4211 (physalidicalyx); 4214 (aloysiifolium).

Varela, F.J. de 100 (tweedieanum); 386 (aloysiifolium); 633 (salicifolium); 687 (chenopodioides); 1269 (zuloagae); 1367 (sinuatirecurvum); 1652 (tweedieanum).

Vargas C, I.G. 85 (aloysiifolium); 145 (tripartitum); 490 (aloysiifolium); 753 (fiebrigii); 787 (alliariifolium); 855 (tripartitum); 2444 (palitans); 2444 (tripartitum); 2663 (americanum); 4559 (palitans); 4559 (palitans); 7039 (sinuatiexcisum).

Vargas L, J.H. 4821 (antisuyo).

Vargas, ? 63 (nigrescens).

Vargas, C. 205 (radicans); 673 (grandidentatum); 742 (cochabambense); 777 (cochabambense); 1966 (polytrichostylum); 4006 (fiebrigii); 4016 (fiebrigii); 5926 (fragile); 6185 (americanum); 7509 (pallidum); 8342 (pallidum); 8478 (radicans); 10748 (polytrichostylum); USM-14721 (arequipense); 14918 (pallidum).

Vargas, C.A. 2486 (macrotonum).

Vargas, E. 104 (radicans).

Vargas, H. 2316 (antisuyo); 4821 (macrotonum).

Vargas, J.M. 63 (nigrescens).

Vargas, L. 1266 (americanum).

Vargas, M. 31 (tripartitum); 104 (tripartitum).

Vargas, N. 273 (gonocladum).

Vásquez, R. 13379 (americanum); 20795 (americanum); 25177 (americanum); 26746 (longifilamentum); 27281 (longifilamentum); 29198 (cochabambense); 30036 (cochabambense); 30204 (longifilamentum); 31058 (subtusviolaceum); 32897 (antisuyo); 32900 (antisuyo); 33796 (juninense); 33798 (antisuyo); 35372 (subtusviolaceum); 35374 (subtusviolaceum); 35721 (longifilamentum).

Vattuone, I.C. 57 (tweedieanum); 58 (profusum); 61 (profusum); 183 (tripartitum); 184 (tripartitum).

Vega Ocaña, C. 341 (cochabambense).

Vega, C. 870[a] (chenopodioides); 870[b] (pygmaeum).

Vegetti, A.C. 746 (americanum).

Velarde Nuñez, O. 874 (corymbosum).

Velasco L, P. 384 (interandinum).

Velasco, V. 177 (nigrescens).

Vélez N, M.C. 743 (americanum).

Vélez, C. 6333 (americanum).

Vendruscolo, G.S. 474 (americanum); 487 (americanum); 497 (chenopodioides); 510 (paucidens); 517 (chenopodioides); 582 (chenopodioides); 596 (chenopodioides); 605 (chenopodioides); 607 (paucidens); 649 (chenopodioides); 672 (chenopodioides); 688 (paucidens); 721 (sarrachoides); 727 (chenopodioides); 731 (paucidens); 737 (chenopodioides); 773 (chenopodioides); 782 (chenopodioides).

Ventania, P. 870 (chenopodioides).

Venturi, S. 58b (fiebrigii); 58 (fiebrigii); 58a (fiebrigii); 64 (chenopodioides); 115 (aloysiifolium); 159 (palitans); 245 (glandulosipilosum); 266 (americanum); 350 (pygmaeum); 355 (physalidicalyx); 569 (tweedieanum); 604 (tweedieanum); 624 (pilcomayense); 683 (aloysiifolium); 819 (tweedieanum); 919 (aloysiifolium); 1090 (fiebrigii); 1142 (tweedieanum); 1195 (physalidicalyx); 1430 (fiebrigii); 2239 (palitans); 2241 (aloysiifolium); 2431 (tweedieanum); 2448 (glandulosipilosum); 2450 (glandulosipilosum); 3112 (weddellii); 3143 (cochabambense); 3855 (physalidicalyx); 4760 (cochabambense); 4938 (tripartitum); 4992 (physalidicalyx); 5005 (chenopodioides); 5009 (aloysiifolium); 5064 (zuloagae); 5256 (cochabambense); 5435 (caesium); 6068 (tweedieanum); 6994 (tweedieanum); 7100 (aloysiifolium); 7211 (huayavillense); 7776 (riojense); 7777 (salicifolium); 7997 (nitidibaccatum); 8024 (tweedieanum); 8138 (sinuatirecurvum); 8507 (annuum); 8622 (weddellii); 9012 (salicifolium); 9531 (huayavillense); 9533 (weddellii); 9951 (fiebrigii); 10389 (sinuatirecurvum); 10556 (aloysiifolium); 10625 (fiebrigii); 40368 (palitans).

Vera, M. 070 (pilcomayense).

Verdi, M. 2220 (paucidens).

Vergara, L.K. 84 (nigrescens).

Vervoorst, F. 3555 (physalifolium); 5723 (triflorum).

Viana, J.J. JPB 18720 (americanum).

Viani, R.A.G. 178 (paucidens).

Vianna, E.C. 11 (americanum); 18 (americanum); 146 (americanum).

Vianna, M.C. 192 (enantiophyllanthum).

Vico Gimena, R. 174 (aloysiifolium).

Vidal, M.R.R. 354 (americanum).

Vieira, R.F. 430 (americanum); 1676 (americanum).

Vignoli-Silva, M. 25 (paucidens); 91 (paucidens); 239 (americanum).

Viirsoo, E. c (tweedieanum); d (tweedieanum); 5 (tweedieanum); 7 (tweedieanum); 16 (salicifolium); 100[a] (salicifolium); 100[b] (tweedieanum); 102 (tweedieanum).

Vilca C, S. 71 (antisuyo); 254 (subtusviolaceum).

Vilcapoma, G. 42 (arequipense); 56 (corymbosum); 100 (antisuyo); 122 (interandinum); 150 (radicans); 157 (corymbosum); 283 (arequipense); 1649A (pseudoamericanum); 4150 (polytrichostylum); 5330 (pseudoamericanum); 5981 (arequipense).

Villa, E. 605 (glandulosipilosum).

Villacrés, P. 186 (interandinum).

Villafañe, A. 529 (salicifolium); 569 (tweedieanum); 666 (tweedieanum); 672 (salicifolium); 1277 (salamancae).

Villalobos, J. 426 (fiebrigii); 452A (aloysiifolium); 538 (cochabambense); 927 (alliariifolium); 1198 (fiebrigii); 1341 (fiebrigii); 1363 (huayavillense); 1430 (aloysiifolium); 1539 (aloysiifolium).

Villamil, C.B. 6705 (tweedieanum); 11706 (pygmaeum); 11955 (triflorum).

Villamil, E. 113 (nigrescens).

Villavicencio L, X. 318 (weddellii).

Villegas D, M. 71 (americanum).

Villegas, J.A. 30 (pentlandii).

Vink, R. 351 (interandinum).

Vogel, S. 402 (leptocaulon); 523 (dianthum).

Vogelmann, H.W. 2006 (macrotonum).

Volponi, C.R. 69 (salicifolium); 183 (echegarayi).

Von Platen, L. 149 (triflorum).

Vuilleumier, B.B.S. 334 (pallidum).

Wagenknecht, R. 18532 (furcatum).

Wall, E. 238 (pilcomayense).

Walter, B.M.T. 997 (americanum); 4143 (americanum).

Wasshausen, D.C. 786 (pallidum); 1948 (fiebrigii).

Wasum, R. 307 (paucidens); 507 (paucidens); 1084 (chenopodioides); 1941 (americanum); 3157 (americanum); 3993 (americanum); 4079 (chenopodioides); 4298 (sarrachoides); 7398 (chenopodioides); 10938 (paucidens).

Wawra, H. 2599 (americanum).

Weberbauer, A. 685 (pallidum); 2868 (grandidentatum); 6598 (juninense); 6789 (cochabambense); 7516a (juninense); 7517 (cochabambense).

Webster, G.L. 23162 (radicans); 31281 (interandinum).

Weigend, M. 2000/54 (fragile); 2000/54 (fragile); 2000/505 (grandidentatum); 2000/579 (arequipense); 97/925 (arequipense); 7228 (polytrichostylum).

Weinberg, B. 636 (americanum).

Wells, J.D. 1060 (pallidum).

Werdermann, E. 359 (furcatum).

Werling, L. 236 (macrotonum).

Wessels Boer, J.G. 1740 (macrotonum).

West, J. 3704 (polytrichostylum); 8028 (cochabambense); 8031 (cochabambense); 8170 (interandinum); 8216 (tripartitum); 8217 (sinuatirecurvum).

Weyland, M.C. 447 (americanum); 2040 (americanum).

Whalen, M.D. 853 (americanum); 881 (interandinum); 887 (cochabambense); 895 (americanum).

White, O.E. 165 (leptocaulon); 1091 (americanum).

White, S. 596 (interandinum); 731 (americanum).

Wickens, G.E. 11 (sinuatirecurvum).

Wilczek, E. 244 (tweedieanum); 241 (salicifolium).

Williams, D.E. 959 (americanum); 960 (arenicola).

Williams, L. 12825 (americanum).

Williams, R.S. 826 (polytrichostylum); 843 (albescens).

Williamson, J. 440 (triflorum); 2446 (chenopodioides).

Willian, J.G. 234 (aloysiifolium); 258[a] (americanum); 258[b] (aloysiifolium).

Windisch, P.G. 8670 (paucidens).

Without Collector 7 (triflorum); 11 (furcatum); 48 (echegarayi); 81 (aloysiifolium); 120 (pilcomayense); 128 (chenopodioides); 137 (triflorum); 172 (pygmaeum); 175 (gonocladum); 179 (chenopodioides); 184 (chenopodioides); 215 (pygmaeum); 269 (nitidibaccatum); 452 (leptocaulon); DM. 483 (furcatum); 520 (pygmaeum); 558 (sarrachoides); 848 (corymbosum); 2802 (cochabambense); 3431 (triflorum); 3584 (triflorum); 3648 (triflorum); 9097 (palitans); 11183 (pseudoamericanum); 12406 (cochabambense).

Wolstenholme, G.E. 31 (gonocladum); 33 (gonocladum).

Wolston, A. 856 (americanum).

Wood, J.R.I. 7591 (tripartitum); 7709 (aloysiifolium); 7771 (cochabambense); 7793 (gonocladum); 7810 (sinuatiexcisum); 7889 (sinuatirecurvum); 7954 (sinuatiexcisum); 8463 (aloysiifolium); 8652 (caesium); 8945 (cochabambense); 9248 (leptocaulon); 9317 (alliariifolium); 9756 (cochabambense); 9786 (fiebrigii); 9890 (zuloagae); 10688 (gonocladum); 10758 (weddellii); 11464 (dianthum); 11871 (physalifolium); 11974 (rhizomatum); 12710 (alliariifolium); 12842 (leptocaulon); 13577 (americanum); 14224 (caesium); 14390 (gonocladum); 15220 (antisuyo); 15303 (hunzikeri); 15515 (sinuatiexcisum); 15516 (polytrichostylum); 15542 (sinuatiexcisum); 15552 (cochabambense); 15615 (caesium); 15876 (huayavillense); 16818 (tweedieanum); 16901 (hunzikeri); 17025 (cochabambense); 17115 (physalifolium); 17116 (physalifolium); 17120 (aloysiifolium); 17194 (americanum); 17348 (arenicola); 17690 (rhizomatum); 17726 (tripartitum); 17868 (hunzikeri); 18072 (grandidentatum); 18597 (tweedieanum); 18672 (gonocladum); 18764 (tweedieanum); 18884 (aloysiifolium); 19040 (grandidentatum); 19041 (polytrichostylum); 19085 (aloysiifolium); 19114 (dianthum); 19134 (rhizomatum); 19209 (gilioides); 19282 (gonocladum); 19303 (physalifolium); 19333 (cochabambense); 19378 (sinuatiexcisum); 19440 (physalifolium); 19487 (sinuatiexcisum); 19562 (cochabambense); 19616 (woodii); 20104 (caesium); 20129 (caesium); 20266 (rhizomatum); 20974 (cochabambense); 21130 (hunzikeri); 21272 (sinuatiexcisum); 21590 (rhizomatum); 21662 (aloysiifolium); 21698 (americanum); 21774 (alliariifolium); 21787 (woodii); 21970 (palitans); 21975 (physalifolium); 22109 (zuloagae); 22168 (tripartitum); 22193 (cochabambense); 22206 (cochabambense); 22252 (aloysiifolium); 22341 (woodii); 22375 (aloysiifolium); 22394 (hunzikeri); 22538 (caesium); 22557 (cochabambense); 22595 (glandulosipilosum); 22612 (hunzikeri); 22613 (aloysiifolium); 22684 (aloysiifolium); 22689 (gilioides); 22690 (gilioides); 22719 (gilioides); 22778 (rhizomatum); 22788 (aloysiifolium); 22790 (woodii); 22840 (woodii); 22964 (dianthum); 27618 (tweedieanum); 28374 (woodii); 28434 (physalifolium).

Worth, C.R. 15704 (arequipense); 15743 (radicans); 16487 (furcatum).

Woytkowski, F. 153 (cochabambense); 154 (subtusviolaceum); 230 (polytrichostylum); 239 (pallidum); 241 (polytrichostylum); 296 (pallidum); 317 (pallidum); 323 (pallidum); 353 (grandidentatum); 525 (longifilamentum); 574 (pallidum); 576 (radicans); 722 (juninense); 728 (radicans); 789 (juninense); 1041 (juninense); 6758 (corymbosum); 7340 (subtusviolaceum); 7964 (cochabambense); 8152 (americanum).

Wullschlägel, H.R. 374 (americanum).

Wunderlin, R. 8688 (nigrescens).

Wurdack, J.J. 795 (juninense).

Xavier, F.C. 78 (americanum).

Xavier, L.P. JPB 887 (americanum); JPB 2117 (americanum); JPB 3611 (americanum).

Yarupaitán, G. 806 (arequipense).

Yepes A, S. 789 (macrotonum); 1118 (americanum).

Young, K. 583 (antisuyo); 1956 (juninense); 2012 (juninense); 2224 (interandinum); 4265 (longifilamentum).

Zabala, M. WT-19 (gonocladum).

Zabala, S. 18 (aloysiifolium); 174 (aloysiifolium).

Zachia, R.A. 176 (americanum); 3097 (americanum); 5726 (chenopodioides).

Zachia, R.A.

Zadro, ? 95 (aloysiifolium).

Zak, V. 1.419 (macrotonum); 661 (macrotonum); 859 (macrotonum); 1009 (macrotonum); 1065 (antisuyo); 1140 (macrotonum); 1375 (macrotonum); 1377 (macrotonum); 1378 (macrotonum); 1379 (macrotonum); 1394 (macrotonum); 1400 (macrotonum); 1417 (macrotonum); 1419 (antisuyo); 1421 (interandinum); 1466 (macrotonum); 1550 (macrotonum); 1551 (antisuyo); 1553 (macrotonum); 1555 (antisuyo); 1556 (macrotonum); 1557 (antisuyo); 1559 (macrotonum); 1562 (macrotonum); 1579 (macrotonum); 1581 (macrotonum); 1582 (antisuyo); 1584 (interandinum); 1588 (macrotonum); 1610 (interandinum); 1619 (macrotonum); 1632 (interandinum); 1633 (interandinum); 1638 (macrotonum); 1639 (radicans); 1647 (interandinum); 1736 (macrotonum); 1740 (macrotonum); 1765 (macrotonum); 1787 (interandinum); 1913 (macrotonum); 1914 (macrotonum); 1980 (americanum); 2018 (interandinum); 2077 (macrotonum); 2091 (antisuyo); 2097 (macrotonum); 2099 (interandinum); 2243 (interandinum); 2369 (interandinum); 2370 (interandinum); 2382 (interandinum); 2391 (interandinum); 2422 (interandinum); 2425 (interandinum); 2446 (longifilamentum); 2472 (interandinum); 2474 (macrotonum); 2483 (macrotonum); 2490 (macrotonum); 2499 (macrotonum); 2501 (macrotonum); 2724 (interandinum); 2731 (interandinum); 2742 (macrotonum); 2972 (interandinum); 2973 (interandinum); 2979 (interandinum); 3038 (macrotonum); 3536 (interandinum).

Zambrano, G. 66 (nigrescens).

Zanotti, C.A. 187 (tripartitum); 673 (sinuatirecurvum); 748 (echegarayi).

Zapata, M. 17447 (interandinum).

Zárate B, M. MZ 2248 (gonocladum).

Zardini, E.M. 131 (triflorum); 1849 (aloysiifolium); 1859 (tripartitum); 1960 (tripartitum); 2570 (pilcomayense); 2671 (pilcomayense); 2712 (americanum); 3385 (americanum); 3426 (americanum); 4974 (americanum); 5335 (americanum); 5701 (americanum); 5753 (americanum); 5756 (americanum); 5792 (americanum); 6642 (americanum); 6650 (americanum); 6983 (americanum); 11390 (americanum); 13465 (americanum); 13605 (americanum); 14630 (americanum); 15600 (americanum); 16042 (americanum); 16720 (pilcomayense); 20401 (pilcomayense); 20409 (pilcomayense); 20517 (americanum); 21222 (americanum); 22107 (americanum); 22242 (pilcomayense); 22282 (americanum); 22367 (pilcomayense); 22372 (pilcomayense); 22460 (pilcomayense); 22465 (americanum); 22745 (americanum); 22784 (pilcomayense); 22836 (pilcomayense); 23431 (pilcomayense); 23560 (pilcomayense); 23560 (pilcomayense); 28234 (americanum); 28350 (americanum); 30819 (paucidens); 31586 (pilcomayense); 31731 (pilcomayense); 32112 (americanum); 32116 (americanum); 32856 (americanum); 35178 (pilcomayense); 36913 (pilcomayense); 39346 (pilcomayense); 40428 (sarrachoides); 54796 (americanum); 55288 (americanum).

Zaremba, R. 188 (paucidens).

Zarucchi, J.L. 2623 (americanum); 4006 (americanum); 4198 (americanum); 5807 (nigrescens); 6886 (macrotonum); 6949 (macrotonum).

Zavala-Gallo, L. 180 (triflorum); 264 (aloysiifolium); 266 (tweedieanum); 269 (aloysiifolium); 273 (triflorum).

Zehutner, Dr. 169 (americanum).

Zenteno, F. 9852 (sinuatirecurvum); 10122 (sinuatirecurvum); 12091 (gilioides).

Zimmerer, K.S. 207 (pallidum).

Zöllner, O. 1353 (weddellii); 8950 (furcatum); 9593 (radicans); 14543 (furcatum).

Zuloaga, F.O. 225 (zuloagae); 252 (aloysiifolium); 388 (aloysiifolium); 393 (fiebrigii); 1107 (fiebrigii); 1594 (dianthum); 1619 (caesium); 1623 (aloysiifolium); 1732 (pallidum); 1948 (americanum); 1997 (americanum); 3475 (aloysiifolium); 3487 (aloysiifolium); 3587 (tripartitum); 3795 (aloysiifolium); 4258 (macrotonum); 4282 (macrotonum); 5577 (americanum); 5954 (sinuatirecurvum); 6011 (weddellii); 6653 (americanum); 6987 (americanum); 7464 (aloysiifolium); 7521 (caesium); 7541 (aloysiifolium); 7848 (fiebrigii); 7865 (aloysiifolium); 7915 (americanum); 8323 (americanum); 8493 (aloysiifolium); 8691 (fiebrigii); 9202 (salicifolium); 9219 (tripartitum); 9249 (salicifolium); 9314 (sinuatirecurvum); 9344 (riojense); 9356 (tripartitum); 9388 (palitans); 9502 (tweedieanum); 10110 (aloysiifolium); 10114 (cochabambense); 10128 (aloysiifolium); 10199b (weddellii); 10289 (aloysiifolium); 10411 (caesium); 10428 (cochabambense); 10506 (zuloagae); 10518 (aloysiifolium); 10633 (sinuatirecurvum); 10642 (salicifolium); 10812 (weddellii); 10829 (tripartitum); 10832 (palitans); 10936 (tripartitum); 11060 (aloysiifolium); 11134 (sinuatirecurvum); 11162 (sinuatirecurvum); 11256 (tiinae); 11260 (cochabambense); 11463 (huayavillense); 11551 (aloysiifolium); 11659 (aloysiifolium); 11661 (aloysiifolium); 11669 (aloysiifolium); 11695 (fiebrigii); 11808 (sinuatirecurvum); 11919 (tweedieanum); 12045a (salicifolium); 12067 (salicifolium); 12125 (tweedieanum); 12148 (chenopodioides); 12427 (triflorum); 12842 (tweedieanum); 12851 (tweedieanum); 12872 (echegarayi); 12932 (salamancae); 12975 (cochabambense); 12992 (tripartitum); 13102 (weddellii); 13452 (aloysiifolium); 13579 (salicifolium); 13595 (aloysiifolium); 13596 (aloysiifolium); 14179 (aloysiifolium); 14423 (weddellii); 14536 (aloysiifolium); 14549 (salamancae); 15242 (triflorum); 15919 (sinuatirecurvum); 16204 (sinuatirecurvum); 16214 (weddellii); 16226 (gilioides); 16360 (gilioides); 16415 (tiinae).

Zurita, E. 395 (leptocaulon).

Zygadlo, J.A. 1 (chenopodioides); 2 (chenopodioides); 11 (salicifolium); 26 (chenopodioides); 27 (chenopodioides); 84 (aloysiifolium); 86 (tripartitum); 88 (palitans); 90 (tripartitum); 94 (aloysiifolium); 106 (chenopodioides); 113 (americanum); 114 (americanum); 124 (tripartitum); 140 (salicifolium); 160 (chenopodioides); 161 (chenopodioides); 165 (aloysiifolium); 228 (palitans).
